# Supplementary material for: Macrovipera lebetinus obtusa Venom and Its Fractions Affect Human Dermal Microvascular Endothelial and Fibrosarcoma Cells
Source: Int J Mol Sci. 2025 Apr 11;26(8):3601. doi: 10.3390/ijms26083601 (PMC12026461; doi:10.3390/ijms26083601)
Supplement: Supplementary file 1 [file ijms-26-03601-s001.zip › Supplementary materials/Supplementary-File2_Mascot-Search-Results.pdf]

Mascot Search Results   Fr1-1

```
User         :
Email        :
Search title : vipera_All_8IDplus4 file:\\Proteomics1\d\data\2105\210505_tliepol_1_68252-68266\68252_68266_lpardo\0_G5\1\1SRef\pdata\1\peaklist.xml
Database     : vipera  (1414 sequences; 347452 residues)
Timestamp    : 5 May 2021 at 09:33:34 GMT
Top Score    : 68 for Mixture 1, DIDB_CERVI + DID4_MACLO
```

Mascot Score Histogram

Protein score is -10*Log(P), where P is the probability that the observed match is a random event.
Protein scores greater than 44 are significant (p<0.05).

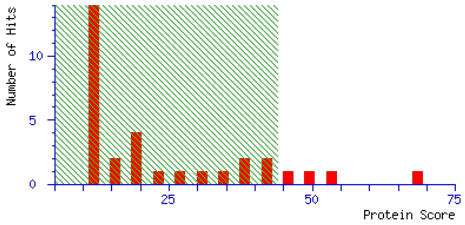

Concise Protein Summary Report

Format As | Concise Protein Summary | Help

Significance threshold p< 0.05   Max. number of hits 10

Re-Search All   Search Unmatched

1. **Mixture 1** **Total score:** 68 **Expect:** 0.0002 **Matches:** 12
   Components (only one family member shown for each component):
   DIDB_CERVI **Mass:** 7584 **Score:** 54 **Expect:** 0.006 **Matches:** 6
   Disintegrin CV-11-beta (Fragment) OS=Cerastes vipera OX=8698 PE=2 SV=1
   DID4_MACLO **Mass:** 7673 **Score:** 48 **Expect:** 0.02 **Matches:** 6
   Disintegrin VLO4 OS=Macrovipera lebetina obtusa OX=209528 PE=1 SV=1

2. DIDB_CERVI **Mass:** 7584 **Score:** 54 **Expect:** 0.006 **Matches:** 6
   Disintegrin CV-11-beta (Fragment) OS=Cerastes vipera OX=8698 PE=2 SV=1
   DIDA_CERVI **Mass:** 12717 **Score:** 33 **Expect:** 0.73 **Matches:** 5
   Disintegrin CV-11-alpha OS=Cerastes vipera OX=8698 PE=2 SV=1

3. DID4_MACLO **Mass:** 7673 **Score:** 48 **Expect:** 0.02 **Matches:** 6
   Disintegrin VLO4 OS=Macrovipera lebetina obtusa OX=209528 PE=1 SV=1
   DID5B_MACLO **Mass:** 8235 **Score:** 15 **Expect:** 48 **Matches:** 2
   Disintegrin VLO5B OS=Macrovipera lebetina obtusa OX=209528 PE=1 SV=1

4. DID1A_MACLB **Mass:** 12718 **Score:** 46 **Expect:** 0.039 **Matches:** 7
   Disintegrin lebein-1-alpha OS=Macrovipera lebetina OX=8709 PE=1 SV=2

5. DID7A_VIPBB **Mass:** 7574 **Score:** 41 **Expect:** 0.11 **Matches:** 5
   Disintegrin VB7A OS=Vipera berus berus OX=31156 PE=1 SV=1

6. VM2A6_VIPAA **Mass:** 7588 **Score:** 41 **Expect:** 0.11 **Matches:** 5
   Disintegrin VA6 OS=Vipera ammodytes ammodytes OX=8705 PE=1 SV=1

7. VM25A_MACLO **Mass:** 7570 **Score:** 38 **Expect:** 0.25 **Matches:** 5
   Disintegrin VLO5A OS=Macrovipera lebetina obtusa OX=209528 PE=1 SV=1

8. A0A6G5ZVR7_9SAUR **Mass:** 9593 **Score:** 37 **Expect:** 0.27 **Matches:** 5
   Disintegrin (Fragment) OS=Vipera anatolica senliki OX=2604287 PE=2 SV=1
   A0A1J0CZN0_VIPAA **Mass:** 12765 **Score:** 34 **Expect:** 0.62 **Matches:** 5
   Dis-2 OS=Vipera ammodytes ammodytes OX=8705 PE=2 SV=1

9. Q1JRG9_MACLN **Mass:** 7698 **Score:** 36 **Expect:** 0.38 **Matches:** 5
   VGD-containing dimeric disintegrin subunit ML-G1 (Fragment) OS=Macrovipera lebetina transmediterranea OX=384075 GN=ml-G1 PE=4 SV=1

10. DID1B_MACLB **Mass:** 7686 **Score:** 29 **Expect:** 1.8 **Matches:** 4
    Disintegrin lebein-1-beta OS=Macrovipera lebetina OX=8709 PE=1 SV=1

Search Parameters

```
Type of search         : Peptide Mass Fingerprint
Enzyme                 : Trypsin
Fixed modifications    : Carbamidomethyl (C)
Variable modifications : Oxidation (M)
Mass values            : Monoisotopic
Protein Mass           : Unrestricted
Peptide Mass Tolerance : ± 100 ppm
Peptide Charge State   : 1+
Max Missed Cleavages   : 1
Number of queries      : 94
Selected for scoring   : 67
```

Mascot: http://www.matrixscience.com/

Mascot Search Results

**User**	:
**Email**	:
**Search title**	: **vipera_all_Lift file:\\Proteomics1\d\data\2105\210505_tliepol_1_68252-68266\68252_68266_1pardo\0_G5\1\0_G5.xml**
**Database**	: **vipera (1414 sequences; 347452 residues)**
**Timestamp**	: **5 May 2021 at 09:42:53 GMT**
**Protein hits**	: DID1A_MACLB Disintegrin lebein-1-alpha OS=Macrovipera lebetina OX=8709 PE=1 SV=2<br>VM2A6_VIPAA Disintegrin VA6 OS=Vipera ammodytes ammodytes OX=8705 PE=1 SV=1<br>Q1JRG9_MACLN VGD-containing dimeric disintegrin subunit ML-G1 (Fragment) OS=Macrovipera lebetina transmediterranea OX=384075 GN=ml-G1 PE=4 SV=1

Mascot Score Histogram

Ions score is -10*Log(P), where P is the probability that the observed match is a random event.
Individual ions scores > 10 indicate identity or extensive homology (p<0.05).
Protein scores are derived from ions scores as a non-probabilistic basis for ranking protein hits.

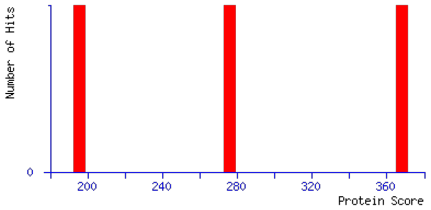

Peptide Summary Report

Format As | Peptide Summary | Help

Significance threshold p< 0.05 | Max. number of hits 10

Standard scoring ○ MudPIT scoring ◉ | Ions score or expect cut-off 0 | Show sub-sets 0

Show pop-ups ◉ Suppress pop-ups ○ | Sort unassigned Decreasing Score | Require bold red ☐

Overview Table

Click on column header to jump to entry in results list.
Move mouse over any indicator to highlight identical peptides.
Click on an indicator to see details of individual match.
Use check boxes to select sub-set of queries for new search.

Mouse over: -Query- | -Accession- | -Sequence-

Hit:	1	2	3
☑ 837.4590 (1+)			
☑ 855.0732 (1+)			
☑ 868.5456 (1+)			
☑ 878.4849 (1+)			
☑ 972.4876 (1+)			
☑ 1010.5007 (1+)			
☑ 1029.5111 (1+)			
☑ 1055.5251 (1+)			
☑ 1074.5893 (1+)			
☑ 1079.5234 (1+)			
☑ 1106.5610 (1+)			
☑ 1108.5781 (1+)			
☑ 1121.6009 (1+)			
☑ 1146.5920 (1+)			
☑ 1148.5748 (1+)			
☑ 1150.5860 (1+)			
☑ 1162.5862 (1+)			
☑ 1165.6008 (1+)	●		
☑ 1176.6034 (1+)			
☑ 1179.6108 (1+)			
☑ 1187.5821 (1+)			
☑ 1191.6161 (1+)			
☑ 1207.6099 (1+)			
☑ 1261.5103 (1+)			
☑ 1277.7239 (1+)			
☑ 1317.5968 (1+)			
☑ 1318.5293 (1+)			
☑ 1319.7004 (1+)			
☑ 1332.5416 (1+)			
☑ 1337.6172 (1+)			
☑ 1410.6020 (1+)			
☑ 1417.6061 (1+)			
☑ 1455.6224 (1+)			
☑ 1459.9017 (1+)			
☑ 1467.6202 (1+)			
☑ 1471.6172 (1+)			
☑ 1474.6300 (1+)	●	●	●
☑ 1478.6090 (1+)			
☑ 1485.6342 (1+)			
☑ 1488.6426 (1+)			
☑ 1500.6438 (1+)			
☑ 1516.6438 (1+)			

☑ 1542.6550 (1+)			
☑ 1567.7594 (1+)			
☑ 1583.6739 (1+)			
☑ 1608.7943 (1+)			
☑ 1647.6980 (1+)			
☑ 1697.7378 (1+)			
☑ 1715.7443 (1+)			
☑ 1742.7547 (1+)			
☑ 1756.7073 (1+)			
☑ 1761.7619 (1+)			
☑ 1763.7522 (1+)			
☑ 1775.7627 (1+)			
☑ 1782.7593 (1+)			
☑ 1813.7391 (1+)			
☑ 1815.7433 (1+)			
☑ 1826.7504 (1+)			
☑ 1829.7585 (1+)			
☑ 1841.7572 (1+)			
☑ 1845.7658 (1+)			
☑ 1853.7546 (1+)			
☑ 1862.7583 (1+)			
☑ 1869.7470 (1+)			
☑ 1872.7671 (1+)			
☑ 1875.7495 (1+)			
☑ 1883.7683 (1+)			
☑ 1886.7775 (1+)			
☑ 1889.7721 (1+)			
☑ 1892.8010 (1+)			
☑ 1904.7710 (1+)			
☑ 1908.7915 (1+)			
☑ 1921.7036 (1+)			
☑ 1923.7691 (1+)			
☑ 1934.7780 (1+)			
☑ 1959.7266 (1+)			
☑ 1962.7504 (1+)	●	●	
☑ 1975.7480 (1+)			
☑ 1978.7390 (1+)	●	●	
☑ 1992.7417 (1+)			
☑ 2004.7552 (1+)			
☑ 2024.7607 (1+)			
☑ 2091.8009 (1+)			
☑ 2094.7873 (1+)			
☑ 2103.8168 (1+)			
☑ 2130.7947 (1+)			
☑ 2133.8410 (1+)			●
☑ 2146.8301 (1+)			
☑ 2149.8290 (1+)			●
☑ 2163.8343 (1+)			
☑ 2175.8409 (1+)			
☑ 2205.8686 (1+)			
☑ 2265.1750 (1+)			
☑ 3346.6915 (1+)			

Select All | Select None | Search Selected | ☐ **Error tolerant** | Archive Report

1. DID1A_MACLB **Mass:** 12718 **Score:** 368 **Matches:** 4(4) **Sequences:** 3(3)
Disintegrin lebein-1-alpha OS=Macrovipera lebetina OX=8709 PE=1 SV=2
☐ Check to include this hit in error tolerant search or archive report

Query	Observed	Mr(expt)	Mr(calc)	ppm	Miss	Score	Expect	Rank	Unique	Peptide
18	1165.6008	1164.5935	1164.5710	19.4	0	71	8.7e-008	1	U	K.FLNAGTICNR.A
37	1474.6300	1473.6227	1473.6024	13.8	1	62	5.8e-007	1		R.RGEHCVSGPCCR.N
77	1962.7504	1961.7432	1961.7190	12.3	0	141	8.7e-015	1	U	R.GDDMNDYCTGISSDCPR.N
79	1978.7390	1977.7317	1977.7139	9.00	0	(133)	4.5e-014	1	U	R.GDDMNDYCTGISSDCPR.N + Oxidation (M)

2. VM2A6_VIPAA **Mass:** 7588 **Score:** 277 **Matches:** 3(3) **Sequences:** 2(2)
Disintegrin VA6 OS=Vipera ammodytes ammodytes OX=8705 PE=1 SV=1
☐ Check to include this hit in error tolerant search or archive report

Query	Observed	Mr(expt)	Mr(calc)	ppm	Miss	Score	Expect	Rank	Unique	Peptide
37	1474.6300	1473.6227	1473.6024	13.8	1	62	5.8e-007	1		R.RGEHCVSGPCCR.N
77	1962.7504	1961.7432	1961.7190	12.3	0	123	4.6e-013	2	U	R.GDDMNDYCTGVTSDCPR.N
79	1978.7390	1977.7317	1977.7139	8.99	0	(117)	2e-012	2	U	R.GDDMNDYCTGVTSDCPR.N + Oxidation (M)

3. Q1JRG9_MACLN **Mass:** 7698 **Score:** 196 **Matches:** 3(3) **Sequences:** 2(2)
VGD-containing dimeric disintegrin subunit ML-G1 (Fragment) OS=Macrovipera lebetina transmediterranea OX=384075 GN=ml-G1 PE=4 SV=1
☐ Check to include this hit in error tolerant search or archive report

Query	Observed	Mr(expt)	Mr(calc)	ppm	Miss	Score	Expect	Rank	Unique	Peptide
37	1474.6300	1473.6227	1473.6024	13.8	1	62	5.8e-007	1		R.RGEHCVSGPCCR.N
87	2133.8410	2132.8337	2132.8085	11.8	0	146	2.4e-015	1	U	R.AVGDDMDDYCTGISSDCPR.N
89	2149.8290	2148.8217	2148.8035	8.48	0	(13)	0.049	1	U	R.AVGDDMDDYCTGISSDCPR.N + Oxidation (M)

Peptide matches not assigned to protein hits: (no details means no match)

	Query	Observed	Mr(expt)	Mr(calc)	ppm	Miss	Score	Expect	Rank	Unique	Peptide
☑	1	*837.4590*	*836.4517*								
☑	2	*855.0732*	*854.0659*								

☑	3	868.5456	867.5384
☑	4	878.4849	877.4776
☑	5	972.4876	971.4803
☑	6	1010.5007	1009.4934
☑	7	1029.5111	1028.5038
☑	8	1055.5251	1054.5178
☑	9	1074.5893	1073.5820
☑	10	1079.5234	1078.5162
☑	11	1106.5610	1105.5537
☑	12	1108.5781	1107.5708
☑	13	1121.6009	1120.5936
☑	14	1146.5920	1145.5847
☑	15	1148.5748	1147.5675
☑	16	1150.5860	1149.5787
☑	17	1162.5862	1161.5789
☑	19	1176.6034	1175.5962
☑	20	1179.6108	1178.6035
☑	21	1187.5821	1186.5749
☑	22	1191.6161	1190.6088
☑	23	1207.6099	1206.6026
☑	24	1261.5103	1260.5031
☑	25	1277.7239	1276.7166
☑	26	1317.5968	1316.5895
☑	27	1318.5293	1317.5220
☑	28	1319.7004	1318.6932
☑	29	1332.5416	1331.5343
☑	30	1337.6172	1336.6099
☑	31	1410.6020	1409.5947
☑	32	1417.6061	1416.5988
☑	33	1455.6224	1454.6151
☑	34	1459.9017	1458.8944
☑	35	1467.6202	1466.6129
☑	36	1471.6172	1470.6099
☑	38	1478.6090	1477.6017
☑	39	1485.6342	1484.6269
☑	40	1488.6426	1487.6354
☑	41	1500.6438	1499.6365
☑	42	1516.6438	1515.6365
☑	43	1542.6550	1541.6477
☑	44	1567.7594	1566.7521
☑	45	1583.6739	1582.6666
☑	46	1608.7943	1607.7870
☑	47	1647.6980	1646.6907
☑	48	1697.7378	1696.7306
☑	49	1715.7443	1714.7370
☑	50	1742.7547	1741.7475
☑	51	1756.7073	1755.7001
☑	52	1761.7619	1760.7546
☑	53	1763.7522	1762.7449
☑	54	1775.7627	1774.7554
☑	55	1782.7593	1781.7520
☑	56	1813.7391	1812.7319
☑	57	1815.7433	1814.7360
☑	58	1826.7504	1825.7431
☑	59	1829.7585	1828.7512
☑	60	1841.7572	1840.7499
☑	61	1845.7658	1844.7586
☑	62	1853.7546	1852.7473
☑	63	1862.7583	1861.7510
☑	64	1869.7470	1868.7397
☑	65	1872.7671	1871.7598
☑	66	1875.7495	1874.7423
☑	67	1883.7683	1882.7610
☑	68	1886.7775	1885.7703
☑	69	1889.7721	1888.7649
☑	70	1892.8010	1891.7937
☑	71	1904.7710	1903.7637
☑	72	1908.7915	1907.7842
☑	73	1921.7036	1920.6963
☑	74	1923.7691	1922.7618
☑	75	1934.7780	1933.7707
☑	76	1959.7266	1958.7194
☑	78	1975.7480	1974.7407
☑	80	1992.7417	1991.7345
☑	81	2004.7552	2003.7480
☑	82	2024.7607	2023.7534
☑	83	2091.8009	2090.7936
☑	84	2094.7873	2093.7800
☑	85	2103.8168	2102.8095
☑	86	2130.7947	2129.7874
☑	88	2146.8301	2145.8228
☑	90	2163.8343	2162.8270
☑	91	2175.8409	2174.8336
☑	92	2205.8686	2204.8614
☑	93	2265.1750	2264.1677
☑	94	3346.6915	3345.6843

Search Parameters

Type of search : MS/MS Ion Search
Enzyme : Trypsin
Fixed modifications : Carbamidomethyl (C)
Variable modifications : Oxidation (M)
Mass values : Monoisotopic
Protein Mass : Unrestricted
Peptide Mass Tolerance : ± 100 ppm
Fragment Mass Tolerance: ± 0.7 Da
Max Missed Cleavages : 1
Instrument type : MALDI-TOF-TOF
Number of queries : 94

Mascot: http://www.matrixscience.com/

Mascot Search Results

```
User          :
Email         :
Search title  : vipera_all_Lift file:\\Proteomics1\d\data\2105\210505_tliepol_1_68252-68266\68252_68266_lpardo\0_G5\1\0_G5.xml
Database      : vipera  (1414 sequences; 347452 residues)
Timestamp     : 5 May 2021 at 09:42:53 GMT
Warning       : A Peptide summary report will usually give a much clearer picture of MS/MS search results.
Top Score     : 323 for DID1A_MACLB, Disintegrin lebein-1-alpha OS=Macrovipera lebetina OX=8709 PE=1 SV=2
```

Mascot Score Histogram

Protein score is -10*Log(P), where P is the probability that the observed match is a random event.
Protein scores greater than 44 are significant (p<0.05).
Protein scores are derived from ions scores as a non-probabilistic basis for ranking protein hits.

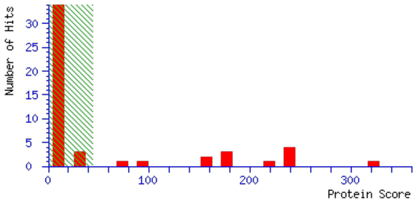

Protein Summary Report

Format As | Protein Summary (deprecated) | Help

Significance threshold p< 0.05 Max. number of hits 20

Overview Table

Click on column header to jump to entry in results list.
Move mouse over any indicator to highlight identical peptides.
Click on an indicator to see details of individual match.
Use check boxes to select sub-set of queries for new search.

Mouse over: -Query- -Accession- -Sequence-

Hit:	1	2	3	4	5	6	7	8	9	10	11	12	13	14	15	16	17	18	19	20
☑ 837.4590 (1+)																				
☑ 855.0732 (1+)																				
☑ 868.5456 (1+)																				
☑ 878.4849 (1+)																				
☑ 972.4876 (1+)																				
☑ 1010.5007 (1+)																				
☑ 1029.5111 (1+)																				
☑ 1055.5251 (1+)																				
☑ 1074.5893 (1+)																				
☑ 1079.5234 (1+)																				
☑ 1106.5610 (1+)																				
☑ 1108.5781 (1+)																				
☑ 1121.6009 (1+)																				
☑ 1146.5920 (1+)																				
☑ 1148.5748 (1+)																				
☑ 1150.5860 (1+)																				
☑ 1162.5862 (1+)																				
☑ 1165.6008 (1+)	●																			
☑ 1176.6034 (1+)																				
☑ 1179.6108 (1+)																				
☑ 1187.5821 (1+)																				
☑ 1191.6161 (1+)																				
☑ 1207.6099 (1+)																				
☑ 1261.5103 (1+)																				
☑ 1277.7239 (1+)																				
☑ 1317.5968 (1+)																				
☑ 1318.5293 (1+)																				
☑ 1319.7004 (1+)																				
☑ 1332.5416 (1+)																				
☑ 1337.6172 (1+)																				
☑ 1410.6020 (1+)																				
☑ 1417.6061 (1+)																				
☑ 1455.6224 (1+)																				
☑ 1459.9017 (1+)																				
☑ 1467.6202 (1+)																				
☑ 1471.6172 (1+)																				
☑ 1474.6300 (1+)	●	●	●	●	●	●						●	●							
☑ 1478.6090 (1+)																				
☑ 1485.6342 (1+)																				
☑ 1488.6426 (1+)																				
☑ 1500.6438 (1+)																				
☑ 1516.6438 (1+)																				
☑ 1542.6550 (1+)																				
☑ 1567.7594 (1+)																				
☑ 1583.6739 (1+)																				
☑ 1608.7943 (1+)																				
☑ 1647.6980 (1+)																				
☑ 1697.7378 (1+)																				
☑ 1715.7443 (1+)																				

☑ 1742.7547 (1+)																				
☑ 1756.7073 (1+)																				
☑ 1761.7619 (1+)																				
☑ 1763.7522 (1+)																				
☑ 1775.7627 (1+)																				
☑ 1782.7593 (1+)																				
☑ 1813.7391 (1+)																				
☑ 1815.7433 (1+)																				
☑ 1826.7504 (1+)																				
☑ 1829.7585 (1+)																				
☑ 1841.7572 (1+)																				
☑ 1845.7658 (1+)																				
☑ 1853.7546 (1+)																				
☑ 1862.7583 (1+)																				
☑ 1869.7470 (1+)																				
☑ 1872.7671 (1+)																				
☑ 1875.7495 (1+)																				
☑ 1883.7683 (1+)																				
☑ 1886.7775 (1+)																				
☑ 1889.7721 (1+)																				
☑ 1892.8010 (1+)																				
☑ 1904.7710 (1+)																				
☑ 1908.7915 (1+)																				
☑ 1921.7036 (1+)																				
☑ 1923.7691 (1+)																				
☑ 1934.7780 (1+)																				
☑ 1959.7266 (1+)																				
☑ 1962.7504 (1+)	●		●	●	●	●	●		●	●										
☑ 1975.7480 (1+)																				
☑ 1978.7390 (1+)	●		●	●	●	●	●		●	●										
☑ 1992.7417 (1+)																				
☑ 2004.7552 (1+)																				
☑ 2024.7607 (1+)																				
☑ 2091.8009 (1+)																				
☑ 2094.7873 (1+)																				
☑ 2103.8168 (1+)																				
☑ 2130.7947 (1+)																				
☑ 2133.8410 (1+)		●						●			●									
☑ 2146.8301 (1+)																				
☑ 2149.8290 (1+)		●						●			●									
☑ 2163.8343 (1+)																				
☑ 2175.8409 (1+)																				
☑ 2205.8686 (1+)																				
☑ 2265.1750 (1+)																				
☑ 3346.6915 (1+)																				

Select All Select None Search Selected

Index

	Accession	Mass	Score	Description
1.	DID1A_MACLB	12718	323	Disintegrin lebein-1-alpha OS=Macrovipera lebetina OX=8709 PE=1 SV=2
2.	Q1JRG9_MACLN	7698	239	VGD-containing dimeric disintegrin subunit ML-G1 (Fragment) OS=Macrovipera lebetina transmediterranea OX=384075 GN=ml-G1 PE=4 SV=1
3.	DID7A_VIPBB	7574	238	Disintegrin VB7A OS=Vipera berus berus OX=31156 PE=1 SV=1
4.	A0A6G5ZVR7_9SAUR	9593	235	Disintegrin (Fragment) OS=Vipera anatolica senliki OX=2604287 PE=2 SV=1
5.	A0A1J0CZN0_VIPAA	12765	231	Dis-2 OS=Vipera ammodytes OX=8705 PE=2 SV=1
6.	VM2A6_VIPAA	7588	221	Disintegrin VA6 OS=Vipera ammodytes ammodytes OX=8705 PE=1 SV=1
7.	DIDB_CERVI	7584	187	Disintegrin CV-11-beta (Fragment) OS=Cerastes vipera OX=8698 PE=2 SV=1
8.	VM25A_MACLO	7570	178	Disintegrin VLO5A OS=Macrovipera lebetina obtusa OX=209528 PE=1 SV=1
9.	DIDA_CERVI	12717	168	Disintegrin CV-11-alpha OS=Cerastes vipera OX=8698 PE=2 SV=1
10.	DID1B_MACLB	7686	165	Disintegrin lebein-1-beta OS=Macrovipera lebetina OX=8709 PE=1 SV=1
11.	VM2L2_MACLB	54586	155	Zinc metalloproteinase/disintegrin OS=Macrovipera lebetina OX=8709 PE=1 SV=1
12.	DID4_MACLO	7673	104	Disintegrin VLO4 OS=Macrovipera lebetina obtusa OX=209528 PE=1 SV=1
13.	DID5B_MACLO	8235	76	Disintegrin VLO5B OS=Macrovipera lebetina obtusa OX=209528 PE=1 SV=1
14.	A0A6G5ZUN8_9SAUR	61639	25	Atriopeptidase (Fragment) OS=Vipera anatolica senliki OX=2604287 PE=2 SV=1
15.	PA2AB_DABSI	15673	21	Acidic phospholipase A2 daboiatoxin B chain (Fragment) OS=Daboia siamensis OX=343250 PE=1 SV=1
16.	Q6A339_VIPAZ	16488	21	Vaspin basic subunit OS=Vipera aspis zinnikeri OX=55427 PE=2 SV=1
17.	A0A6G5ZW48_9SAUR	86121	20	Atriopeptidase OS=Vipera anatolica senliki OX=2604287 PE=2 SV=1
18.	A0A223PK48_DABRR	27702	18	Serotriflin (Fragment) OS=Daboia russelii OX=8707 PE=2 SV=1
19.	PA2B5_DABRR	14376	18	Basic phospholipase A2 VRV-PL-V OS=Daboia russelii OX=8707 PE=1 SV=1
20.	PA2B3_DABRR	14476	18	Basic phospholipase A2 3 OS=Daboia russelii OX=8707 PE=1 SV=1

Results List

1. DID1A_MACLB **Mass:** 12718 **Score:** 323 **Expect:** 7.1e-030 **Matches:** 7

Disintegrin lebein-1-alpha OS=Macrovipera lebetina OX=8709 PE=1 SV=2

Observed	Mr(expt)	Mr(calc)	ppm	Start	End	Miss	Ions	Peptide
1165.6008	1164.5935	1164.5710	19.4	78 -	87	0	71	K.FLNAGTICNR.A
1318.5293	1317.5220	1317.5013	15.8	64 -	74	0	---	R.GEHCVSGPCCR.N
1474.6300	1473.6227	1473.6024	13.8	63 -	74	1	62	R.RGEHCVSGPCCR.N
1567.7594	1566.7521	1566.7395	8.04	75 -	87	1	---	R.NCKFLNAGTICNR.A
1962.7504	1961.7432	1961.7190	12.3	90 -	106	0	141	R.GDDMNDYCTGISSDCPR.N
1978.7390	1977.7317	1977.7139	9.00	90 -	106	0	(133)	R.GDDMNDYCTGISSDCPR.N + Oxidation (M)
2205.8686	2204.8614	2204.8521	4.19	88 -	106	1	---	R.ARGDDMNDYCTGISSDCPR.N + Oxidation (M)

No match to: 837.4590, 855.0732, 868.5456, 878.4849, 972.4876, 1010.5007, 1029.5111, 1055.5251, 1074.5893, 1079.5234, 1106.5610, 1108.5781, 1121.6009, 1146.5920, 1148.5748, 1150.5860, 1162.5862, 1176.6034, 1179.6108, 1187.5821, 1191.6161, 1207.6099, 1261.5103, 1277.7239, 1317.5968, 1319.7004, 1332.5416, 1337.6172, 1410.6020, 1417.6061, 1455.6224, 1459.9017, 1467.6202, 1471.6172, 1478.6090, 1485.6342, 1488.6426, 1500.6438, 1516.6438, 1542.6550, 1583.6739, 1608.7943, 1647.6980, 1697.7378, 1715.7443, 1742.7547, 1756.7073, 1761.7619, 1763.7522, 1775.7627, 1782.7593, 1813.7391, 1815.7433, 1826.7504, 1829.7585, 1841.7572, 1845.7658, 1853.7546, 1862.7583, 1869.7470, 1872.7671, 1875.7495, 1883.7683, 1886.7775, 1889.7721, 1892.8010, 1904.7710, 1908.7915, 1921.7036, 1923.7691, 1934.7780, 1959.7266, 1975.7480, 1992.7417, 2004.7552, 2024.7607, 2091.8009, 2094.7873, 2103.8168, 2130.7947, 2133.8410, 2146.8301, 2149.8290, 2163.8343, 2175.8409, 2265.1750, 3346.6915

2. Q1JRG9_MACLN **Mass:** 7698 **Score:** 239 **Expect:** 1.8e-021 **Matches:** 5

VGD-containing dimeric disintegrin subunit ML-G1 (Fragment) OS=Macrovipera lebetina transmediterranea OX=384075 GN=ml-G1 PE=4 SV=1

Observed	Mr(expt)	Mr(calc)	ppm	Start	End	Miss	Ions	Peptide
837.4590	836.4517	836.4327	22.7	29 -	34	1	---	R.NCKFLR.A
1318.5293	1317.5220	1317.5013	15.8	18 -	28	0	---	R.GEHCVSGPCCR.N
1474.6300	1473.6227	1473.6024	13.8	17 -	28	1	62	R.RGEHCVSGPCCR.N
2133.8410	2132.8337	2132.8085	11.8	42 -	60	0	146	R.AVGDDMDDYCTGISSDCPR.N
2149.8290	2148.8217	2148.8035	8.48	42 -	60	0	(13)	R.AVGDDMDDYCTGISSDCPR.N + Oxidation (M)

No match to: 855.0732, 868.5456, 878.4849, 972.4876, 1010.5007, 1029.5111, 1055.5251, 1074.5893, 1079.5234, 1106.5610, 1108.5781, 1121.6009, 1146.5920,

1148.5748, 1150.5860, 1162.5862, 1165.6008, 1176.6034, 1179.6108, 1187.5821, 1191.6161, 1207.6099, 1261.5103, 1277.7239, 1317.5968, 1319.7004, 1332.5416, 1337.6172, 1410.6020, 1417.6061, 1455.6224, 1459.9017, 1467.6202, 1471.6172, 1478.6090, 1485.6342, 1488.6426, 1500.6438, 1516.6438, 1542.6550, 1567.7594, 1583.6739, 1608.7943, 1647.6980, 1697.7378, 1715.7443, 1742.7547, 1756.7073, 1761.7619, 1763.7522, 1775.7627, 1782.7593, 1813.7391, 1815.7433, 1826.7504, 1829.7585, 1841.7572, 1845.7658, 1853.7546, 1862.7583, 1869.7470, 1872.7671, 1875.7495, 1883.7683, 1886.7775, 1889.7721, 1892.8010, 1904.7710, 1908.7915, 1921.7036, 1923.7691, 1934.7780, 1959.7266, 1962.7504, 1975.7480, 1978.7390, 1992.7417, 2004.7552, 2024.7607, 2091.8009, 2094.7873, 2103.8168, 2130.7947, 2146.8301, 2163.8343, 2175.8409, 2205.8686, 2265.1750, 3346.6915

3. DID7A_VIPBB **Mass:** 7574 **Score:** 238 **Expect:** 2.2e-021 **Matches:** 5

Disintegrin VB7A OS=Vipera berus berus OX=31156 PE=1 SV=1

Observed	Mr(expt)	Mr(calc)	ppm	Start	End	Miss	Ions	Peptide
1318.5293	1317.5220	1317.5013	15.8	17 -	27	0	---	R.GEHCVSGPCCR.N
1474.6300	1473.6227	1473.6024	13.8	16 -	27	1	62	R.RGEHCVSGPCCR.N
1761.7619	1760.7546	1760.7393	8.73	1 -	15	0	---	-.NSGNPCCDPVTCKPR.R
1962.7504	1961.7432	1961.7190	12.3	43 -	59	0	141	R.GDDMNDYCTGISSDCPR.N
1978.7390	1977.7317	1977.7139	9.00	43 -	59	0	(133)	R.GDDMNDYCTGISSDCPR.N + Oxidation (M)

No match to: 837.4590, 855.0732, 868.5456, 878.4849, 972.4876, 1010.5007, 1029.5111, 1055.5251, 1074.5893, 1079.5234, 1106.5610, 1108.5781, 1121.6009, 1146.5920, 1148.5748, 1150.5860, 1162.5862, 1165.6008, 1176.6034, 1179.6108, 1187.5821, 1191.6161, 1207.6099, 1261.5103, 1277.7239, 1317.5968, 1319.7004, 1332.5416, 1337.6172, 1410.6020, 1417.6061, 1455.6224, 1459.9017, 1467.6202, 1471.6172, 1478.6090, 1485.6342, 1488.6426, 1500.6438, 1516.6438, 1542.6550, 1567.7594, 1583.6739, 1608.7943, 1647.6980, 1697.7378, 1715.7443, 1742.7547, 1756.7073, 1763.7522, 1775.7627, 1782.7593, 1813.7391, 1815.7433, 1826.7504, 1829.7585, 1841.7572, 1845.7658, 1853.7546, 1862.7583, 1869.7470, 1872.7671, 1875.7495, 1883.7683, 1886.7775, 1889.7721, 1892.8010, 1904.7710, 1908.7915, 1921.7036, 1923.7691, 1934.7780, 1959.7266, 1975.7480, 1992.7417, 2004.7552, 2024.7607, 2091.8009, 2094.7873, 2103.8168, 2130.7947, 2133.8410, 2146.8301, 2149.8290, 2163.8343, 2175.8409, 2205.8686, 2265.1750, 3346.6915

4. A0A6G5ZVR7_9SAUR **Mass:** 9593 **Score:** 235 **Expect:** 4.5e-021 **Matches:** 5

Disintegrin (Fragment) OS=Vipera anatolica senliki OX=2604287 PE=2 SV=1

Observed	Mr(expt)	Mr(calc)	ppm	Start	End	Miss	Ions	Peptide
1318.5293	1317.5220	1317.5013	15.8	35 -	45	0	---	R.GEHCVSGPCCR.N
1474.6300	1473.6227	1473.6024	13.8	34 -	45	1	62	R.RGEHCVSGPCCR.N
1815.7433	1814.7360	1814.8556	-65.88	46 -	60	1	---	R.NCKFLNAGTICQYAR.G
1962.7504	1961.7432	1961.7190	12.3	61 -	77	0	141	R.GDDMNDYCTGISSDCPR.N
1978.7390	1977.7317	1977.7139	9.00	61 -	77	0	(133)	R.GDDMNDYCTGISSDCPR.N + Oxidation (M)

No match to: 837.4590, 855.0732, 868.5456, 878.4849, 972.4876, 1010.5007, 1029.5111, 1055.5251, 1074.5893, 1079.5234, 1106.5610, 1108.5781, 1121.6009, 1146.5920, 1148.5748, 1150.5860, 1162.5862, 1165.6008, 1176.6034, 1179.6108, 1187.5821, 1191.6161, 1207.6099, 1261.5103, 1277.7239, 1317.5968, 1319.7004, 1332.5416, 1337.6172, 1410.6020, 1417.6061, 1455.6224, 1459.9017, 1467.6202, 1471.6172, 1478.6090, 1485.6342, 1488.6426, 1500.6438, 1516.6438, 1542.6550, 1567.7594, 1583.6739, 1608.7943, 1647.6980, 1697.7378, 1715.7443, 1742.7547, 1756.7073, 1761.7619, 1763.7522, 1775.7627, 1782.7593, 1813.7391, 1826.7504, 1829.7585, 1841.7572, 1845.7658, 1853.7546, 1862.7583, 1869.7470, 1872.7671, 1875.7495, 1883.7683, 1886.7775, 1889.7721, 1892.8010, 1904.7710, 1908.7915, 1921.7036, 1923.7691, 1934.7780, 1959.7266, 1975.7480, 1992.7417, 2004.7552, 2024.7607, 2091.8009, 2094.7873, 2103.8168, 2130.7947, 2133.8410, 2146.8301, 2149.8290, 2163.8343, 2175.8409, 2205.8686, 2265.1750, 3346.6915

5. A0A1J0CZN0_VIPAA **Mass:** 12765 **Score:** 231 **Expect:** 1.1e-020 **Matches:** 5

Dis-2 OS=Vipera ammodytes ammodytes OX=8705 PE=2 SV=1

Observed	Mr(expt)	Mr(calc)	ppm	Start	End	Miss	Ions	Peptide
1318.5293	1317.5220	1317.5013	15.8	64 -	74	0	---	R.GEHCVSGPCCR.N
1474.6300	1473.6227	1473.6024	13.8	63 -	74	1	62	R.RGEHCVSGPCCR.N
1815.7433	1814.7360	1814.8556	-65.88	75 -	89	1	---	R.NCKFLNAGTICQYAR.G
1962.7504	1961.7432	1961.7190	12.3	90 -	106	0	141	R.GDDMNDYCTGISSDCPR.N
1978.7390	1977.7317	1977.7139	9.00	90 -	106	0	(133)	R.GDDMNDYCTGISSDCPR.N + Oxidation (M)

No match to: 837.4590, 855.0732, 868.5456, 878.4849, 972.4876, 1010.5007, 1029.5111, 1055.5251, 1074.5893, 1079.5234, 1106.5610, 1108.5781, 1121.6009, 1146.5920, 1148.5748, 1150.5860, 1162.5862, 1165.6008, 1176.6034, 1179.6108, 1187.5821, 1191.6161, 1207.6099, 1261.5103, 1277.7239, 1317.5968, 1319.7004, 1332.5416, 1337.6172, 1410.6020, 1417.6061, 1455.6224, 1459.9017, 1467.6202, 1471.6172, 1478.6090, 1485.6342, 1488.6426, 1500.6438, 1516.6438, 1542.6550, 1567.7594, 1583.6739, 1608.7943, 1647.6980, 1697.7378, 1715.7443, 1742.7547, 1756.7073, 1761.7619, 1763.7522, 1775.7627, 1782.7593, 1813.7391, 1826.7504, 1829.7585, 1841.7572, 1845.7658, 1853.7546, 1862.7583, 1869.7470, 1872.7671, 1875.7495, 1883.7683, 1886.7775, 1889.7721, 1892.8010, 1904.7710, 1908.7915, 1921.7036, 1923.7691, 1934.7780, 1959.7266, 1975.7480, 1992.7417, 2004.7552, 2024.7607, 2091.8009, 2094.7873, 2103.8168, 2130.7947, 2133.8410, 2146.8301, 2149.8290, 2163.8343, 2175.8409, 2205.8686, 2265.1750, 3346.6915

6. VM2A6_VIPAA **Mass:** 7588 **Score:** 221 **Expect:** 1.1e-019 **Matches:** 5

Disintegrin VA6 OS=Vipera ammodytes ammodytes OX=8705 PE=1 SV=1

Observed	Mr(expt)	Mr(calc)	ppm	Start	End	Miss	Ions	Peptide
1318.5293	1317.5220	1317.5013	15.8	17 -	27	0	---	R.GEHCVSGPCCR.N
1474.6300	1473.6227	1473.6024	13.8	16 -	27	1	62	R.RGEHCVSGPCCR.N
1775.7627	1774.7554	1774.7549	0.29	1 -	15	0	---	-.NSANPCCDPVTCKPR.R
1962.7504	1961.7432	1961.7190	12.3	43 -	59	0	123	R.GDDMNDYCTGVTSDCPR.N
1978.7390	1977.7317	1977.7139	8.99	43 -	59	0	(117)	R.GDDMNDYCTGVTSDCPR.N + Oxidation (M)

No match to: 837.4590, 855.0732, 868.5456, 878.4849, 972.4876, 1010.5007, 1029.5111, 1055.5251, 1074.5893, 1079.5234, 1106.5610, 1108.5781, 1121.6009, 1146.5920, 1148.5748, 1150.5860, 1162.5862, 1165.6008, 1176.6034, 1179.6108, 1187.5821, 1191.6161, 1207.6099, 1261.5103, 1277.7239, 1317.5968, 1319.7004, 1332.5416, 1337.6172, 1410.6020, 1417.6061, 1455.6224, 1459.9017, 1467.6202, 1471.6172, 1478.6090, 1485.6342, 1488.6426, 1500.6438, 1516.6438, 1542.6550, 1567.7594, 1583.6739, 1608.7943, 1647.6980, 1697.7378, 1715.7443, 1742.7547, 1756.7073, 1761.7619, 1763.7522, 1782.7593, 1813.7391, 1815.7433, 1826.7504, 1829.7585, 1841.7572, 1845.7658, 1853.7546, 1862.7583, 1869.7470, 1872.7671, 1875.7495, 1883.7683, 1886.7775, 1889.7721, 1892.8010, 1904.7710, 1908.7915, 1921.7036, 1923.7691, 1934.7780, 1959.7266, 1975.7480, 1992.7417, 2004.7552, 2024.7607, 2091.8009, 2094.7873, 2103.8168, 2130.7947, 2133.8410, 2146.8301, 2149.8290, 2163.8343, 2175.8409, 2205.8686, 2265.1750, 3346.6915

7. DIDB_CERVI **Mass:** 7584 **Score:** 187 **Expect:** 2.8e-016 **Matches:** 6

Disintegrin CV-11-beta (Fragment) OS=Cerastes vipera OX=8698 PE=2 SV=1

Observed	Mr(expt)	Mr(calc)	ppm	Start	End	Miss	Ions	Peptide
1150.5860	1149.5787	1149.6216	-37.30	31 -	40	1	---	K.FLSPGTICKK.A
1332.5416	1331.5343	1331.5169	13.1	17 -	27	0	---	R.GEHCISGPCCR.N
1488.6426	1487.6354	1487.6180	11.7	16 -	27	1	---	K.RGEHCISGPCCR.N
1962.7504	1961.7432	1961.7190	12.3	43 -	59	0	141	R.GDDMNDYCTGISSDCPR.N
1978.7390	1977.7317	1977.7139	9.00	43 -	59	0	(133)	R.GDDMNDYCTGISSDCPR.N + Oxidation (M)
2205.8686	2204.8614	2204.8521	4.19	41 -	59	1	---	K.ARGDDMNDYCTGISSDCPR.N + Oxidation (M)

No match to: 837.4590, 855.0732, 868.5456, 878.4849, 972.4876, 1010.5007, 1029.5111, 1055.5251, 1074.5893, 1079.5234, 1106.5610, 1108.5781, 1121.6009, 1146.5920, 1148.5748, 1162.5862, 1165.6008, 1176.6034, 1179.6108, 1187.5821, 1191.6161, 1207.6099, 1261.5103, 1277.7239, 1317.5968, 1318.5293, 1319.7004, 1337.6172, 1410.6020, 1417.6061, 1455.6224, 1459.9017, 1467.6202, 1471.6172, 1474.6300, 1478.6090, 1485.6342, 1500.6438, 1516.6438, 1542.6550, 1567.7594, 1583.6739, 1608.7943, 1647.6980, 1697.7378, 1715.7443, 1742.7547, 1756.7073, 1761.7619, 1763.7522, 1775.7627, 1782.7593, 1813.7391, 1815.7433, 1826.7504, 1829.7585, 1841.7572, 1845.7658, 1853.7546, 1862.7583, 1869.7470, 1872.7671, 1875.7495, 1883.7683, 1886.7775, 1889.7721, 1892.8010, 1904.7710, 1908.7915, 1921.7036, 1923.7691, 1934.7780, 1959.7266, 1975.7480, 1992.7417, 2004.7552, 2024.7607, 2091.8009, 2094.7873, 2103.8168, 2130.7947, 2133.8410, 2146.8301, 2149.8290, 2163.8343, 2175.8409, 2265.1750, 3346.6915

8. VM25A_MACLO **Mass:** 7570 **Score:** 178 **Expect:** 2.2e-015 **Matches:** 5

Disintegrin VLO5A OS=Macrovipera lebetina obtusa OX=209528 PE=1 SV=1

Observed	Mr(expt)	Mr(calc)	ppm	Start	End	Miss	Ions	Peptide
837.4590	836.4517	836.4327	22.7	28 -	33	1	---	R.NCKFLR.A
1029.5111	1028.5038	1028.4822	21.0	16 -	24	1	---	R.RGEHCVSGK.C
1761.7619	1760.7546	1760.7029	29.4	1 -	15	0	---	-.NSGNPCCDPVTCQPR.R
2133.8410	2132.8337	2132.8085	11.8	41 -	59	0	146	R.AVGDDMDDYCTGISSDCPR.N
2149.8290	2148.8217	2148.8035	8.48	41 -	59	0	(13)	R.AVGDDMDDYCTGISSDCPR.N + Oxidation (M)

No match to: 855.0732, 868.5456, 878.4849, 972.4876, 1010.5007, 1055.5251, 1074.5893, 1079.5234, 1106.5610, 1108.5781, 1121.6009, 1146.5920, 1148.5748, 1150.5860, 1162.5862, 1165.6008, 1176.6034, 1179.6108, 1187.5821, 1191.6161, 1207.6099, 1261.5103, 1277.7239, 1317.5968, 1318.5293, 1319.7004, 1332.5416, 1337.6172, 1410.6020, 1417.6061, 1455.6224, 1459.9017, 1467.6202, 1471.6172, 1474.6300, 1478.6090, 1485.6342, 1488.6426, 1500.6438, 1516.6438, 1542.6550, 1567.7594, 1583.6739, 1608.7943, 1647.6980, 1697.7378, 1715.7443, 1742.7547, 1756.7073, 1763.7522, 1775.7627, 1782.7593, 1813.7391, 1815.7433, 1826.7504, 1829.7585, 1841.7572, 1845.7658, 1853.7546, 1862.7583, 1869.7470, 1872.7671, 1875.7495, 1883.7683, 1886.7775, 1889.7721, 1892.8010, 1904.7710, 1908.7915, 1921.7036, 1923.7691, 1934.7780, 1959.7266, 1962.7504, 1975.7480, 1978.7390, 1992.7417, 2004.7552, 2024.7607, 2091.8009, 2094.7873, 2103.8168, 2130.7947, 2146.8301, 2163.8343, 2175.8409, 2205.8686, 2265.1750, 3346.6915

9. DIDA_CERVI **Mass:** 12717 **Score:** 168 **Expect:** 2.2e-014 **Matches:** 5

Disintegrin CV-11-alpha OS=Cerastes vipera OX=8698 PE=2 SV=1

Observed	Mr(expt)	Mr(calc)	ppm	Start	End	Miss	Ions	Peptide
1150.5860	1149.5787	1149.6216	-37.30	78 -	87	1	---	K.FLSPGTICKK.A
1332.5416	1331.5343	1331.5169	13.1	64 -	74	0	---	R.GEHCISGPCCR.N
1488.6426	1487.6354	1487.6180	11.7	63 -	74	1	---	K.RGEHCISGPCCR.N
1962.7504	1961.7432	1961.7190	12.3	90 -	106	0	141	K.GDDMNDYCTGISSDCPR.N
1978.7390	1977.7317	1977.7139	9.00	90 -	106	0	(133)	K.GDDMNDYCTGISSDCPR.N + Oxidation (M)

No match to: 837.4590, 855.0732, 868.5456, 878.4849, 972.4876, 1010.5007, 1029.5111, 1055.5251, 1074.5893, 1079.5234, 1106.5610, 1108.5781, 1121.6009, 1146.5920, 1148.5748, 1162.5862, 1165.6008, 1176.6034, 1179.6108, 1187.5821, 1191.6161, 1207.6099, 1261.5103, 1277.7239, 1317.5968, 1318.5293, 1319.7004, 1337.6172, 1410.6020, 1417.6061, 1455.6224, 1459.9017, 1467.6202, 1471.6172, 1474.6300, 1478.6090, 1485.6342, 1500.6438, 1516.6438, 1542.6550, 1567.7594,

1583.6739, 1608.7943, 1647.6980, 1697.7378, 1715.7443, 1742.7547, 1756.7073, 1761.7619, 1763.7522, 1775.7627, 1782.7593, 1813.7391, 1815.7433, 1826.7504, 1829.7585, 1841.7572, 1845.7658, 1853.7546, 1862.7583, 1869.7470, 1872.7671, 1875.7495, 1883.7683, 1886.7775, 1889.7721, 1892.8010, 1904.7710, 1908.7915, 1921.7036, 1923.7691, 1934.7780, 1959.7266, 1975.7480, 1992.7417, 2004.7552, 2024.7607, 2091.8009, 2094.7873, 2103.8168, 2130.7947, 2133.8410, 2146.8301, 2149.8290, 2163.8343, 2175.8409, 2205.8686, 2265.1750, 3346.6915

10. DID1B_MACLB **Mass:** 7686 **Score:** 165 **Expect:** 4.5e-014 **Matches:** 4

Disintegrin lebein-1-beta OS=Macrovipera lebetina OX=8709 PE=1 SV=1

Observed	Mr(expt)	Mr(calc)	ppm	Start	End	Miss	Ions	Peptide
1761.7619	1760.7546	1760.7393	8.73	1 -	15	0	---	-.NSGNPCCDPVTCKPR.R
1962.7504	1961.7432	1961.7190	12.3	43 -	59	0	141	R.GDDMNDYCTGISSDCPR.N
1978.7390	1977.7317	1977.7139	9.00	43 -	59	0	(133)	R.GDDMNDYCTGISSDCPR.N + Oxidation (M)
2205.8686	2204.8614	2204.8521	4.19	41 -	59	1	---	R.ARGDDMNDYCTGISSDCPR.N + Oxidation (M)

No match to: 837.4590, 855.0732, 868.5456, 878.4849, 972.4876, 1010.5007, 1029.5111, 1055.5251, 1074.5893, 1079.5234, 1106.5610, 1108.5781, 1121.6009, 1146.5920, 1148.5748, 1150.5860, 1162.5862, 1165.6008, 1176.6034, 1179.6108, 1187.5821, 1191.6161, 1207.6099, 1261.5103, 1277.7239, 1317.5968, 1318.5293, 1319.7004, 1332.5416, 1337.6172, 1410.6020, 1417.6061, 1455.6224, 1459.9017, 1467.6202, 1471.6172, 1474.6300, 1478.6090, 1485.6342, 1488.6426, 1500.6438, 1516.6438, 1542.6550, 1567.7594, 1583.6739, 1608.7943, 1647.6980, 1697.7378, 1715.7443, 1742.7547, 1756.7073, 1763.7522, 1775.7627, 1782.7593, 1813.7391, 1815.7433, 1826.7504, 1829.7585, 1841.7572, 1845.7658, 1853.7546, 1862.7583, 1869.7470, 1872.7671, 1875.7495, 1883.7683, 1886.7775, 1889.7721, 1892.8010, 1904.7710, 1908.7915, 1921.7036, 1923.7691, 1934.7780, 1959.7266, 1975.7480, 1992.7417, 2004.7552, 2024.7607, 2091.8009, 2094.7873, 2103.8168, 2130.7947, 2133.8410, 2146.8301, 2149.8290, 2163.8343, 2175.8409, 2265.1750, 3346.6915

11. VM2L2_MACLB **Mass:** 54586 **Score:** 155 **Expect:** 4.5e-013 **Matches:** 5

Zinc metalloproteinase/disintegrin OS=Macrovipera lebetina OX=8709 PE=1 SV=1

Observed	Mr(expt)	Mr(calc)	ppm	Start	End	Miss	Ions	Peptide
837.4590	836.4517	836.4327	22.7	442 -	447	1	---	R.NCKFLR.A
1029.5111	1028.5038	1028.4822	21.0	430 -	438	1	---	R.RGEHCVSGK.C
1162.5862	1161.5789	1161.6281	-42.39	136 -	145	0	---	R.GETYLIEPLK.L
2133.8410	2132.8337	2132.8085	11.8	455 -	473	0	146	R.AVGDDMDDYCTGISSDCPR.N
2149.8290	2148.8217	2148.8035	8.48	455 -	473	0	(13)	R.AVGDDMDDYCTGISSDCPR.N + Oxidation (M)

No match to: 855.0732, 868.5456, 878.4849, 972.4876, 1010.5007, 1055.5251, 1074.5893, 1079.5234, 1106.5610, 1108.5781, 1121.6009, 1146.5920, 1148.5748, 1150.5860, 1165.6008, 1176.6034, 1179.6108, 1187.5821, 1191.6161, 1207.6099, 1261.5103, 1277.7239, 1317.5968, 1318.5293, 1319.7004, 1332.5416, 1337.6172, 1410.6020, 1417.6061, 1455.6224, 1459.9017, 1467.6202, 1471.6172, 1474.6300, 1478.6090, 1485.6342, 1488.6426, 1500.6438, 1516.6438, 1542.6550, 1567.7594, 1583.6739, 1608.7943, 1647.6980, 1697.7378, 1715.7443, 1742.7547, 1756.7073, 1761.7619, 1763.7522, 1775.7627, 1782.7593, 1813.7391, 1815.7433, 1826.7504, 1829.7585, 1841.7572, 1845.7658, 1853.7546, 1862.7583, 1869.7470, 1872.7671, 1875.7495, 1883.7683, 1886.7775, 1889.7721, 1892.8010, 1904.7710, 1908.7915, 1921.7036, 1923.7691, 1934.7780, 1959.7266, 1962.7504, 1975.7480, 1978.7390, 1992.7417, 2004.7552, 2024.7607, 2091.8009, 2094.7873, 2103.8168, 2130.7947, 2146.8301, 2163.8343, 2175.8409, 2205.8686, 2265.1750, 3346.6915

12. DID4_MACLO **Mass:** 7673 **Score:** 104 **Expect:** 5.6e-008 **Matches:** 6

Disintegrin VLO4 OS=Macrovipera lebetina obtusa OX=209528 PE=1 SV=1

Observed	Mr(expt)	Mr(calc)	ppm	Start	End	Miss	Ions	Peptide
1179.6108	1178.6035	1178.6230	-16.52	32 -	41	1	---	K.FLNAGTICKR.A
1318.5293	1317.5220	1317.5013	15.8	18 -	28	0	---	R.GEHCVSGPCCR.N
1474.6300	1473.6227	1473.6024	13.8	17 -	28	1	62	R.RGEHCVSGPCCR.N
1761.7619	1760.7546	1760.7393	8.73	2 -	16	0	---	M.NSGNPCCDPVTCKPR.R
1892.8010	1891.7937	1891.7798	7.39	1 -	16	0	---	-.MNSGNPCCDPVTCKPR.R
1908.7915	1907.7842	1907.7747	4.98	1 -	16	0	---	-.MNSGNPCCDPVTCKPR.R + Oxidation (M)

No match to: 837.4590, 855.0732, 868.5456, 878.4849, 972.4876, 1010.5007, 1029.5111, 1055.5251, 1074.5893, 1079.5234, 1106.5610, 1108.5781, 1121.6009, 1146.5920, 1148.5748, 1150.5860, 1162.5862, 1165.6008, 1176.6034, 1187.5821, 1191.6161, 1207.6099, 1261.5103, 1277.7239, 1317.5968, 1319.7004, 1332.5416, 1337.6172, 1410.6020, 1417.6061, 1455.6224, 1459.9017, 1467.6202, 1471.6172, 1478.6090, 1485.6342, 1488.6426, 1500.6438, 1516.6438, 1542.6550, 1567.7594, 1583.6739, 1608.7943, 1647.6980, 1697.7378, 1715.7443, 1742.7547, 1756.7073, 1763.7522, 1775.7627, 1782.7593, 1813.7391, 1815.7433, 1826.7504, 1829.7585, 1841.7572, 1845.7658, 1853.7546, 1862.7583, 1869.7470, 1872.7671, 1875.7495, 1883.7683, 1886.7775, 1889.7721, 1904.7710, 1921.7036, 1923.7691, 1934.7780, 1959.7266, 1962.7504, 1975.7480, 1978.7390, 1992.7417, 2004.7552, 2024.7607, 2091.8009, 2094.7873, 2103.8168, 2130.7947, 2133.8410, 2146.8301, 2149.8290, 2163.8343, 2175.8409, 2205.8686, 2265.1750, 3346.6915

13. DID5B_MACLO **Mass:** 8235 **Score:** 76 **Expect:** 3.6e-005 **Matches:** 2

Disintegrin VLO5B OS=Macrovipera lebetina obtusa OX=209528 PE=1 SV=1

Observed	Mr(expt)	Mr(calc)	ppm	Start	End	Miss	Ions	Peptide
1318.5293	1317.5220	1317.5013	15.8	18 -	28	0	---	R.GEHCVSGPCCR.N
1474.6300	1473.6227	1473.6024	13.8	17 -	28	1	62	R.RGEHCVSGPCCR.N

No match to: 837.4590, 855.0732, 868.5456, 878.4849, 972.4876, 1010.5007, 1029.5111, 1055.5251, 1074.5893, 1079.5234, 1106.5610, 1108.5781, 1121.6009, 1146.5920, 1148.5748, 1150.5860, 1162.5862, 1165.6008, 1176.6034, 1179.6108, 1187.5821, 1191.6161, 1207.6099, 1261.5103, 1277.7239, 1317.5968, 1319.7004, 1332.5416, 1337.6172, 1410.6020, 1417.6061, 1455.6224, 1459.9017, 1467.6202, 1471.6172, 1478.6090, 1485.6342, 1488.6426, 1500.6438, 1516.6438, 1542.6550, 1567.7594, 1583.6739, 1608.7943, 1647.6980, 1697.7378, 1715.7443, 1742.7547, 1756.7073, 1761.7619, 1763.7522, 1775.7627, 1782.7593, 1813.7391, 1815.7433, 1826.7504, 1829.7585, 1841.7572, 1845.7658, 1853.7546, 1862.7583, 1869.7470, 1872.7671, 1875.7495, 1883.7683, 1886.7775, 1889.7721, 1892.8010, 1904.7710, 1908.7915, 1921.7036, 1923.7691, 1934.7780, 1959.7266, 1962.7504, 1975.7480, 1978.7390, 1992.7417, 2004.7552, 2024.7607, 2091.8009, 2094.7873, 2103.8168, 2130.7947, 2133.8410, 2146.8301, 2149.8290, 2163.8343, 2175.8409, 2205.8686, 2265.1750, 3346.6915

14. A0A6G5ZUN8_9SAUR **Mass:** 61639 **Score:** 25 **Expect:** 4.8 **Matches:** 9

Atriopeptidase (Fragment) OS=Vipera anatolica senliki OX=2604287 PE=2 SV=1

Observed	Mr(expt)	Mr(calc)	ppm	Start	End	Miss	Ions	Peptide
1010.5007	1009.4934	1009.5556	-61.65	354 -	362	1	---	K.SVISKYSAR.E
1108.5781	1107.5708	1107.5342	33.0	59 -	68	1	---	K.TSDCIKSAAR.I
1317.5968	1316.5895	1316.6104	-15.91	372 -	382	0	---	R.YIMDMVSSLSR.E + Oxidation (M)
1516.6438	1515.6365	1515.7542	-77.66	208 -	220	0	---	K.NSSQHVLHVDQPR.L
1567.7594	1566.7521	1566.7599	-5.01	451 -	463	1	---	K.TLEELSWMDAKTK.K + Oxidation (M)
1608.7943	1607.7870	1607.8420	-34.21	394 -	407	1	---	K.VLYGTTSDAAIWRR.C
1853.7546	1852.7473	1852.8183	-38.28	4 -	19	0	---	K.SESQMDITEMNSPKPK.R + 2 Oxidation (M)
1886.7775	1885.7703	1885.9032	-70.52	308 -	322	1	---	R.NFSMEINNKTFDWIK.F
1934.7780	1933.7707	1933.9244	-79.44	221 -	236	1	---	R.LGLPSRDYYECTGLYK.E

No match to: 837.4590, 855.0732, 868.5456, 878.4849, 972.4876, 1029.5111, 1055.5251, 1074.5893, 1079.5234, 1106.5610, 1121.6009, 1146.5920, 1148.5748, 1150.5860, 1162.5862, 1165.6008, 1176.6034, 1179.6108, 1187.5821, 1191.6161, 1207.6099, 1261.5103, 1277.7239, 1318.5293, 1319.7004, 1332.5416, 1337.6172, 1410.6020, 1417.6061, 1455.6224, 1459.9017, 1467.6202, 1471.6172, 1474.6300, 1478.6090, 1485.6342, 1488.6426, 1500.6438, 1542.6550, 1583.6739, 1647.6980, 1697.7378, 1715.7443, 1742.7547, 1756.7073, 1761.7619, 1763.7522, 1775.7627, 1782.7593, 1813.7391, 1815.7433, 1826.7504, 1829.7585, 1841.7572, 1845.7658, 1862.7583, 1869.7470, 1872.7671, 1875.7495, 1883.7683, 1889.7721, 1892.8010, 1904.7710, 1908.7915, 1921.7036, 1923.7691, 1959.7266, 1962.7504, 1975.7480, 1978.7390, 1992.7417, 2004.7552, 2024.7607, 2091.8009, 2094.7873, 2103.8168, 2130.7947, 2133.8410, 2146.8301, 2149.8290, 2163.8343, 2175.8409, 2205.8686, 2265.1750, 3346.6915

15. PA2AB_DABSI **Mass:** 15673 **Score:** 21 **Expect:** 11 **Matches:** 4

Acidic phospholipase A2 daboiatoxin B chain (Fragment) OS=Daboia siamensis OX=343250 PE=1 SV=1

Observed	Mr(expt)	Mr(calc)	ppm	Start	End	Miss	Ions	Peptide
1179.6108	1178.6035	1178.5608	36.3	69 -	77	0	---	K.LVEYSYSYR.T
1261.5103	1260.5031	1260.5591	-44.46	81 -	91	0	---	K.IVCGGDDPCLR.A
1467.6202	1466.6129	1466.6938	-55.14	114 -	124	1	---	K.KYMLYSIFDCK.E
2103.8168	2102.8095	2102.8271	-8.37	115 -	130	1	---	K.YMLYSIFDCKEESDQC.- + Oxidation (M)

No match to: 837.4590, 855.0732, 868.5456, 878.4849, 972.4876, 1010.5007, 1029.5111, 1055.5251, 1074.5893, 1079.5234, 1106.5610, 1108.5781, 1121.6009, 1146.5920, 1148.5748, 1150.5860, 1162.5862, 1165.6008, 1176.6034, 1187.5821, 1191.6161, 1207.6099, 1277.7239, 1317.5968, 1318.5293, 1319.7004, 1332.5416, 1337.6172, 1410.6020, 1417.6061, 1455.6224, 1459.9017, 1471.6172, 1474.6300, 1478.6090, 1485.6342, 1488.6426, 1500.6438, 1516.6438, 1542.6550, 1567.7594, 1583.6739, 1608.7943, 1647.6980, 1697.7378, 1715.7443, 1742.7547, 1756.7073, 1761.7619, 1763.7522, 1775.7627, 1782.7593, 1813.7391, 1815.7433, 1826.7504, 1829.7585, 1841.7572, 1845.7658, 1853.7546, 1862.7583, 1869.7470, 1872.7671, 1875.7495, 1883.7683, 1886.7775, 1889.7721, 1892.8010, 1904.7710, 1908.7915, 1921.7036, 1923.7691, 1934.7780, 1959.7266, 1962.7504, 1975.7480, 1978.7390, 1992.7417, 2004.7552, 2024.7607, 2091.8009, 2094.7873, 2130.7947, 2133.8410, 2146.8301, 2149.8290, 2163.8343, 2175.8409, 2205.8686, 2265.1750, 3346.6915

16. Q6A339_VIPAZ **Mass:** 16488 **Score:** 21 **Expect:** 11 **Matches:** 4

Vaspin basic subunit OS=Vipera aspis zinnikeri OX=55427 PE=2 SV=1

Observed	Mr(expt)	Mr(calc)	ppm	Start	End	Miss	Ions	Peptide
837.4590	836.4517	836.3963	66.2	107 -	113	0	---	R.VAANCFR.Q
1207.6099	1206.6026	1206.5928	8.17	107 -	116	1	---	R.VAANCFRQNK.N
1647.6980	1646.6907	1646.8126	-74.06	72 -	85	1	---	R.GCNPKLAIYSYSFK.K
2091.8009	2090.7936	2090.7816	5.75	54 -	69	1	---	K.DATDRCCFVHDCCYGR.V

No match to: 855.0732, 868.5456, 878.4849, 972.4876, 1010.5007, 1029.5111, 1055.5251, 1074.5893, 1079.5234, 1106.5610, 1108.5781, 1121.6009, 1146.5920, 1148.5748, 1150.5860, 1162.5862, 1165.6008, 1176.6034, 1179.6108, 1187.5821, 1191.6161, 1261.5103, 1277.7239, 1317.5968, 1318.5293, 1319.7004, 1332.5416, 1337.6172, 1410.6020, 1417.6061, 1455.6224, 1459.9017, 1467.6202, 1471.6172, 1474.6300, 1478.6090, 1485.6342, 1488.6426, 1500.6438, 1516.6438, 1542.6550, 1567.7594, 1583.6739, 1608.7943, 1697.7378, 1715.7443, 1742.7547, 1756.7073, 1761.7619, 1763.7522, 1775.7627, 1782.7593, 1813.7391, 1815.7433, 1826.7504, 1829.7585, 1841.7572, 1845.7658, 1853.7546, 1862.7583, 1869.7470, 1872.7671, 1875.7495, 1883.7683, 1886.7775, 1889.7721, 1892.8010, 1904.7710, 1908.7915, 1921.7036, 1923.7691, 1934.7780, 1959.7266, 1962.7504, 1975.7480, 1978.7390, 1992.7417, 2004.7552, 2024.7607, 2094.7873, 2103.8168, 2130.7947, 2133.8410, 2146.8301, 2149.8290, 2163.8343, 2175.8409, 2205.8686, 2265.1750, 3346.6915

17. A0A6G5ZW48_9SAUR **Mass:** 86121 **Score:** 20 **Expect:** 13 **Matches:** 10
Atriopeptidase OS=Vipera anatolica senliki OX=2604287 PE=2 SV=1

Observed	Mr(expt)	Mr(calc)	ppm	Start	End	Miss	Ions	Peptide
1010.5007	1009.4934	1009.5556	-61.65	354 -	362	1	---	K.SVISKYSAR.E
1108.5781	1107.5708	1107.5342	33.0	59 -	68	1	---	K.TSDCIKSAAR.I
1162.5862	1161.5789	1161.6618	-71.40	671 -	680	1	---	K.RLPGLDLNHK.Q
1317.5968	1316.5895	1316.6104	-15.91	372 -	382	0	---	R.YIMDMVSSLSR.E + Oxidation (M)
1516.6438	1515.6365	1515.7542	-77.66	208 -	220	0	---	K.NSSQHVLHVDQPR.L
1567.7594	1566.7521	1566.7599	-5.01	451 -	463	1	---	K.TLEELSWMDAKTK.K + Oxidation (M)
1608.7943	1607.7870	1607.8420	-34.21	394 -	407	1	---	K.VLYGTTSDAAIWRR.C
1853.7546	1852.7473	1852.8183	-38.28	4 -	19	0	---	K.SESQMDITEMNSPKPK.R + 2 Oxidation (M)
1886.7775	1885.7703	1885.9032	-70.52	308 -	322	1	---	R.NFSMEINNKTFDWIK.F
1934.7780	1933.7707	1933.9244	-79.44	221 -	236	1	---	R.LGLPSRDYYECTGLYK.E

No match to: 837.4590, 855.0732, 868.5456, 878.4849, 972.4876, 1029.5111, 1055.5251, 1074.5893, 1079.5234, 1106.5610, 1121.6009, 1146.5920, 1148.5748, 1150.5860, 1165.6008, 1176.6034, 1179.6108, 1187.5821, 1191.6161, 1207.6099, 1261.5103, 1277.7239, 1318.5293, 1319.7004, 1332.5416, 1337.6172, 1410.6020, 1417.6061, 1455.6224, 1459.9017, 1467.6202, 1471.6172, 1474.6300, 1478.6090, 1485.6342, 1488.6426, 1500.6438, 1542.6550, 1583.6739, 1647.6980, 1697.7378, 1715.7443, 1742.7547, 1756.7073, 1761.7619, 1763.7522, 1775.7627, 1782.7593, 1813.7391, 1815.7433, 1826.7504, 1829.7585, 1841.7572, 1845.7658, 1862.7583, 1869.7470, 1872.7671, 1875.7495, 1883.7683, 1889.7721, 1892.8010, 1904.7710, 1908.7915, 1921.7036, 1923.7691, 1959.7266, 1962.7504, 1975.7480, 1978.7390, 1992.7417, 2004.7552, 2024.7607, 2091.8009, 2094.7873, 2103.8168, 2130.7947, 2133.8410, 2146.8301, 2149.8290, 2163.8343, 2175.8409, 2205.8686, 2265.1750, 3346.6915

18. A0A223PK48_DABRR **Mass:** 27702 **Score:** 18 **Expect:** 22 **Matches:** 4
Serotriflin (Fragment) OS=Daboia russelii OX=8707 PE=2 SV=1

Observed	Mr(expt)	Mr(calc)	ppm	Start	End	Miss	Ions	Peptide
1148.5748	1147.5675	1147.5907	-20.23	47 -	57	0	---	R.SVTPTASNMLK.M
1756.7073	1755.7001	1755.8297	-73.85	75 -	89	1	---	R.CSFAHSPQHLRTMGK.L
1921.7036	1920.6963	1920.8247	-66.85	92 -	107	0	---	K.CGENLFMSSHPSPWTR.V + Oxidation (M)
2146.8301	2145.8228	2146.0088	-86.66	90 -	107	1	---	K.LKCGENLFMSSHPSPWTR.V

No match to: 837.4590, 855.0732, 868.5456, 878.4849, 972.4876, 1010.5007, 1029.5111, 1055.5251, 1074.5893, 1079.5234, 1106.5610, 1108.5781, 1121.6009, 1146.5920, 1150.5860, 1162.5862, 1165.6008, 1176.6034, 1179.6108, 1187.5821, 1191.6161, 1207.6099, 1261.5103, 1277.7239, 1317.5968, 1318.5293, 1319.7004, 1332.5416, 1337.6172, 1410.6020, 1417.6061, 1455.6224, 1459.9017, 1467.6202, 1471.6172, 1474.6300, 1478.6090, 1485.6342, 1488.6426, 1500.6438, 1516.6438, 1542.6550, 1567.7594, 1583.6739, 1608.7943, 1647.6980, 1697.7378, 1715.7443, 1742.7547, 1761.7619, 1763.7522, 1775.7627, 1782.7593, 1813.7391, 1815.7433, 1826.7504, 1829.7585, 1841.7572, 1845.7658, 1853.7546, 1862.7583, 1869.7470, 1872.7671, 1875.7495, 1883.7683, 1886.7775, 1889.7721, 1892.8010, 1904.7710, 1908.7915, 1923.7691, 1934.7780, 1959.7266, 1962.7504, 1975.7480, 1978.7390, 1992.7417, 2004.7552, 2024.7607, 2091.8009, 2094.7873, 2103.8168, 2130.7947, 2133.8410, 2149.8290, 2163.8343, 2175.8409, 2205.8686, 2265.1750, 3346.6915

19. PA2B5_DABRR **Mass:** 14376 **Score:** 18 **Expect:** 23 **Matches:** 3
Basic phospholipase A2 VRV-PL-V OS=Daboia russelii OX=8707 PE=1 SV=1

Observed	Mr(expt)	Mr(calc)	ppm	Start	End	Miss	Ions	Peptide
1478.6090	1477.6017	1477.6985	-65.53	106 -	116	0	---	K.IYMLYPDFLCK.G + Oxidation (M)
1697.7378	1696.7306	1696.8416	-65.41	1 -	15	0	---	-.SLLEFGMMILEETGK.L
1889.7721	1888.7649	1888.9467	-96.26	106 -	120	1	---	K.IYMLYPDFLCKGELK.C

No match to: 837.4590, 855.0732, 868.5456, 878.4849, 972.4876, 1010.5007, 1029.5111, 1055.5251, 1074.5893, 1079.5234, 1106.5610, 1108.5781, 1121.6009, 1146.5920, 1148.5748, 1150.5860, 1162.5862, 1165.6008, 1176.6034, 1179.6108, 1187.5821, 1191.6161, 1207.6099, 1261.5103, 1277.7239, 1317.5968, 1318.5293, 1319.7004, 1332.5416, 1337.6172, 1410.6020, 1417.6061, 1455.6224, 1459.9017, 1467.6202, 1471.6172, 1474.6300, 1485.6342, 1488.6426, 1500.6438, 1516.6438, 1542.6550, 1567.7594, 1583.6739, 1608.7943, 1647.6980, 1715.7443, 1742.7547, 1756.7073, 1761.7619, 1763.7522, 1775.7627, 1782.7593, 1813.7391, 1815.7433, 1826.7504, 1829.7585, 1841.7572, 1845.7658, 1853.7546, 1862.7583, 1869.7470, 1872.7671, 1875.7495, 1883.7683, 1886.7775, 1892.8010, 1904.7710, 1908.7915, 1921.7036, 1923.7691, 1934.7780, 1959.7266, 1962.7504, 1975.7480, 1978.7390, 1992.7417, 2004.7552, 2024.7607, 2091.8009, 2094.7873, 2103.8168, 2130.7947, 2133.8410, 2146.8301, 2149.8290, 2163.8343, 2175.8409, 2205.8686, 2265.1750, 3346.6915

20. PA2B3_DABRR **Mass:** 14476 **Score:** 18 **Expect:** 23 **Matches:** 3
Basic phospholipase A2 3 OS=Daboia russelii OX=8707 PE=1 SV=1

Observed	Mr(expt)	Mr(calc)	ppm	Start	End	Miss	Ions	Peptide
1478.6090	1477.6017	1477.6985	-65.53	106 -	116	0	---	K.IYMLYPDFLCK.G + Oxidation (M)
1697.7378	1696.7306	1696.8416	-65.41	1 -	15	0	---	-.SLLEFGMMILEETGK.L
1889.7721	1888.7649	1888.9467	-96.26	106 -	120	1	---	K.IYMLYPDFLCKGELK.C

No match to: 837.4590, 855.0732, 868.5456, 878.4849, 972.4876, 1010.5007, 1029.5111, 1055.5251, 1074.5893, 1079.5234, 1106.5610, 1108.5781, 1121.6009, 1146.5920, 1148.5748, 1150.5860, 1162.5862, 1165.6008, 1176.6034, 1179.6108, 1187.5821, 1191.6161, 1207.6099, 1261.5103, 1277.7239, 1317.5968, 1318.5293, 1319.7004, 1332.5416, 1337.6172, 1410.6020, 1417.6061, 1455.6224, 1459.9017, 1467.6202, 1471.6172, 1474.6300, 1485.6342, 1488.6426, 1500.6438, 1516.6438, 1542.6550, 1567.7594, 1583.6739, 1608.7943, 1647.6980, 1715.7443, 1742.7547, 1756.7073, 1761.7619, 1763.7522, 1775.7627, 1782.7593, 1813.7391, 1815.7433, 1826.7504, 1829.7585, 1841.7572, 1845.7658, 1853.7546, 1862.7583, 1869.7470, 1872.7671, 1875.7495, 1883.7683, 1886.7775, 1892.8010, 1904.7710, 1908.7915, 1921.7036, 1923.7691, 1934.7780, 1959.7266, 1962.7504, 1975.7480, 1978.7390, 1992.7417, 2004.7552, 2024.7607, 2091.8009, 2094.7873, 2103.8168, 2130.7947, 2133.8410, 2146.8301, 2149.8290, 2163.8343, 2175.8409, 2205.8686, 2265.1750, 3346.6915

Search Parameters

Type of search : MS/MS Ion Search
Enzyme : Trypsin
Fixed modifications : Carbamidomethyl (C)
Variable modifications : Oxidation (M)
Mass values : Monoisotopic
Protein Mass : Unrestricted
Peptide Mass Tolerance : ± 100 ppm
Fragment Mass Tolerance: ± 0.7 Da
Max Missed Cleavages : 1
Instrument type : MALDI-TOF-TOF
Query1 (837.4590,1+) : <no title>
Query2 (855.0732,1+) : <no title>
Query3 (868.5456,1+) : <no title>
Query4 (878.4849,1+) : <no title>
Query5 (972.4876,1+) : <no title>
Query6 (1010.5007,1+) : <no title>
Query7 (1029.5111,1+) : <no title>
Query8 (1055.5251,1+) : <no title>
Query9 (1074.5893,1+) : <no title>
Query10 (1079.5234,1+) : <no title>
Query11 (1106.5610,1+) : <no title>
Query12 (1108.5781,1+) : <no title>
Query13 (1121.6009,1+) : <no title>
Query14 (1146.5920,1+) : <no title>
Query15 (1148.5748,1+) : <no title>
Query16 (1150.5860,1+) : <no title>
Query17 (1162.5862,1+) : <no title>
Query18 (1165.6008,1+) : \\PROTEOMICS1\D\DATA\2105\210505_TLIEPOL_1_68252-68266\68252_68266_LPARDO\0_G5\1\1165.6008.LIFT\1SREF\PDATA\1\1R
Query19 (1176.6034,1+) : <no title>
Query20 (1179.6108,1+) : <no title>
Query21 (1187.5821,1+) : <no title>
Query22 (1191.6161,1+) : \\PROTEOMICS1\D\DATA\2105\210505_TLIEPOL_1_68252-68266\68252_68266_LPARDO\0_G5\1\1191.6161.LIFT\1SREF\PDATA\1\1R
Query23 (1207.6099,1+) : <no title>
Query24 (1261.5103,1+) : <no title>
Query25 (1277.7239,1+) : <no title>
Query26 (1317.5968,1+) : <no title>
Query27 (1318.5293,1+) : <no title>
Query28 (1319.7004,1+) : <no title>
Query29 (1332.5416,1+) : <no title>
Query30 (1337.6172,1+) : <no title>
Query31 (1410.6020,1+) : <no title>
Query32 (1417.6061,1+) : <no title>
Query33 (1455.6224,1+) : <no title>
Query34 (1459.9017,1+) : <no title>
Query35 (1467.6202,1+) : <no title>
Query36 (1471.6172,1+) : <no title>
Query37 (1474.6300,1+) : \\PROTEOMICS1\D\DATA\2105\210505_TLIEPOL_1_68252-68266\68252_68266_LPARDO\0_G5\1\1474.6300.LIFT\1SREF\PDATA\1\1R
Query38 (1478.6090,1+) : <no title>
Query39 (1485.6342,1+) : <no title>
Query40 (1488.6426,1+) : <no title>
Query41 (1500.6438,1+) : <no title>
Query42 (1516.6438,1+) : <no title>
Query43 (1542.6550,1+) : <no title>

```
Query44 (1567.7594,1+) : <no title>
Query45 (1583.6739,1+) : <no title>
Query46 (1608.7943,1+) : <no title>
Query47 (1647.6980,1+) : <no title>
Query48 (1697.7378,1+) : <no title>
Query49 (1715.7443,1+) : <no title>
Query50 (1742.7547,1+) : <no title>
Query51 (1756.7073,1+) : <no title>
Query52 (1761.7619,1+) : <no title>
Query53 (1763.7522,1+) : <no title>
Query54 (1775.7627,1+) : <no title>
Query55 (1782.7593,1+) : <no title>
Query56 (1813.7391,1+) : <no title>
Query57 (1815.7433,1+) : <no title>
Query58 (1826.7504,1+) : <no title>
Query59 (1829.7585,1+) : <no title>
Query60 (1841.7572,1+) : <no title>
Query61 (1845.7658,1+) : <no title>
Query62 (1853.7546,1+) : <no title>
Query63 (1862.7583,1+) : <no title>
Query64 (1869.7470,1+) : <no title>
Query65 (1872.7671,1+) : \\PROTEOMICS1\D\DATA\2105\210505_TLIEPOL_1_68252-68266\68252_68266_LPARDO\0_G5\1\1872.7671.LIFT\1SREF\PDATA\1\1R
Query66 (1875.7495,1+) : <no title>
Query67 (1883.7683,1+) : <no title>
Query68 (1886.7775,1+) : <no title>
Query69 (1889.7721,1+) : <no title>
Query70 (1892.8010,1+) : <no title>
Query71 (1904.7710,1+) : <no title>
Query72 (1908.7915,1+) : <no title>
Query73 (1921.7036,1+) : <no title>
Query74 (1923.7691,1+) : <no title>
Query75 (1934.7780,1+) : <no title>
Query76 (1959.7266,1+) : <no title>
Query77 (1962.7504,1+) : \\PROTEOMICS1\D\DATA\2105\210505_TLIEPOL_1_68252-68266\68252_68266_LPARDO\0_G5\1\1962.7504.LIFT\1SREF\PDATA\1\1R
Query78 (1975.7480,1+) : <no title>
Query79 (1978.7390,1+) : \\PROTEOMICS1\D\DATA\2105\210505_TLIEPOL_1_68252-68266\68252_68266_LPARDO\0_G5\1\1978.7390.LIFT\1SREF\PDATA\1\1R
Query80 (1992.7417,1+) : <no title>
Query81 (2004.7552,1+) : <no title>
Query82 (2024.7607,1+) : <no title>
Query83 (2091.8009,1+) : <no title>
Query84 (2094.7873,1+) : <no title>
Query85 (2103.8168,1+) : <no title>
Query86 (2130.7947,1+) : <no title>
Query87 (2133.8410,1+) : \\PROTEOMICS1\D\DATA\2105\210505_TLIEPOL_1_68252-68266\68252_68266_LPARDO\0_G5\1\2133.8410.LIFT\1SREF\PDATA\1\1R
Query88 (2146.8301,1+) : <no title>
Query89 (2149.8290,1+) : \\PROTEOMICS1\D\DATA\2105\210505_TLIEPOL_1_68252-68266\68252_68266_LPARDO\0_G5\1\2149.8290.LIFT\1SREF\PDATA\1\1R
Query90 (2163.8343,1+) : <no title>
Query91 (2175.8409,1+) : <no title>
Query92 (2205.8686,1+) : <no title>
Query93 (2265.1750,1+) : <no title>
Query94 (3346.6915,1+) : <no title>
```

**Mascot:** http://www.matrixscience.com/

Mascot Search Results

Fr2-1

User :
Email :
Search title : vipera_All_8IDplus4 file:\\Proteomics1\d\data\2105\210505_tliepol_1_68252-68266\68252_68266_lpardo\0_H5\1\1SRef\pdata\1\peaklist.xml
Database : vipera (1414 sequences; 347452 residues)
Timestamp : 5 May 2021 at 09:33:49 GMT
Top Score : 71 for Mixture 1, DID1A_MACLB + VM25A_MACLO

Mascot Score Histogram

Protein score is -10*Log(P), where P is the probability that the observed match is a random event.
Protein scores greater than 44 are significant (p<0.05).

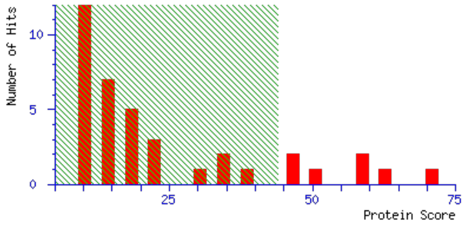

Concise Protein Summary Report

Format As | Concise Protein Summary | Help

Significance threshold p< 0.05 Max. number of hits 10

Re-Search All | Search Unmatched

1. **Mixture 1** **Total score:** 71 **Expect:** 0.00012 **Matches:** 13
Components (only one family member shown for each component):
DID1A_MACLB **Mass:** 12718 **Score:** 57 **Expect:** 0.003 **Matches:** 7
Disintegrin lebein-1-alpha OS=Macrovipera lebetina OX=8709 PE=1 SV=2
VM25A_MACLO **Mass:** 7570 **Score:** 46 **Expect:** 0.032 **Matches:** 6
Disintegrin VLO5A OS=Macrovipera lebetina obtusa OX=209528 PE=1 SV=1

2. Q1JRG9_MACLN **Mass:** 7698 **Score:** 61 **Expect:** 0.0012 **Matches:** 9
VGD-containing dimeric disintegrin subunit ML-G1 (Fragment) OS=Macrovipera lebetina transmediterranea OX=384075 GN=ml-G1 PE=4 SV=1
DID5B_MACLO **Mass:** 8235 **Score:** 32 **Expect:** 0.83 **Matches:** 5
Disintegrin VLO5B OS=Macrovipera lebetina obtusa OX=209528 PE=1 SV=1

3. **Mixture 2** **Total score:** 60 **Expect:** 0.0015 **Matches:** 11
Components (only one family member shown for each component):
VM25A_MACLO **Mass:** 7570 **Score:** 46 **Expect:** 0.032 **Matches:** 6
Disintegrin VLO5A OS=Macrovipera lebetina obtusa OX=209528 PE=1 SV=1
A0A6G5ZVR7_9SAUR **Mass:** 9593 **Score:** 46 **Expect:** 0.035 **Matches:** 5
Disintegrin (Fragment) OS=Vipera anatolica senliki OX=2604287 PE=2 SV=1

4. DID1A_MACLB **Mass:** 12718 **Score:** 57 **Expect:** 0.003 **Matches:** 7
Disintegrin lebein-1-alpha OS=Macrovipera lebetina OX=8709 PE=1 SV=2
NTF4_MACLB **Mass:** 4819 **Score:** 10 **Expect:** 1.3e+002 **Matches:** 1
Neurotrophin-4 (Fragment) OS=Macrovipera lebetina OX=8709 GN=NTF4 PE=3 SV=1

5. DID7A_VIPBB **Mass:** 7574 **Score:** 50 **Expect:** 0.014 **Matches:** 5
Disintegrin VB7A OS=Vipera berus berus OX=31156 PE=1 SV=1

6. VM25A_MACLO **Mass:** 7570 **Score:** 46 **Expect:** 0.032 **Matches:** 6
Disintegrin VLO5A OS=Macrovipera lebetina obtusa OX=209528 PE=1 SV=1
VM2L2_MACLB **Mass:** 54586 **Score:** 13 **Expect:** 74 **Matches:** 5
Zinc metalloproteinase/disintegrin OS=Macrovipera lebetina OX=8709 PE=1 SV=1

7. A0A6G5ZVR7_9SAUR **Mass:** 9593 **Score:** 46 **Expect:** 0.035 **Matches:** 5
Disintegrin (Fragment) OS=Vipera anatolica senliki OX=2604287 PE=2 SV=1
A0A1J0CZN0_VIPAA **Mass:** 12765 **Score:** 42 **Expect:** 0.081 **Matches:** 5
Dis-2 OS=Vipera ammodytes ammodytes OX=8705 PE=2 SV=1

8. DIDB_CERVI **Mass:** 7584 **Score:** 37 **Expect:** 0.31 **Matches:** 6
Disintegrin CV-11-beta (Fragment) OS=Cerastes vipera OX=8698 PE=2 SV=1
DIDA_CERVI **Mass:** 12717 **Score:** 18 **Expect:** 24 **Matches:** 4
Disintegrin CV-11-alpha OS=Cerastes vipera OX=8698 PE=2 SV=1

9. DID1B_MACLB **Mass:** 7686 **Score:** 35 **Expect:** 0.41 **Matches:** 4
Disintegrin lebein-1-beta OS=Macrovipera lebetina OX=8709 PE=1 SV=1

10. VM2A6_VIPAA **Mass:** 7588 **Score:** 35 **Expect:** 0.49 **Matches:** 5
Disintegrin VA6 OS=Vipera ammodytes ammodytes OX=8705 PE=1 SV=1

Search Parameters

Type of search : Peptide Mass Fingerprint
Enzyme : Trypsin
Fixed modifications : Carbamidomethyl (C)
Variable modifications : Oxidation (M)
Mass values : Monoisotopic
Protein Mass : Unrestricted
Peptide Mass Tolerance : ± 100 ppm
Peptide Charge State : 1+
Max Missed Cleavages : 1
Number of queries : 99
Selected for scoring : 40

**Mascot:** http://www.matrixscience.com/

Mascot Search Results

User :
Email :
Search title : **vipera_all_Lift file:\\Proteomics1\d\data\2105\210505_tliepol_1_68252-68266\68252_68266_lpardo\0_H5\1\0_H5.xml**
Database : **vipera (1414 sequences; 347452 residues)**
Timestamp : **5 May 2021 at 09:44:29 GMT**
Protein hits : Q1JRG9_MACLN VGD-containing dimeric disintegrin subunit ML-G1 (Fragment) OS=Macrovipera lebetina transmediterranea OX=384075 GN=ml-G1 PE=4 SV=1
DID1A_MACLB Disintegrin lebein-1-alpha OS=Macrovipera lebetina OX=8709 PE=1 SV=2
VM2A6_VIPAA Disintegrin VA6 OS=Vipera ammodytes ammodytes OX=8705 PE=1 SV=1
A0A223PK45_DABRR Group 6 secretory phospholipase A2 (Fragment) OS=Daboia russelii OX=8707 PE=2 SV=1

Mascot Score Histogram

Ions score is -10*Log(P), where P is the probability that the observed match is a random event.
Individual ions scores > 10 indicate identity or extensive homology (p<0.05).
Protein scores are derived from ions scores as a non-probabilistic basis for ranking protein hits.

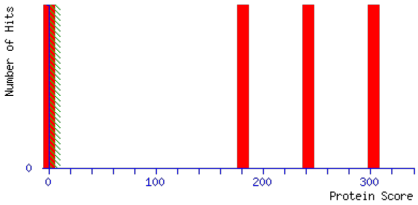

Peptide Summary Report

Format As: Peptide Summary — Help

Significance threshold p< 0.05 — Max. number of hits 10

Standard scoring ○ MudPIT scoring ◉ Ions score or expect cut-off 0 — Show sub-sets 0

Show pop-ups ◉ Suppress pop-ups ○ Sort unassigned Decreasing Score — Require bold red ☐

Overview Table

Click on column header to jump to entry in results list.
Move mouse over any indicator to highlight identical peptides.
Click on an indicator to see details of individual match.
Use check boxes to select sub-set of queries for new search.

Mouse over: -Query- -Accession- -Sequence-

Hit:	1	2	3	4
☑ 837.4636 (1+)				
☑ 855.4421 (1+)				
☑ 857.4530 (1+)				
☑ 878.4896 (1+)				
☑ 972.4927 (1+)				
☑ 1029.5150 (1+)				
☑ 1051.4952 (1+)				
☑ 1055.5298 (1+)				
☑ 1067.9243 (1+)				
☑ 1074.5970 (1+)				
☑ 1108.5827 (1+)				
☑ 1121.6016 (1+)				
☑ 1146.5961 (1+)				
☑ 1150.5950 (1+)				
☑ 1163.5883 (1+)				
☑ 1165.6056 (1+)		●		
☑ 1176.6072 (1+)				
☑ 1179.6184 (1+)				
☑ 1191.6215 (1+)				
☑ 1207.6166 (1+)				
☑ 1261.5141 (1+)				
☑ 1318.5339 (1+)				
☑ 1410.6084 (1+)				
☑ 1417.6150 (1+)				
☑ 1455.6294 (1+)				
☑ 1467.6290 (1+)				
☑ 1474.6361 (1+)	●	●	●	
☑ 1488.6485 (1+)				
☑ 1500.6513 (1+)				
☑ 1516.6512 (1+)				
☑ 1546.7027 (1+)				
☑ 1567.7723 (1+)				
☑ 1608.7992 (1+)				
☑ 1647.7029 (1+)				
☑ 1724.7331 (1+)				
☑ 1756.7135 (1+)				●
☑ 1761.7616 (1+)				
☑ 1770.7319 (1+)				
☑ 1781.7622 (1+)				
☑ 1797.7334 (1+)				
☑ 1812.7381 (1+)				
☑ 1815.7513 (1+)				
☑ 1823.7408 (1+)				

☑ 1826.7562 (1+)				
☑ 1829.7702 (1+)				
☑ 1868.7659 (1+)				
☑ 1872.7738 (1+)				
☑ 1883.7702 (1+)				
☑ 1886.7890 (1+)				
☑ 1892.8123 (1+)				
☑ 1895.7442 (1+)				
☑ 1899.7901 (1+)				
☑ 1902.7794 (1+)				
☑ 1905.7432 (1+)				
☑ 1910.7142 (1+)				
☑ 1914.7672 (1+)				
☑ 1918.7763 (1+)				
☑ 1934.8110 (1+)				
☑ 1945.7541 (1+)				
☑ 1962.7609 (1+)		●	●	
☑ 1975.7763 (1+)				
☑ 1978.7508 (1+)				
☑ 1992.7598 (1+)				
☑ 2004.7669 (1+)				
☑ 2017.7917 (1+)				
☑ 2024.7746 (1+)				
☑ 2032.8253 (1+)				
☑ 2042.8378 (1+)				
☑ 2045.8362 (1+)				
☑ 2059.8188 (1+)				
☑ 2073.8169 (1+)				
☑ 2076.8302 (1+)				
☑ 2085.8450 (1+)				
☑ 2087.8275 (1+)				
☑ 2089.8494 (1+)				
☑ 2092.8140 (1+)				
☑ 2094.8053 (1+)				
☑ 2103.8398 (1+)				
☑ 2116.8347 (1+)				
☑ 2119.8331 (1+)				
☑ 2128.9533 (1+)				
☑ 2131.8331 (1+)				
☑ 2133.8520 (1+)	●			
☑ 2146.8641 (1+)				
☑ 2149.8433 (1+)	●			
☑ 2155.8348 (1+)				
☑ 2159.8681 (1+)				
☑ 2163.8504 (1+)				
☑ 2166.8197 (1+)				
☑ 2171.7913 (1+)				
☑ 2175.8612 (1+)				
☑ 2189.8881 (1+)				
☑ 2227.0026 (1+)				
☑ 2245.0130 (1+)				
☑ 2259.0228 (1+)				
☑ 2277.8956 (1+)				
☑ 2321.8875 (1+)				
☑ 2359.0533 (1+)				
☑ 2887.3127 (1+)				

Select All | Select None | Search Selected | ☐ **Error tolerant** | Archive Report

1. Q1JRG9_MACLN **Mass:** 7698 **Score:** 302 **Matches:** 3(3) **Sequences:** 2(2)
VGD-containing dimeric disintegrin subunit ML-G1 (Fragment) OS=Macrovipera lebetina transmediterranea OX=384075 GN=ml-G1 PE=4 SV=1
☐ Check to include this hit in error tolerant search or archive report

Query	Observed	Mr(expt)	Mr(calc)	ppm	Miss	Score	Expect	Rank	Unique	Peptide
27	1474.6361	1473.6289	1473.6024	18.0	1	60	9.9e-007	1		R.RGEHCVSGPCCR.N
83	2133.8520	2132.8447	2132.8085	16.9	0	151	8.2e-016	1	U	R.AVGDDMDDYCTGISSDCPR.N
85	2149.8433	2148.8360	2148.8035	15.2	0	(117)	1.8e-012	1	U	R.AVGDDMDDYCTGISSDCPR.N + Oxidation (M)

2. DID1A_MACLB **Mass:** 12718 **Score:** 248 **Matches:** 3(3) **Sequences:** 3(3)
Disintegrin lebein-1-alpha OS=Macrovipera lebetina OX=8709 PE=1 SV=2
☐ Check to include this hit in error tolerant search or archive report

Query	Observed	Mr(expt)	Mr(calc)	ppm	Miss	Score	Expect	Rank	Unique	Peptide
16	1165.6056	1164.5983	1164.5710	23.5	0	70	1.1e-007	1	U	K.FLNAGTICNR.A
27	1474.6361	1473.6289	1473.6024	18.0	1	60	9.9e-007	1		R.RGEHCVSGPCCR.N
60	1962.7609	1961.7536	1961.7190	17.6	0	144	4e-015	1	U	R.GDDMNDYCTGISSDCPR.N

3. VM2A6_VIPAA **Mass:** 7588 **Score:** 173 **Matches:** 2(2) **Sequences:** 2(2)
Disintegrin VA6 OS=Vipera ammodytes ammodytes OX=8705 PE=1 SV=1
☐ Check to include this hit in error tolerant search or archive report

Query	Observed	Mr(expt)	Mr(calc)	ppm	Miss	Score	Expect	Rank	Unique	Peptide
27	1474.6361	1473.6289	1473.6024	18.0	1	60	9.9e-007	1		R.RGEHCVSGPCCR.N
60	1962.7609	1961.7536	1961.7190	17.6	0	126	2.3e-013	2	U	R.GDDMNDYCTGVTSDCPR.N

4. A0A223PK45_DABRR **Mass:** 89589 **Score:** 0 **Matches:** 1(0) **Sequences:** 1(0)
Group 6 secretory phospholipase A2 (Fragment) OS=Daboia russelii OX=8707 PE=2 SV=1
☐ Check to include this hit in error tolerant search or archive report

Query	Observed	Mr(expt)	Mr(calc)	ppm	Miss	Score	Expect	Rank	Unique	Peptide

36	1756.7135	1755.7062	1755.7497	-24.80	0	5	0.32	1	U	R.AAAWCEMIDAHYFR.F + Oxidation (M)

Peptide matches not assigned to protein hits: (no details means no match)

	Query	Observed	Mr(expt)	Mr(calc)	ppm	Miss	Score	Expect	Rank	Unique	Peptide
☑	1	837.4636	836.4564								
☑	2	855.4421	854.4348								
☑	3	857.4530	856.4458								
☑	4	878.4896	877.4823								
☑	5	972.4927	971.4855								
☑	6	1029.5150	1028.5078								
☑	7	1051.4952	1050.4879								
☑	8	1055.5298	1054.5225								
☑	9	1067.9243	1066.9171								
☑	10	1074.5970	1073.5898								
☑	11	1108.5827	1107.5754								
☑	12	1121.6016	1120.5943								
☑	13	1146.5961	1145.5888								
☑	14	1150.5950	1149.5878								
☑	15	1163.5883	1162.5810								
☑	17	1176.6072	1175.6000								
☑	18	1179.6184	1178.6111								
☑	19	1191.6215	1190.6143								
☑	20	1207.6166	1206.6094								
☑	21	1261.5141	1260.5068								
☑	22	1318.5339	1317.5266								
☑	23	1410.6084	1409.6011								
☑	24	1417.6150	1416.6078								
☑	25	1455.6294	1454.6221								
☑	26	1467.6290	1466.6217								
☑	28	1488.6485	1487.6412								
☑	29	1500.6513	1499.6440								
☑	30	1516.6512	1515.6439								
☑	31	1546.7027	1545.6954								
☑	32	1567.7723	1566.7650								
☑	33	1608.7992	1607.7919								
☑	34	1647.7029	1646.6956								
☑	35	1724.7331	1723.7258								
☑	37	1761.7616	1760.7543								
☑	38	1770.7319	1769.7246								
☑	39	1781.7622	1780.7550								
☑	40	1797.7334	1796.7262								
☑	41	1812.7381	1811.7309								
☑	42	1815.7513	1814.7440								
☑	43	1823.7408	1822.7336								
☑	44	1826.7562	1825.7490								
☑	45	1829.7702	1828.7629								
☑	46	1868.7659	1867.7586								
☑	47	1872.7738	1871.7666								
☑	48	1883.7702	1882.7629								
☑	49	1886.7890	1885.7817								
☑	50	1892.8123	1891.8050								
☑	51	1895.7442	1894.7370								
☑	52	1899.7901	1898.7828								
☑	53	1902.7794	1901.7721								
☑	54	1905.7432	1904.7359								
☑	55	1910.7142	1909.7070								
☑	56	1914.7672	1913.7599								
☑	57	1918.7763	1917.7691								
☑	58	1934.8110	1933.8038								
☑	59	1945.7541	1944.7468								
☑	61	1975.7763	1974.7690								
☑	62	1978.7508	1977.7435								
☑	63	1992.7598	1991.7525								
☑	64	2004.7669	2003.7596								
☑	65	2017.7917	2016.7844								
☑	66	2024.7746	2023.7673								
☑	67	2032.8253	2031.8181								
☑	68	2042.8378	2041.8305								
☑	69	2045.8362	2044.8289								
☑	70	2059.8188	2058.8115								
☑	71	2073.8169	2072.8096								
☑	72	2076.8302	2075.8229								
☑	73	2085.8450	2084.8377								
☑	74	2087.8275	2086.8202								
☑	75	2089.8494	2088.8421								
☑	76	2092.8140	2091.8067								
☑	77	2094.8053	2093.7980								
☑	78	2103.8398	2102.8326								
☑	79	2116.8347	2115.8274								
☑	80	2119.8331	2118.8258								
☑	81	2128.9533	2127.9460								
☑	82	2131.8331	2130.8259								
☑	84	2146.8641	2145.8568								
☑	86	2155.8348	2154.8275								
☑	87	2159.8681	2158.8608								
☑	88	2163.8504	2162.8431								
☑	89	2166.8197	2165.8124								
☑	90	2171.7913	2170.7840								
☑	91	2175.8612	2174.8540								
☑	92	2189.8881	2188.8809								
☑	93	2227.0026	2225.9953								
☑	94	2245.0130	2244.0058								
☑	95	2259.0228	2258.0155								
☑	96	2277.8956	2276.8883								
☑	97	2321.8875	2320.8802								
☑	98	2359.0533	2358.0461								
☑	99	2887.3127	2886.3055								

Search Parameters

```
Type of search         : MS/MS Ion Search
Enzyme                 : Trypsin
Fixed modifications    : Carbamidomethyl (C)
Variable modifications : Oxidation (M)
Mass values            : Monoisotopic
Protein Mass           : Unrestricted
Peptide Mass Tolerance : ± 100 ppm
Fragment Mass Tolerance: ± 0.7 Da
Max Missed Cleavages   : 1
Instrument type        : MALDI-TOF-TOF
Number of queries      : 99
```

Mascot: http://www.matrixscience.com/

Mascot Search Results

```
User          :
Email         :
Search title  : vipera_all_Lift file:\\Proteomics1\d\data\2105\210505_tliepol_1_68252-68266\68252_68266_lpardo\0_H5\1\0_H5.xml
Database      : vipera  (1414 sequences; 347452 residues)
Timestamp     : 5 May 2021 at 09:44:29 GMT
Warning       : A Peptide summary report will usually give a much clearer picture of MS/MS search results.
Top Score     : 322 for DID1A_MACLB, Disintegrin lebein-1-alpha OS=Macrovipera lebetina OX=8709 PE=1 SV=2
```

Mascot Score Histogram

Protein score is -10*Log(P), where P is the probability that the observed match is a random event.
Protein scores greater than 44 are significant (p<0.05).
Protein scores are derived from ions scores as a non-probabilistic basis for ranking protein hits.

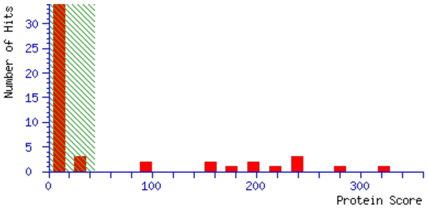

Protein Summary Report

Format As | Protein Summary (deprecated) | Help

Significance threshold p< 0.05 Max. number of hits 10

Overview Table

Click on column header to jump to entry in results list.
Move mouse over any indicator to highlight identical peptides.
Click on an indicator to see details of individual match.
Use check boxes to select sub-set of queries for new search.

Mouse over: -Query-
-Accession- -Sequence-

Hit:	1	2	3	4	5	6	7	8	9	10
☑ 837.4636 (1+)										
☑ 855.4421 (1+)										
☑ 857.4530 (1+)										
☑ 878.4896 (1+)										
☑ 972.4927 (1+)										
☑ 1029.5150 (1+)										
☑ 1051.4952 (1+)										
☑ 1055.5298 (1+)										
☑ 1067.9243 (1+)										
☑ 1074.5970 (1+)										
☑ 1108.5827 (1+)										
☑ 1121.6016 (1+)										
☑ 1146.5961 (1+)										
☑ 1150.5950 (1+)										
☑ 1163.5883 (1+)										
☑ 1165.6056 (1+)	●									
☑ 1176.6072 (1+)										
☑ 1179.6184 (1+)										
☑ 1191.6215 (1+)										
☑ 1207.6166 (1+)										
☑ 1261.5141 (1+)										
☑ 1318.5339 (1+)										
☑ 1410.6084 (1+)										
☑ 1417.6150 (1+)										
☑ 1455.6294 (1+)										
☑ 1467.6290 (1+)										
☑ 1474.6361 (1+)	●	●	●	●	●	●				
☑ 1488.6485 (1+)										
☑ 1500.6513 (1+)										
☑ 1516.6512 (1+)										
☑ 1546.7027 (1+)										
☑ 1567.7723 (1+)										
☑ 1608.7992 (1+)										
☑ 1647.7029 (1+)										
☑ 1724.7331 (1+)										
☑ 1756.7135 (1+)										
☑ 1761.7616 (1+)										
☑ 1770.7319 (1+)										
☑ 1781.7622 (1+)										
☑ 1797.7334 (1+)										
☑ 1812.7381 (1+)										

☑ 1815.7513 (1+)										
☑ 1823.7408 (1+)										
☑ 1826.7562 (1+)										
☑ 1829.7702 (1+)										
☑ 1868.7659 (1+)										
☑ 1872.7738 (1+)										
☑ 1883.7702 (1+)										
☑ 1886.7890 (1+)										
☑ 1892.8123 (1+)										
☑ 1895.7442 (1+)										
☑ 1899.7901 (1+)										
☑ 1902.7794 (1+)										
☑ 1905.7432 (1+)										
☑ 1910.7142 (1+)										
☑ 1914.7672 (1+)										
☑ 1918.7763 (1+)										
☑ 1934.8110 (1+)										
☑ 1945.7541 (1+)										
☑ 1962.7609 (1+)	●		●	●	●	●		●	●	●
☑ 1975.7763 (1+)										
☑ 1978.7508 (1+)										
☑ 1992.7598 (1+)										
☑ 2004.7669 (1+)										
☑ 2017.7917 (1+)										
☑ 2024.7746 (1+)										
☑ 2032.8253 (1+)										
☑ 2042.8378 (1+)										
☑ 2045.8362 (1+)										
☑ 2059.8188 (1+)										
☑ 2073.8169 (1+)										
☑ 2076.8302 (1+)										
☑ 2085.8450 (1+)										
☑ 2087.8275 (1+)										
☑ 2089.8494 (1+)										
☑ 2092.8140 (1+)										
☑ 2094.8053 (1+)										
☑ 2103.8398 (1+)										
☑ 2116.8347 (1+)										
☑ 2119.8331 (1+)										
☑ 2128.9533 (1+)										
☑ 2131.8331 (1+)										
☑ 2133.8520 (1+)		●					●			
☑ 2146.8641 (1+)										
☑ 2149.8433 (1+)		●					●			
☑ 2155.8348 (1+)										
☑ 2159.8681 (1+)										
☑ 2163.8504 (1+)										
☑ 2166.8197 (1+)										
☑ 2171.7913 (1+)										
☑ 2175.8612 (1+)										
☑ 2189.8881 (1+)										
☑ 2227.0026 (1+)										
☑ 2245.0130 (1+)										
☑ 2259.0228 (1+)										
☑ 2277.8956 (1+)										
☑ 2321.8875 (1+)										
☑ 2359.0533 (1+)										
☑ 2887.3127 (1+)										

Select All | Select None | Search Selected

Index

	Accession	Mass	Score	Description
1.	DID1A_MACLB	12718	322	Disintegrin lebein-1-alpha OS=Macrovipera lebetina OX=8709 PE=1 SV=2
2.	Q1JRG9_MACLN	7698	276	VGD-containing dimeric disintegrin subunit ML-G1 (Fragment) OS=Macrovipera lebetina transmediterranea OX=384075 GN=ml-G1 PE=4 SV=1
3.	DID7A_VIPBB	7574	238	Disintegrin VB7A OS=Vipera berus berus OX=31156 PE=1 SV=1
4.	A0A6G5ZVR7_9SAUR	9593	235	Disintegrin (Fragment) OS=Vipera anatolica senliki OX=2604287 PE=2 SV=1
5.	A0A1J0CZN0_VIPAA	12765	231	Dis-2 OS=Vipera ammodytes ammodytes OX=8705 PE=2 SV=1
6.	VM2A6_VIPAA	7588	219	Disintegrin VA6 OS=Vipera ammodytes ammodytes OX=8705 PE=1 SV=1
7.	VM25A_MACLO	7570	191	Disintegrin VLO5A OS=Macrovipera lebetina obtusa OX=209528 PE=1 SV=1
8.	DIDB_CERVI	7584	191	Disintegrin CV-11-beta (Fragment) OS=Cerastes vipera OX=8698 PE=2 SV=1
9.	DID1B_MACLB	7686	168	Disintegrin lebein-1-beta OS=Macrovipera lebetina OX=8709 PE=1 SV=1
10.	DIDA_CERVI	12717	162	Disintegrin CV-11-alpha OS=Cerastes vipera OX=8698 PE=2 SV=1

Results List

1. DID1A_MACLB **Mass:** 12718 **Score:** 322 **Expect:** 8.9e-030 **Matches:** 7

Disintegrin lebein-1-alpha OS=Macrovipera lebetina OX=8709 PE=1 SV=2

Observed	Mr(expt)	Mr(calc)	ppm	Start	End	Miss	Ions	Peptide
1165.6056	1164.5983	1164.5710	23.5	78 -	87	0	70	K.FLNAGTICNR.A
1318.5339	1317.5266	1317.5013	19.2	64 -	74	0	---	R.GEHCVSGPCCR.N
1474.6361	1473.6289	1473.6024	18.0	63 -	74	1	60	R.RGEHCVSGPCCR.N
1567.7723	1566.7650	1566.7395	16.3	75 -	87	1	---	R.NCKFLNAGTICNR.A
1962.7609	1961.7536	1961.7190	17.6	90 -	106	0	144	R.GDDMNDYCTGISSDCPR.N

1978.7508	1977.7435	1977.7139	15.0	90 -	106	0	---	R.GDDMNDYCTGISSDCPR.N + Oxidation (M)
2189.8881	2188.8809	2188.8572	10.8	88 -	106	1	---	R.ARGDDMNDYCTGISSDCPR.N

No match to: 837.4636, 855.4421, 857.4530, 878.4896, 972.4927, 1029.5150, 1051.4952, 1055.5298, 1067.9243, 1074.5970, 1108.5827, 1121.6016, 1146.5961, 1150.5950, 1163.5883, 1176.6072, 1179.6184, 1191.6215, 1207.6166, 1261.5141, 1410.6084, 1417.6150, 1455.6294, 1467.6290, 1488.6485, 1500.6513, 1516.6512, 1546.7027, 1608.7992, 1647.7029, 1724.7331, 1756.7135, 1761.7616, 1770.7319, 1781.7622, 1797.7334, 1812.7381, 1815.7513, 1823.7408, 1826.7562, 1829.7702, 1868.7659, 1872.7738, 1883.7702, 1886.7890, 1892.8123, 1895.7442, 1899.7901, 1902.7794, 1905.7432, 1910.7142, 1914.7672, 1918.7763, 1934.8110, 1945.7541, 1975.7763, 1992.7598, 2004.7669, 2017.7917, 2024.7746, 2032.8253, 2042.8378, 2045.8362, 2059.8188, 2073.8169, 2076.8302, 2085.8450, 2087.8275, 2089.8494, 2092.8140, 2094.8053, 2103.8398, 2116.8347, 2119.8331, 2128.9533, 2131.8331, 2133.8520, 2146.8641, 2149.8433, 2155.8348, 2159.8681, 2163.8504, 2166.8197, 2171.7913, 2175.8612, 2227.0026, 2245.0130, 2259.0228, 2277.8956, 2321.8875, 2359.0533, 2887.3127

2. Q1JRG9_MACLN **Mass:** 7698 **Score:** 276 **Expect:** 3.6e-025 **Matches:** 9
VGD-containing dimeric disintegrin subunit ML-G1 (Fragment) OS=Macrovipera lebetina transmediterranea OX=384075 GN=ml-G1 PE=4 SV=1

Observed	Mr(expt)	Mr(calc)	ppm	Start	End	Miss	Ions	Peptide
837.4636	836.4564	836.4327	28.3	29 -	34	1	---	R.NCKFLR.A
1051.4952	1050.4879	1050.5644	-72.81	32 -	40	1	---	K.FLRAGTVCK.R
1318.5339	1317.5266	1317.5013	19.2	18 -	28	0	---	R.GEHCVSGPCCR.N
1474.6361	1473.6289	1473.6024	18.0	17 -	28	1	60	R.RGEHCVSGPCCR.N
1945.7541	1944.7468	1944.8717	-64.20	2 -	17	1	---	M.NSANPCCDPITCKPRR.G
2076.8302	2075.8229	2075.9121	-42.98	1 -	17	1	---	-.MNSANPCCDPITCKPRR.G
2092.8140	2091.8067	2091.9071	-47.97	1 -	17	1	---	-.MNSANPCCDPITCKPRR.G + Oxidation (M)
2133.8520	2132.8447	2132.8085	16.9	42 -	60	0	151	R.AVGDDMDDYCTGISSDCPR.N
2149.8433	2148.8360	2148.8035	15.2	42 -	60	0	(117)	R.AVGDDMDDYCTGISSDCPR.N + Oxidation (M)

No match to: 855.4421, 857.4530, 878.4896, 972.4927, 1029.5150, 1055.5298, 1067.9243, 1074.5970, 1108.5827, 1121.6016, 1146.5961, 1150.5950, 1163.5883, 1165.6056, 1176.6072, 1179.6184, 1191.6215, 1207.6166, 1261.5141, 1410.6084, 1417.6150, 1455.6294, 1467.6290, 1488.6485, 1500.6513, 1516.6512, 1546.7027, 1567.7723, 1608.7992, 1647.7029, 1724.7331, 1756.7135, 1761.7616, 1770.7319, 1781.7622, 1797.7334, 1812.7381, 1815.7513, 1823.7408, 1826.7562, 1829.7702, 1868.7659, 1872.7738, 1883.7702, 1886.7890, 1892.8123, 1895.7442, 1899.7901, 1902.7794, 1905.7432, 1910.7142, 1914.7672, 1918.7763, 1934.8110, 1962.7609, 1975.7763, 1978.7508, 1992.7598, 2004.7669, 2017.7917, 2024.7746, 2032.8253, 2042.8378, 2045.8362, 2059.8188, 2073.8169, 2085.8450, 2087.8275, 2089.8494, 2094.8053, 2103.8398, 2116.8347, 2119.8331, 2128.9533, 2131.8331, 2146.8641, 2155.8348, 2159.8681, 2163.8504, 2166.8197, 2171.7913, 2175.8612, 2189.8881, 2227.0026, 2245.0130, 2259.0228, 2277.8956, 2321.8875, 2359.0533, 2887.3127

3. DID7A_VIPBB **Mass:** 7574 **Score:** 238 **Expect:** 2.2e-021 **Matches:** 5
Disintegrin VB7A OS=Vipera berus berus OX=31156 PE=1 SV=1

Observed	Mr(expt)	Mr(calc)	ppm	Start	End	Miss	Ions	Peptide
1318.5339	1317.5266	1317.5013	19.2	17 -	27	0	---	R.GEHCVSGPCCR.N
1474.6361	1473.6289	1473.6024	18.0	16 -	27	1	60	R.RGEHCVSGPCCR.N
1761.7616	1760.7543	1760.7393	8.54	1 -	15	0	---	-.NSGNPCCDPVTCKPR.R
1962.7609	1961.7536	1961.7190	17.6	43 -	59	0	144	R.GDDMNDYCTGISSDCPR.N
1978.7508	1977.7435	1977.7139	15.0	43 -	59	0	---	R.GDDMNDYCTGISSDCPR.N + Oxidation (M)

No match to: 837.4636, 855.4421, 857.4530, 878.4896, 972.4927, 1029.5150, 1051.4952, 1055.5298, 1067.9243, 1074.5970, 1108.5827, 1121.6016, 1146.5961, 1150.5950, 1163.5883, 1165.6056, 1176.6072, 1179.6184, 1191.6215, 1207.6166, 1261.5141, 1410.6084, 1417.6150, 1455.6294, 1467.6290, 1488.6485, 1500.6513, 1516.6512, 1546.7027, 1567.7723, 1608.7992, 1647.7029, 1724.7331, 1756.7135, 1770.7319, 1781.7622, 1797.7334, 1812.7381, 1815.7513, 1823.7408, 1826.7562, 1829.7702, 1868.7659, 1872.7738, 1883.7702, 1886.7890, 1892.8123, 1895.7442, 1899.7901, 1902.7794, 1905.7432, 1910.7142, 1914.7672, 1918.7763, 1934.8110, 1945.7541, 1975.7763, 1992.7598, 2004.7669, 2017.7917, 2024.7746, 2032.8253, 2042.8378, 2045.8362, 2059.8188, 2073.8169, 2076.8302, 2085.8450, 2087.8275, 2089.8494, 2092.8140, 2094.8053, 2103.8398, 2116.8347, 2119.8331, 2128.9533, 2131.8331, 2133.8520, 2146.8641, 2149.8433, 2155.8348, 2159.8681, 2163.8504, 2166.8197, 2171.7913, 2175.8612, 2189.8881, 2227.0026, 2245.0130, 2259.0228, 2277.8956, 2321.8875, 2359.0533, 2887.3127

4. A0A6G5ZVR7_9SAUR **Mass:** 9593 **Score:** 235 **Expect:** 4.5e-021 **Matches:** 5
Disintegrin (Fragment) OS=Vipera anatolica senliki OX=2604287 PE=2 SV=1

Observed	Mr(expt)	Mr(calc)	ppm	Start	End	Miss	Ions	Peptide
1318.5339	1317.5266	1317.5013	19.2	35 -	45	0	---	R.GEHCVSGPCCR.N
1474.6361	1473.6289	1473.6024	18.0	34 -	45	1	60	R.RGEHCVSGPCCR.N
1815.7513	1814.7440	1814.8556	-61.46	46 -	60	1	---	R.NCKFLNAGTICQYAR.G
1962.7609	1961.7536	1961.7190	17.6	61 -	77	0	144	R.GDDMNDYCTGISSDCPR.N
1978.7508	1977.7435	1977.7139	15.0	61 -	77	0	---	R.GDDMNDYCTGISSDCPR.N + Oxidation (M)

No match to: 837.4636, 855.4421, 857.4530, 878.4896, 972.4927, 1029.5150, 1051.4952, 1055.5298, 1067.9243, 1074.5970, 1108.5827, 1121.6016, 1146.5961, 1150.5950, 1163.5883, 1165.6056, 1176.6072, 1179.6184, 1191.6215, 1207.6166, 1261.5141, 1410.6084, 1417.6150, 1455.6294, 1467.6290, 1488.6485, 1500.6513, 1516.6512, 1546.7027, 1567.7723, 1608.7992, 1647.7029, 1724.7331, 1756.7135, 1761.7616, 1770.7319, 1781.7622, 1797.7334, 1812.7381, 1823.7408, 1826.7562, 1829.7702, 1868.7659, 1872.7738, 1883.7702, 1886.7890, 1892.8123, 1895.7442, 1899.7901, 1902.7794, 1905.7432, 1910.7142, 1914.7672, 1918.7763, 1934.8110, 1945.7541, 1975.7763, 1992.7598, 2004.7669, 2017.7917, 2024.7746, 2032.8253, 2042.8378, 2045.8362, 2059.8188, 2073.8169, 2076.8302, 2085.8450, 2087.8275, 2089.8494, 2092.8140, 2094.8053, 2103.8398, 2116.8347, 2119.8331, 2128.9533, 2131.8331, 2133.8520, 2146.8641, 2149.8433, 2155.8348, 2159.8681, 2163.8504, 2166.8197, 2171.7913, 2175.8612, 2189.8881, 2227.0026, 2245.0130, 2259.0228, 2277.8956, 2321.8875, 2359.0533, 2887.3127

5. A0A1J0CZN0_VIPAA **Mass:** 12765 **Score:** 231 **Expect:** 1.1e-020 **Matches:** 5
Dis-2 OS=Vipera ammodytes ammodytes OX=8705 PE=2 SV=1

Observed	Mr(expt)	Mr(calc)	ppm	Start	End	Miss	Ions	Peptide
1318.5339	1317.5266	1317.5013	19.2	64 -	74	0	---	R.GEHCVSGPCCR.N
1474.6361	1473.6289	1473.6024	18.0	63 -	74	1	60	R.RGEHCVSGPCCR.N
1815.7513	1814.7440	1814.8556	-61.46	75 -	89	1	---	R.NCKFLNAGTICQYAR.G
1962.7609	1961.7536	1961.7190	17.6	90 -	106	0	144	R.GDDMNDYCTGISSDCPR.N
1978.7508	1977.7435	1977.7139	15.0	90 -	106	0	---	R.GDDMNDYCTGISSDCPR.N + Oxidation (M)

No match to: 837.4636, 855.4421, 857.4530, 878.4896, 972.4927, 1029.5150, 1051.4952, 1055.5298, 1067.9243, 1074.5970, 1108.5827, 1121.6016, 1146.5961, 1150.5950, 1163.5883, 1165.6056, 1176.6072, 1179.6184, 1191.6215, 1207.6166, 1261.5141, 1410.6084, 1417.6150, 1455.6294, 1467.6290, 1488.6485, 1500.6513, 1516.6512, 1546.7027, 1567.7723, 1608.7992, 1647.7029, 1724.7331, 1756.7135, 1761.7616, 1770.7319, 1781.7622, 1797.7334, 1812.7381, 1823.7408, 1826.7562, 1829.7702, 1868.7659, 1872.7738, 1883.7702, 1886.7890, 1892.8123, 1895.7442, 1899.7901, 1902.7794, 1905.7432, 1910.7142, 1914.7672, 1918.7763, 1934.8110, 1945.7541, 1975.7763, 1992.7598, 2004.7669, 2017.7917, 2024.7746, 2032.8253, 2042.8378, 2045.8362, 2059.8188, 2073.8169, 2076.8302, 2085.8450, 2087.8275, 2089.8494, 2092.8140, 2094.8053, 2103.8398, 2116.8347, 2119.8331, 2128.9533, 2131.8331, 2133.8520, 2146.8641, 2149.8433, 2155.8348, 2159.8681, 2163.8504, 2166.8197, 2171.7913, 2175.8612, 2189.8881, 2227.0026, 2245.0130, 2259.0228, 2277.8956, 2321.8875, 2359.0533, 2887.3127

6. VM2A6_VIPAA **Mass:** 7588 **Score:** 219 **Expect:** 1.8e-019 **Matches:** 5
Disintegrin VA6 OS=Vipera ammodytes ammodytes OX=8705 PE=1 SV=1

Observed	Mr(expt)	Mr(calc)	ppm	Start	End	Miss	Ions	Peptide
1051.4952	1050.4879	1050.5280	-38.18	31 -	39	0	---	K.FLNAGTICR.Y
1318.5339	1317.5266	1317.5013	19.2	17 -	27	0	---	R.GEHCVSGPCCR.N
1474.6361	1473.6289	1473.6024	18.0	16 -	27	1	60	R.RGEHCVSGPCCR.N
1962.7609	1961.7536	1961.7190	17.6	43 -	59	0	126	R.GDDMNDYCTGVTSDCPR.N
1978.7508	1977.7435	1977.7139	15.0	43 -	59	0	---	R.GDDMNDYCTGVTSDCPR.N + Oxidation (M)

No match to: 837.4636, 855.4421, 857.4530, 878.4896, 972.4927, 1029.5150, 1055.5298, 1067.9243, 1074.5970, 1108.5827, 1121.6016, 1146.5961, 1150.5950, 1163.5883, 1165.6056, 1176.6072, 1179.6184, 1191.6215, 1207.6166, 1261.5141, 1410.6084, 1417.6150, 1455.6294, 1467.6290, 1488.6485, 1500.6513, 1516.6512, 1546.7027, 1567.7723, 1608.7992, 1647.7029, 1724.7331, 1756.7135, 1761.7616, 1770.7319, 1781.7622, 1797.7334, 1812.7381, 1815.7513, 1823.7408, 1826.7562, 1829.7702, 1868.7659, 1872.7738, 1883.7702, 1886.7890, 1892.8123, 1895.7442, 1899.7901, 1902.7794, 1905.7432, 1910.7142, 1914.7672, 1918.7763, 1934.8110, 1945.7541, 1975.7763, 1992.7598, 2004.7669, 2017.7917, 2024.7746, 2032.8253, 2042.8378, 2045.8362, 2059.8188, 2073.8169, 2076.8302, 2085.8450, 2087.8275, 2089.8494, 2092.8140, 2094.8053, 2103.8398, 2116.8347, 2119.8331, 2128.9533, 2131.8331, 2133.8520, 2146.8641, 2149.8433, 2155.8348, 2159.8681, 2163.8504, 2166.8197, 2171.7913, 2175.8612, 2189.8881, 2227.0026, 2245.0130, 2259.0228, 2277.8956, 2321.8875, 2359.0533, 2887.3127

7. VM25A_MACLO **Mass:** 7570 **Score:** 191 **Expect:** 1.1e-016 **Matches:** 6
Disintegrin VLO5A OS=Macrovipera lebetina obtusa OX=209528 PE=1 SV=1

Observed	Mr(expt)	Mr(calc)	ppm	Start	End	Miss	Ions	Peptide
837.4636	836.4564	836.4327	28.3	28 -	33	1	---	R.NCKFLR.A
1029.5150	1028.5078	1028.4822	24.9	16 -	24	1	---	R.RGEHCVSGK.C
1051.4952	1050.4879	1050.5644	-72.81	31 -	39	1	---	K.FLRAGTVCK.R
1761.7616	1760.7543	1760.7029	29.2	1 -	15	0	---	-.NSGNPCCDPVTCQPR.R
2133.8520	2132.8447	2132.8085	16.9	41 -	59	0	151	R.AVGDDMDDYCTGISSDCPR.N
2149.8433	2148.8360	2148.8035	15.2	41 -	59	0	(117)	R.AVGDDMDDYCTGISSDCPR.N + Oxidation (M)

No match to: 855.4421, 857.4530, 878.4896, 972.4927, 1055.5298, 1067.9243, 1074.5970, 1108.5827, 1121.6016, 1146.5961, 1150.5950, 1163.5883, 1165.6056, 1176.6072, 1179.6184, 1191.6215, 1207.6166, 1261.5141, 1318.5339, 1410.6084, 1417.6150, 1455.6294, 1467.6290, 1474.6361, 1488.6485, 1500.6513, 1516.6512, 1546.7027, 1567.7723, 1608.7992, 1647.7029, 1724.7331, 1756.7135, 1770.7319, 1781.7622, 1797.7334, 1812.7381, 1815.7513, 1823.7408, 1826.7562, 1829.7702, 1868.7659, 1872.7738, 1883.7702, 1886.7890, 1892.8123, 1895.7442, 1899.7901, 1902.7794, 1905.7432, 1910.7142, 1914.7672, 1918.7763, 1934.8110, 1945.7541, 1962.7609, 1975.7763, 1978.7508, 1992.7598, 2004.7669, 2017.7917, 2024.7746, 2032.8253, 2042.8378, 2045.8362, 2059.8188, 2073.8169, 2076.8302, 2085.8450, 2087.8275, 2089.8494, 2092.8140, 2094.8053, 2103.8398, 2116.8347, 2119.8331, 2128.9533, 2131.8331, 2146.8641, 2155.8348, 2159.8681, 2163.8504, 2166.8197, 2171.7913, 2175.8612, 2189.8881, 2227.0026, 2245.0130, 2259.0228, 2277.8956, 2321.8875, 2359.0533, 2887.3127

8. DIDB_CERVI **Mass:** 7584 **Score:** 191 **Expect:** 1.1e-016 **Matches:** 6

Disintegrin CV-11-beta (Fragment) OS=Cerastes vipera OX=8698 PE=2 SV=1

Observed	Mr(expt)	Mr(calc)	ppm	Start	End	Miss	Ions	Peptide
1150.5950	1149.5878	1149.6216	-29.44	31 -	40	1	---	K.FLSPGTICKK.A
1488.6485	1487.6412	1487.6180	15.6	16 -	27	1	---	K.RGEHCISGPCCR.N
1770.7319	1769.7246	1769.7648	-22.69	1 -	15	0	---	-.NSAHPCCDPVTCKPK.R
1962.7609	1961.7536	1961.7190	17.6	43 -	59	0	144	R.GDDMNDYCTGISSDCPR.N
1978.7508	1977.7435	1977.7139	15.0	43 -	59	0	---	R.GDDMNDYCTGISSDCPR.N + Oxidation (M)
2189.8881	2188.8809	2188.8572	10.8	41 -	59	1	---	K.ARGDDMNDYCTGISSDCPR.N

No match to: 837.4636, 855.4421, 857.4530, 878.4896, 972.4927, 1029.5150, 1051.4952, 1055.5298, 1067.9243, 1074.5970, 1108.5827, 1121.6016, 1146.5961, 1163.5883, 1165.6056, 1176.6072, 1179.6184, 1191.6215, 1207.6166, 1261.5141, 1318.5339, 1410.6084, 1417.6150, 1455.6294, 1467.6290, 1474.6361, 1500.6513, 1516.6512, 1546.7027, 1567.7723, 1608.7992, 1647.7029, 1724.7331, 1756.7135, 1761.7616, 1781.7622, 1797.7334, 1812.7381, 1815.7513, 1823.7408, 1826.7562, 1829.7702, 1868.7659, 1872.7738, 1883.7702, 1886.7890, 1892.8123, 1895.7442, 1899.7901, 1902.7794, 1905.7432, 1910.7142, 1914.7672, 1918.7763, 1934.8110, 1945.7541, 1975.7763, 1992.7598, 2004.7669, 2017.7917, 2024.7746, 2032.8253, 2042.8378, 2045.8362, 2059.8188, 2073.8169, 2076.8302, 2085.8450, 2087.8275, 2089.8494, 2092.8140, 2094.8053, 2103.8398, 2116.8347, 2119.8331, 2128.9533, 2131.8331, 2133.8520, 2146.8641, 2149.8433, 2155.8348, 2159.8681, 2163.8504, 2166.8197, 2171.7913, 2175.8612, 2227.0026, 2245.0130, 2259.0228, 2277.8956, 2321.8875, 2359.0533, 2887.3127

9. DID1B_MACLB **Mass:** 7686 **Score:** 168 **Expect:** 2.2e-014 **Matches:** 4

Disintegrin lebein-1-beta OS=Macrovipera lebetina OX=8709 PE=1 SV=1

Observed	Mr(expt)	Mr(calc)	ppm	Start	End	Miss	Ions	Peptide
1761.7616	1760.7543	1760.7393	8.54	1 -	15	0	---	-.NSGNPCCDPVTCKPR.R
1962.7609	1961.7536	1961.7190	17.6	43 -	59	0	144	R.GDDMNDYCTGISSDCPR.N
1978.7508	1977.7435	1977.7139	15.0	43 -	59	0	---	R.GDDMNDYCTGISSDCPR.N + Oxidation (M)
2189.8881	2188.8809	2188.8572	10.8	41 -	59	1	---	R.ARGDDMNDYCTGISSDCPR.N

No match to: 837.4636, 855.4421, 857.4530, 878.4896, 972.4927, 1029.5150, 1051.4952, 1055.5298, 1067.9243, 1074.5970, 1108.5827, 1121.6016, 1146.5961, 1150.5950, 1163.5883, 1165.6056, 1176.6072, 1179.6184, 1191.6215, 1207.6166, 1261.5141, 1318.5339, 1410.6084, 1417.6150, 1455.6294, 1467.6290, 1474.6361, 1488.6485, 1500.6513, 1516.6512, 1546.7027, 1567.7723, 1608.7992, 1647.7029, 1724.7331, 1756.7135, 1770.7319, 1781.7622, 1797.7334, 1812.7381, 1815.7513, 1823.7408, 1826.7562, 1829.7702, 1868.7659, 1872.7738, 1883.7702, 1886.7890, 1892.8123, 1895.7442, 1899.7901, 1902.7794, 1905.7432, 1910.7142, 1914.7672, 1918.7763, 1934.8110, 1945.7541, 1975.7763, 1992.7598, 2004.7669, 2017.7917, 2024.7746, 2032.8253, 2042.8378, 2045.8362, 2059.8188, 2073.8169, 2076.8302, 2085.8450, 2087.8275, 2089.8494, 2092.8140, 2094.8053, 2103.8398, 2116.8347, 2119.8331, 2128.9533, 2131.8331, 2133.8520, 2146.8641, 2149.8433, 2155.8348, 2159.8681, 2163.8504, 2166.8197, 2171.7913, 2175.8612, 2227.0026, 2245.0130, 2259.0228, 2277.8956, 2321.8875, 2359.0533, 2887.3127

10. DIDA_CERVI **Mass:** 12717 **Score:** 162 **Expect:** 8.9e-014 **Matches:** 4

Disintegrin CV-11-alpha OS=Cerastes vipera OX=8698 PE=2 SV=1

Observed	Mr(expt)	Mr(calc)	ppm	Start	End	Miss	Ions	Peptide
1150.5950	1149.5878	1149.6216	-29.44	78 -	87	1	---	K.FLSPGTICKK.A
1488.6485	1487.6412	1487.6180	15.6	63 -	74	1	---	K.RGEHCISGPCCR.N
1962.7609	1961.7536	1961.7190	17.6	90 -	106	0	144	K.GDDMNDYCTGISSDCPR.N
1978.7508	1977.7435	1977.7139	15.0	90 -	106	0	---	K.GDDMNDYCTGISSDCPR.N + Oxidation (M)

No match to: 837.4636, 855.4421, 857.4530, 878.4896, 972.4927, 1029.5150, 1051.4952, 1055.5298, 1067.9243, 1074.5970, 1108.5827, 1121.6016, 1146.5961, 1163.5883, 1165.6056, 1176.6072, 1179.6184, 1191.6215, 1207.6166, 1261.5141, 1318.5339, 1410.6084, 1417.6150, 1455.6294, 1467.6290, 1474.6361, 1500.6513, 1516.6512, 1546.7027, 1567.7723, 1608.7992, 1647.7029, 1724.7331, 1756.7135, 1761.7616, 1770.7319, 1781.7622, 1797.7334, 1812.7381, 1815.7513, 1823.7408, 1826.7562, 1829.7702, 1868.7659, 1872.7738, 1883.7702, 1886.7890, 1892.8123, 1895.7442, 1899.7901, 1902.7794, 1905.7432, 1910.7142, 1914.7672, 1918.7763, 1934.8110, 1945.7541, 1975.7763, 1992.7598, 2004.7669, 2017.7917, 2024.7746, 2032.8253, 2042.8378, 2045.8362, 2059.8188, 2073.8169, 2076.8302, 2085.8450, 2087.8275, 2089.8494, 2092.8140, 2094.8053, 2103.8398, 2116.8347, 2119.8331, 2128.9533, 2131.8331, 2133.8520, 2146.8641, 2149.8433, 2155.8348, 2159.8681, 2163.8504, 2166.8197, 2171.7913, 2175.8612, 2189.8881, 2227.0026, 2245.0130, 2259.0228, 2277.8956, 2321.8875, 2359.0533, 2887.3127

Search Parameters

```
Type of search         : MS/MS Ion Search
Enzyme                 : Trypsin
Fixed modifications    : Carbamidomethyl (C)
Variable modifications : Oxidation (M)
Mass values            : Monoisotopic
Protein Mass           : Unrestricted
Peptide Mass Tolerance : ± 100 ppm
Fragment Mass Tolerance: ± 0.7 Da
Max Missed Cleavages   : 1
Instrument type        : MALDI-TOF-TOF
Query1  (837.4636,1+)  : <no title>
Query2  (855.4421,1+)  : <no title>
Query3  (857.4530,1+)  : <no title>
Query4  (878.4896,1+)  : <no title>
Query5  (972.4927,1+)  : <no title>
Query6  (1029.5150,1+) : <no title>
Query7  (1051.4952,1+) : <no title>
Query8  (1055.5298,1+) : <no title>
Query9  (1067.9243,1+) : <no title>
Query10  (1074.5970,1+) : <no title>
Query11  (1108.5827,1+) : <no title>
Query12  (1121.6016,1+) : <no title>
Query13  (1146.5961,1+) : <no title>
Query14  (1150.5950,1+) : <no title>
Query15  (1163.5883,1+) : <no title>
Query16  (1165.6056,1+) : \\PROTEOMICS1\D\DATA\2105\210505_TLIEPOL_1_68252-68266\68252_68266_LPARDO\0_H5\1\1165.6056.LIFT\1SREF\PDATA\1\1R
Query17  (1176.6072,1+) : <no title>
Query18  (1179.6184,1+) : <no title>
Query19  (1191.6215,1+) : <no title>
Query20  (1207.6166,1+) : <no title>
Query21  (1261.5141,1+) : <no title>
Query22  (1318.5339,1+) : <no title>
Query23  (1410.6084,1+) : <no title>
Query24  (1417.6150,1+) : <no title>
Query25  (1455.6294,1+) : <no title>
Query26  (1467.6290,1+) : <no title>
Query27  (1474.6361,1+) : \\PROTEOMICS1\D\DATA\2105\210505_TLIEPOL_1_68252-68266\68252_68266_LPARDO\0_H5\1\1474.6361.LIFT\1SREF\PDATA\1\1R
Query28  (1488.6485,1+) : <no title>
Query29  (1500.6513,1+) : <no title>
Query30  (1516.6512,1+) : <no title>
Query31  (1546.7027,1+) : <no title>
Query32  (1567.7723,1+) : <no title>
Query33  (1608.7992,1+) : <no title>
Query34  (1647.7029,1+) : <no title>
Query35  (1724.7331,1+) : <no title>
Query36  (1756.7135,1+) : \\PROTEOMICS1\D\DATA\2105\210505_TLIEPOL_1_68252-68266\68252_68266_LPARDO\0_H5\1\1756.7135.LIFT\1SREF\PDATA\1\1R
Query37  (1761.7616,1+) : <no title>
Query38  (1770.7319,1+) : <no title>
Query39  (1781.7622,1+) : <no title>
Query40  (1797.7334,1+) : <no title>
Query41  (1812.7381,1+) : <no title>
Query42  (1815.7513,1+) : <no title>
Query43  (1823.7408,1+) : <no title>
```

```
Query44 (1826.7562,1+) : <no title>
Query45 (1829.7702,1+) : <no title>
Query46 (1868.7659,1+) : <no title>
Query47 (1872.7738,1+) : \\PROTEOMICS1\D\DATA\2105\210505_TLIEPOL_1_68252-68266\68252_68266_LPARDO\0_H5\1\1872.7738.LIFT\1SREF\PDATA\1\1R
Query48 (1883.7702,1+) : <no title>
Query49 (1886.7890,1+) : \\PROTEOMICS1\D\DATA\2105\210505_TLIEPOL_1_68252-68266\68252_68266_LPARDO\0_H5\1\1886.7890.LIFT\1SREF\PDATA\1\1R
Query50 (1892.8123,1+) : <no title>
Query51 (1895.7442,1+) : <no title>
Query52 (1899.7901,1+) : <no title>
Query53 (1902.7794,1+) : <no title>
Query54 (1905.7432,1+) : <no title>
Query55 (1910.7142,1+) : <no title>
Query56 (1914.7672,1+) : <no title>
Query57 (1918.7763,1+) : <no title>
Query58 (1934.8110,1+) : <no title>
Query59 (1945.7541,1+) : <no title>
Query60 (1962.7609,1+) : \\PROTEOMICS1\D\DATA\2105\210505_TLIEPOL_1_68252-68266\68252_68266_LPARDO\0_H5\1\1962.7609.LIFT\1SREF\PDATA\1\1R
Query61 (1975.7763,1+) : <no title>
Query62 (1978.7508,1+) : <no title>
Query63 (1992.7598,1+) : <no title>
Query64 (2004.7669,1+) : <no title>
Query65 (2017.7917,1+) : <no title>
Query66 (2024.7746,1+) : <no title>
Query67 (2032.8253,1+) : <no title>
Query68 (2042.8378,1+) : <no title>
Query69 (2045.8362,1+) : <no title>
Query70 (2059.8188,1+) : <no title>
Query71 (2073.8169,1+) : <no title>
Query72 (2076.8302,1+) : <no title>
Query73 (2085.8450,1+) : <no title>
Query74 (2087.8275,1+) : <no title>
Query75 (2089.8494,1+) : <no title>
Query76 (2092.8140,1+) : <no title>
Query77 (2094.8053,1+) : <no title>
Query78 (2103.8398,1+) : <no title>
Query79 (2116.8347,1+) : <no title>
Query80 (2119.8331,1+) : <no title>
Query81 (2128.9533,1+) : <no title>
Query82 (2131.8331,1+) : <no title>
Query83 (2133.8520,1+) : \\PROTEOMICS1\D\DATA\2105\210505_TLIEPOL_1_68252-68266\68252_68266_LPARDO\0_H5\1\2133.8520.LIFT\1SREF\PDATA\1\1R
Query84 (2146.8641,1+) : <no title>
Query85 (2149.8433,1+) : \\PROTEOMICS1\D\DATA\2105\210505_TLIEPOL_1_68252-68266\68252_68266_LPARDO\0_H5\1\2149.8433.LIFT\1SREF\PDATA\1\1R
Query86 (2155.8348,1+) : <no title>
Query87 (2159.8681,1+) : <no title>
Query88 (2163.8504,1+) : <no title>
Query89 (2166.8197,1+) : <no title>
Query90 (2171.7913,1+) : <no title>
Query91 (2175.8612,1+) : <no title>
Query92 (2189.8881,1+) : <no title>
Query93 (2227.0026,1+) : <no title>
Query94 (2245.0130,1+) : <no title>
Query95 (2259.0228,1+) : <no title>
Query96 (2277.8956,1+) : <no title>
Query97 (2321.8875,1+) : <no title>
Query98 (2359.0533,1+) : <no title>
Query99 (2887.3127,1+) : <no title>
```

Mascot: http://www.matrixscience.com/

MATRIX SCIENCE Mascot Search Results

Fr2-2

```
User            :
Email           :
Search title    : vipera_All_8IDplus4 file:\\Proteomics1\d\data\2105\210505_tliepol_1_68252-68266\68252_68266_lpardo\0_G6\1\1SRef\pdata\1\peaklist.xml
Database        : vipera  (1414 sequences; 347452 residues)
Timestamp       : 5 May 2021 at 09:33:21 GMT
Top Score       : 76 for DID7A_VIPBB, Disintegrin VB7A OS=Vipera berus berus OX=31156 PE=1 SV=1
```

Mascot Score Histogram

Protein score is -10*Log(P), where P is the probability that the observed match is a random event.
Protein scores greater than 44 are significant (p<0.05).

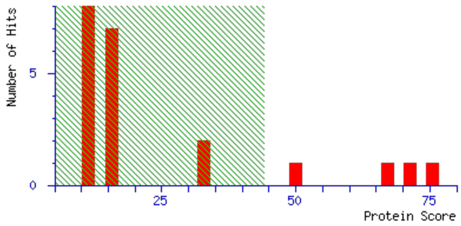

Concise Protein Summary Report

Format As | Concise Protein Summary | Help

Significance threshold p< 0.05 Max. number of hits 10

Re-Search All | Search Unmatched

1. DID7A_VIPBB **Mass:** 7574 **Score:** **76** **Expect:** 3.9e-005 **Matches:** 5
   Disintegrin VB7A OS=Vipera berus berus OX=31156 PE=1 SV=1
   VM2A6_VIPAA **Mass:** 7588 **Score:** **53** **Expect:** 0.0071 **Matches:** 4
   Disintegrin VA6 OS=Vipera ammodytes ammodytes OX=8705 PE=1 SV=1
   A0A6G5ZVR7_9SAUR **Mass:** 9593 **Score:** **50** **Expect:** 0.014 **Matches:** 4
   Disintegrin (Fragment) OS=Vipera anatolica senliki OX=2604287 PE=2 SV=1
   A0A1J0CZN0_VIPAA **Mass:** 12765 **Score:** **47** **Expect:** 0.026 **Matches:** 4
   Dis-2 OS=Vipera ammodytes ammodytes OX=8705 PE=2 SV=1
   SLB_MACLB **Mass:** 4871 **Score:** 16 **Expect:** 32 **Matches:** 1
   Snaclec lebecetin subunit beta (Fragment) OS=Macrovipera lebetina OX=8709 PE=1 SV=1
   VKT3_DABRR **Mass:** 9753 **Score:** 14 **Expect:** 51 **Matches:** 2
   Kunitz-type serine protease inhibitor 3 OS=Daboia russelii OX=8707 PE=2 SV=1
   H9BFA3_DABRR **Mass:** 10467 **Score:** 14 **Expect:** 55 **Matches:** 2
   Kunitz-type protease inhibitor OS=Daboia russelii OX=8707 PE=2 SV=1
   SLLC1_MACLB **Mass:** 16969 **Score:** 12 **Expect:** 89 **Matches:** 1
   Snaclec coagulation factor X-activating enzyme light chain 1 OS=Macrovipera lebetina OX=8709 GN=LC1 PE=1 SV=1
   A0A1I9KNR7_VIPAA **Mass:** 14615 **Score:** 12 **Expect:** 91 **Matches:** 1
   Disintegrin Dis-1 OS=Vipera ammodytes ammodytes OX=8705 PE=2 SV=1

---

2. DID4_MACLO **Mass:** 7673 **Score:** **71** **Expect:** 0.00011 **Matches:** 7
   Disintegrin VLO4 OS=Macrovipera lebetina obtusa OX=209528 PE=1 SV=1

---

3. DID1A_MACLB **Mass:** 12718 **Score:** **67** **Expect:** 0.00028 **Matches:** 8
   Disintegrin lebein-1-alpha OS=Macrovipera lebetina OX=8709 PE=1 SV=2
   NTF4_MACLB **Mass:** 4819 **Score:** 16 **Expect:** 35 **Matches:** 1
   Neurotrophin-4 (Fragment) OS=Macrovipera lebetina OX=8709 GN=NTF4 PE=3 SV=1

---

4. Q1JRG9_MACLN **Mass:** 7698 **Score:** **51** **Expect:** 0.012 **Matches:** 5
   VGD-containing dimeric disintegrin subunit ML-G1 (Fragment) OS=Macrovipera lebetina transmediterranea OX=384075 GN=ml-G1 PE=4 SV=1
   DID5B_MACLO **Mass:** 8235 **Score:** 31 **Expect:** 1.2 **Matches:** 3
   Disintegrin VLO5B OS=Macrovipera lebetina obtusa OX=209528 PE=1 SV=1
   Q7ZZQ1_DABSI **Mass:** 16246 **Score:** 12 **Expect:** 80 **Matches:** 1
   Phospholipase A(2) OS=Daboia siamensis OX=343250 PE=2 SV=1
   PA2A1_DABRR **Mass:** 16174 **Score:** 12 **Expect:** 80 **Matches:** 1
   Acidic phospholipase A2 Drk-a1 OS=Daboia russelii OX=8707 PE=1 SV=1
   PA2AA_DABSI **Mass:** 16240 **Score:** 12 **Expect:** 80 **Matches:** 1
   Acidic phospholipase A2 daboiatoxin A chain OS=Daboia siamensis OX=343250 PE=1 SV=1

---

5. VM25A_MACLO **Mass:** 7570 **Score:** 35 **Expect:** 0.47 **Matches:** 4
   Disintegrin VLO5A OS=Macrovipera lebetina obtusa OX=209528 PE=1 SV=1

---

6. DID1B_MACLB **Mass:** 7686 **Score:** 33 **Expect:** 0.76 **Matches:** 5
   Disintegrin lebein-1-beta OS=Macrovipera lebetina OX=8709 PE=1 SV=1

---

7. VM3VB_MACLB **Mass:** 71022 **Score:** 16 **Expect:** 37 **Matches:** 5
   Zinc metalloproteinase-disintegrin-like VLAIP-B OS=Macrovipera lebetina OX=8709 PE=1 SV=1

---

8. DIDB_CERVI **Mass:** 7584 **Score:** 16 **Expect:** 38 **Matches:** 7
   Disintegrin CV-11-beta (Fragment) OS=Cerastes vipera OX=8698 PE=2 SV=1
   DIDA_CERVI **Mass:** 12717 **Score:** 13 **Expect:** 65 **Matches:** 5
   Disintegrin CV-11-alpha OS=Cerastes vipera OX=8698 PE=2 SV=1

---

9. VKT_VIPRE **Mass:** 7707 **Score:** 15 **Expect:** 41 **Matches:** 2
   Kunitz-type serine protease inhibitor Vur-KIn OS=Vipera renardi OX=927686 PE=1 SV=1
   S4S374_VIPRE **Mass:** 10144 **Score:** 14 **Expect:** 55 **Matches:** 2
   Venom Kunitz trypsin inhibitor protein 2 OS=Vipera renardi OX=927686 PE=2 SV=1

---

10. A0A6G5ZUN8_9SAUR **Mass:** 61639 **Score:** 15 **Expect:** 45 **Matches:** 7
    Atriopeptidase (Fragment) OS=Vipera anatolica senliki OX=2604287 PE=2 SV=1

A0A6G5ZUW8_9SAUR **Mass:** 13665 **Score:** 12 **Expect:** 83 **Matches:** 1
Vascular endothelial growth factor 5 (Fragment) OS=Vipera anatolica senliki OX=2604287 PE=2 SV=1
Q6A389_VIPBB **Mass:** 16410 **Score:** 11 **Expect:** 1e+002 **Matches:** 1
Ammodytin L(2) variant OS=Vipera berus berus OX=31156 PE=2 SV=1
Q6A371_VIPAM **Mass:** 16461 **Score:** 11 **Expect:** 1e+002 **Matches:** 1
Ammodytin L(3) isoform OS=Vipera ammodytes montandoni OX=235554 PE=2 SV=1
Q6A394_VIPBB **Mass:** 16414 **Score:** 11 **Expect:** 1e+002 **Matches:** 1
Ammodytin L(2) isoform OS=Vipera berus berus OX=31156 PE=2 SV=1
Q6A388_VIPBB **Mass:** 16415 **Score:** 11 **Expect:** 1e+002 **Matches:** 1
Ammodytin L(2) variant OS=Vipera berus berus OX=31156 PE=2 SV=1

Search Parameters

Type of search : Peptide Mass Fingerprint
Enzyme : Trypsin
Fixed modifications : Carbamidomethyl (C)
Variable modifications : Oxidation (M)
Mass values : Monoisotopic
Protein Mass : Unrestricted
Peptide Mass Tolerance : ± 100 ppm
Peptide Charge State : 1+
Max Missed Cleavages : 1
Number of queries : 97
Selected for scoring : 10

Mascot: http://www.matrixscience.com/

MATRIX SCIENCE Mascot Search Results

```
User          :
Email         :
Search title  : vipera_all_Lift file:\\Proteomics1\d\data\2105\210505_tliepol_1_68252-68266\68252_68266_lpardo\0_G6\1\0_G6.xml
Database      : vipera  (1414 sequences; 347452 residues)
Timestamp     : 5 May 2021 at 09:41:18 GMT
Protein hits  : DID7A_VIPBB       Disintegrin VB7A OS=Vipera berus berus OX=31156 PE=1 SV=1
                DID1A_MACLB       Disintegrin lebein-1-alpha OS=Macrovipera lebetina OX=8709 PE=1 SV=2
                VM2A6_VIPAA       Disintegrin VA6 OS=Vipera ammodytes ammodytes OX=8705 PE=1 SV=1
                DID4_MACLO        Disintegrin VLO4 OS=Macrovipera lebetina obtusa OX=209528 PE=1 SV=1
                Q1JRG9_MACLN      VGD-containing dimeric disintegrin subunit ML-G1 (Fragment) OS=Macrovipera lebetina transmediterranea OX=384075 GN=ml-G1 PE=4 SV=1
                VM25A_MACLO       Disintegrin VLO5A OS=Macrovipera lebetina obtusa OX=209528 PE=1 SV=1
                A0A6G5ZVV3_9SAUR  Vascular endothelial growth factor 2 OS=Vipera anatolica senliki OX=2604287 PE=2 SV=1
                A0A1Q1NMP5_VIPBE  V(D)J recombination-activating protein 1 (Fragment) OS=Vipera berus OX=31155 GN=RAG1 PE=3 SV=1
```

Mascot Score Histogram

Ions score is -10*Log(P), where P is the probability that the observed match is a random event.
Individual ions scores > 11 indicate identity or extensive homology (p<0.05).
Protein scores are derived from ions scores as a non-probabilistic basis for ranking protein hits.

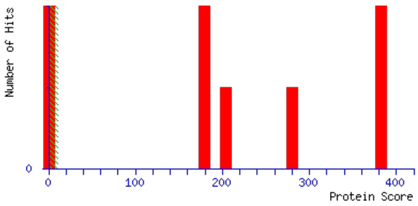

Peptide Summary Report

Format As | Peptide Summary | Help

Significance threshold p< 0.05 | Max. number of hits 10
Standard scoring ○ MudPIT scoring ◉ | Ions score or expect cut-off 0 | Show sub-sets 0
Show pop-ups ◉ Suppress pop-ups ○ | Sort unassigned Decreasing Score | Require bold red ☐

Overview Table

Click on column header to jump to entry in results list.
Move mouse over any indicator to highlight identical peptides.
Click on an indicator to see details of individual match.
Use check boxes to select sub-set of queries for new search.

Mouse over: -Query- | -Accession- | -Sequence-

Hit:	1	2	3	4	5	6	7	8
☑ 1029.5132 (1+)								
☑ 1074.5952 (1+)								
☑ 1106.5680 (1+)								
☑ 1108.5847 (1+)								
☑ 1119.5947 (1+)								
☑ 1121.6051 (1+)								
☑ 1146.5963 (1+)								
☑ 1148.5788 (1+)								
☑ 1150.5916 (1+)								
☑ 1162.5933 (1+)								
☑ 1165.6061 (1+)		●						●
☑ 1176.6084 (1+)								
☑ 1179.6202 (1+)								
☑ 1187.5818 (1+)								
☑ 1189.6030 (1+)								
☑ 1191.6211 (1+)								
☑ 1197.5771 (1+)								
☑ 1207.6162 (1+)								
☑ 1261.5168 (1+)								
☑ 1317.6060 (1+)								
☑ 1318.5355 (1+)	●	●	●	●	●		●	
☑ 1332.5510 (1+)								
☑ 1383.6258 (1+)								
☑ 1397.4491 (1+)								
☑ 1410.6107 (1+)								
☑ 1417.6152 (1+)								
☑ 1455.6313 (1+)								
☑ 1459.9186 (1+)								
☑ 1467.6321 (1+)								
☑ 1471.6241 (1+)								
☑ 1474.6378 (1+)	●	●	●	●	●			
☑ 1485.6406 (1+)								
☑ 1488.6529 (1+)								
☑ 1496.6199 (1+)								
☑ 1500.6537 (1+)								
☑ 1516.6503 (1+)								
☑ 1524.6495 (1+)								
☑ 1542.6655 (1+)								
☑ 1567.7730 (1+)								
☑ 1583.6915 (1+)								

☑ 1608.8002 (1+)								
☑ 1647.7150 (1+)								
☑ 1697.7476 (1+)								
☑ 1715.7585 (1+)								
☑ 1742.7690 (1+)								
☑ 1761.7738 (1+)	●			●		●		
☑ 1772.7726 (1+)								
☑ 1776.7684 (1+)								
☑ 1787.7767 (1+)								
☑ 1802.7917 (1+)								
☑ 1817.7702 (1+)								
☑ 1826.7647 (1+)								
☑ 1828.7826 (1+)								
☑ 1830.7847 (1+)								
☑ 1834.7863 (1+)								
☑ 1844.7780 (1+)								
☑ 1846.7894 (1+)								
☑ 1848.7918 (1+)								
☑ 1862.7775 (1+)								
☑ 1869.7501 (1+)								
☑ 1872.7776 (1+)								
☑ 1875.7943 (1+)								
☑ 1885.7714 (1+)								
☑ 1889.7890 (1+)								
☑ 1892.8182 (1+)				●				
☑ 1903.7892 (1+)								
☑ 1904.7346 (1+)								
☑ 1905.7619 (1+)								
☑ 1906.7841 (1+)								
☑ 1908.8129 (1+)								
☑ 1914.7625 (1+)								
☑ 1916.7465 (1+)								
☑ 1918.2815 (1+)								
☑ 1918.7903 (1+)								
☑ 1921.7303 (1+)								
☑ 1934.8231 (1+)								
☑ 1943.7515 (1+)								
☑ 1945.7522 (1+)								
☑ 1948.7478 (1+)								
☑ 1950.8218 (1+)								
☑ 1958.7646 (1+)								
☑ 1960.7335 (1+)								
☑ 1962.7632 (1+)	●	●	●					
☑ 1975.7723 (1+)								
☑ 1978.7513 (1+)	●	●	●					
☑ 1984.7457 (1+)								
☑ 1988.7698 (1+)								
☑ 1992.7606 (1+)								
☑ 2000.6976 (1+)								
☑ 2004.7706 (1+)								
☑ 2019.7737 (1+)								
☑ 2133.8517 (1+)					●	●		
☑ 2149.8364 (1+)								
☑ 2189.8910 (1+)								
☑ 2205.8830 (1+)								
☑ 2227.0072 (1+)								
☑ 2245.0161 (1+)								

Select All | Select None | Search Selected | ☐ **Error tolerant** | Archive Report

1. DID7A_VIPBB **Mass:** 7574 **Score:** 383 **Matches:** 5(5) **Sequences:** 4(4)
Disintegrin VB7A OS=Vipera berus berus OX=31156 PE=1 SV=1
☐ Check to include this hit in error tolerant search or archive report

Query	Observed	Mr(expt)	Mr(calc)	ppm	Miss	Score	Expect	Rank	Unique	Peptide
21	1318.5355	1317.5282	1317.5013	20.4	0	34	0.00039	1		R.GEHCVSGPCCR.N
31	1474.6378	1473.6305	1473.6024	19.1	1	60	9.4e-007	1		R.RGEHCVSGPCCR.N
46	1761.7738	1760.7666	1760.7393	15.5	0	80	9.5e-009	1		-.NSGNPCCDPVTCKPR.R
83	1962.7632	1961.7559	1961.7190	18.8	0	142	6.3e-015	1		R.GDDMNDYCTGISSDCPR.N
85	1978.7513	1977.7441	1977.7139	15.2	0	(118)	1.5e-012	1		R.GDDMNDYCTGISSDCPR.N + Oxidation (M)

2. DID1A_MACLB **Mass:** 12718 **Score:** 373 **Matches:** 5(5) **Sequences:** 4(4)
Disintegrin lebein-1-alpha OS=Macrovipera lebetina OX=8709 PE=1 SV=2
☐ Check to include this hit in error tolerant search or archive report

Query	Observed	Mr(expt)	Mr(calc)	ppm	Miss	Score	Expect	Rank	Unique	Peptide
11	1165.6061	1164.5988	1164.5710	23.9	0	71	8.6e-008	1	U	K.FLNAGTICNR.A
21	1318.5355	1317.5282	1317.5013	20.4	0	34	0.00039	1		R.GEHCVSGPCCR.N
31	1474.6378	1473.6305	1473.6024	19.1	1	60	9.4e-007	1		R.RGEHCVSGPCCR.N
83	1962.7632	1961.7559	1961.7190	18.8	0	142	6.3e-015	1		R.GDDMNDYCTGISSDCPR.N
85	1978.7513	1977.7441	1977.7139	15.2	0	(118)	1.5e-012	1		R.GDDMNDYCTGISSDCPR.N + Oxidation (M)

3. VM2A6_VIPAA **Mass:** 7588 **Score:** 284 **Matches:** 4(4) **Sequences:** 3(3)
Disintegrin VA6 OS=Vipera ammodytes ammodytes OX=8705 PE=1 SV=1
☐ Check to include this hit in error tolerant search or archive report

Query	Observed	Mr(expt)	Mr(calc)	ppm	Miss	Score	Expect	Rank	Unique	Peptide
21	1318.5355	1317.5282	1317.5013	20.4	0	34	0.00039	1		R.GEHCVSGPCCR.N
31	1474.6378	1473.6305	1473.6024	19.1	1	60	9.4e-007	1		R.RGEHCVSGPCCR.N
83	1962.7632	1961.7559	1961.7190	18.8	0	125	3.3e-013	2	U	R.GDDMNDYCTGVTSDCPR.N
85	1978.7513	1977.7440	1977.7139	15.2	0	(103)	4.5e-011	2	U	R.GDDMNDYCTGVTSDCPR.N + Oxidation (M)

4. DID4_MACLO **Mass:** 7673 **Score:** 213 **Matches:** 4(4) **Sequences:** 4(4)
Disintegrin VLO4 OS=Macrovipera lebetina obtusa OX=209528 PE=1 SV=1
☐ Check to include this hit in error tolerant search or archive report

Query	Observed	Mr(expt)	Mr(calc)	ppm	Miss	Score	Expect	Rank	Unique	Peptide
21	1318.5355	1317.5282	1317.5013	20.4	0	34	0.00039	1		R.GEHCVSGPCCR.N
31	1474.6378	1473.6305	1473.6024	19.1	1	60	9.4e-007	1		R.RGEHCVSGPCCR.N
46	1761.7738	1760.7666	1760.7393	15.5	0	80	9.5e-009	1		M.NSGNPCCDPVTCKPR.R
65	**1892.8182**	**1891.8109**	**1891.7798**	**16.5**	**0**	**77**	**1.9e-008**	**1**	**U**	**-.MNSGNPCCDPVTCKPR.R**

5. Q1JRG9_MACLN **Mass:** 7698 **Score:** 178 **Matches:** 3(3) **Sequences:** 3(3)
VGD-containing dimeric disintegrin subunit ML-G1 (Fragment) OS=Macrovipera lebetina transmediterranea OX=384075 GN=ml-G1 PE=4 SV=1
☐ Check to include this hit in error tolerant search or archive report

Query	Observed	Mr(expt)	Mr(calc)	ppm	Miss	Score	Expect	Rank	Unique	Peptide
21	1318.5355	1317.5282	1317.5013	20.4	0	34	0.00039	1		R.GEHCVSGPCCR.N
31	1474.6378	1473.6305	1473.6024	19.1	1	60	9.4e-007	1		R.RGEHCVSGPCCR.N
92	**2133.8517**	**2132.8444**	**2132.8085**	**16.8**	**0**	**110**	**1e-011**	**1**		**R.AVGDDMDDYCTGISSDCPR.N**

6. VM25A_MACLO **Mass:** 7570 **Score:** 177 **Matches:** 2(2) **Sequences:** 2(2)
Disintegrin VLO5A OS=Macrovipera lebetina obtusa OX=209528 PE=1 SV=1
☐ Check to include this hit in error tolerant search or archive report

Query	Observed	Mr(expt)	Mr(calc)	ppm	Miss	Score	Expect	Rank	Unique	Peptide
46	1761.7738	1760.7666	1760.7029	36.2	0	80	9.5e-009	1	U	-.NSGNPCCDPVTCQPR.R
92	2133.8517	2132.8444	2132.8085	16.8	0	110	1e-011	1		R.AVGDDMDDYCTGISSDCPR.N

7. A0A6G5ZVV3_9SAUR **Score:** 0 **Matches:** 1(0) **Sequences:** 1(0)
Vascular endothelial growth factor 2 OS=Vipera anatolica senliki OX=2604287 PE=2 SV=1
☐ Check to include this hit in error tolerant search or archive report

Query	Observed	Mr(expt)	Mr(calc)	ppm	Miss	Score	Expect	Rank	Unique	Peptide
21	1318.5355	1317.5282	1317.6459	-89.31	1	6	0.25	2	U	K.QLELNERTCR.C

Proteins matching the same set of peptides:
VEGFA_VIPAA **Mass:** 23357 **Score:** 0 **Matches:** 1(0) **Sequences:** 1(0)
Vascular endothelial growth factor A OS=Vipera ammodytes ammodytes OX=8705 PE=2 SV=1

8. A0A1Q1NMP5_VIPBE **Score:** 0 **Matches:** 1(0) **Sequences:** 1(0)
V(D)J recombination-activating protein 1 (Fragment) OS=Vipera berus OX=31155 GN=RAG1 PE=3 SV=1
☐ Check to include this hit in error tolerant search or archive report

Query	Observed	Mr(expt)	Mr(calc)	ppm	Miss	Score	Expect	Rank	Unique	Peptide
11	1165.6061	1164.5988	1164.6172	-15.83	1	6	0.23	2	U	K.KTMLNSQLSK.K + Oxidation (M)

Peptide matches not assigned to protein hits: (no details means no match)

	Query	Observed	Mr(expt)	Mr(calc)	ppm	Miss	Score	Expect	Rank	Unique	Peptide
☑	1	*1029.5132*	*1028.5059*								
☑	2	*1074.5952*	*1073.5879*								
☑	3	*1106.5680*	*1105.5607*								
☑	4	*1108.5847*	*1107.5774*								
☑	5	*1119.5947*	*1118.5874*								
☑	6	*1121.6051*	*1120.5978*								
☑	7	*1146.5963*	*1145.5890*								
☑	8	*1148.5788*	*1147.5716*								
☑	9	*1150.5916*	*1149.5843*								
☑	10	*1162.5933*	*1161.5860*								
☑	12	*1176.6084*	*1175.6011*								
☑	13	*1179.6202*	*1178.6129*								
☑	14	*1187.5818*	*1186.5746*								
☑	15	*1189.6030*	*1188.5957*								
☑	16	*1191.6211*	*1190.6139*								
☑	17	*1197.5771*	*1196.5699*								
☑	18	*1207.6162*	*1206.6089*								
☑	19	*1261.5168*	*1260.5095*								
☑	20	*1317.6060*	*1316.5987*								
☑	22	*1332.5510*	*1331.5437*								
☑	23	*1383.6258*	*1382.6185*								
☑	24	*1397.4491*	*1396.4419*								
☑	25	*1410.6107*	*1409.6034*								
☑	26	*1417.6152*	*1416.6080*								
☑	27	*1455.6313*	*1454.6241*								
☑	28	*1459.9186*	*1458.9113*								
☑	29	*1467.6321*	*1466.6248*								
☑	30	*1471.6241*	*1470.6168*								
☑	32	*1485.6406*	*1484.6333*								
☑	33	*1488.6529*	*1487.6456*								
☑	34	*1496.6199*	*1495.6126*								
☑	35	*1500.6537*	*1499.6464*								
☑	36	*1516.6503*	*1515.6430*								
☑	37	*1524.6495*	*1523.6422*								
☑	38	*1542.6655*	*1541.6582*								
☑	39	*1567.7730*	*1566.7657*								
☑	40	*1583.6915*	*1582.6842*								
☑	41	*1608.8002*	*1607.7930*								
☑	42	*1647.7150*	*1646.7077*								
☑	43	*1697.7476*	*1696.7403*								
☑	44	*1715.7585*	*1714.7512*								
☑	45	*1742.7690*	*1741.7617*								
☑	47	*1772.7726*	*1771.7653*								
☑	48	*1776.7684*	*1775.7611*								
☑	49	*1787.7767*	*1786.7694*								
☑	50	*1802.7917*	*1801.7844*								
☑	51	*1817.7702*	*1816.7629*								
☑	52	*1826.7647*	*1825.7574*								
☑	53	*1828.7826*	*1827.7753*								

☑	54	*1830.7847*	*1829.7775*
☑	55	*1834.7863*	*1833.7790*
☑	56	*1844.7780*	*1843.7707*
☑	57	*1846.7894*	*1845.7821*
☑	58	*1848.7918*	*1847.7845*
☑	59	*1862.7775*	*1861.7702*
☑	60	*1869.7501*	*1868.7428*
☑	61	*1872.7776*	*1871.7703*
☑	62	*1875.7943*	*1874.7870*
☑	63	*1885.7714*	*1884.7641*
☑	64	*1889.7890*	*1888.7817*
☑	66	*1903.7892*	*1902.7820*
☑	67	*1904.7346*	*1903.7273*
☑	68	*1905.7619*	*1904.7546*
☑	69	*1906.7841*	*1905.7768*
☑	70	*1908.8129*	*1907.8056*
☑	71	*1914.7625*	*1913.7552*
☑	72	*1916.7465*	*1915.7393*
☑	73	*1918.2815*	*1917.2742*
☑	74	*1918.7903*	*1917.7830*
☑	75	*1921.7303*	*1920.7231*
☑	76	*1934.8231*	*1933.8158*
☑	77	*1943.7515*	*1942.7443*
☑	78	*1945.7522*	*1944.7449*
☑	79	*1948.7478*	*1947.7405*
☑	80	*1950.8218*	*1949.8145*
☑	81	*1958.7646*	*1957.7573*
☑	82	*1960.7335*	*1959.7262*
☑	84	*1975.7723*	*1974.7650*
☑	86	*1984.7457*	*1983.7384*
☑	87	*1988.7698*	*1987.7625*
☑	88	*1992.7606*	*1991.7533*
☑	89	*2000.6976*	*1999.6903*
☑	90	*2004.7706*	*2003.7633*
☑	91	*2019.7737*	*2018.7664*
☑	93	*2149.8364*	*2148.8291*
☑	94	*2189.8910*	*2188.8837*
☑	95	*2205.8830*	*2204.8758*
☑	96	*2227.0072*	*2225.9999*
☑	97	*2245.0161*	*2244.0088*

Search Parameters

```
Type of search         : MS/MS Ion Search
Enzyme                 : Trypsin
Fixed modifications    : Carbamidomethyl (C)
Variable modifications : Oxidation (M)
Mass values            : Monoisotopic
Protein Mass           : Unrestricted
Peptide Mass Tolerance : ± 100 ppm
Fragment Mass Tolerance: ± 0.7 Da
Max Missed Cleavages   : 1
Instrument type        : MALDI-TOF-TOF
Number of queries      : 97
```

Mascot: http://www.matrixscience.com/

Mascot Search Results

```
User          :
Email         :
Search title  : vipera_all_Lift file:\\Proteomics1\d\data\2105\210505_tliepol_1_68252-68266\68252_68266_1pardo\0_G6\1\0_G6.xml
Database      : vipera  (1414 sequences; 347452 residues)
Timestamp     : 5 May 2021 at 09:41:18 GMT
Warning       : A Peptide summary report will usually give a much clearer picture of MS/MS search results.
Top Score     : 356 for DID1A_MACLB, Disintegrin lebein-1-alpha OS=Macrovipera lebetina OX=8709 PE=1 SV=2
```

Mascot Score Histogram

Protein score is -10*Log(P), where P is the probability that the observed match is a random event.
Protein scores greater than 44 are significant (p<0.05).
Protein scores are derived from ions scores as a non-probabilistic basis for ranking protein hits.

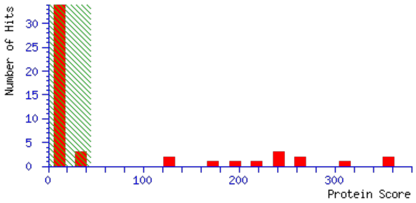

Protein Summary Report

Format As | Protein Summary (deprecated) | Help

Significance threshold p< 0.05 Max. number of hits 20

Overview Table

Click on column header to jump to entry in results list.
Move mouse over any indicator to highlight identical peptides.
Click on an indicator to see details of individual match.
Use check boxes to select sub-set of queries for new search.

Mouse over: -Query-
-Accession- -Sequence-

Hit:	1	2	3	4	5	6	7	8	9	10	11	12	13	14	15	16	17	18	19	20
☑ 1029.5132 (1+)																				
☑ 1074.5952 (1+)																				
☑ 1106.5680 (1+)																				
☑ 1108.5847 (1+)																				
☑ 1119.5947 (1+)																				
☑ 1121.6051 (1+)																				
☑ 1146.5963 (1+)																				
☑ 1148.5788 (1+)																				
☑ 1150.5916 (1+)																				
☑ 1162.5933 (1+)																				
☑ 1165.6061 (1+)	●																			
☑ 1176.6084 (1+)																				
☑ 1179.6202 (1+)																				
☑ 1187.5818 (1+)																				
☑ 1189.6030 (1+)																				
☑ 1191.6211 (1+)																				
☑ 1197.5771 (1+)																				
☑ 1207.6162 (1+)																				
☑ 1261.5168 (1+)																				
☑ 1317.6060 (1+)																				
☑ 1318.5355 (1+)	●	●	●	●	●		●	●					●	●	●	●				
☑ 1332.5510 (1+)																				
☑ 1383.6258 (1+)																				
☑ 1397.4491 (1+)																				
☑ 1410.6107 (1+)																				
☑ 1417.6152 (1+)																				
☑ 1455.6313 (1+)																				
☑ 1459.9186 (1+)																				
☑ 1467.6321 (1+)																				
☑ 1471.6241 (1+)																				
☑ 1474.6378 (1+)	●	●	●	●	●		●	●					●							
☑ 1485.6406 (1+)																				
☑ 1488.6529 (1+)																				
☑ 1496.6199 (1+)																				
☑ 1500.6537 (1+)																				
☑ 1516.6503 (1+)																				
☑ 1524.6495 (1+)																				
☑ 1542.6655 (1+)																				
☑ 1567.7730 (1+)																				
☑ 1583.6915 (1+)																				
☑ 1608.8002 (1+)																				
☑ 1647.7150 (1+)																				
☑ 1697.7476 (1+)																				
☑ 1715.7585 (1+)																				
☑ 1742.7690 (1+)																				
☑ 1761.7738 (1+)		●	●			●			●											
☑ 1772.7726 (1+)																				
☑ 1776.7684 (1+)																				
☑ 1787.7767 (1+)																				

☑ 1802.7917 (1+)																				
☑ 1817.7702 (1+)																				
☑ 1826.7647 (1+)																				
☑ 1828.7826 (1+)																				
☑ 1830.7847 (1+)																				
☑ 1834.7863 (1+)																				
☑ 1844.7780 (1+)																				
☑ 1846.7894 (1+)																				
☑ 1848.7918 (1+)																				
☑ 1862.7775 (1+)																				
☑ 1869.7501 (1+)																				
☑ 1872.7776 (1+)																				
☑ 1875.7943 (1+)																				
☑ 1885.7714 (1+)																				
☑ 1889.7890 (1+)																				
☑ 1892.8182 (1+)			●																	
☑ 1903.7892 (1+)																				
☑ 1904.7346 (1+)																				
☑ 1905.7619 (1+)																				
☑ 1906.7841 (1+)																				
☑ 1908.8129 (1+)																				
☑ 1914.7625 (1+)																				
☑ 1916.7465 (1+)																				
☑ 1918.2815 (1+)																				
☑ 1918.7903 (1+)																				
☑ 1921.7303 (1+)																				
☑ 1934.8231 (1+)																				
☑ 1943.7515 (1+)																				
☑ 1945.7522 (1+)																				
☑ 1948.7478 (1+)																				
☑ 1950.8218 (1+)																				
☑ 1958.7646 (1+)																				
☑ 1960.7335 (1+)																				
☑ 1962.7632 (1+)	●	●		●	●	●	●			●	●									
☑ 1975.7723 (1+)																				
☑ 1978.7513 (1+)	●	●		●	●	●	●			●	●									
☑ 1984.7457 (1+)																				
☑ 1988.7698 (1+)																				
☑ 1992.7606 (1+)																				
☑ 2000.6976 (1+)																				
☑ 2004.7706 (1+)																				
☑ 2019.7737 (1+)																				
☑ 2133.8517 (1+)								●	●			●								
☑ 2149.8364 (1+)																				
☑ 2189.8910 (1+)																				
☑ 2205.8830 (1+)																				
☑ 2227.0072 (1+)																				
☑ 2245.0161 (1+)																				

Select All | Select None | Search Selected

Index

	Accession	Mass	Score	Description
1.	DID1A_MACLB	12718	356	Disintegrin lebein-1-alpha OS=Macrovipera lebetina OX=8709 PE=1 SV=2
2.	DID7A_VIPBB	7574	351	Disintegrin VB7A OS=Vipera berus berus OX=31156 PE=1 SV=1
3.	DID4_MACLO	7673	305	Disintegrin VLO4 OS=Macrovipera lebetina obtusa OX=209528 PE=1 SV=1
4.	A0A6G5ZVR7_9SAUR	9593	257	Disintegrin (Fragment) OS=Vipera anatolica senliki OX=2604287 PE=2 SV=1
5.	A0A1J0CZN0_VIPAA	12765	255	Dis-2 OS=Vipera ammodytes ammodytes OX=8705 PE=2 SV=1
6.	DID1B_MACLB	7686	247	Disintegrin lebein-1-beta OS=Macrovipera lebetina OX=8709 PE=1 SV=1
7.	VM2A6_VIPAA	7588	243	Disintegrin VA6 OS=Vipera ammodytes ammodytes OX=8705 PE=1 SV=1
8.	Q1JRG9_MACLN	7698	236	VGD-containing dimeric disintegrin subunit ML-G1 (Fragment) OS=Macrovipera lebetina transmediterranea OX=384075 GN=ml-G1 PE=4 SV=1
9.	VM25A_MACLO	7570	214	Disintegrin VLO5A OS=Macrovipera lebetina obtusa OX=209528 PE=1 SV=1
10.	DIDB_CERVI	7584	188	Disintegrin CV-11-beta (Fragment) OS=Cerastes vipera OX=8698 PE=2 SV=1
11.	DIDA_CERVI	12717	169	Disintegrin CV-11-alpha OS=Cerastes vipera OX=8698 PE=2 SV=1
12.	VM2L2_MACLB	54586	120	Zinc metalloproteinase/disintegrin OS=Macrovipera lebetina OX=8709 PE=1 SV=1
13.	DID5B_MACLO	8235	116	Disintegrin VLO5B OS=Macrovipera lebetina obtusa OX=209528 PE=1 SV=1
14.	A0A1I9KNR4_VIPAA	14601	43	Disintegrin Dis-1 OS=Vipera ammodytes ammodytes OX=8705 PE=2 SV=1
15.	DID2A_MACLB	14638	43	Disintegrin lebein-2-alpha OS=Macrovipera lebetina OX=8709 PE=1 SV=1
16.	A0A1I9KNR7_VIPAA	14615	38	Disintegrin Dis-1 OS=Vipera ammodytes ammodytes OX=8705 PE=2 SV=1
17.	TXVE2_MACLB	13022	19	Snake venom vascular endothelial growth factor toxin ICPP OS=Macrovipera lebetina OX=8709 PE=1 SV=2
18.	K4GS84_DABRR	37760	18	Solute carrier family 8 member 1 (Fragment) OS=Daboia russelii OX=8707 GN=SLC8A1 PE=4 SV=1
19.	Q8UVG8_DABRR	52931	18	Vimentin OS=Daboia russelii OX=8707 PE=2 SV=1
20.	A0A6G5ZW48_9SAUR	86121	18	Atriopeptidase OS=Vipera anatolica senliki OX=2604287 PE=2 SV=1

Results List

1. DID1A_MACLB **Mass:** 12718 **Score:** 356 **Expect:** 3.6e-033 **Matches:** 8
Disintegrin lebein-1-alpha OS=Macrovipera lebetina OX=8709 PE=1 SV=2

Observed	Mr(expt)	Mr(calc)	ppm	Start	End	Miss	Ions	Peptide
1165.6061	1164.5988	1164.5710	23.9	78 -	87	0	71	K.FLNAGTICNR.A
1318.5355	1317.5282	1317.5013	20.4	64 -	74	0	34	R.GEHCVSGPCCR.N
1474.6378	1473.6305	1473.6024	19.1	63 -	74	1	60	R.RGEHCVSGPCCR.N
1567.7730	1566.7657	1566.7395	16.7	75 -	87	1	---	R.NCKFLNAGTICNR.A
1962.7632	1961.7559	1961.7190	18.8	90 -	106	0	142	R.GDDMNDYCTGISSDCPR.N
1978.7513	1977.7441	1977.7139	15.2	90 -	106	0	(118)	R.GDDMNDYCTGISSDCPR.N + Oxidation (M)
2189.8910	2188.8837	2188.8572	12.1	88 -	106	1	---	R.ARGDDMNDYCTGISSDCPR.N
2205.8830	2204.8758	2204.8521	10.7	88 -	106	1	---	R.ARGDDMNDYCTGISSDCPR.N + Oxidation (M)

No match to: 1029.5132, 1074.5952, 1106.5680, 1108.5847, 1119.5947, 1121.6051, 1146.5963, 1148.5788, 1150.5916, 1162.5933, 1176.6084, 1179.6202, 1187.5818, 1189.6030, 1191.6211, 1197.5771, 1207.6162, 1261.5168, 1317.6060, 1332.5510, 1383.6258, 1397.4491, 1410.6107, 1417.6152, 1455.6313, 1459.9186, 1467.6321, 1471.6241, 1485.6406, 1488.6529, 1496.6199, 1500.6537, 1516.6503, 1524.6495, 1542.6655, 1583.6915, 1608.8002, 1647.7150, 1697.7476, 1715.7585, 1742.7690, 1761.7738, 1772.7726, 1776.7684, 1787.7767, 1802.7917, 1817.7702, 1826.7647, 1828.7826, 1830.7847, 1834.7863, 1844.7780, 1846.7894, 1848.7918, 1862.7775, 1869.7501, 1872.7776, 1875.7943, 1885.7714, 1889.7890, 1892.8182, 1903.7892, 1904.7346, 1905.7619, 1906.7841, 1908.8129, 1914.7625, 1916.7465, 1918.2815, 1918.7903, 1921.7303, 1934.8231, 1943.7515, 1945.7522, 1948.7478, 1950.8218, 1958.7646, 1960.7335, 1975.7723, 1984.7457, 1988.7698, 1992.7606, 2000.6976, 2004.7706, 2019.7737, 2133.8517, 2149.8364, 2227.0072, 2245.0161

2. DID7A_VIPBB **Mass:** 7574 **Score:** 351 **Expect:** 1.1e-032 **Matches:** 5
Disintegrin VB7A OS=Vipera berus berus OX=31156 PE=1 SV=1

Observed	Mr(expt)	Mr(calc)	ppm	Start	End	Miss	Ions	Peptide
1318.5355	1317.5282	1317.5013	20.4	17 -	27	0	34	R.GEHCVSGPCCR.N

1474.6378	1473.6305	1473.6024	19.1	16 -	27	1	60	R.RGEHCVSGPCCR.N
1761.7738	1760.7666	1760.7393	15.5	1 -	15	0	80	-.NSGNPCCDPVTCKPR.R
1962.7632	1961.7559	1961.7190	18.8	43 -	59	0	142	R.GDDMNDYCTGISSDCPR.N
1978.7513	1977.7441	1977.7139	15.2	43 -	59	0	(118)	R.GDDMNDYCTGISSDCPR.N + Oxidation (M)

No match to: 1029.5132, 1074.5952, 1106.5680, 1108.5847, 1119.5947, 1121.6051, 1146.5963, 1148.5788, 1150.5916, 1162.5933, 1165.6061, 1176.6084, 1179.6202, 1187.5818, 1189.6030, 1191.6211, 1197.5771, 1207.6162, 1261.5168, 1317.6060, 1332.5510, 1383.6258, 1397.4491, 1410.6107, 1417.6152, 1455.6313, 1459.9186, 1467.6321, 1471.6241, 1485.6406, 1488.6529, 1496.6199, 1500.6537, 1516.6503, 1524.6495, 1542.6655, 1567.7730, 1583.6915, 1608.8002, 1647.7150, 1697.7476, 1715.7585, 1742.7690, 1772.7726, 1776.7684, 1787.7767, 1802.7917, 1817.7702, 1826.7647, 1828.7826, 1830.7847, 1834.7863, 1844.7780, 1846.7894, 1848.7918, 1862.7775, 1869.7501, 1872.7776, 1875.7943, 1885.7714, 1889.7890, 1892.8182, 1903.7892, 1904.7346, 1905.7619, 1906.7841, 1908.8129, 1914.7625, 1916.7465, 1918.2815, 1918.7903, 1921.7303, 1934.8231, 1943.7515, 1945.7522, 1948.7478, 1950.8218, 1958.7646, 1960.7335, 1975.7723, 1984.7457, 1988.7698, 1992.7606, 2000.6976, 2004.7706, 2019.7737, 2133.8517, 2149.8364, 2189.8910, 2205.8830, 2227.0072, 2245.0161

3. DID4_MACLO **Mass:** 7673 **Score:** 305 **Expect:** 4.5e-028 **Matches:** 7

Disintegrin VLO4 OS=Macrovipera lebetina obtusa OX=209528 PE=1 SV=1

Observed	Mr(expt)	Mr(calc)	ppm	Start	End	Miss	Ions	Peptide
1179.6202	1178.6129	1178.6230	-8.53	32 -	41	1	---	K.FLNAGTICKR.A
1318.5355	1317.5282	1317.5013	20.4	18 -	28	0	34	R.GEHCVSGPCCR.N
1474.6378	1473.6305	1473.6024	19.1	17 -	28	1	60	R.RGEHCVSGPCCR.N
1761.7738	1760.7666	1760.7393	15.5	2 -	16	0	80	M.NSGNPCCDPVTCKPR.R
1892.8182	1891.8109	1891.7798	16.5	1 -	16	0	77	-.MNSGNPCCDPVTCKPR.R
1908.8129	1907.8056	1907.7747	16.2	1 -	16	0	---	-.MNSGNPCCDPVTCKPR.R + Oxidation (M)
1988.7698	1987.7625	1987.7347	14.0	44 -	60	0	---	R.GDDMNDYCTGISPDCPR.N + Oxidation (M)

No match to: 1029.5132, 1074.5952, 1106.5680, 1108.5847, 1119.5947, 1121.6051, 1146.5963, 1148.5788, 1150.5916, 1162.5933, 1165.6061, 1176.6084, 1187.5818, 1189.6030, 1191.6211, 1197.5771, 1207.6162, 1261.5168, 1317.6060, 1332.5510, 1383.6258, 1397.4491, 1410.6107, 1417.6152, 1455.6313, 1459.9186, 1467.6321, 1471.6241, 1485.6406, 1488.6529, 1496.6199, 1500.6537, 1516.6503, 1524.6495, 1542.6655, 1567.7730, 1583.6915, 1608.8002, 1647.7150, 1697.7476, 1715.7585, 1742.7690, 1772.7726, 1776.7684, 1787.7767, 1802.7917, 1817.7702, 1826.7647, 1828.7826, 1830.7847, 1834.7863, 1844.7780, 1846.7894, 1848.7918, 1862.7775, 1869.7501, 1872.7776, 1875.7943, 1885.7714, 1889.7890, 1903.7892, 1904.7346, 1905.7619, 1906.7841, 1914.7625, 1916.7465, 1918.2815, 1918.7903, 1921.7303, 1934.8231, 1943.7515, 1945.7522, 1948.7478, 1950.8218, 1958.7646, 1960.7335, 1962.7632, 1975.7723, 1978.7513, 1984.7457, 1992.7606, 2000.6976, 2004.7706, 2019.7737, 2133.8517, 2149.8364, 2189.8910, 2205.8830, 2227.0072, 2245.0161

4. A0A6G5ZVR7_9SAUR **Mass:** 9593 **Score:** 257 **Expect:** 2.8e-023 **Matches:** 4

Disintegrin (Fragment) OS=Vipera anatolica senliki OX=2604287 PE=2 SV=1

Observed	Mr(expt)	Mr(calc)	ppm	Start	End	Miss	Ions	Peptide
1318.5355	1317.5282	1317.5013	20.4	35 -	45	0	34	R.GEHCVSGPCCR.N
1474.6378	1473.6305	1473.6024	19.1	34 -	45	1	60	R.RGEHCVSGPCCR.N
1962.7632	1961.7559	1961.7190	18.8	61 -	77	0	142	R.GDDMNDYCTGISSDCPR.N
1978.7513	1977.7441	1977.7139	15.2	61 -	77	0	(118)	R.GDDMNDYCTGISSDCPR.N + Oxidation (M)

No match to: 1029.5132, 1074.5952, 1106.5680, 1108.5847, 1119.5947, 1121.6051, 1146.5963, 1148.5788, 1150.5916, 1162.5933, 1165.6061, 1176.6084, 1179.6202, 1187.5818, 1189.6030, 1191.6211, 1197.5771, 1207.6162, 1261.5168, 1317.6060, 1332.5510, 1383.6258, 1397.4491, 1410.6107, 1417.6152, 1455.6313, 1459.9186, 1467.6321, 1471.6241, 1485.6406, 1488.6529, 1496.6199, 1500.6537, 1516.6503, 1524.6495, 1542.6655, 1567.7730, 1583.6915, 1608.8002, 1647.7150, 1697.7476, 1715.7585, 1742.7690, 1761.7738, 1772.7726, 1776.7684, 1787.7767, 1802.7917, 1817.7702, 1826.7647, 1828.7826, 1830.7847, 1834.7863, 1844.7780, 1846.7894, 1848.7918, 1862.7775, 1869.7501, 1872.7776, 1875.7943, 1885.7714, 1889.7890, 1892.8182, 1903.7892, 1904.7346, 1905.7619, 1906.7841, 1908.8129, 1914.7625, 1916.7465, 1918.2815, 1918.7903, 1921.7303, 1934.8231, 1943.7515, 1945.7522, 1948.7478, 1950.8218, 1958.7646, 1960.7335, 1975.7723, 1984.7457, 1988.7698, 1992.7606, 2000.6976, 2004.7706, 2019.7737, 2133.8517, 2149.8364, 2189.8910, 2205.8830, 2227.0072, 2245.0161

5. A0A1J0CZN0_VIPAA **Mass:** 12765 **Score:** 255 **Expect:** 4.5e-023 **Matches:** 4

Dis-2 OS=Vipera ammodytes ammodytes OX=8705 PE=2 SV=1

Observed	Mr(expt)	Mr(calc)	ppm	Start	End	Miss	Ions	Peptide
1318.5355	1317.5282	1317.5013	20.4	64 -	74	0	34	R.GEHCVSGPCCR.N
1474.6378	1473.6305	1473.6024	19.1	63 -	74	1	60	R.RGEHCVSGPCCR.N
1962.7632	1961.7559	1961.7190	18.8	90 -	106	0	142	R.GDDMNDYCTGISSDCPR.N
1978.7513	1977.7441	1977.7139	15.2	90 -	106	0	(118)	R.GDDMNDYCTGISSDCPR.N + Oxidation (M)

No match to: 1029.5132, 1074.5952, 1106.5680, 1108.5847, 1119.5947, 1121.6051, 1146.5963, 1148.5788, 1150.5916, 1162.5933, 1165.6061, 1176.6084, 1179.6202, 1187.5818, 1189.6030, 1191.6211, 1197.5771, 1207.6162, 1261.5168, 1317.6060, 1332.5510, 1383.6258, 1397.4491, 1410.6107, 1417.6152, 1455.6313, 1459.9186, 1467.6321, 1471.6241, 1485.6406, 1488.6529, 1496.6199, 1500.6537, 1516.6503, 1524.6495, 1542.6655, 1567.7730, 1583.6915, 1608.8002, 1647.7150, 1697.7476, 1715.7585, 1742.7690, 1761.7738, 1772.7726, 1776.7684, 1787.7767, 1802.7917, 1817.7702, 1826.7647, 1828.7826, 1830.7847, 1834.7863, 1844.7780, 1846.7894, 1848.7918, 1862.7775, 1869.7501, 1872.7776, 1875.7943, 1885.7714, 1889.7890, 1892.8182, 1903.7892, 1904.7346, 1905.7619, 1906.7841, 1908.8129, 1914.7625, 1916.7465, 1918.2815, 1918.7903, 1921.7303, 1934.8231, 1943.7515, 1945.7522, 1948.7478, 1950.8218, 1958.7646, 1960.7335, 1975.7723, 1984.7457, 1988.7698, 1992.7606, 2000.6976, 2004.7706, 2019.7737, 2133.8517, 2149.8364, 2189.8910, 2205.8830, 2227.0072, 2245.0161

6. DID1B_MACLB **Mass:** 7686 **Score:** 247 **Expect:** 2.8e-022 **Matches:** 5

Disintegrin lebein-1-beta OS=Macrovipera lebetina OX=8709 PE=1 SV=1

Observed	Mr(expt)	Mr(calc)	ppm	Start	End	Miss	Ions	Peptide
1761.7738	1760.7666	1760.7393	15.5	1 -	15	0	80	-.NSGNPCCDPVTCKPR.R
1962.7632	1961.7559	1961.7190	18.8	43 -	59	0	142	R.GDDMNDYCTGISSDCPR.N
1978.7513	1977.7441	1977.7139	15.2	43 -	59	0	(118)	R.GDDMNDYCTGISSDCPR.N + Oxidation (M)
2189.8910	2188.8837	2188.8572	12.1	41 -	59	1	---	R.ARGDDMNDYCTGISSDCPR.N
2205.8830	2204.8758	2204.8521	10.7	41 -	59	1	---	R.ARGDDMNDYCTGISSDCPR.N + Oxidation (M)

No match to: 1029.5132, 1074.5952, 1106.5680, 1108.5847, 1119.5947, 1121.6051, 1146.5963, 1148.5788, 1150.5916, 1162.5933, 1165.6061, 1176.6084, 1179.6202, 1187.5818, 1189.6030, 1191.6211, 1197.5771, 1207.6162, 1261.5168, 1317.6060, 1318.5355, 1332.5510, 1383.6258, 1397.4491, 1410.6107, 1417.6152, 1455.6313, 1459.9186, 1467.6321, 1471.6241, 1474.6378, 1485.6406, 1488.6529, 1496.6199, 1500.6537, 1516.6503, 1524.6495, 1542.6655, 1567.7730, 1583.6915, 1608.8002, 1647.7150, 1697.7476, 1715.7585, 1742.7690, 1772.7726, 1776.7684, 1787.7767, 1802.7917, 1817.7702, 1826.7647, 1828.7826, 1830.7847, 1834.7863, 1844.7780, 1846.7894, 1848.7918, 1862.7775, 1869.7501, 1872.7776, 1875.7943, 1885.7714, 1889.7890, 1892.8182, 1903.7892, 1904.7346, 1905.7619, 1906.7841, 1908.8129, 1914.7625, 1916.7465, 1918.2815, 1918.7903, 1921.7303, 1934.8231, 1943.7515, 1945.7522, 1948.7478, 1950.8218, 1958.7646, 1960.7335, 1975.7723, 1984.7457, 1988.7698, 1992.7606, 2000.6976, 2004.7706, 2019.7737, 2133.8517, 2149.8364, 2227.0072, 2245.0161

7. VM2A6_VIPAA **Mass:** 7588 **Score:** 243 **Expect:** 7.1e-022 **Matches:** 4

Disintegrin VA6 OS=Vipera ammodytes ammodytes OX=8705 PE=1 SV=1

Observed	Mr(expt)	Mr(calc)	ppm	Start	End	Miss	Ions	Peptide
1318.5355	1317.5282	1317.5013	20.4	17 -	27	0	34	R.GEHCVSGPCCR.N
1474.6378	1473.6305	1473.6024	19.1	16 -	27	1	60	R.RGEHCVSGPCCR.N
1962.7632	1961.7559	1961.7190	18.8	43 -	59	0	125	R.GDDMNDYCTGVTSDCPR.N
1978.7513	1977.7440	1977.7139	15.2	43 -	59	0	(103)	R.GDDMNDYCTGVTSDCPR.N + Oxidation (M)

No match to: 1029.5132, 1074.5952, 1106.5680, 1108.5847, 1119.5947, 1121.6051, 1146.5963, 1148.5788, 1150.5916, 1162.5933, 1165.6061, 1176.6084, 1179.6202, 1187.5818, 1189.6030, 1191.6211, 1197.5771, 1207.6162, 1261.5168, 1317.6060, 1332.5510, 1383.6258, 1397.4491, 1410.6107, 1417.6152, 1455.6313, 1459.9186, 1467.6321, 1471.6241, 1485.6406, 1488.6529, 1496.6199, 1500.6537, 1516.6503, 1524.6495, 1542.6655, 1567.7730, 1583.6915, 1608.8002, 1647.7150, 1697.7476, 1715.7585, 1742.7690, 1761.7738, 1772.7726, 1776.7684, 1787.7767, 1802.7917, 1817.7702, 1826.7647, 1828.7826, 1830.7847, 1834.7863, 1844.7780, 1846.7894, 1848.7918, 1862.7775, 1869.7501, 1872.7776, 1875.7943, 1885.7714, 1889.7890, 1892.8182, 1903.7892, 1904.7346, 1905.7619, 1906.7841, 1908.8129, 1914.7625, 1916.7465, 1918.2815, 1918.7903, 1921.7303, 1934.8231, 1943.7515, 1945.7522, 1948.7478, 1950.8218, 1958.7646, 1960.7335, 1975.7723, 1984.7457, 1988.7698, 1992.7606, 2000.6976, 2004.7706, 2019.7737, 2133.8517, 2149.8364, 2189.8910, 2205.8830, 2227.0072, 2245.0161

8. Q1JRG9_MACLN **Mass:** 7698 **Score:** 236 **Expect:** 3.6e-021 **Matches:** 5

VGD-containing dimeric disintegrin subunit ML-G1 (Fragment) OS=Macrovipera lebetina transmediterranea OX=384075 GN=ml-G1 PE=4 SV=1

Observed	Mr(expt)	Mr(calc)	ppm	Start	End	Miss	Ions	Peptide
1318.5355	1317.5282	1317.5013	20.4	18 -	28	0	34	R.GEHCVSGPCCR.N
1474.6378	1473.6305	1473.6024	19.1	17 -	28	1	60	R.RGEHCVSGPCCR.N
1945.7522	1944.7449	1944.8717	-65.19	2 -	17	1	---	M.NSANPCCDPITCKPRR.G
2133.8517	2132.8444	2132.8085	16.8	42 -	60	0	110	R.AVGDDMDDYCTGISSDCPR.N
2149.8364	2148.8291	2148.8035	11.9	42 -	60	0	---	R.AVGDDMDDYCTGISSDCPR.N + Oxidation (M)

No match to: 1029.5132, 1074.5952, 1106.5680, 1108.5847, 1119.5947, 1121.6051, 1146.5963, 1148.5788, 1150.5916, 1162.5933, 1165.6061, 1176.6084, 1179.6202, 1187.5818, 1189.6030, 1191.6211, 1197.5771, 1207.6162, 1261.5168, 1317.6060, 1332.5510, 1383.6258, 1397.4491, 1410.6107, 1417.6152, 1455.6313, 1459.9186, 1467.6321, 1471.6241, 1485.6406, 1488.6529, 1496.6199, 1500.6537, 1516.6503, 1524.6495, 1542.6655, 1567.7730, 1583.6915, 1608.8002, 1647.7150, 1697.7476, 1715.7585, 1742.7690, 1761.7738, 1772.7726, 1776.7684, 1787.7767, 1802.7917, 1817.7702, 1826.7647, 1828.7826, 1830.7847, 1834.7863, 1844.7780, 1846.7894, 1848.7918, 1862.7775, 1869.7501, 1872.7776, 1875.7943, 1885.7714, 1889.7890, 1892.8182, 1903.7892, 1904.7346, 1905.7619, 1906.7841, 1908.8129, 1914.7625, 1916.7465, 1918.2815, 1918.7903, 1921.7303, 1934.8231, 1943.7515, 1948.7478, 1950.8218, 1958.7646, 1960.7335, 1962.7632, 1975.7723, 1978.7513, 1984.7457, 1988.7698, 1992.7606, 2000.6976, 2004.7706, 2019.7737, 2189.8910, 2205.8830, 2227.0072, 2245.0161

9. VM25A_MACLO **Mass:** 7570 **Score:** 214 **Expect:** 5.6e-019 **Matches:** 4

Disintegrin VLO5A OS=Macrovipera lebetina obtusa OX=209528 PE=1 SV=1

Observed	Mr(expt)	Mr(calc)	ppm	Start	End	Miss	Ions	Peptide
1029.5132	1028.5059	1028.4822	23.1	16 -	24	1	---	R.RGEHCVSGK.C
1761.7738	1760.7666	1760.7029	36.2	1 -	15	0	80	-.NSGNPCCDPVTCQPR.R
2133.8517	2132.8444	2132.8085	16.8	41 -	59	0	110	R.AVGDDMDDYCTGISSDCPR.N
2149.8364	2148.8291	2148.8035	11.9	41 -	59	0	---	R.AVGDDMDDYCTGISSDCPR.N + Oxidation (M)

No match to: 1074.5952, 1106.5680, 1108.5847, 1119.5947, 1121.6051, 1146.5963, 1148.5788, 1150.5916, 1162.5933, 1165.6061, 1176.6084, 1179.6202, 1187.5818, 1189.6030, 1191.6211, 1197.5771, 1207.6162, 1261.5168, 1317.6060, 1318.5355, 1332.5510, 1383.6258, 1397.4491, 1410.6107, 1417.6152, 1455.6313, 1459.9186, 1467.6321, 1471.6241, 1474.6378, 1485.6406, 1488.6529, 1496.6199, 1500.6537, 1516.6503, 1524.6495, 1542.6655, 1567.7730, 1583.6915, 1608.8002, 1647.7150, 1697.7476, 1715.7585, 1742.7690, 1772.7726, 1776.7684, 1787.7767, 1802.7917, 1817.7702, 1826.7647, 1828.7826, 1830.7847, 1834.7863, 1844.7780, 1846.7894, 1848.7918, 1862.7775, 1869.7501, 1872.7776, 1875.7943, 1885.7714, 1889.7890, 1892.8182, 1903.7892, 1904.7346, 1905.7619, 1906.7841, 1908.8129, 1914.7625, 1916.7465, 1918.2815, 1918.7903, 1921.7303, 1934.8231, 1943.7515, 1945.7522, 1948.7478, 1950.8218, 1958.7646, 1960.7335, 1962.7632, 1975.7723, 1978.7513, 1984.7457, 1988.7698, 1992.7606, 2000.6976, 2004.7706, 2019.7737, 2189.8910, 2205.8830, 2227.0072, 2245.0161

10. DIDB_CERVI **Mass:** 7584 **Score:** 188 **Expect:** 2.2e-016 **Matches:** 7

Disintegrin CV-11-beta (Fragment) OS=Cerastes vipera OX=8698 PE=2 SV=1

Observed	Mr(expt)	Mr(calc)	ppm	Start	End	Miss	Ions	Peptide
1150.5916	1149.5843	1149.6216	-32.43	31	40	1	---	K.FLSPGTICKK.A
1332.5510	1331.5437	1331.5169	20.1	17	27	0	---	R.GEHCISGPCCR.N
1488.6529	1487.6456	1487.6180	18.5	16	27	1	---	K.RGEHCISGPCCR.N
1962.7632	1961.7559	1961.7190	18.8	43	59	0	142	R.GDDMNDYCTGISSDCPR.N
1978.7513	1977.7441	1977.7139	15.2	43	59	0	(118)	R.GDDMNDYCTGISSDCPR.N + Oxidation (M)
2189.8910	2188.8837	2188.8572	12.1	41	59	1	---	K.ARGDDMNDYCTGISSDCPR.N
2205.8830	2204.8758	2204.8521	10.7	41	59	1	---	K.ARGDDMNDYCTGISSDCPR.N + Oxidation (M)

No match to: 1029.5132, 1074.5952, 1106.5680, 1108.5847, 1119.5947, 1121.6051, 1146.5963, 1148.5788, 1162.5933, 1165.6061, 1176.6084, 1179.6202, 1187.5818, 1189.6030, 1191.6211, 1197.5771, 1207.6162, 1261.5168, 1317.6060, 1318.5355, 1383.6258, 1397.4491, 1410.6107, 1417.6152, 1455.6313, 1459.9186, 1467.6321, 1471.6241, 1474.6378, 1485.6406, 1496.6199, 1500.6537, 1516.6503, 1524.6495, 1542.6655, 1567.7730, 1583.6915, 1608.8002, 1647.7150, 1697.7476, 1715.7585, 1742.7690, 1761.7738, 1772.7726, 1776.7684, 1787.7767, 1802.7917, 1817.7702, 1826.7647, 1828.7826, 1830.7847, 1834.7863, 1844.7780, 1846.7894, 1848.7918, 1862.7775, 1869.7501, 1872.7776, 1875.7943, 1885.7714, 1889.7890, 1892.8182, 1903.7892, 1904.7346, 1905.7619, 1906.7841, 1908.8129, 1914.7625, 1916.7465, 1918.2815, 1918.7903, 1921.7303, 1934.8231, 1943.7515, 1945.7522, 1948.7478, 1950.8218, 1958.7646, 1960.7335, 1975.7723, 1984.7457, 1988.7698, 1992.7606, 2000.6976, 2004.7706, 2019.7737, 2133.8517, 2149.8364, 2227.0072, 2245.0161

11. DIDA_CERVI **Mass:** 12717 **Score:** 169 **Expect:** 1.8e-014 **Matches:** 5

Disintegrin CV-11-alpha OS=Cerastes vipera OX=8698 PE=2 SV=1

Observed	Mr(expt)	Mr(calc)	ppm	Start	End	Miss	Ions	Peptide
1150.5916	1149.5843	1149.6216	-32.43	78	87	1	---	K.FLSPGTICKK.A
1332.5510	1331.5437	1331.5169	20.1	64	74	0	---	R.GEHCISGPCCR.N
1488.6529	1487.6456	1487.6180	18.5	63	74	1	---	K.RGEHCISGPCCR.N
1962.7632	1961.7559	1961.7190	18.8	90	106	0	142	K.GDDMNDYCTGISSDCPR.N
1978.7513	1977.7441	1977.7139	15.2	90	106	0	(118)	K.GDDMNDYCTGISSDCPR.N + Oxidation (M)

No match to: 1029.5132, 1074.5952, 1106.5680, 1108.5847, 1119.5947, 1121.6051, 1146.5963, 1148.5788, 1162.5933, 1165.6061, 1176.6084, 1179.6202, 1187.5818, 1189.6030, 1191.6211, 1197.5771, 1207.6162, 1261.5168, 1317.6060, 1318.5355, 1383.6258, 1397.4491, 1410.6107, 1417.6152, 1455.6313, 1459.9186, 1467.6321, 1471.6241, 1474.6378, 1485.6406, 1496.6199, 1500.6537, 1516.6503, 1524.6495, 1542.6655, 1567.7730, 1583.6915, 1608.8002, 1647.7150, 1697.7476, 1715.7585, 1742.7690, 1761.7738, 1772.7726, 1776.7684, 1787.7767, 1802.7917, 1817.7702, 1826.7647, 1828.7826, 1830.7847, 1834.7863, 1844.7780, 1846.7894, 1848.7918, 1862.7775, 1869.7501, 1872.7776, 1875.7943, 1885.7714, 1889.7890, 1892.8182, 1903.7892, 1904.7346, 1905.7619, 1906.7841, 1908.8129, 1914.7625, 1916.7465, 1918.2815, 1918.7903, 1921.7303, 1934.8231, 1943.7515, 1945.7522, 1948.7478, 1950.8218, 1958.7646, 1960.7335, 1975.7723, 1984.7457, 1988.7698, 1992.7606, 2000.6976, 2004.7706, 2019.7737, 2133.8517, 2149.8364, 2189.8910, 2205.8830, 2227.0072, 2245.0161

12. VM2L2_MACLB **Mass:** 54586 **Score:** 120 **Expect:** 1.4e-009 **Matches:** 5

Zinc metalloproteinase/disintegrin OS=Macrovipera lebetina OX=8709 PE=1 SV=1

Observed	Mr(expt)	Mr(calc)	ppm	Start	End	Miss	Ions	Peptide
1029.5132	1028.5059	1028.4822	23.1	430	438	1	---	R.RGEHCVSGK.C
1162.5933	1161.5860	1161.6281	-36.23	136	145	0	---	R.GETYLIEPLK.L
1958.7646	1957.7573	1957.8806	-62.98	78	94	1	---	R.GLFSKDYSETHYSPDGR.E
2133.8517	2132.8444	2132.8085	16.8	455	473	0	110	R.AVGDDMDDYCTGISSDCPR.N
2149.8364	2148.8291	2148.8035	11.9	455	473	0	---	R.AVGDDMDDYCTGISSDCPR.N + Oxidation (M)

No match to: 1074.5952, 1106.5680, 1108.5847, 1119.5947, 1121.6051, 1146.5963, 1148.5788, 1150.5916, 1165.6061, 1176.6084, 1179.6202, 1187.5818, 1189.6030, 1191.6211, 1197.5771, 1207.6162, 1261.5168, 1317.6060, 1318.5355, 1332.5510, 1383.6258, 1397.4491, 1410.6107, 1417.6152, 1455.6313, 1459.9186, 1467.6321, 1471.6241, 1474.6378, 1485.6406, 1488.6529, 1496.6199, 1500.6537, 1516.6503, 1524.6495, 1542.6655, 1567.7730, 1583.6915, 1608.8002, 1647.7150, 1697.7476, 1715.7585, 1742.7690, 1761.7738, 1772.7726, 1776.7684, 1787.7767, 1802.7917, 1817.7702, 1826.7647, 1828.7826, 1830.7847, 1834.7863, 1844.7780, 1846.7894, 1848.7918, 1862.7775, 1869.7501, 1872.7776, 1875.7943, 1885.7714, 1889.7890, 1892.8182, 1903.7892, 1904.7346, 1905.7619, 1906.7841, 1908.8129, 1914.7625, 1916.7465, 1918.2815, 1918.7903, 1921.7303, 1934.8231, 1943.7515, 1945.7522, 1948.7478, 1950.8218, 1960.7335, 1962.7632, 1975.7723, 1978.7513, 1984.7457, 1988.7698, 1992.7606, 2000.6976, 2004.7706, 2019.7737, 2189.8910, 2205.8830, 2227.0072, 2245.0161

13. DID5B_MACLO **Mass:** 8235 **Score:** 116 **Expect:** 3.6e-009 **Matches:** 3

Disintegrin VLO5B OS=Macrovipera lebetina obtusa OX=209528 PE=1 SV=1

Observed	Mr(expt)	Mr(calc)	ppm	Start	End	Miss	Ions	Peptide
1318.5355	1317.5282	1317.5013	20.4	18	28	0	34	R.GEHCVSGPCCR.N
1474.6378	1473.6305	1473.6024	19.1	17	28	1	60	R.RGEHCVSGPCCR.N
1945.7522	1944.7449	1944.8717	-65.19	2	17	1	---	M.NSANPCCDPITCKPRR.G

No match to: 1029.5132, 1074.5952, 1106.5680, 1108.5847, 1119.5947, 1121.6051, 1146.5963, 1148.5788, 1150.5916, 1162.5933, 1165.6061, 1176.6084, 1179.6202, 1187.5818, 1189.6030, 1191.6211, 1197.5771, 1207.6162, 1261.5168, 1317.6060, 1332.5510, 1383.6258, 1397.4491, 1410.6107, 1417.6152, 1455.6313, 1459.9186, 1467.6321, 1471.6241, 1485.6406, 1488.6529, 1496.6199, 1500.6537, 1516.6503, 1524.6495, 1542.6655, 1567.7730, 1583.6915, 1608.8002, 1647.7150, 1697.7476, 1715.7585, 1742.7690, 1761.7738, 1772.7726, 1776.7684, 1787.7767, 1802.7917, 1817.7702, 1826.7647, 1828.7826, 1830.7847, 1834.7863, 1844.7780, 1846.7894, 1848.7918, 1862.7775, 1869.7501, 1872.7776, 1875.7943, 1885.7714, 1889.7890, 1892.8182, 1903.7892, 1904.7346, 1905.7619, 1906.7841, 1908.8129, 1914.7625, 1916.7465, 1918.2815, 1918.7903, 1921.7303, 1934.8231, 1943.7515, 1948.7478, 1950.8218, 1958.7646, 1960.7335, 1962.7632, 1975.7723, 1978.7513, 1984.7457, 1988.7698, 1992.7606, 2000.6976, 2004.7706, 2019.7737, 2133.8517, 2149.8364, 2189.8910, 2205.8830, 2227.0072, 2245.0161

14. A0A1I9KNR4_VIPAA **Mass:** 14601 **Score:** 43 **Expect:** 0.068 **Matches:** 2

Disintegrin Dis-1 OS=Vipera ammodytes ammodytes OX=8705 PE=2 SV=1

Observed	Mr(expt)	Mr(calc)	ppm	Start	End	Miss	Ions	Peptide
1150.5916	1149.5843	1149.5852	-0.78	118	128	1	---	K.WSATAKGSVLM.-
1318.5355	1317.5282	1317.5013	20.4	64	74	0	34	K.GEHCVSGPCCR.N

No match to: 1029.5132, 1074.5952, 1106.5680, 1108.5847, 1119.5947, 1121.6051, 1146.5963, 1148.5788, 1162.5933, 1165.6061, 1176.6084, 1179.6202, 1187.5818, 1189.6030, 1191.6211, 1197.5771, 1207.6162, 1261.5168, 1317.6060, 1332.5510, 1383.6258, 1397.4491, 1410.6107, 1417.6152, 1455.6313, 1459.9186, 1467.6321, 1471.6241, 1474.6378, 1485.6406, 1488.6529, 1496.6199, 1500.6537, 1516.6503, 1524.6495, 1542.6655, 1567.7730, 1583.6915, 1608.8002, 1647.7150, 1697.7476, 1715.7585, 1742.7690, 1761.7738, 1772.7726, 1776.7684, 1787.7767, 1802.7917, 1817.7702, 1826.7647, 1828.7826, 1830.7847, 1834.7863, 1844.7780, 1846.7894, 1848.7918, 1862.7775, 1869.7501, 1872.7776, 1875.7943, 1885.7714, 1889.7890, 1892.8182, 1903.7892, 1904.7346, 1905.7619, 1906.7841, 1908.8129, 1914.7625, 1916.7465, 1918.2815, 1918.7903, 1921.7303, 1934.8231, 1943.7515, 1945.7522, 1948.7478, 1950.8218, 1958.7646, 1960.7335, 1962.7632, 1975.7723, 1978.7513, 1984.7457, 1988.7698, 1992.7606, 2000.6976, 2004.7706, 2019.7737, 2133.8517, 2149.8364, 2189.8910, 2205.8830, 2227.0072, 2245.0161

15. DID2A_MACLB **Mass:** 14638 **Score:** 43 **Expect:** 0.068 **Matches:** 2

Disintegrin lebein-2-alpha OS=Macrovipera lebetina OX=8709 PE=1 SV=1

Observed	Mr(expt)	Mr(calc)	ppm	Start	End	Miss	Ions	Peptide
1150.5916	1149.5843	1149.5852	-0.78	118	128	1	---	K.WSATAKGSVLM.-
1318.5355	1317.5282	1317.5013	20.4	64	74	0	34	K.GEHCVSGPCCR.N

No match to: 1029.5132, 1074.5952, 1106.5680, 1108.5847, 1119.5947, 1121.6051, 1146.5963, 1148.5788, 1162.5933, 1165.6061, 1176.6084, 1179.6202, 1187.5818, 1189.6030, 1191.6211, 1197.5771, 1207.6162, 1261.5168, 1317.6060, 1332.5510, 1383.6258, 1397.4491, 1410.6107, 1417.6152, 1455.6313, 1459.9186, 1467.6321, 1471.6241, 1474.6378, 1485.6406, 1488.6529, 1496.6199, 1500.6537, 1516.6503, 1524.6495, 1542.6655, 1567.7730, 1583.6915, 1608.8002, 1647.7150, 1697.7476, 1715.7585, 1742.7690, 1761.7738, 1772.7726, 1776.7684, 1787.7767, 1802.7917, 1817.7702, 1826.7647, 1828.7826, 1830.7847, 1834.7863, 1844.7780, 1846.7894, 1848.7918, 1862.7775, 1869.7501, 1872.7776, 1875.7943, 1885.7714, 1889.7890, 1892.8182, 1903.7892, 1904.7346, 1905.7619, 1906.7841, 1908.8129, 1914.7625, 1916.7465, 1918.2815, 1918.7903, 1921.7303, 1934.8231, 1943.7515, 1945.7522, 1948.7478, 1950.8218, 1958.7646, 1960.7335, 1962.7632, 1975.7723, 1978.7513, 1984.7457, 1988.7698, 1992.7606, 2000.6976, 2004.7706, 2019.7737, 2133.8517, 2149.8364, 2189.8910, 2205.8830, 2227.0072, 2245.0161

16. A0A1I9KNR7_VIPAA **Mass:** 14615 **Score:** 38 **Expect:** 0.2 **Matches:** 1

Disintegrin Dis-1 OS=Vipera ammodytes ammodytes OX=8705 PE=2 SV=1

Observed	Mr(expt)	Mr(calc)	ppm	Start	End	Miss	Ions	Peptide
1318.5355	1317.5282	1317.5013	20.4	64	74	0	34	K.GEHCVSGPCCR.N

No match to: 1029.5132, 1074.5952, 1106.5680, 1108.5847, 1119.5947, 1121.6051, 1146.5963, 1148.5788, 1150.5916, 1162.5933, 1165.6061, 1176.6084, 1179.6202, 1187.5818, 1189.6030, 1191.6211, 1197.5771, 1207.6162, 1261.5168, 1317.6060, 1332.5510, 1383.6258, 1397.4491, 1410.6107, 1417.6152, 1455.6313, 1459.9186, 1467.6321, 1471.6241, 1474.6378, 1485.6406, 1488.6529, 1496.6199, 1500.6537, 1516.6503, 1524.6495, 1542.6655, 1567.7730, 1583.6915, 1608.8002, 1647.7150, 1697.7476, 1715.7585, 1742.7690, 1761.7738, 1772.7726, 1776.7684, 1787.7767, 1802.7917, 1817.7702, 1826.7647, 1828.7826, 1830.7847, 1834.7863, 1844.7780, 1846.7894, 1848.7918, 1862.7775, 1869.7501, 1872.7776, 1875.7943, 1885.7714, 1889.7890, 1892.8182, 1903.7892, 1904.7346, 1905.7619, 1906.7841, 1908.8129, 1914.7625, 1916.7465, 1918.2815, 1918.7903, 1921.7303, 1934.8231, 1943.7515, 1945.7522, 1948.7478, 1950.8218, 1958.7646, 1960.7335, 1962.7632, 1975.7723, 1978.7513, 1984.7457, 1988.7698, 1992.7606, 2000.6976, 2004.7706, 2019.7737, 2133.8517, 2149.8364, 2189.8910, 2205.8830, 2227.0072, 2245.0161

17. TXVE2_MACLB **Mass:** 13022 **Score:** 19 **Expect:** 19 **Matches:** 4

Snake venom vascular endothelial growth factor toxin ICPP OS=Macrovipera lebetina OX=8709 PE=1 SV=2

Observed	Mr(expt)	Mr(calc)	ppm	Start	End	Miss	Ions	Peptide
1146.5963	1145.5890	1145.5322	49.6	62	70	0	---	K.HTVDMQIMR.V + Oxidation (M)
1162.5933	1161.5860	1161.5271	50.8	62	70	0	---	K.HTVDMQIMR.V + 2 Oxidation (M)
1772.7726	1771.7653	1771.8532	-49.60	56	70	1	---	K.CTPVGKHTVDMQIMR.V

1958.7646	1957.7573	1957.8002	-21.93	45	- 61	1	---	R.CSGCCTDESLKCTPVGK.H

No match to: 1029.5132, 1074.5952, 1106.5680, 1108.5847, 1119.5947, 1121.6051, 1148.5788, 1150.5916, 1165.6061, 1176.6084, 1179.6202, 1187.5818, 1189.6030, 1191.6211, 1197.5771, 1207.6162, 1261.5168, 1317.6060, 1318.5355, 1332.5510, 1383.6258, 1397.4491, 1410.6107, 1417.6152, 1455.6313, 1459.9186, 1467.6321, 1471.6241, 1474.6378, 1485.6406, 1488.6529, 1496.6199, 1500.6537, 1516.6503, 1524.6495, 1542.6655, 1567.7730, 1583.6915, 1608.8002, 1647.7150, 1697.7476, 1715.7585, 1742.7690, 1761.7738, 1776.7684, 1787.7767, 1802.7917, 1817.7702, 1826.7647, 1828.7826, 1830.7847, 1834.7863, 1844.7780, 1846.7894, 1848.7918, 1862.7775, 1869.7501, 1872.7776, 1875.7943, 1885.7714, 1889.7890, 1892.8182, 1903.7892, 1904.7346, 1905.7619, 1906.7841, 1908.8129, 1914.7625, 1916.7465, 1918.2815, 1918.7903, 1921.7303, 1934.8231, 1943.7515, 1945.7522, 1948.7478, 1950.8218, 1960.7335, 1962.7632, 1975.7723, 1978.7513, 1984.7457, 1988.7698, 1992.7606, 2000.6976, 2004.7706, 2019.7737, 2133.8517, 2149.8364, 2189.8910, 2205.8830, 2227.0072, 2245.0161

18. K4GS84_DABRR **Mass:** 37760 **Score:** 18 **Expect:** 20 **Matches:** 5

Solute carrier family 8 member 1 (Fragment) OS=Daboia russelii OX=8707 GN=SLC8A1 PE=4 SV=1

Observed	Mr(expt)	Mr(calc)	ppm	Start	End	Miss	Ions	Peptide
1189.6030	1188.5957	1188.6649	-58.17	295	- 305	1	---	R.LMTGAGNILKR.H + Oxidation (M)
1317.6060	1316.5987	1316.7048	-80.61	41	- 52	1	---	K.KPNGETSKTTVR.I
1496.6199	1495.6126	1495.6977	-56.85	197	- 210	0	---	R.GMIIEHEGEGPPSK.A + Oxidation (M)
1542.6655	1541.6582	1541.7395	-52.76	23	- 35	0	---	R.FMSSIEVITSQER.E + Oxidation (M)
2245.0161	2244.0088	2244.0151	-2.78	313	- 332	1	---	R.KAVSMHEVNCEVADNDPVSK.- + Oxidation (M)

No match to: 1029.5132, 1074.5952, 1106.5680, 1108.5847, 1119.5947, 1121.6051, 1146.5963, 1148.5788, 1150.5916, 1162.5933, 1165.6061, 1176.6084, 1179.6202, 1187.5818, 1191.6211, 1197.5771, 1207.6162, 1261.5168, 1318.5355, 1332.5510, 1383.6258, 1397.4491, 1410.6107, 1417.6152, 1455.6313, 1459.9186, 1467.6321, 1471.6241, 1474.6378, 1485.6406, 1488.6529, 1500.6537, 1516.6503, 1524.6495, 1567.7730, 1583.6915, 1608.8002, 1647.7150, 1697.7476, 1715.7585, 1742.7690, 1761.7738, 1772.7726, 1776.7684, 1787.7767, 1802.7917, 1817.7702, 1826.7647, 1828.7826, 1830.7847, 1834.7863, 1844.7780, 1846.7894, 1848.7918, 1862.7775, 1869.7501, 1872.7776, 1875.7943, 1885.7714, 1889.7890, 1892.8182, 1903.7892, 1904.7346, 1905.7619, 1906.7841, 1908.8129, 1914.7625, 1916.7465, 1918.2815, 1918.7903, 1921.7303, 1934.8231, 1943.7515, 1945.7522, 1948.7478, 1950.8218, 1958.7646, 1960.7335, 1962.7632, 1975.7723, 1978.7513, 1984.7457, 1988.7698, 1992.7606, 2000.6976, 2004.7706, 2019.7737, 2133.8517, 2149.8364, 2189.8910, 2205.8830, 2227.0072

19. Q8UVG8_DABRR **Mass:** 52931 **Score:** 18 **Expect:** 22 **Matches:** 6

Vimentin OS=Daboia russelii OX=8707 PE=2 SV=1

Observed	Mr(expt)	Mr(calc)	ppm	Start	End	Miss	Ions	Peptide
1029.5132	1028.5059	1028.5363	-29.53	300	- 308	1	---	R.NNDALRQAK.Q
1148.5788	1147.5716	1147.5543	15.1	360	- 368	0	---	R.LQEEIQNMK.E + Oxidation (M)
1524.6495	1523.6422	1523.7256	-54.71	278	- 289	1	---	K.NLQEAEEWYKSK.F
1583.6915	1582.6842	1582.7838	-62.95	420	- 433	0	---	R.ETNLESIPIDTHSK.R
1776.7684	1775.7611	1775.8550	-52.89	290	- 305	1	---	K.FADLSEAANRNNDALR.Q
1914.7625	1913.7552	1913.9378	-95.40	18	- 35	1	---	R.MFGSGRPSSTRSVTSTTR.Y

No match to: 1074.5952, 1106.5680, 1108.5847, 1119.5947, 1121.6051, 1146.5963, 1150.5916, 1162.5933, 1165.6061, 1176.6084, 1179.6202, 1187.5818, 1189.6030, 1191.6211, 1197.5771, 1207.6162, 1261.5168, 1317.6060, 1318.5355, 1332.5510, 1383.6258, 1397.4491, 1410.6107, 1417.6152, 1455.6313, 1459.9186, 1467.6321, 1471.6241, 1474.6378, 1485.6406, 1488.6529, 1496.6199, 1500.6537, 1516.6503, 1542.6655, 1567.7730, 1608.8002, 1647.7150, 1697.7476, 1715.7585, 1742.7690, 1761.7738, 1772.7726, 1787.7767, 1802.7917, 1817.7702, 1826.7647, 1828.7826, 1830.7847, 1834.7863, 1844.7780, 1846.7894, 1848.7918, 1862.7775, 1869.7501, 1872.7776, 1875.7943, 1885.7714, 1889.7890, 1892.8182, 1903.7892, 1904.7346, 1905.7619, 1906.7841, 1908.8129, 1916.7465, 1918.2815, 1918.7903, 1921.7303, 1934.8231, 1943.7515, 1945.7522, 1948.7478, 1950.8218, 1958.7646, 1960.7335, 1962.7632, 1975.7723, 1978.7513, 1984.7457, 1988.7698, 1992.7606, 2000.6976, 2004.7706, 2019.7737, 2133.8517, 2149.8364, 2189.8910, 2205.8830, 2227.0072, 2245.0161

20. A0A6G5ZW48_9SAUR **Mass:** 86121 **Score:** 18 **Expect:** 22 **Matches:** 8

Atriopeptidase OS=Vipera anatolica senliki OX=2604287 PE=2 SV=1

Observed	Mr(expt)	Mr(calc)	ppm	Start	End	Miss	Ions	Peptide
1108.5847	1107.5774	1107.5342	39.0	59	- 68	1	---	K.TSDCIKSAAR.I
1162.5933	1161.5860	1161.6618	-65.24	671	- 680	1	---	K.RLPGLDLNHK.Q
1317.6060	1316.5987	1316.6104	-8.93	372	- 382	0	---	R.YIMDMVSSLSR.E + Oxidation (M)
1516.6503	1515.6430	1515.7542	-73.38	208	- 220	0	---	K.NSSQHVLHVDQPR.L
1567.7730	1566.7657	1566.7599	3.70	451	- 463	1	---	K.TLEELSWMDAKTK.K + Oxidation (M)
1608.8002	1607.7930	1607.8420	-30.49	394	- 407	1	---	K.VLYGTTSDAAIWRR.C
1934.8231	1933.8158	1933.9244	-56.15	221	- 236	1	---	R.LGLPSRDYYECTGLYK.E
2245.0161	2244.0088	2244.0950	-38.38	496	- 513	1	---	K.GLNYKEEEYFENLIENLK.F

No match to: 1029.5132, 1074.5952, 1106.5680, 1119.5947, 1121.6051, 1146.5963, 1148.5788, 1150.5916, 1165.6061, 1176.6084, 1179.6202, 1187.5818, 1189.6030, 1191.6211, 1197.5771, 1207.6162, 1261.5168, 1318.5355, 1332.5510, 1383.6258, 1397.4491, 1410.6107, 1417.6152, 1455.6313, 1459.9186, 1467.6321, 1471.6241, 1474.6378, 1485.6406, 1488.6529, 1496.6199, 1500.6537, 1524.6495, 1542.6655, 1583.6915, 1647.7150, 1697.7476, 1715.7585, 1742.7690, 1761.7738, 1772.7726, 1776.7684, 1787.7767, 1802.7917, 1817.7702, 1826.7647, 1828.7826, 1830.7847, 1834.7863, 1844.7780, 1846.7894, 1848.7918, 1862.7775, 1869.7501, 1872.7776, 1875.7943, 1885.7714, 1889.7890, 1892.8182, 1903.7892, 1904.7346, 1905.7619, 1906.7841, 1908.8129, 1914.7625, 1916.7465, 1918.2815, 1918.7903, 1921.7303, 1943.7515, 1945.7522, 1948.7478, 1950.8218, 1958.7646, 1960.7335, 1962.7632, 1975.7723, 1978.7513, 1984.7457, 1988.7698, 1992.7606, 2000.6976, 2004.7706, 2019.7737, 2133.8517, 2149.8364, 2189.8910, 2205.8830, 2227.0072

Search Parameters

```
Type of search         : MS/MS Ion Search
Enzyme                 : Trypsin
Fixed modifications    : Carbamidomethyl (C)
Variable modifications : Oxidation (M)
Mass values            : Monoisotopic
Protein Mass           : Unrestricted
Peptide Mass Tolerance : ± 100 ppm
Fragment Mass Tolerance: ± 0.7 Da
Max Missed Cleavages   : 1
Instrument type        : MALDI-TOF-TOF
Query1  (1029.5132,1+) : <no title>
Query2  (1074.5952,1+) : <no title>
Query3  (1106.5680,1+) : <no title>
Query4  (1108.5847,1+) : <no title>
Query5  (1119.5947,1+) : <no title>
Query6  (1121.6051,1+) : <no title>
Query7  (1146.5963,1+) : <no title>
Query8  (1148.5788,1+) : <no title>
Query9  (1150.5916,1+) : <no title>
Query10 (1162.5933,1+) : <no title>
Query11 (1165.6061,1+) : \\PROTEOMICS1\D\DATA\2105\210505_TLIEPOL_1_68252-68266\68252_68266_LPARDO\0_G6\1\1165.6061.LIFT\1SREF\PDATA\1\1R
Query12 (1176.6084,1+) : <no title>
Query13 (1179.6202,1+) : <no title>
Query14 (1187.5818,1+) : <no title>
Query15 (1189.6030,1+) : <no title>
Query16 (1191.6211,1+) : \\PROTEOMICS1\D\DATA\2105\210505_TLIEPOL_1_68252-68266\68252_68266_LPARDO\0_G6\1\1191.6211.LIFT\1SREF\PDATA\1\1R
Query17 (1197.5771,1+) : <no title>
Query18 (1207.6162,1+) : <no title>
Query19 (1261.5168,1+) : <no title>
Query20 (1317.6060,1+) : <no title>
Query21 (1318.5355,1+) : \\PROTEOMICS1\D\DATA\2105\210505_TLIEPOL_1_68252-68266\68252_68266_LPARDO\0_G6\1\1318.5355.LIFT\1SREF\PDATA\1\1R
Query22 (1332.5510,1+) : <no title>
Query23 (1383.6258,1+) : <no title>
Query24 (1397.4491,1+) : <no title>
Query25 (1410.6107,1+) : <no title>
Query26 (1417.6152,1+) : <no title>
Query27 (1455.6313,1+) : <no title>
Query28 (1459.9186,1+) : <no title>
Query29 (1467.6321,1+) : <no title>
Query30 (1471.6241,1+) : <no title>
Query31 (1474.6378,1+) : \\PROTEOMICS1\D\DATA\2105\210505_TLIEPOL_1_68252-68266\68252_68266_LPARDO\0_G6\1\1474.6378.LIFT\1SREF\PDATA\1\1R
Query32 (1485.6406,1+) : <no title>
Query33 (1488.6529,1+) : <no title>
Query34 (1496.6199,1+) : <no title>
Query35 (1500.6537,1+) : <no title>
Query36 (1516.6503,1+) : <no title>
Query37 (1524.6495,1+) : <no title>
Query38 (1542.6655,1+) : <no title>
Query39 (1567.7730,1+) : <no title>
Query40 (1583.6915,1+) : <no title>
Query41 (1608.8002,1+) : <no title>
Query42 (1647.7150,1+) : <no title>
Query43 (1697.7476,1+) : <no title>
Query44 (1715.7585,1+) : <no title>
Query45 (1742.7690,1+) : <no title>
Query46 (1761.7738,1+) : \\PROTEOMICS1\D\DATA\2105\210505_TLIEPOL_1_68252-68266\68252_68266_LPARDO\0_G6\1\1761.7738.LIFT\1SREF\PDATA\1\1R
Query47 (1772.7726,1+) : <no title>
```

```
Query48 (1776.7684,1+) : <no title>
Query49 (1787.7767,1+) : <no title>
Query50 (1802.7917,1+) : <no title>
Query51 (1817.7702,1+) : <no title>
Query52 (1826.7647,1+) : <no title>
Query53 (1828.7826,1+) : <no title>
Query54 (1830.7847,1+) : <no title>
Query55 (1834.7863,1+) : <no title>
Query56 (1844.7780,1+) : <no title>
Query57 (1846.7894,1+) : <no title>
Query58 (1848.7918,1+) : <no title>
Query59 (1862.7775,1+) : <no title>
Query60 (1869.7501,1+) : <no title>
Query61 (1872.7776,1+) : <no title>
Query62 (1875.7943,1+) : <no title>
Query63 (1885.7714,1+) : <no title>
Query64 (1889.7890,1+) : <no title>
Query65 (1892.8182,1+) : \\PROTEOMICS1\D\DATA\2105\210505_TLIEPOL_1_68252-68266\68252_68266_LPARDO\0_G6\1\1892.8182.LIFT\1SREF\PDATA\1\1R
Query66 (1903.7892,1+) : <no title>
Query67 (1904.7346,1+) : <no title>
Query68 (1905.7619,1+) : <no title>
Query69 (1906.7841,1+) : <no title>
Query70 (1908.8129,1+) : <no title>
Query71 (1914.7625,1+) : <no title>
Query72 (1916.7465,1+) : <no title>
Query73 (1918.2815,1+) : <no title>
Query74 (1918.7903,1+) : <no title>
Query75 (1921.7303,1+) : <no title>
Query76 (1934.8231,1+) : <no title>
Query77 (1943.7515,1+) : <no title>
Query78 (1945.7522,1+) : <no title>
Query79 (1948.7478,1+) : <no title>
Query80 (1950.8218,1+) : <no title>
Query81 (1958.7646,1+) : <no title>
Query82 (1960.7335,1+) : <no title>
Query83 (1962.7632,1+) : \\PROTEOMICS1\D\DATA\2105\210505_TLIEPOL_1_68252-68266\68252_68266_LPARDO\0_G6\1\1962.7632.LIFT\1SREF\PDATA\1\1R
Query84 (1975.7723,1+) : <no title>
Query85 (1978.7513,1+) : \\PROTEOMICS1\D\DATA\2105\210505_TLIEPOL_1_68252-68266\68252_68266_LPARDO\0_G6\1\1978.7513.LIFT\1SREF\PDATA\1\1R
Query86 (1984.7457,1+) : <no title>
Query87 (1988.7698,1+) : <no title>
Query88 (1992.7606,1+) : <no title>
Query89 (2000.6976,1+) : <no title>
Query90 (2004.7706,1+) : <no title>
Query91 (2019.7737,1+) : <no title>
Query92 (2133.8517,1+) : \\PROTEOMICS1\D\DATA\2105\210505_TLIEPOL_1_68252-68266\68252_68266_LPARDO\0_G6\1\2133.8517.LIFT\1SREF\PDATA\1\1R
Query93 (2149.8364,1+) : <no title>
Query94 (2189.8910,1+) : <no title>
Query95 (2205.8830,1+) : <no title>
Query96 (2227.0072,1+) : <no title>
Query97 (2245.0161,1+) : <no title>
```

Mascot: http://www.matrixscience.com/

Mascot Search Results **Fr3-1**

User :
Email :
Search title : vipera_All_8IDplus4 file:\\Proteomics1\d\data\2105\210505_tliepol_1_68252-68266\68252_68266_lpardo\0_H6\1\1SRef\pdata\1\peaklist.xml
Database : vipera (1414 sequences; 347452 residues)
Timestamp : 5 May 2021 at 09:34:03 GMT
Top Score : 57 for DID7A_VIPBB, Disintegrin VB7A OS=Vipera berus berus OX=31156 PE=1 SV=1

Mascot Score Histogram

Protein score is -10*Log(P), where P is the probability that the observed match is a random event.
Protein scores greater than 44 are significant (p<0.05).

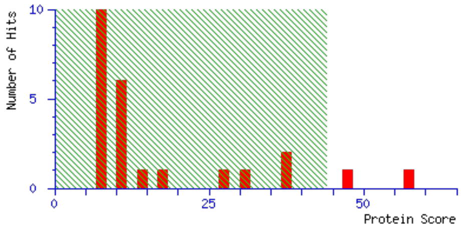

Concise Protein Summary Report

Format As | Concise Protein Summary | Help
Significance threshold p< 0.05 | Max. number of hits 10

Re-Search All | Search Unmatched

1. DID7A_VIPBB **Mass:** 7574 **Score:** 57 **Expect:** 0.0026 **Matches:** 5
Disintegrin VB7A OS=Vipera berus berus OX=31156 PE=1 SV=1
VM2A6_VIPAA **Mass:** 7588 **Score:** 40 **Expect:** 0.14 **Matches:** 4
Disintegrin VA6 OS=Vipera ammodytes ammodytes OX=8705 PE=1 SV=1
A0A6G5ZVR7_9SAUR **Mass:** 9593 **Score:** 37 **Expect:** 0.28 **Matches:** 4
Disintegrin (Fragment) OS=Vipera anatolica senliki OX=2604287 PE=2 SV=1
A0A1J0CZN0_VIPAA **Mass:** 12765 **Score:** 34 **Expect:** 0.53 **Matches:** 4
Dis-2 OS=Vipera ammodytes ammodytes OX=8705 PE=2 SV=1
DID1B_MACLB **Mass:** 7686 **Score:** 24 **Expect:** 5.1 **Matches:** 3
Disintegrin lebein-1-beta OS=Macrovipera lebetina OX=8709 PE=1 SV=1
DID5B_MACLO **Mass:** 8235 **Score:** 22 **Expect:** 8.9 **Matches:** 2
Disintegrin VLO5B OS=Macrovipera lebetina obtusa OX=209528 PE=1 SV=1
SLB_MACLB **Mass:** 4871 **Score:** 12 **Expect:** 83 **Matches:** 1
Snaclec lebecetin subunit beta (Fragment) OS=Macrovipera lebetina OX=8709 PE=1 SV=1
VKT3_DABRR **Mass:** 9753 **Score:** 10 **Expect:** 1.3e+002 **Matches:** 2
Kunitz-type serine protease inhibitor 3 OS=Daboia russelii OX=8707 PE=2 SV=1
H9BFA3_DABRR **Mass:** 10467 **Score:** 10 **Expect:** 1.3e+002 **Matches:** 2
Kunitz-type protease inhibitor OS=Daboia russelii OX=8707 PE=2 SV=1
SLLC1_MACLB **Mass:** 16969 **Score:** 8 **Expect:** 2.2e+002 **Matches:** 1
Snaclec coagulation factor X-activating enzyme light chain 1 OS=Macrovipera lebetina OX=8709 GN=LC1 PE=1 SV=1
A0A1I9KNR7_VIPAA **Mass:** 14615 **Score:** 8 **Expect:** 2.2e+002 **Matches:** 1
Disintegrin Dis-1 OS=Vipera ammodytes ammodytes OX=8705 PE=2 SV=1
A0A1I9KNR4_VIPAA **Mass:** 14601 **Score:** 8 **Expect:** 2.2e+002 **Matches:** 1
Disintegrin Dis-1 OS=Vipera ammodytes ammodytes OX=8705 PE=2 SV=1
DID2A_MACLB **Mass:** 14638 **Score:** 8 **Expect:** 2.2e+002 **Matches:** 1
Disintegrin lebein-2-alpha OS=Macrovipera lebetina OX=8709 PE=1 SV=1
A0A6B7FPT0_VIPAA **Mass:** 15144 **Score:** 8 **Expect:** 2.3e+002 **Matches:** 1
Endogenous tripeptide metalloproteinase inhibitor MPi-5 OS=Vipera ammodytes ammodytes OX=8705 PE=2 SV=1

---

2. DID1A_MACLB **Mass:** 12718 **Score:** 49 **Expect:** 0.018 **Matches:** 7
Disintegrin lebein-1-alpha OS=Macrovipera lebetina OX=8709 PE=1 SV=2
NTF4_MACLB **Mass:** 4819 **Score:** 12 **Expect:** 91 **Matches:** 1
Neurotrophin-4 (Fragment) OS=Macrovipera lebetina OX=8709 GN=NTF4 PE=3 SV=1

---

3. Q1JRG9_MACLN **Mass:** 7698 **Score:** 38 **Expect:** 0.24 **Matches:** 4
VGD-containing dimeric disintegrin subunit ML-G1 (Fragment) OS=Macrovipera lebetina transmediterranea OX=384075 GN=ml-G1 PE=4 SV=1
Q7ZZQ1_DABSI **Mass:** 16246 **Score:** 9 **Expect:** 1.9e+002 **Matches:** 1
Phospholipase A(2) OS=Daboia siamensis OX=343250 PE=2 SV=1
PA2A1_DABRR **Mass:** 16174 **Score:** 9 **Expect:** 1.9e+002 **Matches:** 1
Acidic phospholipase A2 Drk-a1 OS=Daboia russelii OX=8707 PE=1 SV=1
PA2AA_DABSI **Mass:** 16240 **Score:** 9 **Expect:** 1.9e+002 **Matches:** 1
Acidic phospholipase A2 daboiatoxin A chain OS=Daboia siamensis OX=343250 PE=1 SV=1

---

4. DID4_MACLO **Mass:** 7673 **Score:** 36 **Expect:** 0.37 **Matches:** 6
Disintegrin VLO4 OS=Macrovipera lebetina obtusa OX=209528 PE=1 SV=1

---

5. A0A6B7FRF2_VIPAA **Mass:** 57466 **Score:** 29 **Expect:** 1.7 **Matches:** 6
Amine oxidase OS=Vipera ammodytes ammodytes OX=8705 PE=2 SV=1
B8QCN0_DABRR **Mass:** 13967 **Score:** 11 **Expect:** 1e+002 **Matches:** 1
Prostaglandin E receptor 4 subtype EP4 (Fragment) OS=Daboia russelii OX=8707 GN=PTGER4 PE=4 SV=1
J9YLX0_9SAUR **Mass:** 22376 **Score:** 10 **Expect:** 1.5e+002 **Matches:** 1
NADH-ubiquinone oxidoreductase chain 4 (Fragment) OS=Vipera latastei OX=246179 GN=NAD4 PE=3 SV=1
OXLA_VIPBB **Mass:** 10345 **Score:** 10 **Expect:** 1.6e+002 **Matches:** 1
L-amino-acid oxidase (Fragments) OS=Vipera berus berus OX=31156 PE=1 SV=1
C5H0Z8_DABRR **Mass:** 21841 **Score:** 9 **Expect:** 1.7e+002 **Matches:** 1
Follistatin-like 5 (Fragment) OS=Daboia russelii OX=8707 GN=FSTL5 PE=4 SV=1

---

6. VM25A_MACLO **Mass:** 7570 **Score:** 26 **Expect:** 3.2 **Matches:** 4
Disintegrin VLO5A OS=Macrovipera lebetina obtusa OX=209528 PE=1 SV=1

7. A0A223PK32_DABRR **Mass:** 57907 **Score:** 16 **Expect:** 36 **Matches:** 4
Atrial natriuretic peptide receptor 3 (Fragment) OS=Daboia russelii OX=8707 PE=2 SV=1

8. K4GQE6_DABRR **Mass:** 33004 **Score:** 14 **Expect:** 50 **Matches:** 3
Cartilage intermediate layer protein (Fragment) OS=Daboia russelii OX=8707 GN=CILP PE=4 SV=1

9. DIDB_CERVI **Mass:** 7584 **Score:** 12 **Expect:** 93 **Matches:** 3
Disintegrin CV-11-beta (Fragment) OS=Cerastes vipera OX=8698 PE=2 SV=1
DIDA_CERVI **Mass:** 12717 **Score:** 10 **Expect:** 1.5e+002 **Matches:** 3
Disintegrin CV-11-alpha OS=Cerastes vipera OX=8698 PE=2 SV=1

10. A0A6G5ZUG1_9SAUR **Mass:** 36148 **Score:** 12 **Expect:** 96 **Matches:** 3
Snake venom serine protease OS=Vipera anatolica senliki OX=2604287 PE=2 SV=1
Q6A389_VIPBB **Mass:** 16410 **Score:** 8 **Expect:** 2.5e+002 **Matches:** 1
Ammodytin L(2) variant OS=Vipera berus berus OX=31156 PE=2 SV=1

Search Parameters

```
Type of search         : Peptide Mass Fingerprint
Enzyme                 : Trypsin
Fixed modifications    : Carbamidomethyl (C)
Variable modifications : Oxidation (M)
Mass values            : Monoisotopic
Protein Mass           : Unrestricted
Peptide Mass Tolerance : ± 100 ppm
Peptide Charge State   : 1+
Max Missed Cleavages   : 1
Number of queries      : 85
Selected for scoring   : 27
```

Mascot: http://www.matrixscience.com/

Mascot Search Results

```
User          :
Email         :
Search title  : vipera_all_Lift file:\\Proteomics1\d\data\2105\210505_tliepol_1_68252-68266\68252_68266_lpardo\0_H6\1\0_H6.xml
Database      : vipera  (1414 sequences; 347452 residues)
Timestamp     : 5 May 2021 at 09:46:17 GMT
Protein hits  : DID1A_MACLB Disintegrin lebein-1-alpha OS=Macrovipera lebetina OX=8709 PE=1 SV=2
                DID7A_VIPBB Disintegrin VB7A OS=Vipera berus berus OX=31156 PE=1 SV=1
                VM2A6_VIPAA Disintegrin VA6 OS=Vipera ammodytes ammodytes OX=8705 PE=1 SV=1
                VM25A_MACLO Disintegrin VLO5A OS=Macrovipera lebetina obtusa OX=209528 PE=1 SV=1
```

Mascot Score Histogram

Ions score is -10*Log(P), where P is the probability that the observed match is a random event.
Individual ions scores > 11 indicate identity or extensive homology (p<0.05).
Protein scores are derived from ions scores as a non-probabilistic basis for ranking protein hits.

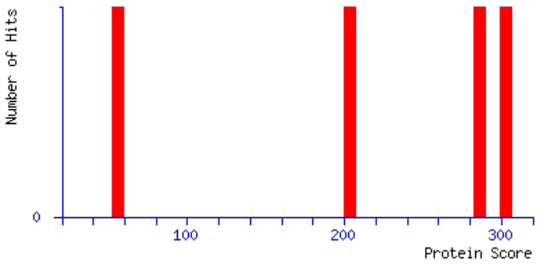

Peptide Summary Report

Format As: Peptide Summary — Help

Significance threshold p< 0.05 | Max. number of hits 10

Standard scoring ○ MudPIT scoring ◉ | Ions score or expect cut-off 0 | Show sub-sets 0

Show pop-ups ◉ Suppress pop-ups ○ | Sort unassigned Decreasing Score | Require bold red ☐

Overview Table

Click on column header to jump to entry in results list.
Move mouse over any indicator to highlight identical peptides.
Click on an indicator to see details of individual match.
Use check boxes to select sub-set of queries for new search.

Mouse over: -Query- -Accession- -Sequence-

Hit:	1	2	3	4
☑ 817.1322 (1+)				
☑ 837.0947 (1+)				
☑ 839.1124 (1+)				
☑ 855.0854 (1+)				
☑ 859.0801 (1+)				
☑ 861.0985 (1+)				
☑ 868.5550 (1+)				
☑ 871.0587 (1+)				
☑ 874.0421 (1+)				
☑ 875.0528 (1+)				
☑ 877.0736 (1+)				
☑ 887.0291 (1+)				
☑ 893.0448 (1+)				
☑ 896.0269 (1+)				
☑ 903.4585 (1+)				
☑ 909.4351 (1+)				
☑ 925.3589 (1+)				
☑ 1022.1395 (1+)				
☑ 1029.5195 (1+)				
☑ 1044.1206 (1+)				
☑ 1055.5381 (1+)				
☑ 1064.0886 (1+)				
☑ 1066.1082 (1+)				
☑ 1074.6014 (1+)				

☑ 1082.0785 (1+)				
☑ 1098.0510 (1+)				
☑ 1108.5873 (1+)				
☑ 1113.7325 (1+)				
☑ 1129.6079 (1+)				
☑ 1146.5988 (1+)				
☑ 1161.6304 (1+)				
☑ 1162.6108 (1+)				
☑ 1163.5712 (1+)				
☑ 1165.6093 (1+)	●			
☑ 1176.6148 (1+)				
☑ 1179.6216 (1+)				
☑ 1187.5867 (1+)				
☑ 1191.6235 (1+)				
☑ 1203.5432 (1+)				
☑ 1207.6186 (1+)				
☑ 1249.1296 (1+)				
☑ 1254.6020 (1+)				
☑ 1271.1102 (1+)				
☑ 1287.0895 (1+)				
☑ 1317.5934 (1+)				
☑ 1318.1391 (1+)				
☑ 1318.5393 (1+)	●	●	●	
☑ 1410.6058 (1+)				
☑ 1455.6296 (1+)				
☑ 1467.6363 (1+)				
☑ 1474.6385 (1+)	●	●	●	
☑ 1476.1205 (1+)				
☑ 1488.6501 (1+)				
☑ 1500.6546 (1+)				
☑ 1608.7985 (1+)				
☑ 1647.7052 (1+)				
☑ 1756.7243 (1+)				
☑ 1761.7746 (1+)		●		●
☑ 1791.7565 (1+)				
☑ 1826.7598 (1+)				
☑ 1872.7755 (1+)				
☑ 1886.7809 (1+)				
☑ 1892.8119 (1+)				
☑ 1908.7852 (1+)				
☑ 1923.7729 (1+)				
☑ 1962.7592 (1+)	●	●	●	
☑ 1975.7640 (1+)				
☑ 1978.7453 (1+)	●	●	●	
☑ 1992.7542 (1+)				
☑ 2016.6822 (1+)				
☑ 2094.7906 (1+)				
☑ 2133.8489 (1+)				
☑ 2149.8328 (1+)				
☑ 2227.0048 (1+)				
☑ 2245.0062 (1+)				
☑ 2265.1728 (1+)				
☑ 2277.1109 (1+)				
☑ 2631.2491 (1+)				
☑ 2647.2327 (1+)				
☑ 2839.3395 (1+)				
☑ 2887.3153 (1+)				
☑ 2914.5357 (1+)				
☑ 3094.6507 (1+)				
☑ 3346.7074 (1+)				
☑ 3350.6517 (1+)				

Select All | Select None | Search Selected | ☐ **Error tolerant** | Archive Report

1. DID1A_MACLB **Mass:** 12718 **Score:** 303 **Matches:** 5(5) **Sequences:** 4(4)
Disintegrin lebein-1-alpha OS=Macrovipera lebetina OX=8709 PE=1 SV=2
☐ Check to include this hit in error tolerant search or archive report

Query Observed Mr(expt) Mr(calc) ppm Miss Score Expect Rank Unique Peptide

Query	Observed	Mr(expt)	Mr(calc)	ppm	Miss	Score	Expect	Rank	Unique	Peptide
**34**	**1165.6093**	**1164.6021**	**1164.5710**	**26.7**	**0**	**74**	**3.9e-008**	**1**	**U**	**K.FLNAGTICNR.A**
**47**	**1318.5393**	**1317.5320**	**1317.5013**	**23.3**	**0**	**22**	**0.0059**	**1**		**R.GEHCVSGPCCR.N**
**51**	**1474.6385**	**1473.6312**	**1473.6024**	**19.5**	**1**	**48**	**1.5e-005**	**1**		**R.RGEHCVSGPCCR.N**
**66**	**1962.7592**	**1961.7520**	**1961.7190**	**16.8**	**0**	**109**	**1.3e-011**	**1**		**R.GDDMNDYCTGISSDCPR.N**
**68**	**1978.7453**	**1977.7381**	**1977.7139**	**12.2**	**0**	**(102)**	**7e-011**	**1**		**R.GDDMNDYCTGISSDCPR.N + Oxidation (M)**

2. DID7A_VIPBB **Mass:** 7574 **Score:** 284 **Matches:** 5(5) **Sequences:** 4(4)
Disintegrin VB7A OS=Vipera berus berus OX=31156 PE=1 SV=1
☐ Check to include this hit in error tolerant search or archive report

Query	Observed	Mr(expt)	Mr(calc)	ppm	Miss	Score	Expect	Rank	Unique	Peptide
47	1318.5393	1317.5320	1317.5013	23.3	0	22	0.0059	1		R.GEHCVSGPCCR.N
51	1474.6385	1473.6312	1473.6024	19.5	1	48	1.5e-005	1		R.RGEHCVSGPCCR.N
**58**	**1761.7746**	**1760.7673**	**1760.7393**	**15.9**	**0**	**55**	**3e-006**	**1**	**U**	**-.NSGNPCCDPVTCKPR.R**
66	1962.7592	1961.7520	1961.7190	16.8	0	109	1.3e-011	1		R.GDDMNDYCTGISSDCPR.N
68	1978.7453	1977.7381	1977.7139	12.2	0	(102)	7e-011	1		R.GDDMNDYCTGISSDCPR.N + Oxidation (M)

3. VM2A6_VIPAA **Mass:** 7588 **Score:** 207 **Matches:** 4(4) **Sequences:** 3(3)
Disintegrin VA6 OS=Vipera ammodytes ammodytes OX=8705 PE=1 SV=1
☐ Check to include this hit in error tolerant search or archive report

Query	Observed	Mr(expt)	Mr(calc)	ppm	Miss	Score	Expect	Rank	Unique	Peptide
47	1318.5393	1317.5320	1317.5013	23.3	0	22	0.0059	1		R.GEHCVSGPCCR.N
51	1474.6385	1473.6312	1473.6024	19.5	1	48	1.5e-005	1		R.RGEHCVSGPCCR.N
66	1962.7592	1961.7520	1961.7190	16.8	0	91	8.9e-010	2	U	R.GDDMNDYCTGVTSDCPR.N
68	1978.7453	1977.7381	1977.7139	12.2	0	(85)	3.3e-009	2	U	R.GDDMNDYCTGVTSDCPR.N + Oxidation (M)

4. VM25A_MACLO **Mass:** 7570 **Score:** 55 **Matches:** 1(1) **Sequences:** 1(1)
Disintegrin VLO5A OS=Macrovipera lebetina obtusa OX=209528 PE=1 SV=1
☐ Check to include this hit in error tolerant search or archive report

Query	Observed	Mr(expt)	Mr(calc)	ppm	Miss	Score	Expect	Rank	Unique	Peptide
58	1761.7746	1760.7673	1760.7029	36.6	0	55	3e-006	1	U	-.NSGNPCCDPVTCQPR.R

Peptide matches not assigned to protein hits: (no details means no match)

	Query	Observed	Mr(expt)	Mr(calc)	ppm	Miss	Score	Expect	Rank	Unique	Peptide
☑	1	*817.1322*	*816.1249*								
☑	2	*837.0947*	*836.0874*								
☑	3	*839.1124*	*838.1052*								
☑	4	*855.0854*	*854.0782*								
☑	5	*859.0801*	*858.0728*								
☑	6	*861.0985*	*860.0912*								
☑	7	*868.5550*	*867.5477*								
☑	8	*871.0587*	*870.0514*								
☑	9	*874.0421*	*873.0348*								
☑	10	*875.0528*	*874.0455*								
☑	11	*877.0736*	*876.0663*								
☑	12	*887.0291*	*886.0218*								
☑	13	*893.0448*	*892.0375*								
☑	14	*896.0269*	*895.0197*								
☑	15	*903.4585*	*902.4512*								
☑	16	*909.4351*	*908.4278*								
☑	17	*925.3589*	*924.3516*								
☑	18	*1022.1395*	*1021.1322*								
☑	19	*1029.5195*	*1028.5122*								
☑	20	*1044.1206*	*1043.1133*								
☑	21	*1055.5381*	*1054.5308*								
☑	22	*1064.0886*	*1063.0814*								
☑	23	*1066.1082*	*1065.1009*								
☑	24	*1074.6014*	*1073.5941*								
☑	25	*1082.0785*	*1081.0712*								
☑	26	*1098.0510*	*1097.0437*								
☑	27	*1108.5873*	*1107.5800*								
☑	28	*1113.7325*	*1112.7252*								
☑	29	*1129.6079*	*1128.6007*								
☑	30	*1146.5988*	*1145.5916*								
☑	31	*1161.6304*	*1160.6231*								
☑	32	*1162.6108*	*1161.6035*								
☑	33	*1163.5712*	*1162.5639*								
☑	35	*1176.6148*	*1175.6076*								
☑	36	*1179.6216*	*1178.6143*								
☑	37	*1187.5867*	*1186.5794*								
☑	38	*1191.6235*	*1190.6162*								
☑	39	*1203.5432*	*1202.5360*								
☑	40	*1207.6186*	*1206.6113*								

☑	41	*1249.1296*	*1248.1223*
☑	42	*1254.6020*	*1253.5947*
☑	43	*1271.1102*	*1270.1029*
☑	44	*1287.0895*	*1286.0822*
☑	45	*1317.5934*	*1316.5861*
☑	46	*1318.1391*	*1317.1318*
☑	48	*1410.6058*	*1409.5986*
☑	49	*1455.6296*	*1454.6223*
☑	50	*1467.6363*	*1466.6290*
☑	52	*1476.1205*	*1475.1132*
☑	53	*1488.6501*	*1487.6429*
☑	54	*1500.6546*	*1499.6474*
☑	55	*1608.7985*	*1607.7912*
☑	56	*1647.7052*	*1646.6980*
☑	57	*1756.7243*	*1755.7170*
☑	59	*1791.7565*	*1790.7493*
☑	60	*1826.7598*	*1825.7525*
☑	61	*1872.7755*	*1871.7682*
☑	62	*1886.7809*	*1885.7737*
☑	63	*1892.8119*	*1891.8047*
☑	64	*1908.7852*	*1907.7779*
☑	65	*1923.7729*	*1922.7656*
☑	67	*1975.7640*	*1974.7567*
☑	69	*1992.7542*	*1991.7470*
☑	70	*2016.6822*	*2015.6749*
☑	71	*2094.7906*	*2093.7833*
☑	72	*2133.8489*	*2132.8416*
☑	73	*2149.8328*	*2148.8255*
☑	74	*2227.0048*	*2225.9976*
☑	75	*2245.0062*	*2243.9989*
☑	76	*2265.1728*	*2264.1655*
☑	77	*2277.1109*	*2276.1036*
☑	78	*2631.2491*	*2630.2419*
☑	79	*2647.2327*	*2646.2254*
☑	80	*2839.3395*	*2838.3322*
☑	81	*2887.3153*	*2886.3080*
☑	82	*2914.5357*	*2913.5284*
☑	83	*3094.6507*	*3093.6434*
☑	84	*3346.7074*	*3345.7001*
☑	85	*3350.6517*	*3349.6444*

Search Parameters

Type of search : MS/MS Ion Search
Enzyme : Trypsin
Fixed modifications : Carbamidomethyl (C)
Variable modifications : Oxidation (M)
Mass values : Monoisotopic
Protein Mass : Unrestricted
Peptide Mass Tolerance : ± 100 ppm
Fragment Mass Tolerance: ± 0.7 Da
Max Missed Cleavages : 1
Instrument type : MALDI-TOF-TOF
Number of queries : 85

Mascot: http://www.matrixscience.com/

Mascot Search Results

```
User          :
Email         :
Search title  : vipera_all_Lift file:\\Proteomics1\d\data\2105\210505_tliepol_1_68252-68266\68252_68266_1pardo\0_H6\1\0_H6.xml
Database      : vipera  (1414 sequences; 347452 residues)
Timestamp     : 5 May 2021 at 09:46:17 GMT
Warning       : A Peptide summary report will usually give a much clearer picture of MS/MS search results.
Top Score     : 295 for DID1A_MACLB, Disintegrin lebein-1-alpha OS=Macrovipera lebetina OX=8709 PE=1 SV=2
```

Mascot Score Histogram

Protein score is -10*Log(P), where P is the probability that the observed match is a random event.
Protein scores greater than 44 are significant (p<0.05).
Protein scores are derived from ions scores as a non-probabilistic basis for ranking protein hits.

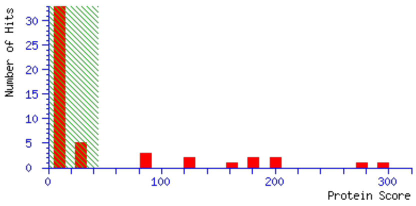

Protein Summary Report

Format As | Protein Summary (deprecated) | Help

Significance threshold p< 0.05 Max. number of hits 20

Overview Table

Click on column header to jump to entry in results list.
Move mouse over any indicator to highlight identical peptides.
Click on an indicator to see details of individual match.
Use check boxes to select sub-set of queries for new search.

Mouse over: -Query- -Accession- -Sequence-

Hit:	1	2	3	4	5	6	7	8	9	10	11	12	13	14	15	16	17	18	19	20
☑ 817.1322 (1+)																				
☑ 837.0947 (1+)																				
☑ 839.1124 (1+)																				
☑ 855.0854 (1+)																				
☑ 859.0801 (1+)																				
☑ 861.0985 (1+)																				
☑ 868.5550 (1+)																				
☑ 871.0587 (1+)																				
☑ 874.0421 (1+)																				
☑ 875.0528 (1+)																				
☑ 877.0736 (1+)																				
☑ 887.0291 (1+)																				
☑ 893.0448 (1+)																				
☑ 896.0269 (1+)																				
☑ 903.4585 (1+)																				
☑ 909.4351 (1+)																				
☑ 925.3589 (1+)																				
☑ 1022.1395 (1+)																				
☑ 1029.5195 (1+)																				
☑ 1044.1206 (1+)																				
☑ 1055.5381 (1+)																				
☑ 1064.0886 (1+)																				
☑ 1066.1082 (1+)																				
☑ 1074.6014 (1+)																				
☑ 1082.0785 (1+)																				
☑ 1098.0510 (1+)																				
☑ 1108.5873 (1+)																				
☑ 1113.7325 (1+)																				
☑ 1129.6079 (1+)																				
☑ 1146.5988 (1+)																				
☑ 1161.6304 (1+)																				
☑ 1162.6108 (1+)																				
☑ 1163.5712 (1+)																				
☑ 1165.6093 (1+)	●																			
☑ 1176.6148 (1+)																				
☑ 1179.6216 (1+)																				
☑ 1187.5867 (1+)																				
☑ 1191.6235 (1+)																				
☑ 1203.5432 (1+)																				
☑ 1207.6186 (1+)																				
☑ 1249.1296 (1+)																				
☑ 1254.6020 (1+)																				
☑ 1271.1102 (1+)																				
☑ 1287.0895 (1+)																				
☑ 1317.5934 (1+)																				
☑ 1318.1391 (1+)																				
☑ 1318.5393 (1+)	●	●	●	●	●		●			●	●		●	●	●					
☑ 1410.6058 (1+)																				
☑ 1455.6296 (1+)																				

Query	1	2	3	4	5	6	7	8	9	10	11	12	13	14	15	16	17	18	19	20
☑ 1467.6363 (1+)																				
☑ 1474.6385 (1+)	●	●	●	●	●		●			●	●									
☑ 1476.1205 (1+)																				
☑ 1488.6501 (1+)																				
☑ 1500.6546 (1+)																				
☑ 1608.7985 (1+)																				
☑ 1647.7052 (1+)																				
☑ 1756.7243 (1+)																				
☑ 1761.7746 (1+)		●				●	●					●								
☑ 1791.7565 (1+)																				
☑ 1826.7598 (1+)																				
☑ 1872.7755 (1+)																				
☑ 1886.7809 (1+)																				
☑ 1892.8119 (1+)																				
☑ 1908.7852 (1+)																				
☑ 1923.7729 (1+)																				
☑ 1962.7592 (1+)	●	●	●	●	●	●		●	●											
☑ 1975.7640 (1+)																				
☑ 1978.7453 (1+)	●	●	●	●	●	●		●	●											
☑ 1992.7542 (1+)																				
☑ 2016.6822 (1+)																				
☑ 2094.7906 (1+)																				
☑ 2133.8489 (1+)																				
☑ 2149.8328 (1+)																				
☑ 2227.0048 (1+)																				
☑ 2245.0062 (1+)																				
☑ 2265.1728 (1+)																				
☑ 2277.1109 (1+)																				
☑ 2631.2491 (1+)																				
☑ 2647.2327 (1+)																				
☑ 2839.3395 (1+)																				
☑ 2887.3153 (1+)																				
☑ 2914.5357 (1+)																				
☑ 3094.6507 (1+)																				
☑ 3346.7074 (1+)																				
☑ 3350.6517 (1+)																				

Select All   Select None   Search Selected

Index

	Accession	Mass	Score	Description
1.	DID1A_MACLB	12718	295	Disintegrin lebein-1-alpha OS=Macrovipera lebetina OX=8709 PE=1 SV=2
2.	DID7A_VIPBB	7574	272	Disintegrin VB7A OS=Vipera berus berus OX=31156 PE=1 SV=1
3.	A0A6G5ZVR7_9SAUR	9593	202	Disintegrin (Fragment) OS=Vipera anatolica senliki OX=2604287 PE=2 SV=1
4.	A0A1J0CZN0_VIPAA	12765	199	Dis-2 OS=Vipera ammodytes ammodytes OX=8705 PE=2 SV=1
5.	VM2A6_VIPAA	7588	186	Disintegrin VA6 OS=Vipera ammodytes ammodytes OX=8705 PE=1 SV=1
6.	DID1B_MACLB	7686	181	Disintegrin lebein-1-beta OS=Macrovipera lebetina OX=8709 PE=1 SV=1
7.	DID4_MACLO	7673	169	Disintegrin VLO4 OS=Macrovipera lebetina obtusa OX=209528 PE=1 SV=1
8.	DIDB_CERVI	7584	125	Disintegrin CV-11-beta (Fragment) OS=Cerastes vipera OX=8698 PE=2 SV=1
9.	DIDA_CERVI	12717	121	Disintegrin CV-11-alpha OS=Cerastes vipera OX=8698 PE=2 SV=1
10.	Q1JRG9_MACLN	7698	94	VGD-containing dimeric disintegrin subunit ML-G1 (Fragment) OS=Macrovipera lebetina transmediterranea OX=384075 GN=ml-G1 PE=4 SV=1
11.	DID5B_MACLO	8235	85	Disintegrin VLO5B OS=Macrovipera lebetina obtusa OX=209528 PE=1 SV=1
12.	VM25A_MACLO	7570	81	Disintegrin VLO5A OS=Macrovipera lebetina obtusa OX=209528 PE=1 SV=1
13.	A0A1I9KNR7_VIPAA	14615	27	Disintegrin Dis-1 OS=Vipera ammodytes ammodytes OX=8705 PE=2 SV=1
14.	A0A1I9KNR4_VIPAA	14601	27	Disintegrin Dis-1 OS=Vipera ammodytes ammodytes OX=8705 PE=2 SV=1
15.	DID2A_MACLB	14638	27	Disintegrin lebein-2-alpha OS=Macrovipera lebetina OX=8709 PE=1 SV=1
16.	A0A6B7FRF2_VIPAA	57466	25	Amine oxidase OS=Vipera ammodytes ammodytes OX=8705 PE=2 SV=1
17.	OXLA_MACLB	12541	21	L-amino-acid oxidase (Fragments) OS=Macrovipera lebetina OX=8709 PE=1 SV=2
18.	A0A6G5ZUD1_9SAUR	30200	19	Lysosomal acid lipase/cholesteryl ester hydrolase 2 (Fragment) OS=Vipera anatolica senliki OX=2604287 PE=2 SV=1
19.	DIS_MACLO	4854	18	Disintegrin obtustatin OS=Macrovipera lebetina obtusa OX=209528 PE=1 SV=1
20.	M4T472_9PERO	26404	17	Ring finger protein 213 (Fragment) OS=Echiichthys vipera OX=94984 PE=4 SV=1

Results List

1. DID1A_MACLB **Mass:** 12718 **Score:** 295 **Expect:** 4.5e-027 **Matches:** 7

Disintegrin lebein-1-alpha OS=Macrovipera lebetina OX=8709 PE=1 SV=2

Observed	Mr(expt)	Mr(calc)	ppm	Start	End	Miss	Ions	Peptide
1165.6093	1164.6021	1164.5710	26.7	78 -	87	0	74	K.FLNAGTICNR.A
1318.5393	1317.5320	1317.5013	23.3	64 -	74	0	22	R.GEHCVSGPCCR.N
1474.6385	1473.6312	1473.6024	19.5	63 -	74	1	48	R.RGEHCVSGPCCR.N
1962.7592	1961.7520	1961.7190	16.8	90 -	106	0	109	R.GDDMNDYCTGISSDCPR.N
1978.7453	1977.7381	1977.7139	12.2	90 -	106	0	(102)	R.GDDMNDYCTGISSDCPR.N + Oxidation (M)
2631.2491	2630.2419	2630.2074	13.1	39 -	62	0	---	K.VTVLPTGAMNSGNPCCDPVTCKPR.R
2647.2327	2646.2254	2646.2023	8.75	39 -	62	0	---	K.VTVLPTGAMNSGNPCCDPVTCKPR.R + Oxidation (M)

No match to: 817.1322, 837.0947, 839.1124, 855.0854, 859.0801, 861.0985, 868.5550, 871.0587, 874.0421, 875.0528, 877.0736, 887.0291, 893.0448, 896.0269, 903.4585, 909.4351, 925.3589, 1022.1395, 1029.5195, 1044.1206, 1055.5381, 1064.0886, 1066.1082, 1074.6014, 1082.0785, 1098.0510, 1108.5873, 1113.7325, 1129.6079, 1146.5988, 1161.6304, 1162.6108, 1163.5712, 1176.6148, 1179.6216, 1187.5867, 1191.6235, 1203.5432, 1207.6186, 1249.1296, 1254.6020, 1271.1102, 1287.0895, 1317.5934, 1318.1391, 1410.6058, 1455.6296, 1467.6363, 1476.1205, 1488.6501, 1500.6546, 1608.7985, 1647.7052, 1756.7243, 1761.7746, 1791.7565, 1826.7598, 1872.7755, 1886.7809, 1892.8119, 1908.7852, 1923.7729, 1975.7640, 1992.7542, 2016.6822, 2094.7906, 2133.8489, 2149.8328, 2227.0048, 2245.0062, 2265.1728, 2277.1109, 2839.3395, 2887.3153, 2914.5357, 3094.6507, 3346.7074, 3350.6517

2. DID7A_VIPBB **Mass:** 7574 **Score:** 272 **Expect:** 8.9e-025 **Matches:** 5

Disintegrin VB7A OS=Vipera berus berus OX=31156 PE=1 SV=1

Observed	Mr(expt)	Mr(calc)	ppm	Start	End	Miss	Ions	Peptide
1318.5393	1317.5320	1317.5013	23.3	17 -	27	0	22	R.GEHCVSGPCCR.N
1474.6385	1473.6312	1473.6024	19.5	16 -	27	1	48	R.RGEHCVSGPCCR.N
1761.7746	1760.7673	1760.7393	15.9	1 -	15	0	55	-.NSGNPCCDPVTCKPR.R
1962.7592	1961.7520	1961.7190	16.8	43 -	59	0	109	R.GDDMNDYCTGISSDCPR.N
1978.7453	1977.7381	1977.7139	12.2	43 -	59	0	(102)	R.GDDMNDYCTGISSDCPR.N + Oxidation (M)

No match to: 817.1322, 837.0947, 839.1124, 855.0854, 859.0801, 861.0985, 868.5550, 871.0587, 874.0421, 875.0528, 877.0736, 887.0291, 893.0448, 896.0269, 903.4585, 909.4351, 925.3589, 1022.1395, 1029.5195, 1044.1206, 1055.5381, 1064.0886, 1066.1082, 1074.6014, 1082.0785, 1098.0510, 1108.5873, 1113.7325, 1129.6079, 1146.5988, 1161.6304, 1162.6108, 1163.5712, 1165.6093, 1176.6148, 1179.6216, 1187.5867, 1191.6235, 1203.5432, 1207.6186, 1249.1296, 1254.6020, 1271.1102, 1287.0895, 1317.5934, 1318.1391, 1410.6058, 1455.6296, 1467.6363, 1476.1205, 1488.6501, 1500.6546, 1608.7985, 1647.7052, 1756.7243, 1791.7565, 1826.7598, 1872.7755, 1886.7809, 1892.8119, 1908.7852, 1923.7729, 1975.7640, 1992.7542, 2016.6822, 2094.7906, 2133.8489, 2149.8328, 2227.0048, 2245.0062, 2265.1728, 2277.1109, 2631.2491, 2647.2327, 2839.3395, 2887.3153, 2914.5357, 3094.6507, 3346.7074, 3350.6517

3. A0A6G5ZVR7_9SAUR **Mass:** 9593 **Score:** 202 **Expect:** 8.9e-018 **Matches:** 4

Disintegrin (Fragment) OS=Vipera anatolica senliki OX=2604287 PE=2 SV=1

Observed	Mr(expt)	Mr(calc)	ppm	Start	End	Miss	Ions	Peptide
1318.5393	1317.5320	1317.5013	23.3	35 -	45	0	22	R.GEHCVSGPCCR.N
1474.6385	1473.6312	1473.6024	19.5	34 -	45	1	48	R.RGEHCVSGPCCR.N
1962.7592	1961.7520	1961.7190	16.8	61 -	77	0	109	R.GDDMNDYCTGISSDCPR.N

1978.7453	1977.7381	1977.7139	12.2	61 -	77	0	(102)	R.GDDMNDYCTGISSDCPR.N + Oxidation (M)

No match to: 817.1322, 837.0947, 839.1124, 855.0854, 859.0801, 861.0985, 868.5550, 871.0587, 874.0421, 875.0528, 877.0736, 887.0291, 893.0448, 896.0269, 903.4585, 909.4351, 925.3589, 1022.1395, 1029.5195, 1044.1206, 1055.5381, 1064.0886, 1066.1082, 1074.6014, 1082.0785, 1098.0510, 1108.5873, 1113.7325, 1129.6079, 1146.5988, 1161.6304, 1162.6108, 1163.5712, 1165.6093, 1176.6148, 1179.6216, 1187.5867, 1191.6235, 1203.5432, 1207.6186, 1249.1296, 1254.6020, 1271.1102, 1287.0895, 1317.5934, 1318.1391, 1410.6058, 1455.6296, 1467.6363, 1476.1205, 1488.6501, 1500.6546, 1608.7985, 1647.7052, 1756.7243, 1761.7746, 1791.7565, 1826.7598, 1872.7755, 1886.7809, 1892.8119, 1908.7852, 1923.7729, 1975.7640, 1992.7542, 2016.6822, 2094.7906, 2133.8489, 2149.8328, 2227.0048, 2245.0062, 2265.1728, 2277.1109, 2631.2491, 2647.2327, 2839.3395, 2887.3153, 2914.5357, 3094.6507, 3346.7074, 3350.6517

4. A0A1J0CZN0_VIPAA **Mass:** 12765 **Score:** 199 **Expect:** 1.8e-017 **Matches:** 4
Dis-2 OS=Vipera ammodytes ammodytes OX=8705 PE=2 SV=1

Observed	Mr(expt)	Mr(calc)	ppm	Start	End	Miss	Ions	Peptide
1318.5393	1317.5320	1317.5013	23.3	64 -	74	0	22	R.GEHCVSGPCCR.N
1474.6385	1473.6312	1473.6024	19.5	63 -	74	1	48	R.RGEHCVSGPCCR.N
1962.7592	1961.7520	1961.7190	16.8	90 -	106	0	109	R.GDDMNDYCTGISSDCPR.N
1978.7453	1977.7381	1977.7139	12.2	90 -	106	0	(102)	R.GDDMNDYCTGISSDCPR.N + Oxidation (M)

No match to: 817.1322, 837.0947, 839.1124, 855.0854, 859.0801, 861.0985, 868.5550, 871.0587, 874.0421, 875.0528, 877.0736, 887.0291, 893.0448, 896.0269, 903.4585, 909.4351, 925.3589, 1022.1395, 1029.5195, 1044.1206, 1055.5381, 1064.0886, 1066.1082, 1074.6014, 1082.0785, 1098.0510, 1108.5873, 1113.7325, 1129.6079, 1146.5988, 1161.6304, 1162.6108, 1163.5712, 1165.6093, 1176.6148, 1179.6216, 1187.5867, 1191.6235, 1203.5432, 1207.6186, 1249.1296, 1254.6020, 1271.1102, 1287.0895, 1317.5934, 1318.1391, 1410.6058, 1455.6296, 1467.6363, 1476.1205, 1488.6501, 1500.6546, 1608.7985, 1647.7052, 1756.7243, 1761.7746, 1791.7565, 1826.7598, 1872.7755, 1886.7809, 1892.8119, 1908.7852, 1923.7729, 1975.7640, 1992.7542, 2016.6822, 2094.7906, 2133.8489, 2149.8328, 2227.0048, 2245.0062, 2265.1728, 2277.1109, 2631.2491, 2647.2327, 2839.3395, 2887.3153, 2914.5357, 3094.6507, 3346.7074, 3350.6517

5. VM2A6_VIPAA **Mass:** 7588 **Score:** 186 **Expect:** 3.6e-016 **Matches:** 4
Disintegrin VA6 OS=Vipera ammodytes ammodytes OX=8705 PE=1 SV=1

Observed	Mr(expt)	Mr(calc)	ppm	Start	End	Miss	Ions	Peptide
1318.5393	1317.5320	1317.5013	23.3	17 -	27	0	22	R.GEHCVSGPCCR.N
1474.6385	1473.6312	1473.6024	19.5	16 -	27	1	48	R.RGEHCVSGPCCR.N
1962.7592	1961.7520	1961.7190	16.8	43 -	59	0	91	R.GDDMNDYCTGVTSDCPR.N
1978.7453	1977.7381	1977.7139	12.2	43 -	59	0	(85)	R.GDDMNDYCTGVTSDCPR.N + Oxidation (M)

No match to: 817.1322, 837.0947, 839.1124, 855.0854, 859.0801, 861.0985, 868.5550, 871.0587, 874.0421, 875.0528, 877.0736, 887.0291, 893.0448, 896.0269, 903.4585, 909.4351, 925.3589, 1022.1395, 1029.5195, 1044.1206, 1055.5381, 1064.0886, 1066.1082, 1074.6014, 1082.0785, 1098.0510, 1108.5873, 1113.7325, 1129.6079, 1146.5988, 1161.6304, 1162.6108, 1163.5712, 1165.6093, 1176.6148, 1179.6216, 1187.5867, 1191.6235, 1203.5432, 1207.6186, 1249.1296, 1254.6020, 1271.1102, 1287.0895, 1317.5934, 1318.1391, 1410.6058, 1455.6296, 1467.6363, 1476.1205, 1488.6501, 1500.6546, 1608.7985, 1647.7052, 1756.7243, 1761.7746, 1791.7565, 1826.7598, 1872.7755, 1886.7809, 1892.8119, 1908.7852, 1923.7729, 1975.7640, 1992.7542, 2016.6822, 2094.7906, 2133.8489, 2149.8328, 2227.0048, 2245.0062, 2265.1728, 2277.1109, 2631.2491, 2647.2327, 2839.3395, 2887.3153, 2914.5357, 3094.6507, 3346.7074, 3350.6517

6. DID1B_MACLB **Mass:** 7686 **Score:** 181 **Expect:** 1.1e-015 **Matches:** 3
Disintegrin lebein-1-beta OS=Macrovipera lebetina OX=8709 PE=1 SV=1

Observed	Mr(expt)	Mr(calc)	ppm	Start	End	Miss	Ions	Peptide
1761.7746	1760.7673	1760.7393	15.9	1 -	15	0	55	-.NSGNPCCDPVTCKPR.R
1962.7592	1961.7520	1961.7190	16.8	43 -	59	0	109	R.GDDMNDYCTGISSDCPR.N
1978.7453	1977.7381	1977.7139	12.2	43 -	59	0	(102)	R.GDDMNDYCTGISSDCPR.N + Oxidation (M)

No match to: 817.1322, 837.0947, 839.1124, 855.0854, 859.0801, 861.0985, 868.5550, 871.0587, 874.0421, 875.0528, 877.0736, 887.0291, 893.0448, 896.0269, 903.4585, 909.4351, 925.3589, 1022.1395, 1029.5195, 1044.1206, 1055.5381, 1064.0886, 1066.1082, 1074.6014, 1082.0785, 1098.0510, 1108.5873, 1113.7325, 1129.6079, 1146.5988, 1161.6304, 1162.6108, 1163.5712, 1165.6093, 1176.6148, 1179.6216, 1187.5867, 1191.6235, 1203.5432, 1207.6186, 1249.1296, 1254.6020, 1271.1102, 1287.0895, 1317.5934, 1318.1391, 1318.5393, 1410.6058, 1455.6296, 1467.6363, 1474.6385, 1476.1205, 1488.6501, 1500.6546, 1608.7985, 1647.7052, 1756.7243, 1791.7565, 1826.7598, 1872.7755, 1886.7809, 1892.8119, 1908.7852, 1923.7729, 1975.7640, 1992.7542, 2016.6822, 2094.7906, 2133.8489, 2149.8328, 2227.0048, 2245.0062, 2265.1728, 2277.1109, 2631.2491, 2647.2327, 2839.3395, 2887.3153, 2914.5357, 3094.6507, 3346.7074, 3350.6517

7. DID4_MACLO **Mass:** 7673 **Score:** 169 **Expect:** 1.8e-014 **Matches:** 6
Disintegrin VLO4 OS=Macrovipera lebetina obtusa OX=209528 PE=1 SV=1

Observed	Mr(expt)	Mr(calc)	ppm	Start	End	Miss	Ions	Peptide
1179.6216	1178.6143	1178.6230	-7.37	32 -	41	1	---	K.FLNAGTICKR.A
1318.5393	1317.5320	1317.5013	23.3	18 -	28	0	22	R.GEHCVSGPCCR.N
1474.6385	1473.6312	1473.6024	19.5	17 -	28	1	48	R.RGEHCVSGPCCR.N
1761.7746	1760.7673	1760.7393	15.9	2 -	16	0	55	M.NSGNPCCDPVTCKPR.R
1892.8119	1891.8047	1891.7798	13.2	1 -	16	0	---	-.MNSGNPCCDPVTCKPR.R
1908.7852	1907.7779	1907.7747	1.69	1 -	16	0	---	-.MNSGNPCCDPVTCKPR.R + Oxidation (M)

No match to: 817.1322, 837.0947, 839.1124, 855.0854, 859.0801, 861.0985, 868.5550, 871.0587, 874.0421, 875.0528, 877.0736, 887.0291, 893.0448, 896.0269, 903.4585, 909.4351, 925.3589, 1022.1395, 1029.5195, 1044.1206, 1055.5381, 1064.0886, 1066.1082, 1074.6014, 1082.0785, 1098.0510, 1108.5873, 1113.7325, 1129.6079, 1146.5988, 1161.6304, 1162.6108, 1163.5712, 1165.6093, 1176.6148, 1187.5867, 1191.6235, 1203.5432, 1207.6186, 1249.1296, 1254.6020, 1271.1102, 1287.0895, 1317.5934, 1318.1391, 1410.6058, 1455.6296, 1467.6363, 1476.1205, 1488.6501, 1500.6546, 1608.7985, 1647.7052, 1756.7243, 1791.7565, 1826.7598, 1872.7755, 1886.7809, 1923.7729, 1962.7592, 1975.7640, 1978.7453, 1992.7542, 2016.6822, 2094.7906, 2133.8489, 2149.8328, 2227.0048, 2245.0062, 2265.1728, 2277.1109, 2631.2491, 2647.2327, 2839.3395, 2887.3153, 2914.5357, 3094.6507, 3346.7074, 3350.6517

8. DIDB_CERVI **Mass:** 7584 **Score:** 125 **Expect:** 4.5e-010 **Matches:** 3
Disintegrin CV-11-beta (Fragment) OS=Cerastes vipera OX=8698 PE=2 SV=1

Observed	Mr(expt)	Mr(calc)	ppm	Start	End	Miss	Ions	Peptide
1488.6501	1487.6429	1487.6180	16.7	16 -	27	1	---	K.RGEHCISGPCCR.N
1962.7592	1961.7520	1961.7190	16.8	43 -	59	0	109	R.GDDMNDYCTGISSDCPR.N
1978.7453	1977.7381	1977.7139	12.2	43 -	59	0	(102)	R.GDDMNDYCTGISSDCPR.N + Oxidation (M)

No match to: 817.1322, 837.0947, 839.1124, 855.0854, 859.0801, 861.0985, 868.5550, 871.0587, 874.0421, 875.0528, 877.0736, 887.0291, 893.0448, 896.0269, 903.4585, 909.4351, 925.3589, 1022.1395, 1029.5195, 1044.1206, 1055.5381, 1064.0886, 1066.1082, 1074.6014, 1082.0785, 1098.0510, 1108.5873, 1113.7325, 1129.6079, 1146.5988, 1161.6304, 1162.6108, 1163.5712, 1165.6093, 1176.6148, 1179.6216, 1187.5867, 1191.6235, 1203.5432, 1207.6186, 1249.1296, 1254.6020, 1271.1102, 1287.0895, 1317.5934, 1318.1391, 1318.5393, 1410.6058, 1455.6296, 1467.6363, 1474.6385, 1476.1205, 1500.6546, 1608.7985, 1647.7052, 1756.7243, 1761.7746, 1791.7565, 1826.7598, 1872.7755, 1886.7809, 1892.8119, 1908.7852, 1923.7729, 1975.7640, 1992.7542, 2016.6822, 2094.7906, 2133.8489, 2149.8328, 2227.0048, 2245.0062, 2265.1728, 2277.1109, 2631.2491, 2647.2327, 2839.3395, 2887.3153, 2914.5357, 3094.6507, 3346.7074, 3350.6517

9. DIDA_CERVI **Mass:** 12717 **Score:** 121 **Expect:** 1.1e-009 **Matches:** 3
Disintegrin CV-11-alpha OS=Cerastes vipera OX=8698 PE=2 SV=1

Observed	Mr(expt)	Mr(calc)	ppm	Start	End	Miss	Ions	Peptide
1488.6501	1487.6429	1487.6180	16.7	63 -	74	1	---	K.RGEHCISGPCCR.N
1962.7592	1961.7520	1961.7190	16.8	90 -	106	0	109	K.GDDMNDYCTGISSDCPR.N
1978.7453	1977.7381	1977.7139	12.2	90 -	106	0	(102)	K.GDDMNDYCTGISSDCPR.N + Oxidation (M)

No match to: 817.1322, 837.0947, 839.1124, 855.0854, 859.0801, 861.0985, 868.5550, 871.0587, 874.0421, 875.0528, 877.0736, 887.0291, 893.0448, 896.0269, 903.4585, 909.4351, 925.3589, 1022.1395, 1029.5195, 1044.1206, 1055.5381, 1064.0886, 1066.1082, 1074.6014, 1082.0785, 1098.0510, 1108.5873, 1113.7325, 1129.6079, 1146.5988, 1161.6304, 1162.6108, 1163.5712, 1165.6093, 1176.6148, 1179.6216, 1187.5867, 1191.6235, 1203.5432, 1207.6186, 1249.1296, 1254.6020, 1271.1102, 1287.0895, 1317.5934, 1318.1391, 1318.5393, 1410.6058, 1455.6296, 1467.6363, 1474.6385, 1476.1205, 1500.6546, 1608.7985, 1647.7052, 1756.7243, 1761.7746, 1791.7565, 1826.7598, 1872.7755, 1886.7809, 1892.8119, 1908.7852, 1923.7729, 1975.7640, 1992.7542, 2016.6822, 2094.7906, 2133.8489, 2149.8328, 2227.0048, 2245.0062, 2265.1728, 2277.1109, 2631.2491, 2647.2327, 2839.3395, 2887.3153, 2914.5357, 3094.6507, 3346.7074, 3350.6517

10. Q1JRG9_MACLN **Mass:** 7698 **Score:** 94 **Expect:** 6.2e-007 **Matches:** 4
VGD-containing dimeric disintegrin subunit ML-G1 (Fragment) OS=Macrovipera lebetina transmediterranea OX=384075 GN=ml-G1 PE=4 SV=1

Observed	Mr(expt)	Mr(calc)	ppm	Start	End	Miss	Ions	Peptide
1318.5393	1317.5320	1317.5013	23.3	18 -	28	0	22	R.GEHCVSGPCCR.N
1474.6385	1473.6312	1473.6024	19.5	17 -	28	1	48	R.RGEHCVSGPCCR.N
2133.8489	2132.8416	2132.8085	15.5	42 -	60	0	---	R.AVGDDMDDYCTGISSDCPR.N
2149.8328	2148.8255	2148.8035	10.3	42 -	60	0	---	R.AVGDDMDDYCTGISSDCPR.N + Oxidation (M)

No match to: 817.1322, 837.0947, 839.1124, 855.0854, 859.0801, 861.0985, 868.5550, 871.0587, 874.0421, 875.0528, 877.0736, 887.0291, 893.0448, 896.0269, 903.4585, 909.4351, 925.3589, 1022.1395, 1029.5195, 1044.1206, 1055.5381, 1064.0886, 1066.1082, 1074.6014, 1082.0785, 1098.0510, 1108.5873, 1113.7325, 1129.6079, 1146.5988, 1161.6304, 1162.6108, 1163.5712, 1165.6093, 1176.6148, 1179.6216, 1187.5867, 1191.6235, 1203.5432, 1207.6186, 1249.1296, 1254.6020, 1271.1102, 1287.0895, 1317.5934, 1318.1391, 1410.6058, 1455.6296, 1467.6363, 1476.1205, 1488.6501, 1500.6546, 1608.7985, 1647.7052, 1756.7243, 1761.7746, 1791.7565, 1826.7598, 1872.7755, 1886.7809, 1892.8119, 1908.7852, 1923.7729, 1962.7592, 1975.7640, 1978.7453, 1992.7542, 2016.6822, 2094.7906, 2227.0048, 2245.0062, 2265.1728, 2277.1109, 2631.2491, 2647.2327, 2839.3395, 2887.3153, 2914.5357, 3094.6507, 3346.7074, 3350.6517

11. DID5B_MACLO **Mass:** 8235 **Score:** 85 **Expect:** 4.8e-006 **Matches:** 2
Disintegrin VLO5B OS=Macrovipera lebetina obtusa OX=209528 PE=1 SV=1

Observed	Mr(expt)	Mr(calc)	ppm	Start	End	Miss	Ions	Peptide
1318.5393	1317.5320	1317.5013	23.3	18 -	28	0	22	R.GEHCVSGPCCR.N
1474.6385	1473.6312	1473.6024	19.5	17 -	28	1	48	R.RGEHCVSGPCCR.N

No match to: 817.1322, 837.0947, 839.1124, 855.0854, 859.0801, 861.0985, 868.5550, 871.0587, 874.0421, 875.0528, 877.0736, 887.0291, 893.0448, 896.0269, 903.4585, 909.4351, 925.3589, 1022.1395, 1029.5195, 1044.1206, 1055.5381, 1064.0886, 1066.1082, 1074.6014, 1082.0785, 1098.0510, 1108.5873, 1113.7325, 1129.6079, 1146.5988, 1161.6304, 1162.6108, 1163.5712, 1165.6093, 1176.6148, 1179.6216, 1187.5867, 1191.6235, 1203.5432, 1207.6186, 1249.1296, 1254.6020, 1271.1102, 1287.0895, 1317.5934, 1318.1391, 1410.6058, 1455.6296, 1467.6363, 1476.1205, 1488.6501, 1500.6546, 1608.7985, 1647.7052, 1756.7243, 1761.7746, 1791.7565, 1826.7598, 1872.7755, 1886.7809, 1892.8119, 1908.7852, 1923.7729, 1962.7592, 1975.7640, 1978.7453, 1992.7542, 2016.6822, 2094.7906, 2133.8489,

2149.8328, 2227.0048, 2245.0062, 2265.1728, 2277.1109, 2631.2491, 2647.2327, 2839.3395, 2887.3153, 2914.5357, 3094.6507, 3346.7074, 3350.6517

12. VM25A_MACLO **Mass:** 7570 **Score:** 81 **Expect:** 1.2e-005 **Matches:** 4

Disintegrin VLO5A OS=Macrovipera lebetina obtusa OX=209528 PE=1 SV=1

Observed	Mr(expt)	Mr(calc)	ppm	Start	End	Miss	Ions	Peptide
1029.5195	1028.5122	1028.4822	29.2	16 -	24	1	---	R.RGEHCVSGK.C
1761.7746	1760.7673	1760.7029	36.6	1 -	15	0	55	-.NSGNPCCDPVTCQPR.R
2133.8489	2132.8416	2132.8085	15.5	41 -	59	0	---	R.AVGDDMDDYCTGISSDCPR.N
2149.8328	2148.8255	2148.8035	10.3	41 -	59	0	---	R.AVGDDMDDYCTGISSDCPR.N + Oxidation (M)

No match to: 817.1322, 837.0947, 839.1124, 855.0854, 859.0801, 861.0985, 868.5550, 871.0587, 874.0421, 875.0528, 877.0736, 887.0291, 893.0448, 896.0269, 903.4585, 909.4351, 925.3589, 1022.1395, 1044.1206, 1055.5381, 1064.0886, 1066.1082, 1074.6014, 1082.0785, 1098.0510, 1108.5873, 1113.7325, 1129.6079, 1146.5988, 1161.6304, 1162.6108, 1163.5712, 1165.6093, 1176.6148, 1179.6216, 1187.5867, 1191.6235, 1203.5432, 1207.6186, 1249.1296, 1254.6020, 1271.1102, 1287.0895, 1317.5934, 1318.1391, 1318.5393, 1410.6058, 1455.6296, 1467.6363, 1474.6385, 1476.1205, 1488.6501, 1500.6546, 1608.7985, 1647.7052, 1756.7243, 1791.7565, 1826.7598, 1872.7755, 1886.7809, 1892.8119, 1908.7852, 1923.7729, 1962.7592, 1975.7640, 1978.7453, 1992.7542, 2016.6822, 2094.7906, 2227.0048, 2245.0062, 2265.1728, 2277.1109, 2631.2491, 2647.2327, 2839.3395, 2887.3153, 2914.5357, 3094.6507, 3346.7074, 3350.6517

13. A0A1I9KNR7_VIPAA **Mass:** 14615 **Score:** 27 **Expect:** 2.9 **Matches:** 1

Disintegrin Dis-1 OS=Vipera ammodytes ammodytes OX=8705 PE=2 SV=1

Observed	Mr(expt)	Mr(calc)	ppm	Start	End	Miss	Ions	Peptide
1318.5393	1317.5320	1317.5013	23.3	64 -	74	0	22	K.GEHCVSGPCCR.N

No match to: 817.1322, 837.0947, 839.1124, 855.0854, 859.0801, 861.0985, 868.5550, 871.0587, 874.0421, 875.0528, 877.0736, 887.0291, 893.0448, 896.0269, 903.4585, 909.4351, 925.3589, 1022.1395, 1029.5195, 1044.1206, 1055.5381, 1064.0886, 1066.1082, 1074.6014, 1082.0785, 1098.0510, 1108.5873, 1113.7325, 1129.6079, 1146.5988, 1161.6304, 1162.6108, 1163.5712, 1165.6093, 1176.6148, 1179.6216, 1187.5867, 1191.6235, 1203.5432, 1207.6186, 1249.1296, 1254.6020, 1271.1102, 1287.0895, 1317.5934, 1318.1391, 1410.6058, 1455.6296, 1467.6363, 1474.6385, 1476.1205, 1488.6501, 1500.6546, 1608.7985, 1647.7052, 1756.7243, 1761.7746, 1791.7565, 1826.7598, 1872.7755, 1886.7809, 1892.8119, 1908.7852, 1923.7729, 1962.7592, 1975.7640, 1978.7453, 1992.7542, 2016.6822, 2094.7906, 2133.8489, 2149.8328, 2227.0048, 2245.0062, 2265.1728, 2277.1109, 2631.2491, 2647.2327, 2839.3395, 2887.3153, 2914.5357, 3094.6507, 3346.7074, 3350.6517

14. A0A1I9KNR4_VIPAA **Mass:** 14601 **Score:** 27 **Expect:** 2.9 **Matches:** 1

Disintegrin Dis-1 OS=Vipera ammodytes ammodytes OX=8705 PE=2 SV=1

Observed	Mr(expt)	Mr(calc)	ppm	Start	End	Miss	Ions	Peptide
1318.5393	1317.5320	1317.5013	23.3	64 -	74	0	22	K.GEHCVSGPCCR.N

No match to: 817.1322, 837.0947, 839.1124, 855.0854, 859.0801, 861.0985, 868.5550, 871.0587, 874.0421, 875.0528, 877.0736, 887.0291, 893.0448, 896.0269, 903.4585, 909.4351, 925.3589, 1022.1395, 1029.5195, 1044.1206, 1055.5381, 1064.0886, 1066.1082, 1074.6014, 1082.0785, 1098.0510, 1108.5873, 1113.7325, 1129.6079, 1146.5988, 1161.6304, 1162.6108, 1163.5712, 1165.6093, 1176.6148, 1179.6216, 1187.5867, 1191.6235, 1203.5432, 1207.6186, 1249.1296, 1254.6020, 1271.1102, 1287.0895, 1317.5934, 1318.1391, 1410.6058, 1455.6296, 1467.6363, 1474.6385, 1476.1205, 1488.6501, 1500.6546, 1608.7985, 1647.7052, 1756.7243, 1761.7746, 1791.7565, 1826.7598, 1872.7755, 1886.7809, 1892.8119, 1908.7852, 1923.7729, 1962.7592, 1975.7640, 1978.7453, 1992.7542, 2016.6822, 2094.7906, 2133.8489, 2149.8328, 2227.0048, 2245.0062, 2265.1728, 2277.1109, 2631.2491, 2647.2327, 2839.3395, 2887.3153, 2914.5357, 3094.6507, 3346.7074, 3350.6517

15. DID2A_MACLB **Mass:** 14638 **Score:** 27 **Expect:** 2.9 **Matches:** 1

Disintegrin lebein-2-alpha OS=Macrovipera lebetina OX=8709 PE=1 SV=1

Observed	Mr(expt)	Mr(calc)	ppm	Start	End	Miss	Ions	Peptide
1318.5393	1317.5320	1317.5013	23.3	64 -	74	0	22	K.GEHCVSGPCCR.N

No match to: 817.1322, 837.0947, 839.1124, 855.0854, 859.0801, 861.0985, 868.5550, 871.0587, 874.0421, 875.0528, 877.0736, 887.0291, 893.0448, 896.0269, 903.4585, 909.4351, 925.3589, 1022.1395, 1029.5195, 1044.1206, 1055.5381, 1064.0886, 1066.1082, 1074.6014, 1082.0785, 1098.0510, 1108.5873, 1113.7325, 1129.6079, 1146.5988, 1161.6304, 1162.6108, 1163.5712, 1165.6093, 1176.6148, 1179.6216, 1187.5867, 1191.6235, 1203.5432, 1207.6186, 1249.1296, 1254.6020, 1271.1102, 1287.0895, 1317.5934, 1318.1391, 1410.6058, 1455.6296, 1467.6363, 1474.6385, 1476.1205, 1488.6501, 1500.6546, 1608.7985, 1647.7052, 1756.7243, 1761.7746, 1791.7565, 1826.7598, 1872.7755, 1886.7809, 1892.8119, 1908.7852, 1923.7729, 1962.7592, 1975.7640, 1978.7453, 1992.7542, 2016.6822, 2094.7906, 2133.8489, 2149.8328, 2227.0048, 2245.0062, 2265.1728, 2277.1109, 2631.2491, 2647.2327, 2839.3395, 2887.3153, 2914.5357, 3094.6507, 3346.7074, 3350.6517

16. A0A6B7FRF2_VIPAA **Mass:** 57466 **Score:** 25 **Expect:** 4.7 **Matches:** 6

Amine oxidase OS=Vipera ammodytes ammodytes OX=8705 PE=2 SV=1

Observed	Mr(expt)	Mr(calc)	ppm	Start	End	Miss	Ions	Peptide
903.4585	902.4512	902.4457	6.08	497 -	504	1	---	R.DVNRASEL.-
1165.6093	1164.6021	1164.6907	-76.06	320 -	329	1	---	R.IKFEPPLPPK.K
1500.6546	1499.6474	1499.6667	-12.92	31 -	42	0	---	R.EDDYEEFLEIAK.N
2265.1728	2264.1655	2264.2100	-19.65	53 -	76	0	---	K.HIVVVGAGMSGLSAAYVLAGAGHK.V
2887.3153	2886.3080	2886.3528	-15.52	250 -	274	1	---	K.RFDEIVGGMDQLPTSMYQAIEESVR.F + Oxidation (M)
3346.7074	3345.7001	3345.5962	31.0	210 -	240	0	---	K.EANLSPGAVDMIGDLLNEDSGYYVSFIESLK.H

No match to: 817.1322, 837.0947, 839.1124, 855.0854, 859.0801, 861.0985, 868.5550, 871.0587, 874.0421, 875.0528, 877.0736, 887.0291, 893.0448, 896.0269, 909.4351, 925.3589, 1022.1395, 1029.5195, 1044.1206, 1055.5381, 1064.0886, 1066.1082, 1074.6014, 1082.0785, 1098.0510, 1108.5873, 1113.7325, 1129.6079, 1146.5988, 1161.6304, 1162.6108, 1163.5712, 1176.6148, 1179.6216, 1187.5867, 1191.6235, 1203.5432, 1207.6186, 1249.1296, 1254.6020, 1271.1102, 1287.0895, 1317.5934, 1318.1391, 1318.5393, 1410.6058, 1455.6296, 1467.6363, 1474.6385, 1476.1205, 1488.6501, 1608.7985, 1647.7052, 1756.7243, 1761.7746, 1791.7565, 1826.7598, 1872.7755, 1886.7809, 1892.8119, 1908.7852, 1923.7729, 1962.7592, 1975.7640, 1978.7453, 1992.7542, 2016.6822, 2094.7906, 2133.8489, 2149.8328, 2227.0048, 2245.0062, 2277.1109, 2631.2491, 2647.2327, 2839.3395, 2914.5357, 3094.6507, 3350.6517

17. OXLA_MACLB **Mass:** 12541 **Score:** 21 **Expect:** 12 **Matches:** 3

L-amino-acid oxidase (Fragments) OS=Macrovipera lebetina OX=8709 PE=1 SV=2

Observed	Mr(expt)	Mr(calc)	ppm	Start	End	Miss	Ions	Peptide
1254.6020	1253.5947	1253.5962	-1.15	58 -	68	1	---	K.FDEIVGGMDKK.F + Oxidation (M)
1500.6546	1499.6474	1499.6667	-12.92	13 -	24	0	---	R.EDDYEEFLEIAK.N
2245.0062	2243.9989	2244.0045	-2.48	49 -	67	1	---	K.HDDIFAYEKFDEIVGGMDK.K + Oxidation (M)

No match to: 817.1322, 837.0947, 839.1124, 855.0854, 859.0801, 861.0985, 868.5550, 871.0587, 874.0421, 875.0528, 877.0736, 887.0291, 893.0448, 896.0269, 903.4585, 909.4351, 925.3589, 1022.1395, 1029.5195, 1044.1206, 1055.5381, 1064.0886, 1066.1082, 1074.6014, 1082.0785, 1098.0510, 1108.5873, 1113.7325, 1129.6079, 1146.5988, 1161.6304, 1162.6108, 1163.5712, 1165.6093, 1176.6148, 1179.6216, 1187.5867, 1191.6235, 1203.5432, 1207.6186, 1249.1296, 1271.1102, 1287.0895, 1317.5934, 1318.1391, 1318.5393, 1410.6058, 1455.6296, 1467.6363, 1474.6385, 1476.1205, 1488.6501, 1608.7985, 1647.7052, 1756.7243, 1761.7746, 1791.7565, 1826.7598, 1872.7755, 1886.7809, 1892.8119, 1908.7852, 1923.7729, 1962.7592, 1975.7640, 1978.7453, 1992.7542, 2016.6822, 2094.7906, 2133.8489, 2149.8328, 2227.0048, 2265.1728, 2277.1109, 2631.2491, 2647.2327, 2839.3395, 2887.3153, 2914.5357, 3094.6507, 3346.7074, 3350.6517

18. A0A6G5ZUD1_9SAUR **Mass:** 30200 **Score:** 19 **Expect:** 20 **Matches:** 4

Lysosomal acid lipase/cholesteryl ester hydrolase 2 (Fragment) OS=Vipera anatolica senliki OX=2604287 PE=2 SV=1

Observed	Mr(expt)	Mr(calc)	ppm	Start	End	Miss	Ions	Peptide
1162.6108	1161.6035	1161.6104	-5.88	75 -	84	0	---	K.LGDLPEFMIK.E
1467.6363	1466.6290	1466.6655	-24.85	246 -	256	1	---	R.MFRDMIQMMHK.V
1647.7052	1646.6980	1646.7367	-23.54	249 -	261	1	---	R.DMIQMMHKVQYAH.- + Oxidation (M)
2839.3395	2838.3322	2838.4235	-32.18	136 -	160	1	---	R.VEVYSTHCPAGTSVRNILHWSQAVK.S

No match to: 817.1322, 837.0947, 839.1124, 855.0854, 859.0801, 861.0985, 868.5550, 871.0587, 874.0421, 875.0528, 877.0736, 887.0291, 893.0448, 896.0269, 903.4585, 909.4351, 925.3589, 1022.1395, 1029.5195, 1044.1206, 1055.5381, 1064.0886, 1066.1082, 1074.6014, 1082.0785, 1098.0510, 1108.5873, 1113.7325, 1129.6079, 1146.5988, 1161.6304, 1163.5712, 1165.6093, 1176.6148, 1179.6216, 1187.5867, 1191.6235, 1203.5432, 1207.6186, 1249.1296, 1254.6020, 1271.1102, 1287.0895, 1317.5934, 1318.1391, 1318.5393, 1410.6058, 1455.6296, 1474.6385, 1476.1205, 1488.6501, 1500.6546, 1608.7985, 1756.7243, 1761.7746, 1791.7565, 1826.7598, 1872.7755, 1886.7809, 1892.8119, 1908.7852, 1923.7729, 1962.7592, 1975.7640, 1978.7453, 1992.7542, 2016.6822, 2094.7906, 2133.8489, 2149.8328, 2227.0048, 2245.0062, 2265.1728, 2277.1109, 2631.2491, 2647.2327, 2887.3153, 2914.5357, 3094.6507, 3346.7074, 3350.6517

19. DIS_MACLO **Mass:** 4854 **Score:** 18 **Expect:** 25 **Matches:** 2

Disintegrin obtustatin OS=Macrovipera lebetina obtusa OX=209528 PE=1 SV=1

Observed	Mr(expt)	Mr(calc)	ppm	Start	End	Miss	Ions	Peptide
1161.6304	1160.6231	1160.6012	18.9	12 -	21	0	---	K.LKPAGTTCWK.T
1254.6020	1253.5947	1253.5710	18.9	22 -	32	0	---	K.TSLTSHYCTGK.S

No match to: 817.1322, 837.0947, 839.1124, 855.0854, 859.0801, 861.0985, 868.5550, 871.0587, 874.0421, 875.0528, 877.0736, 887.0291, 893.0448, 896.0269, 903.4585, 909.4351, 925.3589, 1022.1395, 1029.5195, 1044.1206, 1055.5381, 1064.0886, 1066.1082, 1074.6014, 1082.0785, 1098.0510, 1108.5873, 1113.7325, 1129.6079, 1146.5988, 1162.6108, 1163.5712, 1165.6093, 1176.6148, 1179.6216, 1187.5867, 1191.6235, 1203.5432, 1207.6186, 1249.1296, 1271.1102, 1287.0895, 1317.5934, 1318.1391, 1318.5393, 1410.6058, 1455.6296, 1467.6363, 1474.6385, 1476.1205, 1488.6501, 1500.6546, 1608.7985, 1647.7052, 1756.7243, 1761.7746, 1791.7565, 1826.7598, 1872.7755, 1886.7809, 1892.8119, 1908.7852, 1923.7729, 1962.7592, 1975.7640, 1978.7453, 1992.7542, 2016.6822, 2094.7906, 2133.8489, 2149.8328, 2227.0048, 2245.0062, 2265.1728, 2277.1109, 2631.2491, 2647.2327, 2839.3395, 2887.3153, 2914.5357, 3094.6507, 3346.7074, 3350.6517

20. M4T472_9PERO **Mass:** 26404 **Score:** 17 **Expect:** 30 **Matches:** 4

Ring finger protein 213 (Fragment) OS=Echiichthys vipera OX=94984 PE=4 SV=1

Observed	Mr(expt)	Mr(calc)	ppm	Start	End	Miss	Ions	Peptide
903.4585	902.4512	902.4466	5.11	94 -	100	0	---	K.MLAIHMR.F + 2 Oxidation (M)
1029.5195	1028.5122	1028.4709	40.1	221 -	228	0	---	K.HTDEMIQR.L
1163.5712	1162.5639	1162.6346	-60.77	40 -	49	0	---	R.ALYEGLTLQR.V
1191.6235	1190.6162	1190.6845	-57.40	120 -	128	1	---	R.LIKFLCELR.R

No match to: 817.1322, 837.0947, 839.1124, 855.0854, 859.0801, 861.0985, 868.5550, 871.0587, 874.0421, 875.0528, 877.0736, 887.0291, 893.0448, 896.0269, 909.4351, 925.3589, 1022.1395, 1044.1206, 1055.5381, 1064.0886, 1066.1082, 1074.6014, 1082.0785, 1098.0510, 1108.5873, 1113.7325, 1129.6079, 1146.5988, 1161.6304, 1162.6108, 1165.6093, 1176.6148, 1179.6216, 1187.5867, 1203.5432, 1207.6186, 1249.1296, 1254.6020, 1271.1102, 1287.0895, 1317.5934, 1318.1391, 1318.5393, 1410.6058, 1455.6296, 1467.6363, 1474.6385, 1476.1205, 1488.6501, 1500.6546, 1608.7985, 1647.7052, 1756.7243, 1761.7746, 1791.7565, 1826.7598, 1872.7755, 1886.7809, 1892.8119, 1908.7852, 1923.7729, 1962.7592, 1975.7640, 1978.7453, 1992.7542, 2016.6822, 2094.7906, 2133.8489, 2149.8328, 2227.0048,

2245.0062, 2265.1728, 2277.1109, 2631.2491, 2647.2327, 2839.3395, 2887.3153, 2914.5357, 3094.6507, 3346.7074, 3350.6517

Search Parameters

```
Type of search         : MS/MS Ion Search
Enzyme                 : Trypsin
Fixed modifications    : Carbamidomethyl (C)
Variable modifications : Oxidation (M)
Mass values            : Monoisotopic
Protein Mass           : Unrestricted
Peptide Mass Tolerance : ± 100 ppm
Fragment Mass Tolerance: ± 0.7 Da
Max Missed Cleavages   : 1
Instrument type        : MALDI-TOF-TOF
Query1 (817.1322,1+)   : <no title>
Query2 (837.0947,1+)   : <no title>
Query3 (839.1124,1+)   : <no title>
Query4 (855.0854,1+)   : \\PROTEOMICS1\D\DATA\2105\210505_TLIEPOL_1_68252-68266\68252_68266_LPARDO\0_H6\1\855.0854.LIFT\1SREF\PDATA\1\1R
Query5 (859.0801,1+)   : <no title>
Query6 (861.0985,1+)   : <no title>
Query7 (868.5550,1+)   : <no title>
Query8 (871.0587,1+)   : \\PROTEOMICS1\D\DATA\2105\210505_TLIEPOL_1_68252-68266\68252_68266_LPARDO\0_H6\1\871.0587.LIFT\1SREF\PDATA\1\1R
Query9 (874.0421,1+)   : <no title>
Query10 (875.0528,1+)  : <no title>
Query11 (877.0736,1+)  : <no title>
Query12 (887.0291,1+)  : <no title>
Query13 (893.0448,1+)  : <no title>
Query14 (896.0269,1+)  : <no title>
Query15 (903.4585,1+)  : <no title>
Query16 (909.4351,1+)  : <no title>
Query17 (925.3589,1+)  : <no title>
Query18 (1022.1395,1+) : <no title>
Query19 (1029.5195,1+) : <no title>
Query20 (1044.1206,1+) : <no title>
Query21 (1055.5381,1+) : <no title>
Query22 (1064.0886,1+) : <no title>
Query23 (1066.1082,1+) : <no title>
Query24 (1074.6014,1+) : <no title>
Query25 (1082.0785,1+) : <no title>
Query26 (1098.0510,1+) : <no title>
Query27 (1108.5873,1+) : <no title>
Query28 (1113.7325,1+) : <no title>
Query29 (1129.6079,1+) : <no title>
Query30 (1146.5988,1+) : <no title>
Query31 (1161.6304,1+) : <no title>
Query32 (1162.6108,1+) : <no title>
Query33 (1163.5712,1+) : <no title>
Query34 (1165.6093,1+) : \\PROTEOMICS1\D\DATA\2105\210505_TLIEPOL_1_68252-68266\68252_68266_LPARDO\0_H6\1\1165.6093.LIFT\1SREF\PDATA\1\1R
Query35 (1176.6148,1+) : <no title>
Query36 (1179.6216,1+) : <no title>
Query37 (1187.5867,1+) : <no title>
Query38 (1191.6235,1+) : <no title>
Query39 (1203.5432,1+) : <no title>
Query40 (1207.6186,1+) : <no title>
Query41 (1249.1296,1+) : <no title>
Query42 (1254.6020,1+) : <no title>
Query43 (1271.1102,1+) : <no title>
Query44 (1287.0895,1+) : <no title>
Query45 (1317.5934,1+) : <no title>
Query46 (1318.1391,1+) : <no title>
Query47 (1318.5393,1+) : \\PROTEOMICS1\D\DATA\2105\210505_TLIEPOL_1_68252-68266\68252_68266_LPARDO\0_H6\1\1318.5393.LIFT\1SREF\PDATA\1\1R
Query48 (1410.6058,1+) : <no title>
Query49 (1455.6296,1+) : <no title>
Query50 (1467.6363,1+) : <no title>
Query51 (1474.6385,1+) : \\PROTEOMICS1\D\DATA\2105\210505_TLIEPOL_1_68252-68266\68252_68266_LPARDO\0_H6\1\1474.6385.LIFT\1SREF\PDATA\1\1R
Query52 (1476.1205,1+) : <no title>
Query53 (1488.6501,1+) : <no title>
Query54 (1500.6546,1+) : <no title>
Query55 (1608.7985,1+) : <no title>
Query56 (1647.7052,1+) : <no title>
Query57 (1756.7243,1+) : <no title>
Query58 (1761.7746,1+) : \\PROTEOMICS1\D\DATA\2105\210505_TLIEPOL_1_68252-68266\68252_68266_LPARDO\0_H6\1\1761.7746.LIFT\1SREF\PDATA\1\1R
Query59 (1791.7565,1+) : <no title>
Query60 (1826.7598,1+) : <no title>
Query61 (1872.7755,1+) : \\PROTEOMICS1\D\DATA\2105\210505_TLIEPOL_1_68252-68266\68252_68266_LPARDO\0_H6\1\1872.7755.LIFT\1SREF\PDATA\1\1R
Query62 (1886.7809,1+) : <no title>
Query63 (1892.8119,1+) : <no title>
Query64 (1908.7852,1+) : <no title>
Query65 (1923.7729,1+) : <no title>
Query66 (1962.7592,1+) : \\PROTEOMICS1\D\DATA\2105\210505_TLIEPOL_1_68252-68266\68252_68266_LPARDO\0_H6\1\1962.7592.LIFT\1SREF\PDATA\1\1R
Query67 (1975.7640,1+) : <no title>
Query68 (1978.7453,1+) : \\PROTEOMICS1\D\DATA\2105\210505_TLIEPOL_1_68252-68266\68252_68266_LPARDO\0_H6\1\1978.7453.LIFT\1SREF\PDATA\1\1R
Query69 (1992.7542,1+) : <no title>
Query70 (2016.6822,1+) : <no title>
Query71 (2094.7906,1+) : <no title>
Query72 (2133.8489,1+) : <no title>
Query73 (2149.8328,1+) : <no title>
Query74 (2227.0048,1+) : <no title>
Query75 (2245.0062,1+) : <no title>
Query76 (2265.1728,1+) : <no title>
Query77 (2277.1109,1+) : <no title>
Query78 (2631.2491,1+) : <no title>
Query79 (2647.2327,1+) : <no title>
Query80 (2839.3395,1+) : <no title>
Query81 (2887.3153,1+) : <no title>
Query82 (2914.5357,1+) : <no title>
Query83 (3094.6507,1+) : <no title>
Query84 (3346.7074,1+) : <no title>
Query85 (3350.6517,1+) : <no title>
```

Mascot: http://www.matrixscience.com/

Mascot Search Results Fr3-2

User :
Email :
Search title : vipera_All_8IDplus4 file:\\Proteomics1\d\data\2105\210505_tliepol_1_68252-68266\68252_68266_lpardo\0_G7\1\1SRef\pdata\1\peaklist.xml
Database : vipera (1414 sequences; 347452 residues)
Timestamp : 5 May 2021 at 09:34:52 GMT
Top Score : 57 for DIS_MACLO, Disintegrin obtustatin OS=Macrovipera lebetina obtusa OX=209528 PE=1 SV=1

Mascot Score Histogram

Protein score is -10*Log(P), where P is the probability that the observed match is a random event.
Protein scores greater than 44 are significant (p<0.05).

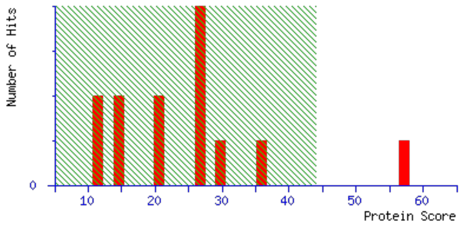

Concise Protein Summary Report

Format As | Concise Protein Summary | Help

Significance threshold p< 0.05 Max. number of hits 10

Re-Search All | Search Unmatched

1. DIS_MACLO **Mass:** 4854 **Score:** 57 **Expect:** 0.0027 **Matches:** 4
Disintegrin obtustatin OS=Macrovipera lebetina obtusa OX=209528 PE=1 SV=1
Q1JRG7_MACLN **Mass:** 5041 **Score:** 35 **Expect:** 0.44 **Matches:** 2
RTS-containing short disintegrin ML-G3 (Fragment) OS=Macrovipera lebetina transmediterranea OX=384075 GN=ml-G3 PE=4 SV=1
DIS_CERVI **Mass:** 5041 **Score:** 35 **Expect:** 0.44 **Matches:** 2
Disintegrin CV OS=Cerastes vipera OX=8698 PE=2 SV=1
OXLA_MACLB **Mass:** 12541 **Score:** 13 **Expect:** 76 **Matches:** 1
L-amino-acid oxidase (Fragments) OS=Macrovipera lebetina OX=8709 PE=1 SV=2
A0A1B3B3L1_9SAUR **Mass:** 11972 **Score:** 12 **Expect:** 85 **Matches:** 1
Jun proto-oncogene (Fragment) OS=Vipera eriwanensis OX=796011 GN=jun PE=4 SV=1
A0A6G5ZVM2_9SAUR **Mass:** 13023 **Score:** 12 **Expect:** 85 **Matches:** 1
Myotrophin OS=Vipera anatolica senliki OX=2604287 PE=2 SV=1

2. DIS_DABPA **Mass:** 4914 **Score:** 35 **Expect:** 0.42 **Matches:** 3
Disintegrin viperistatin OS=Daboia palaestinae OX=1170828 PE=1 SV=1

3. DIS_MACLB **Mass:** 12004 **Score:** 28 **Expect:** 2 **Matches:** 3
Disintegrin lebestatin OS=Macrovipera lebetina OX=8709 PE=1 SV=1

4. PA2A2_VIPRE **Mass:** 16161 **Score:** 28 **Expect:** 2.2 **Matches:** 2
Acidic phospholipase A2 Vur-PL2B OS=Vipera renardi OX=927686 PE=1 SV=1
PA2A_VIPAE **Mass:** 14428 **Score:** 12 **Expect:** 96 **Matches:** 1
Acidic phospholipase A2 homolog vipoxin A chain OS=Vipera ammodytes meridionalis OX=73841 PE=1 SV=3
Q6A3N1_VIPAZ **Mass:** 14972 **Score:** 12 **Expect:** 98 **Matches:** 1
Vaspin acidic subunit (1) variant OS=Vipera aspis zinnikeri OX=55427 PE=2 SV=1
Q6A3N2_VIPAZ **Mass:** 15032 **Score:** 12 **Expect:** 98 **Matches:** 1
Vaspin acidic subunit (1) variant OS=Vipera aspis zinnikeri OX=55427 PE=2 SV=1
PA2AB_DABSI **Mass:** 15673 **Score:** 12 **Expect:** 1e+002 **Matches:** 1
Acidic phospholipase A2 daboiatoxin B chain (Fragment) OS=Daboia siamensis OX=343250 PE=1 SV=1
Q6A3G8_VIPAA **Mass:** 15517 **Score:** 11 **Expect:** 1e+002 **Matches:** 1
Ammodytin I1(A) variant OS=Vipera ammodytes ammodytes OX=8705 PE=2 SV=1
Q6A3A3_VIPAM **Mass:** 16235 **Score:** 11 **Expect:** 1e+002 **Matches:** 1
Ammodytin I2(C) isoform OS=Vipera ammodytes montandoni OX=235554 PE=2 SV=1
Q6YCN7_VIPBB **Mass:** 16154 **Score:** 11 **Expect:** 1e+002 **Matches:** 1
Ammodytin I2 OS=Vipera berus berus OX=31156 GN=AmtI2 PE=2 SV=1
Q6A3C0_VIPAP **Mass:** 16127 **Score:** 11 **Expect:** 1e+002 **Matches:** 1
Ammodytin I2(B) isoform OS=Vipera aspis aspis OX=194601 PE=2 SV=1
Q6A3E7_VIPBB **Mass:** 16142 **Score:** 11 **Expect:** 1e+002 **Matches:** 1
Ammodytin I2(A) variant OS=Vipera berus berus OX=31156 PE=2 SV=1
Q6A3C3_VIPBB **Mass:** 16120 **Score:** 11 **Expect:** 1e+002 **Matches:** 1
Ammodytin I2(A) variant OS=Vipera berus berus OX=31156 PE=2 SV=1
Q6A3A7_VIPAE **Mass:** 16265 **Score:** 11 **Expect:** 1e+002 **Matches:** 1
Ammodytin I2(C) variant OS=Vipera ammodytes meridionalis OX=73841 PE=2 SV=1
Q6A3B5_VIPAS **Mass:** 16127 **Score:** 11 **Expect:** 1e+002 **Matches:** 1
Ammodytin I2(B) isoform OS=Vipera aspis atra OX=246182 PE=2 SV=1
Q6A3C2_VIPUR **Mass:** 16136 **Score:** 11 **Expect:** 1e+002 **Matches:** 1
Ammodytin I2(D) isoform OS=Vipera ursinii OX=103942 PE=2 SV=1
Q6YCN8_VIPAS **Mass:** 16154 **Score:** 11 **Expect:** 1e+002 **Matches:** 1
Ammodytin I2 OS=Vipera aspis OX=8706 GN=AmtI2 PE=3 SV=1
Q6A3A1_VIPAM **Mass:** 16263 **Score:** 11 **Expect:** 1e+002 **Matches:** 1
Ammodytin I2(C) variant OS=Vipera ammodytes montandoni OX=235554 PE=2 SV=1
Q6A3A0_VIPAM **Mass:** 16163 **Score:** 11 **Expect:** 1e+002 **Matches:** 1
Ammodytin I2(C) variant OS=Vipera ammodytes montandoni OX=235554 PE=2 SV=1
Q6A3A4_VIPAE **Mass:** 16220 **Score:** 11 **Expect:** 1e+002 **Matches:** 1
Ammodytin I2(C) isoform OS=Vipera ammodytes meridionalis OX=73841 PE=2 SV=1
Q6A3A5_VIPAE **Mass:** 16219 **Score:** 11 **Expect:** 1e+002 **Matches:** 1
Ammodytin I2(C) variant OS=Vipera ammodytes meridionalis OX=73841 PE=2 SV=1
Q6A3F4_VIPAP **Mass:** 16124 **Score:** 11 **Expect:** 1e+002 **Matches:** 1
Ammodytin I2(A) variant OS=Vipera aspis aspis OX=194601 PE=2 SV=1

Q6A3E3_VIPAP **Mass:** 16138 **Score:** 11 **Expect:** 1e+002 **Matches:** 1
Ammodytin I2(A''') isoform OS=Vipera aspis aspis OX=194601 PE=2 SV=1
Q6A3D7_VIPAP **Mass:** 16096 **Score:** 11 **Expect:** 1e+002 **Matches:** 1
Ammodytin I2(A) variant OS=Vipera aspis aspis OX=194601 PE=2 SV=1
Q6A3D4_VIPAP **Mass:** 16151 **Score:** 11 **Expect:** 1e+002 **Matches:** 1
Ammodytin I2(A) variant OS=Vipera aspis aspis OX=194601 PE=2 SV=1
Q6A3E0_VIPAP **Mass:** 16170 **Score:** 11 **Expect:** 1e+002 **Matches:** 1
Ammodytin I2(A) variant OS=Vipera aspis aspis OX=194601 PE=2 SV=1
Q6A3D1_VIPAP **Mass:** 16168 **Score:** 11 **Expect:** 1e+002 **Matches:** 1
Ammodytin I2(A) variant OS=Vipera aspis aspis OX=194601 PE=2 SV=1
Q6A3C9_VIPAP **Mass:** 16169 **Score:** 11 **Expect:** 1e+002 **Matches:** 1
Ammodytin I2(A) variant OS=Vipera aspis aspis OX=194601 PE=2 SV=1
Q6A3B9_VIPAP **Mass:** 16093 **Score:** 11 **Expect:** 1e+002 **Matches:** 1
Ammodytin I2(B) variant OS=Vipera aspis aspis OX=194601 PE=2 SV=1
Q6A3D3_VIPAP **Mass:** 16178 **Score:** 11 **Expect:** 1e+002 **Matches:** 1
Ammodytin I2(A) variant OS=Vipera aspis aspis OX=194601 PE=2 SV=1

---

5. Q8UVG8_DABRR **Mass:** 52931 **Score:** 26 **Expect:** 3.2 **Matches:** 6
Vimentin OS=Daboia russelii OX=8707 PE=2 SV=1

---

6. Q6A3M4_VIPAP **Mass:** 16217 **Score:** 26 **Expect:** 3.6 **Matches:** 2
Ammodytin I1(C) variant OS=Vipera aspis aspis OX=194601 PE=2 SV=1
A0A6G5ZVX2_9SAUR **Mass:** 16256 **Score:** 11 **Expect:** 1e+002 **Matches:** 1
Cystatin 1 OS=Vipera anatolica senliki OX=2604287 PE=2 SV=1

---

7. PA2B1_DABRR **Mass:** 16709 **Score:** 26 **Expect:** 3.6 **Matches:** 3
Basic phospholipase A2 Drk-b1 OS=Daboia russelii OX=8707 PE=1 SV=1

---

8. K4GS13_DABRR **Mass:** 30008 **Score:** 21 **Expect:** 11 **Matches:** 4
Ubiquitinyl hydrolase 1 (Fragment) OS=Daboia russelii OX=8707 GN=VCPIP PE=4 SV=1

---

9. A0A6G5ZUS0_9SAUR **Mass:** 42933 **Score:** 20 **Expect:** 16 **Matches:** 4
Glutaminyl cyclase OS=Vipera anatolica senliki OX=2604287 PE=2 SV=1
DID2A_MACLB **Mass:** 14638 **Score:** 13 **Expect:** 65 **Matches:** 1
Disintegrin lebein-2-alpha OS=Macrovipera lebetina OX=8709 PE=1 SV=1

---

10. R4NNL0_VIPAA **Mass:** 70725 **Score:** 14 **Expect:** 50 **Matches:** 2
H3 metalloproteinase 1 OS=Vipera ammodytes ammodytes OX=8705 PE=2 SV=1
A0A6B7FNN4_VIPAA **Mass:** 70467 **Score:** 14 **Expect:** 50 **Matches:** 2
Metalloproteinase of class P-III MPIII-5 OS=Vipera ammodytes ammodytes OX=8705 PE=2 SV=1
A0A1S5QK73_VIPAA **Mass:** 10753 **Score:** 13 **Expect:** 68 **Matches:** 1
Kunitz/BPTI inhibitor-5 OS=Vipera ammodytes ammodytes OX=8705 PE=3 SV=1
SLB_DABPA **Mass:** 15546 **Score:** 12 **Expect:** 93 **Matches:** 1
Snaclec VP12 subunit B OS=Daboia palaestinae OX=1170828 PE=1 SV=1

Search Parameters

Type of search : Peptide Mass Fingerprint
Enzyme : Trypsin
Fixed modifications : Carbamidomethyl (C)
Variable modifications : Oxidation (M)
Mass values : Monoisotopic
Protein Mass : Unrestricted
Peptide Mass Tolerance : ± 100 ppm
Peptide Charge State : 1+
Max Missed Cleavages : 1
Number of queries : 94
Selected for scoring : 10

Mascot: http://www.matrixscience.com/

MATRIX SCIENCE Mascot Search Results

```
User          :
Email         :
Search title  : vipera_all_Lift file:\\Proteomics1\d\data\2105\210505_tliepol_1_68252-68266\68252_68266_lpardo\0_G7\1\0_G7.xml
Database      : vipera  (1414 sequences; 347452 residues)
Timestamp     : 5 May 2021 at 09:50:14 GMT
Protein hits  : DIS_MACLO        Disintegrin obtustatin OS=Macrovipera lebetina obtusa OX=209528 PE=1 SV=1
                DIS_CERVI        Disintegrin CV OS=Cerastes vipera OX=8698 PE=2 SV=1
                A0A1S5QK15_VIPAA Kunitz/BPTI inhibitor-4 (Fragment) OS=Vipera ammodytes ammodytes OX=8705 PE=3 SV=1
```

Mascot Score Histogram

Ions score is -10*Log(P), where P is the probability that the observed match is a random event.
Individual ions scores > 10 indicate identity or extensive homology (p<0.05).
Protein scores are derived from ions scores as a non-probabilistic basis for ranking protein hits.

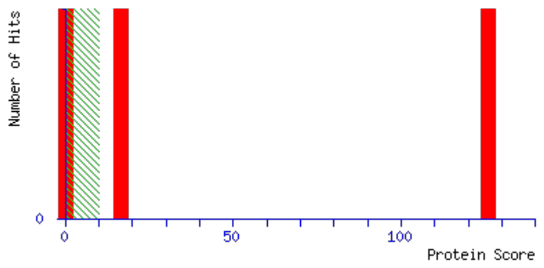

Peptide Summary Report

Format As: Peptide Summary — Help

Significance threshold p< 0.05 — Max. number of hits 10

Standard scoring ○ MudPIT scoring ◉ — Ions score or expect cut-off 0 — Show sub-sets 0

Show pop-ups ◉ Suppress pop-ups ○ — Sort unassigned Decreasing Score — Require bold red ☐

Overview Table

Click on column header to jump to entry in results list.
Move mouse over any indicator to highlight identical peptides.
Click on an indicator to see details of individual match.
Use check boxes to select sub-set of queries for new search.

Mouse over: -Query- -Accession- -Sequence-

Hit:	1	2	3
☑ 855.1002 (1+)			
☑ 863.3795 (1+)			
☑ 868.5621 (1+)			
☑ 871.0730 (1+)			
☑ 875.3810 (1+)			
☑ 877.3714 (1+)			
☑ 895.3562 (1+)			
☑ 897.3687 (1+)			
☑ 903.3991 (1+)			
☑ 905.3927 (1+)			
☑ 907.3757 (1+)			
☑ 909.3719 (1+)			
☑ 920.4159 (1+)			
☑ 921.3920 (1+)			
☑ 925.3669 (1+)			
☑ 935.3831 (1+)			
☑ 937.3702 (1+)			
☑ 947.3855 (1+)			
☑ 952.3780 (1+)			
☑ 953.3893 (1+)			
☑ 954.3922 (1+)			
☑ 966.3939 (1+)			
☑ 968.3917 (1+)			
☑ 977.3909 (1+)			
☑ 979.3925 (1+)			

☑ 980.4000 (1+)			
☑ 982.3881 (1+)			
☑ 984.3620 (1+)			
☑ 986.3655 (1+)			
☑ 992.4049 (1+)			
☑ 993.4047 (1+)			
☑ 994.3935 (1+)			
☑ 996.4006 (1+)			
☑ 998.3753 (1+)			
☑ 1004.3794 (1+)			
☑ 1006.3835 (1+)			
☑ 1009.4004 (1+)			
☑ 1011.4157 (1+)	●	●	
☑ 1022.1538 (1+)			
☑ 1039.4114 (1+)			
☑ 1043.3901 (1+)			
☑ 1044.1374 (1+)			
☑ 1066.1246 (1+)			
☑ 1090.4345 (1+)			
☑ 1106.3991 (1+)			
☑ 1142.6361 (1+)			
☑ 1159.6219 (1+)			
☑ 1161.6411 (1+)	●		
☑ 1163.4129 (1+)			
☑ 1165.6247 (1+)			
☑ 1172.6415 (1+)			
☑ 1175.6433 (1+)			
☑ 1177.6357 (1+)			
☑ 1178.6413 (1+)			
☑ 1183.6355 (1+)			
☑ 1187.6553 (1+)			
☑ 1193.6308 (1+)			
☑ 1197.5917 (1+)			
☑ 1200.6345 (1+)			
☑ 1202.6678 (1+)			
☑ 1206.6509 (1+)			
☑ 1209.6355 (1+)			
☑ 1210.6269 (1+)			
☑ 1215.6644 (1+)			
☑ 1217.6575 (1+)			
☑ 1219.6486 (1+)			
☑ 1225.6373 (1+)			
☑ 1228.6796 (1+)			
☑ 1234.6590 (1+)			
☑ 1249.1486 (1+)			
☑ 1250.6539 (1+)			
☑ 1254.6129 (1+)	●	●	●
☑ 1276.6050 (1+)			
☑ 1280.6284 (1+)			
☑ 1292.5783 (1+)			
☑ 1296.6213 (1+)			
☑ 1322.6371 (1+)			
☑ 1618.8519 (1+)			
☑ 1650.8407 (1+)			
☑ 1872.7759 (1+)			
☑ 1905.7288 (1+)			
☑ 1962.7642 (1+)			
☑ 1974.7638 (1+)			
☑ 1994.0056 (1+)			
☑ 2055.9612 (1+)			
☑ 2148.9294 (1+)			
☑ 2345.0400 (1+)			
☑ 2359.0435 (1+)			
☑ 2397.2085 (1+)	●		
☑ 2438.2368 (1+)			
☑ 2454.2310 (1+)			
☑ 2470.2291 (1+)			

☑ 3346.7170 (1+)			
☑ 3446.6176 (1+)			

Select All | Select None | Search Selected | ☐ Error tolerant | Archive Report

1. DIS_MACLO **Mass:** 4854 **Score:** 126 **Matches:** 4(4) **Sequences:** 4(4)
Disintegrin obtustatin OS=Macrovipera lebetina obtusa OX=209528 PE=1 SV=1
☐ Check to include this hit in error tolerant search or archive report

Query	Observed	Mr(expt)	Mr(calc)	ppm	Miss	Score	Expect	Rank	Unique	Peptide
38	**1011.4157**	**1010.4084**	**1010.3732**	**34.8**	**0**	**15**	**0.033**	**1**		**-.CTTGPCCR.Q**
48	**1161.6411**	**1160.6339**	**1160.6012**	**28.1**	**0**	**68**	**2.2e-007**	**1**	**U**	**K.LKPAGTTCWK.T**
72	**1254.6129**	**1253.6056**	**1253.5710**	**27.6**	**0**	**60**	**1.4e-006**	**1**	**U**	**K.TSLTSHYCTGK.S**
89	**2397.2085**	**2396.2012**	**2396.1617**	**16.5**	**1**	**24**	**0.0039**	**1**	**U**	**K.LKPAGTTCWKTSLTSHYCTGK.S**

2. DIS_CERVI **Mass:** 5041 **Score:** 15 **Matches:** 2(1) **Sequences:** 2(1)
Disintegrin CV OS=Cerastes vipera OX=8698 PE=2 SV=1
☐ Check to include this hit in error tolerant search or archive report

Query	Observed	Mr(expt)	Mr(calc)	ppm	Miss	Score	Expect	Rank	Unique	Peptide
38	1011.4157	1010.4084	1010.3732	34.8	0	15	0.033	1		-.CTTGPCCR.Q
72	1254.6129	1253.6056	1253.5459	47.6	0	4	0.58	3	U	R.TSVSSHYCTGR.S

Proteins matching the same set of peptides:
Q1JRG7_MACLN **Mass:** 5041 **Score:** 15 **Matches:** 2(1) **Sequences:** 2(1)
RTS-containing short disintegrin ML-G3 (Fragment) OS=Macrovipera lebetina transmediterranea OX=384075 GN=ml-G3 PE=4 SV=1

3. A0A1S5QK15_VIPAA **Mass:** 10077 **Score:** 0 **Matches:** 1(0) **Sequences:** 1(0)
Kunitz/BPTI inhibitor-4 (Fragment) OS=Vipera ammodytes ammodytes OX=8705 PE=3 SV=1
☐ Check to include this hit in error tolerant search or archive report

Query	Observed	Mr(expt)	Mr(calc)	ppm	Miss	Score	Expect	Rank	Unique	Peptide
72	1254.6129	1253.6056	1253.6187	-10.40	1	5	0.44	2	U	R.HTCVASGKGIQP.-

Peptide matches not assigned to protein hits: (no details means no match)

	Query	Observed	Mr(expt)	Mr(calc)	ppm	Miss	Score	Expect	Rank	Unique	Peptide
☑	1	*855.1002*	*854.0929*								
☑	2	*863.3795*	*862.3722*								
☑	3	*868.5621*	*867.5549*								
☑	4	*871.0730*	*870.0657*								
☑	5	*875.3810*	*874.3737*								
☑	6	*877.3714*	*876.3641*								
☑	7	*895.3562*	*894.3489*								
☑	8	*897.3687*	*896.3614*								
☑	9	*903.3991*	*902.3918*								
☑	10	*905.3927*	*904.3854*								
☑	11	*907.3757*	*906.3685*								
☑	12	*909.3719*	*908.3646*								
☑	13	*920.4159*	*919.4086*								
☑	14	*921.3920*	*920.3848*								
☑	15	*925.3669*	*924.3596*								
☑	16	*935.3831*	*934.3758*								
☑	17	*937.3702*	*936.3629*								
☑	18	*947.3855*	*946.3782*								
☑	19	*952.3780*	*951.3707*								
☑	20	*953.3893*	*952.3820*								
☑	21	*954.3922*	*953.3849*								
☑	22	*966.3939*	*965.3867*								
☑	23	*968.3917*	*967.3844*								
☑	24	*977.3909*	*976.3836*								
☑	25	*979.3925*	*978.3852*								
☑	26	*980.4000*	*979.3927*								
☑	27	*982.3881*	*981.3808*								
☑	28	*984.3620*	*983.3547*								
☑	29	*986.3655*	*985.3582*								
☑	30	*992.4049*	*991.3977*								
☑	31	*993.4047*	*992.3975*								
☑	32	*994.3935*	*993.3863*								
☑	33	*996.4006*	*995.3933*								
☑	34	*998.3753*	*997.3681*								
☑	35	*1004.3794*	*1003.3722*								
☑	36	*1006.3835*	*1005.3762*								
☑	37	*1009.4004*	*1008.3931*								
☑	39	*1022.1538*	*1021.1465*								
☑	40	*1039.4114*	*1038.4041*								
☑	41	*1043.3901*	*1042.3828*								

☑	42	*1044.1374*	*1043.1301*
☑	43	*1066.1246*	*1065.1174*
☑	44	*1090.4345*	*1089.4272*
☑	45	*1106.3991*	*1105.3919*
☑	46	*1142.6361*	*1141.6288*
☑	47	*1159.6219*	*1158.6146*
☑	49	*1163.4129*	*1162.4057*
☑	50	*1165.6247*	*1164.6175*
☑	51	*1172.6415*	*1171.6342*
☑	52	*1175.6433*	*1174.6360*
☑	53	*1177.6357*	*1176.6285*
☑	54	*1178.6413*	*1177.6340*
☑	55	*1183.6355*	*1182.6282*
☑	56	*1187.6553*	*1186.6480*
☑	57	*1193.6308*	*1192.6235*
☑	58	*1197.5917*	*1196.5844*
☑	59	*1200.6345*	*1199.6273*
☑	60	*1202.6678*	*1201.6606*
☑	61	*1206.6509*	*1205.6436*
☑	62	*1209.6355*	*1208.6282*
☑	63	*1210.6269*	*1209.6196*
☑	64	*1215.6644*	*1214.6571*
☑	65	*1217.6575*	*1216.6503*
☑	66	*1219.6486*	*1218.6413*
☑	67	*1225.6373*	*1224.6301*
☑	68	*1228.6796*	*1227.6723*
☑	69	*1234.6590*	*1233.6517*
☑	70	*1249.1486*	*1248.1413*
☑	71	*1250.6539*	*1249.6466*
☑	73	*1276.6050*	*1275.5977*
☑	74	*1280.6284*	*1279.6211*
☑	75	*1292.5783*	*1291.5710*
☑	76	*1296.6213*	*1295.6141*
☑	77	*1322.6371*	*1321.6298*
☑	78	*1618.8519*	*1617.8446*
☑	79	*1650.8407*	*1649.8335*
☑	80	*1872.7759*	*1871.7686*
☑	81	*1905.7288*	*1904.7215*
☑	82	*1962.7642*	*1961.7569*
☑	83	*1974.7638*	*1973.7565*
☑	84	*1994.0056*	*1992.9983*
☑	85	*2055.9612*	*2054.9540*
☑	86	*2148.9294*	*2147.9221*
☑	87	*2345.0400*	*2344.0328*
☑	88	*2359.0435*	*2358.0362*
☑	90	*2438.2368*	*2437.2295*
☑	91	*2454.2310*	*2453.2237*
☑	92	*2470.2291*	*2469.2218*
☑	93	*3346.7170*	*3345.7097*
☑	94	*3446.6176*	*3445.6103*

Search Parameters

```
Type of search         : MS/MS Ion Search
Enzyme                 : Trypsin
Fixed modifications    : Carbamidomethyl (C)
Variable modifications : Oxidation (M)
Mass values            : Monoisotopic
Protein Mass           : Unrestricted
Peptide Mass Tolerance : ± 100 ppm
Fragment Mass Tolerance: ± 0.7 Da
Max Missed Cleavages   : 1
Instrument type        : MALDI-TOF-TOF
Number of queries      : 94
```

Mascot: http://www.matrixscience.com/

Mascot Search Results

```
User         :
Email        :
Search title : vipera_all_Lift file:\\Proteomics1\d\data\2105\210505_tliepol_1_68252-68266\68252_68266_lpardo\0_G7\1\0_G7.xml
Database     : vipera  (1414 sequences; 347452 residues)
Timestamp    : 5 May 2021 at 09:50:14 GMT
Warning      : A Peptide summary report will usually give a much clearer picture of MS/MS search results.
Top Score    : 209 for DIS_MACLO, Disintegrin obtustatin OS=Macrovipera lebetina obtusa OX=209528 PE=1 SV=1
```

Mascot Score Histogram

Protein score is -10*Log(P), where P is the probability that the observed match is a random event.
Protein scores greater than 44 are significant (p<0.05).
Protein scores are derived from ions scores as a non-probabilistic basis for ranking protein hits.

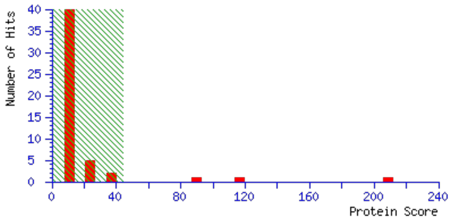

Protein Summary Report

Format As | Protein Summary (deprecated) | Help

Significance threshold p< 0.05 Max. number of hits 20

Overview Table

Click on column header to jump to entry in results list.
Move mouse over any indicator to highlight identical peptides.
Click on an indicator to see details of individual match.
Use check boxes to select sub-set of queries for new search.

Mouse over: -Query-
-Accession- -Sequence-

Hit:	1	2	3	4	5	6	7	8	9	10	11	12	13	14	15	16	17	18	19	20
☑ 855.1002 (1+)																				
☑ 863.3795 (1+)																				
☑ 868.5621 (1+)																				
☑ 871.0730 (1+)																				
☑ 875.3810 (1+)																				
☑ 877.3714 (1+)																				
☑ 895.3562 (1+)																				
☑ 897.3687 (1+)																				
☑ 903.3991 (1+)																				
☑ 905.3927 (1+)																				
☑ 907.3757 (1+)																				
☑ 909.3719 (1+)																				
☑ 920.4159 (1+)																				
☑ 921.3920 (1+)																				
☑ 925.3669 (1+)																				
☑ 935.3831 (1+)																				
☑ 937.3702 (1+)																				
☑ 947.3855 (1+)																				
☑ 952.3780 (1+)																				
☑ 953.3893 (1+)																				
☑ 954.3922 (1+)																				
☑ 966.3939 (1+)																				
☑ 968.3917 (1+)																				
☑ 977.3909 (1+)																				
☑ 979.3925 (1+)																				
☑ 980.4000 (1+)																				
☑ 982.3881 (1+)																				
☑ 984.3620 (1+)																				
☑ 986.3655 (1+)																				
☑ 992.4049 (1+)																				
☑ 993.4047 (1+)																				
☑ 994.3935 (1+)																				
☑ 996.4006 (1+)																				
☑ 998.3753 (1+)																				
☑ 1004.3794 (1+)																				
☑ 1006.3835 (1+)																				
☑ 1009.4004 (1+)																				
☑ 1011.4157 (1+)	●	●		●	●															
☑ 1022.1538 (1+)																				
☑ 1039.4114 (1+)																				
☑ 1043.3901 (1+)																				
☑ 1044.1374 (1+)																				
☑ 1066.1246 (1+)																				
☑ 1090.4345 (1+)																				

☑ 1106.3991 (1+)																				
☑ 1142.6361 (1+)																				
☑ 1159.6219 (1+)																				
☑ 1161.6411 (1+)	●	●	●																	
☑ 1163.4129 (1+)																				
☑ 1165.6247 (1+)																				
☑ 1172.6415 (1+)																				
☑ 1175.6433 (1+)																				
☑ 1177.6357 (1+)																				
☑ 1178.6413 (1+)																				
☑ 1183.6355 (1+)																				
☑ 1187.6553 (1+)																				
☑ 1193.6308 (1+)																				
☑ 1197.5917 (1+)																				
☑ 1200.6345 (1+)																				
☑ 1202.6678 (1+)																				
☑ 1206.6509 (1+)																				
☑ 1209.6355 (1+)																				
☑ 1210.6269 (1+)																				
☑ 1215.6644 (1+)																				
☑ 1217.6575 (1+)																				
☑ 1219.6486 (1+)																				
☑ 1225.6373 (1+)																				
☑ 1228.6796 (1+)																				
☑ 1234.6590 (1+)																				
☑ 1249.1486 (1+)																				
☑ 1250.6539 (1+)																				
☑ 1254.6129 (1+)	●			●	●					●										
☑ 1276.6050 (1+)																				
☑ 1280.6284 (1+)																				
☑ 1292.5783 (1+)																				
☑ 1296.6213 (1+)																				
☑ 1322.6371 (1+)																				
☑ 1618.8519 (1+)																				
☑ 1650.8407 (1+)																				
☑ 1872.7759 (1+)																				
☑ 1905.7288 (1+)																				
☑ 1962.7642 (1+)																				
☑ 1974.7638 (1+)																				
☑ 1994.0056 (1+)																				
☑ 2055.9612 (1+)																				
☑ 2148.9294 (1+)																				
☑ 2345.0400 (1+)																				
☑ 2359.0435 (1+)																				
☑ 2397.2085 (1+)	●																			
☑ 2438.2368 (1+)																				
☑ 2454.2310 (1+)																				
☑ 2470.2291 (1+)																				
☑ 3346.7170 (1+)																				
☑ 3446.6176 (1+)																				

Select All Select None Search Selected

Index

	Accession	Mass	Score	Description
1.	DIS_MACLO	4854	209	Disintegrin obtustatin OS=Macrovipera lebetina obtusa OX=209528 PE=1 SV=1
2.	DIS_DABPA	4914	110	Disintegrin viperistatin OS=Daboia palaestinae OX=1170828 PE=1 SV=1
3.	DIS_MACLB	12004	86	Disintegrin lebestatin OS=Macrovipera lebetina OX=8709 PE=1 SV=1
4.	Q1JRG7_MACLN	5041	34	RTS-containing short disintegrin ML-G3 (Fragment) OS=Macrovipera lebetina transmediterranea OX=384075 GN=ml-G3 PE=4 SV=1
5.	DIS_CERVI	5041	34	Disintegrin CV OS=Cerastes vipera OX=8698 PE=2 SV=1
6.	Q8UVG8_DABRR	52931	20	Vimentin OS=Daboia russelii OX=8707 PE=2 SV=1
7.	A0A6G5ZUS7_9SAUR	24311	20	Lactadherin 8 (Fragment) OS=Vipera anatolica senliki OX=2604287 PE=2 SV=1
8.	OXLA_DABRR	57251	19	L-amino-acid oxidase OS=Daboia russelii OX=8707 PE=1 SV=1
9.	A0A223PK64_DABRR	49512	18	Vascular endothelial growth factor C OS=Daboia russelii OX=8707 PE=2 SV=1
10.	A0A1S5QK15_VIPAA	10077	18	Kunitz/BPTI inhibitor-4 (Fragment) OS=Vipera ammodytes ammodytes OX=8705 PE=3 SV=1
11.	A0A223PK23_DABRR	24306	17	Endothelin 1 OS=Daboia russelii OX=8707 PE=2 SV=1
12.	Q6A371_VIPAM	16461	17	Ammodytin L(3) isoform OS=Vipera ammodytes montandoni OX=235554 PE=2 SV=1
13.	Q6A382_VIPAA	16431	17	Ammodytin L(1'') variant OS=Vipera ammodytes ammodytes OX=8705 PE=2 SV=1
14.	Q6A376_VIPAA	16455	17	Ammodytin L(1'') variant OS=Vipera ammodytes ammodytes OX=8705 PE=2 SV=1
15.	Q6A367_VIPAM	16403	17	Ammodytin L(3) variant OS=Vipera ammodytes montandoni OX=235554 PE=2 SV=1
16.	Q6A369_VIPAM	16449	17	Ammodytin L(3) variant OS=Vipera ammodytes montandoni OX=235554 PE=2 SV=1
17.	Q6A370_VIPAM	16461	17	Ammodytin L(3) variant OS=Vipera ammodytes montandoni OX=235554 PE=2 SV=1
18.	Q6A368_VIPAM	16489	17	Ammodytin L(3) variant OS=Vipera ammodytes montandoni OX=235554 PE=2 SV=1
19.	Q6A378_VIPAA	16459	17	Ammodytin L(1'') isoform OS=Vipera ammodytes ammodytes OX=8705 PE=2 SV=1
20.	Q6A387_VIPAM	16471	17	Ammodytin L(1) isoform OS=Vipera ammodytes ruffoi OX=246549 PE=2 SV=1

Results List

1. DIS_MACLO **Mass:** 4854 **Score:** 209 **Expect:** 1.8e-018 **Matches:** 4

Disintegrin obtustatin OS=Macrovipera lebetina obtusa OX=209528 PE=1 SV=1

Observed	Mr(expt)	Mr(calc)	ppm	Start		End	Miss	Ions	Peptide
1011.4157	1010.4084	1010.3732	34.8	1	-	8	0	15	-.CTTGPCCR.Q
1161.6411	1160.6339	1160.6012	28.1	12	-	21	0	68	K.LKPAGTTCWK.T
1254.6129	1253.6056	1253.5710	27.6	22	-	32	0	60	K.TSLTSHYCTGK.S
2397.2085	2396.2012	2396.1617	16.5	12	-	32	1	24	K.LKPAGTTCWKTSLTSHYCTGK.S

No match to: 855.1002, 863.3795, 868.5621, 871.0730, 875.3810, 877.3714, 895.3562, 897.3687, 903.3991, 905.3927, 907.3757, 909.3719, 920.4159, 921.3920, 925.3669, 935.3831, 937.3702, 947.3855, 952.3780, 953.3893, 954.3922, 966.3939, 968.3917, 977.3909, 979.3925, 980.4000, 982.3881, 984.3620, 986.3655, 992.4049, 993.4047, 994.3935, 996.4006, 998.3753, 1004.3794, 1006.3835, 1009.4004, 1022.1538, 1039.4114, 1043.3901, 1044.1374, 1066.1246, 1090.4345, 1106.3991, 1142.6361, 1159.6219, 1163.4129, 1165.6247, 1172.6415, 1175.6433, 1177.6357, 1178.6413, 1183.6355, 1187.6553, 1193.6308, 1197.5917, 1200.6345, 1202.6678, 1206.6509, 1209.6355, 1210.6269, 1215.6644, 1217.6575, 1219.6486, 1225.6373, 1228.6796, 1234.6590, 1249.1486, 1250.6539, 1276.6050, 1280.6284, 1292.5783, 1296.6213, 1322.6371, 1618.8519, 1650.8407, 1872.7759, 1905.7288, 1962.7642, 1974.7638, 1994.0056, 2055.9612, 2148.9294, 2345.0400, 2359.0435, 2438.2368, 2454.2310, 2470.2291, 3346.7170, 3446.6176

2. DIS_DABPA **Mass:** 4914 **Score:** 110 **Expect:** 1.4e-008 **Matches:** 3
Disintegrin viperistatin OS=Daboia palaestinae OX=1170828 PE=1 SV=1

Observed	Mr(expt)	Mr(calc)	ppm	Start	End	Miss	Ions	Peptide
953.3893	952.3820	952.4073	-26.48	25 -	32	0	---	R.TSHYCTGK.S
1011.4157	1010.4084	1010.3732	34.8	1 -	8	0	15	-.CTTGPCCR.Q
1161.6411	1160.6339	1160.6012	28.1	12 -	21	0	68	K.LKPAGTTCWK.T

No match to: 855.1002, 863.3795, 868.5621, 871.0730, 875.3810, 877.3714, 895.3562, 897.3687, 903.3991, 905.3927, 907.3757, 909.3719, 920.4159, 921.3920, 925.3669, 935.3831, 937.3702, 947.3855, 952.3780, 954.3922, 966.3939, 968.3917, 977.3909, 979.3925, 980.4000, 982.3881, 984.3620, 986.3655, 992.4049, 993.4047, 994.3935, 996.4006, 998.3753, 1004.3794, 1006.3835, 1009.4004, 1022.1538, 1039.4114, 1043.3901, 1044.1374, 1066.1246, 1090.4345, 1106.3991, 1142.6361, 1159.6219, 1163.4129, 1165.6247, 1172.6415, 1175.6433, 1177.6357, 1178.6413, 1183.6355, 1187.6553, 1193.6308, 1197.5917, 1200.6345, 1202.6678, 1206.6509, 1209.6355, 1210.6269, 1215.6644, 1217.6575, 1219.6486, 1225.6373, 1228.6796, 1234.6590, 1249.1486, 1250.6539, 1254.6129, 1276.6050, 1280.6284, 1292.5783, 1296.6213, 1322.6371, 1618.8519, 1650.8407, 1872.7759, 1905.7288, 1962.7642, 1974.7638, 1994.0056, 2055.9612, 2148.9294, 2345.0400, 2359.0435, 2397.2085, 2438.2368, 2454.2310, 2470.2291, 3346.7170, 3446.6176

3. DIS_MACLB **Mass:** 12004 **Score:** 86 **Expect:** 3.8e-006 **Matches:** 3
Disintegrin lebestatin OS=Macrovipera lebetina OX=8709 PE=1 SV=1

Observed	Mr(expt)	Mr(calc)	ppm	Start	End	Miss	Ions	Peptide
953.3893	952.3820	952.4073	-26.48	89 -	96	0	---	R.TSHYCTGK.S
1161.6411	1160.6339	1160.6012	28.1	76 -	85	0	68	K.LKPAGTTCWK.T
1254.6129	1253.6056	1253.6074	-1.41	45 -	55	1	---	K.GAVEEKHEPMK.G

No match to: 855.1002, 863.3795, 868.5621, 871.0730, 875.3810, 877.3714, 895.3562, 897.3687, 903.3991, 905.3927, 907.3757, 909.3719, 920.4159, 921.3920, 925.3669, 935.3831, 937.3702, 947.3855, 952.3780, 954.3922, 966.3939, 968.3917, 977.3909, 979.3925, 980.4000, 982.3881, 984.3620, 986.3655, 992.4049, 993.4047, 994.3935, 996.4006, 998.3753, 1004.3794, 1006.3835, 1009.4004, 1011.4157, 1022.1538, 1039.4114, 1043.3901, 1044.1374, 1066.1246, 1090.4345, 1106.3991, 1142.6361, 1159.6219, 1163.4129, 1165.6247, 1172.6415, 1175.6433, 1177.6357, 1178.6413, 1183.6355, 1187.6553, 1193.6308, 1197.5917, 1200.6345, 1202.6678, 1206.6509, 1209.6355, 1210.6269, 1215.6644, 1217.6575, 1219.6486, 1225.6373, 1228.6796, 1234.6590, 1249.1486, 1250.6539, 1276.6050, 1280.6284, 1292.5783, 1296.6213, 1322.6371, 1618.8519, 1650.8407, 1872.7759, 1905.7288, 1962.7642, 1974.7638, 1994.0056, 2055.9612, 2148.9294, 2345.0400, 2359.0435, 2397.2085, 2438.2368, 2454.2310, 2470.2291, 3346.7170, 3446.6176

4. Q1JRG7_MACLN **Mass:** 5041 **Score:** 34 **Expect:** 0.5 **Matches:** 2
RTS-containing short disintegrin ML-G3 (Fragment) OS=Macrovipera lebetina transmediterranea OX=384075 GN=ml-G3 PE=4 SV=1

Observed	Mr(expt)	Mr(calc)	ppm	Start	End	Miss	Ions	Peptide
1011.4157	1010.4084	1010.3732	34.8	1 -	8	0	15	-.CTTGPCCR.Q
1254.6129	1253.6056	1253.5459	47.6	22 -	32	0	4	R.TSVSSHYCTGR.S

No match to: 855.1002, 863.3795, 868.5621, 871.0730, 875.3810, 877.3714, 895.3562, 897.3687, 903.3991, 905.3927, 907.3757, 909.3719, 920.4159, 921.3920, 925.3669, 935.3831, 937.3702, 947.3855, 952.3780, 953.3893, 954.3922, 966.3939, 968.3917, 977.3909, 979.3925, 980.4000, 982.3881, 984.3620, 986.3655, 992.4049, 993.4047, 994.3935, 996.4006, 998.3753, 1004.3794, 1006.3835, 1009.4004, 1022.1538, 1039.4114, 1043.3901, 1044.1374, 1066.1246, 1090.4345, 1106.3991, 1142.6361, 1159.6219, 1161.6411, 1163.4129, 1165.6247, 1172.6415, 1175.6433, 1177.6357, 1178.6413, 1183.6355, 1187.6553, 1193.6308, 1197.5917, 1200.6345, 1202.6678, 1206.6509, 1209.6355, 1210.6269, 1215.6644, 1217.6575, 1219.6486, 1225.6373, 1228.6796, 1234.6590, 1249.1486, 1250.6539, 1276.6050, 1280.6284, 1292.5783, 1296.6213, 1322.6371, 1618.8519, 1650.8407, 1872.7759, 1905.7288, 1962.7642, 1974.7638, 1994.0056, 2055.9612, 2148.9294, 2345.0400, 2359.0435, 2397.2085, 2438.2368, 2454.2310, 2470.2291, 3346.7170, 3446.6176

5. DIS_CERVI **Mass:** 5041 **Score:** 34 **Expect:** 0.5 **Matches:** 2
Disintegrin CV OS=Cerastes vipera OX=8698 PE=2 SV=1

Observed	Mr(expt)	Mr(calc)	ppm	Start	End	Miss	Ions	Peptide
1011.4157	1010.4084	1010.3732	34.8	1 -	8	0	15	-.CTTGPCCR.Q
1254.6129	1253.6056	1253.5459	47.6	22 -	32	0	4	R.TSVSSHYCTGR.S

No match to: 855.1002, 863.3795, 868.5621, 871.0730, 875.3810, 877.3714, 895.3562, 897.3687, 903.3991, 905.3927, 907.3757, 909.3719, 920.4159, 921.3920, 925.3669, 935.3831, 937.3702, 947.3855, 952.3780, 953.3893, 954.3922, 966.3939, 968.3917, 977.3909, 979.3925, 980.4000, 982.3881, 984.3620, 986.3655, 992.4049, 993.4047, 994.3935, 996.4006, 998.3753, 1004.3794, 1006.3835, 1009.4004, 1022.1538, 1039.4114, 1043.3901, 1044.1374, 1066.1246, 1090.4345, 1106.3991, 1142.6361, 1159.6219, 1161.6411, 1163.4129, 1165.6247, 1172.6415, 1175.6433, 1177.6357, 1178.6413, 1183.6355, 1187.6553, 1193.6308, 1197.5917, 1200.6345, 1202.6678, 1206.6509, 1209.6355, 1210.6269, 1215.6644, 1217.6575, 1219.6486, 1225.6373, 1228.6796, 1234.6590, 1249.1486, 1250.6539, 1276.6050, 1280.6284, 1292.5783, 1296.6213, 1322.6371, 1618.8519, 1650.8407, 1872.7759, 1905.7288, 1962.7642, 1974.7638, 1994.0056, 2055.9612, 2148.9294, 2345.0400, 2359.0435, 2397.2085, 2438.2368, 2454.2310, 2470.2291, 3346.7170, 3446.6176

6. Q8UVG8_DABRR **Mass:** 52931 **Score:** 20 **Expect:** 15 **Matches:** 6
Vimentin OS=Daboia russelii OX=8707 PE=2 SV=1

Observed	Mr(expt)	Mr(calc)	ppm	Start	End	Miss	Ions	Peptide
905.3927	904.3854	904.4250	-43.76	330 -	337	0	---	K.GTNESLER.Q
1161.6411	1160.6339	1160.6302	3.16	116 -	124	1	---	K.VRFLEQQNK.I
1209.6355	1208.6282	1208.5785	41.1	306 -	315	1	---	R.QAKQEASEYR.R
1254.6129	1253.6056	1253.5598	36.6	141 -	150	0	---	R.LGDIYEEEMR.E
2454.2310	2453.2237	2453.3278	-42.41	398 -	419	1	---	K.LLEGEESRINVPVPTFASLNLR.E
3446.6176	3445.6103	3445.7623	-44.13	239 -	268	1	---	R.ELQAQIQDQHIQIDMDVAKPDLTAALRDVR.Q + Oxidation (M)

No match to: 855.1002, 863.3795, 868.5621, 871.0730, 875.3810, 877.3714, 895.3562, 897.3687, 903.3991, 907.3757, 909.3719, 920.4159, 921.3920, 925.3669, 935.3831, 937.3702, 947.3855, 952.3780, 953.3893, 954.3922, 966.3939, 968.3917, 977.3909, 979.3925, 980.4000, 982.3881, 984.3620, 986.3655, 992.4049, 993.4047, 994.3935, 996.4006, 998.3753, 1004.3794, 1006.3835, 1009.4004, 1011.4157, 1022.1538, 1039.4114, 1043.3901, 1044.1374, 1066.1246, 1090.4345, 1106.3991, 1142.6361, 1159.6219, 1163.4129, 1165.6247, 1172.6415, 1175.6433, 1177.6357, 1178.6413, 1183.6355, 1187.6553, 1193.6308, 1197.5917, 1200.6345, 1202.6678, 1206.6509, 1210.6269, 1215.6644, 1217.6575, 1219.6486, 1225.6373, 1228.6796, 1234.6590, 1249.1486, 1250.6539, 1276.6050, 1280.6284, 1292.5783, 1296.6213, 1322.6371, 1618.8519, 1650.8407, 1872.7759, 1905.7288, 1962.7642, 1974.7638, 1994.0056, 2055.9612, 2148.9294, 2345.0400, 2359.0435, 2397.2085, 2438.2368, 2470.2291, 3346.7170

7. A0A6G5ZUS7_9SAUR **Mass:** 24311 **Score:** 20 **Expect:** 15 **Matches:** 6
Lactadherin 8 (Fragment) OS=Vipera anatolica senliki OX=2604287 PE=2 SV=1

Observed	Mr(expt)	Mr(calc)	ppm	Start	End	Miss	Ions	Peptide
1009.4004	1008.3931	1008.4876	-93.73	1 -	9	0	---	-.QIFSGNTDK.Y
1618.8519	1617.8446	1617.9103	-40.63	190 -	202	1	---	R.ILPEAWHNRITLR.V
1974.7638	1973.7565	1973.9119	-78.74	142 -	158	1	---	K.VAYSNDGKSWTVYQDNK.T
2438.2368	2437.2295	2437.0647	67.6	41 -	61	1	---	R.FELIGCEMNGCSDPLGMKSHR.I
2454.2310	2453.2237	2453.0596	66.9	41 -	61	1	---	R.FELIGCEMNGCSDPLGMKSHR.I + Oxidation (M)
2470.2291	2469.2218	2469.0545	67.8	41 -	61	1	---	R.FELIGCEMNGCSDPLGMKSHR.I + 2 Oxidation (M)

No match to: 855.1002, 863.3795, 868.5621, 871.0730, 875.3810, 877.3714, 895.3562, 897.3687, 903.3991, 905.3927, 907.3757, 909.3719, 920.4159, 921.3920, 925.3669, 935.3831, 937.3702, 947.3855, 952.3780, 953.3893, 954.3922, 966.3939, 968.3917, 977.3909, 979.3925, 980.4000, 982.3881, 984.3620, 986.3655, 992.4049, 993.4047, 994.3935, 996.4006, 998.3753, 1004.3794, 1006.3835, 1011.4157, 1022.1538, 1039.4114, 1043.3901, 1044.1374, 1066.1246, 1090.4345, 1106.3991, 1142.6361, 1159.6219, 1161.6411, 1163.4129, 1165.6247, 1172.6415, 1175.6433, 1177.6357, 1178.6413, 1183.6355, 1187.6553, 1193.6308, 1197.5917, 1200.6345, 1202.6678, 1206.6509, 1209.6355, 1210.6269, 1215.6644, 1217.6575, 1219.6486, 1225.6373, 1228.6796, 1234.6590, 1249.1486, 1250.6539, 1254.6129, 1276.6050, 1280.6284, 1292.5783, 1296.6213, 1322.6371, 1650.8407, 1872.7759, 1905.7288, 1962.7642, 1994.0056, 2055.9612, 2148.9294, 2345.0400, 2359.0435, 2397.2085, 3346.7170, 3446.6176

8. OXLA_DABRR **Mass:** 57251 **Score:** 19 **Expect:** 20 **Matches:** 7
L-amino-acid oxidase OS=Daboia russelii OX=8707 PE=1 SV=1

Observed	Mr(expt)	Mr(calc)	ppm	Start	End	Miss	Ions	Peptide
877.3714	876.3641	876.3865	-25.51	198 -	204	0	---	K.YDTYSTK.E
903.3991	902.3918	902.4457	-59.76	497 -	504	1	---	R.DVNRASEL.-
1159.6219	1158.6146	1158.6105	3.53	490 -	500	1	---	K.SGLTAARDVNR.A
1209.6355	1208.6282	1208.6526	-20.22	331 -	340	1	---	K.AHALRSVHYR.S
1280.6284	1279.6211	1279.6408	-15.37	170 -	181	0	---	K.SAGQLYQESLGK.A
1650.8407	1649.8335	1649.7984	21.3	95 -	108	1	---	R.NVKEGWYANLGPMR.V + Oxidation (M)
1974.7638	1973.7565	1973.8863	-65.77	251 -	267	0	---	R.FDEIVGGMDQLPTSMYR.A + Oxidation (M)

No match to: 855.1002, 863.3795, 868.5621, 871.0730, 875.3810, 895.3562, 897.3687, 905.3927, 907.3757, 909.3719, 920.4159, 921.3920, 925.3669, 935.3831, 937.3702, 947.3855, 952.3780, 953.3893, 954.3922, 966.3939, 968.3917, 977.3909, 979.3925, 980.4000, 982.3881, 984.3620, 986.3655, 992.4049, 993.4047, 994.3935, 996.4006, 998.3753, 1004.3794, 1006.3835, 1009.4004, 1011.4157, 1022.1538, 1039.4114, 1043.3901, 1044.1374, 1066.1246, 1090.4345, 1106.3991, 1142.6361, 1161.6411, 1163.4129, 1165.6247, 1172.6415, 1175.6433, 1177.6357, 1178.6413, 1183.6355, 1187.6553, 1193.6308, 1197.5917, 1200.6345, 1202.6678, 1206.6509, 1210.6269, 1215.6644, 1217.6575, 1219.6486, 1225.6373, 1228.6796, 1234.6590, 1249.1486, 1250.6539, 1254.6129, 1276.6050, 1292.5783, 1296.6213, 1322.6371, 1618.8519, 1872.7759, 1905.7288, 1962.7642, 1994.0056, 2055.9612, 2148.9294, 2345.0400, 2359.0435, 2397.2085, 2438.2368, 2454.2310, 2470.2291, 3346.7170, 3446.6176

9. A0A223PK64_DABRR **Mass:** 49512 **Score:** 18 **Expect:** 24 **Matches:** 6

Vascular endothelial growth factor C OS=Daboia russelii OX=8707 PE=2 SV=1

Observed	Mr(expt)	Mr(calc)	ppm	Start	End	Miss	Ions	Peptide
905.3927	904.3854	904.4324	-51.97	136 -	143	0	---	R.EVCVDVGK.E
909.3719	908.3646	908.4538	-98.19	371 -	377	1	---	K.CFLKGER.F
920.4159	919.4086	919.4368	-30.66	129 -	135	1	---	K.KTQCMPR.E
952.3780	951.3707	951.4046	-35.66	94 -	101	0	---	R.EQSNFDGR.T
953.3893	952.3820	952.4477	-68.91	410 -	416	0	---	R.CVPTYWK.L
1187.6553	1186.6480	1186.5475	84.7	212 -	220	1	---	R.CMSKLDVYR.Q + Oxidation (M)

No match to: 855.1002, 863.3795, 868.5621, 871.0730, 875.3810, 877.3714, 895.3562, 897.3687, 903.3991, 907.3757, 921.3920, 925.3669, 935.3831, 937.3702, 947.3855, 954.3922, 966.3939, 968.3917, 977.3909, 979.3925, 980.4000, 982.3881, 984.3620, 986.3655, 992.4049, 993.4047, 994.3935, 996.4006, 998.3753, 1004.3794, 1006.3835, 1009.4004, 1011.4157, 1022.1538, 1039.4114, 1043.3901, 1044.1374, 1066.1246, 1090.4345, 1106.3991, 1142.6361, 1159.6219, 1161.6411, 1163.4129, 1165.6247, 1172.6415, 1175.6433, 1177.6357, 1178.6413, 1183.6355, 1193.6308, 1197.5917, 1200.6345, 1202.6678, 1206.6509, 1209.6355, 1210.6269, 1215.6644, 1217.6575, 1219.6486, 1225.6373, 1228.6796, 1234.6590, 1249.1486, 1250.6539, 1254.6129, 1276.6050, 1280.6284, 1292.5783, 1296.6213, 1322.6371, 1618.8519, 1650.8407, 1872.7759, 1905.7288, 1962.7642, 1974.7638, 1994.0056, 2055.9612, 2148.9294, 2345.0400, 2359.0435, 2397.2085, 2438.2368, 2454.2310, 2470.2291, 3346.7170, 3446.6176

10. A0A1S5QK15_VIPAA **Mass:** 10077 **Score:** 18 **Expect:** 25 **Matches:** 2

Kunitz/BPTI inhibitor-4 (Fragment) OS=Vipera ammodytes ammodytes OX=8705 PE=3 SV=1

Observed	Mr(expt)	Mr(calc)	ppm	Start	End	Miss	Ions	Peptide
1254.6129	1253.6056	1253.6187	-10.40	79 -	90	1	5	R.HTCVASGKGIQP.-
1872.7759	1871.7686	1871.8043	-19.05	56 -	72	1	---	K.CKQFSFGGCPGNANNSK.T

No match to: 855.1002, 863.3795, 868.5621, 871.0730, 875.3810, 877.3714, 895.3562, 897.3687, 903.3991, 905.3927, 907.3757, 909.3719, 920.4159, 921.3920, 925.3669, 935.3831, 937.3702, 947.3855, 952.3780, 953.3893, 954.3922, 966.3939, 968.3917, 977.3909, 979.3925, 980.4000, 982.3881, 984.3620, 986.3655, 992.4049, 993.4047, 994.3935, 996.4006, 998.3753, 1004.3794, 1006.3835, 1009.4004, 1011.4157, 1022.1538, 1039.4114, 1043.3901, 1044.1374, 1066.1246, 1090.4345, 1106.3991, 1142.6361, 1159.6219, 1161.6411, 1163.4129, 1165.6247, 1172.6415, 1175.6433, 1177.6357, 1178.6413, 1183.6355, 1187.6553, 1193.6308, 1197.5917, 1200.6345, 1202.6678, 1206.6509, 1209.6355, 1210.6269, 1215.6644, 1217.6575, 1219.6486, 1225.6373, 1228.6796, 1234.6590, 1249.1486, 1250.6539, 1276.6050, 1280.6284, 1292.5783, 1296.6213, 1322.6371, 1618.8519, 1650.8407, 1905.7288, 1962.7642, 1974.7638, 1994.0056, 2055.9612, 2148.9294, 2345.0400, 2359.0435, 2397.2085, 2438.2368, 2454.2310, 2470.2291, 3346.7170, 3446.6176

11. A0A223PK23_DABRR **Mass:** 24306 **Score:** 17 **Expect:** 27 **Matches:** 4

Endothelin 1 OS=Daboia russelii OX=8707 PE=2 SV=1

Observed	Mr(expt)	Mr(calc)	ppm	Start	End	Miss	Ions	Peptide
875.3810	874.3737	874.3789	-5.98	101 -	107	0	---	R.CHCASLK.D
947.3855	946.3782	946.4621	-88.58	189 -	195	1	---	K.HNRTYEK.Q
1161.6411	1160.6339	1160.6554	-18.52	71 -	81	0	---	R.IVPYGLGSLSR.T
1163.4129	1162.4057	1162.4457	-34.44	44 -	52	0	---	R.CSCYSLMDK.E

No match to: 855.1002, 863.3795, 868.5621, 871.0730, 877.3714, 895.3562, 897.3687, 903.3991, 905.3927, 907.3757, 909.3719, 920.4159, 921.3920, 925.3669, 935.3831, 937.3702, 952.3780, 953.3893, 954.3922, 966.3939, 968.3917, 977.3909, 979.3925, 980.4000, 982.3881, 984.3620, 986.3655, 992.4049, 993.4047, 994.3935, 996.4006, 998.3753, 1004.3794, 1006.3835, 1009.4004, 1011.4157, 1022.1538, 1039.4114, 1043.3901, 1044.1374, 1066.1246, 1090.4345, 1106.3991, 1142.6361, 1159.6219, 1165.6247, 1172.6415, 1175.6433, 1177.6357, 1178.6413, 1183.6355, 1187.6553, 1193.6308, 1197.5917, 1200.6345, 1202.6678, 1206.6509, 1209.6355, 1210.6269, 1215.6644, 1217.6575, 1219.6486, 1225.6373, 1228.6796, 1234.6590, 1249.1486, 1250.6539, 1254.6129, 1276.6050, 1280.6284, 1292.5783, 1296.6213, 1322.6371, 1618.8519, 1650.8407, 1872.7759, 1905.7288, 1962.7642, 1974.7638, 1994.0056, 2055.9612, 2148.9294, 2345.0400, 2359.0435, 2397.2085, 2438.2368, 2454.2310, 2470.2291, 3346.7170, 3446.6176

12. Q6A371_VIPAM **Mass:** 16461 **Score:** 17 **Expect:** 28 **Matches:** 5

Ammodytin L(3) isoform OS=Vipera ammodytes montandoni OX=235554 PE=2 SV=1

Observed	Mr(expt)	Mr(calc)	ppm	Start	End	Miss	Ions	Peptide
980.4000	979.3927	979.3851	7.72	100 -	106	0	---	K.QICECDR.A
993.4047	992.3975	992.4484	-51.38	24 -	31	0	---	K.MIQEETDK.N
1009.4004	1008.3931	1008.4434	-49.84	24 -	31	0	---	K.MIQEETDK.N + Oxidation (M)
1177.6357	1176.6285	1176.5557	61.8	70 -	79	1	---	K.LSDCSPKTNR.Y
1292.5783	1291.5710	1291.6707	-77.15	107 -	117	1	---	R.AAAICFRENLK.T

No match to: 855.1002, 863.3795, 868.5621, 871.0730, 875.3810, 877.3714, 895.3562, 897.3687, 903.3991, 905.3927, 907.3757, 909.3719, 920.4159, 921.3920, 925.3669, 935.3831, 937.3702, 947.3855, 952.3780, 953.3893, 954.3922, 966.3939, 968.3917, 977.3909, 979.3925, 982.3881, 984.3620, 986.3655, 992.4049, 994.3935, 996.4006, 998.3753, 1004.3794, 1006.3835, 1011.4157, 1022.1538, 1039.4114, 1043.3901, 1044.1374, 1066.1246, 1090.4345, 1106.3991, 1142.6361, 1159.6219, 1161.6411, 1163.4129, 1165.6247, 1172.6415, 1175.6433, 1178.6413, 1183.6355, 1187.6553, 1193.6308, 1197.5917, 1200.6345, 1202.6678, 1206.6509, 1209.6355, 1210.6269, 1215.6644, 1217.6575, 1219.6486, 1225.6373, 1228.6796, 1234.6590, 1249.1486, 1250.6539, 1254.6129, 1276.6050, 1280.6284, 1296.6213, 1322.6371, 1618.8519, 1650.8407, 1872.7759, 1905.7288, 1962.7642, 1974.7638, 1994.0056, 2055.9612, 2148.9294, 2345.0400, 2359.0435, 2397.2085, 2438.2368, 2454.2310, 2470.2291, 3346.7170, 3446.6176

13. Q6A382_VIPAA **Mass:** 16431 **Score:** 17 **Expect:** 28 **Matches:** 5

Ammodytin L(1'') variant OS=Vipera ammodytes ammodytes OX=8705 PE=2 SV=1

Observed	Mr(expt)	Mr(calc)	ppm	Start	End	Miss	Ions	Peptide
980.4000	979.3927	979.3851	7.72	100 -	106	0	---	K.QICECDR.A
993.4047	992.3975	992.4484	-51.38	24 -	31	0	---	K.MIQEETDK.N
1009.4004	1008.3931	1008.4434	-49.84	24 -	31	0	---	K.MIQEETDK.N + Oxidation (M)
1177.6357	1176.6285	1176.5557	61.8	70 -	79	1	---	K.LSDCSPKTNR.Y
1292.5783	1291.5710	1291.6707	-77.15	107 -	117	1	---	R.AAAICFRENLK.T

No match to: 855.1002, 863.3795, 868.5621, 871.0730, 875.3810, 877.3714, 895.3562, 897.3687, 903.3991, 905.3927, 907.3757, 909.3719, 920.4159, 921.3920, 925.3669, 935.3831, 937.3702, 947.3855, 952.3780, 953.3893, 954.3922, 966.3939, 968.3917, 977.3909, 979.3925, 982.3881, 984.3620, 986.3655, 992.4049, 994.3935, 996.4006, 998.3753, 1004.3794, 1006.3835, 1011.4157, 1022.1538, 1039.4114, 1043.3901, 1044.1374, 1066.1246, 1090.4345, 1106.3991, 1142.6361, 1159.6219, 1161.6411, 1163.4129, 1165.6247, 1172.6415, 1175.6433, 1178.6413, 1183.6355, 1187.6553, 1193.6308, 1197.5917, 1200.6345, 1202.6678, 1206.6509, 1209.6355, 1210.6269, 1215.6644, 1217.6575, 1219.6486, 1225.6373, 1228.6796, 1234.6590, 1249.1486, 1250.6539, 1254.6129, 1276.6050, 1280.6284, 1296.6213, 1322.6371, 1618.8519, 1650.8407, 1872.7759, 1905.7288, 1962.7642, 1974.7638, 1994.0056, 2055.9612, 2148.9294, 2345.0400, 2359.0435, 2397.2085, 2438.2368, 2454.2310, 2470.2291, 3346.7170, 3446.6176

14. Q6A376_VIPAA **Mass:** 16455 **Score:** 17 **Expect:** 28 **Matches:** 5

Ammodytin L(1'') variant OS=Vipera ammodytes ammodytes OX=8705 PE=2 SV=1

Observed	Mr(expt)	Mr(calc)	ppm	Start	End	Miss	Ions	Peptide
980.4000	979.3927	979.3851	7.72	100 -	106	0	---	K.QICECDR.A
993.4047	992.3975	992.4484	-51.38	24 -	31	0	---	K.MIQEETDK.N
1009.4004	1008.3931	1008.4434	-49.84	24 -	31	0	---	K.MIQEETDK.N + Oxidation (M)
1177.6357	1176.6285	1176.5557	61.8	70 -	79	1	---	K.LSDCSPKTNR.Y
1292.5783	1291.5710	1291.6707	-77.15	107 -	117	1	---	R.AAAICFRENLK.T

No match to: 855.1002, 863.3795, 868.5621, 871.0730, 875.3810, 877.3714, 895.3562, 897.3687, 903.3991, 905.3927, 907.3757, 909.3719, 920.4159, 921.3920, 925.3669, 935.3831, 937.3702, 947.3855, 952.3780, 953.3893, 954.3922, 966.3939, 968.3917, 977.3909, 979.3925, 982.3881, 984.3620, 986.3655, 992.4049, 994.3935, 996.4006, 998.3753, 1004.3794, 1006.3835, 1011.4157, 1022.1538, 1039.4114, 1043.3901, 1044.1374, 1066.1246, 1090.4345, 1106.3991, 1142.6361, 1159.6219, 1161.6411, 1163.4129, 1165.6247, 1172.6415, 1175.6433, 1178.6413, 1183.6355, 1187.6553, 1193.6308, 1197.5917, 1200.6345, 1202.6678, 1206.6509, 1209.6355, 1210.6269, 1215.6644, 1217.6575, 1219.6486, 1225.6373, 1228.6796, 1234.6590, 1249.1486, 1250.6539, 1254.6129, 1276.6050, 1280.6284, 1296.6213, 1322.6371, 1618.8519, 1650.8407, 1872.7759, 1905.7288, 1962.7642, 1974.7638, 1994.0056, 2055.9612, 2148.9294, 2345.0400, 2359.0435, 2397.2085, 2438.2368, 2454.2310, 2470.2291, 3346.7170, 3446.6176

15. Q6A367_VIPAM **Mass:** 16403 **Score:** 17 **Expect:** 28 **Matches:** 5

Ammodytin L(3) variant OS=Vipera ammodytes montandoni OX=235554 PE=2 SV=1

Observed	Mr(expt)	Mr(calc)	ppm	Start	End	Miss	Ions	Peptide
980.4000	979.3927	979.3851	7.72	100 -	106	0	---	K.QICECDR.A
993.4047	992.3975	992.4484	-51.38	24 -	31	0	---	K.MIQEETDK.N
1009.4004	1008.3931	1008.4434	-49.84	24 -	31	0	---	K.MIQEETDK.N + Oxidation (M)
1177.6357	1176.6285	1176.5557	61.8	70 -	79	1	---	K.LSDCSPKTNR.Y
1292.5783	1291.5710	1291.6707	-77.15	107 -	117	1	---	R.AAAICFRENLK.T

No match to: 855.1002, 863.3795, 868.5621, 871.0730, 875.3810, 877.3714, 895.3562, 897.3687, 903.3991, 905.3927, 907.3757, 909.3719, 920.4159, 921.3920, 925.3669, 935.3831, 937.3702, 947.3855, 952.3780, 953.3893, 954.3922, 966.3939, 968.3917, 977.3909, 979.3925, 982.3881, 984.3620, 986.3655, 992.4049, 994.3935, 996.4006, 998.3753, 1004.3794, 1006.3835, 1011.4157, 1022.1538, 1039.4114, 1043.3901, 1044.1374, 1066.1246, 1090.4345, 1106.3991, 1142.6361, 1159.6219, 1161.6411, 1163.4129, 1165.6247, 1172.6415, 1175.6433, 1178.6413, 1183.6355, 1187.6553, 1193.6308, 1197.5917, 1200.6345, 1202.6678, 1206.6509, 1209.6355, 1210.6269, 1215.6644, 1217.6575, 1219.6486, 1225.6373, 1228.6796, 1234.6590, 1249.1486, 1250.6539, 1254.6129, 1276.6050, 1280.6284, 1296.6213, 1322.6371, 1618.8519, 1650.8407, 1872.7759, 1905.7288, 1962.7642, 1974.7638, 1994.0056, 2055.9612, 2148.9294, 2345.0400, 2359.0435, 2397.2085, 2438.2368, 2454.2310, 2470.2291, 3346.7170, 3446.6176

16. Q6A369_VIPAM **Mass:** 16449 **Score:** 17 **Expect:** 28 **Matches:** 5

Ammodytin L(3) variant OS=Vipera ammodytes montandoni OX=235554 PE=2 SV=1

Observed	Mr(expt)	Mr(calc)	ppm	Start	End	Miss	Ions	Peptide
980.4000	979.3927	979.3851	7.72	100 -	106	0	---	K.QICECDR.A
993.4047	992.3975	992.4484	-51.38	24 -	31	0	---	K.MIQEETDK.N
1009.4004	1008.3931	1008.4434	-49.84	24 -	31	0	---	K.MIQEETDK.N + Oxidation (M)
1177.6357	1176.6285	1176.5557	61.8	70 -	79	1	---	K.LSDCSPKTNR.Y
1292.5783	1291.5710	1291.6707	-77.15	107 -	117	1	---	R.AAAICFRENLK.T

No match to: 855.1002, 863.3795, 868.5621, 871.0730, 875.3810, 877.3714, 895.3562, 897.3687, 903.3991, 905.3927, 907.3757, 909.3719, 920.4159, 921.3920, 925.3669, 935.3831, 937.3702, 947.3855, 952.3780, 953.3893, 954.3922, 966.3939, 968.3917, 977.3909, 979.3925, 982.3881, 984.3620, 986.3655, 992.4049, 994.3935, 996.4006, 998.3753, 1004.3794, 1006.3835, 1011.4157, 1022.1538, 1039.4114, 1043.3901, 1044.1374, 1066.1246, 1090.4345, 1106.3991, 1142.6361, 1159.6219, 1161.6411, 1163.4129, 1165.6247, 1172.6415, 1175.6433, 1178.6413, 1183.6355, 1187.6553, 1193.6308, 1197.5917, 1200.6345, 1202.6678, 1206.6509, 1209.6355, 1210.6269, 1215.6644, 1217.6575, 1219.6486, 1225.6373, 1228.6796, 1234.6590, 1249.1486, 1250.6539, 1254.6129, 1276.6050, 1280.6284, 1296.6213, 1322.6371, 1618.8519, 1650.8407, 1872.7759, 1905.7288, 1962.7642, 1974.7638, 1994.0056, 2055.9612, 2148.9294, 2345.0400, 2359.0435, 2397.2085, 2438.2368, 2454.2310, 2470.2291, 3346.7170, 3446.6176

17. Q6A370_VIPAM **Mass:** 16461 **Score:** 17 **Expect:** 28 **Matches:** 5

Ammodytin L(3) variant OS=Vipera ammodytes montandoni OX=235554 PE=2 SV=1

Observed	Mr(expt)	Mr(calc)	ppm	Start	End	Miss	Ions	Peptide
980.4000	979.3927	979.3851	7.72	100 -	106	0	---	K.QICECDR.A
993.4047	992.3975	992.4484	-51.38	24 -	31	0	---	K.MIQEETDK.N
1009.4004	1008.3931	1008.4434	-49.84	24 -	31	0	---	K.MIQEETDK.N + Oxidation (M)
1177.6357	1176.6285	1176.5557	61.8	70 -	79	1	---	K.LSDCSPKTNR.Y
1292.5783	1291.5710	1291.6707	-77.15	107 -	117	1	---	R.AAAICFRENLK.T

No match to: 855.1002, 863.3795, 868.5621, 871.0730, 875.3810, 877.3714, 895.3562, 897.3687, 903.3991, 905.3927, 907.3757, 909.3719, 920.4159, 921.3920, 925.3669, 935.3831, 937.3702, 947.3855, 952.3780, 953.3893, 954.3922, 966.3939, 968.3917, 977.3909, 979.3925, 982.3881, 984.3620, 986.3655, 992.4049, 994.3935, 996.4006, 998.3753, 1004.3794, 1006.3835, 1011.4157, 1022.1538, 1039.4114, 1043.3901, 1044.1374, 1066.1246, 1090.4345, 1106.3991, 1142.6361, 1159.6219, 1161.6411, 1163.4129, 1165.6247, 1172.6415, 1175.6433, 1178.6413, 1183.6355, 1187.6553, 1193.6308, 1197.5917, 1200.6345, 1202.6678, 1206.6509, 1209.6355, 1210.6269, 1215.6644, 1217.6575, 1219.6486, 1225.6373, 1228.6796, 1234.6590, 1249.1486, 1250.6539, 1254.6129, 1276.6050, 1280.6284, 1296.6213, 1322.6371, 1618.8519, 1650.8407, 1872.7759, 1905.7288, 1962.7642, 1974.7638, 1994.0056, 2055.9612, 2148.9294, 2345.0400, 2359.0435, 2397.2085, 2438.2368, 2454.2310, 2470.2291, 3346.7170, 3446.6176

18. Q6A368_VIPAM **Mass:** 16489 **Score:** 17 **Expect:** 28 **Matches:** 5

Ammodytin L(3) variant OS=Vipera ammodytes montandoni OX=235554 PE=2 SV=1

Observed	Mr(expt)	Mr(calc)	ppm	Start	End	Miss	Ions	Peptide
980.4000	979.3927	979.3851	7.72	100 -	106	0	---	K.QICECDR.A
993.4047	992.3975	992.4484	-51.38	24 -	31	0	---	K.MIQEETDK.N
1009.4004	1008.3931	1008.4434	-49.84	24 -	31	0	---	K.MIQEETDK.N + Oxidation (M)
1177.6357	1176.6285	1176.5557	61.8	70 -	79	1	---	K.LSDCSPKTNR.Y
1292.5783	1291.5710	1291.6707	-77.15	107 -	117	1	---	R.AAAICFRENLK.T

No match to: 855.1002, 863.3795, 868.5621, 871.0730, 875.3810, 877.3714, 895.3562, 897.3687, 903.3991, 905.3927, 907.3757, 909.3719, 920.4159, 921.3920, 925.3669, 935.3831, 937.3702, 947.3855, 952.3780, 953.3893, 954.3922, 966.3939, 968.3917, 977.3909, 979.3925, 982.3881, 984.3620, 986.3655, 992.4049, 994.3935, 996.4006, 998.3753, 1004.3794, 1006.3835, 1011.4157, 1022.1538, 1039.4114, 1043.3901, 1044.1374, 1066.1246, 1090.4345, 1106.3991, 1142.6361, 1159.6219, 1161.6411, 1163.4129, 1165.6247, 1172.6415, 1175.6433, 1178.6413, 1183.6355, 1187.6553, 1193.6308, 1197.5917, 1200.6345, 1202.6678, 1206.6509, 1209.6355, 1210.6269, 1215.6644, 1217.6575, 1219.6486, 1225.6373, 1228.6796, 1234.6590, 1249.1486, 1250.6539, 1254.6129, 1276.6050, 1280.6284, 1296.6213, 1322.6371, 1618.8519, 1650.8407, 1872.7759, 1905.7288, 1962.7642, 1974.7638, 1994.0056, 2055.9612, 2148.9294, 2345.0400, 2359.0435, 2397.2085, 2438.2368, 2454.2310, 2470.2291, 3346.7170, 3446.6176

19. Q6A378_VIPAA **Mass:** 16459 **Score:** 17 **Expect:** 28 **Matches:** 5

Ammodytin L(1'') isoform OS=Vipera ammodytes ammodytes OX=8705 PE=2 SV=1

Observed	Mr(expt)	Mr(calc)	ppm	Start	End	Miss	Ions	Peptide
980.4000	979.3927	979.3851	7.72	100 -	106	0	---	K.QICECDR.A
993.4047	992.3975	992.4484	-51.38	24 -	31	0	---	K.MIQEETDK.N
1009.4004	1008.3931	1008.4434	-49.84	24 -	31	0	---	K.MIQEETDK.N + Oxidation (M)
1177.6357	1176.6285	1176.5557	61.8	70 -	79	1	---	K.LSDCSPKTNR.Y
1292.5783	1291.5710	1291.6707	-77.15	107 -	117	1	---	R.AAAICFRENLK.T

No match to: 855.1002, 863.3795, 868.5621, 871.0730, 875.3810, 877.3714, 895.3562, 897.3687, 903.3991, 905.3927, 907.3757, 909.3719, 920.4159, 921.3920, 925.3669, 935.3831, 937.3702, 947.3855, 952.3780, 953.3893, 954.3922, 966.3939, 968.3917, 977.3909, 979.3925, 982.3881, 984.3620, 986.3655, 992.4049, 994.3935, 996.4006, 998.3753, 1004.3794, 1006.3835, 1011.4157, 1022.1538, 1039.4114, 1043.3901, 1044.1374, 1066.1246, 1090.4345, 1106.3991, 1142.6361, 1159.6219, 1161.6411, 1163.4129, 1165.6247, 1172.6415, 1175.6433, 1178.6413, 1183.6355, 1187.6553, 1193.6308, 1197.5917, 1200.6345, 1202.6678, 1206.6509, 1209.6355, 1210.6269, 1215.6644, 1217.6575, 1219.6486, 1225.6373, 1228.6796, 1234.6590, 1249.1486, 1250.6539, 1254.6129, 1276.6050, 1280.6284, 1296.6213, 1322.6371, 1618.8519, 1650.8407, 1872.7759, 1905.7288, 1962.7642, 1974.7638, 1994.0056, 2055.9612, 2148.9294, 2345.0400, 2359.0435, 2397.2085, 2438.2368, 2454.2310, 2470.2291, 3346.7170, 3446.6176

20. Q6A387_VIPAM **Mass:** 16471 **Score:** 17 **Expect:** 28 **Matches:** 5

Ammodytin L(1) isoform OS=Vipera ammodytes ruffoi OX=246549 PE=2 SV=1

Observed	Mr(expt)	Mr(calc)	ppm	Start	End	Miss	Ions	Peptide
980.4000	979.3927	979.3851	7.72	100 -	106	0	---	K.QICECDR.A
993.4047	992.3975	992.4484	-51.38	24 -	31	0	---	K.MIQEETDK.N
1009.4004	1008.3931	1008.4434	-49.84	24 -	31	0	---	K.MIQEETDK.N + Oxidation (M)
1177.6357	1176.6285	1176.5557	61.8	70 -	79	1	---	K.LSDCSPKTNR.Y
1292.5783	1291.5710	1291.6707	-77.15	107 -	117	1	---	R.AAAICFRENLK.T

No match to: 855.1002, 863.3795, 868.5621, 871.0730, 875.3810, 877.3714, 895.3562, 897.3687, 903.3991, 905.3927, 907.3757, 909.3719, 920.4159, 921.3920, 925.3669, 935.3831, 937.3702, 947.3855, 952.3780, 953.3893, 954.3922, 966.3939, 968.3917, 977.3909, 979.3925, 982.3881, 984.3620, 986.3655, 992.4049, 994.3935, 996.4006, 998.3753, 1004.3794, 1006.3835, 1011.4157, 1022.1538, 1039.4114, 1043.3901, 1044.1374, 1066.1246, 1090.4345, 1106.3991, 1142.6361, 1159.6219, 1161.6411, 1163.4129, 1165.6247, 1172.6415, 1175.6433, 1178.6413, 1183.6355, 1187.6553, 1193.6308, 1197.5917, 1200.6345, 1202.6678, 1206.6509, 1209.6355, 1210.6269, 1215.6644, 1217.6575, 1219.6486, 1225.6373, 1228.6796, 1234.6590, 1249.1486, 1250.6539, 1254.6129, 1276.6050, 1280.6284, 1296.6213, 1322.6371, 1618.8519, 1650.8407, 1872.7759, 1905.7288, 1962.7642, 1974.7638, 1994.0056, 2055.9612, 2148.9294, 2345.0400, 2359.0435, 2397.2085, 2438.2368, 2454.2310, 2470.2291, 3346.7170, 3446.6176

Search Parameters

Type of search : MS/MS Ion Search
Enzyme : Trypsin
Fixed modifications : Carbamidomethyl (C)
Variable modifications : Oxidation (M)
Mass values : Monoisotopic
Protein Mass : Unrestricted
Peptide Mass Tolerance : ± 100 ppm
Fragment Mass Tolerance: ± 0.7 Da
Max Missed Cleavages : 1
Instrument type : MALDI-TOF-TOF
Query1 (855.1002,1+) : <no title>
Query2 (863.3795,1+) : <no title>
Query3 (868.5621,1+) : <no title>
Query4 (871.0730,1+) : <no title>
Query5 (875.3810,1+) : <no title>
Query6 (877.3714,1+) : <no title>
Query7 (895.3562,1+) : <no title>
Query8 (897.3687,1+) : <no title>
Query9 (903.3991,1+) : <no title>
Query10 (905.3927,1+) : <no title>
Query11 (907.3757,1+) : <no title>
Query12 (909.3719,1+) : <no title>
Query13 (920.4159,1+) : <no title>
Query14 (921.3920,1+) : <no title>
Query15 (925.3669,1+) : <no title>
Query16 (935.3831,1+) : <no title>
Query17 (937.3702,1+) : <no title>
Query18 (947.3855,1+) : <no title>

```
Query19 (952.3780,1+)  : <no title>
Query20 (953.3893,1+)  : <no title>
Query21 (954.3922,1+)  : \\PROTEOMICS1\D\DATA\2105\210505_TLIEPOL_1_68252-68266\68252_68266_LPARDO\0_G7\1\954.3922.LIFT\1SREF\PDATA\1\1R
Query22 (966.3939,1+)  : \\PROTEOMICS1\D\DATA\2105\210505_TLIEPOL_1_68252-68266\68252_68266_LPARDO\0_G7\1\966.3939.LIFT\1SREF\PDATA\1\1R
Query23 (968.3917,1+)  : <no title>
Query24 (977.3909,1+)  : <no title>
Query25 (979.3925,1+)  : <no title>
Query26 (980.4000,1+)  : <no title>
Query27 (982.3881,1+)  : \\PROTEOMICS1\D\DATA\2105\210505_TLIEPOL_1_68252-68266\68252_68266_LPARDO\0_G7\1\982.3881.LIFT\1SREF\PDATA\1\1R
Query28 (984.3620,1+)  : <no title>
Query29 (986.3655,1+)  : <no title>
Query30 (992.4049,1+)  : <no title>
Query31 (993.4047,1+)  : <no title>
Query32 (994.3935,1+)  : <no title>
Query33 (996.4006,1+)  : <no title>
Query34 (998.3753,1+)  : <no title>
Query35 (1004.3794,1+) : <no title>
Query36 (1006.3835,1+) : <no title>
Query37 (1009.4004,1+) : <no title>
Query38 (1011.4157,1+) : \\PROTEOMICS1\D\DATA\2105\210505_TLIEPOL_1_68252-68266\68252_68266_LPARDO\0_G7\1\1011.4157.LIFT\1SREF\PDATA\1\1R
Query39 (1022.1538,1+) : <no title>
Query40 (1039.4114,1+) : <no title>
Query41 (1043.3901,1+) : <no title>
Query42 (1044.1374,1+) : <no title>
Query43 (1066.1246,1+) : <no title>
Query44 (1090.4345,1+) : <no title>
Query45 (1106.3991,1+) : <no title>
Query46 (1142.6361,1+) : <no title>
Query47 (1159.6219,1+) : <no title>
Query48 (1161.6411,1+) : \\PROTEOMICS1\D\DATA\2105\210505_TLIEPOL_1_68252-68266\68252_68266_LPARDO\0_G7\1\1161.6411.LIFT\1SREF\PDATA\1\1R
Query49 (1163.4129,1+) : <no title>
Query50 (1165.6247,1+) : <no title>
Query51 (1172.6415,1+) : <no title>
Query52 (1175.6433,1+) : <no title>
Query53 (1177.6357,1+) : <no title>
Query54 (1178.6413,1+) : <no title>
Query55 (1183.6355,1+) : <no title>
Query56 (1187.6553,1+) : <no title>
Query57 (1193.6308,1+) : <no title>
Query58 (1197.5917,1+) : <no title>
Query59 (1200.6345,1+) : <no title>
Query60 (1202.6678,1+) : <no title>
Query61 (1206.6509,1+) : <no title>
Query62 (1209.6355,1+) : <no title>
Query63 (1210.6269,1+) : <no title>
Query64 (1215.6644,1+) : <no title>
Query65 (1217.6575,1+) : <no title>
Query66 (1219.6486,1+) : <no title>
Query67 (1225.6373,1+) : <no title>
Query68 (1228.6796,1+) : <no title>
Query69 (1234.6590,1+) : <no title>
Query70 (1249.1486,1+) : <no title>
Query71 (1250.6539,1+) : <no title>
Query72 (1254.6129,1+) : \\PROTEOMICS1\D\DATA\2105\210505_TLIEPOL_1_68252-68266\68252_68266_LPARDO\0_G7\1\1254.6129.LIFT\1SREF\PDATA\1\1R
Query73 (1276.6050,1+) : <no title>
Query74 (1280.6284,1+) : <no title>
Query75 (1292.5783,1+) : <no title>
Query76 (1296.6213,1+) : <no title>
Query77 (1322.6371,1+) : <no title>
Query78 (1618.8519,1+) : <no title>
Query79 (1650.8407,1+) : <no title>
Query80 (1872.7759,1+) : <no title>
Query81 (1905.7288,1+) : <no title>
Query82 (1962.7642,1+) : <no title>
Query83 (1974.7638,1+) : <no title>
Query84 (1994.0056,1+) : <no title>
Query85 (2055.9612,1+) : <no title>
Query86 (2148.9294,1+) : <no title>
Query87 (2345.0400,1+) : \\PROTEOMICS1\D\DATA\2105\210505_TLIEPOL_1_68252-68266\68252_68266_LPARDO\0_G7\1\2345.0400.LIFT\1SREF\PDATA\1\1R
Query88 (2359.0435,1+) : <no title>
Query89 (2397.2085,1+) : \\PROTEOMICS1\D\DATA\2105\210505_TLIEPOL_1_68252-68266\68252_68266_LPARDO\0_G7\1\2397.2085.LIFT\1SREF\PDATA\1\1R
Query90 (2438.2368,1+) : <no title>
Query91 (2454.2310,1+) : <no title>
Query92 (2470.2291,1+) : <no title>
Query93 (3346.7170,1+) : <no title>
Query94 (3446.6176,1+) : <no title>
```

**Mascot:** http://www.matrixscience.com/

MATRIX SCIENCE Mascot Search Results **Fr4-1**

```
User         :
Email        :
Search title : vipera_All_8IDplus4 file:\\Proteomics1\d\data\2105\210505_tliepol_1_68252-68266\68252_68266_lpardo\0_H7\1\1SRef\pdata\1\peaklist.xml
Database     : vipera  (1414 sequences; 347452 residues)
Timestamp    : 5 May 2021 at 09:35:06 GMT
Top Score    : 80 for VM25A_MACLO, Disintegrin VLO5A OS=Macrovipera lebetina obtusa OX=209528 PE=1 SV=1
```

Mascot Score Histogram

Protein score is -10*Log(P), where P is the probability that the observed match is a random event.
Protein scores greater than 44 are significant (p<0.05).

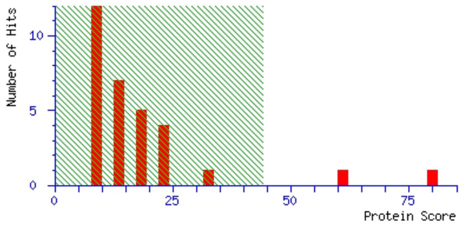

Concise Protein Summary Report

Format As | Concise Protein Summary | Help

Significance threshold p< 0.05 Max. number of hits 10

Re-Search All | Search Unmatched

1. VM25A_MACLO **Mass:** 7570 **Score:** 80 **Expect:** 1.4e-005 **Matches:** 7
   Disintegrin VLO5A OS=Macrovipera lebetina obtusa OX=209528 PE=1 SV=1
   VM2L2_MACLB **Mass:** 54586 **Score:** 28 **Expect:** 2.3 **Matches:** 6
   Zinc metalloproteinase/disintegrin OS=Macrovipera lebetina OX=8709 PE=1 SV=1
   Q7ZZQ1_DABSI **Mass:** 16246 **Score:** 17 **Expect:** 30 **Matches:** 2
   Phospholipase A(2) OS=Daboia siamensis OX=343250 PE=2 SV=1
   PA2A1_DABRR **Mass:** 16174 **Score:** 17 **Expect:** 30 **Matches:** 2
   Acidic phospholipase A2 Drk-a1 OS=Daboia russelii OX=8707 PE=1 SV=1
   PA2AA_DABSI **Mass:** 16240 **Score:** 17 **Expect:** 30 **Matches:** 2
   Acidic phospholipase A2 daboiatoxin A chain OS=Daboia siamensis OX=343250 PE=1 SV=1
   LEC1_DABRR **Mass:** 2551 **Score:** 15 **Expect:** 45 **Matches:** 1
   C-type lectin domain-containing protein 1 (Fragment) OS=Daboia russelii OX=8707 PE=1 SV=1
   DID1B_MACLB **Mass:** 7686 **Score:** 10 **Expect:** 1.5e+002 **Matches:** 1
   Disintegrin lebein-1-beta OS=Macrovipera lebetina OX=8709 PE=1 SV=1
   DID7A_VIPBB **Mass:** 7574 **Score:** 10 **Expect:** 1.5e+002 **Matches:** 1
   Disintegrin VB7A OS=Vipera berus berus OX=31156 PE=1 SV=1
   DID4_MACLO **Mass:** 7673 **Score:** 10 **Expect:** 1.6e+002 **Matches:** 1
   Disintegrin VLO4 OS=Macrovipera lebetina obtusa OX=209528 PE=1 SV=1

---

2. Q1JRG9_MACLN **Mass:** 7698 **Score:** 63 **Expect:** 0.00073 **Matches:** 8
   VGD-containing dimeric disintegrin subunit ML-G1 (Fragment) OS=Macrovipera lebetina transmediterranea OX=384075 GN=ml-G1 PE=4 SV=1
   A0A6G5ZVS4_9SAUR **Mass:** 10064 **Score:** 9 **Expect:** 1.8e+002 **Matches:** 2
   Serine protease inhibitor 5 OS=Vipera anatolica senliki OX=2604287 PE=2 SV=1

---

3. DID5B_MACLO **Mass:** 8235 **Score:** 34 **Expect:** 0.58 **Matches:** 6
   Disintegrin VLO5B OS=Macrovipera lebetina obtusa OX=209528 PE=1 SV=1

---

4. A0A6G5ZW27_9SAUR **Mass:** 38471 **Score:** 24 **Expect:** 5 **Matches:** 6
   Venom factor 6 (Fragment) OS=Vipera anatolica senliki OX=2604287 PE=2 SV=1

---

5. VM27B_VIPBB **Mass:** 7563 **Score:** 23 **Expect:** 6.6 **Matches:** 2
   Disintegrin VB7B OS=Vipera berus berus OX=31156 PE=1 SV=1

---

6. VKT_VIPRE **Mass:** 7707 **Score:** 22 **Expect:** 8 **Matches:** 2
   Kunitz-type serine protease inhibitor Vur-KIn OS=Vipera renardi OX=927686 PE=1 SV=1
   S4S374_VIPRE **Mass:** 10144 **Score:** 20 **Expect:** 14 **Matches:** 2
   Venom Kunitz trypsin inhibitor protein 2 OS=Vipera renardi OX=927686 PE=2 SV=1
   A0A6G5ZW00_9SAUR **Mass:** 10113 **Score:** 9 **Expect:** 1.8e+002 **Matches:** 1
   Cholinesterase 17 (Fragment) OS=Vipera anatolica senliki OX=2604287 PE=2 SV=1

---

7. A0A6G5ZVY2_9SAUR **Mass:** 25830 **Score:** 22 **Expect:** 8.7 **Matches:** 3
   Disintegrin and metalloproteinase domain-containing protein 8 (Fragment) OS=Vipera anatolica senliki OX=2604287 PE=2 SV=1

---

8. A0A6G5ZUH5_9SAUR **Mass:** 34006 **Score:** 18 **Expect:** 25 **Matches:** 3
   Snake venom serine protease (Fragment) OS=Vipera anatolica senliki OX=2604287 PE=2 SV=1
   A0A1S5QK15_VIPAA **Mass:** 10077 **Score:** 9 **Expect:** 1.9e+002 **Matches:** 1
   Kunitz/BPTI inhibitor-4 (Fragment) OS=Vipera ammodytes ammodytes OX=8705 PE=3 SV=1

---

9. PA2A8_DABPA **Mass:** 16194 **Score:** 17 **Expect:** 30 **Matches:** 2
   Acidic phospholipase A2 VP8 OS=Daboia palaestinae OX=1170828 PE=3 SV=1

---

10. Q6A3C0_VIPAP **Mass:** 16127 **Score:** 17 **Expect:** 30 **Matches:** 3
    Ammodytin I2(B) isoform OS=Vipera aspis aspis OX=194601 PE=2 SV=1
    Q6A3B5_VIPAS **Mass:** 16127 **Score:** 17 **Expect:** 30 **Matches:** 3
    Ammodytin I2(B) isoform OS=Vipera aspis atra OX=246182 PE=2 SV=1
    Q6A3B9_VIPAP **Mass:** 16093 **Score:** 17 **Expect:** 30 **Matches:** 3
    Ammodytin I2(B) variant OS=Vipera aspis aspis OX=194601 PE=2 SV=1
    Q6A3B7_VIPAP **Mass:** 16099 **Score:** 17 **Expect:** 30 **Matches:** 3
    Ammodytin I2(B) variant OS=Vipera aspis aspis OX=194601 PE=2 SV=1

Q7LZQ5_VIPAS **Mass:** 6213 **Score:** 11 **Expect:** 1.1e+002 **Matches:** 1
Phospholipase A2 II (Fragment) OS=Vipera aspis OX=8706 PE=3 SV=1
Q6A3D8_VIPAP **Mass:** 8011 **Score:** 10 **Expect:** 1.4e+002 **Matches:** 1
Ammodytin I2(A) isoform OS=Vipera aspis aspis OX=194601 PE=2 SV=1

Search Parameters

```
Type of search         : Peptide Mass Fingerprint
Enzyme                 : Trypsin
Fixed modifications    : Carbamidomethyl (C)
Variable modifications : Oxidation (M)
Mass values            : Monoisotopic
Protein Mass           : Unrestricted
Peptide Mass Tolerance : ± 100 ppm
Peptide Charge State   : 1+
Max Missed Cleavages   : 1
Number of queries      : 97
Selected for scoring   : 40
```

Mascot: http://www.matrixscience.com/

Mascot Search Results

```
User          :
Email         :
Search title  : vipera_all_Lift file:\\Proteomics1\d\data\2105\210505_tliepol_1_68252-68266\68252_68266_lpardo\0_H7\1\0_H7.xml
Database      : vipera  (1414 sequences; 347452 residues)
Timestamp     : 5 May 2021 at 09:52:28 GMT
Protein hits  : VM25A_MACLO Disintegrin VLO5A OS=Macrovipera lebetina obtusa OX=209528 PE=1 SV=1
                VM27B_VIPBB Disintegrin VB7B OS=Vipera berus berus OX=31156 PE=1 SV=1
                DID1B_MACLB Disintegrin lebein-1-beta OS=Macrovipera lebetina OX=8709 PE=1 SV=1
```

Mascot Score Histogram

Ions score is -10*Log(P), where P is the probability that the observed match is a random event.
Individual ions scores > 10 indicate identity or extensive homology (p<0.05).
Protein scores are derived from ions scores as a non-probabilistic basis for ranking protein hits.

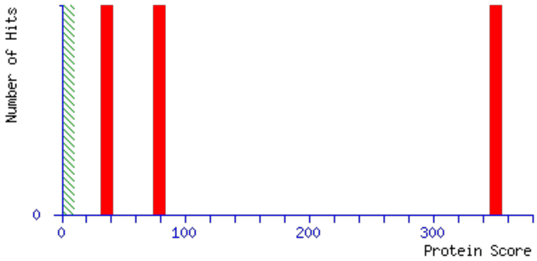

Peptide Summary Report

Format As [Peptide Summary] Help

Significance threshold p< 0.05 Max. number of hits 10

Standard scoring ○ MudPIT scoring ◉ Ions score or expect cut-off 0 Show sub-sets 0

Show pop-ups ◉ Suppress pop-ups ○ Sort unassigned [Decreasing Score] Require bold red ☐

Overview Table

Click on column header to jump to entry in results list.
Move mouse over any indicator to highlight identical peptides.
Click on an indicator to see details of individual match.
Use check boxes to select sub-set of queries for new search.

Mouse over: -Query- -Accession- -Sequence-

Hit:	1	2	3
☑ 837.4639 (1+)	●		
☑ 855.4397 (1+)			
☑ 857.4550 (1+)			
☑ 865.4570 (1+)			
☑ 878.4913 (1+)			
☑ 972.4913 (1+)			
☑ 1010.5079 (1+)			
☑ 1026.4958 (1+)			
☑ 1029.5188 (1+)	●		
☑ 1040.5171 (1+)			
☑ 1049.5677 (1+)			
☑ 1051.4970 (1+)			
☑ 1055.5324 (1+)			
☑ 1067.4164 (1+)			
☑ 1067.9287 (1+)			
☑ 1071.5325 (1+)			
☑ 1079.5338 (1+)			
☑ 1277.7337 (1+)			
☑ 1389.6057 (1+)			
☑ 1446.6389 (1+)			
☑ 1546.7035 (1+)			
☑ 1572.7099 (1+)			
☑ 1583.6660 (1+)			
☑ 1647.6988 (1+)			
☑ 1724.7416 (1+)			

☑ **1756.7151** (1+)			
☑ **1761.7398** (1+)	●		●
☑ **1770.7345** (1+)			
☑ **1781.7648** (1+)			
☑ **1783.7721** (1+)			
☑ **1796.7339** (1+)			
☑ **1812.7353** (1+)			
☑ **1815.7528** (1+)			
☑ **1823.7347** (1+)			
☑ **1826.7583** (1+)			
☑ **1828.7661** (1+)			
☑ **1831.7523** (1+)			
☑ **1841.7675** (1+)			
☑ **1843.7419** (1+)			
☑ **1855.7505** (1+)			
☑ **1868.7756** (1+)			
☑ **1870.7489** (1+)			
☑ **1872.7762** (1+)			
☑ **1883.7767** (1+)			
☑ **1886.7924** (1+)			
☑ **1894.7645** (1+)			
☑ **1900.7969** (1+)			
☑ **1903.7666** (1+)			
☑ **1905.7433** (1+)			
☑ **1910.7355** (1+)			
☑ **1914.7927** (1+)			
☑ **1920.8407** (1+)			
☑ **2002.7907** (1+)			
☑ **2017.7975** (1+)			
☑ **2024.7742** (1+)			
☑ **2031.8279** (1+)			
☑ **2042.8409** (1+)			
☑ **2044.8576** (1+)			
☑ **2058.8185** (1+)			
☑ **2072.8250** (1+)			
☑ **2076.8298** (1+)			
☑ **2085.8457** (1+)			
☑ **2087.8271** (1+)			
☑ **2089.8493** (1+)			
☑ **2092.8135** (1+)			
☑ **2094.8032** (1+)			
☑ **2103.8451** (1+)			
☑ **2105.8450** (1+)			
☑ **2114.8469** (1+)			
☑ **2117.8290** (1+)			
☑ **2119.2383** (1+)			
☑ **2120.8392** (1+)			
☑ **2128.9018** (1+)			
☑ **2131.8309** (1+)			
☑ **2133.8534** (1+)	●		
☑ **2146.8669** (1+)			
☑ **2148.8858** (1+)			
☑ **2149.8462** (1+)	●		
☑ **2159.8698** (1+)			
☑ **2163.8532** (1+)			
☑ **2174.9196** (1+)			
☑ **2176.8605** (1+)			
☑ **2181.9842** (1+)			
☑ **2190.9033** (1+)			
☑ **2198.9939** (1+)			
☑ **2226.0084** (1+)			
☑ **2233.9062** (1+)			
☑ **2245.0192** (1+)		●	
☑ **2259.0326** (1+)			
☑ **2271.0323** (1+)			
☑ **2277.8915** (1+)			
☑ **2285.8514** (1+)			

☑ 2305.8763 (1+)	●		
☑ 2321.8870 (1+)			
☑ 2359.0611 (1+)			
☑ 2699.2368 (1+)			
☑ 2887.3239 (1+)			

Select All Select None Search Selected ☐ **Error tolerant** Archive Report

1. VM25A_MACLO **Mass:** 7570 **Score:** 350 **Matches:** 6(6) **Sequences:** 5(5)
Disintegrin VLO5A OS=Macrovipera lebetina obtusa OX=209528 PE=1 SV=1
☐ Check to include this hit in error tolerant search or archive report

Query	Observed	Mr(expt)	Mr(calc)	ppm	Miss	Score	Expect	Rank	Unique	Peptide
1	837.4639	836.4566	836.4327	28.6	1	24	0.0036	1	U	R.NCKFLR.A
9	1029.5188	1028.5115	1028.4822	28.5	1	31	0.0017	1	U	R.RGEHCVSGK.C
27	1761.7398	1760.7325	1760.7029	16.8	0	36	0.00023	1	U	-.NSGNPCCDPVTCQPR.R
75	2133.8534	2132.8462	2132.8085	17.6	0	151	8.2e-016	1	U	R.AVGDDMDDYCTGISSDCPR.N
78	2149.8462	2148.8390	2148.8035	16.5	0	(117)	1.8e-012	1	U	R.AVGDDMDDYCTGISSDCPR.N + Oxidation (M)
93	2305.8763	2304.8690	2304.9046	-15.43	1	58	1.7e-006	1	U	K.RAVGDDMDDYCTGISSDCPR.N + Oxidation (M)

2. VM27B_VIPBB **Mass:** 7563 **Score:** 75 **Matches:** 1(1) **Sequences:** 1(1)
Disintegrin VB7B OS=Vipera berus berus OX=31156 PE=1 SV=1
☐ Check to include this hit in error tolerant search or archive report

Query	Observed	Mr(expt)	Mr(calc)	ppm	Miss	Score	Expect	Rank	Unique	Peptide
88	2245.0192	2244.0119	2244.0086	1.51	0	75	3.4e-008	1	U	-.ELLQNSGNPCCDPVTCKPR.E

3. DID1B_MACLB **Mass:** 7686 **Score:** 36 **Matches:** 1(1) **Sequences:** 1(1)
Disintegrin lebein-1-beta OS=Macrovipera lebetina OX=8709 PE=1 SV=1
☐ Check to include this hit in error tolerant search or archive report

Query	Observed	Mr(expt)	Mr(calc)	ppm	Miss	Score	Expect	Rank	Unique	Peptide
27	1761.7398	1760.7325	1760.7393	-3.84	0	36	0.00023	1	U	-.NSGNPCCDPVTCKPR.R

Proteins matching the same set of peptides:
DID4_MACLO **Mass:** 7673 **Score:** 36 **Matches:** 1(1) **Sequences:** 1(1)
Disintegrin VLO4 OS=Macrovipera lebetina obtusa OX=209528 PE=1 SV=1
DID7A_VIPBB **Mass:** 7574 **Score:** 36 **Matches:** 1(1) **Sequences:** 1(1)
Disintegrin VB7A OS=Vipera berus berus OX=31156 PE=1 SV=1

Peptide matches not assigned to protein hits: (no details means no match)

	Query	Observed	Mr(expt)	Mr(calc)	ppm	Miss	Score	Expect	Rank	Unique	Peptide
☑	2	*855.4397*	*854.4325*								
☑	3	*857.4550*	*856.4477*								
☑	4	*865.4570*	*864.4497*								
☑	5	*878.4913*	*877.4840*								
☑	6	*972.4913*	*971.4840*								
☑	7	*1010.5079*	*1009.5006*								
☑	8	*1026.4958*	*1025.4885*								
☑	10	*1040.5171*	*1039.5098*								
☑	11	*1049.5677*	*1048.5604*								
☑	12	*1051.4970*	*1050.4897*								
☑	13	*1055.5324*	*1054.5251*								
☑	14	*1067.4164*	*1066.4091*								
☑	15	*1067.9287*	*1066.9215*								
☑	16	*1071.5325*	*1070.5253*								
☑	17	*1079.5338*	*1078.5265*								
☑	18	*1277.7337*	*1276.7264*								
☑	19	*1389.6057*	*1388.5985*								
☑	20	*1446.6389*	*1445.6317*								
☑	21	*1546.7035*	*1545.6962*								
☑	22	*1572.7099*	*1571.7027*								
☑	23	*1583.6660*	*1582.6587*								
☑	24	*1647.6988*	*1646.6915*								
☑	25	*1724.7416*	*1723.7343*								
☑	26	*1756.7151*	*1755.7078*								
☑	28	*1770.7345*	*1769.7272*								
☑	29	*1781.7648*	*1780.7575*								
☑	30	*1783.7721*	*1782.7648*								
☑	31	*1796.7339*	*1795.7266*								
☑	32	*1812.7353*	*1811.7280*								
☑	33	*1815.7528*	*1814.7455*								
☑	34	*1823.7347*	*1822.7274*								
☑	35	*1826.7583*	*1825.7511*								
☑	36	*1828.7661*	*1827.7588*								

☑	37	1831.7523	1830.7451
☑	38	1841.7675	1840.7602
☑	39	1843.7419	1842.7346
☑	40	1855.7505	1854.7432
☑	41	1868.7756	1867.7683
☑	42	1870.7489	1869.7417
☑	43	1872.7762	1871.7689
☑	44	1883.7767	1882.7694
☑	45	1886.7924	1885.7852
☑	46	1894.7645	1893.7572
☑	47	1900.7969	1899.7897
☑	48	1903.7666	1902.7593
☑	49	1905.7433	1904.7360
☑	50	1910.7355	1909.7282
☑	51	1914.7927	1913.7854
☑	52	1920.8407	1919.8335
☑	53	2002.7907	2001.7834
☑	54	2017.7975	2016.7903
☑	55	2024.7742	2023.7669
☑	56	2031.8279	2030.8206
☑	57	2042.8409	2041.8336
☑	58	2044.8576	2043.8503
☑	59	2058.8185	2057.8113
☑	60	2072.8250	2071.8177
☑	61	2076.8298	2075.8225
☑	62	2085.8457	2084.8385
☑	63	2087.8271	2086.8198
☑	64	2089.8493	2088.8420
☑	65	2092.8135	2091.8062
☑	66	2094.8032	2093.7960
☑	67	2103.8451	2102.8378
☑	68	2105.8450	2104.8377
☑	69	2114.8469	2113.8396
☑	70	2117.8290	2116.8217
☑	71	2119.2383	2118.2310
☑	72	2120.8392	2119.8319
☑	73	2128.9018	2127.8945
☑	74	2131.8309	2130.8236
☑	76	2146.8669	2145.8596
☑	77	2148.8858	2147.8785
☑	79	2159.8698	2158.8625
☑	80	2163.8532	2162.8459
☑	81	2174.9196	2173.9123
☑	82	2176.8605	2175.8533
☑	83	2181.9842	2180.9769
☑	84	2190.9033	2189.8960
☑	85	2198.9939	2197.9866
☑	86	2226.0084	2225.0011
☑	87	2233.9062	2232.8989
☑	89	2259.0326	2258.0253
☑	90	2271.0323	2270.0250
☑	91	2277.8915	2276.8842
☑	92	2285.8514	2284.8441
☑	94	2321.8870	2320.8797
☑	95	2359.0611	2358.0538
☑	96	2699.2368	2698.2295
☑	97	2887.3239	2886.3167

Search Parameters

Type of search : MS/MS Ion Search
Enzyme : Trypsin
Fixed modifications : Carbamidomethyl (C)
Variable modifications : Oxidation (M)
Mass values : Monoisotopic
Protein Mass : Unrestricted
Peptide Mass Tolerance : ± 100 ppm
Fragment Mass Tolerance: ± 0.7 Da
Max Missed Cleavages : 1
Instrument type : MALDI-TOF-TOF
Number of queries : 97

Mascot: http://www.matrixscience.com/

Mascot Search Results

```
User            :
Email           :
Search title    : vipera_all_Lift file:\\Proteomics1\d\data\2105\210505_tliepol_1_68252-68266\68252_68266_1pardo\0_H7\1\0_H7.xml
Database        : vipera  (1414 sequences; 347452 residues)
Timestamp       : 5 May 2021 at 09:52:28 GMT
Warning         : A Peptide summary report will usually give a much clearer picture of MS/MS search results.
Top Score       : 356 for VM25A_MACLO, Disintegrin VLO5A OS=Macrovipera lebetina obtusa OX=209528 PE=1 SV=1
```

Mascot Score Histogram

Protein score is -10*Log(P), where P is the probability that the observed match is a random event.
Protein scores greater than 44 are significant (p<0.05).
Protein scores are derived from ions scores as a non-probabilistic basis for ranking protein hits.

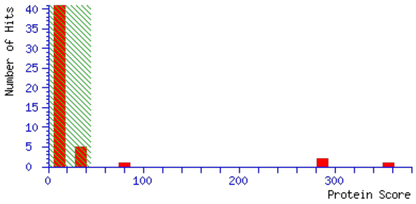

Protein Summary Report

Format As | Protein Summary (deprecated) | Help

Significance threshold p< 0.05 Max. number of hits 20

Overview Table

Click on column header to jump to entry in results list.
Move mouse over any indicator to highlight identical peptides.
Click on an indicator to see details of individual match.
Use check boxes to select sub-set of queries for new search.

Mouse over: -Query-
-Accession- -Sequence-

Hit:	1	2	3	4	5	6	7	8	9	10	11	12	13	14	15	16	17	18	19	20
☑ 837.4639 (1+)	●	●	●																	
☑ 855.4397 (1+)																				
☑ 857.4550 (1+)																				
☑ 865.4570 (1+)																				
☑ 878.4913 (1+)																				
☑ 972.4913 (1+)																				
☑ 1010.5079 (1+)																				
☑ 1026.4958 (1+)																				
☑ 1029.5188 (1+)	●		●																	
☑ 1040.5171 (1+)																				
☑ 1049.5677 (1+)																				
☑ 1051.4970 (1+)																				
☑ 1055.5324 (1+)																				
☑ 1067.4164 (1+)																				
☑ 1067.9287 (1+)																				
☑ 1071.5325 (1+)																				
☑ 1079.5338 (1+)																				
☑ 1277.7337 (1+)																				
☑ 1389.6057 (1+)																				
☑ 1446.6389 (1+)																				
☑ 1546.7035 (1+)																				
☑ 1572.7099 (1+)																				
☑ 1583.6660 (1+)																				
☑ 1647.6988 (1+)																				
☑ 1724.7416 (1+)																				
☑ 1756.7151 (1+)																				
☑ 1761.7398 (1+)	●				●	●	●													
☑ 1770.7345 (1+)																				
☑ 1781.7648 (1+)																				
☑ 1783.7721 (1+)																				
☑ 1796.7339 (1+)																				
☑ 1812.7353 (1+)																				
☑ 1815.7528 (1+)																				
☑ 1823.7347 (1+)																				
☑ 1826.7583 (1+)																				
☑ 1828.7661 (1+)																				
☑ 1831.7523 (1+)																				
☑ 1841.7675 (1+)																				
☑ 1843.7419 (1+)																				
☑ 1855.7505 (1+)																				
☑ 1868.7756 (1+)																				
☑ 1870.7489 (1+)																				
☑ 1872.7762 (1+)																				
☑ 1883.7767 (1+)																				
☑ 1886.7924 (1+)																				
☑ 1894.7645 (1+)																				
☑ 1900.7969 (1+)																				
☑ 1903.7666 (1+)																				
☑ 1905.7433 (1+)																				

☑ 1910.7355 (1+)																				
☑ 1914.7927 (1+)																				
☑ 1920.8407 (1+)																				
☑ 2002.7907 (1+)																				
☑ 2017.7975 (1+)																				
☑ 2024.7742 (1+)																				
☑ 2031.8279 (1+)																				
☑ 2042.8409 (1+)																				
☑ 2044.8576 (1+)																				
☑ 2058.8185 (1+)																				
☑ 2072.8250 (1+)																				
☑ 2076.8298 (1+)																				
☑ 2085.8457 (1+)																				
☑ 2087.8271 (1+)																				
☑ 2089.8493 (1+)																				
☑ 2092.8135 (1+)																				
☑ 2094.8032 (1+)																				
☑ 2103.8451 (1+)																				
☑ 2105.8450 (1+)																				
☑ 2114.8469 (1+)																				
☑ 2117.8290 (1+)																				
☑ 2119.2383 (1+)																				
☑ 2120.8392 (1+)																				
☑ 2128.9018 (1+)																				
☑ 2131.8309 (1+)																				
☑ 2133.8534 (1+)	●	●	●																	
☑ 2146.8669 (1+)																				
☑ 2148.8858 (1+)																				
☑ 2149.8462 (1+)	●	●	●																	
☑ 2159.8698 (1+)																				
☑ 2163.8532 (1+)																				
☑ 2174.9196 (1+)																				
☑ 2176.8605 (1+)																				
☑ 2181.9842 (1+)																				
☑ 2190.9033 (1+)																				
☑ 2198.9939 (1+)																				
☑ 2226.0084 (1+)																				
☑ 2233.9062 (1+)																				
☑ 2245.0192 (1+)				●																
☑ 2259.0326 (1+)																				
☑ 2271.0323 (1+)																				
☑ 2277.8915 (1+)																				
☑ 2285.8514 (1+)																				
☑ 2305.8763 (1+)	●	●	●																	
☑ 2321.8870 (1+)																				
☑ 2359.0611 (1+)																				
☑ 2699.2368 (1+)																				
☑ 2887.3239 (1+)																				

Select All | Select None | Search Selected

Index

	Accession	Mass	Score	Description
1.	VM25A_MACLO	7570	356	Disintegrin VLO5A OS=Macrovipera lebetina obtusa OX=209528 PE=1 SV=1
2.	Q1JRG9_MACLN	7698	289	VGD-containing dimeric disintegrin subunit ML-G1 (Fragment) OS=Macrovipera lebetina transmediterranea OX=384075 GN=ml-G1 PE=4 SV=1
3.	VM2L2_MACLB	54586	277	Zinc metalloproteinase/disintegrin OS=Macrovipera lebetina OX=8709 PE=1 SV=1
4.	VM27B_VIPBB	7563	91	Disintegrin VB7B OS=Vipera berus berus OX=31156 PE=1 SV=1
5.	DID1B_MACLB	7686	43	Disintegrin lebein-1-beta OS=Macrovipera lebetina OX=8709 PE=1 SV=1
6.	DID7A_VIPBB	7574	43	Disintegrin VB7A OS=Vipera berus berus OX=31156 PE=1 SV=1
7.	DID4_MACLO	7673	43	Disintegrin VLO4 OS=Macrovipera lebetina obtusa OX=209528 PE=1 SV=1
8.	DID5B_MACLO	8235	34	Disintegrin VLO5B OS=Macrovipera lebetina obtusa OX=209528 PE=1 SV=1
9.	PA2AB_DABSI	15673	24	Acidic phospholipase A2 daboiatoxin B chain (Fragment) OS=Daboia siamensis OX=343250 PE=1 SV=1
10.	A0A6G5ZUI9_9SAUR	58303	21	Angiopoietin OS=Vipera anatolica senliki OX=2604287 PE=2 SV=1
11.	M4T472_9PERO	26404	20	Ring finger protein 213 (Fragment) OS=Echiichthys vipera OX=94984 PE=4 SV=1
12.	B8Q051_9PERO	33786	20	RNF213 (Fragment) OS=Echiichthys vipera OX=94984 GN=RNF213 PE=4 SV=1
13.	A0A286S1C5_9SAUR	39024	19	E3 ubiquitin-protein ligase RAG1 (Fragment) OS=Vipera seoanei OX=110211 GN=RAG1 PE=4 SV=1
14.	Q6A3C6_VIPAM	16212	17	Ammodytin I2(A) isoform OS=Vipera ammodytes ruffoi OX=246549 PE=2 SV=1
15.	Q6A3C0_VIPAP	16127	17	Ammodytin I2(B) isoform OS=Vipera aspis aspis OX=194601 PE=2 SV=1
16.	Q6A3B5_VIPAS	16127	17	Ammodytin I2(B) isoform OS=Vipera aspis atra OX=246182 PE=2 SV=1
17.	Q6A3B9_VIPAP	16093	17	Ammodytin I2(B) variant OS=Vipera aspis aspis OX=194601 PE=2 SV=1
18.	Q6A3B7_VIPAP	16099	17	Ammodytin I2(B) variant OS=Vipera aspis aspis OX=194601 PE=2 SV=1
19.	W8EEP5_MACLB	41360	17	Hyaluronidase (Fragment) OS=Macrovipera lebetina OX=8709 PE=2 SV=1
20.	VKT2_DABSI	7187	16	Kunitz-type serine protease inhibitor 2 OS=Daboia siamensis OX=343250 PE=1 SV=1

Results List

1. VM25A_MACLO **Mass:** 7570 **Score:** 356 **Expect:** 3.6e-033 **Matches:** 7

Disintegrin VLO5A OS=Macrovipera lebetina obtusa OX=209528 PE=1 SV=1

Observed	Mr(expt)	Mr(calc)	ppm	Start	End	Miss	Ions	Peptide
837.4639	836.4566	836.4327	28.6	28 -	33	1	24	R.NCKFLR.A
1029.5188	1028.5115	1028.4822	28.5	16 -	24	1	31	R.RGEHCVSGK.C
1051.4970	1050.4897	1050.5644	-71.12	31 -	39	1	---	K.FLRAGTVCK.R
1761.7398	1760.7325	1760.7029	16.8	1 -	15	0	36	-.NSGNPCCDPVTCQPR.R
2133.8534	2132.8462	2132.8085	17.6	41 -	59	0	151	R.AVGDDMDDYCTGISSDCPR.N
2149.8462	2148.8390	2148.8035	16.5	41 -	59	0	(117)	R.AVGDDMDDYCTGISSDCPR.N + Oxidation (M)
2305.8763	2304.8690	2304.9046	-15.43	40 -	59	1	58	K.RAVGDDMDDYCTGISSDCPR.N + Oxidation (M)

No match to: 855.4397, 857.4550, 865.4570, 878.4913, 972.4913, 1010.5079, 1026.4958, 1040.5171, 1049.5677, 1055.5324, 1067.4164, 1067.9287, 1071.5325, 1079.5338, 1277.7337, 1389.6057, 1446.6389, 1546.7035, 1572.7099, 1583.6660, 1647.6988, 1724.7416, 1756.7151, 1770.7345, 1781.7648, 1783.7721, 1796.7339, 1812.7353, 1815.7528, 1823.7347, 1826.7583, 1828.7661, 1831.7523, 1841.7675, 1843.7419, 1855.7505, 1868.7756, 1870.7489, 1872.7762, 1886.7924, 1894.7645, 1900.7969, 1903.7666, 1905.7433, 1910.7355, 1914.7927, 1920.8407, 2002.7907, 2017.7975, 2024.7742, 2031.8279, 2042.8409, 2044.8576, 2058.8185, 2072.8250, 2076.8298, 2085.8457, 2087.8271, 2089.8493, 2092.8135, 2094.8032, 2103.8451, 2105.8450, 2114.8469, 2117.8290, 2119.2383, 2120.8392, 2128.9018, 2131.8309, 2146.8669, 2148.8858, 2159.8698, 2163.8532, 2174.9196, 2176.8605, 2181.9842, 2190.9033, 2198.9939, 2226.0084, 2233.9062, 2245.0192, 2259.0326, 2271.0323, 2277.8915, 2285.8514, 2321.8870, 2359.0611, 2699.2368, 2887.3239

2. Q1JRG9_MACLN **Mass:** 7698 **Score:** 289 **Expect:** 1.8e-026 **Matches:** 8

VGD-containing dimeric disintegrin subunit ML-G1 (Fragment) OS=Macrovipera lebetina transmediterranea OX=384075 GN=ml-G1 PE=4 SV=1

Observed	Mr(expt)	Mr(calc)	ppm	Start	End	Miss	Ions	Peptide
837.4639	836.4566	836.4327	28.6	29 -	34	1	24	R.NCKFLR.A
1051.4970	1050.4897	1050.5644	-71.12	32 -	40	1	---	K.FLRAGTVCK.R

1920.8407	1919.8335	1919.8110	11.7	1	16	0	---	-.MNSANPCCDPITCKPR.R
2076.8298	2075.8225	2075.9121	-43.18	1	17	1	---	-.MNSANPCCDPITCKPRR.G
2092.8135	2091.8062	2091.9071	-48.20	1	17	1	---	-.MNSANPCCDPITCKPRR.G + Oxidation (M)
2133.8534	2132.8462	2132.8085	17.6	42	60	0	151	R.AVGDDMDDYCTGISSDCPR.N
2149.8462	2148.8390	2148.8035	16.5	42	60	0	(117)	R.AVGDDMDDYCTGISSDCPR.N + Oxidation (M)
2305.8763	2304.8690	2304.9046	-15.43	41	60	1	58	K.RAVGDDMDDYCTGISSDCPR.N + Oxidation (M)

No match to: 855.4397, 857.4550, 865.4570, 878.4913, 972.4913, 1010.5079, 1026.4958, 1029.5188, 1040.5171, 1049.5677, 1055.5324, 1067.4164, 1067.9287, 1071.5325, 1079.5338, 1277.7337, 1389.6057, 1446.6389, 1546.7035, 1572.7099, 1583.6660, 1647.6988, 1724.7416, 1756.7151, 1761.7398, 1770.7345, 1781.7648, 1783.7721, 1796.7339, 1812.7353, 1815.7528, 1823.7347, 1826.7583, 1828.7661, 1831.7523, 1841.7675, 1843.7419, 1855.7505, 1868.7756, 1870.7489, 1872.7762, 1883.7767, 1886.7924, 1894.7645, 1900.7969, 1903.7666, 1905.7433, 1910.7355, 1914.7927, 2002.7907, 2017.7975, 2024.7742, 2031.8279, 2042.8409, 2044.8576, 2058.8185, 2072.8250, 2085.8457, 2087.8271, 2089.8493, 2094.8032, 2103.8451, 2105.8450, 2114.8469, 2117.8290, 2119.2383, 2120.8392, 2128.9018, 2131.8309, 2146.8669, 2148.8858, 2159.8698, 2163.8532, 2174.9196, 2176.8605, 2181.9842, 2190.9033, 2198.9939, 2226.0084, 2233.9062, 2245.0192, 2259.0326, 2271.0323, 2277.8915, 2285.8514, 2321.8870, 2359.0611, 2699.2368, 2887.3239

3. VM2L2_MACLB **Mass:** 54586 **Score:** 277 **Expect:** 2.8e-025 **Matches:** 6
Zinc metalloproteinase/disintegrin OS=Macrovipera lebetina OX=8709 PE=1 SV=1

Observed	Mr(expt)	Mr(calc)	ppm	Start	End	Miss	Ions	Peptide
837.4639	836.4566	836.4327	28.6	442	447	1	24	R.NCKFLR.A
1029.5188	1028.5115	1028.4822	28.5	430	438	1	31	R.RGEHCVSGK.C
1051.4970	1050.4897	1050.5644	-71.12	445	453	1	---	K.FLRAGTVCK.R
2133.8534	2132.8462	2132.8085	17.6	455	473	0	151	R.AVGDDMDDYCTGISSDCPR.N
2149.8462	2148.8390	2148.8035	16.5	455	473	0	(117)	R.AVGDDMDDYCTGISSDCPR.N + Oxidation (M)
2305.8763	2304.8690	2304.9046	-15.43	454	473	1	58	K.RAVGDDMDDYCTGISSDCPR.N + Oxidation (M)

No match to: 855.4397, 857.4550, 865.4570, 878.4913, 972.4913, 1010.5079, 1026.4958, 1040.5171, 1049.5677, 1055.5324, 1067.4164, 1067.9287, 1071.5325, 1079.5338, 1277.7337, 1389.6057, 1446.6389, 1546.7035, 1572.7099, 1583.6660, 1647.6988, 1724.7416, 1756.7151, 1761.7398, 1770.7345, 1781.7648, 1783.7721, 1796.7339, 1812.7353, 1815.7528, 1823.7347, 1826.7583, 1828.7661, 1831.7523, 1841.7675, 1843.7419, 1855.7505, 1868.7756, 1870.7489, 1872.7762, 1883.7767, 1886.7924, 1894.7645, 1900.7969, 1903.7666, 1905.7433, 1910.7355, 1914.7927, 1920.8407, 2002.7907, 2017.7975, 2024.7742, 2031.8279, 2042.8409, 2044.8576, 2058.8185, 2072.8250, 2076.8298, 2085.8457, 2087.8271, 2089.8493, 2092.8135, 2094.8032, 2103.8451, 2105.8450, 2114.8469, 2117.8290, 2119.2383, 2120.8392, 2128.9018, 2131.8309, 2146.8669, 2148.8858, 2159.8698, 2163.8532, 2174.9196, 2176.8605, 2181.9842, 2190.9033, 2198.9939, 2226.0084, 2233.9062, 2245.0192, 2259.0326, 2271.0323, 2277.8915, 2285.8514, 2321.8870, 2359.0611, 2699.2368, 2887.3239

4. VM27B_VIPBB **Mass:** 7563 **Score:** 91 **Expect:** 1.1e-006 **Matches:** 2
Disintegrin VB7B OS=Vipera berus berus OX=31156 PE=1 SV=1

Observed	Mr(expt)	Mr(calc)	ppm	Start	End	Miss	Ions	Peptide
2146.8669	2145.8596	2145.8303	13.7	47	64	1	---	K.GDWMNNYCTGISSDCPRN.-
2245.0192	2244.0119	2244.0086	1.51	1	19	0	75	-.ELLQNSGNPCCDPVTCKPR.E

No match to: 837.4639, 855.4397, 857.4550, 865.4570, 878.4913, 972.4913, 1010.5079, 1026.4958, 1029.5188, 1040.5171, 1049.5677, 1051.4970, 1055.5324, 1067.4164, 1067.9287, 1071.5325, 1079.5338, 1277.7337, 1389.6057, 1446.6389, 1546.7035, 1572.7099, 1583.6660, 1647.6988, 1724.7416, 1756.7151, 1761.7398, 1770.7345, 1781.7648, 1783.7721, 1796.7339, 1812.7353, 1815.7528, 1823.7347, 1826.7583, 1828.7661, 1831.7523, 1841.7675, 1843.7419, 1855.7505, 1868.7756, 1870.7489, 1872.7762, 1883.7767, 1886.7924, 1894.7645, 1900.7969, 1903.7666, 1905.7433, 1910.7355, 1914.7927, 1920.8407, 2002.7907, 2017.7975, 2024.7742, 2031.8279, 2042.8409, 2044.8576, 2058.8185, 2072.8250, 2076.8298, 2085.8457, 2087.8271, 2089.8493, 2092.8135, 2094.8032, 2103.8451, 2105.8450, 2114.8469, 2117.8290, 2119.2383, 2120.8392, 2128.9018, 2131.8309, 2133.8534, 2148.8858, 2149.8462, 2159.8698, 2163.8532, 2174.9196, 2176.8605, 2181.9842, 2190.9033, 2198.9939, 2226.0084, 2233.9062, 2259.0326, 2271.0323, 2277.8915, 2285.8514, 2305.8763, 2321.8870, 2359.0611, 2699.2368, 2887.3239

5. DID1B_MACLB **Mass:** 7686 **Score:** 43 **Expect:** 0.073 **Matches:** 1
Disintegrin lebein-1-beta OS=Macrovipera lebetina OX=8709 PE=1 SV=1

Observed	Mr(expt)	Mr(calc)	ppm	Start	End	Miss	Ions	Peptide
1761.7398	1760.7325	1760.7393	-3.84	1	15	0	36	-.NSGNPCCDPVTCKPR.R

No match to: 837.4639, 855.4397, 857.4550, 865.4570, 878.4913, 972.4913, 1010.5079, 1026.4958, 1029.5188, 1040.5171, 1049.5677, 1051.4970, 1055.5324, 1067.4164, 1067.9287, 1071.5325, 1079.5338, 1277.7337, 1389.6057, 1446.6389, 1546.7035, 1572.7099, 1583.6660, 1647.6988, 1724.7416, 1756.7151, 1770.7345, 1781.7648, 1783.7721, 1796.7339, 1812.7353, 1815.7528, 1823.7347, 1826.7583, 1828.7661, 1831.7523, 1841.7675, 1843.7419, 1855.7505, 1868.7756, 1870.7489, 1872.7762, 1883.7767, 1886.7924, 1894.7645, 1900.7969, 1903.7666, 1905.7433, 1910.7355, 1914.7927, 1920.8407, 2002.7907, 2017.7975, 2024.7742, 2031.8279, 2042.8409, 2044.8576, 2058.8185, 2072.8250, 2076.8298, 2085.8457, 2087.8271, 2089.8493, 2092.8135, 2094.8032, 2103.8451, 2105.8450, 2114.8469, 2117.8290, 2119.2383, 2120.8392, 2128.9018, 2131.8309, 2133.8534, 2146.8669, 2148.8858, 2149.8462, 2159.8698, 2163.8532, 2174.9196, 2176.8605, 2181.9842, 2190.9033, 2198.9939, 2226.0084, 2233.9062, 2245.0192, 2259.0326, 2271.0323, 2277.8915, 2285.8514, 2305.8763, 2321.8870, 2359.0611, 2699.2368, 2887.3239

6. DID7A_VIPBB **Mass:** 7574 **Score:** 43 **Expect:** 0.073 **Matches:** 1
Disintegrin VB7A OS=Vipera berus berus OX=31156 PE=1 SV=1

Observed	Mr(expt)	Mr(calc)	ppm	Start	End	Miss	Ions	Peptide
1761.7398	1760.7325	1760.7393	-3.84	1	15	0	36	-.NSGNPCCDPVTCKPR.R

No match to: 837.4639, 855.4397, 857.4550, 865.4570, 878.4913, 972.4913, 1010.5079, 1026.4958, 1029.5188, 1040.5171, 1049.5677, 1051.4970, 1055.5324, 1067.4164, 1067.9287, 1071.5325, 1079.5338, 1277.7337, 1389.6057, 1446.6389, 1546.7035, 1572.7099, 1583.6660, 1647.6988, 1724.7416, 1756.7151, 1770.7345, 1781.7648, 1783.7721, 1796.7339, 1812.7353, 1815.7528, 1823.7347, 1826.7583, 1828.7661, 1831.7523, 1841.7675, 1843.7419, 1855.7505, 1868.7756, 1870.7489, 1872.7762, 1883.7767, 1886.7924, 1894.7645, 1900.7969, 1903.7666, 1905.7433, 1910.7355, 1914.7927, 1920.8407, 2002.7907, 2017.7975, 2024.7742, 2031.8279, 2042.8409, 2044.8576, 2058.8185, 2072.8250, 2076.8298, 2085.8457, 2087.8271, 2089.8493, 2092.8135, 2094.8032, 2103.8451, 2105.8450, 2114.8469, 2117.8290, 2119.2383, 2120.8392, 2128.9018, 2131.8309, 2133.8534, 2146.8669, 2148.8858, 2149.8462, 2159.8698, 2163.8532, 2174.9196, 2176.8605, 2181.9842, 2190.9033, 2198.9939, 2226.0084, 2233.9062, 2245.0192, 2259.0326, 2271.0323, 2277.8915, 2285.8514, 2305.8763, 2321.8870, 2359.0611, 2699.2368, 2887.3239

7. DID4_MACLO **Mass:** 7673 **Score:** 43 **Expect:** 0.074 **Matches:** 1
Disintegrin VLO4 OS=Macrovipera lebetina obtusa OX=209528 PE=1 SV=1

Observed	Mr(expt)	Mr(calc)	ppm	Start	End	Miss	Ions	Peptide
1761.7398	1760.7325	1760.7393	-3.84	2	16	0	36	M.NSGNPCCDPVTCKPR.R

No match to: 837.4639, 855.4397, 857.4550, 865.4570, 878.4913, 972.4913, 1010.5079, 1026.4958, 1029.5188, 1040.5171, 1049.5677, 1051.4970, 1055.5324, 1067.4164, 1067.9287, 1071.5325, 1079.5338, 1277.7337, 1389.6057, 1446.6389, 1546.7035, 1572.7099, 1583.6660, 1647.6988, 1724.7416, 1756.7151, 1770.7345, 1781.7648, 1783.7721, 1796.7339, 1812.7353, 1815.7528, 1823.7347, 1826.7583, 1828.7661, 1831.7523, 1841.7675, 1843.7419, 1855.7505, 1868.7756, 1870.7489, 1872.7762, 1883.7767, 1886.7924, 1894.7645, 1900.7969, 1903.7666, 1905.7433, 1910.7355, 1914.7927, 1920.8407, 2002.7907, 2017.7975, 2024.7742, 2031.8279, 2042.8409, 2044.8576, 2058.8185, 2072.8250, 2076.8298, 2085.8457, 2087.8271, 2089.8493, 2092.8135, 2094.8032, 2103.8451, 2105.8450, 2114.8469, 2117.8290, 2119.2383, 2120.8392, 2128.9018, 2131.8309, 2133.8534, 2146.8669, 2148.8858, 2149.8462, 2159.8698, 2163.8532, 2174.9196, 2176.8605, 2181.9842, 2190.9033, 2198.9939, 2226.0084, 2233.9062, 2245.0192, 2259.0326, 2271.0323, 2277.8915, 2285.8514, 2305.8763, 2321.8870, 2359.0611, 2699.2368, 2887.3239

8. DID5B_MACLO **Mass:** 8235 **Score:** 34 **Expect:** 0.6 **Matches:** 6
Disintegrin VLO5B OS=Macrovipera lebetina obtusa OX=209528 PE=1 SV=1

Observed	Mr(expt)	Mr(calc)	ppm	Start	End	Miss	Ions	Peptide
1049.5677	1048.5604	1048.5376	21.8	32	40	0	---	K.FLNPGTICK.R
1920.8407	1919.8335	1919.8110	11.7	1	16	0	---	-.MNSANPCCDPITCKPR.R
2076.8298	2075.8225	2075.9121	-43.18	1	17	1	---	-.MNSANPCCDPITCKPRR.G
2092.8135	2091.8062	2091.9071	-48.20	1	17	1	---	-.MNSANPCCDPITCKPRR.G + Oxidation (M)
2174.9196	2173.9123	2173.9079	2.03	42	60	0	---	R.TMLDGLNDYCTGVTSDCPR.N
2190.9033	2189.8960	2189.9028	-3.08	42	60	0	---	R.TMLDGLNDYCTGVTSDCPR.N + Oxidation (M)

No match to: 837.4639, 855.4397, 857.4550, 865.4570, 878.4913, 972.4913, 1010.5079, 1026.4958, 1029.5188, 1040.5171, 1051.4970, 1055.5324, 1067.4164, 1067.9287, 1071.5325, 1079.5338, 1277.7337, 1389.6057, 1446.6389, 1546.7035, 1572.7099, 1583.6660, 1647.6988, 1724.7416, 1756.7151, 1761.7398, 1770.7345, 1781.7648, 1783.7721, 1796.7339, 1812.7353, 1815.7528, 1823.7347, 1826.7583, 1828.7661, 1831.7523, 1841.7675, 1843.7419, 1855.7505, 1868.7756, 1870.7489, 1872.7762, 1883.7767, 1886.7924, 1894.7645, 1900.7969, 1903.7666, 1905.7433, 1910.7355, 1914.7927, 2002.7907, 2017.7975, 2024.7742, 2031.8279, 2042.8409, 2044.8576, 2058.8185, 2072.8250, 2085.8457, 2087.8271, 2089.8493, 2094.8032, 2103.8451, 2105.8450, 2114.8469, 2117.8290, 2119.2383, 2120.8392, 2128.9018, 2131.8309, 2133.8534, 2146.8669, 2148.8858, 2149.8462, 2159.8698, 2163.8532, 2176.8605, 2181.9842, 2198.9939, 2226.0084, 2233.9062, 2245.0192, 2259.0326, 2271.0323, 2277.8915, 2285.8514, 2305.8763, 2321.8870, 2359.0611, 2699.2368, 2887.3239

9. PA2AB_DABSI **Mass:** 15673 **Score:** 24 **Expect:** 6 **Matches:** 5
Acidic phospholipase A2 daboiatoxin B chain (Fragment) OS=Daboia siamensis OX=343250 PE=1 SV=1

Observed	Mr(expt)	Mr(calc)	ppm	Start	End	Miss	Ions	Peptide
1770.7345	1769.7272	1769.8593	-74.65	1	15	0	---	-.MCLIGVEGNLFQFAR.L + Oxidation (M)
1831.7523	1830.7451	1830.8393	-51.45	99	113	1	---	R.VAAICFRENMNTYDK.K
2087.8271	2086.8198	2086.8322	-5.92	115	130	1	---	K.YMLYSIFDCKEESDQC.-
2103.8451	2102.8378	2102.8271	5.10	115	130	1	---	K.YMLYSIFDCKEESDQC.- + Oxidation (M)
2105.8450	2104.8377	2104.7972	19.3	46	61	1	---	K.DATDRCCFVHDCCYAR.V

No match to: 837.4639, 855.4397, 857.4550, 865.4570, 878.4913, 972.4913, 1010.5079, 1026.4958, 1029.5188, 1040.5171, 1049.5677, 1051.4970, 1055.5324, 1067.4164, 1067.9287, 1071.5325, 1079.5338, 1277.7337, 1389.6057, 1446.6389, 1546.7035, 1572.7099, 1583.6660, 1647.6988, 1724.7416, 1756.7151, 1761.7398, 1781.7648, 1783.7721, 1796.7339, 1812.7353, 1815.7528, 1823.7347, 1826.7583, 1828.7661, 1841.7675, 1843.7419, 1855.7505, 1868.7756, 1870.7489, 1872.7762, 1883.7767, 1886.7924, 1894.7645, 1900.7969, 1903.7666, 1905.7433, 1910.7355, 1914.7927, 1920.8407, 2002.7907, 2017.7975, 2024.7742, 2031.8279, 2042.8409, 2044.8576, 2058.8185, 2072.8250, 2076.8298, 2085.8457, 2089.8493, 2092.8135, 2094.8032, 2114.8469, 2117.8290, 2119.2383, 2120.8392, 2128.9018, 2131.8309, 2133.8534, 2146.8669, 2148.8858, 2149.8462, 2159.8698, 2163.8532, 2174.9196, 2176.8605, 2181.9842, 2190.9033, 2198.9939, 2226.0084, 2233.9062, 2245.0192, 2259.0326, 2271.0323, 2277.8915, 2285.8514, 2305.8763, 2321.8870, 2359.0611, 2699.2368, 2887.3239

10. A0A6G5ZUI9_9SAUR **Mass:** 58303 **Score:** 21 **Expect:** 11 **Matches:** 7
Angiopoietin OS=Vipera anatolica senliki OX=2604287 PE=2 SV=1

Observed	Mr(expt)	Mr(calc)	ppm	Start	End	Miss	Ions	Peptide

865.4570	864.4497	864.4705	-24.08	197 - 203	1	---	R.NSFLEKK.V
1040.5171	1039.5098	1039.4678	40.4	220 - 227	1	---	K.TEKMEMQK.L + Oxidation (M)
1079.5338	1078.5265	1078.5156	10.2	336 - 344	1	---	R.RDGSVDFQR.T
1277.7337	1276.7264	1276.6411	66.8	106 - 115	0	---	K.LENYIQENVR.I
2017.7975	2016.7903	2016.9146	-61.65	318 - 335	0	---	K.ALCDMETSGGGWTVIQHR.R
2105.8450	2104.8377	2105.0025	-78.29	413 - 433	1	---	R.YSGTAGRTSSLSPSGTNFSTK.D
2119.2383	2118.2310	2118.0415	89.5	89 - 105	0	---	R.LQQLETVMENNTQWLQK.L + Oxidation (M)

No match to: 837.4639, 855.4397, 857.4550, 878.4913, 972.4913, 1010.5079, 1026.4958, 1029.5188, 1049.5677, 1051.4970, 1055.5324, 1067.4164, 1067.9287, 1071.5325, 1389.6057, 1446.6389, 1546.7035, 1572.7099, 1583.6660, 1647.6988, 1724.7416, 1756.7151, 1761.7398, 1770.7345, 1781.7648, 1783.7721, 1796.7339, 1812.7353, 1815.7528, 1823.7347, 1826.7583, 1828.7661, 1831.7523, 1841.7675, 1843.7419, 1855.7505, 1868.7756, 1870.7489, 1872.7762, 1883.7767, 1886.7924, 1894.7645, 1900.7969, 1903.7666, 1905.7433, 1910.7355, 1914.7927, 1920.8407, 2002.7907, 2024.7742, 2031.8279, 2042.8409, 2044.8576, 2058.8185, 2072.8250, 2076.8298, 2085.8457, 2087.8271, 2089.8493, 2092.8135, 2094.8032, 2103.8451, 2114.8469, 2117.8290, 2120.8392, 2128.9018, 2131.8309, 2133.8534, 2146.8669, 2148.8858, 2149.8462, 2159.8698, 2163.8532, 2174.9196, 2176.8605, 2181.9842, 2190.9033, 2198.9939, 2226.0084, 2233.9062, 2245.0192, 2259.0326, 2271.0323, 2277.8915, 2285.8514, 2305.8763, 2321.8870, 2359.0611, 2699.2368, 2887.3239

11. M4T472_9PERO **Mass:** 26404 **Score:** 20 **Expect:** 15 **Matches:** 5
Ring finger protein 213 (Fragment) OS=Echiichthys vipera OX=94984 PE=4 SV=1

Observed	Mr(expt)	Mr(calc)	ppm	Start - End	Miss	Ions	Peptide
837.4639	836.4566	836.4214	42.1	123 - 128	0	---	K.FLCELR.R
1029.5188	1028.5115	1028.4709	39.5	221 - 228	0	---	K.HTDEMIQR.L
1389.6057	1388.5985	1388.7089	-79.49	50 - 61	0	---	R.VPFNIDFDGLPR.G
1894.7645	1893.7572	1893.9263	-89.31	101 - 117	1	---	R.FRCGIPVIIMGETGCGK.T
2233.9062	2232.8989	2233.0620	-73.02	200 - 219	0	---	K.GESLTPNCGLQIIAACNPYR.K

No match to: 855.4397, 857.4550, 865.4570, 878.4913, 972.4913, 1010.5079, 1026.4958, 1040.5171, 1049.5677, 1051.4970, 1055.5324, 1067.4164, 1067.9287, 1071.5325, 1079.5338, 1277.7337, 1446.6389, 1546.7035, 1572.7099, 1583.6660, 1647.6988, 1724.7416, 1756.7151, 1761.7398, 1770.7345, 1781.7648, 1783.7721, 1796.7339, 1812.7353, 1815.7528, 1823.7347, 1826.7583, 1828.7661, 1831.7523, 1841.7675, 1843.7419, 1855.7505, 1868.7756, 1870.7489, 1872.7762, 1883.7767, 1886.7924, 1900.7969, 1903.7666, 1905.7433, 1910.7355, 1914.7927, 1920.8407, 2002.7907, 2017.7975, 2024.7742, 2031.8279, 2042.8409, 2044.8576, 2058.8185, 2072.8250, 2076.8298, 2085.8457, 2087.8271, 2089.8493, 2092.8135, 2094.8032, 2103.8451, 2105.8450, 2114.8469, 2117.8290, 2119.2383, 2120.8392, 2128.9018, 2131.8309, 2133.8534, 2146.8669, 2148.8858, 2149.8462, 2159.8698, 2163.8532, 2174.9196, 2176.8605, 2181.9842, 2190.9033, 2198.9939, 2226.0084, 2245.0192, 2259.0326, 2271.0323, 2277.8915, 2285.8514, 2305.8763, 2321.8870, 2359.0611, 2699.2368, 2887.3239

12. B8QQ51_9PERO **Mass:** 33786 **Score:** 20 **Expect:** 16 **Matches:** 5
RNF213 (Fragment) OS=Echiichthys vipera OX=94984 GN=RNF213 PE=4 SV=1

Observed	Mr(expt)	Mr(calc)	ppm	Start - End	Miss	Ions	Peptide
837.4639	836.4566	836.4214	42.1	164 - 169	0	---	K.FLCELR.R
1029.5188	1028.5115	1028.4709	39.5	262 - 269	0	---	K.HTDEMIQR.L
1389.6057	1388.5985	1388.7089	-79.49	91 - 102	0	---	R.VPFNIDFDGLPR.G
1894.7645	1893.7572	1893.9263	-89.31	142 - 158	1	---	R.FRCGIPVIIMGETGCGK.T
2233.9062	2232.8989	2233.0620	-73.02	241 - 260	0	---	K.GESLTPNCGLQIIAACNPYR.K

No match to: 855.4397, 857.4550, 865.4570, 878.4913, 972.4913, 1010.5079, 1026.4958, 1040.5171, 1049.5677, 1051.4970, 1055.5324, 1067.4164, 1067.9287, 1071.5325, 1079.5338, 1277.7337, 1446.6389, 1546.7035, 1572.7099, 1583.6660, 1647.6988, 1724.7416, 1756.7151, 1761.7398, 1770.7345, 1781.7648, 1783.7721, 1796.7339, 1812.7353, 1815.7528, 1823.7347, 1826.7583, 1828.7661, 1831.7523, 1841.7675, 1843.7419, 1855.7505, 1868.7756, 1870.7489, 1872.7762, 1883.7767, 1886.7924, 1900.7969, 1903.7666, 1905.7433, 1910.7355, 1914.7927, 1920.8407, 2002.7907, 2017.7975, 2024.7742, 2031.8279, 2042.8409, 2044.8576, 2058.8185, 2072.8250, 2076.8298, 2085.8457, 2087.8271, 2089.8493, 2092.8135, 2094.8032, 2103.8451, 2105.8450, 2114.8469, 2117.8290, 2119.2383, 2120.8392, 2128.9018, 2131.8309, 2133.8534, 2146.8669, 2148.8858, 2149.8462, 2159.8698, 2163.8532, 2174.9196, 2176.8605, 2181.9842, 2190.9033, 2198.9939, 2226.0084, 2245.0192, 2259.0326, 2271.0323, 2277.8915, 2285.8514, 2305.8763, 2321.8870, 2359.0611, 2699.2368, 2887.3239

13. A0A286S1C5_9SAUR **Mass:** 39024 **Score:** 19 **Expect:** 20 **Matches:** 5
E3 ubiquitin-protein ligase RAG1 (Fragment) OS=Vipera seoanei OX=110211 GN=RAG1 PE=4 SV=1

Observed	Mr(expt)	Mr(calc)	ppm	Start - End	Miss	Ions	Peptide
1049.5677	1048.5604	1048.4760	80.5	46 - 53	0	---	K.EVFHMSQR.E + Oxidation (M)
1446.6389	1445.6317	1445.7119	-55.49	219 - 230	1	---	K.MSNCKQIHLSTK.V
1572.7099	1571.7027	1571.8419	-88.61	141 - 154	1	---	R.KVSNAPHEAHLLEK.D
2085.8457	2084.8385	2085.0426	-97.89	81 - 98	1	---	K.CHPVHGPVDEETQALLRK.K
2148.8858	2147.8785	2148.0004	-56.78	1 - 20	1	---	-.ANSAKENMTGSLDITDEQPK.A

No match to: 837.4639, 855.4397, 857.4550, 865.4570, 878.4913, 972.4913, 1010.5079, 1026.4958, 1029.5188, 1040.5171, 1051.4970, 1055.5324, 1067.4164, 1067.9287, 1071.5325, 1079.5338, 1277.7337, 1389.6057, 1546.7035, 1583.6660, 1647.6988, 1724.7416, 1756.7151, 1761.7398, 1770.7345, 1781.7648, 1783.7721, 1796.7339, 1812.7353, 1815.7528, 1823.7347, 1826.7583, 1828.7661, 1831.7523, 1841.7675, 1843.7419, 1855.7505, 1868.7756, 1870.7489, 1872.7762, 1883.7767, 1886.7924, 1894.7645, 1900.7969, 1903.7666, 1905.7433, 1910.7355, 1914.7927, 1920.8407, 2002.7907, 2017.7975, 2024.7742, 2031.8279, 2042.8409, 2044.8576, 2058.8185, 2072.8250, 2076.8298, 2087.8271, 2089.8493, 2092.8135, 2094.8032, 2103.8451, 2105.8450, 2114.8469, 2117.8290, 2119.2383, 2120.8392, 2128.9018, 2131.8309, 2133.8534, 2146.8669, 2149.8462, 2159.8698, 2163.8532, 2174.9196, 2176.8605, 2181.9842, 2190.9033, 2198.9939, 2226.0084, 2233.9062, 2245.0192, 2259.0326, 2271.0323, 2277.8915, 2285.8514, 2305.8763, 2321.8870, 2359.0611, 2699.2368, 2887.3239

14. Q6A3C6_VIPAM **Mass:** 16212 **Score:** 17 **Expect:** 26 **Matches:** 3
Ammodytin I2(A) isoform OS=Vipera ammodytes ruffoi OX=246549 PE=2 SV=1

Observed	Mr(expt)	Mr(calc)	ppm	Start - End	Miss	Ions	Peptide
1900.7969	1899.7897	1899.9036	-60.00	107 - 122	1	---	R.VEAICFGENLNTYDKK.Y
1903.7666	1902.7593	1902.7513	4.25	123 - 137	1	---	K.YKNYPSSHCTETEQC.-
2042.8409	2041.8336	2041.8662	-15.97	32 - 49	0	---	K.SALLSYSNYGCYCGWGGK.G

No match to: 837.4639, 855.4397, 857.4550, 865.4570, 878.4913, 972.4913, 1010.5079, 1026.4958, 1029.5188, 1040.5171, 1049.5677, 1051.4970, 1055.5324, 1067.4164, 1067.9287, 1071.5325, 1079.5338, 1277.7337, 1389.6057, 1446.6389, 1546.7035, 1572.7099, 1583.6660, 1647.6988, 1724.7416, 1756.7151, 1761.7398, 1770.7345, 1781.7648, 1783.7721, 1796.7339, 1812.7353, 1815.7528, 1823.7347, 1826.7583, 1828.7661, 1831.7523, 1841.7675, 1843.7419, 1855.7505, 1868.7756, 1870.7489, 1872.7762, 1883.7767, 1886.7924, 1894.7645, 1905.7433, 1910.7355, 1914.7927, 1920.8407, 2002.7907, 2017.7975, 2024.7742, 2031.8279, 2044.8576, 2058.8185, 2072.8250, 2076.8298, 2085.8457, 2087.8271, 2089.8493, 2092.8135, 2094.8032, 2103.8451, 2105.8450, 2114.8469, 2117.8290, 2119.2383, 2120.8392, 2128.9018, 2131.8309, 2133.8534, 2146.8669, 2148.8858, 2149.8462, 2159.8698, 2163.8532, 2174.9196, 2176.8605, 2181.9842, 2190.9033, 2198.9939, 2226.0084, 2233.9062, 2245.0192, 2259.0326, 2271.0323, 2277.8915, 2285.8514, 2305.8763, 2321.8870, 2359.0611, 2699.2368, 2887.3239

15. Q6A3C0_VIPAP **Mass:** 16127 **Score:** 17 **Expect:** 26 **Matches:** 3
Ammodytin I2(B) isoform OS=Vipera aspis aspis OX=194601 PE=2 SV=1

Observed	Mr(expt)	Mr(calc)	ppm	Start - End	Miss	Ions	Peptide
1815.7528	1814.7455	1814.8873	-78.09	107 - 122	1	---	R.VAAICFGENLSTYDKK.Y
1903.7666	1902.7593	1902.7513	4.25	123 - 137	1	---	K.YKNYPSSHCTETEQC.-
2042.8409	2041.8336	2041.8662	-15.97	32 - 49	0	---	K.SALLSYSNYGCYCGWGGK.G

No match to: 837.4639, 855.4397, 857.4550, 865.4570, 878.4913, 972.4913, 1010.5079, 1026.4958, 1029.5188, 1040.5171, 1049.5677, 1051.4970, 1055.5324, 1067.4164, 1067.9287, 1071.5325, 1079.5338, 1277.7337, 1389.6057, 1446.6389, 1546.7035, 1572.7099, 1583.6660, 1647.6988, 1724.7416, 1756.7151, 1761.7398, 1770.7345, 1781.7648, 1783.7721, 1796.7339, 1812.7353, 1823.7347, 1826.7583, 1828.7661, 1831.7523, 1841.7675, 1843.7419, 1855.7505, 1868.7756, 1870.7489, 1872.7762, 1883.7767, 1886.7924, 1894.7645, 1900.7969, 1905.7433, 1910.7355, 1914.7927, 1920.8407, 2002.7907, 2017.7975, 2024.7742, 2031.8279, 2044.8576, 2058.8185, 2072.8250, 2076.8298, 2085.8457, 2087.8271, 2089.8493, 2092.8135, 2094.8032, 2103.8451, 2105.8450, 2114.8469, 2117.8290, 2119.2383, 2120.8392, 2128.9018, 2131.8309, 2133.8534, 2146.8669, 2148.8858, 2149.8462, 2159.8698, 2163.8532, 2174.9196, 2176.8605, 2181.9842, 2190.9033, 2198.9939, 2226.0084, 2233.9062, 2245.0192, 2259.0326, 2271.0323, 2277.8915, 2285.8514, 2305.8763, 2321.8870, 2359.0611, 2699.2368, 2887.3239

16. Q6A3B5_VIPAS **Mass:** 16127 **Score:** 17 **Expect:** 26 **Matches:** 3
Ammodytin I2(B) isoform OS=Vipera aspis atra OX=246182 PE=2 SV=1

Observed	Mr(expt)	Mr(calc)	ppm	Start - End	Miss	Ions	Peptide
1815.7528	1814.7455	1814.8873	-78.09	107 - 122	1	---	R.VAAICFGENLSTYDKK.Y
1903.7666	1902.7593	1902.7513	4.25	123 - 137	1	---	K.YKNYPSSHCTETEQC.-
2042.8409	2041.8336	2041.8662	-15.97	32 - 49	0	---	K.SALLSYSNYGCYCGWGGK.G

No match to: 837.4639, 855.4397, 857.4550, 865.4570, 878.4913, 972.4913, 1010.5079, 1026.4958, 1029.5188, 1040.5171, 1049.5677, 1051.4970, 1055.5324, 1067.4164, 1067.9287, 1071.5325, 1079.5338, 1277.7337, 1389.6057, 1446.6389, 1546.7035, 1572.7099, 1583.6660, 1647.6988, 1724.7416, 1756.7151, 1761.7398, 1770.7345, 1781.7648, 1783.7721, 1796.7339, 1812.7353, 1823.7347, 1826.7583, 1828.7661, 1831.7523, 1841.7675, 1843.7419, 1855.7505, 1868.7756, 1870.7489, 1872.7762, 1883.7767, 1886.7924, 1894.7645, 1900.7969, 1905.7433, 1910.7355, 1914.7927, 1920.8407, 2002.7907, 2017.7975, 2024.7742, 2031.8279, 2044.8576, 2058.8185, 2072.8250, 2076.8298, 2085.8457, 2087.8271, 2089.8493, 2092.8135, 2094.8032, 2103.8451, 2105.8450, 2114.8469, 2117.8290, 2119.2383, 2120.8392, 2128.9018, 2131.8309, 2133.8534, 2146.8669, 2148.8858, 2149.8462, 2159.8698, 2163.8532, 2174.9196, 2176.8605, 2181.9842, 2190.9033, 2198.9939, 2226.0084, 2233.9062, 2245.0192, 2259.0326, 2271.0323, 2277.8915, 2285.8514, 2305.8763, 2321.8870, 2359.0611, 2699.2368, 2887.3239

17. Q6A3B9_VIPAP **Mass:** 16093 **Score:** 17 **Expect:** 26 **Matches:** 3
Ammodytin I2(B) variant OS=Vipera aspis aspis OX=194601 PE=2 SV=1

Observed	Mr(expt)	Mr(calc)	ppm	Start - End	Miss	Ions	Peptide
1815.7528	1814.7455	1814.8873	-78.09	107 - 122	1	---	R.VAAICFGENLSTYDKK.Y
1903.7666	1902.7593	1902.7513	4.25	123 - 137	1	---	K.YKNYPSSHCTETEQC.-
2042.8409	2041.8336	2041.8662	-15.97	32 - 49	0	---	K.SALLSYSNYGCYCGWGGK.G

No match to: 837.4639, 855.4397, 857.4550, 865.4570, 878.4913, 972.4913, 1010.5079, 1026.4958, 1029.5188, 1040.5171, 1049.5677, 1051.4970, 1055.5324, 1067.4164, 1067.9287, 1071.5325, 1079.5338, 1277.7337, 1389.6057, 1446.6389, 1546.7035, 1572.7099, 1583.6660, 1647.6988, 1724.7416, 1756.7151, 1761.7398, 1770.7345, 1781.7648, 1783.7721, 1796.7339, 1812.7353, 1823.7347, 1826.7583, 1828.7661, 1831.7523, 1841.7675, 1843.7419, 1855.7505, 1868.7756, 1870.7489, 1872.7762, 1883.7767, 1886.7924, 1894.7645, 1900.7969, 1905.7433, 1910.7355, 1914.7927, 1920.8407, 2002.7907, 2017.7975, 2024.7742, 2031.8279, 2044.8576, 2058.8185,

2072.8250, 2076.8298, 2085.8457, 2087.8271, 2089.8493, 2092.8135, 2094.8032, 2103.8451, 2105.8450, 2114.8469, 2117.8290, 2119.2383, 2120.8392, 2128.9018, 2131.8309, 2133.8534, 2146.8669, 2148.8858, 2149.8462, 2159.8698, 2163.8532, 2174.9196, 2176.8605, 2181.9842, 2190.9033, 2198.9939, 2226.0084, 2233.9062, 2245.0192, 2259.0326, 2271.0323, 2277.8915, 2285.8514, 2305.8763, 2321.8870, 2359.0611, 2699.2368, 2887.3239

18. Q6A3B7_VIPAP **Mass:** 16099 **Score:** 17 **Expect:** 26 **Matches:** 3

Ammodytin I2(B) variant OS=Vipera aspis aspis OX=194601 PE=2 SV=1

Observed	Mr(expt)	Mr(calc)	ppm	Start	End	Miss	Ions	Peptide
1815.7528	1814.7455	1814.8873	-78.09	107 -	122	1	---	R.VAAICFGENLSTYDKK.Y
1903.7666	1902.7593	1902.7513	4.25	123 -	137	1	---	K.YKNYPSSHCTETEQC.-
2042.8409	2041.8336	2041.8662	-15.97	32 -	49	0	---	K.SALLSYSNYGCYCGWGGK.G

No match to: 837.4639, 855.4397, 857.4550, 865.4570, 878.4913, 972.4913, 1010.5079, 1026.4958, 1029.5188, 1040.5171, 1049.5677, 1051.4970, 1055.5324, 1067.4164, 1067.9287, 1071.5325, 1079.5338, 1277.7337, 1389.6057, 1446.6389, 1546.7035, 1572.7099, 1583.6660, 1647.6988, 1724.7416, 1756.7151, 1761.7398, 1770.7345, 1781.7648, 1783.7721, 1796.7339, 1812.7353, 1823.7347, 1826.7583, 1828.7661, 1831.7523, 1841.7675, 1843.7419, 1855.7505, 1868.7756, 1870.7489, 1872.7762, 1883.7767, 1886.7924, 1894.7645, 1900.7969, 1905.7433, 1910.7355, 1914.7927, 1920.8407, 2002.7907, 2017.7975, 2024.7742, 2031.8279, 2044.8576, 2058.8185, 2072.8250, 2076.8298, 2085.8457, 2087.8271, 2089.8493, 2092.8135, 2094.8032, 2103.8451, 2105.8450, 2114.8469, 2117.8290, 2119.2383, 2120.8392, 2128.9018, 2131.8309, 2133.8534, 2146.8669, 2148.8858, 2149.8462, 2159.8698, 2163.8532, 2174.9196, 2176.8605, 2181.9842, 2190.9033, 2198.9939, 2226.0084, 2233.9062, 2245.0192, 2259.0326, 2271.0323, 2277.8915, 2285.8514, 2305.8763, 2321.8870, 2359.0611, 2699.2368, 2887.3239

19. W8EEP5_MACLB **Mass:** 41360 **Score:** 17 **Expect:** 30 **Matches:** 6

Hyaluronidase (Fragment) OS=Macrovipera lebetina OX=8709 PE=2 SV=1

Observed	Mr(expt)	Mr(calc)	ppm	Start	End	Miss	Ions	Peptide
837.4639	836.4566	836.4392	20.9	78 -	84	1	---	K.QYEKAAK.S
1051.4970	1050.4897	1050.5280	-36.46	245 -	253	1	---	K.KYMNGPLGR.Y + Oxidation (M)
1079.5338	1078.5265	1078.6022	-70.18	254 -	263	0	---	R.YIVNVTTAAK.I
1770.7345	1769.7272	1769.7713	-24.90	120 -	134	1	---	K.GDQYTGKCPEIEMSR.N
1903.7666	1902.7593	1902.9013	-74.60	280 -	295	0	---	K.HSDSNAFLHLFPESFR.I
2031.8279	2030.8206	2030.9962	-86.49	279 -	295	1	---	R.KHSDSNAFLHLFPESFR.I

No match to: 855.4397, 857.4550, 865.4570, 878.4913, 972.4913, 1010.5079, 1026.4958, 1029.5188, 1040.5171, 1049.5677, 1055.5324, 1067.4164, 1067.9287, 1071.5325, 1277.7337, 1389.6057, 1446.6389, 1546.7035, 1572.7099, 1583.6660, 1647.6988, 1724.7416, 1756.7151, 1761.7398, 1781.7648, 1783.7721, 1796.7339, 1812.7353, 1815.7528, 1823.7347, 1826.7583, 1828.7661, 1831.7523, 1841.7675, 1843.7419, 1855.7505, 1868.7756, 1870.7489, 1872.7762, 1883.7767, 1886.7924, 1894.7645, 1900.7969, 1905.7433, 1910.7355, 1914.7927, 1920.8407, 2002.7907, 2017.7975, 2024.7742, 2042.8409, 2044.8576, 2058.8185, 2072.8250, 2076.8298, 2085.8457, 2087.8271, 2089.8493, 2092.8135, 2094.8032, 2103.8451, 2105.8450, 2114.8469, 2117.8290, 2119.2383, 2120.8392, 2128.9018, 2131.8309, 2133.8534, 2146.8669, 2148.8858, 2149.8462, 2159.8698, 2163.8532, 2174.9196, 2176.8605, 2181.9842, 2190.9033, 2198.9939, 2226.0084, 2233.9062, 2245.0192, 2259.0326, 2271.0323, 2277.8915, 2285.8514, 2305.8763, 2321.8870, 2359.0611, 2699.2368, 2887.3239

20. VKT2_DABSI **Mass:** 7187 **Score:** 16 **Expect:** 38 **Matches:** 2

Kunitz-type serine protease inhibitor 2 OS=Daboia siamensis OX=343250 PE=1 SV=1

Observed	Mr(expt)	Mr(calc)	ppm	Start	End	Miss	Ions	Peptide
1756.7151	1755.7078	1755.8111	-58.84	1 -	15	0	---	-.HDRPTFCNLAPESGR.C
2072.8250	2071.8177	2071.9429	-60.39	1 -	17	1	---	-.HDRPTFCNLAPESGRCR.G

No match to: 837.4639, 855.4397, 857.4550, 865.4570, 878.4913, 972.4913, 1010.5079, 1026.4958, 1029.5188, 1040.5171, 1049.5677, 1051.4970, 1055.5324, 1067.4164, 1067.9287, 1071.5325, 1079.5338, 1277.7337, 1389.6057, 1446.6389, 1546.7035, 1572.7099, 1583.6660, 1647.6988, 1724.7416, 1761.7398, 1770.7345, 1781.7648, 1783.7721, 1796.7339, 1812.7353, 1815.7528, 1823.7347, 1826.7583, 1828.7661, 1831.7523, 1841.7675, 1843.7419, 1855.7505, 1868.7756, 1870.7489, 1872.7762, 1883.7767, 1886.7924, 1894.7645, 1900.7969, 1903.7666, 1905.7433, 1910.7355, 1914.7927, 1920.8407, 2002.7907, 2017.7975, 2024.7742, 2031.8279, 2042.8409, 2044.8576, 2058.8185, 2076.8298, 2085.8457, 2087.8271, 2089.8493, 2092.8135, 2094.8032, 2103.8451, 2105.8450, 2114.8469, 2117.8290, 2119.2383, 2120.8392, 2128.9018, 2131.8309, 2133.8534, 2146.8669, 2148.8858, 2149.8462, 2159.8698, 2163.8532, 2174.9196, 2176.8605, 2181.9842, 2190.9033, 2198.9939, 2226.0084, 2233.9062, 2245.0192, 2259.0326, 2271.0323, 2277.8915, 2285.8514, 2305.8763, 2321.8870, 2359.0611, 2699.2368, 2887.3239

Search Parameters

**Type of search**	**: MS/MS Ion Search**
**Enzyme**	**: Trypsin**
**Fixed modifications**	**: Carbamidomethyl (C)**
**Variable modifications**	**: Oxidation (M)**
**Mass values**	**: Monoisotopic**
**Protein Mass**	**: Unrestricted**
**Peptide Mass Tolerance**	**: ± 100 ppm**
**Fragment Mass Tolerance**	**: ± 0.7 Da**
**Max Missed Cleavages**	**: 1**
**Instrument type**	**: MALDI-TOF-TOF**
**Query1 (837.4639,1+)**	**: \\PROTEOMICS1\D\DATA\2105\210505_TLIEPOL_1_68252-68266\68252_68266_LPARDO\0_H7\1\837.4639.LIFT\1SREF\PDATA\1\1R**
**Query2 (855.4397,1+)**	**: <no title>**
**Query3 (857.4550,1+)**	**: <no title>**
**Query4 (865.4570,1+)**	**: <no title>**
**Query5 (878.4913,1+)**	**: <no title>**
**Query6 (972.4913,1+)**	**: <no title>**
**Query7 (1010.5079,1+)**	**: <no title>**
**Query8 (1026.4958,1+)**	**: <no title>**
**Query9 (1029.5188,1+)**	**: \\PROTEOMICS1\D\DATA\2105\210505_TLIEPOL_1_68252-68266\68252_68266_LPARDO\0_H7\1\1029.5188.LIFT\1SREF\PDATA\1\1R**
**Query10 (1040.5171,1+)**	**: <no title>**
**Query11 (1049.5677,1+)**	**: <no title>**
**Query12 (1051.4970,1+)**	**: \\PROTEOMICS1\D\DATA\2105\210505_TLIEPOL_1_68252-68266\68252_68266_LPARDO\0_H7\1\1051.4970.LIFT\1SREF\PDATA\1\1R**
**Query13 (1055.5324,1+)**	**: <no title>**
**Query14 (1067.4164,1+)**	**: <no title>**
**Query15 (1067.9287,1+)**	**: <no title>**
**Query16 (1071.5325,1+)**	**: <no title>**
**Query17 (1079.5338,1+)**	**: <no title>**
**Query18 (1277.7337,1+)**	**: <no title>**
**Query19 (1389.6057,1+)**	**: <no title>**
**Query20 (1446.6389,1+)**	**: <no title>**
**Query21 (1546.7035,1+)**	**: <no title>**
**Query22 (1572.7099,1+)**	**: <no title>**
**Query23 (1583.6660,1+)**	**: <no title>**
**Query24 (1647.6988,1+)**	**: <no title>**
**Query25 (1724.7416,1+)**	**: <no title>**
**Query26 (1756.7151,1+)**	**: <no title>**
**Query27 (1761.7398,1+)**	**: \\PROTEOMICS1\D\DATA\2105\210505_TLIEPOL_1_68252-68266\68252_68266_LPARDO\0_H7\1\1761.7398.LIFT\1SREF\PDATA\1\1R**
**Query28 (1770.7345,1+)**	**: <no title>**
**Query29 (1781.7648,1+)**	**: <no title>**
**Query30 (1783.7721,1+)**	**: <no title>**
**Query31 (1796.7339,1+)**	**: <no title>**
**Query32 (1812.7353,1+)**	**: <no title>**
**Query33 (1815.7528,1+)**	**: <no title>**
**Query34 (1823.7347,1+)**	**: <no title>**
**Query35 (1826.7583,1+)**	**: \\PROTEOMICS1\D\DATA\2105\210505_TLIEPOL_1_68252-68266\68252_68266_LPARDO\0_H7\1\1826.7583.LIFT\1SREF\PDATA\1\1R**
**Query36 (1828.7661,1+)**	**: <no title>**
**Query37 (1831.7523,1+)**	**: <no title>**
**Query38 (1841.7675,1+)**	**: <no title>**
**Query39 (1843.7419,1+)**	**: <no title>**
**Query40 (1855.7505,1+)**	**: <no title>**
**Query41 (1868.7756,1+)**	**: <no title>**
**Query42 (1870.7489,1+)**	**: <no title>**
**Query43 (1872.7762,1+)**	**: \\PROTEOMICS1\D\DATA\2105\210505_TLIEPOL_1_68252-68266\68252_68266_LPARDO\0_H7\1\1872.7762.LIFT\1SREF\PDATA\1\1R**
**Query44 (1883.7767,1+)**	**: <no title>**
**Query45 (1886.7924,1+)**	**: \\PROTEOMICS1\D\DATA\2105\210505_TLIEPOL_1_68252-68266\68252_68266_LPARDO\0_H7\1\1886.7924.LIFT\1SREF\PDATA\1\1R**
**Query46 (1894.7645,1+)**	**: <no title>**
**Query47 (1900.7969,1+)**	**: <no title>**
**Query48 (1903.7666,1+)**	**: <no title>**
**Query49 (1905.7433,1+)**	**: <no title>**
**Query50 (1910.7355,1+)**	**: <no title>**
**Query51 (1914.7927,1+)**	**: <no title>**
**Query52 (1920.8407,1+)**	**: <no title>**
**Query53 (2002.7907,1+)**	**: <no title>**
**Query54 (2017.7975,1+)**	**: <no title>**
**Query55 (2024.7742,1+)**	**: <no title>**
**Query56 (2031.8279,1+)**	**: <no title>**
**Query57 (2042.8409,1+)**	**: <no title>**
**Query58 (2044.8576,1+)**	**: <no title>**
**Query59 (2058.8185,1+)**	**: <no title>**
**Query60 (2072.8250,1+)**	**: <no title>**
**Query61 (2076.8298,1+)**	**: <no title>**
**Query62 (2085.8457,1+)**	**: <no title>**
**Query63 (2087.8271,1+)**	**: <no title>**

```
Query64 (2089.8493,1+) : <no title>
Query65 (2092.8135,1+) : <no title>
Query66 (2094.8032,1+) : <no title>
Query67 (2103.8451,1+) : <no title>
Query68 (2105.8450,1+) : <no title>
Query69 (2114.8469,1+) : <no title>
Query70 (2117.8290,1+) : <no title>
Query71 (2119.2383,1+) : <no title>
Query72 (2120.8392,1+) : <no title>
Query73 (2128.9018,1+) : <no title>
Query74 (2131.8309,1+) : <no title>
Query75 (2133.8534,1+) : \\PROTEOMICS1\D\DATA\2105\210505_TLIEPOL_1_68252-68266\68252_68266_LPARDO\0_H7\1\2133.8534.LIFT\1SREF\PDATA\1\1R
Query76 (2146.8669,1+) : <no title>
Query77 (2148.8858,1+) : <no title>
Query78 (2149.8462,1+) : \\PROTEOMICS1\D\DATA\2105\210505_TLIEPOL_1_68252-68266\68252_68266_LPARDO\0_H7\1\2149.8462.LIFT\1SREF\PDATA\1\1R
Query79 (2159.8698,1+) : <no title>
Query80 (2163.8532,1+) : <no title>
Query81 (2174.9196,1+) : <no title>
Query82 (2176.8605,1+) : <no title>
Query83 (2181.9842,1+) : <no title>
Query84 (2190.9033,1+) : <no title>
Query85 (2198.9939,1+) : <no title>
Query86 (2226.0084,1+) : <no title>
Query87 (2233.9062,1+) : <no title>
Query88 (2245.0192,1+) : \\PROTEOMICS1\D\DATA\2105\210505_TLIEPOL_1_68252-68266\68252_68266_LPARDO\0_H7\1\2245.0192.LIFT\1SREF\PDATA\1\1R
Query89 (2259.0326,1+) : <no title>
Query90 (2271.0323,1+) : <no title>
Query91 (2277.8915,1+) : <no title>
Query92 (2285.8514,1+) : <no title>
Query93 (2305.8763,1+) : \\PROTEOMICS1\D\DATA\2105\210505_TLIEPOL_1_68252-68266\68252_68266_LPARDO\0_H7\1\2305.8763.LIFT\1SREF\PDATA\1\1R
Query94 (2321.8870,1+) : <no title>
Query95 (2359.0611,1+) : <no title>
Query96 (2699.2368,1+) : <no title>
Query97 (2887.3239,1+) : <no title>
```

**Mascot:** http://www.matrixscience.com/

Mascot Search Results **Fr4-2**

```
User          :
Email         :
Search title  : vipera_All_8IDplus4 file:\\Proteomics1\d\data\2105\210505_tliepol_1_68252-68266\68252_68266_lpardo\0_G8\1\1SRef\pdata\1\peaklist.xml
Database      : vipera  (1414 sequences; 347452 residues)
Timestamp     : 5 May 2021 at 09:34:32 GMT
Top Score     : 88 for DID5B_MACLO, Disintegrin VLO5B OS=Macrovipera lebetina obtusa OX=209528 PE=1 SV=1
```

Mascot Score Histogram

Protein score is -10*Log(P), where P is the probability that the observed match is a random event.
Protein scores greater than 44 are significant (p<0.05).

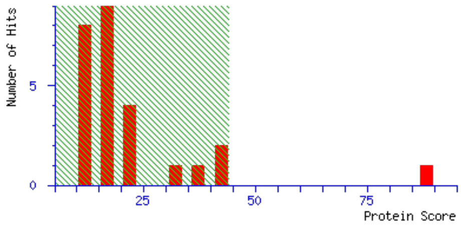

Concise Protein Summary Report

Format As | Concise Protein Summary | Help

Significance threshold p< 0.05 Max. number of hits 10

Re-Search All | Search Unmatched

1. DID5B_MACLO **Mass:** 8235 **Score:** **88** **Expect:** 2.1e-006 **Matches:** 8
Disintegrin VLO5B OS=Macrovipera lebetina obtusa OX=209528 PE=1 SV=1

---

2. A0A1I9KNR7_VIPAA **Mass:** 14615 **Score:** 43 **Expect:** 0.073 **Matches:** 4
Disintegrin Dis-1 OS=Vipera ammodytes ammodytes OX=8705 PE=2 SV=1
A0A1I9KNR4_VIPAA **Mass:** 14601 **Score:** 43 **Expect:** 0.073 **Matches:** 4
Disintegrin Dis-1 OS=Vipera ammodytes ammodytes OX=8705 PE=2 SV=1

---

3. DID2A_MACLB **Mass:** 14638 **Score:** 43 **Expect:** 0.073 **Matches:** 5
Disintegrin lebein-2-alpha OS=Macrovipera lebetina OX=8709 PE=1 SV=1

---

4. Q1JRG9_MACLN **Mass:** 7698 **Score:** 36 **Expect:** 0.36 **Matches:** 5
VGD-containing dimeric disintegrin subunit ML-G1 (Fragment) OS=Macrovipera lebetina transmediterranea OX=384075 GN=ml-G1 PE=4 SV=1

---

5. DID1A_MACLB **Mass:** 12718 **Score:** 34 **Expect:** 0.62 **Matches:** 4
Disintegrin lebein-1-alpha OS=Macrovipera lebetina OX=8709 PE=1 SV=2
DIDB_CERVI **Mass:** 7584 **Score:** 26 **Expect:** 3.9 **Matches:** 3
Disintegrin CV-11-beta (Fragment) OS=Cerastes vipera OX=8698 PE=2 SV=1
DID1B_MACLB **Mass:** 7686 **Score:** 24 **Expect:** 5.4 **Matches:** 3
Disintegrin lebein-1-beta OS=Macrovipera lebetina OX=8709 PE=1 SV=1
VM2A6_VIPAA **Mass:** 7588 **Score:** 24 **Expect:** 6.3 **Matches:** 3
Disintegrin VA6 OS=Vipera ammodytes ammodytes OX=8705 PE=1 SV=1
DID7A_VIPBB **Mass:** 7574 **Score:** 24 **Expect:** 6.3 **Matches:** 3
Disintegrin VB7A OS=Vipera berus berus OX=31156 PE=1 SV=1
A0A6G5ZVR7_9SAUR **Mass:** 9593 **Score:** 22 **Expect:** 9.8 **Matches:** 3
Disintegrin (Fragment) OS=Vipera anatolica senliki OX=2604287 PE=2 SV=1
A0A1J0CZN0_VIPAA **Mass:** 12765 **Score:** 20 **Expect:** 15 **Matches:** 3
Dis-2 OS=Vipera ammodytes ammodytes OX=8705 PE=2 SV=1

---

6. A0A6G5ZVT3_9SAUR **Mass:** 34153 **Score:** 22 **Expect:** 8.5 **Matches:** 3
Tankyrase 3 (Fragment) OS=Vipera anatolica senliki OX=2604287 PE=2 SV=1

---

7. Q6EIM1_VIPAP **Mass:** 36760 **Score:** 20 **Expect:** 13 **Matches:** 3
NADH-ubiquinone oxidoreductase chain 2 (Fragment) OS=Vipera aspis aspis OX=194601 GN=ND2 PE=3 SV=1
Q6EIM6_VIPAP **Mass:** 35276 **Score:** 12 **Expect:** 89 **Matches:** 2
NADH-ubiquinone oxidoreductase chain 2 (Fragment) OS=Vipera aspis aspis OX=194601 GN=ND2 PE=3 SV=1
Q671B8_VIPAP **Mass:** 36585 **Score:** 12 **Expect:** 96 **Matches:** 2
NADH-ubiquinone oxidoreductase chain 2 (Fragment) OS=Vipera aspis aspis OX=194601 GN=ND2 PE=3 SV=1
Q671B2_VIPAZ **Mass:** 36800 **Score:** 12 **Expect:** 96 **Matches:** 2
NADH-ubiquinone oxidoreductase chain 2 (Fragment) OS=Vipera aspis zinnikeri OX=55427 GN=ND2 PE=3 SV=1
Q6EIM4_VIPAP **Mass:** 37194 **Score:** 12 **Expect:** 98 **Matches:** 2
NADH-ubiquinone oxidoreductase chain 2 (Fragment) OS=Vipera aspis aspis OX=194601 GN=ND2 PE=3 SV=1
Q6EIN1_VIPAZ **Mass:** 37200 **Score:** 12 **Expect:** 98 **Matches:** 2
NADH-ubiquinone oxidoreductase chain 2 (Fragment) OS=Vipera aspis zinnikeri OX=55427 GN=ND2 PE=3 SV=1
Q6EIM9_VIPAS **Mass:** 37495 **Score:** 12 **Expect:** 98 **Matches:** 2
NADH-ubiquinone oxidoreductase chain 2 (Fragment) OS=Vipera aspis hugyi OX=246180 GN=ND2 PE=3 SV=1
Q6EIM5_VIPAS **Mass:** 37421 **Score:** 12 **Expect:** 98 **Matches:** 2
NADH-ubiquinone oxidoreductase chain 2 (Fragment) OS=Vipera aspis atra OX=246182 GN=ND2 PE=3 SV=1
Q6EIM8_VIPAS **Mass:** 37801 **Score:** 12 **Expect:** 98 **Matches:** 2
NADH-ubiquinone oxidoreductase chain 2 (Fragment) OS=Vipera aspis francisciredi OX=246181 GN=ND2 PE=3 SV=1
Q671A8_VIPAS **Mass:** 37687 **Score:** 12 **Expect:** 98 **Matches:** 2
NADH-ubiquinone oxidoreductase chain 2 (Fragment) OS=Vipera aspis atra OX=246182 GN=ND2 PE=3 SV=1
Q671C1_VIPAP **Mass:** 37609 **Score:** 12 **Expect:** 98 **Matches:** 2
NADH-ubiquinone oxidoreductase chain 2 (Fragment) OS=Vipera aspis aspis OX=194601 GN=ND2 PE=3 SV=1
Q671A5_VIPAP **Mass:** 37593 **Score:** 12 **Expect:** 98 **Matches:** 2
NADH-ubiquinone oxidoreductase chain 2 (Fragment) OS=Vipera aspis aspis OX=194601 GN=ND2 PE=3 SV=1
B9ZU79_VIPAS **Mass:** 37956 **Score:** 12 **Expect:** 1e+002 **Matches:** 2
NADH-ubiquinone oxidoreductase chain 2 OS=Vipera aspis francisciredi OX=246181 GN=ND2 PE=3 SV=1
B9ZUH9_VIPAS **Mass:** 37913 **Score:** 12 **Expect:** 1e+002 **Matches:** 2

NADH-ubiquinone oxidoreductase chain 2 OS=Vipera aspis hugyi OX=246180 GN=ND2 PE=3 SV=1

---

8. A0A6G5ZUJ2_9SAUR **Mass:** 122875 **Score:** 20 **Expect:** 13 **Matches:** 6
Poly [ADP-ribose] polymerase OS=Vipera anatolica senliki OX=2604287 PE=2 SV=1
A0A6G5ZW66_9SAUR **Mass:** 129763 **Score:** 20 **Expect:** 16 **Matches:** 6
Poly [ADP-ribose] polymerase OS=Vipera anatolica senliki OX=2604287 PE=2 SV=1

---

9. A0A223PK45_DABRR **Mass:** 89589 **Score:** 20 **Expect:** 15 **Matches:** 9
Group 6 secretory phospholipase A2 (Fragment) OS=Daboia russelii OX=8707 PE=2 SV=1

---

10. PA2A1_VIPRE **Mass:** 16228 **Score:** 19 **Expect:** 20 **Matches:** 3
Acidic phospholipase A2 PL1 OS=Vipera renardi OX=927686 PE=2 SV=1
A0A6G5ZVQ9_9SAUR **Mass:** 46045 **Score:** 12 **Expect:** 98 **Matches:** 2
Lipase OS=Vipera anatolica senliki OX=2604287 PE=2 SV=1

Search Parameters

Type of search : Peptide Mass Fingerprint
Enzyme : Trypsin
Fixed modifications : Carbamidomethyl (C)
Variable modifications : Oxidation (M)
Mass values : Monoisotopic
Protein Mass : Unrestricted
Peptide Mass Tolerance : ± 100 ppm
Peptide Charge State : 1+
Max Missed Cleavages : 1
Number of queries : 98
Selected for scoring : 30

Mascot: http://www.matrixscience.com/

MATRIX SCIENCE Mascot Search Results

```
User          :
Email         :
Search title  : vipera_all_Lift file:\\Proteomics1\d\data\2105\210505_tliepol_1_68252-68266\68252_68266_lpardo\0_G8\1\0_G8.xml
Database      : vipera  (1414 sequences; 347452 residues)
Timestamp     : 5 May 2021 at 09:48:41 GMT
Protein hits  : DID5B_MACLO       Disintegrin VLO5B OS=Macrovipera lebetina obtusa OX=209528 PE=1 SV=1
                A0A1I9KNR4_VIPAA  Disintegrin Dis-1 OS=Vipera ammodytes ammodytes OX=8705 PE=2 SV=1
                A0A223PK18_DABRR  C-type lectin domain family 16 member A (Fragment) OS=Daboia russelii OX=8707 PE=2 SV=1
                Q8UVG8_DABRR      Vimentin OS=Daboia russelii OX=8707 PE=2 SV=1
                A0A223PK34_DABRR  Guanylate cyclase (Fragment) OS=Daboia russelii OX=8707 PE=2 SV=1
                A0A6G5ZUB2_9SAUR  Disintegrin and metalloproteinase domain-containing protein 9 (Fragment) OS=Vipera anatolica senliki OX=2604287 PE=2 SV=1
                A0A6G5ZVV3_9SAUR  Vascular endothelial growth factor 2 OS=Vipera anatolica senliki OX=2604287 PE=2 SV=1
                A0A161KGM7_VIPUR  Prolactin receptor (Fragment) OS=Vipera ursinii macrops OX=927675 GN=PRLR PE=4 SV=1
                B8QCD1_DABRR      Endothelin converting enzyme-like protein 1 (Fragment) OS=Daboia russelii OX=8707 GN=ECEL1 PE=4 SV=1
```

Mascot Score Histogram

Ions score is -10*Log(P), where P is the probability that the observed match is a random event.
Individual ions scores > 13 indicate identity or extensive homology (p<0.05).
Protein scores are derived from ions scores as a non-probabilistic basis for ranking protein hits.

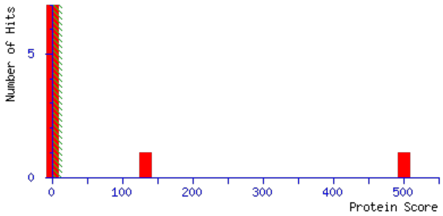

Peptide Summary Report

Format As | Peptide Summary | Help

Significance threshold p< 0.05 | Max. number of hits 10

Standard scoring ○ MudPIT scoring ◉ | Ions score or expect cut-off 0 | Show sub-sets 0

Show pop-ups ◉ Suppress pop-ups ○ | Sort unassigned Decreasing Score | Require bold red ☐

Overview Table

Click on column header to jump to entry in results list.
Move mouse over any indicator to highlight identical peptides.
Click on an indicator to see details of individual match.
Use check boxes to select sub-set of queries for new search.

Mouse over: -Query- | -Accession- | -Sequence-

Hit:	1	2	3	4	5	6	7	8	9
☑ 992.5466 (1+)									
☑ 1030.5622 (1+)									
☑ 1049.5703 (1+)	●	●							
☑ 1063.5850 (1+)									
☑ 1075.5835 (1+)									
☑ 1091.5833 (1+)									
☑ 1148.6528 (1+)									
☑ 1174.5232 (1+)									
☑ 1186.6320 (1+)									
☑ 1203.6590 (1+)									
☑ 1205.6684 (1+)	●	●	●			●			●
☑ 1229.6714 (1+)									
☑ 1246.6979 (1+)									
☑ 1261.5132 (1+)									
☑ 1262.6952 (1+)									
☑ 1318.5360 (1+)	●	●					●		
☑ 1320.6106 (1+)									
☑ 1355.6158 (1+)									
☑ 1368.6087 (1+)									
☑ 1382.6064 (1+)									
☑ 1389.6115 (1+)									
☑ 1415.6205 (1+)									
☑ 1427.6237 (1+)									
☑ 1439.6265 (1+)									
☑ 1443.6212 (1+)									
☑ 1446.6351 (1+)		●							
☑ 1457.6356 (1+)									
☑ 1460.6468 (1+)									
☑ 1472.6502 (1+)									
☑ 1487.6537 (1+)									
☑ 1492.7658 (1+)									
☑ 1497.6465 (1+)									
☑ 1514.6588 (1+)									
☑ 1675.7597 (1+)									
☑ 1789.8050 (1+)	●								

☑ 1804.7936 (1+)									
☑ 1830.8140 (1+)									
☑ 1843.8083 (1+)									
☑ 1846.8021 (1+)									
☑ 1854.8068 (1+)									
☑ 1856.8190 (1+)									
☑ 1859.8156 (1+)									
☑ 1863.8240 (1+)									
☑ 1871.8141 (1+)									
☑ 1873.8084 (1+)									
☑ 1874.8260 (1+)									
☑ 1877.8133 (1+)									
☑ 1886.8107 (1+)									
☑ 1889.8230 (1+)									
☑ 1901.8388 (1+)									
☑ 1903.8212 (1+)									
☑ 1904.8241 (1+)									
☑ 1913.8384 (1+)									
☑ 1916.8348 (1+)									
☑ 1918.8216 (1+)									
☑ 1920.8491 (1+)	●								
☑ 1930.8525 (1+)									
☑ 1932.8511 (1+)									
☑ 1934.8641 (1+)									
☑ 1936.8402 (1+)	●								
☑ 1943.8467 (1+)									
☑ 1946.8604 (1+)									
☑ 1950.8451 (1+)									
☑ 1959.8589 (1+)									
☑ 1962.8577 (1+)									
☑ 1978.8503 (1+)									
☑ 2058.8800 (1+)									
☑ 2072.9044 (1+)									
☑ 2081.9145 (1+)									
☑ 2084.9341 (1+)									
☑ 2098.9058 (1+)									
☑ 2114.9091 (1+)									
☑ 2117.9278 (1+)				●	●			●	
☑ 2127.9375 (1+)									
☑ 2130.9411 (1+)									
☑ 2133.8936 (1+)									
☑ 2135.8843 (1+)									
☑ 2139.9196 (1+)									
☑ 2142.9347 (1+)									
☑ 2145.9233 (1+)									
☑ 2153.9357 (1+)									
☑ 2156.9370 (1+)									
☑ 2159.9208 (1+)									
☑ 2160.2966 (1+)									
☑ 2172.9286 (1+)									
☑ 2174.9524 (1+)	●								
☑ 2186.9516 (1+)									
☑ 2188.9942 (1+)									
☑ 2190.9396 (1+)	●								
☑ 2200.9651 (1+)									
☑ 2205.9396 (1+)									
☑ 2208.9277 (1+)									
☑ 2216.9604 (1+)									
☑ 2231.9587 (1+)									
☑ 2318.9909 (1+)									
☑ 2346.0039 (1+)									
☑ 2361.9830 (1+)									
☑ 2872.4359 (1+)									

Select All | Select None | Search Selected | ☐ **Error tolerant** | Archive Report

1. DID5B_MACLO **Mass:** 8235 **Score:** 500 **Matches:** 8(8) **Sequences:** 6(6)
Disintegrin VLO5B OS=Macrovipera lebetina obtusa OX=209528 PE=1 SV=1
☐ Check to include this hit in error tolerant search or archive report

Query	Observed	Mr(expt)	Mr(calc)	ppm	Miss	Score	Expect	Rank	Unique	Peptide
3	1049.5703	1048.5630	1048.5376	24.3	0	53	1.9e-005	1		K.FLNPGTICK.R
11	1205.6684	1204.6612	1204.6387	18.7	1	30	0.0013	1		K.FLNPGTICKR.T
16	1318.5360	1317.5287	1317.5013	20.8	0	25	0.0031	1		R.GEHCVSGPCCR.N
35	1789.8050	1788.7977	1788.7706	15.2	0	54	4.2e-006	1	U	M.NSANPCCDPITCKPR.R
56	1920.8491	1919.8418	1919.8110	16.0	0	83	4.7e-009	1	U	-.MNSANPCCDPITCKPR.R
60	1936.8402	1935.8329	1935.8060	13.9	0	(58)	1.8e-006	1	U	-.MNSANPCCDPITCKPR.R + Oxidation (M)
86	2174.9524	2173.9451	2173.9079	17.1	0	155	3e-016	1	U	R.TMLDGLNDYCTGVTSDCPR.N
89	2190.9396	2189.9323	2189.9028	13.5	0	(138)	1.7e-014	1	U	R.TMLDGLNDYCTGVTSDCPR.N + Oxidation (M)

2. A0A1I9KNR4_VIPAA **Mass:** 14601 **Score:** 145 **Matches:** 4(4) **Sequences:** 4(4)
Disintegrin Dis-1 OS=Vipera ammodytes ammodytes OX=8705 PE=2 SV=1
☐ Check to include this hit in error tolerant search or archive report

Query	Observed	Mr(expt)	Mr(calc)	ppm	Miss	Score	Expect	Rank	Unique	Peptide

Query	Observed	Mr(expt)	Mr(calc)	ppm	Miss	Score	Expect	Rank	Unique	Peptide
3	1049.5703	1048.5630	1048.5376	24.3	0	53	1.9e-005	1		K.FLNPGTICK.R
11	1205.6684	1204.6612	1204.6387	18.7	1	30	0.0013	1		K.FLNPGTICKR.A
16	1318.5360	1317.5287	1317.5013	20.8	0	25	0.0031	1		K.GEHCVSGPCCR.N
**26**	**1446.6351**	**1445.6279**	**1445.5962**	**21.9**	**1**	**80**	**1e-008**	**1**	**U**	**R.KGEHCVSGPCCR.N**

Proteins matching the same set of peptides:
A0A1I9KNR7_VIPAA **Mass:** 14615 **Score:** 145 **Matches:** 4(4) **Sequences:** 4(4)
Disintegrin Dis-1 OS=Vipera ammodytes ammodytes OX=8705 PE=2 SV=1
DID2A_MACLB **Mass:** 14638 **Score:** 145 **Matches:** 4(4) **Sequences:** 4(4)
Disintegrin lebein-2-alpha OS=Macrovipera lebetina OX=8709 PE=1 SV=1

---

3. A0A223PK18_DABRR **Score:** 0 **Matches:** 1(0) **Sequences:** 1(0)
C-type lectin domain family 16 member A (Fragment) OS=Daboia russelii OX=8707 PE=2 SV=1
☐ Check to include this hit in error tolerant search or archive report

Query	Observed	Mr(expt)	Mr(calc)	ppm	Miss	Score	Expect	Rank	Unique	Peptide
11	1205.6684	1204.6612	1204.5619	82.4	1	9	0.18	2	U	R.RSSCDPTVQR.S

---

4. Q8UVG8_DABRR **Mass:** 52931 **Score:** 0 **Matches:** 1(0) **Sequences:** 1(0)
Vimentin OS=Daboia russelii OX=8707 PE=2 SV=1
☐ Check to include this hit in error tolerant search or archive report

Query	Observed	Mr(expt)	Mr(calc)	ppm	Miss	Score	Expect	Rank	Unique	Peptide
**73**	**2117.9278**	**2116.9206**	**2116.9623**	**-19.72**	**0**	**5**	**1.2**	**1**	**U**	**R.TGGDAMVDFSLADAINTEFK.A + Oxidation (M)**

---

5. A0A223PK34_DABRR **Score:** 0 **Matches:** 1(0) **Sequences:** 1(0)
Guanylate cyclase (Fragment) OS=Daboia russelii OX=8707 PE=2 SV=1
☐ Check to include this hit in error tolerant search or archive report

Query	Observed	Mr(expt)	Mr(calc)	ppm	Miss	Score	Expect	Rank	Unique	Peptide
73	2117.9278	2116.9206	2116.7267	91.6	1	3	1.8	2	U	K.GRXXXXXTGIVSMDQNNDR.D

---

6. A0A6G5ZUB2_9SAUR **Mass:** 81727 **Score:** 0 **Matches:** 1(0) **Sequences:** 1(0)
Disintegrin and metalloproteinase domain-containing protein 9 (Fragment) OS=Vipera anatolica senliki OX=2604287 PE=2 SV=1
☐ Check to include this hit in error tolerant search or archive report

Query	Observed	Mr(expt)	Mr(calc)	ppm	Miss	Score	Expect	Rank	Unique	Peptide
11	1205.6684	1204.6612	1204.5982	52.2	1	2	0.84	3	U	R.TSNCLRDALR.I

---

7. A0A6G5ZVV3_9SAUR **Score:** 0 **Matches:** 1(0) **Sequences:** 1(0)
Vascular endothelial growth factor 2 OS=Vipera anatolica senliki OX=2604287 PE=2 SV=1
☐ Check to include this hit in error tolerant search or archive report

Query	Observed	Mr(expt)	Mr(calc)	ppm	Miss	Score	Expect	Rank	Unique	Peptide
16	1318.5360	1317.5287	1317.6459	-88.95	1	2	0.61	2	U	K.QLELNERTCR.C

Proteins matching the same set of peptides:
VEGFA_VIPAA **Score:** 0 **Matches:** 1(0) **Sequences:** 1(0)

---

8. A0A161KGM7_VIPUR **Score:** 0 **Matches:** 1(0) **Sequences:** 1(0)
Prolactin receptor (Fragment) OS=Vipera ursinii macrops OX=927675 GN=PRLR PE=4 SV=1
☐ Check to include this hit in error tolerant search or archive report

Query	Observed	Mr(expt)	Mr(calc)	ppm	Miss	Score	Expect	Rank	Unique	Peptide
73	2117.9278	2116.9206	2117.0640	-67.77	1	1	3.2	3	U	K.EVGKLLPAAEVYDSDEVQR.N

---

9. B8QCD1_DABRR **Score:** 0 **Matches:** 1(0) **Sequences:** 1(0)
Endothelin converting enzyme-like protein 1 (Fragment) OS=Daboia russelii OX=8707 GN=ECEL1 PE=4 SV=1
☐ Check to include this hit in error tolerant search or archive report

Query	Observed	Mr(expt)	Mr(calc)	ppm	Miss	Score	Expect	Rank	Unique	Peptide
11	1205.6684	1204.6612	1204.6312	24.9	1	0	1.3	4	U	R.RPYQASAERK.V

Peptide matches not assigned to protein hits: (no details means no match)

	Query	Observed	Mr(expt)	Mr(calc)	ppm	Miss	Score	Expect	Rank	Unique	Peptide
☑	1	*992.5466*	*991.5394*								
☑	2	*1030.5622*	*1029.5549*								
☑	4	*1063.5850*	*1062.5777*								
☑	5	*1075.5835*	*1074.5763*								
☑	6	*1091.5833*	*1090.5760*								
☑	7	*1148.6528*	*1147.6455*								
☑	8	*1174.5232*	*1173.5159*								
☑	9	*1186.6320*	*1185.6247*								
☑	10	*1203.6590*	*1202.6517*								
☑	12	*1229.6714*	*1228.6641*								
☑	13	*1246.6979*	*1245.6906*								
☑	14	*1261.5132*	*1260.5059*								
☑	15	*1262.6952*	*1261.6879*								
☑	17	*1320.6106*	*1319.6033*								
☑	18	*1355.6158*	*1354.6086*								
☑	19	*1368.6087*	*1367.6014*								
☑	20	*1382.6064*	*1381.5992*								
☑	21	*1389.6115*	*1388.6043*								
☑	22	*1415.6205*	*1414.6133*								
☑	23	*1427.6237*	*1426.6164*								
☑	24	*1439.6265*	*1438.6192*								
☑	25	*1443.6212*	*1442.6140*								
☑	27	*1457.6356*	*1456.6284*								
☑	28	*1460.6468*	*1459.6395*								
☑	29	*1472.6502*	*1471.6429*								
☑	30	*1487.6537*	*1486.6465*								

☑	31	1492.7658	1491.7585
☑	32	1497.6465	1496.6393
☑	33	1514.6588	1513.6516
☑	34	1675.7597	1674.7524
☑	36	1804.7936	1803.7863
☑	37	1830.8140	1829.8068
☑	38	1843.8083	1842.8010
☑	39	1846.8021	1845.7948
☑	40	1854.8068	1853.7996
☑	41	1856.8190	1855.8118
☑	42	1859.8156	1858.8083
☑	43	1863.8240	1862.8167
☑	44	1871.8141	1870.8068
☑	45	1873.8084	1872.8011
☑	46	1874.8260	1873.8187
☑	47	1877.8133	1876.8060
☑	48	1886.8107	1885.8034
☑	49	1889.8230	1888.8157
☑	50	1901.8388	1900.8316
☑	51	1903.8212	1902.8140
☑	52	1904.8241	1903.8168
☑	53	1913.8384	1912.8312
☑	54	1916.8348	1915.8276
☑	55	1918.8216	1917.8144
☑	57	1930.8525	1929.8452
☑	58	1932.8511	1931.8439
☑	59	1934.8641	1933.8569
☑	61	1943.8467	1942.8394
☑	62	1946.8604	1945.8531
☑	63	1950.8451	1949.8378
☑	64	1959.8589	1958.8516
☑	65	1962.8577	1961.8504
☑	66	1978.8503	1977.8431
☑	67	2058.8800	2057.8727
☑	68	2072.9044	2071.8971
☑	69	2081.9145	2080.9072
☑	70	2084.9341	2083.9268
☑	71	2098.9058	2097.8985
☑	72	2114.9091	2113.9018
☑	74	2127.9375	2126.9302
☑	75	2130.9411	2129.9338
☑	76	2133.8936	2132.8863
☑	77	2135.8843	2134.8771
☑	78	2139.9196	2138.9124
☑	79	2142.9347	2141.9275
☑	80	2145.9233	2144.9160
☑	81	2153.9357	2152.9284
☑	82	2156.9370	2155.9297
☑	83	2159.9208	2158.9136
☑	84	2160.2966	2159.2893
☑	85	2172.9286	2171.9213
☑	87	2186.9516	2185.9443
☑	88	2188.9942	2187.9869
☑	90	2200.9651	2199.9579
☑	91	2205.9396	2204.9324
☑	92	2208.9277	2207.9204
☑	93	2216.9604	2215.9531
☑	94	2231.9587	2230.9514
☑	95	2318.9909	2317.9836
☑	96	2346.0039	2344.9966
☑	97	2361.9830	2360.9757
☑	98	2872.4359	2871.4286

Search Parameters

```
Type of search         : MS/MS Ion Search
Enzyme                 : Trypsin
Fixed modifications    : Carbamidomethyl (C)
Variable modifications : Oxidation (M)
Mass values            : Monoisotopic
Protein Mass           : Unrestricted
Peptide Mass Tolerance : ± 100 ppm
Fragment Mass Tolerance: ± 0.7 Da
Max Missed Cleavages   : 1
Instrument type        : MALDI-TOF-TOF
Number of queries      : 98
```

Mascot: http://www.matrixscience.com/

Mascot Search Results

```
User          :
Email         :
Search title  : vipera_all_Lift file:\\Proteomics1\d\data\2105\210505_tliepol_1_68252-68266\68252_68266_lpardo\0_G8\1\0_G8.xml
Database      : vipera  (1414 sequences; 347452 residues)
Timestamp     : 5 May 2021 at 09:48:41 GMT
Warning       : A Peptide summary report will usually give a much clearer picture of MS/MS search results.
Top Score     : 457 for DID5B_MACLO, Disintegrin VLO5B OS=Macrovipera lebetina obtusa OX=209528 PE=1 SV=1
```

Mascot Score Histogram

Protein score is -10*Log(P), where P is the probability that the observed match is a random event.
Protein scores greater than 44 are significant (p<0.05).
Protein scores are derived from ions scores as a non-probabilistic basis for ranking protein hits.

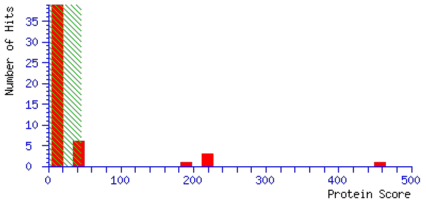

Protein Summary Report

Format As | Protein Summary (deprecated) | Help

Significance threshold p< 0.05 Max. number of hits 20

Overview Table

Click on column header to jump to entry in results list.
Move mouse over any indicator to highlight identical peptides.
Click on an indicator to see details of individual match.
Use check boxes to select sub-set of queries for new search.

Mouse over: -Query-
-Accession- -Sequence-

Hit:	1	2	3	4	5	6	7	8	9	10	11	12	13	14	15	16	17	18	19	20
☑ 992.5466 (1+)																				
☑ 1030.5622 (1+)																				
☑ 1049.5703 (1+)	●	●	●	●																
☑ 1063.5850 (1+)																				
☑ 1075.5835 (1+)																				
☑ 1091.5833 (1+)																				
☑ 1148.6528 (1+)																				
☑ 1174.5232 (1+)																				
☑ 1186.6320 (1+)																				
☑ 1203.6590 (1+)																				
☑ 1205.6684 (1+)	●	●	●	●																
☑ 1229.6714 (1+)																				
☑ 1246.6979 (1+)																				
☑ 1261.5132 (1+)																				
☑ 1262.6952 (1+)																				
☑ 1318.5360 (1+)	●	●	●	●	●	●	●	●	●	●	●									
☑ 1320.6106 (1+)																				
☑ 1355.6158 (1+)																				
☑ 1368.6087 (1+)																				
☑ 1382.6064 (1+)																				
☑ 1389.6115 (1+)																				
☑ 1415.6205 (1+)																				
☑ 1427.6237 (1+)																				
☑ 1439.6265 (1+)																				
☑ 1443.6212 (1+)																				
☑ 1446.6351 (1+)		●	●	●																
☑ 1457.6356 (1+)																				
☑ 1460.6468 (1+)																				
☑ 1472.6502 (1+)																				
☑ 1487.6537 (1+)																				
☑ 1492.7658 (1+)																				
☑ 1497.6465 (1+)																				
☑ 1514.6588 (1+)																				
☑ 1675.7597 (1+)																				
☑ 1789.8050 (1+)	●				●															
☑ 1804.7936 (1+)																				
☑ 1830.8140 (1+)																				
☑ 1843.8083 (1+)																				
☑ 1846.8021 (1+)																				
☑ 1854.8068 (1+)																				
☑ 1856.8190 (1+)																				
☑ 1859.8156 (1+)																				
☑ 1863.8240 (1+)																				
☑ 1871.8141 (1+)																				
☑ 1873.8084 (1+)																				
☑ 1874.8260 (1+)																				
☑ 1877.8133 (1+)																				
☑ 1886.8107 (1+)																				
☑ 1889.8230 (1+)																				

☑ 1901.8388 (1+)																				
☑ 1903.8212 (1+)																				
☑ 1904.8241 (1+)																				
☑ 1913.8384 (1+)																				
☑ 1916.8348 (1+)																				
☑ 1918.8216 (1+)																				
☑ 1920.8491 (1+)	●				●															
☑ 1930.8525 (1+)																				
☑ 1932.8511 (1+)																				
☑ 1934.8641 (1+)																				
☑ 1936.8402 (1+)	●				●															
☑ 1943.8467 (1+)																				
☑ 1946.8604 (1+)																				
☑ 1950.8451 (1+)																				
☑ 1959.8589 (1+)																				
☑ 1962.8577 (1+)																				
☑ 1978.8503 (1+)																				
☑ 2058.8800 (1+)																				
☑ 2072.9044 (1+)																				
☑ 2081.9145 (1+)																				
☑ 2084.9341 (1+)																				
☑ 2098.9058 (1+)																				
☑ 2114.9091 (1+)																				
☑ 2117.9278 (1+)														●						
☑ 2127.9375 (1+)																				
☑ 2130.9411 (1+)																				
☑ 2133.8936 (1+)																				
☑ 2135.8843 (1+)																				
☑ 2139.9196 (1+)																				
☑ 2142.9347 (1+)																				
☑ 2145.9233 (1+)																				
☑ 2153.9357 (1+)																				
☑ 2156.9370 (1+)																				
☑ 2159.9208 (1+)																				
☑ 2160.2966 (1+)																				
☑ 2172.9286 (1+)																				
☑ 2174.9524 (1+)	●																			
☑ 2186.9516 (1+)																				
☑ 2188.9942 (1+)																				
☑ 2190.9396 (1+)	●																			
☑ 2200.9651 (1+)																				
☑ 2205.9396 (1+)																				
☑ 2208.9277 (1+)																				
☑ 2216.9604 (1+)																				
☑ 2231.9587 (1+)																				
☑ 2318.9909 (1+)																				
☑ 2346.0039 (1+)																				
☑ 2361.9830 (1+)																				
☑ 2872.4359 (1+)																				

Select All   Select None   Search Selected

Index

	Accession	Mass	Score	Description
1.	DID5B_MACLO	8235	457	Disintegrin VLO5B OS=Macrovipera lebetina obtusa OX=209528 PE=1 SV=1
2.	DID2A_MACLB	14638	222	Disintegrin lebein-2-alpha OS=Macrovipera lebetina OX=8709 PE=1 SV=1
3.	A0A1I9KNR7_VIPAA	14615	212	Disintegrin Dis-1 OS=Vipera ammodytes ammodytes OX=8705 PE=2 SV=1
4.	A0A1I9KNR4_VIPAA	14601	212	Disintegrin Dis-1 OS=Vipera ammodytes ammodytes OX=8705 PE=2 SV=1
5.	Q1JRG9_MACLN	7698	195	VGD-containing dimeric disintegrin subunit ML-G1 (Fragment) OS=Macrovipera lebetina transmediterranea OX=384075 GN=ml-G1 PE=4 SV=1
6.	DID1A_MACLB	12718	44	Disintegrin lebein-1-alpha OS=Macrovipera lebetina OX=8709 PE=1 SV=2
7.	VM2A6_VIPAA	7588	40	Disintegrin VA6 OS=Vipera ammodytes ammodytes OX=8705 PE=1 SV=1
8.	DID7A_VIPBB	7574	40	Disintegrin VB7A OS=Vipera berus berus OX=31156 PE=1 SV=1
9.	A0A6G5ZVR7_9SAUR	9593	38	Disintegrin (Fragment) OS=Vipera anatolica senliki OX=2604287 PE=2 SV=1
10.	A0A1J0CZN0_VIPAA	12765	36	Dis-2 OS=Vipera ammodytes ammodytes OX=8705 PE=2 SV=1
11.	DID4_MACLO	7673	31	Disintegrin VLO4 OS=Macrovipera lebetina obtusa OX=209528 PE=1 SV=1
12.	M4T472_9PERO	26404	26	Ring finger protein 213 (Fragment) OS=Echiichthys vipera OX=94984 PE=4 SV=1
13.	VM3LC_MACLN	24215	25	Disintegrin-like leberagin-C OS=Macrovipera lebetina transmediterranea OX=384075 PE=1 SV=1
14.	Q8UVG8_DABRR	52931	24	Vimentin OS=Daboia russelii OX=8707 PE=2 SV=1
15.	F2Q6F2_DABRR	27583	24	Cysteine-rich secretory protein Dr-CRPK OS=Daboia russelii OX=8707 PE=2 SV=1
16.	B8QQ51_9PERO	33786	22	RNF213 (Fragment) OS=Echiichthys vipera OX=94984 GN=RNF213 PE=4 SV=1
17.	A0A6G5ZVZ2_9SAUR	53939	19	Lactadherin 1 OS=Vipera anatolica senliki OX=2604287 PE=2 SV=1
18.	A0A6G5ZW39_9SAUR	54634	19	Lactadherin 3 OS=Vipera anatolica senliki OX=2604287 PE=2 SV=1
19.	PA2A8_DABPA	16194	18	Acidic phospholipase A2 VP8 OS=Daboia palaestinae OX=1170828 PE=3 SV=1
20.	A0A286S1C5_9SAUR	39024	18	E3 ubiquitin-protein ligase RAG1 (Fragment) OS=Vipera seoanei OX=110211 GN=RAG1 PE=4 SV=1

Results List

1. DID5B_MACLO **Mass:** 8235 **Score:** 457 **Expect:** 2.8e-043 **Matches:** 8

Disintegrin VLO5B OS=Macrovipera lebetina obtusa OX=209528 PE=1 SV=1

Observed	Mr(expt)	Mr(calc)	ppm	Start	End	Miss	Ions	Peptide
1049.5703	1048.5630	1048.5376	24.3	32 -	40	0	53	K.FLNPGTICK.R
1205.6684	1204.6612	1204.6387	18.7	32 -	41	1	30	K.FLNPGTICKR.T
1318.5360	1317.5287	1317.5013	20.8	18 -	28	0	25	R.GEHCVSGPCCR.N
1789.8050	1788.7977	1788.7706	15.2	2 -	16	0	54	M.NSANPCCDPITCKPR.R
1920.8491	1919.8418	1919.8110	16.0	1 -	16	0	83	-.MNSANPCCDPITCKPR.R
1936.8402	1935.8329	1935.8060	13.9	1 -	16	0	(58)	-.MNSANPCCDPITCKPR.R + Oxidation (M)
2174.9524	2173.9451	2173.9079	17.1	42 -	60	0	155	R.TMLDGLNDYCTGVTSDCPR.N
2190.9396	2189.9323	2189.9028	13.5	42 -	60	0	(138)	R.TMLDGLNDYCTGVTSDCPR.N + Oxidation (M)

No match to: 992.5466, 1030.5622, 1063.5850, 1075.5835, 1091.5833, 1148.6528, 1174.5232, 1186.6320, 1203.6590, 1229.6714, 1246.6979, 1261.5132, 1262.6952, 1320.6106, 1355.6158, 1368.6087, 1382.6064, 1389.6115, 1415.6205, 1427.6237, 1439.6265, 1443.6212, 1446.6351, 1457.6356, 1460.6468, 1472.6502, 1487.6537, 1492.7658, 1497.6465, 1514.6588, 1675.7597, 1804.7936, 1830.8140, 1843.8083, 1846.8021, 1854.8068, 1856.8190, 1859.8156, 1863.8240, 1871.8141, 1873.8084, 1874.8260, 1877.8133, 1886.8107, 1889.8230, 1901.8388, 1903.8212, 1904.8241, 1913.8384, 1916.8348, 1918.8216, 1930.8525, 1932.8511, 1934.8641, 1943.8467, 1946.8604, 1950.8451, 1959.8589, 1962.8577, 1978.8503, 2058.8800, 2072.9044, 2081.9145, 2084.9341, 2098.9058, 2114.9091, 2117.9278, 2127.9375, 2130.9411, 2133.8936, 2135.8843, 2139.9196, 2142.9347, 2145.9233, 2153.9357, 2156.9370, 2159.9208, 2160.2966, 2172.9286, 2186.9516, 2188.9942, 2200.9651, 2205.9396, 2208.9277, 2216.9604, 2231.9587, 2318.9909, 2346.0039, 2361.9830, 2872.4359

2. DID2A_MACLB **Mass:** 14638 **Score:** 222 **Expect:** 8.9e-020 **Matches:** 5

Disintegrin lebein-2-alpha OS=Macrovipera lebetina OX=8709 PE=1 SV=1

Observed	Mr(expt)	Mr(calc)	ppm	Start	End	Miss	Ions	Peptide

1049.5703	1048.5630	1048.5376	24.3	78 -	86	0	53	K.FLNPGTICK.R
1205.6684	1204.6612	1204.6387	18.7	78 -	87	1	30	K.FLNPGTICKR.T
1318.5360	1317.5287	1317.5013	20.8	64 -	74	0	25	K.GEHCVSGPCCR.N
1446.6351	1445.6279	1445.5962	21.9	63 -	74	1	80	R.KGEHCVSGPCCR.N
2188.9942	2187.9869	2187.9235	29.0	88 -	106	0	---	R.TMLDGLNDYCTGITSDCPR.N

No match to: 992.5466, 1030.5622, 1063.5850, 1075.5835, 1091.5833, 1148.6528, 1174.5232, 1186.6320, 1203.6590, 1229.6714, 1246.6979, 1261.5132, 1262.6952, 1320.6106, 1355.6158, 1368.6087, 1382.6064, 1389.6115, 1415.6205, 1427.6237, 1439.6265, 1443.6212, 1457.6356, 1460.6468, 1472.6502, 1487.6537, 1492.7658, 1497.6465, 1514.6588, 1675.7597, 1789.8050, 1804.7936, 1830.8140, 1843.8083, 1846.8021, 1854.8068, 1856.8190, 1859.8156, 1863.8240, 1871.8141, 1873.8084, 1874.8260, 1877.8133, 1886.8107, 1889.8230, 1901.8388, 1903.8212, 1904.8241, 1913.8384, 1916.8348, 1918.8216, 1920.8491, 1930.8525, 1932.8511, 1934.8641, 1936.8402, 1943.8467, 1946.8604, 1950.8451, 1959.8589, 1962.8577, 1978.8503, 2058.8800, 2072.9044, 2081.9145, 2084.9341, 2098.9058, 2114.9091, 2117.9278, 2127.9375, 2130.9411, 2133.8936, 2135.8843, 2139.9196, 2142.9347, 2145.9233, 2153.9357, 2156.9370, 2159.9208, 2160.2966, 2172.9286, 2174.9524, 2186.9516, 2190.9396, 2200.9651, 2205.9396, 2208.9277, 2216.9604, 2231.9587, 2318.9909, 2346.0039, 2361.9830, 2872.4359

3. A0A1I9KNR7_VIPAA **Mass:** 14615 **Score:** 212 **Expect:** 8.9e-019 **Matches:** 4

Disintegrin Dis-1 OS=Vipera ammodytes ammodytes OX=8705 PE=2 SV=1

Observed	Mr(expt)	Mr(calc)	ppm	Start	End	Miss	Ions	Peptide
1049.5703	1048.5630	1048.5376	24.3	78 -	86	0	53	K.FLNPGTICK.R
1205.6684	1204.6612	1204.6387	18.7	78 -	87	1	30	K.FLNPGTICKR.A
1318.5360	1317.5287	1317.5013	20.8	64 -	74	0	25	K.GEHCVSGPCCR.N
1446.6351	1445.6279	1445.5962	21.9	63 -	74	1	80	R.KGEHCVSGPCCR.N

No match to: 992.5466, 1030.5622, 1063.5850, 1075.5835, 1091.5833, 1148.6528, 1174.5232, 1186.6320, 1203.6590, 1229.6714, 1246.6979, 1261.5132, 1262.6952, 1320.6106, 1355.6158, 1368.6087, 1382.6064, 1389.6115, 1415.6205, 1427.6237, 1439.6265, 1443.6212, 1457.6356, 1460.6468, 1472.6502, 1487.6537, 1492.7658, 1497.6465, 1514.6588, 1675.7597, 1789.8050, 1804.7936, 1830.8140, 1843.8083, 1846.8021, 1854.8068, 1856.8190, 1859.8156, 1863.8240, 1871.8141, 1873.8084, 1874.8260, 1877.8133, 1886.8107, 1889.8230, 1901.8388, 1903.8212, 1904.8241, 1913.8384, 1916.8348, 1918.8216, 1920.8491, 1930.8525, 1932.8511, 1934.8641, 1936.8402, 1943.8467, 1946.8604, 1950.8451, 1959.8589, 1962.8577, 1978.8503, 2058.8800, 2072.9044, 2081.9145, 2084.9341, 2098.9058, 2114.9091, 2117.9278, 2127.9375, 2130.9411, 2133.8936, 2135.8843, 2139.9196, 2142.9347, 2145.9233, 2153.9357, 2156.9370, 2159.9208, 2160.2966, 2172.9286, 2174.9524, 2186.9516, 2188.9942, 2190.9396, 2200.9651, 2205.9396, 2208.9277, 2216.9604, 2231.9587, 2318.9909, 2346.0039, 2361.9830, 2872.4359

4. A0A1I9KNR4_VIPAA **Mass:** 14601 **Score:** 212 **Expect:** 8.9e-019 **Matches:** 4

Disintegrin Dis-1 OS=Vipera ammodytes ammodytes OX=8705 PE=2 SV=1

Observed	Mr(expt)	Mr(calc)	ppm	Start	End	Miss	Ions	Peptide
1049.5703	1048.5630	1048.5376	24.3	78 -	86	0	53	K.FLNPGTICK.R
1205.6684	1204.6612	1204.6387	18.7	78 -	87	1	30	K.FLNPGTICKR.A
1318.5360	1317.5287	1317.5013	20.8	64 -	74	0	25	K.GEHCVSGPCCR.N
1446.6351	1445.6279	1445.5962	21.9	63 -	74	1	80	R.KGEHCVSGPCCR.N

No match to: 992.5466, 1030.5622, 1063.5850, 1075.5835, 1091.5833, 1148.6528, 1174.5232, 1186.6320, 1203.6590, 1229.6714, 1246.6979, 1261.5132, 1262.6952, 1320.6106, 1355.6158, 1368.6087, 1382.6064, 1389.6115, 1415.6205, 1427.6237, 1439.6265, 1443.6212, 1457.6356, 1460.6468, 1472.6502, 1487.6537, 1492.7658, 1497.6465, 1514.6588, 1675.7597, 1789.8050, 1804.7936, 1830.8140, 1843.8083, 1846.8021, 1854.8068, 1856.8190, 1859.8156, 1863.8240, 1871.8141, 1873.8084, 1874.8260, 1877.8133, 1886.8107, 1889.8230, 1901.8388, 1903.8212, 1904.8241, 1913.8384, 1916.8348, 1918.8216, 1920.8491, 1930.8525, 1932.8511, 1934.8641, 1936.8402, 1943.8467, 1946.8604, 1950.8451, 1959.8589, 1962.8577, 1978.8503, 2058.8800, 2072.9044, 2081.9145, 2084.9341, 2098.9058, 2114.9091, 2117.9278, 2127.9375, 2130.9411, 2133.8936, 2135.8843, 2139.9196, 2142.9347, 2145.9233, 2153.9357, 2156.9370, 2159.9208, 2160.2966, 2172.9286, 2174.9524, 2186.9516, 2188.9942, 2190.9396, 2200.9651, 2205.9396, 2208.9277, 2216.9604, 2231.9587, 2318.9909, 2346.0039, 2361.9830, 2872.4359

5. Q1JRG9_MACLN **Mass:** 7698 **Score:** 195 **Expect:** 4.5e-017 **Matches:** 5

VGD-containing dimeric disintegrin subunit ML-G1 (Fragment) OS=Macrovipera lebetina transmediterranea OX=384075 GN=ml-G1 PE=4 SV=1

Observed	Mr(expt)	Mr(calc)	ppm	Start	End	Miss	Ions	Peptide
1318.5360	1317.5287	1317.5013	20.8	18 -	28	0	25	R.GEHCVSGPCCR.N
1789.8050	1788.7977	1788.7706	15.2	2 -	16	0	54	M.NSANPCCDPITCKPR.R
1920.8491	1919.8418	1919.8110	16.0	1 -	16	0	83	-.MNSANPCCDPITCKPR.R
1936.8402	1935.8329	1935.8060	13.9	1 -	16	0	(58)	-.MNSANPCCDPITCKPR.R + Oxidation (M)
2133.8936	2132.8863	2132.8085	36.5	42 -	60	0	---	R.AVGDDMDDYCTGISSDCPR.N

No match to: 992.5466, 1030.5622, 1049.5703, 1063.5850, 1075.5835, 1091.5833, 1148.6528, 1174.5232, 1186.6320, 1203.6590, 1205.6684, 1229.6714, 1246.6979, 1261.5132, 1262.6952, 1320.6106, 1355.6158, 1368.6087, 1382.6064, 1389.6115, 1415.6205, 1427.6237, 1439.6265, 1443.6212, 1446.6351, 1457.6356, 1460.6468, 1472.6502, 1487.6537, 1492.7658, 1497.6465, 1514.6588, 1675.7597, 1804.7936, 1830.8140, 1843.8083, 1846.8021, 1854.8068, 1856.8190, 1859.8156, 1863.8240, 1871.8141, 1873.8084, 1874.8260, 1877.8133, 1886.8107, 1889.8230, 1901.8388, 1903.8212, 1904.8241, 1913.8384, 1916.8348, 1918.8216, 1930.8525, 1932.8511, 1934.8641, 1943.8467, 1946.8604, 1950.8451, 1959.8589, 1962.8577, 1978.8503, 2058.8800, 2072.9044, 2081.9145, 2084.9341, 2098.9058, 2114.9091, 2117.9278, 2127.9375, 2130.9411, 2135.8843, 2139.9196, 2142.9347, 2145.9233, 2153.9357, 2156.9370, 2159.9208, 2160.2966, 2172.9286, 2174.9524, 2186.9516, 2188.9942, 2190.9396, 2200.9651, 2205.9396, 2208.9277, 2216.9604, 2231.9587, 2318.9909, 2346.0039, 2361.9830, 2872.4359

6. DID1A_MACLB **Mass:** 12718 **Score:** 44 **Expect:** 0.051 **Matches:** 4

Disintegrin lebein-1-alpha OS=Macrovipera lebetina OX=8709 PE=1 SV=2

Observed	Mr(expt)	Mr(calc)	ppm	Start	End	Miss	Ions	Peptide
1318.5360	1317.5287	1317.5013	20.8	64 -	74	0	25	R.GEHCVSGPCCR.N
1962.8577	1961.8504	1961.7190	67.0	90 -	106	0	---	R.GDDMNDYCTGISSDCPR.N
1978.8503	1977.8431	1977.7139	65.3	90 -	106	0	---	R.GDDMNDYCTGISSDCPR.N + Oxidation (M)
2205.9396	2204.9324	2204.8521	36.4	88 -	106	1	---	R.ARGDDMNDYCTGISSDCPR.N + Oxidation (M)

No match to: 992.5466, 1030.5622, 1049.5703, 1063.5850, 1075.5835, 1091.5833, 1148.6528, 1174.5232, 1186.6320, 1203.6590, 1205.6684, 1229.6714, 1246.6979, 1261.5132, 1262.6952, 1320.6106, 1355.6158, 1368.6087, 1382.6064, 1389.6115, 1415.6205, 1427.6237, 1439.6265, 1443.6212, 1446.6351, 1457.6356, 1460.6468, 1472.6502, 1487.6537, 1492.7658, 1497.6465, 1514.6588, 1675.7597, 1789.8050, 1804.7936, 1830.8140, 1843.8083, 1846.8021, 1854.8068, 1856.8190, 1859.8156, 1863.8240, 1871.8141, 1873.8084, 1874.8260, 1877.8133, 1886.8107, 1889.8230, 1901.8388, 1903.8212, 1904.8241, 1913.8384, 1916.8348, 1918.8216, 1920.8491, 1930.8525, 1932.8511, 1934.8641, 1936.8402, 1943.8467, 1946.8604, 1950.8451, 1959.8589, 2058.8800, 2072.9044, 2081.9145, 2084.9341, 2098.9058, 2114.9091, 2117.9278, 2127.9375, 2130.9411, 2133.8936, 2135.8843, 2139.9196, 2142.9347, 2145.9233, 2153.9357, 2156.9370, 2159.9208, 2160.2966, 2172.9286, 2174.9524, 2186.9516, 2188.9942, 2190.9396, 2200.9651, 2208.9277, 2216.9604, 2231.9587, 2318.9909, 2346.0039, 2361.9830, 2872.4359

7. VM2A6_VIPAA **Mass:** 7588 **Score:** 40 **Expect:** 0.16 **Matches:** 3

Disintegrin VA6 OS=Vipera ammodytes ammodytes OX=8705 PE=1 SV=1

Observed	Mr(expt)	Mr(calc)	ppm	Start	End	Miss	Ions	Peptide
1318.5360	1317.5287	1317.5013	20.8	17 -	27	0	25	R.GEHCVSGPCCR.N
1962.8577	1961.8504	1961.7190	67.0	43 -	59	0	---	R.GDDMNDYCTGVTSDCPR.N
1978.8503	1977.8431	1977.7139	65.3	43 -	59	0	---	R.GDDMNDYCTGVTSDCPR.N + Oxidation (M)

No match to: 992.5466, 1030.5622, 1049.5703, 1063.5850, 1075.5835, 1091.5833, 1148.6528, 1174.5232, 1186.6320, 1203.6590, 1205.6684, 1229.6714, 1246.6979, 1261.5132, 1262.6952, 1320.6106, 1355.6158, 1368.6087, 1382.6064, 1389.6115, 1415.6205, 1427.6237, 1439.6265, 1443.6212, 1446.6351, 1457.6356, 1460.6468, 1472.6502, 1487.6537, 1492.7658, 1497.6465, 1514.6588, 1675.7597, 1789.8050, 1804.7936, 1830.8140, 1843.8083, 1846.8021, 1854.8068, 1856.8190, 1859.8156, 1863.8240, 1871.8141, 1873.8084, 1874.8260, 1877.8133, 1886.8107, 1889.8230, 1901.8388, 1903.8212, 1904.8241, 1913.8384, 1916.8348, 1918.8216, 1920.8491, 1930.8525, 1932.8511, 1934.8641, 1936.8402, 1943.8467, 1946.8604, 1950.8451, 1959.8589, 2058.8800, 2072.9044, 2081.9145, 2084.9341, 2098.9058, 2114.9091, 2117.9278, 2127.9375, 2130.9411, 2133.8936, 2135.8843, 2139.9196, 2142.9347, 2145.9233, 2153.9357, 2156.9370, 2159.9208, 2160.2966, 2172.9286, 2174.9524, 2186.9516, 2188.9942, 2190.9396, 2200.9651, 2205.9396, 2208.9277, 2216.9604, 2231.9587, 2318.9909, 2346.0039, 2361.9830, 2872.4359

8. DID7A_VIPBB **Mass:** 7574 **Score:** 40 **Expect:** 0.16 **Matches:** 3

Disintegrin VB7A OS=Vipera berus berus OX=31156 PE=1 SV=1

Observed	Mr(expt)	Mr(calc)	ppm	Start	End	Miss	Ions	Peptide
1318.5360	1317.5287	1317.5013	20.8	17 -	27	0	25	R.GEHCVSGPCCR.N
1962.8577	1961.8504	1961.7190	67.0	43 -	59	0	---	R.GDDMNDYCTGISSDCPR.N
1978.8503	1977.8431	1977.7139	65.3	43 -	59	0	---	R.GDDMNDYCTGISSDCPR.N + Oxidation (M)

No match to: 992.5466, 1030.5622, 1049.5703, 1063.5850, 1075.5835, 1091.5833, 1148.6528, 1174.5232, 1186.6320, 1203.6590, 1205.6684, 1229.6714, 1246.6979, 1261.5132, 1262.6952, 1320.6106, 1355.6158, 1368.6087, 1382.6064, 1389.6115, 1415.6205, 1427.6237, 1439.6265, 1443.6212, 1446.6351, 1457.6356, 1460.6468, 1472.6502, 1487.6537, 1492.7658, 1497.6465, 1514.6588, 1675.7597, 1789.8050, 1804.7936, 1830.8140, 1843.8083, 1846.8021, 1854.8068, 1856.8190, 1859.8156, 1863.8240, 1871.8141, 1873.8084, 1874.8260, 1877.8133, 1886.8107, 1889.8230, 1901.8388, 1903.8212, 1904.8241, 1913.8384, 1916.8348, 1918.8216, 1920.8491, 1930.8525, 1932.8511, 1934.8641, 1936.8402, 1943.8467, 1946.8604, 1950.8451, 1959.8589, 2058.8800, 2072.9044, 2081.9145, 2084.9341, 2098.9058, 2114.9091, 2117.9278, 2127.9375, 2130.9411, 2133.8936, 2135.8843, 2139.9196, 2142.9347, 2145.9233, 2153.9357, 2156.9370, 2159.9208, 2160.2966, 2172.9286, 2174.9524, 2186.9516, 2188.9942, 2190.9396, 2200.9651, 2205.9396, 2208.9277, 2216.9604, 2231.9587, 2318.9909, 2346.0039, 2361.9830, 2872.4359

9. A0A6G5ZVR7_9SAUR **Mass:** 9593 **Score:** 38 **Expect:** 0.23 **Matches:** 3

Disintegrin (Fragment) OS=Vipera anatolica senliki OX=2604287 PE=2 SV=1

Observed	Mr(expt)	Mr(calc)	ppm	Start	End	Miss	Ions	Peptide
1318.5360	1317.5287	1317.5013	20.8	35 -	45	0	25	R.GEHCVSGPCCR.N
1962.8577	1961.8504	1961.7190	67.0	61 -	77	0	---	R.GDDMNDYCTGISSDCPR.N
1978.8503	1977.8431	1977.7139	65.3	61 -	77	0	---	R.GDDMNDYCTGISSDCPR.N + Oxidation (M)

No match to: 992.5466, 1030.5622, 1049.5703, 1063.5850, 1075.5835, 1091.5833, 1148.6528, 1174.5232, 1186.6320, 1203.6590, 1205.6684, 1229.6714, 1246.6979, 1261.5132, 1262.6952, 1320.6106, 1355.6158, 1368.6087, 1382.6064, 1389.6115, 1415.6205, 1427.6237, 1439.6265, 1443.6212, 1446.6351, 1457.6356, 1460.6468, 1472.6502, 1487.6537, 1492.7658, 1497.6465, 1514.6588, 1675.7597, 1789.8050, 1804.7936, 1830.8140, 1843.8083, 1846.8021, 1854.8068, 1856.8190, 1859.8156, 1863.8240, 1871.8141, 1873.8084, 1874.8260, 1877.8133, 1886.8107, 1889.8230, 1901.8388, 1903.8212, 1904.8241, 1913.8384, 1916.8348, 1918.8216, 1920.8491, 1930.8525, 1932.8511, 1934.8641, 1936.8402, 1943.8467, 1946.8604, 1950.8451, 1959.8589, 2058.8800, 2072.9044, 2081.9145, 2084.9341, 2098.9058, 2114.9091, 2117.9278, 2127.9375, 2130.9411, 2133.8936, 2135.8843, 2139.9196, 2142.9347, 2145.9233, 2153.9357, 2156.9370, 2159.9208, 2160.2966, 2172.9286, 2174.9524, 2186.9516, 2188.9942, 2190.9396, 2200.9651, 2205.9396, 2208.9277, 2216.9604, 2231.9587, 2318.9909, 2346.0039, 2361.9830, 2872.4359

10. A0A1J0CZN0_VIPAA **Mass:** 12765 **Score:** 36 **Expect:** 0.36 **Matches:** 3

Dis-2 OS=Vipera ammodytes ammodytes OX=8705 PE=2 SV=1

Observed	Mr(expt)	Mr(calc)	ppm	Start	End	Miss	Ions	Peptide
1318.5360	1317.5287	1317.5013	20.8	64	74	0	25	R.GEHCVSGPCCR.N
1962.8577	1961.8504	1961.7190	67.0	90	106	0	---	R.GDDMNDYCTGISSDCPR.N
1978.8503	1977.8431	1977.7139	65.3	90	106	0	---	R.GDDMNDYCTGISSDCPR.N + Oxidation (M)

No match to: 992.5466, 1030.5622, 1049.5703, 1063.5850, 1075.5835, 1091.5833, 1148.6528, 1174.5232, 1186.6320, 1203.6590, 1205.6684, 1229.6714, 1246.6979, 1261.5132, 1262.6952, 1320.6106, 1355.6158, 1368.6087, 1382.6064, 1389.6115, 1415.6205, 1427.6237, 1439.6265, 1443.6212, 1446.6351, 1457.6356, 1460.6468, 1472.6502, 1487.6537, 1492.7658, 1497.6465, 1514.6588, 1675.7597, 1789.8050, 1804.7936, 1830.8140, 1843.8083, 1846.8021, 1854.8068, 1856.8190, 1859.8156, 1863.8240, 1871.8141, 1873.8084, 1874.8260, 1877.8133, 1886.8107, 1889.8230, 1901.8388, 1903.8212, 1904.8241, 1913.8384, 1916.8348, 1918.8216, 1920.8491, 1930.8525, 1932.8511, 1934.8641, 1936.8402, 1943.8467, 1946.8604, 1950.8451, 1959.8589, 2058.8800, 2072.9044, 2081.9145, 2084.9341, 2098.9058, 2114.9091, 2117.9278, 2127.9375, 2130.9411, 2133.8936, 2135.8843, 2139.9196, 2142.9347, 2145.9233, 2153.9357, 2156.9370, 2159.9208, 2160.2966, 2172.9286, 2174.9524, 2186.9516, 2188.9942, 2190.9396, 2200.9651, 2205.9396, 2208.9277, 2216.9604, 2231.9587, 2318.9909, 2346.0039, 2361.9830, 2872.4359

11. DID4_MACLO **Mass:** 7673 **Score:** 31 **Expect:** 1.2 **Matches:** 1

Disintegrin VLO4 OS=Macrovipera lebetina obtusa OX=209528 PE=1 SV=1

Observed	Mr(expt)	Mr(calc)	ppm	Start	End	Miss	Ions	Peptide
1318.5360	1317.5287	1317.5013	20.8	18	28	0	25	R.GEHCVSGPCCR.N

No match to: 992.5466, 1030.5622, 1049.5703, 1063.5850, 1075.5835, 1091.5833, 1148.6528, 1174.5232, 1186.6320, 1203.6590, 1205.6684, 1229.6714, 1246.6979, 1261.5132, 1262.6952, 1320.6106, 1355.6158, 1368.6087, 1382.6064, 1389.6115, 1415.6205, 1427.6237, 1439.6265, 1443.6212, 1446.6351, 1457.6356, 1460.6468, 1472.6502, 1487.6537, 1492.7658, 1497.6465, 1514.6588, 1675.7597, 1789.8050, 1804.7936, 1830.8140, 1843.8083, 1846.8021, 1854.8068, 1856.8190, 1859.8156, 1863.8240, 1871.8141, 1873.8084, 1874.8260, 1877.8133, 1886.8107, 1889.8230, 1901.8388, 1903.8212, 1904.8241, 1913.8384, 1916.8348, 1918.8216, 1920.8491, 1930.8525, 1932.8511, 1934.8641, 1936.8402, 1943.8467, 1946.8604, 1950.8451, 1959.8589, 1962.8577, 1978.8503, 2058.8800, 2072.9044, 2081.9145, 2084.9341, 2098.9058, 2114.9091, 2117.9278, 2127.9375, 2130.9411, 2133.8936, 2135.8843, 2139.9196, 2142.9347, 2145.9233, 2153.9357, 2156.9370, 2159.9208, 2160.2966, 2172.9286, 2174.9524, 2186.9516, 2188.9942, 2190.9396, 2200.9651, 2205.9396, 2208.9277, 2216.9604, 2231.9587, 2318.9909, 2346.0039, 2361.9830, 2872.4359

12. M4T472_9PERO **Mass:** 26404 **Score:** 26 **Expect:** 3.7 **Matches:** 6

Ring finger protein 213 (Fragment) OS=Echiichthys vipera OX=94984 PE=4 SV=1

Observed	Mr(expt)	Mr(calc)	ppm	Start	End	Miss	Ions	Peptide
1091.5833	1090.5760	1090.5692	6.24	191	199	1	---	K.EVLCDKTVK.G
1174.5232	1173.5159	1173.6263	-94.03	94	102	1	---	K.MLAIHMRFR.C
1262.6952	1261.6879	1261.6700	14.2	130	141	1	---	R.SGVASENMKLVK.V
1389.6115	1388.6043	1388.7089	-75.32	50	61	0	---	R.VPFNIDFDGLPR.G
1439.6265	1438.6192	1438.6762	-39.62	142	154	0	---	K.VHGGTTSEMIYTK.V + Oxidation (M)
2361.9830	2360.9757	2361.1569	-76.74	200	220	1	---	K.GESLTPNCGLQIIAACNPYRK.H

No match to: 992.5466, 1030.5622, 1049.5703, 1063.5850, 1075.5835, 1148.6528, 1186.6320, 1203.6590, 1205.6684, 1229.6714, 1246.6979, 1261.5132, 1318.5360, 1320.6106, 1355.6158, 1368.6087, 1382.6064, 1415.6205, 1427.6237, 1443.6212, 1446.6351, 1457.6356, 1460.6468, 1472.6502, 1487.6537, 1492.7658, 1497.6465, 1514.6588, 1675.7597, 1789.8050, 1804.7936, 1830.8140, 1843.8083, 1846.8021, 1854.8068, 1856.8190, 1859.8156, 1863.8240, 1871.8141, 1873.8084, 1874.8260, 1877.8133, 1886.8107, 1889.8230, 1901.8388, 1903.8212, 1904.8241, 1913.8384, 1916.8348, 1918.8216, 1920.8491, 1930.8525, 1932.8511, 1934.8641, 1936.8402, 1943.8467, 1946.8604, 1950.8451, 1959.8589, 1962.8577, 1978.8503, 2058.8800, 2072.9044, 2081.9145, 2084.9341, 2098.9058, 2114.9091, 2117.9278, 2127.9375, 2130.9411, 2133.8936, 2135.8843, 2139.9196, 2142.9347, 2145.9233, 2153.9357, 2156.9370, 2159.9208, 2160.2966, 2172.9286, 2174.9524, 2186.9516, 2188.9942, 2190.9396, 2200.9651, 2205.9396, 2208.9277, 2216.9604, 2231.9587, 2318.9909, 2346.0039, 2872.4359

13. VM3LC_MACLN **Mass:** 24215 **Score:** 25 **Expect:** 4.3 **Matches:** 5

Disintegrin-like leberagin-C OS=Macrovipera lebetina transmediterranea OX=384075 PE=1 SV=1

Observed	Mr(expt)	Mr(calc)	ppm	Start	End	Miss	Ions	Peptide
1229.6714	1228.6641	1228.5659	80.0	134	143	1	---	K.NGIGDFYCRK.Q
1262.6952	1261.6879	1261.6489	31.0	161	170	0	---	R.LFCEILPNTR.C
1368.6087	1367.6014	1367.6616	-44.02	55	66	1	---	R.FVTAGTECRATR.S
1514.6588	1513.6516	1513.7381	-57.18	148	160	1	---	R.LIPCAPEDVKCGR.L
1943.8467	1942.8394	1942.8149	12.6	173	190	1	---	K.HAPGDNGMVDPGTKCEDK.K + Oxidation (M)

No match to: 992.5466, 1030.5622, 1049.5703, 1063.5850, 1075.5835, 1091.5833, 1148.6528, 1174.5232, 1186.6320, 1203.6590, 1205.6684, 1246.6979, 1261.5132, 1318.5360, 1320.6106, 1355.6158, 1382.6064, 1389.6115, 1415.6205, 1427.6237, 1439.6265, 1443.6212, 1446.6351, 1457.6356, 1460.6468, 1472.6502, 1487.6537, 1492.7658, 1497.6465, 1675.7597, 1789.8050, 1804.7936, 1830.8140, 1843.8083, 1846.8021, 1854.8068, 1856.8190, 1859.8156, 1863.8240, 1871.8141, 1873.8084, 1874.8260, 1877.8133, 1886.8107, 1889.8230, 1901.8388, 1903.8212, 1904.8241, 1913.8384, 1916.8348, 1918.8216, 1920.8491, 1930.8525, 1932.8511, 1934.8641, 1936.8402, 1946.8604, 1950.8451, 1959.8589, 1962.8577, 1978.8503, 2058.8800, 2072.9044, 2081.9145, 2084.9341, 2098.9058, 2114.9091, 2117.9278, 2127.9375, 2130.9411, 2133.8936, 2135.8843, 2139.9196, 2142.9347, 2145.9233, 2153.9357, 2156.9370, 2159.9208, 2160.2966, 2172.9286, 2174.9524, 2186.9516, 2188.9942, 2190.9396, 2200.9651, 2205.9396, 2208.9277, 2216.9604, 2231.9587, 2318.9909, 2346.0039, 2361.9830, 2872.4359

14. Q8UVG8_DABRR **Mass:** 52931 **Score:** 24 **Expect:** 5.5 **Matches:** 7

Vimentin OS=Daboia russelii OX=8707 PE=2 SV=1

Observed	Mr(expt)	Mr(calc)	ppm	Start	End	Miss	Ions	Peptide
1148.6528	1147.6455	1147.5543	79.5	360	368	0	---	R.LQEEIQNMK.E + Oxidation (M)
1320.6106	1319.6033	1319.6252	-16.54	330	340	1	---	K.GTNESLERQMR.E
1439.6265	1438.6192	1438.7490	-90.16	386	397	1	---	K.MALDIEIATYRK.L + Oxidation (M)
1930.8525	1929.8452	1929.9327	-45.34	18	35	1	---	R.MFGSGRPSSTRSVTSTTR.Y + Oxidation (M)
2117.9278	2116.9206	2116.9623	-19.72	73	92	0	5	R.TGGDAMVDFSLADAINTEFK.A + Oxidation (M)
2156.9370	2155.9297	2155.9480	-8.48	341	359	0	---	R.EMEEAFGVEAANYQDNIAR.L
2172.9286	2171.9213	2171.9429	-9.95	341	359	0	---	R.EMEEAFGVEAANYQDNIAR.L + Oxidation (M)

No match to: 992.5466, 1030.5622, 1049.5703, 1063.5850, 1075.5835, 1091.5833, 1174.5232, 1186.6320, 1203.6590, 1205.6684, 1229.6714, 1246.6979, 1261.5132, 1262.6952, 1318.5360, 1355.6158, 1368.6087, 1382.6064, 1389.6115, 1415.6205, 1427.6237, 1443.6212, 1446.6351, 1457.6356, 1460.6468, 1472.6502, 1487.6537, 1492.7658, 1497.6465, 1514.6588, 1675.7597, 1789.8050, 1804.7936, 1830.8140, 1843.8083, 1846.8021, 1854.8068, 1856.8190, 1859.8156, 1863.8240, 1871.8141, 1873.8084, 1874.8260, 1877.8133, 1886.8107, 1889.8230, 1901.8388, 1903.8212, 1904.8241, 1913.8384, 1916.8348, 1918.8216, 1920.8491, 1932.8511, 1934.8641, 1936.8402, 1943.8467, 1946.8604, 1950.8451, 1959.8589, 1962.8577, 1978.8503, 2058.8800, 2072.9044, 2081.9145, 2084.9341, 2098.9058, 2114.9091, 2127.9375, 2130.9411, 2133.8936, 2135.8843, 2139.9196, 2142.9347, 2145.9233, 2153.9357, 2159.9208, 2160.2966, 2174.9524, 2186.9516, 2188.9942, 2190.9396, 2200.9651, 2205.9396, 2208.9277, 2216.9604, 2231.9587, 2318.9909, 2346.0039, 2361.9830, 2872.4359

15. F2Q6F2_DABRR **Mass:** 27583 **Score:** 24 **Expect:** 5.6 **Matches:** 7

Cysteine-rich seceretory protein Dr-CRPK OS=Daboia russelii OX=8707 PE=2 SV=1

Observed	Mr(expt)	Mr(calc)	ppm	Start	End	Miss	Ions	Peptide
1148.6528	1147.6455	1147.5907	47.8	47	57	0	---	R.SVTPTASNMLK.M
1320.6106	1319.6033	1319.6775	-56.17	105	114	0	---	K.WTAIIHEWHK.E
1920.8491	1919.8418	1919.9213	-41.39	71	85	1	---	R.WAFRCILNHSPYNSR.V
1932.8511	1931.8439	1932.0177	-89.98	30	45	0	---	R.RPEIQNEIVDLHNSLR.R
2156.9370	2155.9297	2156.0468	-54.32	86	104	1	---	R.VIGGIKCGENIYMSPYPMK.W
2172.9286	2171.9213	2172.0417	-55.44	86	104	1	---	R.VIGGIKCGENIYMSPYPMK.W + Oxidation (M)
2188.9942	2187.9869	2188.0367	-22.74	86	104	1	---	R.VIGGIKCGENIYMSPYPMK.W + 2 Oxidation (M)

No match to: 992.5466, 1030.5622, 1049.5703, 1063.5850, 1075.5835, 1091.5833, 1174.5232, 1186.6320, 1203.6590, 1205.6684, 1229.6714, 1246.6979, 1261.5132, 1262.6952, 1318.5360, 1355.6158, 1368.6087, 1382.6064, 1389.6115, 1415.6205, 1427.6237, 1439.6265, 1443.6212, 1446.6351, 1457.6356, 1460.6468, 1472.6502, 1487.6537, 1492.7658, 1497.6465, 1514.6588, 1675.7597, 1789.8050, 1804.7936, 1830.8140, 1843.8083, 1846.8021, 1854.8068, 1856.8190, 1859.8156, 1863.8240, 1871.8141, 1873.8084, 1874.8260, 1877.8133, 1886.8107, 1889.8230, 1901.8388, 1903.8212, 1904.8241, 1913.8384, 1916.8348, 1918.8216, 1930.8525, 1934.8641, 1936.8402, 1943.8467, 1946.8604, 1950.8451, 1959.8589, 1962.8577, 1978.8503, 2058.8800, 2072.9044, 2081.9145, 2084.9341, 2098.9058, 2114.9091, 2117.9278, 2127.9375, 2130.9411, 2133.8936, 2135.8843, 2139.9196, 2142.9347, 2145.9233, 2153.9357, 2159.9208, 2160.2966, 2174.9524, 2186.9516, 2190.9396, 2200.9651, 2205.9396, 2208.9277, 2216.9604, 2231.9587, 2318.9909, 2346.0039, 2361.9830, 2872.4359

16. B8QQ51_9PERO **Mass:** 33786 **Score:** 22 **Expect:** 8 **Matches:** 6

RNF213 (Fragment) OS=Echiichthys vipera OX=94984 GN=RNF213 PE=4 SV=1

Observed	Mr(expt)	Mr(calc)	ppm	Start	End	Miss	Ions	Peptide
1091.5833	1090.5760	1090.5692	6.24	232	240	1	---	K.EVLCDKTVK.G
1174.5232	1173.5159	1173.6263	-94.03	135	143	1	---	K.MLAIHMRFR.C
1262.6952	1261.6879	1261.6700	14.2	171	182	1	---	R.SGVASENMKLVK.V
1389.6115	1388.6043	1388.7089	-75.32	91	102	0	---	R.VPFNIDFDGLPR.G
1439.6265	1438.6192	1438.6762	-39.62	183	195	0	---	K.VHGGTTSEMIYTK.V + Oxidation (M)
2361.9830	2360.9757	2361.1569	-76.74	241	261	1	---	K.GESLTPNCGLQIIAACNPYRK.H

No match to: 992.5466, 1030.5622, 1049.5703, 1063.5850, 1075.5835, 1148.6528, 1186.6320, 1203.6590, 1205.6684, 1229.6714, 1246.6979, 1261.5132, 1318.5360, 1320.6106, 1355.6158, 1368.6087, 1382.6064, 1415.6205, 1427.6237, 1443.6212, 1446.6351, 1457.6356, 1460.6468, 1472.6502, 1487.6537, 1492.7658, 1497.6465, 1514.6588, 1675.7597, 1789.8050, 1804.7936, 1830.8140, 1843.8083, 1846.8021, 1854.8068, 1856.8190, 1859.8156, 1863.8240, 1871.8141, 1873.8084, 1874.8260, 1877.8133, 1886.8107, 1889.8230, 1901.8388, 1903.8212, 1904.8241, 1913.8384, 1916.8348, 1918.8216, 1920.8491, 1930.8525, 1932.8511, 1934.8641, 1936.8402, 1943.8467, 1946.8604, 1950.8451, 1959.8589, 1962.8577, 1978.8503, 2058.8800, 2072.9044, 2081.9145, 2084.9341, 2098.9058, 2114.9091, 2117.9278, 2127.9375, 2130.9411, 2133.8936, 2135.8843, 2139.9196, 2142.9347, 2145.9233, 2153.9357, 2156.9370, 2159.9208, 2160.2966, 2172.9286, 2174.9524, 2186.9516, 2188.9942, 2190.9396, 2200.9651, 2205.9396, 2208.9277, 2216.9604, 2231.9587, 2318.9909, 2346.0039, 2872.4359

17. A0A6G5ZVZ2_9SAUR **Mass:** 53939 **Score:** 19 **Expect:** 18 **Matches:** 7

Lactadherin 1 OS=Vipera anatolica senliki OX=2604287 PE=2 SV=1

Observed	Mr(expt)	Mr(calc)	ppm	Start	End	Miss	Ions	Peptide
1174.5232	1173.5159	1173.5753	-50.59	339	347	0	---	K.MTWHPYLAR.L
1439.6265	1438.6192	1438.7304	-77.23	320	332	0	---	R.ISDQQITASSVYK.T
1936.8402	1935.8329	1935.8026	15.7	105	119	0	---	K.HCQYNVNECLSQPCK.N
2114.9091	2113.9018	2113.8656	17.2	87	104	1	---	R.GDAFSEYMCICRSGYNGK.H
2117.9278	2116.9206	2116.9492	-13.54	82	98	1	---	R.LLPNRGDAFSEYMCICR.S + Oxidation (M)
2130.9411	2129.9338	2129.8605	34.4	87	104	1	---	R.GDAFSEYMCICRSGYNGK.H + Oxidation (M)
2172.9286	2171.9213	2172.0851	-75.42	320	338	1	---	R.ISDQQITASSVYKTWGFNK.M

No match to: 992.5466, 1030.5622, 1049.5703, 1063.5850, 1075.5835, 1091.5833, 1148.6528, 1186.6320, 1203.6590, 1205.6684, 1229.6714, 1246.6979, 1261.5132, 1262.6952, 1318.5360, 1320.6106, 1355.6158, 1368.6087, 1382.6064, 1389.6115, 1415.6205, 1427.6237, 1443.6212, 1446.6351, 1457.6356, 1460.6468, 1472.6502, 1487.6537, 1492.7658, 1497.6465, 1514.6588, 1675.7597, 1789.8050, 1804.7936, 1830.8140, 1843.8083, 1846.8021, 1854.8068, 1856.8190, 1859.8156, 1863.8240, 1871.8141, 1873.8084, 1874.8260, 1877.8133, 1886.8107, 1889.8230, 1901.8388, 1903.8212, 1904.8241, 1913.8384, 1916.8348, 1918.8216, 1920.8491, 1930.8525, 1932.8511, 1934.8641, 1943.8467, 1946.8604, 1950.8451, 1959.8589, 1962.8577, 1978.8503, 2058.8800, 2072.9044, 2081.9145, 2084.9341, 2098.9058, 2127.9375, 2133.8936, 2135.8843, 2139.9196, 2142.9347, 2145.9233, 2153.9357, 2156.9370, 2159.9208, 2160.2966, 2174.9524, 2186.9516, 2188.9942, 2190.9396, 2200.9651, 2205.9396, 2208.9277, 2216.9604, 2231.9587, 2318.9909, 2346.0039, 2361.9830, 2872.4359

18. A0A6G5ZW39_9SAUR **Mass:** 54634 **Score:** 19 **Expect:** 19 **Matches:** 7
Lactadherin 3 OS=Vipera anatolica senliki OX=2604287 PE=2 SV=1

Observed	Mr(expt)	Mr(calc)	ppm	Start	End	Miss	Ions	Peptide
1174.5232	1173.5159	1173.5753	-50.59	345	353	0	---	K.MTWHPYLAR.L
1439.6265	1438.6192	1438.7304	-77.23	326	338	0	---	R.ISDQQITASSVYK.T
1936.8402	1935.8329	1935.8026	15.7	105	119	0	---	K.HCQYNVNECLSQPCK.N
2114.9091	2113.9018	2113.8656	17.2	87	104	1	---	R.GDAFSEYMCICRSGYNGK.H
2117.9278	2116.9206	2116.9492	-13.54	82	98	1	---	R.LLPNRGDAFSEYMCICR.S + Oxidation (M)
2130.9411	2129.9338	2129.8605	34.4	87	104	1	---	R.GDAFSEYMCICRSGYNGK.H + Oxidation (M)
2172.9286	2171.9213	2172.0851	-75.42	326	344	1	---	R.ISDQQITASSVYKTWGFNK.M

No match to: 992.5466, 1030.5622, 1049.5703, 1063.5850, 1075.5835, 1091.5833, 1148.6528, 1186.6320, 1203.6590, 1205.6684, 1229.6714, 1246.6979, 1261.5132, 1262.6952, 1318.5360, 1320.6106, 1355.6158, 1368.6087, 1382.6064, 1389.6115, 1415.6205, 1427.6237, 1443.6212, 1446.6351, 1457.6356, 1460.6468, 1472.6502, 1487.6537, 1492.7658, 1497.6465, 1514.6588, 1675.7597, 1789.8050, 1804.7936, 1830.8140, 1843.8083, 1846.8021, 1854.8068, 1856.8190, 1859.8156, 1863.8240, 1871.8141, 1873.8084, 1874.8260, 1877.8133, 1886.8107, 1889.8230, 1901.8388, 1903.8212, 1904.8241, 1913.8384, 1916.8348, 1918.8216, 1920.8491, 1930.8525, 1932.8511, 1934.8641, 1943.8467, 1946.8604, 1950.8451, 1959.8589, 1962.8577, 1978.8503, 2058.8800, 2072.9044, 2081.9145, 2084.9341, 2098.9058, 2127.9375, 2133.8936, 2135.8843, 2139.9196, 2142.9347, 2145.9233, 2153.9357, 2156.9370, 2159.9208, 2160.2966, 2174.9524, 2186.9516, 2188.9942, 2190.9396, 2200.9651, 2205.9396, 2208.9277, 2216.9604, 2231.9587, 2318.9909, 2346.0039, 2361.9830, 2872.4359

19. PA2A8_DABPA **Mass:** 16194 **Score:** 18 **Expect:** 24 **Matches:** 5
Acidic phospholipase A2 VP8 OS=Daboia palaestinae OX=1170828 PE=3 SV=1

Observed	Mr(expt)	Mr(calc)	ppm	Start	End	Miss	Ions	Peptide
1918.8216	1917.8144	1917.7947	10.3	122	136	0	---	K.YMLHSLFDCMEESEK.C
1930.8525	1929.8452	1929.7662	40.9	32	48	0	---	K.SALSYSDYGCYCGWGGK.G
1934.8641	1933.8569	1933.7896	34.8	122	136	0	---	K.YMLHSLFDCMEESEK.C + Oxidation (M)
1950.8451	1949.8378	1949.7845	27.3	122	136	0	---	K.YMLHSLFDCMEESEK.C + 2 Oxidation (M)
2058.8800	2057.8727	2057.8611	5.62	31	48	1	---	R.KSALSYSDYGCYCGWGGK.G

No match to: 992.5466, 1030.5622, 1049.5703, 1063.5850, 1075.5835, 1091.5833, 1148.6528, 1174.5232, 1186.6320, 1203.6590, 1205.6684, 1229.6714, 1246.6979, 1261.5132, 1262.6952, 1318.5360, 1320.6106, 1355.6158, 1368.6087, 1382.6064, 1389.6115, 1415.6205, 1427.6237, 1439.6265, 1443.6212, 1446.6351, 1457.6356, 1460.6468, 1472.6502, 1487.6537, 1492.7658, 1497.6465, 1514.6588, 1675.7597, 1789.8050, 1804.7936, 1830.8140, 1843.8083, 1846.8021, 1854.8068, 1856.8190, 1859.8156, 1863.8240, 1871.8141, 1873.8084, 1874.8260, 1877.8133, 1886.8107, 1889.8230, 1901.8388, 1903.8212, 1904.8241, 1913.8384, 1916.8348, 1920.8491, 1932.8511, 1936.8402, 1943.8467, 1946.8604, 1959.8589, 1962.8577, 1978.8503, 2072.9044, 2081.9145, 2084.9341, 2098.9058, 2114.9091, 2117.9278, 2127.9375, 2130.9411, 2133.8936, 2135.8843, 2139.9196, 2142.9347, 2145.9233, 2153.9357, 2156.9370, 2159.9208, 2160.2966, 2172.9286, 2174.9524, 2186.9516, 2188.9942, 2190.9396, 2200.9651, 2205.9396, 2208.9277, 2216.9604, 2231.9587, 2318.9909, 2346.0039, 2361.9830, 2872.4359

20. A0A286S1C5_9SAUR **Mass:** 39024 **Score:** 18 **Expect:** 25 **Matches:** 6
E3 ubiquitin-protein ligase RAG1 (Fragment) OS=Vipera seoanei OX=110211 GN=RAG1 PE=4 SV=1

Observed	Mr(expt)	Mr(calc)	ppm	Start	End	Miss	Ions	Peptide
1049.5703	1048.5630	1048.4760	83.0	46	53	0	---	K.EVFHMSQR.E + Oxidation (M)
1075.5835	1074.5763	1074.5855	-8.63	209	217	1	---	K.QLNNKSLMK.K
1091.5833	1090.5760	1090.5804	-4.03	209	217	1	---	K.QLNNKSLMK.K + Oxidation (M)
1446.6351	1445.6279	1445.7119	-58.11	219	230	1	---	K.MSNCKQIHLSTK.V
1943.8467	1942.8394	1942.8955	-28.89	46	61	1	---	K.EVFHMSQRETEAHQAK.L + Oxidation (M)
1950.8451	1949.8378	1949.9768	-71.29	21	38	0	---	K.AAAIMSQVLFEADTELNK.N

No match to: 992.5466, 1030.5622, 1063.5850, 1148.6528, 1174.5232, 1186.6320, 1203.6590, 1205.6684, 1229.6714, 1246.6979, 1261.5132, 1262.6952, 1318.5360, 1320.6106, 1355.6158, 1368.6087, 1382.6064, 1389.6115, 1415.6205, 1427.6237, 1439.6265, 1443.6212, 1457.6356, 1460.6468, 1472.6502, 1487.6537, 1492.7658, 1497.6465, 1514.6588, 1675.7597, 1789.8050, 1804.7936, 1830.8140, 1843.8083, 1846.8021, 1854.8068, 1856.8190, 1859.8156, 1863.8240, 1871.8141, 1873.8084, 1874.8260, 1877.8133, 1886.8107, 1889.8230, 1901.8388, 1903.8212, 1904.8241, 1913.8384, 1916.8348, 1918.8216, 1920.8491, 1930.8525, 1932.8511, 1934.8641, 1936.8402, 1946.8604, 1959.8589, 1962.8577, 1978.8503, 2058.8800, 2072.9044, 2081.9145, 2084.9341, 2098.9058, 2114.9091, 2117.9278, 2127.9375, 2130.9411, 2133.8936, 2135.8843, 2139.9196, 2142.9347, 2145.9233, 2153.9357, 2156.9370, 2159.9208, 2160.2966, 2172.9286, 2174.9524, 2186.9516, 2188.9942, 2190.9396, 2200.9651, 2205.9396, 2208.9277, 2216.9604, 2231.9587, 2318.9909, 2346.0039, 2361.9830, 2872.4359

Search Parameters

```
Type of search         : MS/MS Ion Search
Enzyme                 : Trypsin
Fixed modifications    : Carbamidomethyl (C)
Variable modifications : Oxidation (M)
Mass values            : Monoisotopic
Protein Mass           : Unrestricted
Peptide Mass Tolerance : ± 100 ppm
Fragment Mass Tolerance: ± 0.7 Da
Max Missed Cleavages   : 1
Instrument type        : MALDI-TOF-TOF
Query1  (992.5466,1+)  : <no title>
Query2  (1030.5622,1+) : <no title>
Query3  (1049.5703,1+) : \\PROTEOMICS1\D\DATA\2105\210505_TLIEPOL_1_68252-68266\68252_68266_LPARDO\0_G8\1\1049.5703.LIFT\1SREF\PDATA\1\1R
Query4  (1063.5850,1+) : <no title>
Query5  (1075.5835,1+) : <no title>
Query6  (1091.5833,1+) : <no title>
Query7  (1148.6528,1+) : <no title>
Query8  (1174.5232,1+) : <no title>
Query9  (1186.6320,1+) : <no title>
Query10 (1203.6590,1+) : <no title>
Query11 (1205.6684,1+) : \\PROTEOMICS1\D\DATA\2105\210505_TLIEPOL_1_68252-68266\68252_68266_LPARDO\0_G8\1\1205.6685.LIFT\1SREF\PDATA\1\1R
Query12 (1229.6714,1+) : <no title>
Query13 (1246.6979,1+) : <no title>
Query14 (1261.5132,1+) : <no title>
Query15 (1262.6952,1+) : <no title>
Query16 (1318.5360,1+) : \\PROTEOMICS1\D\DATA\2105\210505_TLIEPOL_1_68252-68266\68252_68266_LPARDO\0_G8\1\1318.5360.LIFT\1SREF\PDATA\1\1R
Query17 (1320.6106,1+) : <no title>
Query18 (1355.6158,1+) : <no title>
Query19 (1368.6087,1+) : <no title>
Query20 (1382.6064,1+) : <no title>
Query21 (1389.6115,1+) : <no title>
Query22 (1415.6205,1+) : <no title>
Query23 (1427.6237,1+) : <no title>
Query24 (1439.6265,1+) : <no title>
Query25 (1443.6212,1+) : <no title>
Query26 (1446.6351,1+) : \\PROTEOMICS1\D\DATA\2105\210505_TLIEPOL_1_68252-68266\68252_68266_LPARDO\0_G8\1\1446.6351.LIFT\1SREF\PDATA\1\1R
Query27 (1457.6356,1+) : <no title>
Query28 (1460.6468,1+) : <no title>
Query29 (1472.6502,1+) : <no title>
Query30 (1487.6537,1+) : <no title>
Query31 (1492.7658,1+) : <no title>
Query32 (1497.6465,1+) : <no title>
Query33 (1514.6588,1+) : <no title>
Query34 (1675.7597,1+) : <no title>
Query35 (1789.8050,1+) : \\PROTEOMICS1\D\DATA\2105\210505_TLIEPOL_1_68252-68266\68252_68266_LPARDO\0_G8\1\1789.8050.LIFT\1SREF\PDATA\1\1R
Query36 (1804.7936,1+) : <no title>
Query37 (1830.8140,1+) : <no title>
Query38 (1843.8083,1+) : <no title>
Query39 (1846.8021,1+) : <no title>
Query40 (1854.8068,1+) : <no title>
```

```
Query41 (1856.8190,1+) : <no title>
Query42 (1859.8156,1+) : <no title>
Query43 (1863.8240,1+) : <no title>
Query44 (1871.8141,1+) : <no title>
Query45 (1873.8084,1+) : <no title>
Query46 (1874.8260,1+) : <no title>
Query47 (1877.8133,1+) : <no title>
Query48 (1886.8107,1+) : <no title>
Query49 (1889.8230,1+) : <no title>
Query50 (1901.8388,1+) : <no title>
Query51 (1903.8212,1+) : <no title>
Query52 (1904.8241,1+) : <no title>
Query53 (1913.8384,1+) : <no title>
Query54 (1916.8348,1+) : <no title>
Query55 (1918.8216,1+) : <no title>
Query56 (1920.8491,1+) : \\PROTEOMICS1\D\DATA\2105\210505_TLIEPOL_1_68252-68266\68252_68266_LPARDO\0_G8\1\1920.8491.LIFT\1SREF\PDATA\1\1R
Query57 (1930.8525,1+) : <no title>
Query58 (1932.8511,1+) : <no title>
Query59 (1934.8641,1+) : <no title>
Query60 (1936.8402,1+) : \\PROTEOMICS1\D\DATA\2105\210505_TLIEPOL_1_68252-68266\68252_68266_LPARDO\0_G8\1\1936.8402.LIFT\1SREF\PDATA\1\1R
Query61 (1943.8467,1+) : <no title>
Query62 (1946.8604,1+) : <no title>
Query63 (1950.8451,1+) : <no title>
Query64 (1959.8589,1+) : <no title>
Query65 (1962.8577,1+) : <no title>
Query66 (1978.8503,1+) : <no title>
Query67 (2058.8800,1+) : <no title>
Query68 (2072.9044,1+) : <no title>
Query69 (2081.9145,1+) : <no title>
Query70 (2084.9341,1+) : <no title>
Query71 (2098.9058,1+) : <no title>
Query72 (2114.9091,1+) : <no title>
Query73 (2117.9278,1+) : \\PROTEOMICS1\D\DATA\2105\210505_TLIEPOL_1_68252-68266\68252_68266_LPARDO\0_G8\1\2117.9278.LIFT\1SREF\PDATA\1\1R
Query74 (2127.9375,1+) : <no title>
Query75 (2130.9411,1+) : <no title>
Query76 (2133.8936,1+) : <no title>
Query77 (2135.8843,1+) : <no title>
Query78 (2139.9196,1+) : <no title>
Query79 (2142.9347,1+) : <no title>
Query80 (2145.9233,1+) : <no title>
Query81 (2153.9357,1+) : <no title>
Query82 (2156.9370,1+) : <no title>
Query83 (2159.9208,1+) : <no title>
Query84 (2160.2966,1+) : <no title>
Query85 (2172.9286,1+) : <no title>
Query86 (2174.9524,1+) : \\PROTEOMICS1\D\DATA\2105\210505_TLIEPOL_1_68252-68266\68252_68266_LPARDO\0_G8\1\2174.9524.LIFT\1SREF\PDATA\1\1R
Query87 (2186.9516,1+) : <no title>
Query88 (2188.9942,1+) : <no title>
Query89 (2190.9396,1+) : \\PROTEOMICS1\D\DATA\2105\210505_TLIEPOL_1_68252-68266\68252_68266_LPARDO\0_G8\1\2190.9396.LIFT\1SREF\PDATA\1\1R
Query90 (2200.9651,1+) : \\PROTEOMICS1\D\DATA\2105\210505_TLIEPOL_1_68252-68266\68252_68266_LPARDO\0_G8\1\2200.9651.LIFT\1SREF\PDATA\1\1R
Query91 (2205.9396,1+) : <no title>
Query92 (2208.9277,1+) : <no title>
Query93 (2216.9604,1+) : \\PROTEOMICS1\D\DATA\2105\210505_TLIEPOL_1_68252-68266\68252_68266_LPARDO\0_G8\1\2216.9604.LIFT\1SREF\PDATA\1\1R
Query94 (2231.9587,1+) : <no title>
Query95 (2318.9909,1+) : <no title>
Query96 (2346.0039,1+) : <no title>
Query97 (2361.9830,1+) : <no title>
Query98 (2872.4359,1+) : <no title>
```

Mascot: http://www.matrixscience.com/

Mascot Search Results Fr5-1

```
User            :
Email           :
Search title    : vipera_All_8IDplus4 file:\\Proteomics1\d\data\2105\210505_tliepol_1_68252-68266\68252_68266_lpardo\0_H8\1\1SRef\pdata\1\peaklist.xml
Database        : vipera  (1414 sequences; 347452 residues)
Timestamp       : 5 May 2021 at 09:35:21 GMT
Top Score       : 49 for A0A6G5ZW74_9SAUR, Veficolin 9 (Fragment) OS=Vipera anatolica senliki OX=2604287 PE=2 SV=1
```

Mascot Score Histogram

Protein score is -10*Log(P), where P is the probability that the observed match is a random event.
Protein scores greater than 44 are significant (p<0.05).

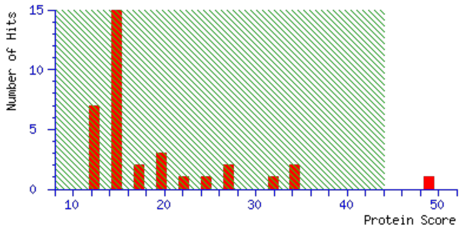

Concise Protein Summary Report

Format As | Concise Protein Summary | Help

Significance threshold p< 0.05 Max. number of hits 10

Re-Search All | Search Unmatched

1. A0A6G5ZW74_9SAUR **Mass:** 32808 **Score:** 49 **Expect:** 0.018 **Matches:** 10
   Veficolin 9 (Fragment) OS=Vipera anatolica senliki OX=2604287 PE=2 SV=1
   A0A6G5ZVU3_9SAUR **Mass:** 11896 **Score:** 27 **Expect:** 3 **Matches:** 4
   Veficolin 4 (Fragment) OS=Vipera anatolica senliki OX=2604287 PE=2 SV=1
   A0A6G5ZUK1_9SAUR **Mass:** 15084 **Score:** 19 **Expect:** 19 **Matches:** 4
   Veficolin 7 (Fragment) OS=Vipera anatolica senliki OX=2604287 PE=2 SV=1

2. A0A6G5ZVX6_9SAUR **Mass:** 37165 **Score:** 35 **Expect:** 0.44 **Matches:** 8
   Veficolin 1 OS=Vipera anatolica senliki OX=2604287 PE=2 SV=1

3. A0A6G5ZVY6_9SAUR **Mass:** 24441 **Score:** 34 **Expect:** 0.6 **Matches:** 7
   Veficolin 6 (Fragment) OS=Vipera anatolica senliki OX=2604287 PE=2 SV=1
   A0A6G5ZUK5_9SAUR **Mass:** 26961 **Score:** 31 **Expect:** 1.2 **Matches:** 7
   Veficolin 5 (Fragment) OS=Vipera anatolica senliki OX=2604287 PE=2 SV=1

4. A0A6G5ZW20_9SAUR **Mass:** 26588 **Score:** 31 **Expect:** 1.1 **Matches:** 6
   Veficolin 8 (Fragment) OS=Vipera anatolica senliki OX=2604287 PE=2 SV=1

5. Q8UVG8_DABRR **Mass:** 52931 **Score:** 28 **Expect:** 2.3 **Matches:** 9
   Vimentin OS=Daboia russelii OX=8707 PE=2 SV=1

6. DIS_MACLO **Mass:** 4854 **Score:** 27 **Expect:** 2.8 **Matches:** 3
   Disintegrin obtustatin OS=Macrovipera lebetina obtusa OX=209528 PE=1 SV=1
   DIS_DABPA **Mass:** 4914 **Score:** 16 **Expect:** 35 **Matches:** 2
   Disintegrin viperistatin OS=Daboia palaestinae OX=1170828 PE=1 SV=1

7. A0A6G5ZVP7_9SAUR **Mass:** 59614 **Score:** 26 **Expect:** 3.9 **Matches:** 8
   Amine oxidase OS=Vipera anatolica senliki OX=2604287 PE=2 SV=1

8. A0A1I9KNP4_VIPAA **Mass:** 20248 **Score:** 21 **Expect:** 11 **Matches:** 4
   Endogenous tripeptide metalloproteinase inhibitor OS=Vipera ammodytes ammodytes OX=8705 PE=2 SV=1
   A0A6B7FPT0_VIPAA **Mass:** 15144 **Score:** 16 **Expect:** 39 **Matches:** 3
   Endogenous tripeptide metalloproteinase inhibitor MPi-5 OS=Vipera ammodytes ammodytes OX=8705 PE=2 SV=1
   A0A1J0CZN1_VIPAA **Mass:** 20176 **Score:** 14 **Expect:** 50 **Matches:** 3
   SVMPi-1 OS=Vipera ammodytes ammodytes OX=8705 PE=2 SV=1

9. A0A6G5ZVL0_9SAUR **Mass:** 21283 **Score:** 20 **Expect:** 16 **Matches:** 4
   Cholinesterase 14 (Fragment) OS=Vipera anatolica senliki OX=2604287 PE=2 SV=1
   A0A6G5ZVP1_9SAUR **Mass:** 29798 **Score:** 16 **Expect:** 33 **Matches:** 4
   Cholinesterase 8 (Fragment) OS=Vipera anatolica senliki OX=2604287 PE=2 SV=1

10. A0A6G5ZUD5_9SAUR **Mass:** 21508 **Score:** 19 **Expect:** 17 **Matches:** 4
    Lysosomal acid lipase/cholesteryl ester hydrolase 3 (Fragment) OS=Vipera anatolica senliki OX=2604287 PE=2 SV=1
    A0A6G5ZUD1_9SAUR **Mass:** 30200 **Score:** 14 **Expect:** 50 **Matches:** 4
    Lysosomal acid lipase/cholesteryl ester hydrolase 2 (Fragment) OS=Vipera anatolica senliki OX=2604287 PE=2 SV=1

Search Parameters

```
Type of search         : Peptide Mass Fingerprint
Enzyme                 : Trypsin
Fixed modifications    : Carbamidomethyl (C)
Variable modifications : Oxidation (M)
Mass values            : Monoisotopic
Protein Mass           : Unrestricted
Peptide Mass Tolerance : ± 100 ppm
Peptide Charge State   : 1+
Max Missed Cleavages   : 1
Number of queries      : 95
```

Mascot: http://www.matrixscience.com/

MATRIX SCIENCE Mascot Search Results

```
User          :
Email         :
Search title  : vipera_all_Lift file:\\Proteomics1\d\data\2105\210505_tliepol_1_68252-68266\68252_68266_lpardo\0_H8\1\0_H8.xml
Database      : vipera  (1414 sequences; 347452 residues)
Timestamp     : 5 May 2021 at 09:54:48 GMT
Protein hits  : A0A6G5ZUK1_9SAUR Veficolin 7 (Fragment) OS=Vipera anatolica senliki OX=2604287 PE=2 SV=1
                VSPY_MACLB       Chymotrypsin-like protease VLCTLP OS=Macrovipera lebetina OX=8709 PE=1 SV=1
                A0A6G5ZUD1_9SAUR Lysosomal acid lipase/cholesteryl ester hydrolase 2 (Fragment) OS=Vipera anatolica senliki OX=2604287 PE=2 SV=1
                A0A223PK32_DABRR Atrial natriuretic peptide receptor 3 (Fragment) OS=Daboia russelii OX=8707 PE=2 SV=1
```

Mascot Score Histogram

Ions score is -10*Log(P), where P is the probability that the observed match is a random event.
Individual ions scores > 11 indicate identity or extensive homology (p<0.05).
Protein scores are derived from ions scores as a non-probabilistic basis for ranking protein hits.

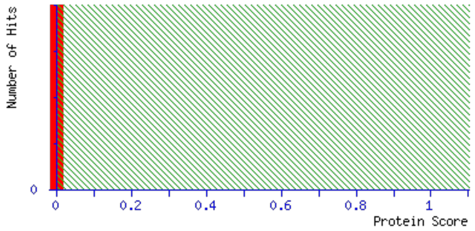

Peptide Summary Report

Format As | Peptide Summary | Help

Significance threshold p< 0.05 | Max. number of hits 10

Standard scoring ○ MudPIT scoring ◉ | Ions score or expect cut-off 0 | Show sub-sets 0

Show pop-ups ◉ Suppress pop-ups ○ | Sort unassigned Decreasing Score | Require bold red ☐

Overview Table

Click on column header to jump to entry in results list.
Move mouse over any indicator to highlight identical peptides.
Click on an indicator to see details of individual match.
Use check boxes to select sub-set of queries for new search.

Mouse over: -Query- -Accession- -Sequence-

Hit:	1	2	3	4
☑ 823.5447 (1+)				
☑ 855.0830 (1+)				
☑ 871.0563 (1+)				
☑ 877.0762 (1+)				
☑ 914.5519 (1+)				
☑ 952.4441 (1+)				
☑ 1004.4711 (1+)				
☑ 1011.4796 (1+)				
☑ 1022.1389 (1+)				
☑ 1022.4856 (1+)				
☑ 1044.1219 (1+)				
☑ 1049.4975 (1+)				
☑ 1068.5036 (1+)				
☑ 1079.5006 (1+)				
☑ 1082.0759 (1+)				
☑ 1082.5129 (1+)				
☑ 1094.5136 (1+)				
☑ 1106.4504 (1+)				
☑ 1155.6361 (1+)				
☑ 1156.6134 (1+)				
☑ 1157.6541 (1+)	●		●	
☑ 1161.6459 (1+)				
☑ 1165.6377 (1+)				
☑ 1171.6321 (1+)		●		
☑ 1173.6479 (1+)				
☑ 1177.6418 (1+)				
☑ 1179.6312 (1+)				
☑ 1183.6566 (1+)				
☑ 1185.6366 (1+)				
☑ 1187.6289 (1+)				
☑ 1189.6426 (1+)				
☑ 1193.6359 (1+)				
☑ 1195.5982 (1+)				

☑ 1199.6461 (1+)				
☑ 1202.6295 (1+)				
☑ 1203.6124 (1+)				
☑ 1205.6366 (1+)				
☑ 1211.6005 (1+)				
☑ 1218.6238 (1+)				
☑ 1220.4949 (1+)				
☑ 1221.6319 (1+)				
☑ 1227.5952 (1+)				
☑ 1237.6244 (1+)				
☑ 1255.6452 (1+)				
☑ 1277.7454 (1+)				
☑ 1282.6532 (1+)				
☑ 1285.7510 (1+)				
☑ 1291.9938 (1+)				
☑ 1293.5963 (1+)				
☑ 1301.7432 (1+)				
☑ 1305.7277 (1+)				
☑ 1311.7648 (1+)				
☑ 1315.7213 (1+)				
☑ 1317.7360 (1+)				
☑ 1327.7567 (1+)				
☑ 1330.5539 (1+)				
☑ 1343.7528 (1+)				
☑ 1344.5305 (1+)				
☑ 1346.5454 (1+)				
☑ 1349.7244 (1+)				
☑ 1350.5366 (1+)				
☑ 1356.5595 (1+)				
☑ 1359.5254 (1+)				
☑ 1362.5398 (1+)				
☑ 1368.5033 (1+)				
☑ 1372.5583 (1+)				
☑ 1378.5329 (1+)				
☑ 1384.4896 (1+)				
☑ 1394.5236 (1+)				
☑ 1475.7859 (1+)				
☑ 1497.7946 (1+)				
☑ 1519.7800 (1+)				
☑ 1535.7454 (1+)				
☑ 1592.7495 (1+)				
☑ 1621.7328 (1+)				
☑ 1625.8878 (1+)				
☑ 1636.7491 (1+)				●
☑ 1651.7516 (1+)				
☑ 1658.7257 (1+)				
☑ 1674.6922 (1+)				
☑ 1678.7590 (1+)				
☑ 1698.8238 (1+)				
☑ 1712.6354 (1+)				
☑ 1724.8379 (1+)				
☑ 1740.8289 (1+)				
☑ 1764.8372 (1+)				
☑ 1994.0179 (1+)				
☑ 2036.9304 (1+)				
☑ 2052.9252 (1+)				
☑ 2074.9097 (1+)				
☑ 2134.9820 (1+)				
☑ 2150.9758 (1+)				
☑ 2166.9679 (1+)				
☑ 2172.9499 (1+)				
☑ 2682.1857 (1+)				

Select All | Select None | Search Selected | ☐ **Error tolerant** | Archive Report

1. A0A6G5ZUK1_9SAUR **Mass:** 15084 **Score:** 0 **Matches:** 1(0) **Sequences:** 1(0)
Veficolin 7 (Fragment) OS=Vipera anatolica senliki OX=2604287 PE=2 SV=1
☐ Check to include this hit in error tolerant search or archive report

Query	Observed	Mr(expt)	Mr(calc)	ppm	Miss	Score	Expect	Rank	Unique	Peptide
21	1157.6541	1156.6468	1156.6088	32.9	1	4	0.37	1	U	K.DKLQDPNSLK.C

Proteins matching the same set of peptides:
A0A6G5ZVU3_9SAUR **Mass:** 11896 **Score:** 0 **Matches:** 1(0) **Sequences:** 1(0)
Veficolin 4 (Fragment) OS=Vipera anatolica senliki OX=2604287 PE=2 SV=1
A0A6G5ZVX6_9SAUR **Mass:** 37165 **Score:** 0 **Matches:** 1(0) **Sequences:** 1(0)
Veficolin 1 OS=Vipera anatolica senliki OX=2604287 PE=2 SV=1
A0A6G5ZW74_9SAUR **Mass:** 32808 **Score:** 0 **Matches:** 1(0) **Sequences:** 1(0)
Veficolin 9 (Fragment) OS=Vipera anatolica senliki OX=2604287 PE=2 SV=1

2. VSPY_MACLB **Mass:** 28944 **Score:** 0 **Matches:** 1(0) **Sequences:** 1(0)
Chymotrypsin-like protease VLCTLP OS=Macrovipera lebetina OX=8709 PE=1 SV=1
☐ Check to include this hit in error tolerant search or archive report

Query	Observed	Mr(expt)	Mr(calc)	ppm	Miss	Score	Expect	Rank	Unique	Peptide
24	**1171.6321**	**1170.6248**	**1170.6105**	**12.3**	**1**	**3**	**0.46**	**1**	**U**	**R.LRNQDEQIR.V**

3. A0A6G5ZUD1_9SAUR **Mass:** 30200 **Score:** 0 **Matches:** 1(0) **Sequences:** 1(0)
Lysosomal acid lipase/cholesteryl ester hydrolase 2 (Fragment) OS=Vipera anatolica senliki OX=2604287 PE=2 SV=1
☐ Check to include this hit in error tolerant search or archive report

Query	Observed	Mr(expt)	Mr(calc)	ppm	Miss	Score	Expect	Rank	Unique	Peptide
21	1157.6541	1156.6468	1156.6604	-11.77	0	3	0.49	2	U	K.QFLPQNAVIK.W

Proteins matching the same set of peptides:
A0A6G5ZUD5_9SAUR **Mass:** 21508 **Score:** 0 **Matches:** 1(0) **Sequences:** 1(0)
Lysosomal acid lipase/cholesteryl ester hydrolase 3 (Fragment) OS=Vipera anatolica senliki OX=2604287 PE=2 SV=1
A0A6G5ZVQ9_9SAUR **Mass:** 46045 **Score:** 0 **Matches:** 1(0) **Sequences:** 1(0)
Lipase OS=Vipera anatolica senliki OX=2604287 PE=2 SV=1

4. A0A223PK32_DABRR **Mass:** 57907 **Score:** 0 **Matches:** 1(0) **Sequences:** 1(0)
Atrial natriuretic peptide receptor 3 (Fragment) OS=Daboia russelii OX=8707 PE=2 SV=1
☐ Check to include this hit in error tolerant search or archive report

Query	Observed	Mr(expt)	Mr(calc)	ppm	Miss	Score	Expect	Rank	Unique	Peptide
77	**1636.7491**	**1635.7419**	**1635.7100**	**19.5**	**1**	**2**	**0.67**	**1**	**U**	**K.FRLFYSDSDCGNR.A**

Peptide matches not assigned to protein hits: (no details means no match)

	Query	Observed	Mr(expt)	Mr(calc)	ppm	Miss	Score	Expect	Rank	Unique	Peptide
☑	1	*823.5447*	*822.5374*								
☑	2	*855.0830*	*854.0758*								
☑	3	*871.0563*	*870.0490*								
☑	4	*877.0762*	*876.0689*								
☑	5	*914.5519*	*913.5446*								
☑	6	*952.4441*	*951.4369*								
☑	7	*1004.4711*	*1003.4638*								
☑	8	*1011.4796*	*1010.4723*								
☑	9	*1022.1389*	*1021.1316*								
☑	10	*1022.4856*	*1021.4784*								
☑	11	*1044.1219*	*1043.1146*								
☑	12	*1049.4975*	*1048.4902*								
☑	13	*1068.5036*	*1067.4963*								
☑	14	*1079.5006*	*1078.4933*								
☑	15	*1082.0759*	*1081.0686*								
☑	16	*1082.5129*	*1081.5056*								
☑	17	*1094.5136*	*1093.5063*								
☑	18	*1106.4504*	*1105.4432*								
☑	19	*1155.6361*	*1154.6288*								
☑	20	*1156.6134*	*1155.6061*								
☑	22	*1161.6459*	*1160.6386*								
☑	23	*1165.6377*	*1164.6304*								
☑	25	*1173.6479*	*1172.6406*								
☑	26	*1177.6418*	*1176.6345*								
☑	27	*1179.6312*	*1178.6239*								
☑	28	*1183.6566*	*1182.6493*								
☑	29	*1185.6366*	*1184.6293*								
☑	30	*1187.6289*	*1186.6216*								
☑	31	*1189.6426*	*1188.6353*								
☑	32	*1193.6359*	*1192.6286*								
☑	33	*1195.5982*	*1194.5909*								
☑	34	*1199.6461*	*1198.6388*								
☑	35	*1202.6295*	*1201.6222*								
☑	36	*1203.6124*	*1202.6051*								
☑	37	*1205.6366*	*1204.6293*								
☑	38	*1211.6005*	*1210.5932*								
☑	39	*1218.6238*	*1217.6165*								
☑	40	*1220.4949*	*1219.4877*								
☑	41	*1221.6319*	*1220.6246*								
☑	42	*1227.5952*	*1226.5879*								
☑	43	*1237.6244*	*1236.6171*								
☑	44	*1255.6452*	*1254.6380*								
☑	45	*1277.7454*	*1276.7381*								
☑	46	*1282.6532*	*1281.6459*								
☑	47	*1285.7510*	*1284.7437*								
☑	48	*1291.9938*	*1290.9865*								
☑	49	*1293.5963*	*1292.5890*								
☑	50	*1301.7432*	*1300.7360*								
☑	51	*1305.7277*	*1304.7204*								
☑	52	*1311.7648*	*1310.7575*								
☑	53	*1315.7213*	*1314.7140*								
☑	54	*1317.7360*	*1316.7287*								
☑	55	*1327.7567*	*1326.7494*								
☑	56	*1330.5539*	*1329.5466*								
☑	57	*1343.7528*	*1342.7455*								
☑	58	*1344.5305*	*1343.5232*								
☑	59	*1346.5454*	*1345.5381*								
☑	60	*1349.7244*	*1348.7171*								

☑	61	*1350.5366*	*1349.5293*
☑	62	*1356.5595*	*1355.5522*
☑	63	*1359.5254*	*1358.5182*
☑	64	*1362.5398*	*1361.5326*
☑	65	*1368.5033*	*1367.4960*
☑	66	*1372.5583*	*1371.5510*
☑	67	*1378.5329*	*1377.5256*
☑	68	*1384.4896*	*1383.4823*
☑	69	*1394.5236*	*1393.5163*
☑	70	*1475.7859*	*1474.7786*
☑	71	*1497.7946*	*1496.7873*
☑	72	*1519.7800*	*1518.7727*
☑	73	*1535.7454*	*1534.7381*
☑	74	*1592.7495*	*1591.7423*
☑	75	*1621.7328*	*1620.7255*
☑	76	*1625.8878*	*1624.8805*
☑	78	*1651.7516*	*1650.7444*
☑	79	*1658.7257*	*1657.7184*
☑	80	*1674.6922*	*1673.6849*
☑	81	*1678.7590*	*1677.7517*
☑	82	*1698.8238*	*1697.8165*
☑	83	*1712.6354*	*1711.6281*
☑	84	*1724.8379*	*1723.8306*
☑	85	*1740.8289*	*1739.8217*
☑	86	*1764.8372*	*1763.8300*
☑	87	*1994.0179*	*1993.0106*
☑	88	*2036.9304*	*2035.9231*
☑	89	*2052.9252*	*2051.9179*
☑	90	*2074.9097*	*2073.9024*
☑	91	*2134.9820*	*2133.9747*
☑	92	*2150.9758*	*2149.9685*
☑	93	*2166.9679*	*2165.9607*
☑	94	*2172.9499*	*2171.9426*
☑	95	*2682.1857*	*2681.1785*

Search Parameters

```
Type of search         : MS/MS Ion Search
Enzyme                 : Trypsin
Fixed modifications    : Carbamidomethyl (C)
Variable modifications : Oxidation (M)
Mass values            : Monoisotopic
Protein Mass           : Unrestricted
Peptide Mass Tolerance : ± 100 ppm
Fragment Mass Tolerance: ± 0.7 Da
Max Missed Cleavages   : 1
Instrument type        : MALDI-TOF-TOF
Number of queries      : 95
```

Mascot: http://www.matrixscience.com/

MATRIX SCIENCE Mascot Search Results

```
User          :
Email         :
Search title  : vipera_all_Lift file:\\Proteomics1\d\data\2105\210505_tliepol_1_68252-68266\68252_68266_lpardo\0_H8\1\0_H8.xml
Database      : vipera  (1414 sequences; 347452 residues)
Timestamp     : 5 May 2021 at 09:54:48 GMT
Warning       : A Peptide summary report will usually give a much clearer picture of MS/MS search results.
Top Score     : 53 for A0A6G5ZW74_9SAUR, Veficolin 9 (Fragment) OS=Vipera anatolica senliki OX=2604287 PE=2 SV=1
```

Mascot Score Histogram

Protein score is -10*Log(P), where P is the probability that the observed match is a random event.
Protein scores greater than 44 are significant (p<0.05).
Protein scores are derived from ions scores as a non-probabilistic basis for ranking protein hits.

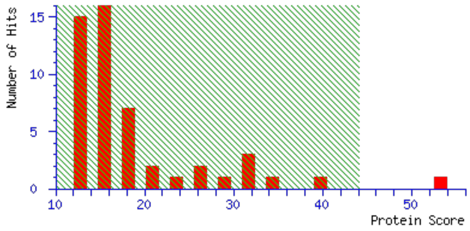

Protein Summary Report

Format As | Protein Summary (deprecated) | Help

Significance threshold p< 0.05 Max. number of hits 20

Overview Table

Click on column header to jump to entry in results list.
Move mouse over any indicator to highlight identical peptides.
Click on an indicator to see details of individual match.
Use check boxes to select sub-set of queries for new search.

Mouse over: -Query-
-Accession- -Sequence-

Hit:	1	2	3	4	5	6	7	8	9	10	11	12	13	14	15	16	17	18	19	20
☑ 823.5447 (1+)																				
☑ 855.0830 (1+)																				
☑ 871.0563 (1+)																				
☑ 877.0762 (1+)																				
☑ 914.5519 (1+)																				
☑ 952.4441 (1+)																				
☑ 1004.4711 (1+)																				
☑ 1011.4796 (1+)																				
☑ 1022.1389 (1+)																				
☑ 1022.4856 (1+)																				
☑ 1044.1219 (1+)																				
☑ 1049.4975 (1+)																				
☑ 1068.5036 (1+)																				
☑ 1079.5006 (1+)																				
☑ 1082.0759 (1+)																				
☑ 1082.5129 (1+)																				
☑ 1094.5136 (1+)																				
☑ 1106.4504 (1+)																				
☑ 1155.6361 (1+)																				
☑ 1156.6134 (1+)																				
☑ 1157.6541 (1+)	●	●		●						●	●					●				
☑ 1161.6459 (1+)																				
☑ 1165.6377 (1+)																				
☑ 1171.6321 (1+)																				
☑ 1173.6479 (1+)																				
☑ 1177.6418 (1+)																				
☑ 1179.6312 (1+)																				
☑ 1183.6566 (1+)																				
☑ 1185.6366 (1+)																				
☑ 1187.6289 (1+)																				
☑ 1189.6426 (1+)																				
☑ 1193.6359 (1+)																				
☑ 1195.5982 (1+)																				
☑ 1199.6461 (1+)																				
☑ 1202.6295 (1+)																				
☑ 1203.6124 (1+)																				
☑ 1205.6366 (1+)																				
☑ 1211.6005 (1+)																				
☑ 1218.6238 (1+)																				

☑ 1220.4949 (1+)																				
☑ 1221.6319 (1+)																				
☑ 1227.5952 (1+)																				
☑ 1237.6244 (1+)																				
☑ 1255.6452 (1+)																				
☑ 1277.7454 (1+)																				
☑ 1282.6532 (1+)																				
☑ 1285.7510 (1+)																				
☑ 1291.9938 (1+)																				
☑ 1293.5963 (1+)																				
☑ 1301.7432 (1+)																				
☑ 1305.7277 (1+)																				
☑ 1311.7648 (1+)																				
☑ 1315.7213 (1+)																				
☑ 1317.7360 (1+)																				
☑ 1327.7567 (1+)																				
☑ 1330.5539 (1+)																				
☑ 1343.7528 (1+)																				
☑ 1344.5305 (1+)																				
☑ 1346.5454 (1+)																				
☑ 1349.7244 (1+)																				
☑ 1350.5366 (1+)																				
☑ 1356.5595 (1+)																				
☑ 1359.5254 (1+)																				
☑ 1362.5398 (1+)																				
☑ 1368.5033 (1+)																				
☑ 1372.5583 (1+)																				
☑ 1378.5329 (1+)																				
☑ 1384.4896 (1+)																				
☑ 1394.5236 (1+)																				
☑ 1475.7859 (1+)																				
☑ 1497.7946 (1+)																				
☑ 1519.7800 (1+)																				
☑ 1535.7454 (1+)																				
☑ 1592.7495 (1+)																				
☑ 1621.7328 (1+)																				
☑ 1625.8878 (1+)																				
☑ 1636.7491 (1+)																				
☑ 1651.7516 (1+)																				
☑ 1658.7257 (1+)																				
☑ 1674.6922 (1+)																				
☑ 1678.7590 (1+)																				
☑ 1698.8238 (1+)																				
☑ 1712.6354 (1+)																				
☑ 1724.8379 (1+)																				
☑ 1740.8289 (1+)																				
☑ 1764.8372 (1+)																				
☑ 1994.0179 (1+)																				
☑ 2036.9304 (1+)																				
☑ 2052.9252 (1+)																				
☑ 2074.9097 (1+)																				
☑ 2134.9820 (1+)																				
☑ 2150.9758 (1+)																				
☑ 2166.9679 (1+)																				
☑ 2172.9499 (1+)																				
☑ 2682.1857 (1+)																				

Select All | Select None | Search Selected

Index

	Accession	Mass	Score	Description
1.	A0A6G5ZW74_9SAUR	32808	53	Veficolin 9 (Fragment) OS=Vipera anatolica senliki OX=2604287 PE=2 SV=1
2.	A0A6G5ZVX6_9SAUR	37165	39	Veficolin 1 OS=Vipera anatolica senliki OX=2604287 PE=2 SV=1
3.	A0A6G5ZVY6_9SAUR	24441	34	Veficolin 6 (Fragment) OS=Vipera anatolica senliki OX=2604287 PE=2 SV=1
4.	A0A6G5ZVU3_9SAUR	11896	31	Veficolin 4 (Fragment) OS=Vipera anatolica senliki OX=2604287 PE=2 SV=1
5.	A0A6G5ZW20_9SAUR	26588	31	Veficolin 8 (Fragment) OS=Vipera anatolica senliki OX=2604287 PE=2 SV=1
6.	A0A6G5ZUK5_9SAUR	26961	31	Veficolin 5 (Fragment) OS=Vipera anatolica senliki OX=2604287 PE=2 SV=1
7.	Q8UVG8_DABRR	52931	28	Vimentin OS=Daboia russelii OX=8707 PE=2 SV=1
8.	DIS_MACLO	4854	27	Disintegrin obtustatin OS=Macrovipera lebetina obtusa OX=209528 PE=1 SV=1
9.	A0A6G5ZVP7_9SAUR	59614	26	Amine oxidase OS=Vipera anatolica senliki OX=2604287 PE=2 SV=1
10.	A0A6G5ZUK1_9SAUR	15084	23	Veficolin 7 (Fragment) OS=Vipera anatolica senliki OX=2604287 PE=2 SV=1
11.	A0A6G5ZUD5_9SAUR	21508	22	Lysosomal acid lipase/cholesteryl ester hydrolase 3 (Fragment) OS=Vipera anatolica senliki OX=2604287 PE=2 SV=1
12.	A0A1I9KNP4_VIPAA	20248	21	Endogenous tripeptide metalloproteinase inhibitor OS=Vipera ammodytes ammodytes OX=8705 PE=2 SV=1
13.	A0A6G5ZVL0_9SAUR	21283	20	Cholinesterase 14 (Fragment) OS=Vipera anatolica senliki OX=2604287 PE=2 SV=1
14.	VKT_MACLN	10904	19	Kunitz-type serine protease inhibitor PIVL OS=Macrovipera lebetina transmediterranea OX=384075 PE=1 SV=1
15.	DIS_MACLB	12004	18	Disintegrin lebestatin OS=Macrovipera lebetina OX=8709 PE=1 SV=1
16.	A0A6G5ZUD1_9SAUR	30200	18	Lysosomal acid lipase/cholesteryl ester hydrolase 2 (Fragment) OS=Vipera anatolica senliki OX=2604287 PE=2 SV=1
17.	V9I1A1_BUNFA	27785	18	Venom nerve growth factor OS=Bungarus fasciatus OX=8613 PE=2 SV=1
18.	M4T472_9PERO	26404	17	Ring finger protein 213 (Fragment) OS=Echiichthys vipera OX=94984 PE=4 SV=1
19.	A0A6B7FPT0_VIPAA	15144	17	Endogenous tripeptide metalloproteinase inhibitor MPi-5 OS=Vipera ammodytes ammodytes OX=8705 PE=2 SV=1
20.	A0A6G5ZW98_9SAUR	25630	17	Serine protease inhibitor 10 (Fragment) OS=Vipera anatolica senliki OX=2604287 PE=2 SV=1

Results List

1. A0A6G5ZW74_9SAUR **Mass:** 32808 **Score:** 53 **Expect:** 0.0066 **Matches:** 10
Veficolin 9 (Fragment) OS=Vipera anatolica senliki OX=2604287 PE=2 SV=1

Observed	Mr(expt)	Mr(calc)	ppm	Start	End	Miss	Ions	Peptide
914.5519	913.5446	913.4869	63.2	255	262	0	---	K.LQDPNSLK.C
1157.6541	1156.6468	1156.6088	32.9	253	262	1	4	K.DKLQDPNSLK.C
1171.6321	1170.6248	1170.5993	21.8	76	87	1	---	K.GEAGKPGEKGNK.G
1185.6366	1184.6293	1184.5574	60.7	153	162	1	---	R.RSDGSVDFFR.D
1199.6461	1198.6388	1198.5917	39.3	243	252	1	---	K.KHSHMPFSTK.D
1330.5539	1329.5466	1329.6136	-50.35	244	254	1	---	K.HSHMPFSTKDK.L + Oxidation (M)
1764.8372	1763.8300	1763.8625	-18.43	55	72	1	---	R.GERGLQGTPGEMGPPGPK.G
1994.0179	1993.0106	1992.8935	58.8	34	54	0	---	R.GCPGMPGSPGPQGPPGFPGAR.G + Oxidation (M)
2134.9820	2133.9747	2133.9837	-4.22	136	153	1	---	R.VLCDMHTDGGGWTVFQRR.S
2150.9758	2149.9685	2149.9786	-4.69	136	153	1	---	R.VLCDMHTDGGGWTVFQRR.S + Oxidation (M)

No match to: 823.5447, 855.0830, 871.0563, 877.0762, 952.4441, 1004.4711, 1011.4796, 1022.1389, 1022.4856, 1044.1219, 1049.4975, 1068.5036, 1079.5006, 1082.0759, 1082.5129, 1094.5136, 1106.4504, 1155.6361, 1156.6134, 1161.6459, 1165.6377, 1173.6479, 1177.6418, 1179.6312, 1183.6566, 1187.6289, 1189.6426, 1193.6359, 1195.5982, 1202.6295, 1203.6124, 1205.6366, 1211.6005, 1218.6238, 1220.4949, 1221.6319, 1227.5952, 1237.6244, 1255.6452, 1277.7454, 1282.6532, 1285.7510, 1291.9938, 1293.5963, 1301.7432, 1305.7277, 1311.7648, 1315.7213, 1317.7360, 1327.7567, 1343.7528, 1344.5305, 1346.5454, 1349.7244, 1350.5366, 1356.5595, 1359.5254, 1362.5398, 1368.5033, 1372.5583, 1378.5329, 1384.4896, 1394.5236, 1475.7859, 1497.7946, 1519.7800, 1535.7454, 1592.7495, 1621.7328, 1625.8878, 1636.7491, 1651.7516, 1658.7257, 1674.6922, 1678.7590, 1698.8238, 1712.6354, 1724.8379, 1740.8289, 2036.9304, 2052.9252, 2074.9097, 2166.9679, 2172.9499, 2682.1857

2. A0A6G5ZVX6_9SAUR **Mass:** 37165 **Score:** 39 **Expect:** 0.16 **Matches:** 8
Veficolin 1 OS=Vipera anatolica senliki OX=2604287 PE=2 SV=1

Observed	Mr(expt)	Mr(calc)	ppm	Start	End	Miss	Ions	Peptide
914.5519	913.5446	913.4869	63.2	267	274	0	---	K.LQDPNSLK.C
1157.6541	1156.6468	1156.6088	32.9	265	274	1	4	K.DKLQDPNSLK.C
1171.6321	1170.6248	1170.5993	21.8	88	99	1	---	K.GEAGKPGEKGNK.G
1199.6461	1198.6388	1198.5917	39.3	255	264	1	---	K.KHSHMPFSTK.D
1211.6005	1210.5933	1210.6095	-13.41	165	174	1	---	R.RSDGLVDFFR.D
1330.5539	1329.5466	1329.6136	-50.35	256	266	1	---	K.HSHMPFSTKDK.L + Oxidation (M)
1764.8372	1763.8300	1763.8625	-18.43	67	84	1	---	R.GERGLQGTPGEMGPPGPK.G
1994.0179	1993.0106	1993.0311	-10.27	4	21	0	---	R.AGKPAALFLLCLMATTCR.G

No match to: 823.5447, 855.0830, 871.0563, 877.0762, 952.4441, 1004.4711, 1011.4796, 1022.1389, 1022.4856, 1044.1219, 1049.4975, 1068.5036, 1079.5006, 1082.0759, 1082.5129, 1094.5136, 1106.4504, 1155.6361, 1156.6134, 1161.6459, 1165.6377, 1173.6479, 1177.6418, 1179.6312, 1183.6566, 1185.6366, 1187.6289, 1189.6426, 1193.6359, 1195.5982, 1202.6295, 1203.6124, 1205.6366, 1218.6238, 1220.4949, 1221.6319, 1227.5952, 1237.6244, 1255.6452, 1277.7454, 1282.6532, 1285.7510, 1291.9938, 1293.5963, 1301.7432, 1305.7277, 1311.7648, 1315.7213, 1317.7360, 1327.7567, 1343.7528, 1344.5305, 1346.5454, 1349.7244, 1350.5366, 1356.5595, 1359.5254, 1362.5398, 1368.5033, 1372.5583, 1378.5329, 1384.4896, 1394.5236, 1475.7859, 1497.7946, 1519.7800, 1535.7454, 1592.7495, 1621.7328, 1625.8878, 1636.7491, 1651.7516, 1658.7257, 1674.6922, 1678.7590, 1698.8238, 1712.6354, 1724.8379, 1740.8289, 2036.9304, 2052.9252, 2074.9097, 2134.9820, 2150.9758, 2166.9679, 2172.9499, 2682.1857

3. A0A6G5ZVY6_9SAUR **Mass:** 24441 **Score:** 34 **Expect:** 0.6 **Matches:** 7
Veficolin 6 (Fragment) OS=Vipera anatolica senliki OX=2604287 PE=2 SV=1

Observed	Mr(expt)	Mr(calc)	ppm	Start	End	Miss	Ions	Peptide
1094.5136	1093.5063	1093.5656	-54.14	214	224	0	---	R.LTVGTFLEGSA.-
1171.6321	1170.6248	1170.5993	21.8	64	75	1	---	K.GEAGKPGEKGNK.G
1211.6005	1210.5933	1210.6095	-13.41	141	150	1	---	R.RSDGLVDFFR.D
1678.7590	1677.7517	1677.8686	-69.65	46	63	1	---	R.GLQGTPGEVGPAGPKGEK.G
1698.8238	1697.8165	1697.7910	15.0	200	213	1	---	K.YGSFRVAAEHDQYR.L
2134.9820	2133.9747	2133.9837	-4.22	124	141	1	---	R.VLCDMHTDGGGWTVFQRR.S
2150.9758	2149.9685	2149.9786	-4.69	124	141	1	---	R.VLCDMHTDGGGWTVFQRR.S + Oxidation (M)

No match to: 823.5447, 855.0830, 871.0563, 877.0762, 914.5519, 952.4441, 1004.4711, 1011.4796, 1022.1389, 1022.4856, 1044.1219, 1049.4975, 1068.5036, 1079.5006, 1082.0759, 1082.5129, 1106.4504, 1155.6361, 1156.6134, 1157.6541, 1161.6459, 1165.6377, 1173.6479, 1177.6418, 1179.6312, 1183.6566, 1185.6366, 1187.6289, 1189.6426, 1193.6359, 1195.5982, 1199.6461, 1202.6295, 1203.6124, 1205.6366, 1218.6238, 1220.4949, 1221.6319, 1227.5952, 1237.6244, 1255.6452, 1277.7454, 1282.6532, 1285.7510, 1291.9938, 1293.5963, 1301.7432, 1305.7277, 1311.7648, 1315.7213, 1317.7360, 1327.7567, 1330.5539, 1343.7528, 1344.5305, 1346.5454, 1349.7244, 1350.5366, 1356.5595, 1359.5254, 1362.5398, 1368.5033, 1372.5583, 1378.5329, 1384.4896, 1394.5236, 1475.7859, 1497.7946, 1519.7800, 1535.7454, 1592.7495, 1621.7328, 1625.8878, 1636.7491, 1651.7516, 1658.7257, 1674.6922, 1712.6354, 1724.8379, 1740.8289, 1764.8372, 1994.0179, 2036.9304, 2052.9252, 2074.9097, 2166.9679, 2172.9499, 2682.1857

4. A0A6G5ZVU3_9SAUR **Mass:** 11896 **Score:** 31 **Expect:** 1.1 **Matches:** 4
Veficolin 4 (Fragment) OS=Vipera anatolica senliki OX=2604287 PE=2 SV=1

Observed	Mr(expt)	Mr(calc)	ppm	Start	End	Miss	Ions	Peptide
914.5519	913.5446	913.4869	63.2	76	83	0	---	K.LQDPNSLK.C
1157.6541	1156.6468	1156.6088	32.9	74	83	1	4	K.DKLQDPNSLK.C
1199.6461	1198.6388	1198.5917	39.3	64	73	1	---	K.KHSHMPFSTK.D
1330.5539	1329.5466	1329.6136	-50.35	65	75	1	---	K.HSHMPFSTKDK.L + Oxidation (M)

No match to: 823.5447, 855.0830, 871.0563, 877.0762, 952.4441, 1004.4711, 1011.4796, 1022.1389, 1022.4856, 1044.1219, 1049.4975, 1068.5036, 1079.5006, 1082.0759, 1082.5129, 1094.5136, 1106.4504, 1155.6361, 1156.6134, 1161.6459, 1165.6377, 1171.6321, 1173.6479, 1177.6418, 1179.6312, 1183.6566, 1185.6366, 1187.6289, 1189.6426, 1193.6359, 1195.5982, 1202.6295, 1203.6124, 1205.6366, 1211.6005, 1218.6238, 1220.4949, 1221.6319, 1227.5952, 1237.6244, 1255.6452, 1277.7454, 1282.6532, 1285.7510, 1291.9938, 1293.5963, 1301.7432, 1305.7277, 1311.7648, 1315.7213, 1317.7360, 1327.7567, 1343.7528, 1344.5305, 1346.5454, 1349.7244, 1350.5366, 1356.5595, 1359.5254, 1362.5398, 1368.5033, 1372.5583, 1378.5329, 1384.4896, 1394.5236, 1475.7859, 1497.7946, 1519.7800, 1535.7454, 1592.7495, 1621.7328, 1625.8878, 1636.7491, 1651.7516, 1658.7257, 1674.6922, 1678.7590, 1698.8238, 1712.6354, 1724.8379, 1740.8289, 1764.8372, 1994.0179, 2036.9304, 2052.9252, 2074.9097, 2134.9820, 2150.9758, 2166.9679, 2172.9499, 2682.1857

5. A0A6G5ZW20_9SAUR **Mass:** 26588 **Score:** 31 **Expect:** 1.1 **Matches:** 6
Veficolin 8 (Fragment) OS=Vipera anatolica senliki OX=2604287 PE=2 SV=1

Observed	Mr(expt)	Mr(calc)	ppm	Start	End	Miss	Ions	Peptide
1094.5136	1093.5063	1093.5656	-54.14	231	241	0	---	R.LTVGTFLEGSA.-
1171.6321	1170.6248	1170.5993	21.8	81	92	1	---	K.GEAGKPGEKGNK.G
1211.6005	1210.5933	1210.6095	-13.41	158	167	1	---	R.RSDGLVDFFR.D
1698.8238	1697.8165	1697.7910	15.0	217	230	1	---	K.YGSFRVAAEHDQYR.L
1764.8372	1763.8300	1763.8625	-18.43	60	77	1	---	R.GERGLQGTPGEMGPPGPK.G
1994.0179	1993.0106	1992.8935	58.8	39	59	0	---	R.GCPGMPGSPGPQGPPGFPGAR.G + Oxidation (M)

No match to: 823.5447, 855.0830, 871.0563, 877.0762, 914.5519, 952.4441, 1004.4711, 1011.4796, 1022.1389, 1022.4856, 1044.1219, 1049.4975, 1068.5036, 1079.5006, 1082.0759, 1082.5129, 1106.4504, 1155.6361, 1156.6134, 1157.6541, 1161.6459, 1165.6377, 1173.6479, 1177.6418, 1179.6312, 1183.6566, 1185.6366, 1187.6289, 1189.6426, 1193.6359, 1195.5982, 1199.6461, 1202.6295, 1203.6124, 1205.6366, 1218.6238, 1220.4949, 1221.6319, 1227.5952, 1237.6244, 1255.6452, 1277.7454, 1282.6532, 1285.7510, 1291.9938, 1293.5963, 1301.7432, 1305.7277, 1311.7648, 1315.7213, 1317.7360, 1327.7567, 1330.5539, 1343.7528, 1344.5305, 1346.5454, 1349.7244, 1350.5366, 1356.5595, 1359.5254, 1362.5398, 1368.5033, 1372.5583, 1378.5329, 1384.4896, 1394.5236, 1475.7859, 1497.7946, 1519.7800, 1535.7454, 1592.7495, 1621.7328, 1625.8878, 1636.7491, 1651.7516, 1658.7257, 1674.6922, 1678.7590, 1712.6354, 1724.8379, 1740.8289, 2036.9304, 2052.9252, 2074.9097, 2134.9820, 2150.9758, 2166.9679, 2172.9499, 2682.1857

6. A0A6G5ZUK5_9SAUR **Mass:** 26961 **Score:** 31 **Expect:** 1.2 **Matches:** 7
Veficolin 5 (Fragment) OS=Vipera anatolica senliki OX=2604287 PE=2 SV=1

Observed	Mr(expt)	Mr(calc)	ppm	Start	End	Miss	Ions	Peptide
1094.5136	1093.5063	1093.5656	-54.14	237	247	0	---	R.LTVGTFLEGSA.-
1171.6321	1170.6248	1170.5993	21.8	87	98	1	---	K.GEAGKPGEKGNK.G

1211.6005	1210.5933	1210.6095	-13.41	164 - 173	1	---	R.RSDGLVDFFR.D
1678.7590	1677.7517	1677.8686	-69.65	69 - 86	1	---	R.GLQGTPGEVGPAGPKGEK.G
1698.8238	1697.8165	1697.7910	15.0	223 - 236	1	---	K.YGSFRVAAEHDQYR.L
2134.9820	2133.9747	2133.9837	-4.22	147 - 164	1	---	R.VLCDMHTDGGGWTVFQRR.S
2150.9758	2149.9685	2149.9786	-4.69	147 - 164	1	---	R.VLCDMHTDGGGWTVFQRR.S + Oxidation (M)

No match to: 823.5447, 855.0830, 871.0563, 877.0762, 914.5519, 952.4441, 1004.4711, 1011.4796, 1022.1389, 1022.4856, 1044.1219, 1049.4975, 1068.5036, 1079.5006, 1082.0759, 1082.5129, 1106.4504, 1155.6361, 1156.6134, 1157.6541, 1161.6459, 1165.6377, 1173.6479, 1177.6418, 1179.6312, 1183.6566, 1185.6366, 1187.6289, 1189.6426, 1193.6359, 1195.5982, 1199.6461, 1202.6295, 1203.6124, 1205.6366, 1218.6238, 1220.4949, 1221.6319, 1227.5952, 1237.6244, 1255.6452, 1277.7454, 1282.6532, 1285.7510, 1291.9938, 1293.5963, 1301.7432, 1305.7277, 1311.7648, 1315.7213, 1317.7360, 1327.7567, 1330.5539, 1343.7528, 1344.5305, 1346.5454, 1349.7244, 1350.5366, 1356.5595, 1359.5254, 1362.5398, 1368.5033, 1372.5583, 1378.5329, 1384.4896, 1394.5236, 1475.7859, 1497.7946, 1519.7800, 1535.7454, 1592.7495, 1621.7328, 1625.8878, 1636.7491, 1651.7516, 1658.7257, 1674.6922, 1712.6354, 1724.8379, 1740.8289, 1764.8372, 1994.0179, 2036.9304, 2052.9252, 2074.9097, 2166.9679, 2172.9499, 2682.1857

7. Q8UVG8_DABRR **Mass:** 52931 **Score:** 28 **Expect:** 2.3 **Matches:** 9

Vimentin OS=Daboia russelii OX=8707 PE=2 SV=1

Observed	Mr(expt)	Mr(calc)	ppm	Start - End	Miss	Ions	Peptide
1155.6361	1154.6288	1154.6910	-53.88	125 - 134	0	---	K.ILVAELEQLK.G
1161.6459	1160.6386	1160.6302	7.24	116 - 124	1	---	K.VRFLEQQNK.I
1173.6479	1172.6406	1172.7129	-61.62	435 - 444	1	---	R.TLLIKTVETR.D
1220.4949	1219.4877	1219.5317	-36.09	5 - 16	0	---	R.TATSSSSSSYR.R
1311.7648	1310.7575	1310.6540	79.0	386 - 396	0	---	K.MALDIEIATYR.K + Oxidation (M)
1327.7567	1326.7494	1326.7255	18.0	171 - 181	1	---	R.DNLAEDILRLR.E
1625.8878	1624.8805	1624.8065	45.5	49 - 63	1	---	R.LVSCPGGIYASKAMR.V + Oxidation (M)
1764.8372	1763.8300	1763.8182	6.69	360 - 373	1	---	R.LQEEIQNMKEEMAR.H + Oxidation (M)
2172.9499	2171.9426	2171.9429	-0.15	341 - 359	0	---	R.EMEEAFGVEAANYQDNIAR.L + Oxidation (M)

No match to: 823.5447, 855.0830, 871.0563, 877.0762, 914.5519, 952.4441, 1004.4711, 1011.4796, 1022.1389, 1022.4856, 1044.1219, 1049.4975, 1068.5036, 1079.5006, 1082.0759, 1082.5129, 1094.5136, 1106.4504, 1156.6134, 1157.6541, 1165.6377, 1171.6321, 1177.6418, 1179.6312, 1183.6566, 1185.6366, 1187.6289, 1189.6426, 1193.6359, 1195.5982, 1199.6461, 1202.6295, 1203.6124, 1205.6366, 1211.6005, 1218.6238, 1221.6319, 1227.5952, 1237.6244, 1255.6452, 1277.7454, 1282.6532, 1285.7510, 1291.9938, 1293.5963, 1301.7432, 1305.7277, 1315.7213, 1317.7360, 1330.5539, 1343.7528, 1344.5305, 1346.5454, 1349.7244, 1350.5366, 1356.5595, 1359.5254, 1362.5398, 1368.5033, 1372.5583, 1378.5329, 1384.4896, 1394.5236, 1475.7859, 1497.7946, 1519.7800, 1535.7454, 1592.7495, 1621.7328, 1636.7491, 1651.7516, 1658.7257, 1674.6922, 1678.7590, 1698.8238, 1712.6354, 1724.8379, 1740.8289, 1994.0179, 2036.9304, 2052.9252, 2074.9097, 2134.9820, 2150.9758, 2166.9679, 2682.1857

8. DIS_MACLO **Mass:** 4854 **Score:** 27 **Expect:** 2.8 **Matches:** 3

Disintegrin obtustatin OS=Macrovipera lebetina obtusa OX=209528 PE=1 SV=1

Observed	Mr(expt)	Mr(calc)	ppm	Start - End	Miss	Ions	Peptide
1011.4796	1010.4723	1010.3732	98.1	1 - 8	0	---	-.CTTGPCCR.Q
1068.5036	1067.4963	1067.4052	85.3	33 - 41	0	---	K.SCDCPLYPG.-
1161.6459	1160.6386	1160.6012	32.2	12 - 21	0	---	K.LKPAGTTCWK.T

No match to: 823.5447, 855.0830, 871.0563, 877.0762, 914.5519, 952.4441, 1004.4711, 1022.1389, 1022.4856, 1044.1219, 1049.4975, 1079.5006, 1082.0759, 1082.5129, 1094.5136, 1106.4504, 1155.6361, 1156.6134, 1157.6541, 1165.6377, 1171.6321, 1173.6479, 1177.6418, 1179.6312, 1183.6566, 1185.6366, 1187.6289, 1189.6426, 1193.6359, 1195.5982, 1199.6461, 1202.6295, 1203.6124, 1205.6366, 1211.6005, 1218.6238, 1220.4949, 1221.6319, 1227.5952, 1237.6244, 1255.6452, 1277.7454, 1282.6532, 1285.7510, 1291.9938, 1293.5963, 1301.7432, 1305.7277, 1311.7648, 1315.7213, 1317.7360, 1327.7567, 1330.5539, 1343.7528, 1344.5305, 1346.5454, 1349.7244, 1350.5366, 1356.5595, 1359.5254, 1362.5398, 1368.5033, 1372.5583, 1378.5329, 1384.4896, 1394.5236, 1475.7859, 1497.7946, 1519.7800, 1535.7454, 1592.7495, 1621.7328, 1625.8878, 1636.7491, 1651.7516, 1658.7257, 1674.6922, 1678.7590, 1698.8238, 1712.6354, 1724.8379, 1740.8289, 1764.8372, 1994.0179, 2036.9304, 2052.9252, 2074.9097, 2134.9820, 2150.9758, 2166.9679, 2172.9499, 2682.1857

9. A0A6G5ZVP7_9SAUR **Mass:** 59614 **Score:** 26 **Expect:** 3.9 **Matches:** 8

Amine oxidase OS=Vipera anatolica senliki OX=2604287 PE=2 SV=1

Observed	Mr(expt)	Mr(calc)	ppm	Start - End	Miss	Ions	Peptide
952.4441	951.4369	951.4913	-57.20	85 - 92	0	---	K.ELGIETYK.V
1179.6312	1178.6239	1178.6295	-4.75	54 - 63	0	---	R.TFTITNQQVK.Y
1203.6124	1202.6051	1202.6441	-32.40	457 - 467	1	---	R.AAREILQSMGK.I
1221.6319	1220.6246	1220.6149	7.94	209 - 219	0	---	R.IFSITNGGQER.K
1317.7360	1316.7287	1316.6248	78.9	471 - 481	0	---	K.NDIWVTEPESK.E
1349.7244	1348.7171	1348.7099	5.34	209 - 220	1	---	R.IFSITNGGQERK.F
1362.5398	1361.5326	1361.6550	-89.95	161 - 170	1	---	K.QFIDKHCWTK.A
1994.0179	1993.0106	1993.0381	-13.79	64 - 81	1	---	K.YVDVGGSYVGPTQNRILR.L

No match to: 823.5447, 855.0830, 871.0563, 877.0762, 914.5519, 1004.4711, 1011.4796, 1022.1389, 1022.4856, 1044.1219, 1049.4975, 1068.5036, 1079.5006, 1082.0759, 1082.5129, 1094.5136, 1106.4504, 1155.6361, 1156.6134, 1157.6541, 1161.6459, 1165.6377, 1171.6321, 1173.6479, 1177.6418, 1183.6566, 1185.6366, 1187.6289, 1189.6426, 1193.6359, 1195.5982, 1199.6461, 1202.6295, 1205.6366, 1211.6005, 1218.6238, 1220.4949, 1227.5952, 1237.6244, 1255.6452, 1277.7454, 1282.6532, 1285.7510, 1291.9938, 1293.5963, 1301.7432, 1305.7277, 1311.7648, 1315.7213, 1327.7567, 1330.5539, 1343.7528, 1344.5305, 1346.5454, 1350.5366, 1356.5595, 1359.5254, 1368.5033, 1372.5583, 1378.5329, 1384.4896, 1394.5236, 1475.7859, 1497.7946, 1519.7800, 1535.7454, 1592.7495, 1621.7328, 1625.8878, 1636.7491, 1651.7516, 1658.7257, 1674.6922, 1678.7590, 1698.8238, 1712.6354, 1724.8379, 1740.8289, 1764.8372, 2036.9304, 2052.9252, 2074.9097, 2134.9820, 2150.9758, 2166.9679, 2172.9499, 2682.1857

10. A0A6G5ZUK1_9SAUR **Mass:** 15084 **Score:** 23 **Expect:** 6.8 **Matches:** 4

Veficolin 7 (Fragment) OS=Vipera anatolica senliki OX=2604287 PE=2 SV=1

Observed	Mr(expt)	Mr(calc)	ppm	Start - End	Miss	Ions	Peptide
914.5519	913.5446	913.4869	63.2	65 - 72	0	---	K.LQDPNSLK.C
1157.6541	1156.6468	1156.6088	32.9	63 - 72	1	4	K.DKLQDPNSLK.C
1199.6461	1198.6388	1198.5917	39.3	53 - 62	1	---	K.KHSHMPFSTK.D
1330.5539	1329.5466	1329.6136	-50.35	54 - 64	1	---	K.HSHMPFSTKDK.L + Oxidation (M)

No match to: 823.5447, 855.0830, 871.0563, 877.0762, 952.4441, 1004.4711, 1011.4796, 1022.1389, 1022.4856, 1044.1219, 1049.4975, 1068.5036, 1079.5006, 1082.0759, 1082.5129, 1094.5136, 1106.4504, 1155.6361, 1156.6134, 1161.6459, 1165.6377, 1171.6321, 1173.6479, 1177.6418, 1179.6312, 1183.6566, 1185.6366, 1187.6289, 1189.6426, 1193.6359, 1195.5982, 1202.6295, 1203.6124, 1205.6366, 1211.6005, 1218.6238, 1220.4949, 1221.6319, 1227.5952, 1237.6244, 1255.6452, 1277.7454, 1282.6532, 1285.7510, 1291.9938, 1293.5963, 1301.7432, 1305.7277, 1311.7648, 1315.7213, 1317.7360, 1327.7567, 1343.7528, 1344.5305, 1346.5454, 1349.7244, 1350.5366, 1356.5595, 1359.5254, 1362.5398, 1368.5033, 1372.5583, 1378.5329, 1384.4896, 1394.5236, 1475.7859, 1497.7946, 1519.7800, 1535.7454, 1592.7495, 1621.7328, 1625.8878, 1636.7491, 1651.7516, 1658.7257, 1674.6922, 1678.7590, 1698.8238, 1712.6354, 1724.8379, 1740.8289, 1764.8372, 1994.0179, 2036.9304, 2052.9252, 2074.9097, 2134.9820, 2150.9758, 2166.9679, 2172.9499, 2682.1857

11. A0A6G5ZUD5_9SAUR **Mass:** 21508 **Score:** 22 **Expect:** 8.5 **Matches:** 4

Lysosomal acid lipase/cholesteryl ester hydrolase 3 (Fragment) OS=Vipera anatolica senliki OX=2604287 PE=2 SV=1

Observed	Mr(expt)	Mr(calc)	ppm	Start - End	Miss	Ions	Peptide
1049.4975	1048.4902	1048.4504	38.0	169 - 176	0	---	R.DMIQMMHK.V + Oxidation (M)
1157.6541	1156.6468	1156.6604	-11.77	11 - 20	0	3	K.QFLPQNAVIK.W
1195.5982	1194.5909	1194.6509	-50.23	71 - 80	0	---	R.NILHWSQAVK.S
1343.7528	1342.7455	1342.6703	56.0	101 - 111	1	---	K.QPTPPPYKMER.M

No match to: 823.5447, 855.0830, 871.0563, 877.0762, 914.5519, 952.4441, 1004.4711, 1011.4796, 1022.1389, 1022.4856, 1044.1219, 1068.5036, 1079.5006, 1082.0759, 1082.5129, 1094.5136, 1106.4504, 1155.6361, 1156.6134, 1161.6459, 1165.6377, 1171.6321, 1173.6479, 1177.6418, 1179.6312, 1183.6566, 1185.6366, 1187.6289, 1189.6426, 1193.6359, 1199.6461, 1202.6295, 1203.6124, 1205.6366, 1211.6005, 1218.6238, 1220.4949, 1221.6319, 1227.5952, 1237.6244, 1255.6452, 1277.7454, 1282.6532, 1285.7510, 1291.9938, 1293.5963, 1301.7432, 1305.7277, 1311.7648, 1315.7213, 1317.7360, 1327.7567, 1330.5539, 1344.5305, 1346.5454, 1349.7244, 1350.5366, 1356.5595, 1359.5254, 1362.5398, 1368.5033, 1372.5583, 1378.5329, 1384.4896, 1394.5236, 1475.7859, 1497.7946, 1519.7800, 1535.7454, 1592.7495, 1621.7328, 1625.8878, 1636.7491, 1651.7516, 1658.7257, 1674.6922, 1678.7590, 1698.8238, 1712.6354, 1724.8379, 1740.8289, 1764.8372, 1994.0179, 2036.9304, 2052.9252, 2074.9097, 2134.9820, 2150.9758, 2166.9679, 2172.9499, 2682.1857

12. A0A1I9KNP4_VIPAA **Mass:** 20248 **Score:** 21 **Expect:** 11 **Matches:** 4
Endogenous tripeptide metalloproteinase inhibitor OS=Vipera ammodytes ammodytes OX=8705 PE=2 SV=1

Observed	Mr(expt)	Mr(calc)	ppm	Start	End	Miss	Ions	Peptide
1237.6244	1236.6171	1236.5007	94.1	170 -	180	1	---	K.VDDHDKGEEGH.-
1475.7859	1474.7786	1474.7562	15.2	156 -	169	1	---	R.IDRIGSVSGLGCNK.V
1698.8238	1697.8165	1697.8195	-1.75	28 -	41	1	---	K.QKWLGPESPPMEQR.W + Oxidation (M)
1764.8372	1763.8300	1763.8023	15.7	125 -	140	1	---	R.MVHHEQQRGGGGGGWR.D + Oxidation (M)

No match to: 823.5447, 855.0830, 871.0563, 877.0762, 914.5519, 952.4441, 1004.4711, 1011.4796, 1022.1389, 1022.4856, 1044.1219, 1049.4975,
1068.5036, 1079.5006, 1082.0759, 1082.5129, 1094.5136, 1106.4504, 1155.6361, 1156.6134, 1157.6541, 1161.6459, 1165.6377, 1171.6321,
1173.6479, 1177.6418, 1179.6312, 1183.6566, 1185.6366, 1187.6289, 1189.6426, 1193.6359, 1195.5982, 1199.6461, 1202.6295, 1203.6124,
1205.6366, 1211.6005, 1218.6238, 1220.4949, 1221.6319, 1227.5952, 1255.6452, 1277.7454, 1282.6532, 1285.7510, 1291.9938, 1293.5963,
1301.7432, 1305.7277, 1311.7648, 1315.7213, 1317.7360, 1327.7567, 1330.5539, 1343.7528, 1344.5305, 1346.5454, 1349.7244, 1350.5366,
1356.5595, 1359.5254, 1362.5398, 1368.5033, 1372.5583, 1378.5329, 1384.4896, 1394.5236, 1497.7946, 1519.7800, 1535.7454, 1592.7495,
1621.7328, 1625.8878, 1636.7491, 1651.7516, 1658.7257, 1674.6922, 1678.7590, 1712.6354, 1724.8379, 1740.8289, 1994.0179, 2036.9304,
2052.9252, 2074.9097, 2134.9820, 2150.9758, 2166.9679, 2172.9499, 2682.1857

13. A0A6G5ZVL0_9SAUR **Mass:** 21283 **Score:** 20 **Expect:** 16 **Matches:** 4
Cholinesterase 14 (Fragment) OS=Vipera anatolica senliki OX=2604287 PE=2 SV=1

Observed	Mr(expt)	Mr(calc)	ppm	Start	End	Miss	Ions	Peptide
952.4441	951.4369	951.5138	-80.82	37 -	45	0	---	K.EVAHAVAQK.F
1205.6366	1204.6293	1204.5935	29.7	125 -	135	0	---	R.SITEAEEALSR.Q
1519.7800	1518.7727	1518.6860	57.1	136 -	146	1	---	R.QMMRYWAQFAR.D + 2 Oxidation (M)
2682.1857	2681.1785	2681.2692	-33.82	56 -	77	1	---	R.LAFSQYMRDYFMVCPLAELAEK.I

No match to: 823.5447, 855.0830, 871.0563, 877.0762, 914.5519, 1004.4711, 1011.4796, 1022.1389, 1022.4856, 1044.1219, 1049.4975, 1068.5036,
1079.5006, 1082.0759, 1082.5129, 1094.5136, 1106.4504, 1155.6361, 1156.6134, 1157.6541, 1161.6459, 1165.6377, 1171.6321, 1173.6479,
1177.6418, 1179.6312, 1183.6566, 1185.6366, 1187.6289, 1189.6426, 1193.6359, 1195.5982, 1199.6461, 1202.6295, 1203.6124, 1211.6005,
1218.6238, 1220.4949, 1221.6319, 1227.5952, 1237.6244, 1255.6452, 1277.7454, 1282.6532, 1285.7510, 1291.9938, 1293.5963, 1301.7432,
1305.7277, 1311.7648, 1315.7213, 1317.7360, 1327.7567, 1330.5539, 1343.7528, 1344.5305, 1346.5454, 1349.7244, 1350.5366, 1356.5595,
1359.5254, 1362.5398, 1368.5033, 1372.5583, 1378.5329, 1384.4896, 1394.5236, 1475.7859, 1497.7946, 1535.7454, 1592.7495, 1621.7328,
1625.8878, 1636.7491, 1651.7516, 1658.7257, 1674.6922, 1678.7590, 1698.8238, 1712.6354, 1724.8379, 1740.8289, 1764.8372, 1994.0179,
2036.9304, 2052.9252, 2074.9097, 2134.9820, 2150.9758, 2166.9679, 2172.9499

14. VKT_MACLN **Mass:** 10904 **Score:** 19 **Expect:** 18 **Matches:** 3
Kunitz-type serine protease inhibitor PIVL OS=Macrovipera lebetina transmediterranea OX=384075 PE=1 SV=1

Observed	Mr(expt)	Mr(calc)	ppm	Start	End	Miss	Ions	Peptide
1094.5136	1093.5063	1093.4716	31.8	47 -	55	0	---	R.FYYDSASNK.C
1346.5454	1345.5381	1345.5979	-44.46	75 -	85	1	---	R.AECRHTCVASR.K
2074.9097	2073.9024	2073.8747	13.4	30 -	46	0	---	K.FCYLPADPAECNAYMPR.F

No match to: 823.5447, 855.0830, 871.0563, 877.0762, 914.5519, 952.4441, 1004.4711, 1011.4796, 1022.1389, 1022.4856, 1044.1219, 1049.4975,
1068.5036, 1079.5006, 1082.0759, 1082.5129, 1106.4504, 1155.6361, 1156.6134, 1157.6541, 1161.6459, 1165.6377, 1171.6321, 1173.6479,
1177.6418, 1179.6312, 1183.6566, 1185.6366, 1187.6289, 1189.6426, 1193.6359, 1195.5982, 1199.6461, 1202.6295, 1203.6124, 1205.6366,
1211.6005, 1218.6238, 1220.4949, 1221.6319, 1227.5952, 1237.6244, 1255.6452, 1277.7454, 1282.6532, 1285.7510, 1291.9938, 1293.5963,
1301.7432, 1305.7277, 1311.7648, 1315.7213, 1317.7360, 1327.7567, 1330.5539, 1343.7528, 1344.5305, 1349.7244, 1350.5366, 1356.5595,
1359.5254, 1362.5398, 1368.5033, 1372.5583, 1378.5329, 1384.4896, 1394.5236, 1475.7859, 1497.7946, 1519.7800, 1535.7454, 1592.7495,
1621.7328, 1625.8878, 1636.7491, 1651.7516, 1658.7257, 1674.6922, 1678.7590, 1698.8238, 1712.6354, 1724.8379, 1740.8289, 1764.8372,
1994.0179, 2036.9304, 2052.9252, 2134.9820, 2150.9758, 2166.9679, 2172.9499, 2682.1857

15. DIS_MACLB **Mass:** 12004 **Score:** 18 **Expect:** 22 **Matches:** 3
Disintegrin lebestatin OS=Macrovipera lebetina OX=8709 PE=1 SV=1

Observed	Mr(expt)	Mr(calc)	ppm	Start	End	Miss	Ions	Peptide
1161.6459	1160.6386	1160.6012	32.2	76 -	85	0	---	K.LKPAGTTCWK.T
1282.6532	1281.6459	1281.6499	-3.15	51 -	61	1	---	K.HEPMKGNTLQK.F
1368.5033	1367.4960	1367.5785	-60.28	62 -	72	0	---	K.FPLCTTGPCCR.Q

No match to: 823.5447, 855.0830, 871.0563, 877.0762, 914.5519, 952.4441, 1004.4711, 1011.4796, 1022.1389, 1022.4856, 1044.1219, 1049.4975,
1068.5036, 1079.5006, 1082.0759, 1082.5129, 1094.5136, 1106.4504, 1155.6361, 1156.6134, 1157.6541, 1165.6377, 1171.6321, 1173.6479,
1177.6418, 1179.6312, 1183.6566, 1185.6366, 1187.6289, 1189.6426, 1193.6359, 1195.5982, 1199.6461, 1202.6295, 1203.6124, 1205.6366,
1211.6005, 1218.6238, 1220.4949, 1221.6319, 1227.5952, 1237.6244, 1255.6452, 1277.7454, 1285.7510, 1291.9938, 1293.5963, 1301.7432,
1305.7277, 1311.7648, 1315.7213, 1317.7360, 1327.7567, 1330.5539, 1343.7528, 1344.5305, 1346.5454, 1349.7244, 1350.5366, 1356.5595,
1359.5254, 1362.5398, 1372.5583, 1378.5329, 1384.4896, 1394.5236, 1475.7859, 1497.7946, 1519.7800, 1535.7454, 1592.7495, 1621.7328,
1625.8878, 1636.7491, 1651.7516, 1658.7257, 1674.6922, 1678.7590, 1698.8238, 1712.6354, 1724.8379, 1740.8289, 1764.8372, 1994.0179,
2036.9304, 2052.9252, 2074.9097, 2134.9820, 2150.9758, 2166.9679, 2172.9499, 2682.1857

16. A0A6G5ZUD1_9SAUR **Mass:** 30200 **Score:** 18 **Expect:** 25 **Matches:** 4
Lysosomal acid lipase/cholesteryl ester hydrolase 2 (Fragment) OS=Vipera anatolica senliki OX=2604287 PE=2 SV=1

Observed	Mr(expt)	Mr(calc)	ppm	Start	End	Miss	Ions	Peptide
1049.4975	1048.4902	1048.4504	38.0	249 -	256	0	---	R.DMIQMMHK.V + Oxidation (M)
1157.6541	1156.6468	1156.6604	-11.77	91 -	100	0	3	K.QFLPQNAVIK.W
1195.5982	1194.5909	1194.6509	-50.23	151 -	160	0	---	R.NILHWSQAVK.S
1343.7528	1342.7455	1342.6703	56.0	181 -	191	1	---	K.QPTPPPYKMER.M

No match to: 823.5447, 855.0830, 871.0563, 877.0762, 914.5519, 952.4441, 1004.4711, 1011.4796, 1022.1389, 1022.4856, 1044.1219, 1068.5036,
1079.5006, 1082.0759, 1082.5129, 1094.5136, 1106.4504, 1155.6361, 1156.6134, 1161.6459, 1165.6377, 1171.6321, 1173.6479, 1177.6418,
1179.6312, 1183.6566, 1185.6366, 1187.6289, 1189.6426, 1193.6359, 1199.6461, 1202.6295, 1203.6124, 1205.6366, 1211.6005, 1218.6238,
1220.4949, 1221.6319, 1227.5952, 1237.6244, 1255.6452, 1277.7454, 1282.6532, 1285.7510, 1291.9938, 1293.5963, 1301.7432, 1305.7277,
1311.7648, 1315.7213, 1317.7360, 1327.7567, 1330.5539, 1344.5305, 1346.5454, 1349.7244, 1350.5366, 1356.5595, 1359.5254, 1362.5398,
1368.5033, 1372.5583, 1378.5329, 1384.4896, 1394.5236, 1475.7859, 1497.7946, 1519.7800, 1535.7454, 1592.7495, 1621.7328, 1625.8878,
1636.7491, 1651.7516, 1658.7257, 1674.6922, 1678.7590, 1698.8238, 1712.6354, 1724.8379, 1740.8289, 1764.8372, 1994.0179, 2036.9304,
2052.9252, 2074.9097, 2134.9820, 2150.9758, 2166.9679, 2172.9499, 2682.1857

17. V9I1A1_BUNFA **Mass:** 27785 **Score:** 18 **Expect:** 25 **Matches:** 4
Venom nerve growth factor OS=Bungarus fasciatus OX=8613 PE=2 SV=1

Observed	Mr(expt)	Mr(calc)	ppm	Start	End	Miss	Ions	Peptide
1293.5963	1292.5890	1292.6217	-25.27	228 -	238	0	---	R.IDTACVCVISR.K
1497.7946	1496.7873	1496.7293	38.8	208 -	220	1	---	R.DTFVKALTMEGNR.A + Oxidation (M)
1625.8878	1624.8805	1624.7740	65.6	184 -	198	1	---	R.NPNPVPSGCRGIDSR.H
2074.9097	2073.9024	2074.0960	-93.32	86 -	103	1	---	R.QFQSPRVLFSTQSPPLSR.D

No match to: 823.5447, 855.0830, 871.0563, 877.0762, 914.5519, 952.4441, 1004.4711, 1011.4796, 1022.1389, 1022.4856, 1044.1219, 1049.4975,
1068.5036, 1079.5006, 1082.0759, 1082.5129, 1094.5136, 1106.4504, 1155.6361, 1156.6134, 1157.6541, 1161.6459, 1165.6377, 1171.6321,
1173.6479, 1177.6418, 1179.6312, 1183.6566, 1185.6366, 1187.6289, 1189.6426, 1193.6359, 1195.5982, 1199.6461, 1202.6295, 1203.6124,
1205.6366, 1211.6005, 1218.6238, 1220.4949, 1221.6319, 1227.5952, 1237.6244, 1255.6452, 1277.7454, 1282.6532, 1285.7510, 1291.9938,
1301.7432, 1305.7277, 1311.7648, 1315.7213, 1317.7360, 1327.7567, 1330.5539, 1343.7528, 1344.5305, 1346.5454, 1349.7244, 1350.5366,
1356.5595, 1359.5254, 1362.5398, 1368.5033, 1372.5583, 1378.5329, 1384.4896, 1394.5236, 1475.7859, 1519.7800, 1535.7454, 1592.7495,
1621.7328, 1636.7491, 1651.7516, 1658.7257, 1674.6922, 1678.7590, 1698.8238, 1712.6354, 1724.8379, 1740.8289, 1764.8372, 1994.0179,
2036.9304, 2052.9252, 2134.9820, 2150.9758, 2166.9679, 2172.9499, 2682.1857

18. M4T472_9PERO **Mass:** 26404 **Score:** 17 **Expect:** 26 **Matches:** 5
Ring finger protein 213 (Fragment) OS=Echiichthys vipera OX=94984 PE=4 SV=1

Observed	Mr(expt)	Mr(calc)	ppm	Start	End	Miss	Ions	Peptide
1094.5136	1093.5063	1093.5186	-11.19	129 -	138	1	---	R.RSGVASENMK.L + Oxidation (M)
1157.6541	1156.6468	1156.5659	70.0	220 -	228	1	---	R.KHTDEMIQR.L
1173.6479	1172.6406	1172.5608	68.1	220 -	228	1	---	R.KHTDEMIQR.L + Oxidation (M)
1678.7590	1677.7517	1677.8508	-59.06	142 -	156	1	---	K.VHGGTTSEMIYTKVR.E
1764.8372	1763.8300	1763.9352	-59.66	35 -	49	1	---	R.NVMTRALYEGLTLQR.V

No match to: 823.5447, 855.0830, 871.0563, 877.0762, 914.5519, 952.4441, 1004.4711, 1011.4796, 1022.1389, 1022.4856, 1044.1219, 1049.4975, 1068.5036, 1079.5006, 1082.0759, 1082.5129, 1106.4504, 1155.6361, 1156.6134, 1161.6459, 1165.6377, 1171.6321, 1177.6418, 1179.6312, 1183.6566, 1185.6366, 1187.6289, 1189.6426, 1193.6359, 1195.5982, 1199.6461, 1202.6295, 1203.6124, 1205.6366, 1211.6005, 1218.6238, 1220.4949, 1221.6319, 1227.5952, 1237.6244, 1255.6452, 1277.7454, 1282.6532, 1285.7510, 1291.9938, 1293.5963, 1301.7432, 1305.7277, 1311.7648, 1315.7213, 1317.7360, 1327.7567, 1330.5539, 1343.7528, 1344.5305, 1346.5454, 1349.7244, 1350.5366, 1356.5595, 1359.5254, 1362.5398, 1368.5033, 1372.5583, 1378.5329, 1384.4896, 1394.5236, 1475.7859, 1497.7946, 1519.7800, 1535.7454, 1592.7495, 1621.7328, 1625.8878, 1636.7491, 1651.7516, 1658.7257, 1674.6922, 1698.8238, 1712.6354, 1724.8379, 1740.8289, 1994.0179, 2036.9304, 2052.9252, 2074.9097, 2134.9820, 2150.9758, 2166.9679, 2172.9499, 2682.1857

19. A0A6B7FPT0_VIPAA **Mass:** 15144 **Score:** 17 **Expect:** 27 **Matches:** 3

Endogenous tripeptide metalloproteinase inhibitor MPi-5 OS=Vipera ammodytes ammodytes OX=8705 PE=2 SV=1

Observed	Mr(expt)	Mr(calc)	ppm	Start	End	Miss	Ions	Peptide
1475.7859	1474.7786	1474.7562	15.2	113 -	126	1	---	R.IDRIGSVSGLGCNK.V
1698.8238	1697.8165	1697.8195	-1.75	28 -	41	1	---	K.QKWLGPESPPMEQR.W + Oxidation (M)
1764.8372	1763.8300	1763.8023	15.7	82 -	97	1	---	R.MVHHEQQRGGGGGGWR.D + Oxidation (M)

No match to: 823.5447, 855.0830, 871.0563, 877.0762, 914.5519, 952.4441, 1004.4711, 1011.4796, 1022.1389, 1022.4856, 1044.1219, 1049.4975, 1068.5036, 1079.5006, 1082.0759, 1082.5129, 1094.5136, 1106.4504, 1155.6361, 1156.6134, 1157.6541, 1161.6459, 1165.6377, 1171.6321, 1173.6479, 1177.6418, 1179.6312, 1183.6566, 1185.6366, 1187.6289, 1189.6426, 1193.6359, 1195.5982, 1199.6461, 1202.6295, 1203.6124, 1205.6366, 1211.6005, 1218.6238, 1220.4949, 1221.6319, 1227.5952, 1237.6244, 1255.6452, 1277.7454, 1282.6532, 1285.7510, 1291.9938, 1293.5963, 1301.7432, 1305.7277, 1311.7648, 1315.7213, 1317.7360, 1327.7567, 1330.5539, 1343.7528, 1344.5305, 1346.5454, 1349.7244, 1350.5366, 1356.5595, 1359.5254, 1362.5398, 1368.5033, 1372.5583, 1378.5329, 1384.4896, 1394.5236, 1497.7946, 1519.7800, 1535.7454, 1592.7495, 1621.7328, 1625.8878, 1636.7491, 1651.7516, 1658.7257, 1674.6922, 1678.7590, 1712.6354, 1724.8379, 1740.8289, 1994.0179, 2036.9304, 2052.9252, 2074.9097, 2134.9820, 2150.9758, 2166.9679, 2172.9499, 2682.1857

20. A0A6G5ZW98_9SAUR **Mass:** 25630 **Score:** 17 **Expect:** 30 **Matches:** 4

Serine protease inhibitor 10 (Fragment) OS=Vipera anatolica senliki OX=2604287 PE=2 SV=1

Observed	Mr(expt)	Mr(calc)	ppm	Start	End	Miss	Ions	Peptide
1220.4949	1219.4877	1219.5431	-45.43	214 -	223	0	---	K.EYLMSNAYTL.- + Oxidation (M)
1285.7510	1284.7437	1284.6510	72.2	100 -	110	1	---	R.VVGPCRASFHR.W
1317.7360	1316.7287	1316.6230	80.2	9 -	19	1	---	K.MVGPCRASFHR.W
1344.5305	1343.5232	1343.5897	-49.45	4 -	14	1	---	R.CHSPKMVGPCR.A + Oxidation (M)

No match to: 823.5447, 855.0830, 871.0563, 877.0762, 914.5519, 952.4441, 1004.4711, 1011.4796, 1022.1389, 1022.4856, 1044.1219, 1049.4975, 1068.5036, 1079.5006, 1082.0759, 1082.5129, 1094.5136, 1106.4504, 1155.6361, 1156.6134, 1157.6541, 1161.6459, 1165.6377, 1171.6321, 1173.6479, 1177.6418, 1179.6312, 1183.6566, 1185.6366, 1187.6289, 1189.6426, 1193.6359, 1195.5982, 1199.6461, 1202.6295, 1203.6124, 1205.6366, 1211.6005, 1218.6238, 1221.6319, 1227.5952, 1237.6244, 1255.6452, 1277.7454, 1282.6532, 1291.9938, 1293.5963, 1301.7432, 1305.7277, 1311.7648, 1315.7213, 1327.7567, 1330.5539, 1343.7528, 1346.5454, 1349.7244, 1350.5366, 1356.5595, 1359.5254, 1362.5398, 1368.5033, 1372.5583, 1378.5329, 1384.4896, 1394.5236, 1475.7859, 1497.7946, 1519.7800, 1535.7454, 1592.7495, 1621.7328, 1625.8878, 1636.7491, 1651.7516, 1658.7257, 1674.6922, 1678.7590, 1698.8238, 1712.6354, 1724.8379, 1740.8289, 1764.8372, 1994.0179, 2036.9304, 2052.9252, 2074.9097, 2134.9820, 2150.9758, 2166.9679, 2172.9499, 2682.1857

Search Parameters

Type of search : MS/MS Ion Search
Enzyme : Trypsin
Fixed modifications : Carbamidomethyl (C)
Variable modifications : Oxidation (M)
Mass values : Monoisotopic
Protein Mass : Unrestricted
Peptide Mass Tolerance : ± 100 ppm
Fragment Mass Tolerance: ± 0.7 Da
Max Missed Cleavages : 1
Instrument type : MALDI-TOF-TOF
Query1 (823.5447,1+) : <no title>
Query2 (855.0830,1+) : <no title>
Query3 (871.0563,1+) : <no title>
Query4 (877.0762,1+) : <no title>
Query5 (914.5519,1+) : \\PROTEOMICS1\D\DATA\2105\210505_TLIEPOL_1_68252-68266\68252_68266_LPARDO\0_H8\1\914.5519.LIFT\1SREF\PDATA\1\1R
Query6 (952.4441,1+) : <no title>
Query7 (1004.4711,1+) : <no title>
Query8 (1011.4796,1+) : <no title>
Query9 (1022.1389,1+) : <no title>
Query10 (1022.4856,1+) : <no title>
Query11 (1044.1219,1+) : <no title>
Query12 (1049.4975,1+) : <no title>
Query13 (1068.5036,1+) : <no title>
Query14 (1079.5006,1+) : <no title>
Query15 (1082.0759,1+) : <no title>
Query16 (1082.5129,1+) : <no title>
Query17 (1094.5136,1+) : <no title>
Query18 (1106.4504,1+) : <no title>
Query19 (1155.6361,1+) : <no title>
Query20 (1156.6134,1+) : <no title>
Query21 (1157.6541,1+) : \\PROTEOMICS1\D\DATA\2105\210505_TLIEPOL_1_68252-68266\68252_68266_LPARDO\0_H8\1\1157.6541.LIFT\1SREF\PDATA\1\1R
Query22 (1161.6459,1+) : <no title>
Query23 (1165.6377,1+) : <no title>
Query24 (1171.6321,1+) : \\PROTEOMICS1\D\DATA\2105\210505_TLIEPOL_1_68252-68266\68252_68266_LPARDO\0_H8\1\1171.6321.LIFT\1SREF\PDATA\1\1R
Query25 (1173.6479,1+) : <no title>
Query26 (1177.6418,1+) : <no title>
Query27 (1179.6312,1+) : <no title>
Query28 (1183.6566,1+) : <no title>
Query29 (1185.6366,1+) : <no title>
Query30 (1187.6289,1+) : <no title>
Query31 (1189.6426,1+) : \\PROTEOMICS1\D\DATA\2105\210505_TLIEPOL_1_68252-68266\68252_68266_LPARDO\0_H8\1\1189.6426.LIFT\1SREF\PDATA\1\1R
Query32 (1193.6359,1+) : <no title>
Query33 (1195.5982,1+) : <no title>
Query34 (1199.6461,1+) : <no title>
Query35 (1202.6295,1+) : <no title>
Query36 (1203.6124,1+) : <no title>
Query37 (1205.6366,1+) : <no title>
Query38 (1211.6005,1+) : <no title>
Query39 (1218.6238,1+) : <no title>
Query40 (1220.4949,1+) : <no title>
Query41 (1221.6319,1+) : <no title>
Query42 (1227.5952,1+) : <no title>
Query43 (1237.6244,1+) : <no title>
Query44 (1255.6452,1+) : <no title>
Query45 (1277.7454,1+) : <no title>
Query46 (1282.6532,1+) : <no title>
Query47 (1285.7510,1+) : <no title>
Query48 (1291.9938,1+) : <no title>
Query49 (1293.5963,1+) : <no title>
Query50 (1301.7432,1+) : <no title>
Query51 (1305.7277,1+) : <no title>
Query52 (1311.7648,1+) : <no title>
Query53 (1315.7213,1+) : <no title>
Query54 (1317.7360,1+) : <no title>
Query55 (1327.7567,1+) : <no title>
Query56 (1330.5539,1+) : \\PROTEOMICS1\D\DATA\2105\210505_TLIEPOL_1_68252-68266\68252_68266_LPARDO\0_H8\1\1330.5539.LIFT\1SREF\PDATA\1\1R
Query57 (1343.7528,1+) : <no title>
Query58 (1344.5305,1+) : <no title>

Query59 (1346.5454,1+) : \\PROTEOMICS1\D\DATA\2105\210505_TLIEPOL_1_68252-68266\68252_68266_LPARDO\0_H8\1\1346.5454.LIFT\1SREF\PDATA\1\1R
Query60 (1349.7244,1+) : <no title>
Query61 (1350.5366,1+) : <no title>
Query62 (1356.5595,1+) : <no title>
Query63 (1359.5254,1+) : <no title>
Query64 (1362.5398,1+) : <no title>
Query65 (1368.5033,1+) : <no title>
Query66 (1372.5583,1+) : <no title>
Query67 (1378.5329,1+) : <no title>
Query68 (1384.4896,1+) : <no title>
Query69 (1394.5236,1+) : <no title>
Query70 (1475.7859,1+) : <no title>
Query71 (1497.7946,1+) : <no title>
Query72 (1519.7800,1+) : <no title>
Query73 (1535.7454,1+) : <no title>
Query74 (1592.7495,1+) : <no title>
Query75 (1621.7328,1+) : <no title>
Query76 (1625.8878,1+) : <no title>
Query77 (1636.7491,1+) : \\PROTEOMICS1\D\DATA\2105\210505_TLIEPOL_1_68252-68266\68252_68266_LPARDO\0_H8\1\1636.7491.LIFT\1SREF\PDATA\1\1R
Query78 (1651.7516,1+) : <no title>
Query79 (1658.7257,1+) : <no title>
Query80 (1674.6922,1+) : <no title>
Query81 (1678.7590,1+) : <no title>
Query82 (1698.8238,1+) : \\PROTEOMICS1\D\DATA\2105\210505_TLIEPOL_1_68252-68266\68252_68266_LPARDO\0_H8\1\1698.8238.LIFT\1SREF\PDATA\1\1R
Query83 (1712.6354,1+) : <no title>
Query84 (1724.8379,1+) : <no title>
Query85 (1740.8289,1+) : <no title>
Query86 (1764.8372,1+) : \\PROTEOMICS1\D\DATA\2105\210505_TLIEPOL_1_68252-68266\68252_68266_LPARDO\0_H8\1\1764.8372.LIFT\1SREF\PDATA\1\1R
Query87 (1994.0179,1+) : \\PROTEOMICS1\D\DATA\2105\210505_TLIEPOL_1_68252-68266\68252_68266_LPARDO\0_H8\1\1994.0179.LIFT\1SREF\PDATA\1\1R
Query88 (2036.9304,1+) : <no title>
Query89 (2052.9252,1+) : <no title>
Query90 (2074.9097,1+) : <no title>
Query91 (2134.9820,1+) : \\PROTEOMICS1\D\DATA\2105\210505_TLIEPOL_1_68252-68266\68252_68266_LPARDO\0_H8\1\2134.9820.LIFT\1SREF\PDATA\1\1R
Query92 (2150.9758,1+) : \\PROTEOMICS1\D\DATA\2105\210505_TLIEPOL_1_68252-68266\68252_68266_LPARDO\0_H8\1\2150.9758.LIFT\1SREF\PDATA\1\1R
Query93 (2166.9679,1+) : <no title>
Query94 (2172.9499,1+) : <no title>
Query95 (2682.1857,1+) : <no title>

Mascot: http://www.matrixscience.com/

Mascot Search Results Fr5-2

```
User         :
Email        :
Search title : vipera_All_8IDplus4 file:\\Proteomics1\d\data\2105\210505_tliepol_1_68252-68266\68252_68266_lpardo\0_G9\1\1SRef\pdata\1\peaklist.xml
Database     : vipera  (1414 sequences; 347452 residues)
Timestamp    : 5 May 2021 at 09:36:20 GMT
Top Score    : 71 for PA2A1_MACLB, Acidic phospholipase A2 1 OS=Macrovipera lebetina OX=8709 PE=1 SV=1
```

Mascot Score Histogram

Protein score is -10*Log(P), where P is the probability that the observed match is a random event.
Protein scores greater than 44 are significant (p<0.05).

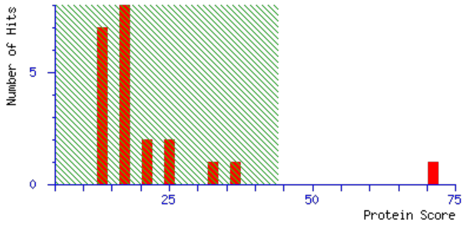

Concise Protein Summary Report

Format As | Concise Protein Summary | Help

Significance threshold p< 0.05 Max. number of hits 10

Re-Search All | Search Unmatched

1. PA2A1_MACLB **Mass:** 16337 **Score:** 71 **Expect:** 0.00011 **Matches:** 8
Acidic phospholipase A2 1 OS=Macrovipera lebetina OX=8709 PE=1 SV=1
PA2A2_MACLB **Mass:** 16247 **Score:** 58 **Expect:** 0.0024 **Matches:** 7
Acidic phospholipase A2 2 OS=Macrovipera lebetina OX=8709 PE=1 SV=1
PA2A_MACLN **Mass:** 16243 **Score:** 33 **Expect:** 0.69 **Matches:** 4
Acidic phospholipase A2 MVL-PLA2 OS=Macrovipera lebetina transmediterranea OX=384075 PE=1 SV=1
PA2A_DABPA **Mass:** 16277 **Score:** 33 **Expect:** 0.69 **Matches:** 4
Acidic phospholipase A2 VpaPLA2 OS=Daboia palaestinae OX=1170828 PE=1 SV=1
Q6A3G8_VIPAA **Mass:** 15517 **Score:** 22 **Expect:** 9.3 **Matches:** 4
Ammodytin I1(A) variant OS=Vipera ammodytes ammodytes OX=8705 PE=2 SV=1
Q6A3G4_VIPAM **Mass:** 16280 **Score:** 21 **Expect:** 10 **Matches:** 4
Ammodytin I1(D) isoform OS=Vipera ammodytes montandoni OX=235554 PE=2 SV=1
Q6A3N0_VIPAM **Mass:** 16279 **Score:** 21 **Expect:** 10 **Matches:** 4
Ammodytin I1(A) isoform OS=Vipera ammodytes ruffoi OX=246549 PE=2 SV=1
Q6A3G5_VIPAE **Mass:** 16280 **Score:** 21 **Expect:** 10 **Matches:** 4
Ammodytin I1(D) isoform OS=Vipera ammodytes meridionalis OX=73841 PE=2 SV=1
Q6YC90_VIPAS **Mass:** 16279 **Score:** 21 **Expect:** 10 **Matches:** 4
Ammodytin I1 isoform 2 OS=Vipera aspis OX=8706 GN=AmtI1 PE=3 SV=1
Q6A3K8_VIPAP **Mass:** 16267 **Score:** 21 **Expect:** 10 **Matches:** 4
Ammodytin I1(A') isoform OS=Vipera aspis aspis OX=194601 PE=2 SV=1
Q6A3K4_VIPAP **Mass:** 16252 **Score:** 21 **Expect:** 10 **Matches:** 4
Ammodytin I1(A) variant OS=Vipera aspis aspis OX=194601 PE=2 SV=1
Q6A3K7_VIPAP **Mass:** 16279 **Score:** 21 **Expect:** 10 **Matches:** 4
Ammodytin I1(A) isoform OS=Vipera aspis aspis OX=194601 PE=2 SV=1
Q6A3H1_VIPAS **Mass:** 16292 **Score:** 21 **Expect:** 10 **Matches:** 4
Ammodytin I1(C) variant OS=Vipera aspis atra OX=246182 PE=2 SV=1
PA2A1_VIPAA **Mass:** 16279 **Score:** 21 **Expect:** 10 **Matches:** 4
Acidic phospholipase A2 ammodytin I1 OS=Vipera ammodytes ammodytes OX=8705 PE=2 SV=1
Q7T1D1_VIPBB **Mass:** 16258 **Score:** 21 **Expect:** 11 **Matches:** 4
Ammodytin I1 OS=Vipera berus berus OX=31156 GN=AmtI1 PE=2 SV=1
Q6A3K0_VIPBB **Mass:** 16214 **Score:** 21 **Expect:** 11 **Matches:** 4
Ammodytin I1(B') variant OS=Vipera berus berus OX=31156 PE=2 SV=1
Q1RP80_VIPBN **Mass:** 16258 **Score:** 21 **Expect:** 11 **Matches:** 4
Phospholipase A2 ammodytin I1 OS=Vipera berus nikolskii OX=1808362 GN=I1 PE=2 SV=1
Q6A3M8_VIPAM **Mass:** 16378 **Score:** 21 **Expect:** 11 **Matches:** 4
Ammodytin I1(A) variant OS=Vipera ammodytes ruffoi OX=246549 PE=2 SV=1
PA2A2_DABRR **Mass:** 16430 **Score:** 21 **Expect:** 12 **Matches:** 4
Acidic phospholipase A2 Drk-a2 OS=Daboia russelii OX=8707 PE=2 SV=1
Q6A3I8_VIPUR **Mass:** 16290 **Score:** 21 **Expect:** 12 **Matches:** 4
Ammodytin I1(B) isoform OS=Vipera ursinii OX=103942 PE=2 SV=1
Q6A3M9_VIPAM **Mass:** 16286 **Score:** 19 **Expect:** 17 **Matches:** 4
Ammodytin I1(B) isoform OS=Vipera ammodytes ruffoi OX=246549 PE=2 SV=1

---

2. PA2A7_DABPA **Mass:** 16190 **Score:** 35 **Expect:** 0.49 **Matches:** 4
Acidic phospholipase A2 VP7 OS=Daboia palaestinae OX=1170828 PE=3 SV=1
PA2A8_DABPA **Mass:** 16194 **Score:** 23 **Expect:** 7.8 **Matches:** 3
Acidic phospholipase A2 VP8 OS=Daboia palaestinae OX=1170828 PE=3 SV=1

---

3. PA2B_DABRR **Mass:** 14415 **Score:** 33 **Expect:** 0.65 **Matches:** 5
Basic phospholipase A2 RVV-VD OS=Daboia russelii OX=8707 PE=1 SV=1
PA2N_DABRR **Mass:** 2192 **Score:** 22 **Expect:** 8.1 **Matches:** 3
Neutral phospholipase A2 RVV-PFIIc' (Fragment) OS=Daboia russelii OX=8707 PE=1 SV=1

---

4. A0A6G5ZVY6_9SAUR **Mass:** 24441 **Score:** 26 **Expect:** 3.2 **Matches:** 4
Veficolin 6 (Fragment) OS=Vipera anatolica senliki OX=2604287 PE=2 SV=1
A0A6G5ZUK5_9SAUR **Mass:** 26961 **Score:** 25 **Expect:** 4.4 **Matches:** 4
Veficolin 5 (Fragment) OS=Vipera anatolica senliki OX=2604287 PE=2 SV=1

---

5. PA2BA_VIPRE **Mass:** 2608 **Score:** 25 **Expect:** 4.4 **Matches:** 2

Basic phospholipase A2 homolog Vur-S49 analog (Fragment) OS=Vipera renardi OX=927686 PE=1 SV=1

6. Q6A3C6_VIPAM **Mass:** 16212 **Score:** 22 **Expect:** 8.1 **Matches:** 3
Ammodytin I2(A) isoform OS=Vipera ammodytes ruffoi OX=246549 PE=2 SV=1

7. DIS_MACLO **Mass:** 4854 **Score:** 20 **Expect:** 13 **Matches:** 3
Disintegrin obtustatin OS=Macrovipera lebetina obtusa OX=209528 PE=1 SV=1

8. A0A6G5ZW74_9SAUR **Mass:** 32808 **Score:** 19 **Expect:** 17 **Matches:** 6
Veficolin 9 (Fragment) OS=Vipera anatolica senliki OX=2604287 PE=2 SV=1
A0A6G5ZVU3_9SAUR **Mass:** 11896 **Score:** 14 **Expect:** 54 **Matches:** 2
Veficolin 4 (Fragment) OS=Vipera anatolica senliki OX=2604287 PE=2 SV=1

9. Q7LZQ5_VIPAS **Mass:** 6213 **Score:** 19 **Expect:** 20 **Matches:** 2
Phospholipase A2 II (Fragment) OS=Vipera aspis OX=8706 PE=3 SV=1
Q6A3D8_VIPAP **Mass:** 8011 **Score:** 17 **Expect:** 29 **Matches:** 2
Ammodytin I2(A) isoform OS=Vipera aspis aspis OX=194601 PE=2 SV=1

10. K4GRJ2_DABRR **Mass:** 41352 **Score:** 18 **Expect:** 22 **Matches:** 4
Solute carrier family 8 member 3 (Fragment) OS=Daboia russelii OX=8707 GN=SLC8A3 PE=4 SV=1

Search Parameters

Type of search : Peptide Mass Fingerprint
Enzyme : Trypsin
Fixed modifications : Carbamidomethyl (C)
Variable modifications : Oxidation (M)
Mass values : Monoisotopic
Protein Mass : Unrestricted
Peptide Mass Tolerance : ± 100 ppm
Peptide Charge State : 1+
Max Missed Cleavages : 1
Number of queries : 93
Selected for scoring : 57

Mascot: http://www.matrixscience.com/

Mascot Search Results

```
User          :
Email         :
Search title  : vipera_all_Lift file:\\Proteomics1\d\data\2105\210505_tliepol_1_68252-68266\68252_68266_lpardo\0_G9\1\0_G9.xml
Database      : vipera  (1414 sequences; 347452 residues)
Timestamp     : 5 May 2021 at 09:59:37 GMT
Protein hits  : PA2A1_MACLB      Acidic phospholipase A2 1 OS=Macrovipera lebetina OX=8709 PE=1 SV=1
                PA2A2_DABRR      Acidic phospholipase A2 Drk-a2 OS=Daboia russelii OX=8707 PE=2 SV=1
                PA2BS_VIPRE      Basic phospholipase A2 homolog Vur-S49 OS=Vipera renardi OX=927686 PE=2 SV=1
                VM3VB_MACLB      Zinc metalloproteinase-disintegrin-like VLAIP-B OS=Macrovipera lebetina OX=8709 PE=1 SV=1
                A0A223PK32_DABRR Atrial natriuretic peptide receptor 3 (Fragment) OS=Daboia russelii OX=8707 PE=2 SV=1
                A0A6G5ZUK1_9SAUR Veficolin 7 (Fragment) OS=Vipera anatolica senliki OX=2604287 PE=2 SV=1
                A0A6G5ZUQ8_9SAUR Rabankyrin OS=Vipera anatolica senliki OX=2604287 PE=2 SV=1
```

Mascot Score Histogram

Ions score is -10*Log(P), where P is the probability that the observed match is a random event.
Individual ions scores > 15 indicate identity or extensive homology (p<0.05).
Protein scores are derived from ions scores as a non-probabilistic basis for ranking protein hits.

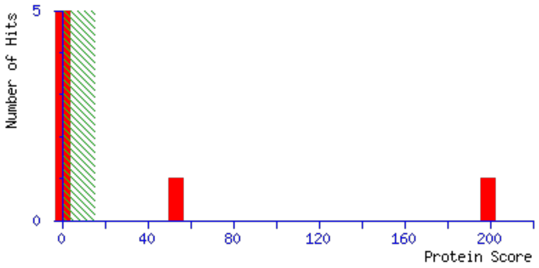

Peptide Summary Report

Format As: Peptide Summary — Help

Significance threshold p< 0.05 — Max. number of hits 10

Standard scoring ○ MudPIT scoring ◉ — Ions score or expect cut-off 0 — Show sub-sets 0

Show pop-ups ◉ Suppress pop-ups ○ — Sort unassigned Decreasing Score — Require bold red ☐

Overview Table

Click on column header to jump to entry in results list.
Move mouse over any indicator to highlight identical peptides.
Click on an indicator to see details of individual match.
Use check boxes to select sub-set of queries for new search.

Mouse over: -Query- -Accession- -Sequence-

Hit:	1	2	3	4	5	6	7
☑ 817.1353 (1+)							
☑ 831.0894 (1+)							
☑ 837.0987 (1+)							
☑ 839.1181 (1+)							
☑ 852.3661 (1+)							
☑ 855.0858 (1+)							
☑ 859.0803 (1+)							
☑ 865.5262 (1+)							
☑ 868.5601 (1+)							
☑ 871.0599 (1+)							
☑ 874.0343 (1+)							
☑ 896.0194 (1+)							
☑ 903.4049 (1+)							
☑ 909.3860 (1+)	●	●					
☑ 936.2407 (1+)							
☑ 952.4442 (1+)							
☑ 987.5164 (1+)	●	●	●				
☑ 1011.4809 (1+)							
☑ 1022.1437 (1+)							
☑ 1022.4987 (1+)							
☑ 1044.1191 (1+)							

☑ 1049.4942 (1+)							
☑ 1064.0866 (1+)							
☑ 1066.1056 (1+)							
☑ 1068.5038 (1+)							
☑ 1082.0794 (1+)							
☑ 1113.6442 (1+)							
☑ 1149.4939 (1+)							
☑ 1157.6529 (1+)						●	●
☑ 1161.6449 (1+)							
☑ 1173.6476 (1+)							
☑ 1189.6416 (1+)							
☑ 1193.6342 (1+)							
☑ 1195.5987 (1+)							
☑ 1205.6345 (1+)							
☑ 1221.6251 (1+)							
☑ 1234.6887 (1+)							
☑ 1238.6385 (1+)							
☑ 1249.1265 (1+)							
☑ 1255.6430 (1+)							
☑ 1267.6252 (1+)							
☑ 1271.1047 (1+)							
☑ 1283.6191 (1+)							
☑ 1285.7483 (1+)							
☑ 1305.5768 (1+)							
☑ 1311.7646 (1+)							
☑ 1323.6353 (1+)							
☑ 1330.5501 (1+)							
☑ 1339.6298 (1+)	●	●					
☑ 1343.5209 (1+)							
☑ 1346.5381 (1+)							
☑ 1355.6208 (1+)	●	●					
☑ 1361.5885 (1+)							
☑ 1368.4953 (1+)							
☑ 1372.5633 (1+)							
☑ 1377.5715 (1+)							
☑ 1393.5665 (1+)							
☑ 1395.7189 (1+)							
☑ 1411.7132 (1+)							
☑ 1433.6712 (1+)							
☑ 1476.1124 (1+)							
☑ 1497.7909 (1+)							
☑ 1625.8814 (1+)							
☑ 1636.7437 (1+)					●		
☑ 1674.6785 (1+)							
☑ 1698.8155 (1+)							
☑ 1718.7743 (1+)							
☑ 1731.7859 (1+)							
☑ 1733.7804 (1+)							
☑ 1736.7580 (1+)							
☑ 1740.7770 (1+)							
☑ 1748.7813 (1+)	●						
☑ 1754.7753 (1+)							
☑ 1756.7402 (1+)							
☑ 1764.8295 (1+)							
☑ 1769.7342 (1+)							
☑ 1771.7337 (1+)							
☑ 1941.8846 (1+)	●			●			
☑ 1967.8497 (1+)							
☑ 2005.7944 (1+)							
☑ 2118.7803 (1+)							
☑ 2134.9720 (1+)							
☑ 2217.9430 (1+)							
☑ 2234.8390 (1+)	●						
☑ 2566.1228 (1+)							
☑ 2618.1553 (1+)							
☑ 2636.1695 (1+)							
☑ 2663.1677 (1+)							

☑ 2682.1808 (1+)	●
☑ 2708.1954 (1+)	
☑ 2720.1183 (1+)	
☑ 2724.1914 (1+)	
☑ 3347.6860 (1+)	

Select All | Select None | Search Selected | ☐ **Error tolerant** | Archive Report

1. PA2A1_MACLB **Mass:** 16337 **Score:** 199 **Matches:** 8(4) **Sequences:** 7(4)
Acidic phospholipase A2 1 OS=Macrovipera lebetina OX=8709 PE=1 SV=1
☐ Check to include this hit in error tolerant search or archive report

Query	Observed	Mr(expt)	Mr(calc)	ppm	Miss	Score	Expect	Rank	Unique	Peptide
14	909.3860	908.3787	908.3480	33.8	0	6	1.5	1		R.AVCECDR.V
17	987.5164	986.5092	986.4781	31.5	0	4	1.5	1		K.GKPQDATDR.C
49	1339.6298	1338.6225	1338.5988	17.7	0	52	1e-005	1	U	K.YMLYSLFDCK.E
52	1355.6208	1354.6135	1354.5937	14.6	0	(2)	0.95	1	U	K.YMLYSLFDCK.E + Oxidation (M)
72	1748.7813	1747.7740	1747.7545	11.1	0	13	0.21	1	U	R.VAAICFGENMNTYDK.K + Oxidation (M)
78	1941.8846	1940.8773	1940.8536	12.2	1	24	0.0039	1	U	K.YMLYSLFDCKEESEK.C
84	2234.8390	2233.8317	2233.8108	9.36	0	43	5.2e-005	1	U	R.CCFVHDCCYGSVNGCDPK.L
89	2682.1808	2681.1736	2681.1374	13.5	0	120	9.4e-013	1	U	K.LSTYSYSFQNGDIVCGDDDPCLR.A

2. PA2A2_DABRR **Mass:** 16430 **Score:** 52 **Matches:** 4(1) **Sequences:** 3(1)
Acidic phospholipase A2 Drk-a2 OS=Daboia russelii OX=8707 PE=2 SV=1
☐ Check to include this hit in error tolerant search or archive report

Query	Observed	Mr(expt)	Mr(calc)	ppm	Miss	Score	Expect	Rank	Unique	Peptide
14	909.3860	908.3787	908.3480	33.8	0	6	1.5	1		R.AVCECDR.V
17	987.5164	986.5092	986.4781	31.5	0	4	1.5	1		K.GKPQDATDR.C
49	1339.6298	1338.6225	1338.5988	17.7	0	52	1e-005	1	U	K.YMLYSIFDCK.E
52	1355.6208	1354.6135	1354.5937	14.6	0	(2)	0.95	1	U	K.YMLYSIFDCK.E + Oxidation (M)

3. PA2BS_VIPRE **Mass:** 16504 **Score:** 0 **Matches:** 1(0) **Sequences:** 1(0)
Basic phospholipase A2 homolog Vur-S49 OS=Vipera renardi OX=927686 PE=2 SV=1
☐ Check to include this hit in error tolerant search or archive report

Query	Observed	Mr(expt)	Mr(calc)	ppm	Miss	Score	Expect	Rank	Unique	Peptide
17	987.5164	986.5092	986.5145	-5.41	1	4	1.5	1	U	K.GKPKDATDR.C

Proteins matching the same set of peptides:
PA2HL_VIPAA **Mass:** 16481 **Score:** 0 **Matches:** 1(0) **Sequences:** 1(0)
Basic phospholipase A2 homolog ammodytin L OS=Vipera ammodytes ammodytes OX=8705 PE=1 SV=1
Q6A368_VIPAM **Mass:** 16489 **Score:** 0 **Matches:** 1(0) **Sequences:** 1(0)
Ammodytin L(3) variant OS=Vipera ammodytes montandoni OX=235554 PE=2 SV=1
Q6A369_VIPAM **Mass:** 16449 **Score:** 0 **Matches:** 1(0) **Sequences:** 1(0)
Ammodytin L(3) variant OS=Vipera ammodytes montandoni OX=235554 PE=2 SV=1
Q6A370_VIPAM **Mass:** 16461 **Score:** 0 **Matches:** 1(0) **Sequences:** 1(0)
Ammodytin L(3) variant OS=Vipera ammodytes montandoni OX=235554 PE=2 SV=1
Q6A371_VIPAM **Mass:** 16461 **Score:** 0 **Matches:** 1(0) **Sequences:** 1(0)
Ammodytin L(3) isoform OS=Vipera ammodytes montandoni OX=235554 PE=2 SV=1
Q6A374_VIPAA **Mass:** 16399 **Score:** 0 **Matches:** 1(0) **Sequences:** 1(0)
Ammodytin L(1'') variant OS=Vipera ammodytes ammodytes OX=8705 PE=2 SV=1
Q6A378_VIPAA **Mass:** 16459 **Score:** 0 **Matches:** 1(0) **Sequences:** 1(0)
Ammodytin L(1'') isoform OS=Vipera ammodytes ammodytes OX=8705 PE=2 SV=1
Q6A380_VIPAA **Mass:** 16415 **Score:** 0 **Matches:** 1(0) **Sequences:** 1(0)
Ammodytin L(1'') variant OS=Vipera ammodytes ammodytes OX=8705 PE=2 SV=1
Q6A381_VIPAA **Mass:** 16433 **Score:** 0 **Matches:** 1(0) **Sequences:** 1(0)
Ammodytin L(1'') variant OS=Vipera ammodytes ammodytes OX=8705 PE=2 SV=1
Q6A382_VIPAA **Mass:** 16431 **Score:** 0 **Matches:** 1(0) **Sequences:** 1(0)
Ammodytin L(1'') variant OS=Vipera ammodytes ammodytes OX=8705 PE=2 SV=1
Q6A383_VIPAA **Mass:** 16503 **Score:** 0 **Matches:** 1(0) **Sequences:** 1(0)
Ammodytin L(1') isoform OS=Vipera ammodytes ammodytes OX=8705 PE=2 SV=1
Q6A385_VIPBB **Mass:** 16442 **Score:** 0 **Matches:** 1(0) **Sequences:** 1(0)
Ammodytin L(2) variant OS=Vipera berus berus OX=31156 PE=2 SV=1
Q6A386_VIPAM **Mass:** 16504 **Score:** 0 **Matches:** 1(0) **Sequences:** 1(0)
Ammodytin L(4) isoform OS=Vipera ammodytes ruffoi OX=246549 PE=2 SV=1
Q6A387_VIPAM **Mass:** 16471 **Score:** 0 **Matches:** 1(0) **Sequences:** 1(0)
Ammodytin L(1) isoform OS=Vipera ammodytes ruffoi OX=246549 PE=2 SV=1
Q6A388_VIPBB **Mass:** 16415 **Score:** 0 **Matches:** 1(0) **Sequences:** 1(0)
Ammodytin L(2) variant OS=Vipera berus berus OX=31156 PE=2 SV=1
Q6A389_VIPBB **Mass:** 16410 **Score:** 0 **Matches:** 1(0) **Sequences:** 1(0)
Ammodytin L(2) variant OS=Vipera berus berus OX=31156 PE=2 SV=1
Q6A390_VIPBB **Mass:** 16398 **Score:** 0 **Matches:** 1(0) **Sequences:** 1(0)
Ammodytin L(2) variant OS=Vipera berus berus OX=31156 PE=2 SV=1
Q6A391_VIPBB **Mass:** 16411 **Score:** 0 **Matches:** 1(0) **Sequences:** 1(0)
Ammodytin L(2) variant OS=Vipera berus berus OX=31156 PE=2 SV=1

Q6A394_VIPBB **Mass:** 16414 **Score:** 0 **Matches:** 1(0) **Sequences:** 1(0)
Ammodytin L(2) isoform OS=Vipera berus berus OX=31156 PE=2 SV=1

---

4. VM3VB_MACLB **Score:** 0 **Matches:** 1(0) **Sequences:** 1(0)
Zinc metalloproteinase-disintegrin-like VLAIP-B OS=Macrovipera lebetina OX=8709 PE=1 SV=1
☐ Check to include this hit in error tolerant search or archive report

Query	Observed	Mr(expt)	Mr(calc)	ppm	Miss	Score	Expect	Rank	Unique	Peptide
78	1941.8846	1940.8773	1940.8940	-8.63	1	2	0.7	2	U	R.IDCTCGAKSCIMSGILR.C

---

5. A0A223PK32_DABRR **Mass:** 57907 **Score:** 0 **Matches:** 1(0) **Sequences:** 1(0)
Atrial natriuretic peptide receptor 3 (Fragment) OS=Daboia russelii OX=8707 PE=2 SV=1
☐ Check to include this hit in error tolerant search or archive report

Query	Observed	Mr(expt)	Mr(calc)	ppm	Miss	Score	Expect	Rank	Unique	Peptide
64	**1636.7437**	**1635.7364**	**1635.7100**	**16.1**	**1**	**2**	**0.7**	**1**	**U**	**K.FRLFYSDSDCGNR.A**

---

6. A0A6G5ZUK1_9SAUR **Mass:** 15084 **Score:** 0 **Matches:** 1(0) **Sequences:** 1(0)
Veficolin 7 (Fragment) OS=Vipera anatolica senliki OX=2604287 PE=2 SV=1
☐ Check to include this hit in error tolerant search or archive report

Query	Observed	Mr(expt)	Mr(calc)	ppm	Miss	Score	Expect	Rank	Unique	Peptide
29	**1157.6529**	**1156.6456**	**1156.6088**	**31.9**	**1**	**2**	**0.71**	**1**	**U**	**K.DKLQDPNSLK.C**

Proteins matching the same set of peptides:
A0A6G5ZVU3_9SAUR **Mass:** 11896 **Score:** 0 **Matches:** 1(0) **Sequences:** 1(0)
Veficolin 4 (Fragment) OS=Vipera anatolica senliki OX=2604287 PE=2 SV=1
A0A6G5ZVX6_9SAUR **Mass:** 37165 **Score:** 0 **Matches:** 1(0) **Sequences:** 1(0)
Veficolin 1 OS=Vipera anatolica senliki OX=2604287 PE=2 SV=1
A0A6G5ZW74_9SAUR **Mass:** 32808 **Score:** 0 **Matches:** 1(0) **Sequences:** 1(0)
Veficolin 9 (Fragment) OS=Vipera anatolica senliki OX=2604287 PE=2 SV=1

---

7. A0A6G5ZUQ8_9SAUR **Score:** 0 **Matches:** 1(0) **Sequences:** 1(0)
Rabankyrin OS=Vipera anatolica senliki OX=2604287 PE=2 SV=1
☐ Check to include this hit in error tolerant search or archive report

Query	Observed	Mr(expt)	Mr(calc)	ppm	Miss	Score	Expect	Rank	Unique	Peptide
29	1157.6529	1156.6456	1156.5360	94.8	0	1	0.83	2	U	R.EPGAAEQVDNK.G

Peptide matches not assigned to protein hits: (no details means no match)

	Query	Observed	Mr(expt)	Mr(calc)	ppm	Miss	Score	Expect	Rank	Unique	Peptide
☑	1	*817.1353*	*816.1281*								
☑	2	*831.0894*	*830.0821*								
☑	3	*837.0987*	*836.0914*								
☑	4	*839.1181*	*838.1108*								
☑	5	*852.3661*	*851.3588*								
☑	6	*855.0858*	*854.0786*								
☑	7	*859.0803*	*858.0730*								
☑	8	*865.5262*	*864.5190*								
☑	9	*868.5601*	*867.5529*								
☑	10	*871.0599*	*870.0526*								
☑	11	*874.0343*	*873.0270*								
☑	12	*896.0194*	*895.0121*								
☑	13	*903.4049*	*902.3976*								
☑	15	*936.2407*	*935.2334*								
☑	16	*952.4442*	*951.4369*								
☑	18	*1011.4809*	*1010.4736*								
☑	19	*1022.1437*	*1021.1364*								
☑	20	*1022.4987*	*1021.4914*								
☑	21	*1044.1191*	*1043.1118*								
☑	22	*1049.4942*	*1048.4869*								
☑	23	*1064.0866*	*1063.0794*								
☑	24	*1066.1056*	*1065.0983*								
☑	25	*1068.5038*	*1067.4966*								
☑	26	*1082.0794*	*1081.0722*								
☑	27	*1113.6442*	*1112.6369*								
☑	28	*1149.4939*	*1148.4866*								
☑	30	*1161.6449*	*1160.6376*								
☑	31	*1173.6476*	*1172.6403*								
☑	32	*1189.6416*	*1188.6343*								
☑	33	*1193.6342*	*1192.6269*								
☑	34	*1195.5987*	*1194.5914*								
☑	35	*1205.6345*	*1204.6272*								
☑	36	*1221.6251*	*1220.6178*								
☑	37	*1234.6887*	*1233.6814*								
☑	38	*1238.6385*	*1237.6313*								

☑	39	1249.1265	1248.1192
☑	40	1255.6430	1254.6357
☑	41	1267.6252	1266.6180
☑	42	1271.1047	1270.0975
☑	43	1283.6191	1282.6119
☑	44	1285.7483	1284.7410
☑	45	1305.5768	1304.5696
☑	46	1311.7646	1310.7573
☑	47	1323.6353	1322.6280
☑	48	1330.5501	1329.5428
☑	50	1343.5209	1342.5136
☑	51	1346.5381	1345.5308
☑	53	1361.5885	1360.5812
☑	54	1368.4953	1367.4880
☑	55	1372.5633	1371.5560
☑	56	1377.5715	1376.5642
☑	57	1393.5665	1392.5592
☑	58	1395.7189	1394.7116
☑	59	1411.7132	1410.7059
☑	60	1433.6712	1432.6639
☑	61	1476.1124	1475.1051
☑	62	1497.7909	1496.7836
☑	63	1625.8814	1624.8741
☑	65	1674.6785	1673.6712
☑	66	1698.8155	1697.8082
☑	67	1718.7743	1717.7670
☑	68	1731.7859	1730.7786
☑	69	1733.7804	1732.7732
☑	70	1736.7580	1735.7507
☑	71	1740.7770	1739.7697
☑	73	1754.7753	1753.7681
☑	74	1756.7402	1755.7330
☑	75	1764.8295	1763.8222
☑	76	1769.7342	1768.7270
☑	77	1771.7337	1770.7264
☑	79	1967.8497	1966.8424
☑	80	2005.7944	2004.7872
☑	81	2118.7803	2117.7730
☑	82	2134.9720	2133.9647
☑	83	2217.9430	2216.9357
☑	85	2566.1228	2565.1156
☑	86	2618.1553	2617.1480
☑	87	2636.1695	2635.1622
☑	88	2663.1677	2662.1604
☑	90	2708.1954	2707.1881
☑	91	2720.1183	2719.1111
☑	92	2724.1914	2723.1841
☑	93	3347.6860	3346.6787

Search Parameters

```
Type of search         : MS/MS Ion Search
Enzyme                 : Trypsin
Fixed modifications    : Carbamidomethyl (C)
Variable modifications : Oxidation (M)
Mass values            : Monoisotopic
Protein Mass           : Unrestricted
Peptide Mass Tolerance : ± 100 ppm
Fragment Mass Tolerance: ± 0.7 Da
Max Missed Cleavages   : 1
Instrument type        : MALDI-TOF-TOF
Number of queries      : 93
```

Mascot: http://www.matrixscience.com/

Mascot Search Results

```
User          :
Email         :
Search title  : vipera_all_Lift file:\\Proteomics1\d\data\2105\210505_tliepol_1_68252-68266\68252_68266_lpardo\0_G9\1\0_G9.xml
Database      : vipera  (1414 sequences; 347452 residues)
Timestamp     : 5 May 2021 at 09:59:37 GMT
Warning       : A Peptide summary report will usually give a much clearer picture of MS/MS search results.
Top Score     : 318 for PA2A1_MACLB, Acidic phospholipase A2 1 OS=Macrovipera lebetina OX=8709 PE=1 SV=1
```

Mascot Score Histogram

Protein score is -10*Log(P), where P is the probability that the observed match is a random event.
Protein scores greater than 44 are significant (p<0.05).
Protein scores are derived from ions scores as a non-probabilistic basis for ranking protein hits.

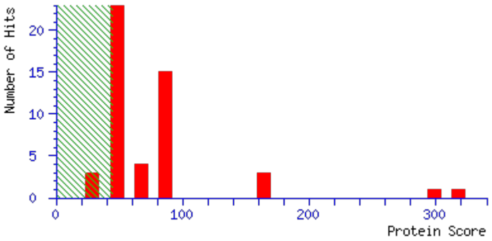

Protein Summary Report

Format As | Protein Summary (deprecated) | Help

Significance threshold p< 0.05 Max. number of hits 50

Overview Table

Click on column header to jump to entry in results list.
Move mouse over any indicator to highlight identical peptides.
Click on an indicator to see details of individual match.
Use check boxes to select sub-set of queries for new search.

Mouse over: -Query- -Accession- -Sequence-

Hit:	1	2	3	4	5	6	7	8	9	10	11	12	13	14	15	16	17	18	19	20	21	22	23	24	25	26	27	28	29	30	31	32	33	34	35	36	37	38	39	40	41	42	43	44	45	46	47	48	49	50
☑ 817.1353 (1+)																																																		
☑ 831.0894 (1+)																																																		
☑ 837.0987 (1+)																																																		
☑ 839.1181 (1+)																																																		
☑ 852.3661 (1+)																																																		
☑ 855.0858 (1+)																																																		
☑ 859.0803 (1+)																																																		
☑ 865.5262 (1+)																																																		
☑ 868.5601 (1+)																																																		
☑ 871.0599 (1+)																																																		
☑ 874.0343 (1+)																																																		
☑ 896.0194 (1+)																																																		
☑ 903.4049 (1+)																																																		
☑ 909.3860 (1+)	●	●	●	●	●	●	●	●	●	●	●	●	●	●	●	●	●	●	●	●	●	●	●	●	●	●	●	●	●	●	●	●	●	●	●	●	●	●	●	●	●	●	●	●	●	●	●	●	●	
☑ 936.2407 (1+)																																																		
☑ 952.4442 (1+)																																																		
☑ 987.5164 (1+)	●	●	●	●	●																●																											●		
☑ 1011.4809 (1+)																																																		
☑ 1022.1437 (1+)																																																		
☑ 1022.4987 (1+)																																																		
☑ 1044.1191 (1+)																																																		
☑ 1049.4942 (1+)																																																		
☑ 1064.0866 (1+)																																																		
☑ 1066.1056 (1+)																																																		
☑ 1068.5038 (1+)																																																		
☑ 1082.0794 (1+)																																																		
☑ 1113.6442 (1+)																																																		
☑ 1149.4939 (1+)																																																		
☑ 1157.6529 (1+)																																																		
☑ 1161.6449 (1+)																																																		
☑ 1173.6476 (1+)																																																		
☑ 1189.6416 (1+)																																																		
☑ 1193.6342 (1+)																																																		
☑ 1195.5987 (1+)																																																		
☑ 1205.6345 (1+)																																																		

☑ 1221.6251 (1+)																																																		
☑ 1234.6887 (1+)																																																		
☑ 1238.6385 (1+)																																																		
☑ 1249.1265 (1+)																																																		
☑ 1255.6430 (1+)																																																		
☑ 1267.6252 (1+)																																																		
☑ 1271.1047 (1+)																																																		
☑ 1283.6191 (1+)																																																		
☑ 1285.7483 (1+)																																																		
☑ 1305.5768 (1+)																																																		
☑ 1311.7646 (1+)																																																		
☑ 1323.6353 (1+)																																																		
☑ 1330.5501 (1+)																																																		
☑ 1339.6298 (1+)	●	●				●	●	●	●	●	●	●	●	●	●	●	●	●	●	●	●	●	●	●																										
☑ 1343.5209 (1+)																																																		
☑ 1346.5381 (1+)																																																		
☑ 1355.6208 (1+)	●	●				●	●	●	●	●	●	●	●	●	●	●	●	●	●	●	●	●	●	●																										
☑ 1361.5885 (1+)																																																		
☑ 1368.4953 (1+)																																																		
☑ 1372.5633 (1+)																																																		
☑ 1377.5715 (1+)																																																		
☑ 1393.5665 (1+)																																																		
☑ 1395.7189 (1+)																																																		
☑ 1411.7132 (1+)																																																		
☑ 1433.6712 (1+)																																																		
☑ 1476.1124 (1+)																																																		
☑ 1497.7909 (1+)																																																		
☑ 1625.8814 (1+)																																																		
☑ 1636.7437 (1+)																																																		
☑ 1674.6785 (1+)																																																		
☑ 1698.8155 (1+)																																																		
☑ 1718.7743 (1+)																																																		
☑ 1731.7859 (1+)																																																		
☑ 1733.7804 (1+)																																																		
☑ 1736.7580 (1+)																																																		
☑ 1740.7770 (1+)																																																		
☑ 1748.7813 (1+)	●		●	●		●	●	●	●	●	●	●	●	●	●	●	●	●	●	●					●	●	●	●	●	●	●	●	●	●	●	●	●	●	●	●	●	●	●	●	●	●	●		●	●
☑ 1754.7753 (1+)																																																		
☑ 1756.7402 (1+)																																																		
☑ 1764.8295 (1+)																																																		
☑ 1769.7342 (1+)																																																		
☑ 1771.7337 (1+)																																																		
☑ 1941.8846 (1+)	●	●																																																
☑ 1967.8497 (1+)																																																		
☑ 2005.7944 (1+)																																																		
☑ 2118.7803 (1+)																																																		
☑ 2134.9720 (1+)																																																		
☑ 2217.9430 (1+)																																																		
☑ 2234.8390 (1+)	●	●																																																
☑ 2566.1228 (1+)																																																		
☑ 2618.1553 (1+)																																																		
☑ 2636.1695 (1+)																																																		
☑ 2663.1677 (1+)																																																		
☑ 2682.1808 (1+)	●	●	●	●	●																																													
☑ 2708.1954 (1+)																																																		
☑ 2720.1183 (1+)																																																		
☑ 2724.1914 (1+)																																																		
☑ 3347.6860 (1+)																																																		

Select All Select None Search Selected

Index

	Accession	Mass	Score	Description
1.	PA2A1_MACLB	16337	318	Acidic phospholipase A2 1 OS=Macrovipera lebetina OX=8709 PE=1 SV=1
2.	PA2A2_MACLB	16247	295	Acidic phospholipase A2 2 OS=Macrovipera lebetina OX=8709 PE=1 SV=1
3.	PA2A_MACLN	16243	168	Acidic phospholipase A2 MVL-PLA2 OS=Macrovipera lebetina transmediterranea OX=384075 PE=1 SV=1
4.	PA2A_DABPA	16277	168	Acidic phospholipase A2 VpaPLA2 OS=Daboia palaestinae OX=1170828 PE=1 SV=1
5.	PA2A7_DABPA	16190	157	Acidic phospholipase A2 VP7 OS=Daboia palaestinae OX=1170828 PE=3 SV=1
6.	Q6A3G8_VIPAA	15517	87	Ammodytin I1(A) variant OS=Vipera ammodytes ammodytes OX=8705 PE=2 SV=1
7.	Q7T1D1_VIPBB	16258	86	Ammodytin I1 OS=Vipera berus berus OX=31156 GN=AmtI1 PE=2 SV=1
8.	Q6A3G4_VIPAM	16280	86	Ammodytin I1(D) isoform OS=Vipera ammodytes montandoni OX=235554 PE=2 SV=1
9.	Q6A3N0_VIPAM	16279	86	Ammodytin I1(A) isoform OS=Vipera ammodytes ruffoi OX=246549 PE=2 SV=1
10.	Q6A3K0_VIPBB	16214	86	Ammodytin I1(B') variant OS=Vipera berus berus OX=31156 PE=2 SV=1
11.	Q6A3G5_VIPAE	16280	86	Ammodytin I1(D) isoform OS=Vipera ammodytes meridionalis OX=73841 PE=2 SV=1
12.	Q1RP80_VIPBN	16258	86	Phospholipase A2 ammodytin I1 OS=Vipera berus nikolskii OX=1808362 GN=I1 PE=2 SV=1
13.	Q6YC90_VIPAS	16279	86	Ammodytin I1 isoform 2 OS=Vipera aspis OX=8706 GN=AmtI1 PE=3 SV=1
14.	Q6A3I8_VIPUR	16290	86	Ammodytin I1(B) isoform OS=Vipera ursinii OX=103942 PE=2 SV=1
15.	Q6A3K8_VIPAP	16267	86	Ammodytin I1(A') isoform OS=Vipera aspis aspis OX=194601 PE=2 SV=1

**16.**	Q6A3K4_VIPAP	16252	86	Ammodytin I1(A) variant OS=Vipera aspis aspis OX=194601 PE=2 SV=1
**17.**	Q6A3K7_VIPAP	16279	86	Ammodytin I1(A) isoform OS=Vipera aspis aspis OX=194601 PE=2 SV=1
**18.**	Q6A3M8_VIPAM	16378	86	Ammodytin I1(A) variant OS=Vipera ammodytes ruffoi OX=246549 PE=2 SV=1
**19.**	Q6A3M9_VIPAM	16286	86	Ammodytin I1(B) isoform OS=Vipera ammodytes ruffoi OX=246549 PE=2 SV=1
**20.**	PA2A1_VIPAA	16279	86	Acidic phospholipase A2 ammodytin I1 OS=Vipera ammodytes ammodytes OX=8705 PE=2 SV=1
**21.**	PA2A2_DABRR	16430	77	Acidic phospholipase A2 Drk-a2 OS=Daboia russelii OX=8707 PE=2 SV=1
**22.**	PA2AB_DABSI	15673	74	Acidic phospholipase A2 daboiatoxin B chain (Fragment) OS=Daboia siamensis OX=343250 PE=1 SV=1
**23.**	Q6A3K3_VIPAP	16307	67	Ammodytin I1(A) variant OS=Vipera aspis aspis OX=194601 PE=2 SV=1
**24.**	PA2A1_VIPRE	16228	67	Acidic phospholipase A2 PL1 OS=Vipera renardi OX=927686 PE=2 SV=1
**25.**	Q6A3J3_VIPBB	16280	42	Ammodytin I1(C) variant OS=Vipera berus berus OX=31156 PE=2 SV=1
**26.**	Q6A3H6_VIPAS	16258	42	Ammodytin I1(C) isoform OS=Vipera aspis atra OX=246182 PE=2 SV=1
**27.**	Q6A3J2_VIPBB	16244	42	Ammodytin I1(C) variant OS=Vipera berus berus OX=31156 PE=2 SV=1
**28.**	Q6A3J0_VIPBB	16280	42	Ammodytin I1(C) variant OS=Vipera berus berus OX=31156 PE=2 SV=1
**29.**	Q6A3H7_VIPAS	16244	42	Ammodytin I1(F) isoform OS=Vipera aspis atra OX=246182 PE=2 SV=1
**30.**	Q6A3H4_VIPAS	16244	42	Ammodytin I1(C) isoform OS=Vipera aspis atra OX=246182 PE=2 SV=1
**31.**	Q7T1D3_VIPAS	16258	42	Ammodytin I1 isoform 1 OS=Vipera aspis OX=8706 GN=AmtI1 PE=3 SV=1
**32.**	Q6A3I1_VIPAP	16288	42	Ammodytin I1(C) variant OS=Vipera aspis aspis OX=194601 PE=2 SV=1
**33.**	Q6A3I0_VIPAP	16272	42	Ammodytin I1(C) variant OS=Vipera aspis aspis OX=194601 PE=2 SV=1
**34.**	Q6A3I4_VIPAP	16272	42	Ammodytin I1(C) variant OS=Vipera aspis aspis OX=194601 PE=2 SV=1
**35.**	Q6A3H8_VIPAP	16330	42	Ammodytin I1(C) variant OS=Vipera aspis aspis OX=194601 PE=2 SV=1
**36.**	Q6A3K9_VIPAP	16202	42	Ammodytin I1(E) variant OS=Vipera aspis aspis OX=194601 PE=2 SV=1
**37.**	Q6A3I3_VIPAP	16286	42	Ammodytin I1(C) isoform OS=Vipera aspis aspis OX=194601 PE=2 SV=1
**38.**	Q6A3M5_VIPAP	16257	42	Ammodytin I1(C) variant OS=Vipera aspis aspis OX=194601 PE=2 SV=1
**39.**	Q6A3L1_VIPAP	16272	42	Ammodytin I1(E) isoform OS=Vipera aspis aspis OX=194601 PE=2 SV=1
**40.**	Q6A3L0_VIPAP	16360	42	Ammodytin I1(E) variant OS=Vipera aspis aspis OX=194601 PE=2 SV=1
**41.**	Q6A3G1_VIPAP	16246	42	Ammodytin I1(C') isoform OS=Vipera aspis aspis OX=194601 PE=2 SV=1
**42.**	Q6A3F7_VIPAP	16310	42	Ammodytin I1(C) variant OS=Vipera aspis aspis OX=194601 PE=2 SV=1
**43.**	Q7T1D4_VIPAP	16258	42	Ammodytin I1 OS=Vipera aspis aspis OX=194601 GN=AmtI1 PE=2 SV=1
**44.**	Q7T1D2_VIPAZ	16258	42	Ammodytin I1 OS=Vipera aspis zinnikeri OX=55427 GN=AmtI1 PE=2 SV=1
**45.**	Q6A3J4_VIPBB	16258	42	Ammodytin I1(C) isoform OS=Vipera berus berus OX=31156 PE=2 SV=1
**46.**	Q6A3H2_VIPAS	16198	42	Ammodytin I1(C) variant OS=Vipera aspis atra OX=246182 PE=2 SV=1
**47.**	Q6A3J6_VIPAZ	16330	42	Ammodytin I1(C) isoform OS=Vipera aspis zinnikeri OX=55427 PE=2 SV=1
**48.**	PA2B_DABRR	14415	35	Basic phospholipase A2 RVV-VD OS=Daboia russelii OX=8707 PE=1 SV=1
**49.**	Q6A3H1_VIPAS	16292	34	Ammodytin I1(C) variant OS=Vipera aspis atra OX=246182 PE=2 SV=1
**50.**	Q6A3F6_VIPAP	16200	29	Ammodytin I1 (C) variant OS=Vipera aspis aspis OX=194601 PE=2 SV=1

Results List

1. PA2A1_MACLB **Mass:** 16337 **Score:** 318 **Expect:** 2.2e-029 **Matches:** 8

Acidic phospholipase A2 1 OS=Macrovipera lebetina OX=8709 PE=1 SV=1

Observed	Mr(expt)	Mr(calc)	ppm	Start	End	Miss	Ions	Peptide
909.3860	908.3787	908.3480	33.8	100 -	106	0	6	R.AVCECDR.V
987.5164	986.5092	986.4781	31.5	50 -	58	0	4	K.GKPQDATDR.C
1339.6298	1338.6225	1338.5988	17.7	123 -	132	0	52	K.YMLYSLFDCK.E
1355.6208	1354.6135	1354.5937	14.6	123 -	132	0	(2)	K.YMLYSLFDCK.E + Oxidation (M)
1748.7813	1747.7740	1747.7545	11.1	107 -	121	0	13	R.VAAICFGENMNTYDK.K + Oxidation (M)
1941.8846	1940.8773	1940.8536	12.2	123 -	137	1	24	K.YMLYSLFDCKEESEK.C
2234.8390	2233.8317	2233.8108	9.36	59 -	76	0	43	R.CCFVHDCCYGSVNGCDPK.L
2682.1808	2681.1736	2681.1374	13.5	77 -	99	0	120	K.LSTYSYSFQNGDIVCGDDDPCLR.A

No match to: 817.1353, 831.0894, 837.0987, 839.1181, 852.3661, 855.0858, 859.0803, 865.5262, 868.5601, 871.0599, 874.0343, 896.0194, 903.4049, 936.2407, 952.4442, 1011.4809, 1022.1437, 1022.4987, 1044.1191, 1049.4942, 1064.0866, 1066.1056, 1068.5038, 1082.0794, 1113.6442, 1149.4939, 1157.6529, 1161.6449, 1173.6476, 1189.6416, 1193.6342, 1195.5987, 1205.6345, 1221.6251, 1234.6887, 1238.6385, 1249.1265, 1255.6430, 1267.6252, 1271.1047, 1283.6191, 1285.7483, 1305.5768, 1311.7646, 1323.6353, 1330.5501, 1343.5209, 1346.5381, 1361.5885, 1368.4953, 1372.5633, 1377.5715, 1393.5665, 1395.7189, 1411.7132, 1433.6712, 1476.1124, 1497.7909, 1625.8814, 1636.7437, 1674.6785, 1698.8155, 1718.7743, 1731.7859, 1733.7804, 1736.7580, 1740.7770, 1754.7753, 1756.7402, 1764.8295, 1769.7342, 1771.7337, 1967.8497, 2005.7944, 2118.7803, 2134.9720, 2217.9430, 2566.1228, 2618.1553, 2636.1695, 2663.1677, 2708.1954, 2720.1183, 2724.1914, 3347.6860

2. PA2A2_MACLB **Mass:** 16247 **Score:** 295 **Expect:** 4.5e-027 **Matches:** 7

Acidic phospholipase A2 2 OS=Macrovipera lebetina OX=8709 PE=1 SV=1

Observed	Mr(expt)	Mr(calc)	ppm	Start	End	Miss	Ions	Peptide
909.3860	908.3787	908.3480	33.8	100 -	106	0	6	R.AVCECDR.V
987.5164	986.5092	986.4781	31.5	50 -	58	0	4	K.GKPQDATDR.C
1339.6298	1338.6225	1338.5988	17.7	123 -	132	0	52	K.YMLYSLFDCK.E
1355.6208	1354.6135	1354.5937	14.6	123 -	132	0	(2)	K.YMLYSLFDCK.E + Oxidation (M)
1941.8846	1940.8773	1940.8536	12.2	123 -	137	1	24	K.YMLYSLFDCKEESEK.C
2234.8390	2233.8317	2233.8108	9.36	59 -	76	0	43	R.CCFVHDCCYGSVNGCDPK.L
2682.1808	2681.1736	2681.1374	13.5	77 -	99	0	120	K.LSTYSYSFQNGDIVCGDDDPCLR.A

No match to: 817.1353, 831.0894, 837.0987, 839.1181, 852.3661, 855.0858, 859.0803, 865.5262, 868.5601, 871.0599, 874.0343, 896.0194, 903.4049, 936.2407, 952.4442, 1011.4809, 1022.1437, 1022.4987, 1044.1191, 1049.4942, 1064.0866, 1066.1056, 1068.5038, 1082.0794, 1113.6442, 1149.4939, 1157.6529, 1161.6449, 1173.6476, 1189.6416, 1193.6342, 1195.5987, 1205.6345, 1221.6251, 1234.6887, 1238.6385, 1249.1265, 1255.6430, 1267.6252, 1271.1047, 1283.6191, 1285.7483, 1305.5768, 1311.7646, 1323.6353, 1330.5501, 1343.5209, 1346.5381, 1361.5885, 1368.4953, 1372.5633, 1377.5715, 1393.5665, 1395.7189, 1411.7132, 1433.6712, 1476.1124, 1497.7909, 1625.8814, 1636.7437, 1674.6785, 1698.8155, 1718.7743, 1731.7859, 1733.7804, 1736.7580, 1740.7770, 1748.7813, 1754.7753, 1756.7402, 1764.8295, 1769.7342, 1771.7337, 1967.8497, 2005.7944, 2118.7803, 2134.9720, 2217.9430, 2566.1228, 2618.1553, 2636.1695, 2663.1677, 2708.1954, 2720.1183, 2724.1914, 3347.6860

3. PA2A_MACLN **Mass:** 16243 **Score:** 168 **Expect:** 2.2e-014 **Matches:** 4

Acidic phospholipase A2 MVL-PLA2 OS=Macrovipera lebetina transmediterranea OX=384075 PE=1 SV=1

Observed	Mr(expt)	Mr(calc)	ppm	Start	End	Miss	Ions	Peptide
909.3860	908.3787	908.3480	33.8	100 -	106	0	6	R.AVCECDR.V
987.5164	986.5092	986.4781	31.5	50 -	58	0	4	K.GKPQDATDR.C
1748.7813	1747.7740	1747.7545	11.1	107 -	121	0	13	R.VAAICFGENMNTYDK.K + Oxidation (M)
2682.1808	2681.1736	2681.1374	13.5	77 -	99	0	120	K.LSTYSYSFQNGDIVCGDDDPCLR.A

No match to: 817.1353, 831.0894, 837.0987, 839.1181, 852.3661, 855.0858, 859.0803, 865.5262, 868.5601, 871.0599, 874.0343, 896.0194, 903.4049, 936.2407, 952.4442, 1011.4809, 1022.1437, 1022.4987, 1044.1191, 1049.4942, 1064.0866, 1066.1056, 1068.5038, 1082.0794, 1113.6442, 1149.4939, 1157.6529, 1161.6449, 1173.6476, 1189.6416, 1193.6342, 1195.5987, 1205.6345, 1221.6251, 1234.6887, 1238.6385, 1249.1265, 1255.6430, 1267.6252, 1271.1047, 1283.6191, 1285.7483, 1305.5768, 1311.7646, 1323.6353, 1330.5501, 1339.6298, 1343.5209, 1346.5381, 1355.6208, 1361.5885, 1368.4953, 1372.5633, 1377.5715, 1393.5665, 1395.7189, 1411.7132, 1433.6712, 1476.1124, 1497.7909, 1625.8814, 1636.7437, 1674.6785, 1698.8155, 1718.7743, 1731.7859, 1733.7804, 1736.7580, 1740.7770, 1754.7753, 1756.7402, 1764.8295, 1769.7342, 1771.7337, 1941.8846, 1967.8497, 2005.7944, 2118.7803, 2134.9720, 2217.9430, 2234.8390, 2566.1228, 2618.1553, 2636.1695, 2663.1677, 2708.1954, 2720.1183, 2724.1914, 3347.6860

4. PA2A_DABPA **Mass:** 16277 **Score:** 168 **Expect:** 2.2e-014 **Matches:** 4

Acidic phospholipase A2 VpaPLA2 OS=Daboia palaestinae OX=1170828 PE=1 SV=1

Observed	Mr(expt)	Mr(calc)	ppm	Start	End	Miss	Ions	Peptide
909.3860	908.3787	908.3480	33.8	100 -	106	0	6	R.AVCECDR.V
987.5164	986.5092	986.4781	31.5	50 -	58	0	4	K.GKPQDATDR.C
1748.7813	1747.7740	1747.7545	11.1	107 -	121	0	13	R.VAAICFGENMNTYDK.K + Oxidation (M)
2682.1808	2681.1736	2681.1374	13.5	77 -	99	0	120	K.LSTYSYSFQNGDIVCGDDDPCLR.A

No match to: 817.1353, 831.0894, 837.0987, 839.1181, 852.3661, 855.0858, 859.0803, 865.5262, 868.5601, 871.0599, 874.0343, 896.0194, 903.4049, 936.2407, 952.4442, 1011.4809, 1022.1437, 1022.4987, 1044.1191, 1049.4942, 1064.0866, 1066.1056, 1068.5038, 1082.0794, 1113.6442, 1149.4939, 1157.6529, 1161.6449, 1173.6476, 1189.6416, 1193.6342, 1195.5987, 1205.6345, 1221.6251, 1234.6887, 1238.6385, 1249.1265, 1255.6430, 1267.6252, 1271.1047, 1283.6191, 1285.7483, 1305.5768, 1311.7646, 1323.6353, 1330.5501, 1339.6298, 1343.5209, 1346.5381, 1355.6208, 1361.5885, 1368.4953, 1372.5633, 1377.5715, 1393.5665, 1395.7189, 1411.7132, 1433.6712, 1476.1124, 1497.7909, 1625.8814, 1636.7437, 1674.6785, 1698.8155, 1718.7743, 1731.7859, 1733.7804, 1736.7580, 1740.7770, 1754.7753, 1756.7402, 1764.8295, 1769.7342, 1771.7337, 1941.8846, 1967.8497, 2005.7944, 2118.7803, 2134.9720, 2217.9430, 2234.8390, 2566.1228, 2618.1553, 2636.1695, 2663.1677, 2708.1954, 2720.1183, 2724.1914, 3347.6860

5. PA2A7_DABPA **Mass:** 16190 **Score:** 157 **Expect:** 2.8e-013 **Matches:** 4

Acidic phospholipase A2 VP7 OS=Daboia palaestinae OX=1170828 PE=3 SV=1

Observed	Mr(expt)	Mr(calc)	ppm	Start	End	Miss	Ions	Peptide
909.3860	908.3787	908.3480	33.8	100 -	106	0	6	R.AVCECDR.V
987.5164	986.5092	986.4781	31.5	50 -	58	0	4	K.GKPQDATDR.C
2682.1808	2681.1736	2681.1374	13.5	77 -	99	0	120	K.LSTYSYSFQNGDIVCGDDDPCLR.A
2724.1914	2723.1841	2723.1448	14.4	100 -	122	1	---	R.AVCECDRVAAICFGENMNTYDTK.Y

No match to: 817.1353, 831.0894, 837.0987, 839.1181, 852.3661, 855.0858, 859.0803, 865.5262, 868.5601, 871.0599, 874.0343, 896.0194, 903.4049, 936.2407, 952.4442, 1011.4809, 1022.1437, 1022.4987, 1044.1191, 1049.4942, 1064.0866, 1066.1056, 1068.5038, 1082.0794, 1113.6442, 1149.4939, 1157.6529, 1161.6449, 1173.6476, 1189.6416, 1193.6342, 1195.5987, 1205.6345, 1221.6251, 1234.6887, 1238.6385, 1249.1265, 1255.6430, 1267.6252, 1271.1047, 1283.6191, 1285.7483, 1305.5768, 1311.7646, 1323.6353, 1330.5501, 1339.6298, 1343.5209, 1346.5381, 1355.6208, 1361.5885, 1368.4953, 1372.5633, 1377.5715, 1393.5665, 1395.7189, 1411.7132, 1433.6712, 1476.1124, 1497.7909, 1625.8814, 1636.7437, 1674.6785, 1698.8155, 1718.7743, 1731.7859, 1733.7804, 1736.7580, 1740.7770, 1748.7813, 1754.7753, 1756.7402, 1764.8295, 1769.7342, 1771.7337, 1941.8846, 1967.8497, 2005.7944, 2118.7803, 2134.9720, 2217.9430, 2234.8390, 2566.1228, 2618.1553, 2636.1695, 2663.1677, 2708.1954, 2720.1183, 3347.6860

6. Q6A3G8_VIPAA **Mass:** 15517 **Score:** 87 **Expect:** 3e-006 **Matches:** 4

Ammodytin I1(A) variant OS=Vipera ammodytes ammodytes OX=8705 PE=2 SV=1

Observed	Mr(expt)	Mr(calc)	ppm	Start	End	Miss	Ions	Peptide
909.3860	908.3787	908.3480	33.8	100 -	106	0	6	R.AVCECDR.V
1339.6298	1338.6225	1338.5988	17.7	123 -	132	0	52	K.YMLYSLFDCK.-
1355.6208	1354.6135	1354.5937	14.6	123 -	132	0	(2)	K.YMLYSLFDCK.- + Oxidation (M)
1748.7813	1747.7740	1747.7545	11.1	107 -	121	0	13	R.VAAICFGENMNTYDK.K + Oxidation (M)

No match to: 817.1353, 831.0894, 837.0987, 839.1181, 852.3661, 855.0858, 859.0803, 865.5262, 868.5601, 871.0599, 874.0343, 896.0194, 903.4049, 936.2407, 952.4442, 987.5164, 1011.4809, 1022.1437, 1022.4987, 1044.1191, 1049.4942, 1064.0866, 1066.1056, 1068.5038, 1082.0794, 1113.6442, 1149.4939, 1157.6529, 1161.6449, 1173.6476, 1189.6416, 1193.6342, 1195.5987, 1205.6345, 1221.6251, 1234.6887, 1238.6385, 1249.1265, 1255.6430, 1267.6252, 1271.1047, 1283.6191, 1285.7483, 1305.5768, 1311.7646, 1323.6353, 1330.5501, 1343.5209, 1346.5381, 1361.5885, 1368.4953, 1372.5633, 1377.5715, 1393.5665, 1395.7189, 1411.7132, 1433.6712, 1476.1124, 1497.7909, 1625.8814, 1636.7437, 1674.6785, 1698.8155, 1718.7743, 1731.7859, 1733.7804, 1736.7580, 1740.7770, 1754.7753, 1756.7402, 1764.8295, 1769.7342, 1771.7337, 1941.8846, 1967.8497, 2005.7944, 2118.7803, 2134.9720, 2217.9430, 2234.8390, 2566.1228, 2618.1553, 2636.1695, 2663.1677, 2682.1808, 2708.1954, 2720.1183, 2724.1914, 3347.6860

7. Q7T1D1_VIPBB **Mass:** 16258 **Score:** 86 **Expect:** 3.3e-006 **Matches:** 4

Ammodytin I1 OS=Vipera berus berus OX=31156 GN=AmtI1 PE=2 SV=1

Observed	Mr(expt)	Mr(calc)	ppm	Start	End	Miss	Ions	Peptide
909.3860	908.3787	908.3480	33.8	100 -	106	0	6	R.AVCECDR.V
1339.6298	1338.6225	1338.5988	17.7	123 -	132	0	52	K.YMLYSLFDCK.E
1355.6208	1354.6135	1354.5937	14.6	123 -	132	0	(2)	K.YMLYSLFDCK.E + Oxidation (M)
1748.7813	1747.7740	1747.7545	11.1	107 -	121	0	13	R.VAAICFGENMNTYDK.K + Oxidation (M)

No match to: 817.1353, 831.0894, 837.0987, 839.1181, 852.3661, 855.0858, 859.0803, 865.5262, 868.5601, 871.0599, 874.0343, 896.0194, 903.4049, 936.2407, 952.4442, 987.5164, 1011.4809, 1022.1437, 1022.4987, 1044.1191, 1049.4942, 1064.0866, 1066.1056, 1068.5038, 1082.0794, 1113.6442, 1149.4939, 1157.6529, 1161.6449, 1173.6476, 1189.6416, 1193.6342, 1195.5987, 1205.6345, 1221.6251, 1234.6887, 1238.6385, 1249.1265, 1255.6430, 1267.6252, 1271.1047, 1283.6191, 1285.7483, 1305.5768, 1311.7646, 1323.6353, 1330.5501, 1343.5209, 1346.5381, 1361.5885, 1368.4953, 1372.5633, 1377.5715, 1393.5665, 1395.7189, 1411.7132, 1433.6712, 1476.1124, 1497.7909, 1625.8814, 1636.7437, 1674.6785, 1698.8155, 1718.7743, 1731.7859, 1733.7804, 1736.7580, 1740.7770, 1754.7753, 1756.7402, 1764.8295, 1769.7342, 1771.7337, 1941.8846, 1967.8497, 2005.7944, 2118.7803, 2134.9720, 2217.9430, 2234.8390, 2566.1228, 2618.1553, 2636.1695, 2663.1677, 2682.1808, 2708.1954, 2720.1183, 2724.1914, 3347.6860

8. Q6A3G4_VIPAM **Mass:** 16280 **Score:** 86 **Expect:** 3.3e-006 **Matches:** 4

Ammodytin I1(D) isoform OS=Vipera ammodytes montandoni OX=235554 PE=2 SV=1

Observed	Mr(expt)	Mr(calc)	ppm	Start	End	Miss	Ions	Peptide
909.3860	908.3787	908.3480	33.8	100 -	106	0	6	R.AVCECDR.V
1339.6298	1338.6225	1338.5988	17.7	123 -	132	0	52	K.YMLYSLFDCK.E
1355.6208	1354.6135	1354.5937	14.6	123 -	132	0	(2)	K.YMLYSLFDCK.E + Oxidation (M)
1748.7813	1747.7740	1747.7545	11.1	107 -	121	0	13	R.VAAICFGENMNTYDK.K + Oxidation (M)

No match to: 817.1353, 831.0894, 837.0987, 839.1181, 852.3661, 855.0858, 859.0803, 865.5262, 868.5601, 871.0599, 874.0343, 896.0194, 903.4049, 936.2407, 952.4442, 987.5164, 1011.4809, 1022.1437, 1022.4987, 1044.1191, 1049.4942, 1064.0866, 1066.1056, 1068.5038, 1082.0794, 1113.6442, 1149.4939, 1157.6529, 1161.6449, 1173.6476, 1189.6416, 1193.6342, 1195.5987, 1205.6345, 1221.6251, 1234.6887, 1238.6385, 1249.1265, 1255.6430, 1267.6252, 1271.1047, 1283.6191, 1285.7483, 1305.5768, 1311.7646, 1323.6353, 1330.5501, 1343.5209, 1346.5381, 1361.5885, 1368.4953, 1372.5633, 1377.5715, 1393.5665, 1395.7189, 1411.7132, 1433.6712, 1476.1124, 1497.7909, 1625.8814, 1636.7437, 1674.6785, 1698.8155, 1718.7743, 1731.7859, 1733.7804, 1736.7580, 1740.7770, 1754.7753, 1756.7402, 1764.8295, 1769.7342, 1771.7337, 1941.8846, 1967.8497, 2005.7944, 2118.7803, 2134.9720, 2217.9430, 2234.8390, 2566.1228, 2618.1553, 2636.1695, 2663.1677, 2682.1808, 2708.1954, 2720.1183, 2724.1914, 3347.6860

9. Q6A3N0_VIPAM **Mass:** 16279 **Score:** 86 **Expect:** 3.3e-006 **Matches:** 4

Ammodytin I1(A) isoform OS=Vipera ammodytes ruffoi OX=246549 PE=2 SV=1

Observed	Mr(expt)	Mr(calc)	ppm	Start	End	Miss	Ions	Peptide
909.3860	908.3787	908.3480	33.8	100 -	106	0	6	R.AVCECDR.V
1339.6298	1338.6225	1338.5988	17.7	123 -	132	0	52	K.YMLYSLFDCK.E
1355.6208	1354.6135	1354.5937	14.6	123 -	132	0	(2)	K.YMLYSLFDCK.E + Oxidation (M)
1748.7813	1747.7740	1747.7545	11.1	107 -	121	0	13	R.VAAICFGENMNTYDK.K + Oxidation (M)

No match to: 817.1353, 831.0894, 837.0987, 839.1181, 852.3661, 855.0858, 859.0803, 865.5262, 868.5601, 871.0599, 874.0343, 896.0194, 903.4049, 936.2407, 952.4442, 987.5164, 1011.4809, 1022.1437, 1022.4987, 1044.1191, 1049.4942, 1064.0866, 1066.1056, 1068.5038, 1082.0794, 1113.6442, 1149.4939, 1157.6529, 1161.6449, 1173.6476, 1189.6416, 1193.6342, 1195.5987, 1205.6345, 1221.6251, 1234.6887, 1238.6385, 1249.1265, 1255.6430, 1267.6252, 1271.1047, 1283.6191, 1285.7483, 1305.5768, 1311.7646, 1323.6353, 1330.5501, 1343.5209, 1346.5381, 1361.5885, 1368.4953, 1372.5633, 1377.5715, 1393.5665, 1395.7189, 1411.7132, 1433.6712, 1476.1124, 1497.7909, 1625.8814, 1636.7437, 1674.6785, 1698.8155, 1718.7743, 1731.7859, 1733.7804, 1736.7580, 1740.7770, 1754.7753, 1756.7402, 1764.8295, 1769.7342, 1771.7337, 1941.8846, 1967.8497, 2005.7944, 2118.7803, 2134.9720, 2217.9430, 2234.8390, 2566.1228, 2618.1553, 2636.1695, 2663.1677, 2682.1808, 2708.1954, 2720.1183, 2724.1914, 3347.6860

10. Q6A3K0_VIPBB **Mass:** 16214 **Score:** 86 **Expect:** 3.3e-006 **Matches:** 4

Ammodytin I1(B') variant OS=Vipera berus berus OX=31156 PE=2 SV=1

Observed	Mr(expt)	Mr(calc)	ppm	Start	End	Miss	Ions	Peptide
909.3860	908.3787	908.3480	33.8	100 -	106	0	6	R.AVCECDR.V
1339.6298	1338.6225	1338.5988	17.7	123 -	132	0	52	K.YMLYSLFDCK.E
1355.6208	1354.6135	1354.5937	14.6	123 -	132	0	(2)	K.YMLYSLFDCK.E + Oxidation (M)
1748.7813	1747.7740	1747.7545	11.1	107 -	121	0	13	R.VAAICFGENMNTYDK.K + Oxidation (M)

No match to: 817.1353, 831.0894, 837.0987, 839.1181, 852.3661, 855.0858, 859.0803, 865.5262, 868.5601, 871.0599, 874.0343, 896.0194, 903.4049, 936.2407, 952.4442, 987.5164, 1011.4809, 1022.1437, 1022.4987, 1044.1191, 1049.4942, 1064.0866, 1066.1056, 1068.5038,

1082.0794, 1113.6442, 1149.4939, 1157.6529, 1161.6449, 1173.6476, 1189.6416, 1193.6342, 1195.5987, 1205.6345, 1221.6251, 1234.6887, 1238.6385, 1249.1265, 1255.6430, 1267.6252, 1271.1047, 1283.6191, 1285.7483, 1305.5768, 1311.7646, 1323.6353, 1330.5501, 1343.5209, 1346.5381, 1361.5885, 1368.4953, 1372.5633, 1377.5715, 1393.5665, 1395.7189, 1411.7132, 1433.6712, 1476.1124, 1497.7909, 1625.8814, 1636.7437, 1674.6785, 1698.8155, 1718.7743, 1731.7859, 1733.7804, 1736.7580, 1740.7770, 1754.7753, 1756.7402, 1764.8295, 1769.7342, 1771.7337, 1941.8846, 1967.8497, 2005.7944, 2118.7803, 2134.9720, 2217.9430, 2234.8390, 2566.1228, 2618.1553, 2636.1695, 2663.1677, 2682.1808, 2708.1954, 2720.1183, 2724.1914, 3347.6860

11. Q6A3G5_VIPAE **Mass:** 16280 **Score:** 86 **Expect:** 3.3e-006 **Matches:** 4

Ammodytin I1(D) isoform OS=Vipera ammodytes meridionalis OX=73841 PE=2 SV=1

Observed	Mr(expt)	Mr(calc)	ppm	Start	End	Miss	Ions	Peptide
909.3860	908.3787	908.3480	33.8	100	106	0	6	R.AVCECDR.V
1339.6298	1338.6225	1338.5988	17.7	123	132	0	52	K.YMLYSLFDCK.E
1355.6208	1354.6135	1354.5937	14.6	123	132	0	(2)	K.YMLYSLFDCK.E + Oxidation (M)
1748.7813	1747.7740	1747.7545	11.1	107	121	0	13	R.VAAICFGENMNTYDK.K + Oxidation (M)

No match to: 817.1353, 831.0894, 837.0987, 839.1181, 852.3661, 855.0858, 859.0803, 865.5262, 868.5601, 871.0599, 874.0343, 896.0194, 903.4049, 936.2407, 952.4442, 987.5164, 1011.4809, 1022.1437, 1022.4987, 1044.1191, 1049.4942, 1064.0866, 1066.1056, 1068.5038, 1082.0794, 1113.6442, 1149.4939, 1157.6529, 1161.6449, 1173.6476, 1189.6416, 1193.6342, 1195.5987, 1205.6345, 1221.6251, 1234.6887, 1238.6385, 1249.1265, 1255.6430, 1267.6252, 1271.1047, 1283.6191, 1285.7483, 1305.5768, 1311.7646, 1323.6353, 1330.5501, 1343.5209, 1346.5381, 1361.5885, 1368.4953, 1372.5633, 1377.5715, 1393.5665, 1395.7189, 1411.7132, 1433.6712, 1476.1124, 1497.7909, 1625.8814, 1636.7437, 1674.6785, 1698.8155, 1718.7743, 1731.7859, 1733.7804, 1736.7580, 1740.7770, 1754.7753, 1756.7402, 1764.8295, 1769.7342, 1771.7337, 1941.8846, 1967.8497, 2005.7944, 2118.7803, 2134.9720, 2217.9430, 2234.8390, 2566.1228, 2618.1553, 2636.1695, 2663.1677, 2682.1808, 2708.1954, 2720.1183, 2724.1914, 3347.6860

12. Q1RP80_VIPBN **Mass:** 16258 **Score:** 86 **Expect:** 3.3e-006 **Matches:** 4

Phospholipase A2 ammodytin I1 OS=Vipera berus nikolskii OX=1808362 GN=I1 PE=2 SV=1

Observed	Mr(expt)	Mr(calc)	ppm	Start	End	Miss	Ions	Peptide
909.3860	908.3787	908.3480	33.8	100	106	0	6	R.AVCECDR.V
1339.6298	1338.6225	1338.5988	17.7	123	132	0	52	K.YMLYSLFDCK.E
1355.6208	1354.6135	1354.5937	14.6	123	132	0	(2)	K.YMLYSLFDCK.E + Oxidation (M)
1748.7813	1747.7740	1747.7545	11.1	107	121	0	13	R.VAAICFGENMNTYDK.K + Oxidation (M)

No match to: 817.1353, 831.0894, 837.0987, 839.1181, 852.3661, 855.0858, 859.0803, 865.5262, 868.5601, 871.0599, 874.0343, 896.0194, 903.4049, 936.2407, 952.4442, 987.5164, 1011.4809, 1022.1437, 1022.4987, 1044.1191, 1049.4942, 1064.0866, 1066.1056, 1068.5038, 1082.0794, 1113.6442, 1149.4939, 1157.6529, 1161.6449, 1173.6476, 1189.6416, 1193.6342, 1195.5987, 1205.6345, 1221.6251, 1234.6887, 1238.6385, 1249.1265, 1255.6430, 1267.6252, 1271.1047, 1283.6191, 1285.7483, 1305.5768, 1311.7646, 1323.6353, 1330.5501, 1343.5209, 1346.5381, 1361.5885, 1368.4953, 1372.5633, 1377.5715, 1393.5665, 1395.7189, 1411.7132, 1433.6712, 1476.1124, 1497.7909, 1625.8814, 1636.7437, 1674.6785, 1698.8155, 1718.7743, 1731.7859, 1733.7804, 1736.7580, 1740.7770, 1754.7753, 1756.7402, 1764.8295, 1769.7342, 1771.7337, 1941.8846, 1967.8497, 2005.7944, 2118.7803, 2134.9720, 2217.9430, 2234.8390, 2566.1228, 2618.1553, 2636.1695, 2663.1677, 2682.1808, 2708.1954, 2720.1183, 2724.1914, 3347.6860

13. Q6YC90_VIPAS **Mass:** 16279 **Score:** 86 **Expect:** 3.3e-006 **Matches:** 4

Ammodytin I1 isoform 2 OS=Vipera aspis OX=8706 GN=AmtI1 PE=3 SV=1

Observed	Mr(expt)	Mr(calc)	ppm	Start	End	Miss	Ions	Peptide
909.3860	908.3787	908.3480	33.8	100	106	0	6	R.AVCECDR.V
1339.6298	1338.6225	1338.5988	17.7	123	132	0	52	K.YMLYSLFDCK.E
1355.6208	1354.6135	1354.5937	14.6	123	132	0	(2)	K.YMLYSLFDCK.E + Oxidation (M)
1748.7813	1747.7740	1747.7545	11.1	107	121	0	13	R.VAAICFGENMNTYDK.K + Oxidation (M)

No match to: 817.1353, 831.0894, 837.0987, 839.1181, 852.3661, 855.0858, 859.0803, 865.5262, 868.5601, 871.0599, 874.0343, 896.0194, 903.4049, 936.2407, 952.4442, 987.5164, 1011.4809, 1022.1437, 1022.4987, 1044.1191, 1049.4942, 1064.0866, 1066.1056, 1068.5038, 1082.0794, 1113.6442, 1149.4939, 1157.6529, 1161.6449, 1173.6476, 1189.6416, 1193.6342, 1195.5987, 1205.6345, 1221.6251, 1234.6887, 1238.6385, 1249.1265, 1255.6430, 1267.6252, 1271.1047, 1283.6191, 1285.7483, 1305.5768, 1311.7646, 1323.6353, 1330.5501, 1343.5209, 1346.5381, 1361.5885, 1368.4953, 1372.5633, 1377.5715, 1393.5665, 1395.7189, 1411.7132, 1433.6712, 1476.1124, 1497.7909, 1625.8814, 1636.7437, 1674.6785, 1698.8155, 1718.7743, 1731.7859, 1733.7804, 1736.7580, 1740.7770, 1754.7753, 1756.7402, 1764.8295, 1769.7342, 1771.7337, 1941.8846, 1967.8497, 2005.7944, 2118.7803, 2134.9720, 2217.9430, 2234.8390, 2566.1228, 2618.1553, 2636.1695, 2663.1677, 2682.1808, 2708.1954, 2720.1183, 2724.1914, 3347.6860

14. Q6A3I8_VIPUR **Mass:** 16290 **Score:** 86 **Expect:** 3.3e-006 **Matches:** 4

Ammodytin I1(B) isoform OS=Vipera ursinii OX=103942 PE=2 SV=1

Observed	Mr(expt)	Mr(calc)	ppm	Start	End	Miss	Ions	Peptide
909.3860	908.3787	908.3480	33.8	100	106	0	6	R.AVCECDR.V
1339.6298	1338.6225	1338.5988	17.7	123	132	0	52	K.YMLYSLFDCK.E
1355.6208	1354.6135	1354.5937	14.6	123	132	0	(2)	K.YMLYSLFDCK.E + Oxidation (M)
1748.7813	1747.7740	1747.7545	11.1	107	121	0	13	R.VAAICFGENMNTYDK.K + Oxidation (M)

No match to: 817.1353, 831.0894, 837.0987, 839.1181, 852.3661, 855.0858, 859.0803, 865.5262, 868.5601, 871.0599, 874.0343, 896.0194, 903.4049, 936.2407, 952.4442, 987.5164, 1011.4809, 1022.1437, 1022.4987, 1044.1191, 1049.4942, 1064.0866, 1066.1056, 1068.5038, 1082.0794, 1113.6442, 1149.4939, 1157.6529, 1161.6449, 1173.6476, 1189.6416, 1193.6342, 1195.5987, 1205.6345, 1221.6251, 1234.6887, 1238.6385, 1249.1265, 1255.6430, 1267.6252, 1271.1047, 1283.6191, 1285.7483, 1305.5768, 1311.7646, 1323.6353, 1330.5501, 1343.5209, 1346.5381, 1361.5885, 1368.4953, 1372.5633, 1377.5715, 1393.5665, 1395.7189, 1411.7132, 1433.6712, 1476.1124, 1497.7909, 1625.8814, 1636.7437, 1674.6785, 1698.8155, 1718.7743, 1731.7859, 1733.7804, 1736.7580, 1740.7770, 1754.7753, 1756.7402, 1764.8295, 1769.7342, 1771.7337, 1941.8846, 1967.8497, 2005.7944, 2118.7803, 2134.9720, 2217.9430, 2234.8390, 2566.1228, 2618.1553, 2636.1695, 2663.1677, 2682.1808, 2708.1954, 2720.1183, 2724.1914, 3347.6860

15. Q6A3K8_VIPAP **Mass:** 16267 **Score:** 86 **Expect:** 3.3e-006 **Matches:** 4

Ammodytin I1(A') isoform OS=Vipera aspis aspis OX=194601 PE=2 SV=1

Observed	Mr(expt)	Mr(calc)	ppm	Start	End	Miss	Ions	Peptide
909.3860	908.3787	908.3480	33.8	100	106	0	6	R.AVCECDR.V
1339.6298	1338.6225	1338.5988	17.7	123	132	0	52	K.YMLYSLFDCK.E
1355.6208	1354.6135	1354.5937	14.6	123	132	0	(2)	K.YMLYSLFDCK.E + Oxidation (M)
1748.7813	1747.7740	1747.7545	11.1	107	121	0	13	R.VAAICFGENMNTYDK.K + Oxidation (M)

No match to: 817.1353, 831.0894, 837.0987, 839.1181, 852.3661, 855.0858, 859.0803, 865.5262, 868.5601, 871.0599, 874.0343, 896.0194, 903.4049, 936.2407, 952.4442, 987.5164, 1011.4809, 1022.1437, 1022.4987, 1044.1191, 1049.4942, 1064.0866, 1066.1056, 1068.5038, 1082.0794, 1113.6442, 1149.4939, 1157.6529, 1161.6449, 1173.6476, 1189.6416, 1193.6342, 1195.5987, 1205.6345, 1221.6251, 1234.6887, 1238.6385, 1249.1265, 1255.6430, 1267.6252, 1271.1047, 1283.6191, 1285.7483, 1305.5768, 1311.7646, 1323.6353, 1330.5501, 1343.5209, 1346.5381, 1361.5885, 1368.4953, 1372.5633, 1377.5715, 1393.5665, 1395.7189, 1411.7132, 1433.6712, 1476.1124, 1497.7909, 1625.8814, 1636.7437, 1674.6785, 1698.8155, 1718.7743, 1731.7859, 1733.7804, 1736.7580, 1740.7770, 1754.7753, 1756.7402, 1764.8295, 1769.7342, 1771.7337, 1941.8846, 1967.8497, 2005.7944, 2118.7803, 2134.9720, 2217.9430, 2234.8390, 2566.1228, 2618.1553, 2636.1695, 2663.1677, 2682.1808, 2708.1954, 2720.1183, 2724.1914, 3347.6860

16. Q6A3K4_VIPAP **Mass:** 16252 **Score:** 86 **Expect:** 3.3e-006 **Matches:** 4

Ammodytin I1(A) variant OS=Vipera aspis aspis OX=194601 PE=2 SV=1

Observed	Mr(expt)	Mr(calc)	ppm	Start	End	Miss	Ions	Peptide
909.3860	908.3787	908.3480	33.8	100	106	0	6	R.AVCECDR.V
1339.6298	1338.6225	1338.5988	17.7	123	132	0	52	K.YMLYSLFDCK.E
1355.6208	1354.6135	1354.5937	14.6	123	132	0	(2)	K.YMLYSLFDCK.E + Oxidation (M)
1748.7813	1747.7740	1747.7545	11.1	107	121	0	13	R.VAAICFGENMNTYDK.K + Oxidation (M)

No match to: 817.1353, 831.0894, 837.0987, 839.1181, 852.3661, 855.0858, 859.0803, 865.5262, 868.5601, 871.0599, 874.0343, 896.0194, 903.4049, 936.2407, 952.4442, 987.5164, 1011.4809, 1022.1437, 1022.4987, 1044.1191, 1049.4942, 1064.0866, 1066.1056, 1068.5038, 1082.0794, 1113.6442, 1149.4939, 1157.6529, 1161.6449, 1173.6476, 1189.6416, 1193.6342, 1195.5987, 1205.6345, 1221.6251, 1234.6887, 1238.6385, 1249.1265, 1255.6430, 1267.6252, 1271.1047, 1283.6191, 1285.7483, 1305.5768, 1311.7646, 1323.6353, 1330.5501, 1343.5209,

1346.5381, 1361.5885, 1368.4953, 1372.5633, 1377.5715, 1393.5665, 1395.7189, 1411.7132, 1433.6712, 1476.1124, 1497.7909, 1625.8814, 1636.7437, 1674.6785, 1698.8155, 1718.7743, 1731.7859, 1733.7804, 1736.7580, 1740.7770, 1754.7753, 1756.7402, 1764.8295, 1769.7342, 1771.7337, 1941.8846, 1967.8497, 2005.7944, 2118.7803, 2134.9720, 2217.9430, 2234.8390, 2566.1228, 2618.1553, 2636.1695, 2663.1677, 2682.1808, 2708.1954, 2720.1183, 2724.1914, 3347.6860

17. Q6A3K7_VIPAP **Mass:** 16279 **Score:** 86 **Expect:** 3.3e-006 **Matches:** 4

Ammodytin I1(A) isoform OS=Vipera aspis aspis OX=194601 PE=2 SV=1

Observed	Mr(expt)	Mr(calc)	ppm	Start	End	Miss	Ions	Peptide
909.3860	908.3787	908.3480	33.8	100	106	0	6	R.AVCECDR.V
1339.6298	1338.6225	1338.5988	17.7	123	132	0	52	K.YMLYSLFDCK.E
1355.6208	1354.6135	1354.5937	14.6	123	132	0	(2)	K.YMLYSLFDCK.E + Oxidation (M)
1748.7813	1747.7740	1747.7545	11.1	107	121	0	13	R.VAAICFGENMNTYDK.K + Oxidation (M)

No match to: 817.1353, 831.0894, 837.0987, 839.1181, 852.3661, 855.0858, 859.0803, 865.5262, 868.5601, 871.0599, 874.0343, 896.0194, 903.4049, 936.2407, 952.4442, 987.5164, 1011.4809, 1022.1437, 1022.4987, 1044.1191, 1049.4942, 1064.0866, 1066.1056, 1068.5038, 1082.0794, 1113.6442, 1149.4939, 1157.6529, 1161.6449, 1173.6476, 1189.6416, 1193.6342, 1195.5987, 1205.6345, 1221.6251, 1234.6887, 1238.6385, 1249.1265, 1255.6430, 1267.6252, 1271.1047, 1283.6191, 1285.7483, 1305.5768, 1311.7646, 1323.6353, 1330.5501, 1343.5209, 1346.5381, 1361.5885, 1368.4953, 1372.5633, 1377.5715, 1393.5665, 1395.7189, 1411.7132, 1433.6712, 1476.1124, 1497.7909, 1625.8814, 1636.7437, 1674.6785, 1698.8155, 1718.7743, 1731.7859, 1733.7804, 1736.7580, 1740.7770, 1754.7753, 1756.7402, 1764.8295, 1769.7342, 1771.7337, 1941.8846, 1967.8497, 2005.7944, 2118.7803, 2134.9720, 2217.9430, 2234.8390, 2566.1228, 2618.1553, 2636.1695, 2663.1677, 2682.1808, 2708.1954, 2720.1183, 2724.1914, 3347.6860

18. Q6A3M8_VIPAM **Mass:** 16378 **Score:** 86 **Expect:** 3.3e-006 **Matches:** 4

Ammodytin I1(A) variant OS=Vipera ammodytes ruffoi OX=246549 PE=2 SV=1

Observed	Mr(expt)	Mr(calc)	ppm	Start	End	Miss	Ions	Peptide
909.3860	908.3787	908.3480	33.8	100	106	0	6	R.AVCECDR.V
1339.6298	1338.6225	1338.5988	17.7	123	132	0	52	K.YMLYSLFDCK.E
1355.6208	1354.6135	1354.5937	14.6	123	132	0	(2)	K.YMLYSLFDCK.E + Oxidation (M)
1748.7813	1747.7740	1747.7545	11.1	107	121	0	13	R.VAAICFGENMNTYDK.K + Oxidation (M)

No match to: 817.1353, 831.0894, 837.0987, 839.1181, 852.3661, 855.0858, 859.0803, 865.5262, 868.5601, 871.0599, 874.0343, 896.0194, 903.4049, 936.2407, 952.4442, 987.5164, 1011.4809, 1022.1437, 1022.4987, 1044.1191, 1049.4942, 1064.0866, 1066.1056, 1068.5038, 1082.0794, 1113.6442, 1149.4939, 1157.6529, 1161.6449, 1173.6476, 1189.6416, 1193.6342, 1195.5987, 1205.6345, 1221.6251, 1234.6887, 1238.6385, 1249.1265, 1255.6430, 1267.6252, 1271.1047, 1283.6191, 1285.7483, 1305.5768, 1311.7646, 1323.6353, 1330.5501, 1343.5209, 1346.5381, 1361.5885, 1368.4953, 1372.5633, 1377.5715, 1393.5665, 1395.7189, 1411.7132, 1433.6712, 1476.1124, 1497.7909, 1625.8814, 1636.7437, 1674.6785, 1698.8155, 1718.7743, 1731.7859, 1733.7804, 1736.7580, 1740.7770, 1754.7753, 1756.7402, 1764.8295, 1769.7342, 1771.7337, 1941.8846, 1967.8497, 2005.7944, 2118.7803, 2134.9720, 2217.9430, 2234.8390, 2566.1228, 2618.1553, 2636.1695, 2663.1677, 2682.1808, 2708.1954, 2720.1183, 2724.1914, 3347.6860

19. Q6A3M9_VIPAM **Mass:** 16286 **Score:** 86 **Expect:** 3.3e-006 **Matches:** 4

Ammodytin I1(B) isoform OS=Vipera ammodytes ruffoi OX=246549 PE=2 SV=1

Observed	Mr(expt)	Mr(calc)	ppm	Start	End	Miss	Ions	Peptide
909.3860	908.3787	908.3480	33.8	100	106	0	6	R.AVCECDR.V
1339.6298	1338.6225	1338.5988	17.7	123	132	0	52	K.YMLYSLFDCK.E
1355.6208	1354.6135	1354.5937	14.6	123	132	0	(2)	K.YMLYSLFDCK.E + Oxidation (M)
1748.7813	1747.7740	1747.7545	11.1	107	121	0	13	R.VAAICFGENMNTYDK.K + Oxidation (M)

No match to: 817.1353, 831.0894, 837.0987, 839.1181, 852.3661, 855.0858, 859.0803, 865.5262, 868.5601, 871.0599, 874.0343, 896.0194, 903.4049, 936.2407, 952.4442, 987.5164, 1011.4809, 1022.1437, 1022.4987, 1044.1191, 1049.4942, 1064.0866, 1066.1056, 1068.5038, 1082.0794, 1113.6442, 1149.4939, 1157.6529, 1161.6449, 1173.6476, 1189.6416, 1193.6342, 1195.5987, 1205.6345, 1221.6251, 1234.6887, 1238.6385, 1249.1265, 1255.6430, 1267.6252, 1271.1047, 1283.6191, 1285.7483, 1305.5768, 1311.7646, 1323.6353, 1330.5501, 1343.5209, 1346.5381, 1361.5885, 1368.4953, 1372.5633, 1377.5715, 1393.5665, 1395.7189, 1411.7132, 1433.6712, 1476.1124, 1497.7909, 1625.8814, 1636.7437, 1674.6785, 1698.8155, 1718.7743, 1731.7859, 1733.7804, 1736.7580, 1740.7770, 1754.7753, 1756.7402, 1764.8295, 1769.7342, 1771.7337, 1941.8846, 1967.8497, 2005.7944, 2118.7803, 2134.9720, 2217.9430, 2234.8390, 2566.1228, 2618.1553, 2636.1695, 2663.1677, 2682.1808, 2708.1954, 2720.1183, 2724.1914, 3347.6860

20. PA2A1_VIPAA **Mass:** 16279 **Score:** 86 **Expect:** 3.3e-006 **Matches:** 4

Acidic phospholipase A2 ammodytin I1 OS=Vipera ammodytes ammodytes OX=8705 PE=2 SV=1

Observed	Mr(expt)	Mr(calc)	ppm	Start	End	Miss	Ions	Peptide
909.3860	908.3787	908.3480	33.8	100	106	0	6	R.AVCECDR.V
1339.6298	1338.6225	1338.5988	17.7	123	132	0	52	K.YMLYSLFDCK.E
1355.6208	1354.6135	1354.5937	14.6	123	132	0	(2)	K.YMLYSLFDCK.E + Oxidation (M)
1748.7813	1747.7740	1747.7545	11.1	107	121	0	13	R.VAAICFGENMNTYDK.K + Oxidation (M)

No match to: 817.1353, 831.0894, 837.0987, 839.1181, 852.3661, 855.0858, 859.0803, 865.5262, 868.5601, 871.0599, 874.0343, 896.0194, 903.4049, 936.2407, 952.4442, 987.5164, 1011.4809, 1022.1437, 1022.4987, 1044.1191, 1049.4942, 1064.0866, 1066.1056, 1068.5038, 1082.0794, 1113.6442, 1149.4939, 1157.6529, 1161.6449, 1173.6476, 1189.6416, 1193.6342, 1195.5987, 1205.6345, 1221.6251, 1234.6887, 1238.6385, 1249.1265, 1255.6430, 1267.6252, 1271.1047, 1283.6191, 1285.7483, 1305.5768, 1311.7646, 1323.6353, 1330.5501, 1343.5209, 1346.5381, 1361.5885, 1368.4953, 1372.5633, 1377.5715, 1393.5665, 1395.7189, 1411.7132, 1433.6712, 1476.1124, 1497.7909, 1625.8814, 1636.7437, 1674.6785, 1698.8155, 1718.7743, 1731.7859, 1733.7804, 1736.7580, 1740.7770, 1754.7753, 1756.7402, 1764.8295, 1769.7342, 1771.7337, 1941.8846, 1967.8497, 2005.7944, 2118.7803, 2134.9720, 2217.9430, 2234.8390, 2566.1228, 2618.1553, 2636.1695, 2663.1677, 2682.1808, 2708.1954, 2720.1183, 2724.1914, 3347.6860

21. PA2A2_DABRR **Mass:** 16430 **Score:** 77 **Expect:** 2.9e-005 **Matches:** 4

Acidic phospholipase A2 Drk-a2 OS=Daboia russelii OX=8707 PE=2 SV=1

Observed	Mr(expt)	Mr(calc)	ppm	Start	End	Miss	Ions	Peptide
909.3860	908.3787	908.3480	33.8	100	106	0	6	R.AVCECDR.V
987.5164	986.5092	986.4781	31.5	50	58	0	4	K.GKPQDATDR.C
1339.6298	1338.6225	1338.5988	17.7	123	132	0	52	K.YMLYSIFDCK.E
1355.6208	1354.6135	1354.5937	14.6	123	132	0	(2)	K.YMLYSIFDCK.E + Oxidation (M)

No match to: 817.1353, 831.0894, 837.0987, 839.1181, 852.3661, 855.0858, 859.0803, 865.5262, 868.5601, 871.0599, 874.0343, 896.0194, 903.4049, 936.2407, 952.4442, 1011.4809, 1022.1437, 1022.4987, 1044.1191, 1049.4942, 1064.0866, 1066.1056, 1068.5038, 1082.0794, 1113.6442, 1149.4939, 1157.6529, 1161.6449, 1173.6476, 1189.6416, 1193.6342, 1195.5987, 1205.6345, 1221.6251, 1234.6887, 1238.6385, 1249.1265, 1255.6430, 1267.6252, 1271.1047, 1283.6191, 1285.7483, 1305.5768, 1311.7646, 1323.6353, 1330.5501, 1343.5209, 1346.5381, 1361.5885, 1368.4953, 1372.5633, 1377.5715, 1393.5665, 1395.7189, 1411.7132, 1433.6712, 1476.1124, 1497.7909, 1625.8814, 1636.7437, 1674.6785, 1698.8155, 1718.7743, 1731.7859, 1733.7804, 1736.7580, 1740.7770, 1748.7813, 1754.7753, 1756.7402, 1764.8295, 1769.7342, 1771.7337, 1941.8846, 1967.8497, 2005.7944, 2118.7803, 2134.9720, 2217.9430, 2234.8390, 2566.1228, 2618.1553, 2636.1695, 2663.1677, 2682.1808, 2708.1954, 2720.1183, 2724.1914, 3347.6860

22. PA2AB_DABSI **Mass:** 15673 **Score:** 74 **Expect:** 5.1e-005 **Matches:** 4

Acidic phospholipase A2 daboiatoxin B chain (Fragment) OS=Daboia siamensis OX=343250 PE=1 SV=1

Observed	Mr(expt)	Mr(calc)	ppm	Start	End	Miss	Ions	Peptide
909.3860	908.3787	908.3480	33.8	92	98	0	6	R.AVCECDR.V
1339.6298	1338.6225	1338.5988	17.7	115	124	0	52	K.YMLYSIFDCK.E
1355.6208	1354.6135	1354.5937	14.6	115	124	0	(2)	K.YMLYSIFDCK.E + Oxidation (M)
1754.7753	1753.7681	1753.8644	-54.92	1	15	0	---	-.MCLIGVEGNLFQFAR.L

No match to: 817.1353, 831.0894, 837.0987, 839.1181, 852.3661, 855.0858, 859.0803, 865.5262, 868.5601, 871.0599, 874.0343, 896.0194, 903.4049, 936.2407, 952.4442, 987.5164, 1011.4809, 1022.1437, 1022.4987, 1044.1191, 1049.4942, 1064.0866, 1066.1056, 1068.5038, 1082.0794, 1113.6442, 1149.4939, 1157.6529, 1161.6449, 1173.6476, 1189.6416, 1193.6342, 1195.5987, 1205.6345, 1221.6251, 1234.6887, 1238.6385, 1249.1265, 1255.6430, 1267.6252, 1271.1047, 1283.6191, 1285.7483, 1305.5768, 1311.7646, 1323.6353, 1330.5501, 1343.5209, 1346.5381, 1361.5885, 1368.4953, 1372.5633, 1377.5715, 1393.5665, 1395.7189, 1411.7132, 1433.6712, 1476.1124, 1497.7909, 1625.8814, 1636.7437, 1674.6785, 1698.8155, 1718.7743, 1731.7859, 1733.7804, 1736.7580, 1740.7770, 1748.7813, 1756.7402, 1764.8295, 1769.7342,

1771.7337, 1941.8846, 1967.8497, 2005.7944, 2118.7803, 2134.9720, 2217.9430, 2234.8390, 2566.1228, 2618.1553, 2636.1695, 2663.1677, 2682.1808, 2708.1954, 2720.1183, 2724.1914, 3347.6860

23. Q6A3K3_VIPAP **Mass:** 16307 **Score:** 67 **Expect:** 0.00031 **Matches:** 3

Ammodytin I1(A) variant OS=Vipera aspis aspis OX=194601 PE=2 SV=1

Observed	Mr(expt)	Mr(calc)	ppm	Start		End	Miss	Ions	Peptide
909.3860	908.3787	908.3480	33.8	100	-	106	0	6	R.AVCECDR.V
1339.6298	1338.6225	1338.5988	17.7	123	-	132	0	52	K.YMLYSLFDCK.E
1355.6208	1354.6135	1354.5937	14.6	123	-	132	0	(2)	K.YMLYSLFDCK.E + Oxidation (M)

No match to: 817.1353, 831.0894, 837.0987, 839.1181, 852.3661, 855.0858, 859.0803, 865.5262, 868.5601, 871.0599, 874.0343, 896.0194, 903.4049, 936.2407, 952.4442, 987.5164, 1011.4809, 1022.1437, 1022.4987, 1044.1191, 1049.4942, 1064.0866, 1066.1056, 1068.5038, 1082.0794, 1113.6442, 1149.4939, 1157.6529, 1161.6449, 1173.6476, 1189.6416, 1193.6342, 1195.5987, 1205.6345, 1221.6251, 1234.6887, 1238.6385, 1249.1265, 1255.6430, 1267.6252, 1271.1047, 1283.6191, 1285.7483, 1305.5768, 1311.7646, 1323.6353, 1330.5501, 1343.5209, 1346.5381, 1361.5885, 1368.4953, 1372.5633, 1377.5715, 1393.5665, 1395.7189, 1411.7132, 1433.6712, 1476.1124, 1497.7909, 1625.8814, 1636.7437, 1674.6785, 1698.8155, 1718.7743, 1731.7859, 1733.7804, 1736.7580, 1740.7770, 1748.7813, 1754.7753, 1756.7402, 1764.8295, 1769.7342, 1771.7337, 1941.8846, 1967.8497, 2005.7944, 2118.7803, 2134.9720, 2217.9430, 2234.8390, 2566.1228, 2618.1553, 2636.1695, 2663.1677, 2682.1808, 2708.1954, 2720.1183, 2724.1914, 3347.6860

24. PA2A1_VIPRE **Mass:** 16228 **Score:** 67 **Expect:** 0.00031 **Matches:** 3

Acidic phospholipase A2 PL1 OS=Vipera renardi OX=927686 PE=2 SV=1

Observed	Mr(expt)	Mr(calc)	ppm	Start		End	Miss	Ions	Peptide
909.3860	908.3787	908.3480	33.8	100	-	106	0	6	R.AVCECDR.V
1339.6298	1338.6225	1338.5988	17.7	123	-	132	0	52	K.YMLYSLFDCK.E
1355.6208	1354.6135	1354.5937	14.6	123	-	132	0	(2)	K.YMLYSLFDCK.E + Oxidation (M)

No match to: 817.1353, 831.0894, 837.0987, 839.1181, 852.3661, 855.0858, 859.0803, 865.5262, 868.5601, 871.0599, 874.0343, 896.0194, 903.4049, 936.2407, 952.4442, 987.5164, 1011.4809, 1022.1437, 1022.4987, 1044.1191, 1049.4942, 1064.0866, 1066.1056, 1068.5038, 1082.0794, 1113.6442, 1149.4939, 1157.6529, 1161.6449, 1173.6476, 1189.6416, 1193.6342, 1195.5987, 1205.6345, 1221.6251, 1234.6887, 1238.6385, 1249.1265, 1255.6430, 1267.6252, 1271.1047, 1283.6191, 1285.7483, 1305.5768, 1311.7646, 1323.6353, 1330.5501, 1343.5209, 1346.5381, 1361.5885, 1368.4953, 1372.5633, 1377.5715, 1393.5665, 1395.7189, 1411.7132, 1433.6712, 1476.1124, 1497.7909, 1625.8814, 1636.7437, 1674.6785, 1698.8155, 1718.7743, 1731.7859, 1733.7804, 1736.7580, 1740.7770, 1748.7813, 1754.7753, 1756.7402, 1764.8295, 1769.7342, 1771.7337, 1941.8846, 1967.8497, 2005.7944, 2118.7803, 2134.9720, 2217.9430, 2234.8390, 2566.1228, 2618.1553, 2636.1695, 2663.1677, 2682.1808, 2708.1954, 2720.1183, 2724.1914, 3347.6860

25. Q6A3J3_VIPBB **Mass:** 16280 **Score:** 42 **Expect:** 0.085 **Matches:** 4

Ammodytin I1(C) variant OS=Vipera berus berus OX=31156 PE=2 SV=1

Observed	Mr(expt)	Mr(calc)	ppm	Start		End	Miss	Ions	Peptide
909.3860	908.3787	908.3480	33.8	100	-	106	0	6	R.AVCECDR.V
1305.5768	1304.5696	1304.6145	-34.41	123	-	132	0	---	K.YMLYSLLDCK.E
1433.6712	1432.6639	1432.7094	-31.76	122	-	132	1	---	K.KYMLYSLLDCK.E
1748.7813	1747.7740	1747.7545	11.1	107	-	121	0	13	R.VAAICFGENMNTYDK.K + Oxidation (M)

No match to: 817.1353, 831.0894, 837.0987, 839.1181, 852.3661, 855.0858, 859.0803, 865.5262, 868.5601, 871.0599, 874.0343, 896.0194, 903.4049, 936.2407, 952.4442, 987.5164, 1011.4809, 1022.1437, 1022.4987, 1044.1191, 1049.4942, 1064.0866, 1066.1056, 1068.5038, 1082.0794, 1113.6442, 1149.4939, 1157.6529, 1161.6449, 1173.6476, 1189.6416, 1193.6342, 1195.5987, 1205.6345, 1221.6251, 1234.6887, 1238.6385, 1249.1265, 1255.6430, 1267.6252, 1271.1047, 1283.6191, 1285.7483, 1311.7646, 1323.6353, 1330.5501, 1339.6298, 1343.5209, 1346.5381, 1355.6208, 1361.5885, 1368.4953, 1372.5633, 1377.5715, 1393.5665, 1395.7189, 1411.7132, 1476.1124, 1497.7909, 1625.8814, 1636.7437, 1674.6785, 1698.8155, 1718.7743, 1731.7859, 1733.7804, 1736.7580, 1740.7770, 1754.7753, 1756.7402, 1764.8295, 1769.7342, 1771.7337, 1941.8846, 1967.8497, 2005.7944, 2118.7803, 2134.9720, 2217.9430, 2234.8390, 2566.1228, 2618.1553, 2636.1695, 2663.1677, 2682.1808, 2708.1954, 2720.1183, 2724.1914, 3347.6860

26. Q6A3H6_VIPAS **Mass:** 16258 **Score:** 42 **Expect:** 0.085 **Matches:** 4

Ammodytin I1(C) isoform OS=Vipera aspis atra OX=246182 PE=2 SV=1

Observed	Mr(expt)	Mr(calc)	ppm	Start		End	Miss	Ions	Peptide
909.3860	908.3787	908.3480	33.8	100	-	106	0	6	R.AVCECDR.V
1305.5768	1304.5696	1304.6145	-34.41	123	-	132	0	---	K.YMLYSLLDCK.E
1433.6712	1432.6639	1432.7094	-31.76	122	-	132	1	---	K.KYMLYSLLDCK.E
1748.7813	1747.7740	1747.7545	11.1	107	-	121	0	13	R.VAAICFGENMNTYDK.K + Oxidation (M)

No match to: 817.1353, 831.0894, 837.0987, 839.1181, 852.3661, 855.0858, 859.0803, 865.5262, 868.5601, 871.0599, 874.0343, 896.0194, 903.4049, 936.2407, 952.4442, 987.5164, 1011.4809, 1022.1437, 1022.4987, 1044.1191, 1049.4942, 1064.0866, 1066.1056, 1068.5038, 1082.0794, 1113.6442, 1149.4939, 1157.6529, 1161.6449, 1173.6476, 1189.6416, 1193.6342, 1195.5987, 1205.6345, 1221.6251, 1234.6887, 1238.6385, 1249.1265, 1255.6430, 1267.6252, 1271.1047, 1283.6191, 1285.7483, 1311.7646, 1323.6353, 1330.5501, 1339.6298, 1343.5209, 1346.5381, 1355.6208, 1361.5885, 1368.4953, 1372.5633, 1377.5715, 1393.5665, 1395.7189, 1411.7132, 1476.1124, 1497.7909, 1625.8814, 1636.7437, 1674.6785, 1698.8155, 1718.7743, 1731.7859, 1733.7804, 1736.7580, 1740.7770, 1754.7753, 1756.7402, 1764.8295, 1769.7342, 1771.7337, 1941.8846, 1967.8497, 2005.7944, 2118.7803, 2134.9720, 2217.9430, 2234.8390, 2566.1228, 2618.1553, 2636.1695, 2663.1677, 2682.1808, 2708.1954, 2720.1183, 2724.1914, 3347.6860

27. Q6A3J2_VIPBB **Mass:** 16244 **Score:** 42 **Expect:** 0.085 **Matches:** 4

Ammodytin I1(C) variant OS=Vipera berus berus OX=31156 PE=2 SV=1

Observed	Mr(expt)	Mr(calc)	ppm	Start		End	Miss	Ions	Peptide
909.3860	908.3787	908.3480	33.8	100	-	106	0	6	R.AVCECDR.V
1305.5768	1304.5696	1304.6145	-34.41	123	-	132	0	---	K.YMLYSLLDCK.E
1433.6712	1432.6639	1432.7094	-31.76	122	-	132	1	---	K.KYMLYSLLDCK.E
1748.7813	1747.7740	1747.7545	11.1	107	-	121	0	13	R.VAAICFGENMNTYDK.K + Oxidation (M)

No match to: 817.1353, 831.0894, 837.0987, 839.1181, 852.3661, 855.0858, 859.0803, 865.5262, 868.5601, 871.0599, 874.0343, 896.0194, 903.4049, 936.2407, 952.4442, 987.5164, 1011.4809, 1022.1437, 1022.4987, 1044.1191, 1049.4942, 1064.0866, 1066.1056, 1068.5038, 1082.0794, 1113.6442, 1149.4939, 1157.6529, 1161.6449, 1173.6476, 1189.6416, 1193.6342, 1195.5987, 1205.6345, 1221.6251, 1234.6887, 1238.6385, 1249.1265, 1255.6430, 1267.6252, 1271.1047, 1283.6191, 1285.7483, 1311.7646, 1323.6353, 1330.5501, 1339.6298, 1343.5209, 1346.5381, 1355.6208, 1361.5885, 1368.4953, 1372.5633, 1377.5715, 1393.5665, 1395.7189, 1411.7132, 1476.1124, 1497.7909, 1625.8814, 1636.7437, 1674.6785, 1698.8155, 1718.7743, 1731.7859, 1733.7804, 1736.7580, 1740.7770, 1754.7753, 1756.7402, 1764.8295, 1769.7342, 1771.7337, 1941.8846, 1967.8497, 2005.7944, 2118.7803, 2134.9720, 2217.9430, 2234.8390, 2566.1228, 2618.1553, 2636.1695, 2663.1677, 2682.1808, 2708.1954, 2720.1183, 2724.1914, 3347.6860

28. Q6A3J0_VIPBB **Mass:** 16280 **Score:** 42 **Expect:** 0.085 **Matches:** 4

Ammodytin I1(C) variant OS=Vipera berus berus OX=31156 PE=2 SV=1

Observed	Mr(expt)	Mr(calc)	ppm	Start		End	Miss	Ions	Peptide
909.3860	908.3787	908.3480	33.8	100	-	106	0	6	R.AVCECDR.V
1305.5768	1304.5696	1304.6145	-34.41	123	-	132	0	---	K.YMLYSLLDCK.E
1433.6712	1432.6639	1432.7094	-31.76	122	-	132	1	---	K.KYMLYSLLDCK.E
1748.7813	1747.7740	1747.7545	11.1	107	-	121	0	13	R.VAAICFGENMNTYDK.K + Oxidation (M)

No match to: 817.1353, 831.0894, 837.0987, 839.1181, 852.3661, 855.0858, 859.0803, 865.5262, 868.5601, 871.0599, 874.0343, 896.0194, 903.4049, 936.2407, 952.4442, 987.5164, 1011.4809, 1022.1437, 1022.4987, 1044.1191, 1049.4942, 1064.0866, 1066.1056, 1068.5038, 1082.0794, 1113.6442, 1149.4939, 1157.6529, 1161.6449, 1173.6476, 1189.6416, 1193.6342, 1195.5987, 1205.6345, 1221.6251, 1234.6887, 1238.6385, 1249.1265, 1255.6430, 1267.6252, 1271.1047, 1283.6191, 1285.7483, 1311.7646, 1323.6353, 1330.5501, 1339.6298, 1343.5209, 1346.5381, 1355.6208, 1361.5885, 1368.4953, 1372.5633, 1377.5715, 1393.5665, 1395.7189, 1411.7132, 1476.1124, 1497.7909, 1625.8814, 1636.7437, 1674.6785, 1698.8155, 1718.7743, 1731.7859, 1733.7804, 1736.7580, 1740.7770, 1754.7753, 1756.7402, 1764.8295, 1769.7342, 1771.7337, 1941.8846, 1967.8497, 2005.7944, 2118.7803, 2134.9720, 2217.9430, 2234.8390, 2566.1228, 2618.1553, 2636.1695, 2663.1677, 2682.1808, 2708.1954, 2720.1183, 2724.1914, 3347.6860

29. Q6A3H7_VIPAS **Mass:** 16244 **Score:** 42 **Expect:** 0.085 **Matches:** 4

Ammodytin I1(F) isoform OS=Vipera aspis atra OX=246182 PE=2 SV=1

Observed	Mr(expt)	Mr(calc)	ppm	Start	End	Miss	Ions	Peptide
909.3860	908.3787	908.3480	33.8	100 -	106	0	6	R.AVCECDR.V
1305.5768	1304.5696	1304.6145	-34.41	123 -	132	0	---	K.YMLYSLLDCK.E
1433.6712	1432.6639	1432.7094	-31.76	122 -	132	1	---	K.KYMLYSLLDCK.E
1748.7813	1747.7740	1747.7545	11.1	107 -	121	0	13	R.VAAICFGENMNTYDK.K + Oxidation (M)

No match to: 817.1353, 831.0894, 837.0987, 839.1181, 852.3661, 855.0858, 859.0803, 865.5262, 868.5601, 871.0599, 874.0343, 896.0194, 903.4049, 936.2407, 952.4442, 987.5164, 1011.4809, 1022.1437, 1022.4987, 1044.1191, 1049.4942, 1064.0866, 1066.1056, 1068.5038, 1082.0794, 1113.6442, 1149.4939, 1157.6529, 1161.6449, 1173.6476, 1189.6416, 1193.6342, 1195.5987, 1205.6345, 1221.6251, 1234.6887, 1238.6385, 1249.1265, 1255.6430, 1267.6252, 1271.1047, 1283.6191, 1285.7483, 1311.7646, 1323.6353, 1330.5501, 1339.6298, 1343.5209, 1346.5381, 1355.6208, 1361.5885, 1368.4953, 1372.5633, 1377.5715, 1393.5665, 1395.7189, 1411.7132, 1476.1124, 1497.7909, 1625.8814, 1636.7437, 1674.6785, 1698.8155, 1718.7743, 1731.7859, 1733.7804, 1736.7580, 1740.7770, 1754.7753, 1756.7402, 1764.8295, 1769.7342, 1771.7337, 1941.8846, 1967.8497, 2005.7944, 2118.7803, 2134.9720, 2217.9430, 2234.8390, 2566.1228, 2618.1553, 2636.1695, 2663.1677, 2682.1808, 2708.1954, 2720.1183, 2724.1914, 3347.6860

30. Q6A3H4_VIPAS **Mass:** 16244 **Score:** 42 **Expect:** 0.085 **Matches:** 4

Ammodytin I1(C) isoform OS=Vipera aspis atra OX=246182 PE=2 SV=1

Observed	Mr(expt)	Mr(calc)	ppm	Start	End	Miss	Ions	Peptide
909.3860	908.3787	908.3480	33.8	100 -	106	0	6	R.AVCECDR.V
1305.5768	1304.5696	1304.6145	-34.41	123 -	132	0	---	K.YMLYSLLDCK.E
1433.6712	1432.6639	1432.7094	-31.76	122 -	132	1	---	K.KYMLYSLLDCK.E
1748.7813	1747.7740	1747.7545	11.1	107 -	121	0	13	R.VAAICFGENMNTYDK.K + Oxidation (M)

No match to: 817.1353, 831.0894, 837.0987, 839.1181, 852.3661, 855.0858, 859.0803, 865.5262, 868.5601, 871.0599, 874.0343, 896.0194, 903.4049, 936.2407, 952.4442, 987.5164, 1011.4809, 1022.1437, 1022.4987, 1044.1191, 1049.4942, 1064.0866, 1066.1056, 1068.5038, 1082.0794, 1113.6442, 1149.4939, 1157.6529, 1161.6449, 1173.6476, 1189.6416, 1193.6342, 1195.5987, 1205.6345, 1221.6251, 1234.6887, 1238.6385, 1249.1265, 1255.6430, 1267.6252, 1271.1047, 1283.6191, 1285.7483, 1311.7646, 1323.6353, 1330.5501, 1339.6298, 1343.5209, 1346.5381, 1355.6208, 1361.5885, 1368.4953, 1372.5633, 1377.5715, 1393.5665, 1395.7189, 1411.7132, 1476.1124, 1497.7909, 1625.8814, 1636.7437, 1674.6785, 1698.8155, 1718.7743, 1731.7859, 1733.7804, 1736.7580, 1740.7770, 1754.7753, 1756.7402, 1764.8295, 1769.7342, 1771.7337, 1941.8846, 1967.8497, 2005.7944, 2118.7803, 2134.9720, 2217.9430, 2234.8390, 2566.1228, 2618.1553, 2636.1695, 2663.1677, 2682.1808, 2708.1954, 2720.1183, 2724.1914, 3347.6860

31. Q7T1D3_VIPAS **Mass:** 16258 **Score:** 42 **Expect:** 0.085 **Matches:** 4

Ammodytin I1 isoform 1 OS=Vipera aspis OX=8706 GN=AmtI1 PE=3 SV=1

Observed	Mr(expt)	Mr(calc)	ppm	Start	End	Miss	Ions	Peptide
909.3860	908.3787	908.3480	33.8	100 -	106	0	6	R.AVCECDR.V
1305.5768	1304.5696	1304.6145	-34.41	123 -	132	0	---	K.YMLYSLLDCK.E
1433.6712	1432.6639	1432.7094	-31.76	122 -	132	1	---	K.KYMLYSLLDCK.E
1748.7813	1747.7740	1747.7545	11.1	107 -	121	0	13	R.VAAICFGENMNTYDK.K + Oxidation (M)

No match to: 817.1353, 831.0894, 837.0987, 839.1181, 852.3661, 855.0858, 859.0803, 865.5262, 868.5601, 871.0599, 874.0343, 896.0194, 903.4049, 936.2407, 952.4442, 987.5164, 1011.4809, 1022.1437, 1022.4987, 1044.1191, 1049.4942, 1064.0866, 1066.1056, 1068.5038, 1082.0794, 1113.6442, 1149.4939, 1157.6529, 1161.6449, 1173.6476, 1189.6416, 1193.6342, 1195.5987, 1205.6345, 1221.6251, 1234.6887, 1238.6385, 1249.1265, 1255.6430, 1267.6252, 1271.1047, 1283.6191, 1285.7483, 1311.7646, 1323.6353, 1330.5501, 1339.6298, 1343.5209, 1346.5381, 1355.6208, 1361.5885, 1368.4953, 1372.5633, 1377.5715, 1393.5665, 1395.7189, 1411.7132, 1476.1124, 1497.7909, 1625.8814, 1636.7437, 1674.6785, 1698.8155, 1718.7743, 1731.7859, 1733.7804, 1736.7580, 1740.7770, 1754.7753, 1756.7402, 1764.8295, 1769.7342, 1771.7337, 1941.8846, 1967.8497, 2005.7944, 2118.7803, 2134.9720, 2217.9430, 2234.8390, 2566.1228, 2618.1553, 2636.1695, 2663.1677, 2682.1808, 2708.1954, 2720.1183, 2724.1914, 3347.6860

32. Q6A3I1_VIPAP **Mass:** 16288 **Score:** 42 **Expect:** 0.085 **Matches:** 4

Ammodytin I1(C) variant OS=Vipera aspis aspis OX=194601 PE=2 SV=1

Observed	Mr(expt)	Mr(calc)	ppm	Start	End	Miss	Ions	Peptide
909.3860	908.3787	908.3480	33.8	100 -	106	0	6	R.AVCECDR.V
1305.5768	1304.5696	1304.6145	-34.41	123 -	132	0	---	K.YMLYSLLDCK.E
1433.6712	1432.6639	1432.7094	-31.76	122 -	132	1	---	K.KYMLYSLLDCK.E
1748.7813	1747.7740	1747.7545	11.1	107 -	121	0	13	R.VAAICFGENMNTYDK.K + Oxidation (M)

No match to: 817.1353, 831.0894, 837.0987, 839.1181, 852.3661, 855.0858, 859.0803, 865.5262, 868.5601, 871.0599, 874.0343, 896.0194, 903.4049, 936.2407, 952.4442, 987.5164, 1011.4809, 1022.1437, 1022.4987, 1044.1191, 1049.4942, 1064.0866, 1066.1056, 1068.5038, 1082.0794, 1113.6442, 1149.4939, 1157.6529, 1161.6449, 1173.6476, 1189.6416, 1193.6342, 1195.5987, 1205.6345, 1221.6251, 1234.6887, 1238.6385, 1249.1265, 1255.6430, 1267.6252, 1271.1047, 1283.6191, 1285.7483, 1311.7646, 1323.6353, 1330.5501, 1339.6298, 1343.5209, 1346.5381, 1355.6208, 1361.5885, 1368.4953, 1372.5633, 1377.5715, 1393.5665, 1395.7189, 1411.7132, 1476.1124, 1497.7909, 1625.8814, 1636.7437, 1674.6785, 1698.8155, 1718.7743, 1731.7859, 1733.7804, 1736.7580, 1740.7770, 1754.7753, 1756.7402, 1764.8295, 1769.7342, 1771.7337, 1941.8846, 1967.8497, 2005.7944, 2118.7803, 2134.9720, 2217.9430, 2234.8390, 2566.1228, 2618.1553, 2636.1695, 2663.1677, 2682.1808, 2708.1954, 2720.1183, 2724.1914, 3347.6860

33. Q6A3I0_VIPAP **Mass:** 16272 **Score:** 42 **Expect:** 0.085 **Matches:** 4

Ammodytin I1(C) variant OS=Vipera aspis aspis OX=194601 PE=2 SV=1

Observed	Mr(expt)	Mr(calc)	ppm	Start	End	Miss	Ions	Peptide
909.3860	908.3787	908.3480	33.8	100 -	106	0	6	R.AVCECDR.V
1305.5768	1304.5696	1304.6145	-34.41	123 -	132	0	---	K.YMLYSLLDCK.E
1433.6712	1432.6639	1432.7094	-31.76	122 -	132	1	---	K.KYMLYSLLDCK.E
1748.7813	1747.7740	1747.7545	11.1	107 -	121	0	13	R.VAAICFGENMNTYDK.K + Oxidation (M)

No match to: 817.1353, 831.0894, 837.0987, 839.1181, 852.3661, 855.0858, 859.0803, 865.5262, 868.5601, 871.0599, 874.0343, 896.0194, 903.4049, 936.2407, 952.4442, 987.5164, 1011.4809, 1022.1437, 1022.4987, 1044.1191, 1049.4942, 1064.0866, 1066.1056, 1068.5038, 1082.0794, 1113.6442, 1149.4939, 1157.6529, 1161.6449, 1173.6476, 1189.6416, 1193.6342, 1195.5987, 1205.6345, 1221.6251, 1234.6887, 1238.6385, 1249.1265, 1255.6430, 1267.6252, 1271.1047, 1283.6191, 1285.7483, 1311.7646, 1323.6353, 1330.5501, 1339.6298, 1343.5209, 1346.5381, 1355.6208, 1361.5885, 1368.4953, 1372.5633, 1377.5715, 1393.5665, 1395.7189, 1411.7132, 1476.1124, 1497.7909, 1625.8814, 1636.7437, 1674.6785, 1698.8155, 1718.7743, 1731.7859, 1733.7804, 1736.7580, 1740.7770, 1754.7753, 1756.7402, 1764.8295, 1769.7342, 1771.7337, 1941.8846, 1967.8497, 2005.7944, 2118.7803, 2134.9720, 2217.9430, 2234.8390, 2566.1228, 2618.1553, 2636.1695, 2663.1677, 2682.1808, 2708.1954, 2720.1183, 2724.1914, 3347.6860

34. Q6A3I4_VIPAP **Mass:** 16272 **Score:** 42 **Expect:** 0.085 **Matches:** 4

Ammodytin I1(C) variant OS=Vipera aspis aspis OX=194601 PE=2 SV=1

Observed	Mr(expt)	Mr(calc)	ppm	Start	End	Miss	Ions	Peptide
909.3860	908.3787	908.3480	33.8	100 -	106	0	6	R.AVCECDR.V
1305.5768	1304.5696	1304.6145	-34.41	123 -	132	0	---	K.YMLYSLLDCK.E
1433.6712	1432.6639	1432.7094	-31.76	122 -	132	1	---	K.KYMLYSLLDCK.E
1748.7813	1747.7740	1747.7545	11.1	107 -	121	0	13	R.VAAICFGENMNTYDK.K + Oxidation (M)

No match to: 817.1353, 831.0894, 837.0987, 839.1181, 852.3661, 855.0858, 859.0803, 865.5262, 868.5601, 871.0599, 874.0343, 896.0194, 903.4049, 936.2407, 952.4442, 987.5164, 1011.4809, 1022.1437, 1022.4987, 1044.1191, 1049.4942, 1064.0866, 1066.1056, 1068.5038, 1082.0794, 1113.6442, 1149.4939, 1157.6529, 1161.6449, 1173.6476, 1189.6416, 1193.6342, 1195.5987, 1205.6345, 1221.6251, 1234.6887, 1238.6385, 1249.1265, 1255.6430, 1267.6252, 1271.1047, 1283.6191, 1285.7483, 1311.7646, 1323.6353, 1330.5501, 1339.6298, 1343.5209, 1346.5381, 1355.6208, 1361.5885, 1368.4953, 1372.5633, 1377.5715, 1393.5665, 1395.7189, 1411.7132, 1476.1124, 1497.7909, 1625.8814, 1636.7437, 1674.6785, 1698.8155, 1718.7743, 1731.7859, 1733.7804, 1736.7580, 1740.7770, 1754.7753, 1756.7402, 1764.8295, 1769.7342, 1771.7337, 1941.8846, 1967.8497, 2005.7944, 2118.7803, 2134.9720, 2217.9430, 2234.8390, 2566.1228, 2618.1553, 2636.1695, 2663.1677, 2682.1808, 2708.1954, 2720.1183, 2724.1914, 3347.6860

35. Q6A3H8_VIPAP **Mass:** 16330 **Score:** 42 **Expect:** 0.085 **Matches:** 4

Ammodytin I1(C) variant OS=Vipera aspis aspis OX=194601 PE=2 SV=1

Observed	Mr(expt)	Mr(calc)	ppm	Start	End	Miss	Ions	Peptide

909.3860	908.3787	908.3480	33.8	100 - 106	0	6	R.AVCECDR.V
1305.5768	1304.5696	1304.6145	-34.41	123 - 132	0	---	K.YMLYSLLDCK.E
1433.6712	1432.6639	1432.7094	-31.76	122 - 132	1	---	K.KYMLYSLLDCK.E
1748.7813	1747.7740	1747.7545	11.1	107 - 121	0	13	R.VAAICFGENMNTYDK.K + Oxidation (M)

No match to: 817.1353, 831.0894, 837.0987, 839.1181, 852.3661, 855.0858, 859.0803, 865.5262, 868.5601, 871.0599, 874.0343, 896.0194, 903.4049, 936.2407, 952.4442, 987.5164, 1011.4809, 1022.1437, 1022.4987, 1044.1191, 1049.4942, 1064.0866, 1066.1056, 1068.5038, 1082.0794, 1113.6442, 1149.4939, 1157.6529, 1161.6449, 1173.6476, 1189.6416, 1193.6342, 1195.5987, 1205.6345, 1221.6251, 1234.6887, 1238.6385, 1249.1265, 1255.6430, 1267.6252, 1271.1047, 1283.6191, 1285.7483, 1311.7646, 1323.6353, 1330.5501, 1339.6298, 1343.5209, 1346.5381, 1355.6208, 1361.5885, 1368.4953, 1372.5633, 1377.5715, 1393.5665, 1395.7189, 1411.7132, 1476.1124, 1497.7909, 1625.8814, 1636.7437, 1674.6785, 1698.8155, 1718.7743, 1731.7859, 1733.7804, 1736.7580, 1740.7770, 1754.7753, 1756.7402, 1764.8295, 1769.7342, 1771.7337, 1941.8846, 1967.8497, 2005.7944, 2118.7803, 2134.9720, 2217.9430, 2234.8390, 2566.1228, 2618.1553, 2636.1695, 2663.1677, 2682.1808, 2708.1954, 2720.1183, 2724.1914, 3347.6860

36. Q6A3K9_VIPAP **Mass:** 16202 **Score:** 42 **Expect:** 0.085 **Matches:** 4
Ammodytin I1(E) variant OS=Vipera aspis aspis OX=194601 PE=2 SV=1

Observed	Mr(expt)	Mr(calc)	ppm	Start - End	Miss	Ions	Peptide
909.3860	908.3787	908.3480	33.8	100 - 106	0	6	R.AVCECDR.V
1305.5768	1304.5696	1304.6145	-34.41	123 - 132	0	---	K.YMLYSLLDCK.E
1433.6712	1432.6639	1432.7094	-31.76	122 - 132	1	---	K.KYMLYSLLDCK.E
1748.7813	1747.7740	1747.7545	11.1	107 - 121	0	13	R.VAAICFGENMNTYDK.K + Oxidation (M)

No match to: 817.1353, 831.0894, 837.0987, 839.1181, 852.3661, 855.0858, 859.0803, 865.5262, 868.5601, 871.0599, 874.0343, 896.0194, 903.4049, 936.2407, 952.4442, 987.5164, 1011.4809, 1022.1437, 1022.4987, 1044.1191, 1049.4942, 1064.0866, 1066.1056, 1068.5038, 1082.0794, 1113.6442, 1149.4939, 1157.6529, 1161.6449, 1173.6476, 1189.6416, 1193.6342, 1195.5987, 1205.6345, 1221.6251, 1234.6887, 1238.6385, 1249.1265, 1255.6430, 1267.6252, 1271.1047, 1283.6191, 1285.7483, 1311.7646, 1323.6353, 1330.5501, 1339.6298, 1343.5209, 1346.5381, 1355.6208, 1361.5885, 1368.4953, 1372.5633, 1377.5715, 1393.5665, 1395.7189, 1411.7132, 1476.1124, 1497.7909, 1625.8814, 1636.7437, 1674.6785, 1698.8155, 1718.7743, 1731.7859, 1733.7804, 1736.7580, 1740.7770, 1754.7753, 1756.7402, 1764.8295, 1769.7342, 1771.7337, 1941.8846, 1967.8497, 2005.7944, 2118.7803, 2134.9720, 2217.9430, 2234.8390, 2566.1228, 2618.1553, 2636.1695, 2663.1677, 2682.1808, 2708.1954, 2720.1183, 2724.1914, 3347.6860

37. Q6A3I3_VIPAP **Mass:** 16286 **Score:** 42 **Expect:** 0.085 **Matches:** 4
Ammodytin I1(C) isoform OS=Vipera aspis aspis OX=194601 PE=2 SV=1

Observed	Mr(expt)	Mr(calc)	ppm	Start - End	Miss	Ions	Peptide
909.3860	908.3787	908.3480	33.8	100 - 106	0	6	R.AVCECDR.V
1305.5768	1304.5696	1304.6145	-34.41	123 - 132	0	---	K.YMLYSLLDCK.E
1433.6712	1432.6639	1432.7094	-31.76	122 - 132	1	---	K.KYMLYSLLDCK.E
1748.7813	1747.7740	1747.7545	11.1	107 - 121	0	13	R.VAAICFGENMNTYDK.K + Oxidation (M)

No match to: 817.1353, 831.0894, 837.0987, 839.1181, 852.3661, 855.0858, 859.0803, 865.5262, 868.5601, 871.0599, 874.0343, 896.0194, 903.4049, 936.2407, 952.4442, 987.5164, 1011.4809, 1022.1437, 1022.4987, 1044.1191, 1049.4942, 1064.0866, 1066.1056, 1068.5038, 1082.0794, 1113.6442, 1149.4939, 1157.6529, 1161.6449, 1173.6476, 1189.6416, 1193.6342, 1195.5987, 1205.6345, 1221.6251, 1234.6887, 1238.6385, 1249.1265, 1255.6430, 1267.6252, 1271.1047, 1283.6191, 1285.7483, 1311.7646, 1323.6353, 1330.5501, 1339.6298, 1343.5209, 1346.5381, 1355.6208, 1361.5885, 1368.4953, 1372.5633, 1377.5715, 1393.5665, 1395.7189, 1411.7132, 1476.1124, 1497.7909, 1625.8814, 1636.7437, 1674.6785, 1698.8155, 1718.7743, 1731.7859, 1733.7804, 1736.7580, 1740.7770, 1754.7753, 1756.7402, 1764.8295, 1769.7342, 1771.7337, 1941.8846, 1967.8497, 2005.7944, 2118.7803, 2134.9720, 2217.9430, 2234.8390, 2566.1228, 2618.1553, 2636.1695, 2663.1677, 2682.1808, 2708.1954, 2720.1183, 2724.1914, 3347.6860

38. Q6A3M5_VIPAP **Mass:** 16257 **Score:** 42 **Expect:** 0.085 **Matches:** 4
Ammodytin I1(C) variant OS=Vipera aspis aspis OX=194601 PE=2 SV=1

Observed	Mr(expt)	Mr(calc)	ppm	Start - End	Miss	Ions	Peptide
909.3860	908.3787	908.3480	33.8	100 - 106	0	6	R.AVCECDR.V
1305.5768	1304.5696	1304.6145	-34.41	123 - 132	0	---	K.YMLYSLLDCK.E
1433.6712	1432.6639	1432.7094	-31.76	122 - 132	1	---	K.KYMLYSLLDCK.E
1748.7813	1747.7740	1747.7545	11.1	107 - 121	0	13	R.VAAICFGENMNTYDK.K + Oxidation (M)

No match to: 817.1353, 831.0894, 837.0987, 839.1181, 852.3661, 855.0858, 859.0803, 865.5262, 868.5601, 871.0599, 874.0343, 896.0194, 903.4049, 936.2407, 952.4442, 987.5164, 1011.4809, 1022.1437, 1022.4987, 1044.1191, 1049.4942, 1064.0866, 1066.1056, 1068.5038, 1082.0794, 1113.6442, 1149.4939, 1157.6529, 1161.6449, 1173.6476, 1189.6416, 1193.6342, 1195.5987, 1205.6345, 1221.6251, 1234.6887, 1238.6385, 1249.1265, 1255.6430, 1267.6252, 1271.1047, 1283.6191, 1285.7483, 1311.7646, 1323.6353, 1330.5501, 1339.6298, 1343.5209, 1346.5381, 1355.6208, 1361.5885, 1368.4953, 1372.5633, 1377.5715, 1393.5665, 1395.7189, 1411.7132, 1476.1124, 1497.7909, 1625.8814, 1636.7437, 1674.6785, 1698.8155, 1718.7743, 1731.7859, 1733.7804, 1736.7580, 1740.7770, 1754.7753, 1756.7402, 1764.8295, 1769.7342, 1771.7337, 1941.8846, 1967.8497, 2005.7944, 2118.7803, 2134.9720, 2217.9430, 2234.8390, 2566.1228, 2618.1553, 2636.1695, 2663.1677, 2682.1808, 2708.1954, 2720.1183, 2724.1914, 3347.6860

39. Q6A3L1_VIPAP **Mass:** 16272 **Score:** 42 **Expect:** 0.085 **Matches:** 4
Ammodytin I1(E) isoform OS=Vipera aspis aspis OX=194601 PE=2 SV=1

Observed	Mr(expt)	Mr(calc)	ppm	Start - End	Miss	Ions	Peptide
909.3860	908.3787	908.3480	33.8	100 - 106	0	6	R.AVCECDR.V
1305.5768	1304.5696	1304.6145	-34.41	123 - 132	0	---	K.YMLYSLLDCK.E
1433.6712	1432.6639	1432.7094	-31.76	122 - 132	1	---	K.KYMLYSLLDCK.E
1748.7813	1747.7740	1747.7545	11.1	107 - 121	0	13	R.VAAICFGENMNTYDK.K + Oxidation (M)

No match to: 817.1353, 831.0894, 837.0987, 839.1181, 852.3661, 855.0858, 859.0803, 865.5262, 868.5601, 871.0599, 874.0343, 896.0194, 903.4049, 936.2407, 952.4442, 987.5164, 1011.4809, 1022.1437, 1022.4987, 1044.1191, 1049.4942, 1064.0866, 1066.1056, 1068.5038, 1082.0794, 1113.6442, 1149.4939, 1157.6529, 1161.6449, 1173.6476, 1189.6416, 1193.6342, 1195.5987, 1205.6345, 1221.6251, 1234.6887, 1238.6385, 1249.1265, 1255.6430, 1267.6252, 1271.1047, 1283.6191, 1285.7483, 1311.7646, 1323.6353, 1330.5501, 1339.6298, 1343.5209, 1346.5381, 1355.6208, 1361.5885, 1368.4953, 1372.5633, 1377.5715, 1393.5665, 1395.7189, 1411.7132, 1476.1124, 1497.7909, 1625.8814, 1636.7437, 1674.6785, 1698.8155, 1718.7743, 1731.7859, 1733.7804, 1736.7580, 1740.7770, 1754.7753, 1756.7402, 1764.8295, 1769.7342, 1771.7337, 1941.8846, 1967.8497, 2005.7944, 2118.7803, 2134.9720, 2217.9430, 2234.8390, 2566.1228, 2618.1553, 2636.1695, 2663.1677, 2682.1808, 2708.1954, 2720.1183, 2724.1914, 3347.6860

40. Q6A3L0_VIPAP **Mass:** 16360 **Score:** 42 **Expect:** 0.085 **Matches:** 4
Ammodytin I1(E) variant OS=Vipera aspis aspis OX=194601 PE=2 SV=1

Observed	Mr(expt)	Mr(calc)	ppm	Start - End	Miss	Ions	Peptide
909.3860	908.3787	908.3480	33.8	100 - 106	0	6	R.AVCECDR.V
1305.5768	1304.5696	1304.6145	-34.41	123 - 132	0	---	K.YMLYSLLDCK.E
1433.6712	1432.6639	1432.7094	-31.76	122 - 132	1	---	K.KYMLYSLLDCK.E
1748.7813	1747.7740	1747.7545	11.1	107 - 121	0	13	R.VAAICFGENMNTYDK.K + Oxidation (M)

No match to: 817.1353, 831.0894, 837.0987, 839.1181, 852.3661, 855.0858, 859.0803, 865.5262, 868.5601, 871.0599, 874.0343, 896.0194, 903.4049, 936.2407, 952.4442, 987.5164, 1011.4809, 1022.1437, 1022.4987, 1044.1191, 1049.4942, 1064.0866, 1066.1056, 1068.5038, 1082.0794, 1113.6442, 1149.4939, 1157.6529, 1161.6449, 1173.6476, 1189.6416, 1193.6342, 1195.5987, 1205.6345, 1221.6251, 1234.6887, 1238.6385, 1249.1265, 1255.6430, 1267.6252, 1271.1047, 1283.6191, 1285.7483, 1311.7646, 1323.6353, 1330.5501, 1339.6298, 1343.5209, 1346.5381, 1355.6208, 1361.5885, 1368.4953, 1372.5633, 1377.5715, 1393.5665, 1395.7189, 1411.7132, 1476.1124, 1497.7909, 1625.8814, 1636.7437, 1674.6785, 1698.8155, 1718.7743, 1731.7859, 1733.7804, 1736.7580, 1740.7770, 1754.7753, 1756.7402, 1764.8295, 1769.7342, 1771.7337, 1941.8846, 1967.8497, 2005.7944, 2118.7803, 2134.9720, 2217.9430, 2234.8390, 2566.1228, 2618.1553, 2636.1695, 2663.1677, 2682.1808, 2708.1954, 2720.1183, 2724.1914, 3347.6860

41. Q6A3G1_VIPAP **Mass:** 16246 **Score:** 42 **Expect:** 0.085 **Matches:** 4
Ammodytin I1(C') isoform OS=Vipera aspis aspis OX=194601 PE=2 SV=1

Observed	Mr(expt)	Mr(calc)	ppm	Start - End	Miss	Ions	Peptide
909.3860	908.3787	908.3480	33.8	100 - 106	0	6	R.AVCECDR.V
1305.5768	1304.5696	1304.6145	-34.41	123 - 132	0	---	K.YMLYSLLDCK.E

1433.6712	1432.6639	1432.7094	-31.76	122	132	1	---	K.KYMLYSLLDCK.E
1748.7813	1747.7740	1747.7545	11.1	107	121	0	13	R.VAAICFGENMNTYDK.K + Oxidation (M)

No match to: 817.1353, 831.0894, 837.0987, 839.1181, 852.3661, 855.0858, 859.0803, 865.5262, 868.5601, 871.0599, 874.0343, 896.0194, 903.4049, 936.2407, 952.4442, 987.5164, 1011.4809, 1022.1437, 1022.4987, 1044.1191, 1049.4942, 1064.0866, 1066.1056, 1068.5038, 1082.0794, 1113.6442, 1149.4939, 1157.6529, 1161.6449, 1173.6476, 1189.6416, 1193.6342, 1195.5987, 1205.6345, 1221.6251, 1234.6887, 1238.6385, 1249.1265, 1255.6430, 1267.6252, 1271.1047, 1283.6191, 1285.7483, 1311.7646, 1323.6353, 1330.5501, 1339.6298, 1343.5209, 1346.5381, 1355.6208, 1361.5885, 1368.4953, 1372.5633, 1377.5715, 1393.5665, 1395.7189, 1411.7132, 1476.1124, 1497.7909, 1625.8814, 1636.7437, 1674.6785, 1698.8155, 1718.7743, 1731.7859, 1733.7804, 1736.7580, 1740.7770, 1754.7753, 1756.7402, 1764.8295, 1769.7342, 1771.7337, 1941.8846, 1967.8497, 2005.7944, 2118.7803, 2134.9720, 2217.9430, 2234.8390, 2566.1228, 2618.1553, 2636.1695, 2663.1677, 2682.1808, 2708.1954, 2720.1183, 2724.1914, 3347.6860

42. Q6A3F7_VIPAP **Mass:** 16310 **Score:** 42 **Expect:** 0.085 **Matches:** 4
Ammodytin I1(C) variant OS=Vipera aspis aspis OX=194601 PE=2 SV=1

Observed	Mr(expt)	Mr(calc)	ppm	Start	End	Miss	Ions	Peptide
909.3860	908.3787	908.3480	33.8	100	106	0	6	R.AVCECDR.V
1305.5768	1304.5696	1304.6145	-34.41	123	132	0	---	K.YMLYSLLDCK.E
1433.6712	1432.6639	1432.7094	-31.76	122	132	1	---	K.KYMLYSLLDCK.E
1748.7813	1747.7740	1747.7545	11.1	107	121	0	13	R.VAAICFGENMNTYDK.K + Oxidation (M)

No match to: 817.1353, 831.0894, 837.0987, 839.1181, 852.3661, 855.0858, 859.0803, 865.5262, 868.5601, 871.0599, 874.0343, 896.0194, 903.4049, 936.2407, 952.4442, 987.5164, 1011.4809, 1022.1437, 1022.4987, 1044.1191, 1049.4942, 1064.0866, 1066.1056, 1068.5038, 1082.0794, 1113.6442, 1149.4939, 1157.6529, 1161.6449, 1173.6476, 1189.6416, 1193.6342, 1195.5987, 1205.6345, 1221.6251, 1234.6887, 1238.6385, 1249.1265, 1255.6430, 1267.6252, 1271.1047, 1283.6191, 1285.7483, 1311.7646, 1323.6353, 1330.5501, 1339.6298, 1343.5209, 1346.5381, 1355.6208, 1361.5885, 1368.4953, 1372.5633, 1377.5715, 1393.5665, 1395.7189, 1411.7132, 1476.1124, 1497.7909, 1625.8814, 1636.7437, 1674.6785, 1698.8155, 1718.7743, 1731.7859, 1733.7804, 1736.7580, 1740.7770, 1754.7753, 1756.7402, 1764.8295, 1769.7342, 1771.7337, 1941.8846, 1967.8497, 2005.7944, 2118.7803, 2134.9720, 2217.9430, 2234.8390, 2566.1228, 2618.1553, 2636.1695, 2663.1677, 2682.1808, 2708.1954, 2720.1183, 2724.1914, 3347.6860

43. Q7T1D4_VIPAP **Mass:** 16258 **Score:** 42 **Expect:** 0.085 **Matches:** 4
Ammodytin I1 OS=Vipera aspis aspis OX=194601 GN=AmtI1 PE=2 SV=1

Observed	Mr(expt)	Mr(calc)	ppm	Start	End	Miss	Ions	Peptide
909.3860	908.3787	908.3480	33.8	100	106	0	6	R.AVCECDR.V
1305.5768	1304.5696	1304.6145	-34.41	123	132	0	---	K.YMLYSLLDCK.E
1433.6712	1432.6639	1432.7094	-31.76	122	132	1	---	K.KYMLYSLLDCK.E
1748.7813	1747.7740	1747.7545	11.1	107	121	0	13	R.VAAICFGENMNTYDK.K + Oxidation (M)

No match to: 817.1353, 831.0894, 837.0987, 839.1181, 852.3661, 855.0858, 859.0803, 865.5262, 868.5601, 871.0599, 874.0343, 896.0194, 903.4049, 936.2407, 952.4442, 987.5164, 1011.4809, 1022.1437, 1022.4987, 1044.1191, 1049.4942, 1064.0866, 1066.1056, 1068.5038, 1082.0794, 1113.6442, 1149.4939, 1157.6529, 1161.6449, 1173.6476, 1189.6416, 1193.6342, 1195.5987, 1205.6345, 1221.6251, 1234.6887, 1238.6385, 1249.1265, 1255.6430, 1267.6252, 1271.1047, 1283.6191, 1285.7483, 1311.7646, 1323.6353, 1330.5501, 1339.6298, 1343.5209, 1346.5381, 1355.6208, 1361.5885, 1368.4953, 1372.5633, 1377.5715, 1393.5665, 1395.7189, 1411.7132, 1476.1124, 1497.7909, 1625.8814, 1636.7437, 1674.6785, 1698.8155, 1718.7743, 1731.7859, 1733.7804, 1736.7580, 1740.7770, 1754.7753, 1756.7402, 1764.8295, 1769.7342, 1771.7337, 1941.8846, 1967.8497, 2005.7944, 2118.7803, 2134.9720, 2217.9430, 2234.8390, 2566.1228, 2618.1553, 2636.1695, 2663.1677, 2682.1808, 2708.1954, 2720.1183, 2724.1914, 3347.6860

44. Q7T1D2_VIPAZ **Mass:** 16258 **Score:** 42 **Expect:** 0.085 **Matches:** 4
Ammodytin I1 OS=Vipera aspis zinnikeri OX=55427 GN=AmtI1 PE=2 SV=1

Observed	Mr(expt)	Mr(calc)	ppm	Start	End	Miss	Ions	Peptide
909.3860	908.3787	908.3480	33.8	100	106	0	6	R.AVCECDR.V
1305.5768	1304.5696	1304.6145	-34.41	123	132	0	---	K.YMLYSLLDCK.E
1433.6712	1432.6639	1432.7094	-31.76	122	132	1	---	K.KYMLYSLLDCK.E
1748.7813	1747.7740	1747.7545	11.1	107	121	0	13	R.VAAICFGENMNTYDK.K + Oxidation (M)

No match to: 817.1353, 831.0894, 837.0987, 839.1181, 852.3661, 855.0858, 859.0803, 865.5262, 868.5601, 871.0599, 874.0343, 896.0194, 903.4049, 936.2407, 952.4442, 987.5164, 1011.4809, 1022.1437, 1022.4987, 1044.1191, 1049.4942, 1064.0866, 1066.1056, 1068.5038, 1082.0794, 1113.6442, 1149.4939, 1157.6529, 1161.6449, 1173.6476, 1189.6416, 1193.6342, 1195.5987, 1205.6345, 1221.6251, 1234.6887, 1238.6385, 1249.1265, 1255.6430, 1267.6252, 1271.1047, 1283.6191, 1285.7483, 1311.7646, 1323.6353, 1330.5501, 1339.6298, 1343.5209, 1346.5381, 1355.6208, 1361.5885, 1368.4953, 1372.5633, 1377.5715, 1393.5665, 1395.7189, 1411.7132, 1476.1124, 1497.7909, 1625.8814, 1636.7437, 1674.6785, 1698.8155, 1718.7743, 1731.7859, 1733.7804, 1736.7580, 1740.7770, 1754.7753, 1756.7402, 1764.8295, 1769.7342, 1771.7337, 1941.8846, 1967.8497, 2005.7944, 2118.7803, 2134.9720, 2217.9430, 2234.8390, 2566.1228, 2618.1553, 2636.1695, 2663.1677, 2682.1808, 2708.1954, 2720.1183, 2724.1914, 3347.6860

45. Q6A3J4_VIPBB **Mass:** 16258 **Score:** 42 **Expect:** 0.085 **Matches:** 4
Ammodytin I1(C) isoform OS=Vipera berus berus OX=31156 PE=2 SV=1

Observed	Mr(expt)	Mr(calc)	ppm	Start	End	Miss	Ions	Peptide
909.3860	908.3787	908.3480	33.8	100	106	0	6	R.AVCECDR.V
1305.5768	1304.5696	1304.6145	-34.41	123	132	0	---	K.YMLYSLLDCK.E
1433.6712	1432.6639	1432.7094	-31.76	122	132	1	---	K.KYMLYSLLDCK.E
1748.7813	1747.7740	1747.7545	11.1	107	121	0	13	R.VAAICFGENMNTYDK.K + Oxidation (M)

No match to: 817.1353, 831.0894, 837.0987, 839.1181, 852.3661, 855.0858, 859.0803, 865.5262, 868.5601, 871.0599, 874.0343, 896.0194, 903.4049, 936.2407, 952.4442, 987.5164, 1011.4809, 1022.1437, 1022.4987, 1044.1191, 1049.4942, 1064.0866, 1066.1056, 1068.5038, 1082.0794, 1113.6442, 1149.4939, 1157.6529, 1161.6449, 1173.6476, 1189.6416, 1193.6342, 1195.5987, 1205.6345, 1221.6251, 1234.6887, 1238.6385, 1249.1265, 1255.6430, 1267.6252, 1271.1047, 1283.6191, 1285.7483, 1311.7646, 1323.6353, 1330.5501, 1339.6298, 1343.5209, 1346.5381, 1355.6208, 1361.5885, 1368.4953, 1372.5633, 1377.5715, 1393.5665, 1395.7189, 1411.7132, 1476.1124, 1497.7909, 1625.8814, 1636.7437, 1674.6785, 1698.8155, 1718.7743, 1731.7859, 1733.7804, 1736.7580, 1740.7770, 1754.7753, 1756.7402, 1764.8295, 1769.7342, 1771.7337, 1941.8846, 1967.8497, 2005.7944, 2118.7803, 2134.9720, 2217.9430, 2234.8390, 2566.1228, 2618.1553, 2636.1695, 2663.1677, 2682.1808, 2708.1954, 2720.1183, 2724.1914, 3347.6860

46. Q6A3H2_VIPAS **Mass:** 16198 **Score:** 42 **Expect:** 0.085 **Matches:** 4
Ammodytin I1(C) variant OS=Vipera aspis atra OX=246182 PE=2 SV=1

Observed	Mr(expt)	Mr(calc)	ppm	Start	End	Miss	Ions	Peptide
909.3860	908.3787	908.3480	33.8	100	106	0	6	R.AVCECDR.V
1305.5768	1304.5696	1304.6145	-34.41	123	132	0	---	K.YMLYSLLDCK.E
1433.6712	1432.6639	1432.7094	-31.76	122	132	1	---	K.KYMLYSLLDCK.E
1748.7813	1747.7740	1747.7545	11.1	107	121	0	13	R.VAAICFGENMNTYDK.K + Oxidation (M)

No match to: 817.1353, 831.0894, 837.0987, 839.1181, 852.3661, 855.0858, 859.0803, 865.5262, 868.5601, 871.0599, 874.0343, 896.0194, 903.4049, 936.2407, 952.4442, 987.5164, 1011.4809, 1022.1437, 1022.4987, 1044.1191, 1049.4942, 1064.0866, 1066.1056, 1068.5038, 1082.0794, 1113.6442, 1149.4939, 1157.6529, 1161.6449, 1173.6476, 1189.6416, 1193.6342, 1195.5987, 1205.6345, 1221.6251, 1234.6887, 1238.6385, 1249.1265, 1255.6430, 1267.6252, 1271.1047, 1283.6191, 1285.7483, 1311.7646, 1323.6353, 1330.5501, 1339.6298, 1343.5209, 1346.5381, 1355.6208, 1361.5885, 1368.4953, 1372.5633, 1377.5715, 1393.5665, 1395.7189, 1411.7132, 1476.1124, 1497.7909, 1625.8814, 1636.7437, 1674.6785, 1698.8155, 1718.7743, 1731.7859, 1733.7804, 1736.7580, 1740.7770, 1754.7753, 1756.7402, 1764.8295, 1769.7342, 1771.7337, 1941.8846, 1967.8497, 2005.7944, 2118.7803, 2134.9720, 2217.9430, 2234.8390, 2566.1228, 2618.1553, 2636.1695, 2663.1677, 2682.1808, 2708.1954, 2720.1183, 2724.1914, 3347.6860

47. Q6A3J6_VIPAZ **Mass:** 16330 **Score:** 42 **Expect:** 0.085 **Matches:** 4
Ammodytin I1(C) isoform OS=Vipera aspis zinnikeri OX=55427 PE=2 SV=1

Observed	Mr(expt)	Mr(calc)	ppm	Start	End	Miss	Ions	Peptide
909.3860	908.3787	908.3480	33.8	100	106	0	6	R.AVCECDR.V
1305.5768	1304.5696	1304.6145	-34.41	123	132	0	---	K.YMLYSLLDCK.E
1433.6712	1432.6639	1432.7094	-31.76	122	132	1	---	K.KYMLYSLLDCK.E
1748.7813	1747.7740	1747.7545	11.1	107	121	0	13	R.VAAICFGENMNTYDK.K + Oxidation (M)

No match to: 817.1353, 831.0894, 837.0987, 839.1181, 852.3661, 855.0858, 859.0803, 865.5262, 868.5601, 871.0599, 874.0343, 896.0194, 903.4049, 936.2407, 952.4442, 987.5164, 1011.4809, 1022.1437, 1022.4987, 1044.1191, 1049.4942, 1064.0866, 1066.1056, 1068.5038, 1082.0794, 1113.6442, 1149.4939, 1157.6529, 1161.6449, 1173.6476, 1189.6416, 1193.6342, 1195.5987, 1205.6345, 1221.6251, 1234.6887, 1238.6385, 1249.1265, 1255.6430, 1267.6252, 1271.1047, 1283.6191, 1285.7483, 1311.7646, 1323.6353, 1330.5501, 1339.6298, 1343.5209, 1346.5381, 1355.6208, 1361.5885, 1368.4953, 1372.5633, 1377.5715, 1393.5665, 1395.7189, 1411.7132, 1476.1124, 1497.7909, 1625.8814, 1636.7437, 1674.6785, 1698.8155, 1718.7743, 1731.7859, 1733.7804, 1736.7580, 1740.7770, 1754.7753, 1756.7402, 1764.8295, 1769.7342, 1771.7337, 1941.8846, 1967.8497, 2005.7944, 2118.7803, 2134.9720, 2217.9430, 2234.8390, 2566.1228, 2618.1553, 2636.1695, 2663.1677, 2682.1808, 2708.1954, 2720.1183, 2724.1914, 3347.6860

48. PA2B_DABRR **Mass:** 14415 **Score:** 35 **Expect:** 0.41 **Matches:** 5

Basic phospholipase A2 RVV-VD OS=Daboia russelii OX=8707 PE=1 SV=1

Observed	Mr(expt)	Mr(calc)	ppm	Start	End	Miss	Ions	Peptide
909.3860	908.3787	908.3480	33.8	84 -	90	0	6	R.AVCECDR.V
987.5164	986.5092	986.4781	31.5	34 -	42	0	4	K.GKPQDATDR.C
1339.6298	1338.6225	1338.7006	-58.31	1 -	11	0	---	-.NLFQFAEMIVK.M
1355.6208	1354.6135	1354.6955	-60.49	1 -	11	0	---	-.NLFQFAEMIVK.M + Oxidation (M)
1756.7402	1755.7330	1755.9052	-98.07	1 -	15	1	---	-.NLFQFAEMIVKMTGK.N

No match to: 817.1353, 831.0894, 837.0987, 839.1181, 852.3661, 855.0858, 859.0803, 865.5262, 868.5601, 871.0599, 874.0343, 896.0194, 903.4049, 936.2407, 952.4442, 1011.4809, 1022.1437, 1022.4987, 1044.1191, 1049.4942, 1064.0866, 1066.1056, 1068.5038, 1082.0794, 1113.6442, 1149.4939, 1157.6529, 1161.6449, 1173.6476, 1189.6416, 1193.6342, 1195.5987, 1205.6345, 1221.6251, 1234.6887, 1238.6385, 1249.1265, 1255.6430, 1267.6252, 1271.1047, 1283.6191, 1285.7483, 1305.5768, 1311.7646, 1323.6353, 1330.5501, 1343.5209, 1346.5381, 1361.5885, 1368.4953, 1372.5633, 1377.5715, 1393.5665, 1395.7189, 1411.7132, 1433.6712, 1476.1124, 1497.7909, 1625.8814, 1636.7437, 1674.6785, 1698.8155, 1718.7743, 1731.7859, 1733.7804, 1736.7580, 1740.7770, 1748.7813, 1754.7753, 1764.8295, 1769.7342, 1771.7337, 1941.8846, 1967.8497, 2005.7944, 2118.7803, 2134.9720, 2217.9430, 2234.8390, 2566.1228, 2618.1553, 2636.1695, 2663.1677, 2682.1808, 2708.1954, 2720.1183, 2724.1914, 3347.6860

49. Q6A3H1_VIPAS **Mass:** 16292 **Score:** 34 **Expect:** 0.51 **Matches:** 4

Ammodytin I1(C) variant OS=Vipera aspis atra OX=246182 PE=2 SV=1

Observed	Mr(expt)	Mr(calc)	ppm	Start	End	Miss	Ions	Peptide
909.3860	908.3787	908.3480	33.8	100 -	106	0	6	R.AVCECDR.V
1339.6298	1338.6225	1338.5988	17.7	123 -	132	0	---	K.YMFYSLLDCK.E
1355.6208	1354.6135	1354.5937	14.6	123 -	132	0	---	K.YMFYSLLDCK.E + Oxidation (M)
1748.7813	1747.7740	1747.7545	11.1	107 -	121	0	13	R.VAAICFGENMNTYDK.K + Oxidation (M)

No match to: 817.1353, 831.0894, 837.0987, 839.1181, 852.3661, 855.0858, 859.0803, 865.5262, 868.5601, 871.0599, 874.0343, 896.0194, 903.4049, 936.2407, 952.4442, 987.5164, 1011.4809, 1022.1437, 1022.4987, 1044.1191, 1049.4942, 1064.0866, 1066.1056, 1068.5038, 1082.0794, 1113.6442, 1149.4939, 1157.6529, 1161.6449, 1173.6476, 1189.6416, 1193.6342, 1195.5987, 1205.6345, 1221.6251, 1234.6887, 1238.6385, 1249.1265, 1255.6430, 1267.6252, 1271.1047, 1283.6191, 1285.7483, 1305.5768, 1311.7646, 1323.6353, 1330.5501, 1343.5209, 1346.5381, 1361.5885, 1368.4953, 1372.5633, 1377.5715, 1393.5665, 1395.7189, 1411.7132, 1433.6712, 1476.1124, 1497.7909, 1625.8814, 1636.7437, 1674.6785, 1698.8155, 1718.7743, 1731.7859, 1733.7804, 1736.7580, 1740.7770, 1754.7753, 1756.7402, 1764.8295, 1769.7342, 1771.7337, 1941.8846, 1967.8497, 2005.7944, 2118.7803, 2134.9720, 2217.9430, 2234.8390, 2566.1228, 2618.1553, 2636.1695, 2663.1677, 2682.1808, 2708.1954, 2720.1183, 2724.1914, 3347.6860

50. Q6A3F6_VIPAP **Mass:** 16200 **Score:** 29 **Expect:** 1.9 **Matches:** 3

Ammodytin I1 (C) variant OS=Vipera aspis aspis OX=194601 PE=2 SV=1

Observed	Mr(expt)	Mr(calc)	ppm	Start	End	Miss	Ions	Peptide
1305.5768	1304.5696	1304.6145	-34.41	123 -	132	0	---	K.YMLYSLLDCK.E
1433.6712	1432.6639	1432.7094	-31.76	122 -	132	1	---	K.KYMLYSLLDCK.E
1748.7813	1747.7740	1747.7545	11.1	107 -	121	0	13	R.VAAICFGENMNTYDK.K + Oxidation (M)

No match to: 817.1353, 831.0894, 837.0987, 839.1181, 852.3661, 855.0858, 859.0803, 865.5262, 868.5601, 871.0599, 874.0343, 896.0194, 903.4049, 909.3860, 936.2407, 952.4442, 987.5164, 1011.4809, 1022.1437, 1022.4987, 1044.1191, 1049.4942, 1064.0866, 1066.1056, 1068.5038, 1082.0794, 1113.6442, 1149.4939, 1157.6529, 1161.6449, 1173.6476, 1189.6416, 1193.6342, 1195.5987, 1205.6345, 1221.6251, 1234.6887, 1238.6385, 1249.1265, 1255.6430, 1267.6252, 1271.1047, 1283.6191, 1285.7483, 1311.7646, 1323.6353, 1330.5501, 1339.6298, 1343.5209, 1346.5381, 1355.6208, 1361.5885, 1368.4953, 1372.5633, 1377.5715, 1393.5665, 1395.7189, 1411.7132, 1476.1124, 1497.7909, 1625.8814, 1636.7437, 1674.6785, 1698.8155, 1718.7743, 1731.7859, 1733.7804, 1736.7580, 1740.7770, 1754.7753, 1756.7402, 1764.8295, 1769.7342, 1771.7337, 1941.8846, 1967.8497, 2005.7944, 2118.7803, 2134.9720, 2217.9430, 2234.8390, 2566.1228, 2618.1553, 2636.1695, 2663.1677, 2682.1808, 2708.1954, 2720.1183, 2724.1914, 3347.6860

Search Parameters

Type of search : MS/MS Ion Search
Enzyme : Trypsin
Fixed modifications : Carbamidomethyl (C)
Variable modifications : Oxidation (M)
Mass values : Monoisotopic
Protein Mass : Unrestricted
Peptide Mass Tolerance : ± 100 ppm
Fragment Mass Tolerance: ± 0.7 Da
Max Missed Cleavages : 1
Instrument type : MALDI-TOF-TOF
Query1 (817.1353,1+) : <no title>
Query2 (831.0894,1+) : <no title>
Query3 (837.0987,1+) : <no title>
Query4 (839.1181,1+) : <no title>
Query5 (852.3661,1+) : <no title>
Query6 (855.0858,1+) : \\PROTEOMICS1\D\DATA\2105\210505_TLIEPOL_1_68252-68266\68252_68266_LPARDO\0_G9\1\855.0858.LIFT\1SREF\PDATA\1\1R
Query7 (859.0803,1+) : <no title>
Query8 (865.5262,1+) : <no title>
Query9 (868.5601,1+) : <no title>
Query10 (871.0599,1+) : <no title>
Query11 (874.0343,1+) : <no title>
Query12 (896.0194,1+) : <no title>
Query13 (903.4049,1+) : <no title>
Query14 (909.3860,1+) : \\PROTEOMICS1\D\DATA\2105\210505_TLIEPOL_1_68252-68266\68252_68266_LPARDO\0_G9\1\909.3860.LIFT\1SREF\PDATA\1\1R
Query15 (936.2407,1+) : <no title>
Query16 (952.4442,1+) : <no title>
Query17 (987.5164,1+) : \\PROTEOMICS1\D\DATA\2105\210505_TLIEPOL_1_68252-68266\68252_68266_LPARDO\0_G9\1\987.5164.LIFT\1SREF\PDATA\1\1R
Query18 (1011.4809,1+) : <no title>
Query19 (1022.1437,1+) : <no title>
Query20 (1022.4987,1+) : <no title>
Query21 (1044.1191,1+) : <no title>
Query22 (1049.4942,1+) : <no title>
Query23 (1064.0866,1+) : <no title>
Query24 (1066.1056,1+) : <no title>
Query25 (1068.5038,1+) : <no title>
Query26 (1082.0794,1+) : <no title>
Query27 (1113.6442,1+) : <no title>
Query28 (1149.4939,1+) : <no title>
Query29 (1157.6529,1+) : \\PROTEOMICS1\D\DATA\2105\210505_TLIEPOL_1_68252-68266\68252_68266_LPARDO\0_G9\1\1157.6529.LIFT\1SREF\PDATA\1\1R
Query30 (1161.6449,1+) : <no title>
Query31 (1173.6476,1+) : <no title>
Query32 (1189.6416,1+) : <no title>
Query33 (1193.6342,1+) : <no title>

Query34 (1195.5987,1+) : <no title>
Query35 (1205.6345,1+) : <no title>
Query36 (1221.6251,1+) : <no title>
Query37 (1234.6887,1+) : <no title>
Query38 (1238.6385,1+) : <no title>
Query39 (1249.1265,1+) : <no title>
Query40 (1255.6430,1+) : <no title>
Query41 (1267.6252,1+) : <no title>
Query42 (1271.1047,1+) : <no title>
Query43 (1283.6191,1+) : <no title>
Query44 (1285.7483,1+) : <no title>
Query45 (1305.5768,1+) : <no title>
Query46 (1311.7646,1+) : <no title>
Query47 (1323.6353,1+) : <no title>
Query48 (1330.5501,1+) : \\PROTEOMICS1\D\DATA\2105\210505_TLIEPOL_1_68252-68266\68252_68266_LPARDO\0_G9\1\1330.5501.LIFT\1SREF\PDATA\1\1R
Query49 (1339.6298,1+) : \\PROTEOMICS1\D\DATA\2105\210505_TLIEPOL_1_68252-68266\68252_68266_LPARDO\0_G9\1\1339.6298.LIFT\1SREF\PDATA\1\1R
Query50 (1343.5209,1+) : <no title>
Query51 (1346.5381,1+) : <no title>
Query52 (1355.6208,1+) : \\PROTEOMICS1\D\DATA\2105\210505_TLIEPOL_1_68252-68266\68252_68266_LPARDO\0_G9\1\1355.6208.LIFT\1SREF\PDATA\1\1R
Query53 (1361.5885,1+) : <no title>
Query54 (1368.4953,1+) : <no title>
Query55 (1372.5633,1+) : <no title>
Query56 (1377.5715,1+) : <no title>
Query57 (1393.5665,1+) : <no title>
Query58 (1395.7189,1+) : <no title>
Query59 (1411.7132,1+) : <no title>
Query60 (1433.6712,1+) : <no title>
Query61 (1476.1124,1+) : <no title>
Query62 (1497.7909,1+) : <no title>
Query63 (1625.8814,1+) : <no title>
Query64 (1636.7437,1+) : \\PROTEOMICS1\D\DATA\2105\210505_TLIEPOL_1_68252-68266\68252_68266_LPARDO\0_G9\1\1636.7437.LIFT\1SREF\PDATA\1\1R
Query65 (1674.6785,1+) : <no title>
Query66 (1698.8155,1+) : <no title>
Query67 (1718.7743,1+) : <no title>
Query68 (1731.7859,1+) : <no title>
Query69 (1733.7804,1+) : <no title>
Query70 (1736.7580,1+) : <no title>
Query71 (1740.7770,1+) : <no title>
Query72 (1748.7813,1+) : \\PROTEOMICS1\D\DATA\2105\210505_TLIEPOL_1_68252-68266\68252_68266_LPARDO\0_G9\1\1748.7813.LIFT\1SREF\PDATA\1\1R
Query73 (1754.7753,1+) : <no title>
Query74 (1756.7402,1+) : <no title>
Query75 (1764.8295,1+) : <no title>
Query76 (1769.7342,1+) : <no title>
Query77 (1771.7337,1+) : <no title>
Query78 (1941.8846,1+) : \\PROTEOMICS1\D\DATA\2105\210505_TLIEPOL_1_68252-68266\68252_68266_LPARDO\0_G9\1\1941.8846.LIFT\1SREF\PDATA\1\1R
Query79 (1967.8497,1+) : <no title>
Query80 (2005.7944,1+) : <no title>
Query81 (2118.7803,1+) : <no title>
Query82 (2134.9720,1+) : <no title>
Query83 (2217.9430,1+) : <no title>
Query84 (2234.8390,1+) : \\PROTEOMICS1\D\DATA\2105\210505_TLIEPOL_1_68252-68266\68252_68266_LPARDO\0_G9\1\2234.8390.LIFT\1SREF\PDATA\1\1R
Query85 (2566.1228,1+) : <no title>
Query86 (2618.1553,1+) : <no title>
Query87 (2636.1695,1+) : <no title>
Query88 (2663.1677,1+) : <no title>
Query89 (2682.1808,1+) : \\PROTEOMICS1\D\DATA\2105\210505_TLIEPOL_1_68252-68266\68252_68266_LPARDO\0_G9\1\2682.1808.LIFT\1SREF\PDATA\1\1R
Query90 (2708.1954,1+) : <no title>
Query91 (2720.1183,1+) : <no title>
Query92 (2724.1914,1+) : <no title>
Query93 (3347.6860,1+) : <no title>

Mascot: http://www.matrixscience.com/

MATRIX SCIENCE Mascot Search Results **Fr6-1**

```
User          :
Email         :
Search title  : vipera_All_8IDplus4 file:\\Proteomics1\d\data\2105\210505_tliepol_1_68252-68266\68252_68266_1pardo\0_H9\1\1SRef\pdata\1\peaklist.xml
Database      : vipera  (1414 sequences; 347452 residues)
Timestamp     : 5 May 2021 at 09:36:37 GMT
Top Score     : 40 for VSP2_MACLB, Venom serine proteinase-like protein 2 OS=Macrovipera lebetina OX=8709 PE=2 SV=1
```

Mascot Score Histogram

Protein score is -10*Log(P), where P is the probability that the observed match is a random event.
Protein scores greater than 44 are significant (p<0.05).

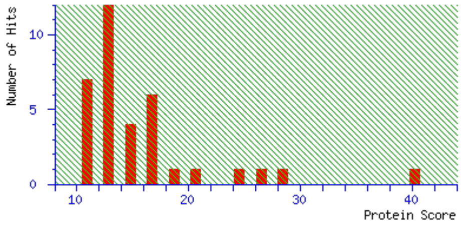

Concise Protein Summary Report

Format As | Concise Protein Summary | Help

Significance threshold p< 0.05 Max. number of hits 10

Re-Search All | Search Unmatched

1. VSP2_MACLB **Mass:** 29559 **Score:** 40 **Expect:** 0.14 **Matches:** 7
   Venom serine proteinase-like protein 2 OS=Macrovipera lebetina OX=8709 PE=2 SV=1
   A0A6B7FMQ3_VIPAA **Mass:** 28909 **Score:** 15 **Expect:** 42 **Matches:** 3
   Snake venom serine protease OS=Vipera ammodytes ammodytes OX=8705 PE=2 SV=1
   PA2N_DABRR **Mass:** 2192 **Score:** 12 **Expect:** 1e+002 **Matches:** 1
   Neutral phospholipase A2 RVV-PFIIc' (Fragment) OS=Daboia russelii OX=8707 PE=1 SV=1

2. A0A6G5ZVU6_9SAUR **Mass:** 11256 **Score:** 28 **Expect:** 2 **Matches:** 4
   Neurotrophin-4 (Fragment) OS=Vipera anatolica senliki OX=2604287 PE=2 SV=1

3. 3SA1_DABRR **Mass:** 2363 **Score:** 27 **Expect:** 2.8 **Matches:** 2
   Cytotoxin drCT-1 (Fragment) OS=Daboia russelii OX=8707 PE=1 SV=1

4. A0A286S1C5_9SAUR **Mass:** 39024 **Score:** 24 **Expect:** 5.4 **Matches:** 9
   E3 ubiquitin-protein ligase RAG1 (Fragment) OS=Vipera seoanei OX=110211 GN=RAG1 PE=4 SV=1
   A0A286S143_VIPAS **Mass:** 38971 **Score:** 11 **Expect:** 1e+002 **Matches:** 5
   E3 ubiquitin-protein ligase RAG1 (Fragment) OS=Vipera aspis OX=8706 GN=RAG1 PE=4 SV=1

5. VASP1_VIPAA **Mass:** 22635 **Score:** 21 **Expect:** 11 **Matches:** 4
   Snake venom serine protease VaSP1 (Fragments) OS=Vipera ammodytes ammodytes OX=8705 PE=1 SV=1

6. A0A6G5ZVX7_9SAUR **Mass:** 29593 **Score:** 19 **Expect:** 18 **Matches:** 7
   Serine protease 7 OS=Vipera anatolica senliki OX=2604287 PE=2 SV=1
   A0A6G5ZUR6_9SAUR **Mass:** 29579 **Score:** 19 **Expect:** 18 **Matches:** 7
   Serine protease 6 OS=Vipera anatolica senliki OX=2604287 PE=2 SV=1
   VSPH1_VIPAA **Mass:** 29593 **Score:** 19 **Expect:** 18 **Matches:** 7
   Vaa serine proteinase homolog 1 OS=Vipera ammodytes ammodytes OX=8705 PE=1 SV=1
   A0A6G5ZUW7_9SAUR **Mass:** 22103 **Score:** 13 **Expect:** 66 **Matches:** 6
   Serine protease 10 (Fragment) OS=Vipera anatolica senliki OX=2604287 PE=2 SV=1

7. D8MIA1_BITRH **Mass:** 29504 **Score:** 18 **Expect:** 25 **Matches:** 4
   Rhinocerase 3 protein (Fragment) OS=Bitis rhinoceros OX=715877 GN=rhinocerase 3 PE=3 SV=1

8. A0A0H3Y3P3_9PERO **Mass:** 25324 **Score:** 17 **Expect:** 26 **Matches:** 4
   Cardiac muscle myosin heavy chain 6 (Fragment) OS=Echiichthys vipera OX=94984 GN=myh6 PE=3 SV=1

9. A0A6G5ZVW2_9SAUR **Mass:** 30105 **Score:** 17 **Expect:** 27 **Matches:** 7
   Calumenin 4 (Fragment) OS=Vipera anatolica senliki OX=2604287 PE=2 SV=1
   A0A6G5ZU96_9SAUR **Mass:** 37065 **Score:** 16 **Expect:** 32 **Matches:** 7
   Calumenin 1 OS=Vipera anatolica senliki OX=2604287 PE=2 SV=1
   A0A6G5ZVL9_9SAUR **Mass:** 37117 **Score:** 16 **Expect:** 32 **Matches:** 7
   Calumenin 3 OS=Vipera anatolica senliki OX=2604287 PE=2 SV=1

10. NTF4_MACLB **Mass:** 4819 **Score:** 17 **Expect:** 27 **Matches:** 3
    Neurotrophin-4 (Fragment) OS=Macrovipera lebetina OX=8709 GN=NTF4 PE=3 SV=1

Search Parameters

```
Type of search         : Peptide Mass Fingerprint
Enzyme                 : Trypsin
Fixed modifications    : Carbamidomethyl (C)
Variable modifications : Oxidation (M)
Mass values            : Monoisotopic
Protein Mass           : Unrestricted
Peptide Mass Tolerance : ± 100 ppm
Peptide Charge State   : 1+
Max Missed Cleavages   : 1
Number of queries      : 98
Selected for scoring   : 50
```

**Mascot:** http://www.matrixscience.com/

Mascot Search Results

```
User          :
Email         :
Search title  : vipera_all_Lift file:\\Proteomics1\d\data\2105\210505_tliepol_1_68252-68266\68252_68266_lpardo\0_H9\1\0_H9.xml
Database      : vipera  (1414 sequences; 347452 residues)
Timestamp     : 5 May 2021 at 10:01:13 GMT
Protein hits  : VSPBF_MACLB      Beta-fibrinogenase OS=Macrovipera lebetina OX=8709 PE=1 SV=1
                A0A6G5ZUR6_9SAUR Serine protease 6 OS=Vipera anatolica senliki OX=2604287 PE=2 SV=1
                A0A6G7GGC6_VIPBE Rhodopsin (Fragment) OS=Vipera berus OX=31155 GN=RHO PE=2 SV=1
```

Mascot Score Histogram

Ions score is -10*Log(P), where P is the probability that the observed match is a random event.
Individual ions scores > 11 indicate identity or extensive homology (p<0.05).
Protein scores are derived from ions scores as a non-probabilistic basis for ranking protein hits.

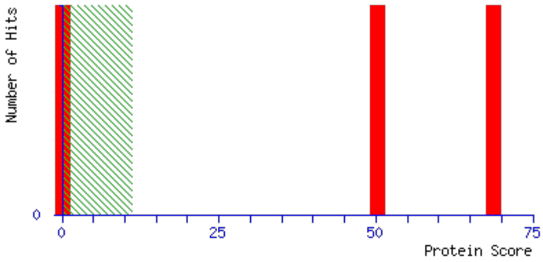

Peptide Summary Report

Format As: Peptide Summary | Help

Significance threshold p< 0.05 | Max. number of hits 10

Standard scoring ○ MudPIT scoring ◉ | Ions score or expect cut-off 0 | Show sub-sets 0

Show pop-ups ◉ Suppress pop-ups ○ | Sort unassigned Decreasing Score | Require bold red ☐

Overview Table

Click on column header to jump to entry in results list.
Move mouse over any indicator to highlight identical peptides.
Click on an indicator to see details of individual match.
Use check boxes to select sub-set of queries for new search.

Mouse over: -Query- | -Accession- | -Sequence-

Hit:	1	2	3
☑ 843.4335 (1+)			
☑ 880.4569 (1+)			
☑ 888.4500 (1+)			
☑ 895.3970 (1+)			
☑ 896.4510 (1+)			
☑ 906.4704 (1+)			
☑ 908.5083 (1+)			
☑ 914.4639 (1+)			
☑ 922.4699 (1+)			
☑ 1009.4438 (1+)			
☑ 1049.4386 (1+)			
☑ 1066.4633 (1+)			
☑ 1077.4646 (1+)			
☑ 1108.4728 (1+)			
☑ 1169.5911 (1+)			
☑ 1170.0892 (1+)			
☑ 1189.6611 (1+)			
☑ 1194.5557 (1+)			
☑ 1205.5920 (1+)			
☑ 1212.6192 (1+)			
☑ 1218.6543 (1+)			
☑ 1231.6425 (1+)			
☑ 1233.6705 (1+)	●		
☑ 1237.6639 (1+)			
☑ 1247.6491 (1+)			

☑ 1249.6639 (1+)		
☑ 1255.6491 (1+)		
☑ 1259.6864 (1+)		
☑ 1265.6592 (1+)		
☑ 1271.6156 (1+)		
☑ 1275.6818 (1+)		
☑ 1281.6541 (1+)		
☑ 1291.6754 (1+)		
☑ 1301.6959 (1+)		
☑ 1304.5938 (1+)		
☑ 1307.6705 (1+)		
☑ 1326.6012 (1+)		
☑ 1334.5881 (1+)		
☑ 1340.7389 (1+)		
☑ 1429.7302 (1+)		
☑ 1433.8111 (1+)		
☑ 1462.7902 (1+)		
☑ 1477.8012 (1+)		
☑ 1481.7954 (1+)		
☑ 1490.7698 (1+)		
☑ 1493.7944 (1+)		
☑ 1498.6923 (1+)		
☑ 1505.7880 (1+)		
☑ 1506.2822 (1+)		
☑ 1507.0891 (1+)		
☑ 1509.7892 (1+)		
☑ 1515.7405 (1+)		
☑ 1521.8025 (1+)		
☑ 1525.7827 (1+)		
☑ 1534.8120 (1+)		
☑ 1547.8074 (1+)		
☑ 1563.8131 (1+)		
☑ 1599.8532 (1+)		
☑ 1606.8106 (1+)		
☑ 1611.7753 (1+)		
☑ 1685.8462 (1+)		
☑ 1711.8596 (1+)		
☑ 1723.7778 (1+)		
☑ 1727.8452 (1+)		
☑ 1728.9532 (1+)		
☑ 1738.0576 (1+)		
☑ 1776.9531 (1+)		
☑ 1792.9390 (1+)		
☑ 1802.9620 (1+)		
☑ 1818.9616 (1+)		
☑ 1821.9696 (1+)		
☑ 1823.9891 (1+)		
☑ 1841.9405 (1+)		
☑ 1861.9851 (1+)		
☑ 2113.0764 (1+)	●	
☑ 2129.0720 (1+)		
☑ 2145.0632 (1+)		
☑ 2146.8377 (1+)		
☑ 2149.1282 (1+)		●
☑ 2164.8462 (1+)		
☑ 2184.0111 (1+)		
☑ 2191.8581 (1+)		
☑ 2210.8694 (1+)		
☑ 2236.8811 (1+)		
☑ 2252.8802 (1+)		
☑ 2338.1486 (1+)		
☑ 2462.2482 (1+)		
☑ 2558.1498 (1+)		
☑ 2609.3629 (1+)		
☑ 2678.3299 (1+)		
☑ 2804.1100 (1+)		
☑ 2818.1227 (1+)		

☑ 2995.7197 (1+)			
☑ 3010.5509 (1+)			
☑ 3026.5407 (1+)			
☑ 3036.5610 (1+)			
☑ 3052.5555 (1+)			
☑ 3234.3318 (1+)			

Select All | Select None | Search Selected | ☐ **Error tolerant** | Archive Report

1. VSPBF_MACLB **Mass:** 28963 **Score:** 69 **Matches:** 1(1) **Sequences:** 1(1)
Beta-fibrinogenase OS=Macrovipera lebetina OX=8709 PE=1 SV=1
☐ Check to include this hit in error tolerant search or archive report

Query	Observed	Mr(expt)	Mr(calc)	ppm	Miss	Score	Expect	Rank	Unique	Peptide
23	1233.6705	1232.6632	1232.6414	17.7	0	69	1.4e-007	1	U	R.AAHPWLPAQSR.T

---

2. A0A6G5ZUR6_9SAUR **Mass:** 29579 **Score:** 48 **Matches:** 1(1) **Sequences:** 1(1)
Serine protease 6 OS=Vipera anatolica senliki OX=2604287 PE=2 SV=1
☐ Check to include this hit in error tolerant search or archive report

Query	Observed	Mr(expt)	Mr(calc)	ppm	Miss	Score	Expect	Rank	Unique	Peptide
75	2113.0764	2112.0692	2112.0462	10.9	0	48	1.5e-005	1	U	R.FYCAGTLINQEWVLTAAR.C

Proteins matching the same set of peptides:
A0A6G5ZVX7_9SAUR **Mass:** 29593 **Score:** 48 **Matches:** 1(1) **Sequences:** 1(1)
Serine protease 7 OS=Vipera anatolica senliki OX=2604287 PE=2 SV=1
VASP1_VIPAA **Mass:** 22635 **Score:** 48 **Matches:** 1(1) **Sequences:** 1(1)
Snake venom serine protease VaSP1 (Fragments) OS=Vipera ammodytes ammodytes OX=8705 PE=1 SV=1
VSPH1_VIPAA **Mass:** 29593 **Score:** 48 **Matches:** 1(1) **Sequences:** 1(1)
Vaa serine proteinase homolog 1 OS=Vipera ammodytes ammodytes OX=8705 PE=1 SV=1
VSP2_MACLB **Mass:** 29559 **Score:** 48 **Matches:** 1(1) **Sequences:** 1(1)
Venom serine proteinase-like protein 2 OS=Macrovipera lebetina OX=8709 PE=2 SV=1

---

3. A0A6G7GGC6_VIPBE **Mass:** 38768 **Score:** 0 **Matches:** 1(0) **Sequences:** 1(0)
Rhodopsin (Fragment) OS=Vipera berus OX=31155 GN=RHO PE=2 SV=1
☐ Check to include this hit in error tolerant search or archive report

Query	Observed	Mr(expt)	Mr(calc)	ppm	Miss	Score	Expect	Rank	Unique	Peptide
79	2149.1282	2148.1209	2148.0673	24.9	1	4	0.37	1	U	K.SSSIYNPAIYITMNKQFR.N + Oxidation (M)

Peptide matches not assigned to protein hits: (no details means no match)

	Query	Observed	Mr(expt)	Mr(calc)	ppm	Miss	Score	Expect	Rank	Unique	Peptide
☑	1	843.4335	842.4262								
☑	2	880.4569	879.4496								
☑	3	888.4500	887.4427								
☑	4	895.3970	894.3897								
☑	5	896.4510	895.4437								
☑	6	906.4704	905.4631								
☑	7	908.5083	907.5010								
☑	8	914.4639	913.4566								
☑	9	922.4699	921.4626								
☑	10	1009.4438	1008.4366								
☑	11	1049.4386	1048.4313								
☑	12	1066.4633	1065.4560								
☑	13	1077.4646	1076.4573								
☑	14	1108.4728	1107.4655								
☑	15	1169.5911	1168.5838								
☑	16	1170.0892	1169.0819								
☑	17	1189.6611	1188.6538								
☑	18	1194.5557	1193.5484								
☑	19	1205.5920	1204.5847								
☑	20	1212.6192	1211.6119								
☑	21	1218.6543	1217.6470								
☑	22	1231.6425	1230.6352								
☑	24	1237.6639	1236.6567								
☑	25	1247.6491	1246.6419								
☑	26	1249.6639	1248.6566								
☑	27	1255.6491	1254.6418								
☑	28	1259.6864	1258.6791								
☑	29	1265.6592	1264.6519								
☑	30	1271.6156	1270.6083								
☑	31	1275.6818	1274.6745								
☑	32	1281.6541	1280.6468								
☑	33	1291.6754	1290.6682								
☑	34	1301.6959	1300.6886								

☑	35	*1304.5938*	*1303.5865*
☑	36	*1307.6705*	*1306.6633*
☑	37	*1326.6012*	*1325.5939*
☑	38	*1334.5881*	*1333.5808*
☑	39	*1340.7389*	*1339.7317*
☑	40	*1429.7302*	*1428.7229*
☑	41	*1433.8111*	*1432.8038*
☑	42	*1462.7902*	*1461.7829*
☑	43	*1477.8012*	*1476.7939*
☑	44	*1481.7954*	*1480.7881*
☑	45	*1490.7698*	*1489.7625*
☑	46	*1493.7944*	*1492.7872*
☑	47	*1498.6923*	*1497.6851*
☑	48	*1505.7880*	*1504.7807*
☑	49	*1506.2822*	*1505.2749*
☑	50	*1507.0891*	*1506.0818*
☑	51	*1509.7892*	*1508.7819*
☑	52	*1515.7405*	*1514.7332*
☑	53	*1521.8025*	*1520.7952*
☑	54	*1525.7827*	*1524.7754*
☑	55	*1534.8120*	*1533.8047*
☑	56	*1547.8074*	*1546.8001*
☑	57	*1563.8131*	*1562.8059*
☑	58	*1599.8532*	*1598.8459*
☑	59	*1606.8106*	*1605.8033*
☑	60	*1611.7753*	*1610.7681*
☑	61	*1685.8462*	*1684.8389*
☑	62	*1711.8596*	*1710.8523*
☑	63	*1723.7778*	*1722.7706*
☑	64	*1727.8452*	*1726.8379*
☑	65	*1728.9532*	*1727.9460*
☑	66	*1738.0576*	*1737.0504*
☑	67	*1776.9531*	*1775.9458*
☑	68	*1792.9390*	*1791.9317*
☑	69	*1802.9620*	*1801.9547*
☑	70	*1818.9616*	*1817.9543*
☑	71	*1821.9696*	*1820.9624*
☑	72	*1823.9891*	*1822.9818*
☑	73	*1841.9405*	*1840.9333*
☑	74	*1861.9851*	*1860.9778*
☑	76	*2129.0720*	*2128.0647*
☑	77	*2145.0632*	*2144.0559*
☑	78	*2146.8377*	*2145.8304*
☑	80	*2164.8462*	*2163.8390*
☑	81	*2184.0111*	*2183.0038*
☑	82	*2191.8581*	*2190.8508*
☑	83	*2210.8694*	*2209.8621*
☑	84	*2236.8811*	*2235.8738*
☑	85	*2252.8802*	*2251.8729*
☑	86	*2338.1486*	*2337.1413*
☑	87	*2462.2482*	*2461.2410*
☑	88	*2558.1498*	*2557.1425*
☑	89	*2609.3629*	*2608.3556*
☑	90	*2678.3299*	*2677.3226*
☑	91	*2804.1100*	*2803.1027*
☑	92	*2818.1227*	*2817.1155*
☑	93	*2995.7197*	*2994.7124*
☑	94	*3010.5509*	*3009.5436*
☑	95	*3026.5407*	*3025.5334*
☑	96	*3036.5610*	*3035.5538*
☑	97	*3052.5555*	*3051.5482*
☑	98	*3234.3318*	*3233.3245*

Search Parameters

```
Type of search         : MS/MS Ion Search
Enzyme                 : Trypsin
Fixed modifications    : Carbamidomethyl (C)
Variable modifications : Oxidation (M)
Mass values            : Monoisotopic
Protein Mass           : Unrestricted
Peptide Mass Tolerance : ± 100 ppm
Fragment Mass Tolerance: ± 0.7 Da
Max Missed Cleavages   : 1
Instrument type        : MALDI-TOF-TOF
Number of queries      : 98
```

Mascot: http://www.matrixscience.com/

Mascot Search Results

```
User          :
Email         :
Search title  : vipera_all_Lift file:\\Proteomics1\d\data\2105\210505_tliepol_1_68252-68266\68252_68266_lpardo\0_H9\1\0_H9.xml
Database      : vipera  (1414 sequences; 347452 residues)
Timestamp     : 5 May 2021 at 10:01:13 GMT
Warning       : A Peptide summary report will usually give a much clearer picture of MS/MS search results.
Top Score     : 78 for VSPBF_MACLB, Beta-fibrinogenase OS=Macrovipera lebetina OX=8709 PE=1 SV=1
```

Mascot Score Histogram

Protein score is -10*Log(P), where P is the probability that the observed match is a random event.
Protein scores greater than 44 are significant (p<0.05).
Protein scores are derived from ions scores as a non-probabilistic basis for ranking protein hits.

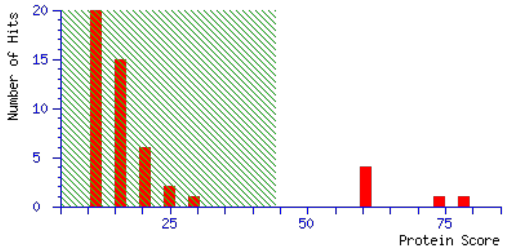

Protein Summary Report

Format As | Protein Summary (deprecated) | Help

Significance threshold p< 0.05 | Max. number of hits 20

Overview Table

Click on column header to jump to entry in results list.
Move mouse over any indicator to highlight identical peptides.
Click on an indicator to see details of individual match.
Use check boxes to select sub-set of queries for new search.

Mouse over: -Query- | -Accession- | -Sequence-

Hit:	1	2	3	4	5	6	7	8	9	10	11	12	13	14	15	16	17	18	19	20
☑ 843.4335 (1+)																				
☑ 880.4569 (1+)																				
☑ 888.4500 (1+)																				
☑ 895.3970 (1+)																				
☑ 896.4510 (1+)																				
☑ 906.4704 (1+)																				
☑ 908.5083 (1+)																				
☑ 914.4639 (1+)																				
☑ 922.4699 (1+)																				
☑ 1009.4438 (1+)																				
☑ 1049.4386 (1+)																				
☑ 1066.4633 (1+)																				
☑ 1077.4646 (1+)																				
☑ 1108.4728 (1+)																				
☑ 1169.5911 (1+)																				
☑ 1170.0892 (1+)																				
☑ 1189.6611 (1+)																				
☑ 1194.5557 (1+)																				
☑ 1205.5920 (1+)																				
☑ 1212.6192 (1+)																				
☑ 1218.6543 (1+)																				
☑ 1231.6425 (1+)																				
☑ 1233.6705 (1+)	●																			
☑ 1237.6639 (1+)																				
☑ 1247.6491 (1+)																				
☑ 1249.6639 (1+)																				
☑ 1255.6491 (1+)																				
☑ 1259.6864 (1+)																				
☑ 1265.6592 (1+)																				
☑ 1271.6156 (1+)																				
☑ 1275.6818 (1+)																				
☑ 1281.6541 (1+)																				
☑ 1291.6754 (1+)																				

☑ 1301.6959 (1+)																				
☑ 1304.5938 (1+)																				
☑ 1307.6705 (1+)																				
☑ 1326.6012 (1+)																				
☑ 1334.5881 (1+)																				
☑ 1340.7389 (1+)																				
☑ 1429.7302 (1+)																				
☑ 1433.8111 (1+)																				
☑ 1462.7902 (1+)																				
☑ 1477.8012 (1+)																				
☑ 1481.7954 (1+)																				
☑ 1490.7698 (1+)																				
☑ 1493.7944 (1+)																				
☑ 1498.6923 (1+)																				
☑ 1505.7880 (1+)																				
☑ 1506.2822 (1+)																				
☑ 1507.0891 (1+)																				
☑ 1509.7892 (1+)																				
☑ 1515.7405 (1+)																				
☑ 1521.8025 (1+)																				
☑ 1525.7827 (1+)																				
☑ 1534.8120 (1+)																				
☑ 1547.8074 (1+)																				
☑ 1563.8131 (1+)																				
☑ 1599.8532 (1+)																				
☑ 1606.8106 (1+)																				
☑ 1611.7753 (1+)																				
☑ 1685.8462 (1+)																				
☑ 1711.8596 (1+)																				
☑ 1723.7778 (1+)																				
☑ 1727.8452 (1+)																				
☑ 1728.9532 (1+)																				
☑ 1738.0576 (1+)																				
☑ 1776.9531 (1+)																				
☑ 1792.9390 (1+)																				
☑ 1802.9620 (1+)																				
☑ 1818.9616 (1+)																				
☑ 1821.9696 (1+)																				
☑ 1823.9891 (1+)																				
☑ 1841.9405 (1+)																				
☑ 1861.9851 (1+)																				
☑ 2113.0764 (1+)		●	●	●	●	●														
☑ 2129.0720 (1+)																				
☑ 2145.0632 (1+)																				
☑ 2146.8377 (1+)																				
☑ 2149.1282 (1+)																				
☑ 2164.8462 (1+)																				
☑ 2184.0111 (1+)																				
☑ 2191.8581 (1+)																				
☑ 2210.8694 (1+)																				
☑ 2236.8811 (1+)																				
☑ 2252.8802 (1+)																				
☑ 2338.1486 (1+)																				
☑ 2462.2482 (1+)																				
☑ 2558.1498 (1+)																				
☑ 2609.3629 (1+)																				
☑ 2678.3299 (1+)																				
☑ 2804.1100 (1+)																				
☑ 2818.1227 (1+)																				
☑ 2995.7197 (1+)																				
☑ 3010.5509 (1+)																				
☑ 3026.5407 (1+)																				
☑ 3036.5610 (1+)																				
☑ 3052.5555 (1+)																				
☑ 3234.3318 (1+)																				

Select All | Select None | Search Selected

Index

	Accession	Mass	Score	Description
1.	VSPBF_MACLB	28963	78	Beta-fibrinogenase OS=Macrovipera lebetina OX=8709 PE=1 SV=1
2.	VSP2_MACLB	29559	72	Venom serine proteinase-like protein 2 OS=Macrovipera lebetina OX=8709 PE=2 SV=1
3.	VASP1_VIPAA	22635	62	Snake venom serine protease VaSP1 (Fragments) OS=Vipera ammodytes ammodytes OX=8705 PE=1 SV=1

**4.**	**A0A6G5ZVX7_9SAUR**	29593	62	Serine protease 7 OS=Vipera anatolica senliki OX=2604287 PE=2 SV=1
**5.**	**A0A6G5ZUR6_9SAUR**	29579	62	Serine protease 6 OS=Vipera anatolica senliki OX=2604287 PE=2 SV=1
**6.**	**VSPH1_VIPAA**	29593	62	Vaa serine proteinase homolog 1 OS=Vipera ammodytes ammodytes OX=8705 PE=1 SV=1
**7.**	**A0A6G5ZVU6_9SAUR**	11256	29	Neurotrophin-4 (Fragment) OS=Vipera anatolica senliki OX=2604287 PE=2 SV=1
**8.**	**A0A286S1C5_9SAUR**	39024	26	E3 ubiquitin-protein ligase RAG1 (Fragment) OS=Vipera seoanei OX=110211 GN=RAG1 PE=4 SV=1
**9.**	**A0A6G5ZU93_9SAUR**	37047	24	Calumenin 2 OS=Vipera anatolica senliki OX=2604287 PE=2 SV=1
**10.**	**3SA1_DABRR**	2363	22	Cytotoxin drCT-1 (Fragment) OS=Daboia russelii OX=8707 PE=1 SV=1
**11.**	**A0A6G5ZUM9_9SAUR**	20561	21	Cholinesterase 11 (Fragment) OS=Vipera anatolica senliki OX=2604287 PE=2 SV=1
**12.**	**A0A6G5ZUN8_9SAUR**	61639	20	Atriopeptidase (Fragment) OS=Vipera anatolica senliki OX=2604287 PE=2 SV=1
**13.**	**A0A6G5ZUS7_9SAUR**	24311	19	Lactadherin 8 (Fragment) OS=Vipera anatolica senliki OX=2604287 PE=2 SV=1
**14.**	**A0A6G5ZVW2_9SAUR**	30105	19	Calumenin 4 (Fragment) OS=Vipera anatolica senliki OX=2604287 PE=2 SV=1
**15.**	**A0A6G5ZW30_9SAUR**	186570	19	Venom factor 1 OS=Vipera anatolica senliki OX=2604287 PE=2 SV=1
**16.**	**A0A6G5ZUW7_9SAUR**	22103	18	Serine protease 10 (Fragment) OS=Vipera anatolica senliki OX=2604287 PE=2 SV=1
**17.**	**A0A6G5ZU96_9SAUR**	37065	17	Calumenin 1 OS=Vipera anatolica senliki OX=2604287 PE=2 SV=1
**18.**	**A0A6G5ZVL9_9SAUR**	37117	17	Calumenin 3 OS=Vipera anatolica senliki OX=2604287 PE=2 SV=1
**19.**	**A0A286S143_VIPAS**	38971	17	E3 ubiquitin-protein ligase RAG1 (Fragment) OS=Vipera aspis OX=8706 GN=RAG1 PE=4 SV=1
**20.**	**A0A6G5ZW48_9SAUR**	86121	17	Atriopeptidase OS=Vipera anatolica senliki OX=2604287 PE=2 SV=1

Results List

1. VSPBF_MACLB **Mass:** 28963 **Score:** 78 **Expect:** 2.1e-005 **Matches:** 3

Beta-fibrinogenase OS=Macrovipera lebetina OX=8709 PE=1 SV=1

Observed	Mr(expt)	Mr(calc)	ppm	Start	End	Miss	Ions	Peptide
888.4500	887.4427	887.4211	24.3	95	101	0	---	K.FFCLSSK.N
1233.6705	1232.6632	1232.6414	17.7	175	185	0	69	R.AAHPWLPAQSR.T
1606.8106	1605.8033	1605.7855	11.1	186	200	0	---	R.TLCAGILQGGIDTCK.G

No match to: 843.4335, 880.4569, 895.3970, 896.4510, 906.4704, 908.5083, 914.4639, 922.4699, 1009.4438, 1049.4386, 1066.4633, 1077.4646, 1108.4728, 1169.5911, 1170.0892, 1189.6611, 1194.5557, 1205.5920, 1212.6192, 1218.6543, 1231.6425, 1237.6639, 1247.6491, 1249.6639, 1255.6491, 1259.6864, 1265.6592, 1271.6156, 1275.6818, 1281.6541, 1291.6754, 1301.6959, 1304.5938, 1307.6705, 1326.6012, 1334.5881, 1340.7389, 1429.7302, 1433.8111, 1462.7902, 1477.8012, 1481.7954, 1490.7698, 1493.7944, 1498.6923, 1505.7880, 1506.2822, 1507.0891, 1509.7892, 1515.7405, 1521.8025, 1525.7827, 1534.8120, 1547.8074, 1563.8131, 1599.8532, 1611.7753, 1685.8462, 1711.8596, 1723.7778, 1727.8452, 1728.9532, 1738.0576, 1776.9531, 1792.9390, 1802.9620, 1818.9616, 1821.9696, 1823.9891, 1841.9405, 1861.9851, 2113.0764, 2129.0720, 2145.0632, 2146.8377, 2149.1282, 2164.8462, 2184.0111, 2191.8581, 2210.8694, 2236.8811, 2252.8802, 2338.1486, 2462.2482, 2558.1498, 2609.3629, 2678.3299, 2804.1100, 2818.1227, 2995.7197, 3010.5509, 3026.5407, 3036.5610, 3052.5555, 3234.3318

2. VSP2_MACLB **Mass:** 29559 **Score:** 72 **Expect:** 8.5e-005 **Matches:** 7

Venom serine proteinase-like protein 2 OS=Macrovipera lebetina OX=8709 PE=2 SV=1

Observed	Mr(expt)	Mr(calc)	ppm	Start	End	Miss	Ions	Peptide
888.4500	887.4427	887.4211	24.3	98	104	0	---	K.FFCLSSK.T
1189.6611	1188.6538	1188.6325	18.0	109	117	1	---	R.WDKDIMLIR.L
1205.5920	1204.5847	1204.6274	-35.44	109	117	1	---	R.WDKDIMLIR.L + Oxidation (M)
1212.6192	1211.6119	1211.5894	18.5	83	92	0	---	K.NVPNEDQQIR.V
1249.6639	1248.6566	1248.7666	-88.10	72	82	1	---	K.NIRIILGVHSK.N
2113.0764	2112.0692	2112.0462	10.9	50	67	0	48	R.FYCAGTLINQEWVLTAAR.C
2558.1498	2557.1425	2557.1189	9.25	157	177	0	---	K.VTYPDVPHCANINMFDYSVCR.K

No match to: 843.4335, 880.4569, 895.3970, 896.4510, 906.4704, 908.5083, 914.4639, 922.4699, 1009.4438, 1049.4386, 1066.4633, 1077.4646, 1108.4728, 1169.5911, 1170.0892, 1194.5557, 1218.6543, 1231.6425, 1233.6705, 1237.6639, 1247.6491, 1255.6491, 1259.6864, 1265.6592, 1271.6156, 1275.6818, 1281.6541, 1291.6754, 1301.6959, 1304.5938, 1307.6705, 1326.6012, 1334.5881, 1340.7389, 1429.7302, 1433.8111, 1462.7902, 1477.8012, 1481.7954, 1490.7698, 1493.7944, 1498.6923, 1505.7880, 1506.2822, 1507.0891, 1509.7892, 1515.7405, 1521.8025, 1525.7827, 1534.8120, 1547.8074, 1563.8131, 1599.8532, 1606.8106, 1611.7753, 1685.8462, 1711.8596, 1723.7778, 1727.8452, 1728.9532, 1738.0576, 1776.9531, 1792.9390, 1802.9620, 1818.9616, 1821.9696, 1823.9891, 1841.9405, 1861.9851, 2129.0720, 2145.0632, 2146.8377, 2149.1282, 2164.8462, 2184.0111, 2191.8581, 2210.8694, 2236.8811, 2252.8802, 2338.1486, 2462.2482, 2609.3629, 2678.3299, 2804.1100, 2818.1227, 2995.7197, 3010.5509, 3026.5407, 3036.5610, 3052.5555, 3234.3318

3. VASP1_VIPAA **Mass:** 22635 **Score:** 62 **Expect:** 0.00081 **Matches:** 4

Snake venom serine protease VaSP1 (Fragments) OS=Vipera ammodytes ammodytes OX=8705 PE=1 SV=1

Observed	Mr(expt)	Mr(calc)	ppm	Start	End	Miss	Ions	Peptide
1189.6611	1188.6538	1188.6325	18.0	85	93	1	---	R.WDKDIMLIR.L
1205.5920	1204.5847	1204.6274	-35.44	85	93	1	---	R.WDKDIMLIR.L + Oxidation (M)
2113.0764	2112.0692	2112.0462	10.9	26	43	0	48	R.FYCAGTLINQEWVLTAAR.C
2462.2482	2461.2410	2461.2172	9.65	1	22	0	---	-.VIGGDECNINEHPFLVALHTAR.X

No match to: 843.4335, 880.4569, 888.4500, 895.3970, 896.4510, 906.4704, 908.5083, 914.4639, 922.4699, 1009.4438, 1049.4386, 1066.4633, 1077.4646, 1108.4728, 1169.5911, 1170.0892, 1194.5557, 1212.6192, 1218.6543, 1231.6425, 1233.6705, 1237.6639, 1247.6491, 1249.6639, 1255.6491, 1259.6864, 1265.6592, 1271.6156, 1275.6818, 1281.6541, 1291.6754, 1301.6959, 1304.5938, 1307.6705, 1326.6012, 1334.5881, 1340.7389, 1429.7302, 1433.8111, 1462.7902, 1477.8012, 1481.7954, 1490.7698, 1493.7944, 1498.6923, 1505.7880, 1506.2822, 1507.0891, 1509.7892, 1515.7405, 1521.8025, 1525.7827, 1534.8120, 1547.8074, 1563.8131, 1599.8532, 1606.8106, 1611.7753, 1685.8462, 1711.8596, 1723.7778, 1727.8452, 1728.9532, 1738.0576, 1776.9531, 1792.9390, 1802.9620, 1818.9616, 1821.9696, 1823.9891, 1841.9405, 1861.9851, 2129.0720, 2145.0632, 2146.8377, 2149.1282, 2164.8462, 2184.0111, 2191.8581, 2210.8694, 2236.8811, 2252.8802, 2338.1486, 2558.1498, 2609.3629, 2678.3299, 2804.1100, 2818.1227, 2995.7197, 3010.5509, 3026.5407, 3036.5610, 3052.5555, 3234.3318

4. A0A6G5ZVX7_9SAUR **Mass:** 29593 **Score:** 62 **Expect:** 0.00096 **Matches:** 7

Serine protease 7 OS=Vipera anatolica senliki OX=2604287 PE=2 SV=1

Observed	Mr(expt)	Mr(calc)	ppm	Start	End	Miss	Ions	Peptide
888.4500	887.4427	887.4211	24.3	98	104	0	---	K.FFCLSSK.T
1189.6611	1188.6538	1188.6325	18.0	109	117	1	---	R.WDKDIMLIR.L
1205.5920	1204.5847	1204.6274	-35.44	109	117	1	---	R.WDKDIMLIR.L + Oxidation (M)
1231.6425	1230.6352	1230.5299	85.6	83	92	0	---	K.NVPNEDEQMR.V
1247.6491	1246.6419	1246.5248	93.9	83	92	0	---	K.NVPNEDEQMR.V + Oxidation (M)
1249.6639	1248.6566	1248.7666	-88.10	72	82	1	---	K.NIRIILGVHSK.N
2113.0764	2112.0692	2112.0462	10.9	50	67	0	48	R.FYCAGTLINQEWVLTAAR.C

No match to: 843.4335, 880.4569, 895.3970, 896.4510, 906.4704, 908.5083, 914.4639, 922.4699, 1009.4438, 1049.4386, 1066.4633, 1077.4646, 1108.4728, 1169.5911, 1170.0892, 1194.5557, 1212.6192, 1218.6543, 1233.6705, 1237.6639, 1255.6491, 1259.6864, 1265.6592, 1271.6156, 1275.6818, 1281.6541, 1291.6754, 1301.6959, 1304.5938, 1307.6705, 1326.6012, 1334.5881, 1340.7389, 1429.7302, 1433.8111, 1462.7902, 1477.8012, 1481.7954, 1490.7698, 1493.7944, 1498.6923, 1505.7880, 1506.2822, 1507.0891, 1509.7892, 1515.7405, 1521.8025, 1525.7827, 1534.8120, 1547.8074, 1563.8131, 1599.8532, 1606.8106, 1611.7753, 1685.8462, 1711.8596, 1723.7778, 1727.8452, 1728.9532, 1738.0576, 1776.9531, 1792.9390, 1802.9620, 1818.9616, 1821.9696, 1823.9891, 1841.9405, 1861.9851, 2129.0720, 2145.0632, 2146.8377, 2149.1282, 2164.8462, 2184.0111, 2191.8581, 2210.8694, 2236.8811, 2252.8802, 2338.1486, 2462.2482, 2558.1498, 2609.3629, 2678.3299, 2804.1100, 2818.1227, 2995.7197, 3010.5509, 3026.5407, 3036.5610, 3052.5555, 3234.3318

5. A0A6G5ZUR6_9SAUR **Mass:** 29579 **Score:** 62 **Expect:** 0.00096 **Matches:** 7

Serine protease 6 OS=Vipera anatolica senliki OX=2604287 PE=2 SV=1

Observed	Mr(expt)	Mr(calc)	ppm	Start	End	Miss	Ions	Peptide
888.4500	887.4427	887.4211	24.3	98	104	0	---	K.FFCLSSK.S

1189.6611	1188.6538	1188.6325	18.0	109 - 117	1	---	R.WDKDIMLIR.L	
1205.5920	1204.5847	1204.6274	-35.44	109 - 117	1	---	R.WDKDIMLIR.L + Oxidation (M)	
1231.6425	1230.6352	1230.5299	85.6	83 - 92	0	---	K.NVPNEDEQMR.V	
1247.6491	1246.6419	1246.5248	93.9	83 - 92	0	---	K.NVPNEDEQMR.V + Oxidation (M)	
1249.6639	1248.6566	1248.7666	-88.10	72 - 82	1	---	K.NIRIILGVHSK.N	
2113.0764	2112.0692	2112.0462	10.9	50 - 67	0	48	R.FYCAGTLINQEWVLTAAR.C	

No match to: 843.4335, 880.4569, 895.3970, 896.4510, 906.4704, 908.5083, 914.4639, 922.4699, 1009.4438, 1049.4386, 1066.4633, 1077.4646, 1108.4728, 1169.5911, 1170.0892, 1194.5557, 1212.6192, 1218.6543, 1233.6705, 1237.6639, 1255.6491, 1259.6864, 1265.6592, 1271.6156, 1275.6818, 1281.6541, 1291.6754, 1301.6959, 1304.5938, 1307.6705, 1326.6012, 1334.5881, 1340.7389, 1429.7302, 1433.8111, 1462.7902, 1477.8012, 1481.7954, 1490.7698, 1493.7944, 1498.6923, 1505.7880, 1506.2822, 1507.0891, 1509.7892, 1515.7405, 1521.8025, 1525.7827, 1534.8120, 1547.8074, 1563.8131, 1599.8532, 1606.8106, 1611.7753, 1685.8462, 1711.8596, 1723.7778, 1727.8452, 1728.9532, 1738.0576, 1776.9531, 1792.9390, 1802.9620, 1818.9616, 1821.9696, 1823.9891, 1841.9405, 1861.9851, 2129.0720, 2145.0632, 2146.8377, 2149.1282, 2164.8462, 2184.0111, 2191.8581, 2210.8694, 2236.8811, 2252.8802, 2338.1486, 2462.2482, 2558.1498, 2609.3629, 2678.3299, 2804.1100, 2818.1227, 2995.7197, 3010.5509, 3026.5407, 3036.5610, 3052.5555, 3234.3318

6. VSPH1_VIPAA **Mass:** 29593 **Score:** 62 **Expect:** 0.00096 **Matches:** 7
Vaa serine proteinase homolog 1 OS=Vipera ammodytes ammodytes OX=8705 PE=1 SV=1

Observed	Mr(expt)	Mr(calc)	ppm	Start - End	Miss	Ions	Peptide
888.4500	887.4427	887.4211	24.3	98 - 104	0	---	K.FFCLSSK.T
1189.6611	1188.6538	1188.6325	18.0	109 - 117	1	---	R.WDKDIMLIR.L
1205.5920	1204.5847	1204.6274	-35.44	109 - 117	1	---	R.WDKDIMLIR.L + Oxidation (M)
1231.6425	1230.6352	1230.5299	85.6	83 - 92	0	---	K.NVPNEDEQMR.V
1247.6491	1246.6419	1246.5248	93.9	83 - 92	0	---	K.NVPNEDEQMR.V + Oxidation (M)
1249.6639	1248.6566	1248.7666	-88.10	72 - 82	1	---	K.NIRIILGVHSK.N
2113.0764	2112.0692	2112.0462	10.9	50 - 67	0	48	R.FYCAGTLINQEWVLTAAR.C

No match to: 843.4335, 880.4569, 895.3970, 896.4510, 906.4704, 908.5083, 914.4639, 922.4699, 1009.4438, 1049.4386, 1066.4633, 1077.4646, 1108.4728, 1169.5911, 1170.0892, 1194.5557, 1212.6192, 1218.6543, 1233.6705, 1237.6639, 1255.6491, 1259.6864, 1265.6592, 1271.6156, 1275.6818, 1281.6541, 1291.6754, 1301.6959, 1304.5938, 1307.6705, 1326.6012, 1334.5881, 1340.7389, 1429.7302, 1433.8111, 1462.7902, 1477.8012, 1481.7954, 1490.7698, 1493.7944, 1498.6923, 1505.7880, 1506.2822, 1507.0891, 1509.7892, 1515.7405, 1521.8025, 1525.7827, 1534.8120, 1547.8074, 1563.8131, 1599.8532, 1606.8106, 1611.7753, 1685.8462, 1711.8596, 1723.7778, 1727.8452, 1728.9532, 1738.0576, 1776.9531, 1792.9390, 1802.9620, 1818.9616, 1821.9696, 1823.9891, 1841.9405, 1861.9851, 2129.0720, 2145.0632, 2146.8377, 2149.1282, 2164.8462, 2184.0111, 2191.8581, 2210.8694, 2236.8811, 2252.8802, 2338.1486, 2462.2482, 2558.1498, 2609.3629, 2678.3299, 2804.1100, 2818.1227, 2995.7197, 3010.5509, 3026.5407, 3036.5610, 3052.5555, 3234.3318

7. A0A6G5ZVU6_9SAUR **Mass:** 11256 **Score:** 29 **Expect:** 1.8 **Matches:** 4
Neurotrophin-4 (Fragment) OS=Vipera anatolica senliki OX=2604287 PE=2 SV=1

Observed	Mr(expt)	Mr(calc)	ppm	Start - End	Miss	Ions	Peptide
1205.5920	1204.5847	1204.5078	63.9	75 - 86	0	---	K.CNPAGGTVGGCR.G
1231.6425	1230.6352	1230.5589	62.0	1 - 12	0	---	-.DSSTQPGQAANR.T
1841.9405	1840.9333	1840.7972	73.9	27 - 42	0	---	R.GEMSVCDSVNVWVTDK.R + Oxidation (M)
2149.1282	2148.1209	2147.9517	78.8	68 - 86	1	---	R.QYFFETKCNPAGGTVGGCR.G

No match to: 843.4335, 880.4569, 888.4500, 895.3970, 896.4510, 906.4704, 908.5083, 914.4639, 922.4699, 1009.4438, 1049.4386, 1066.4633, 1077.4646, 1108.4728, 1169.5911, 1170.0892, 1189.6611, 1194.5557, 1212.6192, 1218.6543, 1233.6705, 1237.6639, 1247.6491, 1249.6639, 1255.6491, 1259.6864, 1265.6592, 1271.6156, 1275.6818, 1281.6541, 1291.6754, 1301.6959, 1304.5938, 1307.6705, 1326.6012, 1334.5881, 1340.7389, 1429.7302, 1433.8111, 1462.7902, 1477.8012, 1481.7954, 1490.7698, 1493.7944, 1498.6923, 1505.7880, 1506.2822, 1507.0891, 1509.7892, 1515.7405, 1521.8025, 1525.7827, 1534.8120, 1547.8074, 1563.8131, 1599.8532, 1606.8106, 1611.7753, 1685.8462, 1711.8596, 1723.7778, 1727.8452, 1728.9532, 1738.0576, 1776.9531, 1792.9390, 1802.9620, 1818.9616, 1821.9696, 1823.9891, 1861.9851, 2113.0764, 2129.0720, 2145.0632, 2146.8377, 2164.8462, 2184.0111, 2191.8581, 2210.8694, 2236.8811, 2252.8802, 2338.1486, 2462.2482, 2558.1498, 2609.3629, 2678.3299, 2804.1100, 2818.1227, 2995.7197, 3010.5509, 3026.5407, 3036.5610, 3052.5555, 3234.3318

8. A0A286S1C5_9SAUR **Mass:** 39024 **Score:** 26 **Expect:** 3.5 **Matches:** 9
E3 ubiquitin-protein ligase RAG1 (Fragment) OS=Vipera seoanei OX=110211 GN=RAG1 PE=4 SV=1

Observed	Mr(expt)	Mr(calc)	ppm	Start - End	Miss	Ions	Peptide
843.4335	842.4262	842.4974	-84.51	207 - 213	1	---	K.VKQLNNK.S
1049.4386	1048.4313	1048.4760	-42.64	46 - 53	0	---	K.EVFHMSQR.E + Oxidation (M)
1247.6491	1246.6419	1246.5652	61.5	315 - 324	0	---	K.GCHEEVFLEK.Y
1259.6864	1258.6791	1258.5843	75.3	155 - 164	0	---	K.DPVEWHPHSR.S
1462.7902	1461.7829	1461.7068	52.1	219 - 230	1	---	K.MSNCKQIHLSTK.V + Oxidation (M)
1599.8532	1598.8459	1598.8021	27.4	62 - 74	1	---	K.LQHLCRICGDSLK.T
2149.1282	2148.1209	2148.0004	56.1	1 - 20	1	---	-.ANSAKENMTGSLDITDEQPK.A
2164.8462	2163.8390	2163.9954	-72.27	1 - 20	1	---	-.ANSAKENMTGSLDITDEQPK.A + Oxidation (M)
2609.3629	2608.3556	2608.1700	71.2	116 - 136	1	---	K.IDVRGDIDTTHPTHFCHNCWK.V

No match to: 880.4569, 888.4500, 895.3970, 896.4510, 906.4704, 908.5083, 914.4639, 922.4699, 1009.4438, 1066.4633, 1077.4646, 1108.4728, 1169.5911, 1170.0892, 1189.6611, 1194.5557, 1205.5920, 1212.6192, 1218.6543, 1231.6425, 1233.6705, 1237.6639, 1249.6639, 1255.6491, 1265.6592, 1271.6156, 1275.6818, 1281.6541, 1291.6754, 1301.6959, 1304.5938, 1307.6705, 1326.6012, 1334.5881, 1340.7389, 1429.7302, 1433.8111, 1477.8012, 1481.7954, 1490.7698, 1493.7944, 1498.6923, 1505.7880, 1506.2822, 1507.0891, 1509.7892, 1515.7405, 1521.8025, 1525.7827, 1534.8120, 1547.8074, 1563.8131, 1606.8106, 1611.7753, 1685.8462, 1711.8596, 1723.7778, 1727.8452, 1728.9532, 1738.0576, 1776.9531, 1792.9390, 1802.9620, 1818.9616, 1821.9696, 1823.9891, 1841.9405, 1861.9851, 2113.0764, 2129.0720, 2145.0632, 2146.8377, 2184.0111, 2191.8581, 2210.8694, 2236.8811, 2252.8802, 2338.1486, 2462.2482, 2558.1498, 2678.3299, 2804.1100, 2818.1227, 2995.7197, 3010.5509, 3026.5407, 3036.5610, 3052.5555, 3234.3318

9. A0A6G5ZU93_9SAUR **Mass:** 37047 **Score:** 24 **Expect:** 6 **Matches:** 8
Calumenin 2 OS=Vipera anatolica senliki OX=2604287 PE=2 SV=1

Observed	Mr(expt)	Mr(calc)	ppm	Start - End	Miss	Ions	Peptide
880.4569	879.4496	879.4008	55.5	251 - 257	1	---	K.MDKEETK.D
896.4510	895.4437	895.3957	53.6	251 - 257	1	---	K.MDKEETK.D + Oxidation (M)
1066.4633	1065.4560	1065.4978	-39.26	87 - 96	0	---	K.DGFATEGELK.D
1233.6705	1232.6632	1232.6037	48.3	274 - 283	1	---	R.HLVYESDKDK.D
1247.6491	1246.6419	1246.6591	-13.85	75 - 86	1	---	R.LGAMVGKIDTDK.D
1259.6864	1258.6791	1258.6769	1.79	164 - 175	1	---	K.IADKDGDLIATK.E
1340.7389	1339.7317	1339.6521	59.4	234 - 243	1	---	K.TEREQFVEFR.D
1534.8120	1533.8047	1533.7232	53.1	192 - 204	0	---	K.DIVVQETMEDIDK.N

No match to: 843.4335, 888.4500, 895.3970, 906.4704, 908.5083, 914.4639, 922.4699, 1009.4438, 1049.4386, 1077.4646, 1108.4728, 1169.5911, 1170.0892, 1189.6611, 1194.5557, 1205.5920, 1212.6192, 1218.6543, 1231.6425, 1237.6639, 1249.6639, 1255.6491, 1265.6592, 1271.6156, 1275.6818, 1281.6541, 1291.6754, 1301.6959, 1304.5938, 1307.6705, 1326.6012, 1334.5881, 1429.7302, 1433.8111, 1462.7902, 1477.8012, 1481.7954, 1490.7698, 1493.7944, 1498.6923, 1505.7880, 1506.2822, 1507.0891, 1509.7892, 1515.7405, 1521.8025, 1525.7827, 1547.8074, 1563.8131, 1599.8532, 1606.8106, 1611.7753, 1685.8462, 1711.8596, 1723.7778, 1727.8452, 1728.9532, 1738.0576, 1776.9531, 1792.9390, 1802.9620, 1818.9616, 1821.9696, 1823.9891, 1841.9405, 1861.9851, 2113.0764, 2129.0720, 2145.0632, 2146.8377, 2149.1282, 2164.8462, 2184.0111, 2191.8581, 2210.8694, 2236.8811, 2252.8802, 2338.1486, 2462.2482, 2558.1498, 2609.3629, 2678.3299, 2804.1100, 2818.1227, 2995.7197, 3010.5509, 3026.5407, 3036.5610, 3052.5555, 3234.3318

10. 3SA1_DABRR **Mass:** 2363 **Score:** 22 **Expect:** 9.8 **Matches:** 2

Cytotoxin drCT-1 (Fragment) OS=Daboia russelii OX=8707 PE=1 SV=1

Observed	Mr(expt)	Mr(calc)	ppm	Start	End	Miss	Ions	Peptide
1281.6541	1280.6468	1280.6951	-37.73	3 -	12	1	---	K.CNKLVPLFYK.T
1493.7944	1492.7872	1492.8112	-16.11	6 -	18	1	---	K.LVPLFYKTCPAGK.N

No match to: 843.4335, 880.4569, 888.4500, 895.3970, 896.4510, 906.4704, 908.5083, 914.4639, 922.4699, 1009.4438, 1049.4386, 1066.4633, 1077.4646, 1108.4728, 1169.5911, 1170.0892, 1189.6611, 1194.5557, 1205.5920, 1212.6192, 1218.6543, 1231.6425, 1233.6705, 1237.6639, 1247.6491, 1249.6639, 1255.6491, 1259.6864, 1265.6592, 1271.6156, 1275.6818, 1291.6754, 1301.6959, 1304.5938, 1307.6705, 1326.6012, 1334.5881, 1340.7389, 1429.7302, 1433.8111, 1462.7902, 1477.8012, 1481.7954, 1490.7698, 1498.6923, 1505.7880, 1506.2822, 1507.0891, 1509.7892, 1515.7405, 1521.8025, 1525.7827, 1534.8120, 1547.8074, 1563.8131, 1599.8532, 1606.8106, 1611.7753, 1685.8462, 1711.8596, 1723.7778, 1727.8452, 1728.9532, 1738.0576, 1776.9531, 1792.9390, 1802.9620, 1818.9616, 1821.9696, 1823.9891, 1841.9405, 1861.9851, 2113.0764, 2129.0720, 2145.0632, 2146.8377, 2149.1282, 2164.8462, 2184.0111, 2191.8581, 2210.8694, 2236.8811, 2252.8802, 2338.1486, 2462.2482, 2558.1498, 2609.3629, 2678.3299, 2804.1100, 2818.1227, 2995.7197, 3010.5509, 3026.5407, 3036.5610, 3052.5555, 3234.3318

11. A0A6G5ZUM9_9SAUR **Mass:** 20561 **Score:** 21 **Expect:** 12 **Matches:** 4

Cholinesterase 11 (Fragment) OS=Vipera anatolica senliki OX=2604287 PE=2 SV=1

Observed	Mr(expt)	Mr(calc)	ppm	Start	End	Miss	Ions	Peptide
1212.6192	1211.6119	1211.6696	-47.60	34 -	44	0	---	R.VIACPLIEAAR.Y
1490.7698	1489.7625	1489.6846	52.3	106 -	116	1	---	R.KMMQYWAEFTR.T
1515.7405	1514.7332	1514.7266	4.36	22 -	33	1	---	K.YRSALSHFYTDR.V
2338.1486	2337.1413	2337.1601	-8.04	1 -	21	1	---	-.TEDFVQTIALRFSEGNQSPAK.Y

No match to: 843.4335, 880.4569, 888.4500, 895.3970, 896.4510, 906.4704, 908.5083, 914.4639, 922.4699, 1009.4438, 1049.4386, 1066.4633, 1077.4646, 1108.4728, 1169.5911, 1170.0892, 1189.6611, 1194.5557, 1205.5920, 1218.6543, 1231.6425, 1233.6705, 1237.6639, 1247.6491, 1249.6639, 1255.6491, 1259.6864, 1265.6592, 1271.6156, 1275.6818, 1281.6541, 1291.6754, 1301.6959, 1304.5938, 1307.6705, 1326.6012, 1334.5881, 1340.7389, 1429.7302, 1433.8111, 1462.7902, 1477.8012, 1481.7954, 1493.7944, 1498.6923, 1505.7880, 1506.2822, 1507.0891, 1509.7892, 1521.8025, 1525.7827, 1534.8120, 1547.8074, 1563.8131, 1599.8532, 1606.8106, 1611.7753, 1685.8462, 1711.8596, 1723.7778, 1727.8452, 1728.9532, 1738.0576, 1776.9531, 1792.9390, 1802.9620, 1818.9616, 1821.9696, 1823.9891, 1841.9405, 1861.9851, 2113.0764, 2129.0720, 2145.0632, 2146.8377, 2149.1282, 2164.8462, 2184.0111, 2191.8581, 2210.8694, 2236.8811, 2252.8802, 2462.2482, 2558.1498, 2609.3629, 2678.3299, 2804.1100, 2818.1227, 2995.7197, 3010.5509, 3026.5407, 3036.5610, 3052.5555, 3234.3318

12. A0A6G5ZUN8_9SAUR **Mass:** 61639 **Score:** 20 **Expect:** 15 **Matches:** 7

Atriopeptidase (Fragment) OS=Vipera anatolica senliki OX=2604287 PE=2 SV=1

Observed	Mr(expt)	Mr(calc)	ppm	Start	End	Miss	Ions	Peptide
922.4699	921.4626	921.4417	22.8	386 -	392	1	---	K.DTRNNFR.K
1108.4728	1107.4655	1107.5342	-62.08	59 -	68	1	---	K.TSDCIKSAAR.I
1281.6541	1280.6468	1280.6513	-3.55	184 -	194	0	---	K.SIAELNFQFGR.K
1301.6959	1300.6886	1300.6155	56.2	372 -	382	0	---	R.YIMDMVSSLSR.E
1685.8462	1684.8389	1684.8321	4.05	359 -	371	1	---	K.YSAREIQNYISWR.Y
1821.9696	1820.9624	1820.8284	73.6	4 -	19	0	---	K.SESQMDITEMNSPKPK.R
2184.0111	2183.0038	2182.9908	5.96	1 -	19	1	---	-.MAKSESQMDITEMNSPKPK.R + 2 Oxidation (M)

No match to: 843.4335, 880.4569, 888.4500, 895.3970, 896.4510, 906.4704, 908.5083, 914.4639, 1009.4438, 1049.4386, 1066.4633, 1077.4646, 1169.5911, 1170.0892, 1189.6611, 1194.5557, 1205.5920, 1212.6192, 1218.6543, 1231.6425, 1233.6705, 1237.6639, 1247.6491, 1249.6639, 1255.6491, 1259.6864, 1265.6592, 1271.6156, 1275.6818, 1291.6754, 1304.5938, 1307.6705, 1326.6012, 1334.5881, 1340.7389, 1429.7302, 1433.8111, 1462.7902, 1477.8012, 1481.7954, 1490.7698, 1493.7944, 1498.6923, 1505.7880, 1506.2822, 1507.0891, 1509.7892, 1515.7405, 1521.8025, 1525.7827, 1534.8120, 1547.8074, 1563.8131, 1599.8532, 1606.8106, 1611.7753, 1711.8596, 1723.7778, 1727.8452, 1728.9532, 1738.0576, 1776.9531, 1792.9390, 1802.9620, 1818.9616, 1823.9891, 1841.9405, 1861.9851, 2113.0764, 2129.0720, 2145.0632, 2146.8377, 2149.1282, 2164.8462, 2191.8581, 2210.8694, 2236.8811, 2252.8802, 2338.1486, 2462.2482, 2558.1498, 2609.3629, 2678.3299, 2804.1100, 2818.1227, 2995.7197, 3010.5509, 3026.5407, 3036.5610, 3052.5555, 3234.3318

13. A0A6G5ZUS7_9SAUR **Mass:** 24311 **Score:** 19 **Expect:** 18 **Matches:** 6

Lactadherin 8 (Fragment) OS=Vipera anatolica senliki OX=2604287 PE=2 SV=1

Observed	Mr(expt)	Mr(calc)	ppm	Start	End	Miss	Ions	Peptide
1009.4438	1008.4366	1008.4876	-50.63	1 -	9	0	---	-.QIFSGNTDK.Y
1233.6705	1232.6632	1232.5761	70.7	177 -	186	0	---	K.NMFDVPFHAR.F
1249.6639	1248.6566	1248.5710	68.6	177 -	186	0	---	K.NMFDVPFHAR.F + Oxidation (M)
1802.9620	1801.9547	1801.8934	34.1	81 -	95	1	---	K.MTWHPYLARLDNTGK.S
1818.9616	1817.9543	1817.8883	36.3	81 -	95	1	---	K.MTWHPYLARLDNTGK.S + Oxidation (M)
2338.1486	2337.1413	2337.2593	-50.50	96 -	116	0	---	K.SNAWAALTNKPGAWLQIDLLR.V

No match to: 843.4335, 880.4569, 888.4500, 895.3970, 896.4510, 906.4704, 908.5083, 914.4639, 922.4699, 1049.4386, 1066.4633, 1077.4646, 1108.4728, 1169.5911, 1170.0892, 1189.6611, 1194.5557, 1205.5920, 1212.6192, 1218.6543, 1231.6425, 1237.6639, 1247.6491, 1255.6491, 1259.6864, 1265.6592, 1271.6156, 1275.6818, 1281.6541, 1291.6754, 1301.6959, 1304.5938, 1307.6705, 1326.6012, 1334.5881, 1340.7389, 1429.7302, 1433.8111, 1462.7902, 1477.8012, 1481.7954, 1490.7698, 1493.7944, 1498.6923, 1505.7880, 1506.2822, 1507.0891, 1509.7892, 1515.7405, 1521.8025, 1525.7827, 1534.8120, 1547.8074, 1563.8131, 1599.8532, 1606.8106, 1611.7753, 1685.8462, 1711.8596, 1723.7778, 1727.8452, 1728.9532, 1738.0576, 1776.9531, 1792.9390, 1821.9696, 1823.9891, 1841.9405, 1861.9851, 2113.0764, 2129.0720, 2145.0632, 2146.8377, 2149.1282, 2164.8462, 2184.0111, 2191.8581, 2210.8694, 2236.8811, 2252.8802, 2462.2482, 2558.1498, 2609.3629, 2678.3299, 2804.1100, 2818.1227, 2995.7197, 3010.5509, 3026.5407, 3036.5610, 3052.5555, 3234.3318

14. A0A6G5ZVW2_9SAUR **Mass:** 30105 **Score:** 19 **Expect:** 19 **Matches:** 7

Calumenin 4 (Fragment) OS=Vipera anatolica senliki OX=2604287 PE=2 SV=1

Observed	Mr(expt)	Mr(calc)	ppm	Start	End	Miss	Ions	Peptide
880.4569	879.4496	879.4008	55.5	189 -	195	1	---	K.MDKEETK.D
896.4510	895.4437	895.3957	53.6	189 -	195	1	---	K.MDKEETK.D + Oxidation (M)
1233.6705	1232.6632	1232.6037	48.3	212 -	221	1	---	R.HLVYESDKDK.D
1259.6864	1258.6791	1258.6769	1.79	102 -	113	1	---	K.IADKDGDLIATK.E
1265.6592	1264.6519	1264.6200	25.2	43 -	51	1	---	K.RWIYEDVER.Q
1340.7389	1339.7317	1339.6521	59.4	172 -	181	1	---	K.TEREQFVEFR.D
1534.8120	1533.8047	1533.7232	53.1	130 -	142	0	---	K.DIVVQETMEDIDK.N

No match to: 843.4335, 888.4500, 895.3970, 906.4704, 908.5083, 914.4639, 922.4699, 1009.4438, 1049.4386, 1066.4633, 1077.4646, 1108.4728, 1169.5911, 1170.0892, 1189.6611, 1194.5557, 1205.5920, 1212.6192, 1218.6543, 1231.6425, 1237.6639, 1247.6491, 1249.6639, 1255.6491, 1271.6156, 1275.6818, 1281.6541, 1291.6754, 1301.6959, 1304.5938, 1307.6705, 1326.6012, 1334.5881, 1429.7302, 1433.8111, 1462.7902, 1477.8012, 1481.7954, 1490.7698, 1493.7944, 1498.6923, 1505.7880, 1506.2822, 1507.0891, 1509.7892, 1515.7405, 1521.8025, 1525.7827, 1547.8074, 1563.8131, 1599.8532, 1606.8106, 1611.7753, 1685.8462, 1711.8596, 1723.7778, 1727.8452, 1728.9532, 1738.0576, 1776.9531, 1792.9390, 1802.9620, 1818.9616, 1821.9696, 1823.9891, 1841.9405, 1861.9851, 2113.0764, 2129.0720, 2145.0632, 2146.8377, 2149.1282, 2164.8462, 2184.0111, 2191.8581, 2210.8694, 2236.8811, 2252.8802, 2338.1486, 2462.2482, 2558.1498, 2609.3629, 2678.3299, 2804.1100, 2818.1227, 2995.7197, 3010.5509, 3026.5407, 3036.5610, 3052.5555, 3234.3318

15. A0A6G5ZW30_9SAUR **Mass:** 186570 **Score:** 19 **Expect:** 19 **Matches:** 12

Venom factor 1 OS=Vipera anatolica senliki OX=2604287 PE=2 SV=1

Observed	Mr(expt)	Mr(calc)	ppm	Start	End	Miss	Ions	Peptide
908.5083	907.5010	907.4875	14.8	1573 -	1579	1	---	R.QYVSQRK.C

1212.6192	1211.6119	1211.6550	-35.55	591	600	1	---	K.AVYVLNDKYK.I
1304.5938	1303.5865	1303.6884	-78.16	674	684	1	---	K.VAEFQDQALRK.C
1340.7389	1339.7317	1339.6078	92.5	703	713	1	---	R.EKYIQEGDACK.A
1429.7302	1428.7229	1428.7712	-33.77	931	943	0	---	K.DIVTVIELDPSTK.G
1462.7902	1461.7829	1461.7576	17.3	671	683	1	---	K.ASKVAEFQDQALR.K
1611.7753	1610.7681	1610.7756	-4.70	546	560	1	---	K.DTCMGTLIVKGASSR.D + Oxidation (M)
1711.8596	1710.8523	1710.9345	-48.03	821	834	1	---	K.DFFIDLRLPYSVVK.N
2129.0720	2128.0647	2128.1124	-22.41	971	990	1	---	K.ISVQGDRVAQIIENSIDGSK.L
2184.0111	2183.0038	2183.0932	-40.96	1422	1440	1	---	K.FEIDNNMAQKGAVVIYLDK.V + Oxidation (M)
3026.5407	3025.5334	3025.5872	-17.79	1171	1197	1	---	K.YEKLQRPYTTALTAYALAAVGLLNDDR.V
3234.3318	3233.3245	3233.5360	-65.40	1382	1410	0	---	R.YLGEVDSTMTIIDVSMLTGFLPDAEDLTR.L + 2 Oxidation (M)

No match to: 843.4335, 880.4569, 888.4500, 895.3970, 896.4510, 906.4704, 914.4639, 922.4699, 1009.4438, 1049.4386, 1066.4633, 1077.4646, 1108.4728, 1169.5911, 1170.0892, 1189.6611, 1194.5557, 1205.5920, 1218.6543, 1231.6425, 1233.6705, 1237.6639, 1247.6491, 1249.6639, 1255.6491, 1259.6864, 1265.6592, 1271.6156, 1275.6818, 1281.6541, 1291.6754, 1301.6959, 1307.6705, 1326.6012, 1334.5881, 1433.8111, 1477.8012, 1481.7954, 1490.7698, 1493.7944, 1498.6923, 1505.7880, 1506.2822, 1507.0891, 1509.7892, 1515.7405, 1521.8025, 1525.7827, 1534.8120, 1547.8074, 1563.8131, 1599.8532, 1606.8106, 1685.8462, 1723.7778, 1727.8452, 1728.9532, 1738.0576, 1776.9531, 1792.9390, 1802.9620, 1818.9616, 1821.9696, 1823.9891, 1841.9405, 1861.9851, 2113.0764, 2145.0632, 2146.8377, 2149.1282, 2164.8462, 2191.8581, 2210.8694, 2236.8811, 2252.8802, 2338.1486, 2462.2482, 2558.1498, 2609.3629, 2678.3299, 2804.1100, 2818.1227, 2995.7197, 3010.5509, 3036.5610, 3052.5555

16. A0A6G5ZUW7_9SAUR **Mass:** 22103 **Score:** 18 **Expect:** 23 **Matches:** 6
Serine protease 10 (Fragment) OS=Vipera anatolica senliki OX=2604287 PE=2 SV=1

Observed	Mr(expt)	Mr(calc)	ppm	Start	End	Miss	Ions	Peptide
888.4500	887.4427	887.4211	24.3	95	101	0	---	K.FFCLSSK.S
1189.6611	1188.6538	1188.6325	18.0	106	114	1	---	R.WDKDIMLIR.L
1205.5920	1204.5847	1204.6274	-35.44	106	114	1	---	R.WDKDIMLIR.L + Oxidation (M)
1231.6425	1230.6352	1230.5299	85.6	80	89	0	---	K.NVPNEDEQMR.V
1247.6491	1246.6419	1246.5248	93.9	80	89	0	---	K.NVPNEDEQMR.V + Oxidation (M)
1249.6639	1248.6566	1248.7666	-88.10	69	79	1	---	K.NIRIILGVHSK.N

No match to: 843.4335, 880.4569, 895.3970, 896.4510, 906.4704, 908.5083, 914.4639, 922.4699, 1009.4438, 1049.4386, 1066.4633, 1077.4646, 1108.4728, 1169.5911, 1170.0892, 1194.5557, 1212.6192, 1218.6543, 1233.6705, 1237.6639, 1255.6491, 1259.6864, 1265.6592, 1271.6156, 1275.6818, 1281.6541, 1291.6754, 1301.6959, 1304.5938, 1307.6705, 1326.6012, 1334.5881, 1340.7389, 1429.7302, 1433.8111, 1462.7902, 1477.8012, 1481.7954, 1490.7698, 1493.7944, 1498.6923, 1505.7880, 1506.2822, 1507.0891, 1509.7892, 1515.7405, 1521.8025, 1525.7827, 1534.8120, 1547.8074, 1563.8131, 1599.8532, 1606.8106, 1611.7753, 1685.8462, 1711.8596, 1723.7778, 1727.8452, 1728.9532, 1738.0576, 1776.9531, 1792.9390, 1802.9620, 1818.9616, 1821.9696, 1823.9891, 1841.9405, 1861.9851, 2113.0764, 2129.0720, 2145.0632, 2146.8377, 2149.1282, 2164.8462, 2184.0111, 2191.8581, 2210.8694, 2236.8811, 2252.8802, 2338.1486, 2462.2482, 2558.1498, 2609.3629, 2678.3299, 2804.1100, 2818.1227, 2995.7197, 3010.5509, 3026.5407, 3036.5610, 3052.5555, 3234.3318

17. A0A6G5ZU96_9SAUR **Mass:** 37065 **Score:** 17 **Expect:** 26 **Matches:** 7
Calumenin 1 OS=Vipera anatolica senliki OX=2604287 PE=2 SV=1

Observed	Mr(expt)	Mr(calc)	ppm	Start	End	Miss	Ions	Peptide
880.4569	879.4496	879.4008	55.5	251	257	1	---	K.MDKEETK.D
896.4510	895.4437	895.3957	53.6	251	257	1	---	K.MDKEETK.D + Oxidation (M)
1233.6705	1232.6632	1232.6037	48.3	274	283	1	---	R.HLVYESDKDK.D
1259.6864	1258.6791	1258.6769	1.79	164	175	1	---	K.IADKDGDLIATK.E
1265.6592	1264.6519	1264.6200	25.2	105	113	1	---	K.RWIYEDVER.Q
1340.7389	1339.7317	1339.6521	59.4	234	243	1	---	K.TEREQFVEFR.D
1534.8120	1533.8047	1533.7232	53.1	192	204	0	---	K.DIVVQETMEDIDK.N

No match to: 843.4335, 888.4500, 895.3970, 906.4704, 908.5083, 914.4639, 922.4699, 1009.4438, 1049.4386, 1066.4633, 1077.4646, 1108.4728, 1169.5911, 1170.0892, 1189.6611, 1194.5557, 1205.5920, 1212.6192, 1218.6543, 1231.6425, 1237.6639, 1247.6491, 1249.6639, 1255.6491, 1271.6156, 1275.6818, 1281.6541, 1291.6754, 1301.6959, 1304.5938, 1307.6705, 1326.6012, 1334.5881, 1429.7302, 1433.8111, 1462.7902, 1477.8012, 1481.7954, 1490.7698, 1493.7944, 1498.6923, 1505.7880, 1506.2822, 1507.0891, 1509.7892, 1515.7405, 1521.8025, 1525.7827, 1547.8074, 1563.8131, 1599.8532, 1606.8106, 1611.7753, 1685.8462, 1711.8596, 1723.7778, 1727.8452, 1728.9532, 1738.0576, 1776.9531, 1792.9390, 1802.9620, 1818.9616, 1821.9696, 1823.9891, 1841.9405, 1861.9851, 2113.0764, 2129.0720, 2145.0632, 2146.8377, 2149.1282, 2164.8462, 2184.0111, 2191.8581, 2210.8694, 2236.8811, 2252.8802, 2338.1486, 2462.2482, 2558.1498, 2609.3629, 2678.3299, 2804.1100, 2818.1227, 2995.7197, 3010.5509, 3026.5407, 3036.5610, 3052.5555, 3234.3318

18. A0A6G5ZVL9_9SAUR **Mass:** 37117 **Score:** 17 **Expect:** 26 **Matches:** 7
Calumenin 3 OS=Vipera anatolica senliki OX=2604287 PE=2 SV=1

Observed	Mr(expt)	Mr(calc)	ppm	Start	End	Miss	Ions	Peptide
880.4569	879.4496	879.4008	55.5	251	257	1	---	K.MDKEETK.D
896.4510	895.4437	895.3957	53.6	251	257	1	---	K.MDKEETK.D + Oxidation (M)
1233.6705	1232.6632	1232.6037	48.3	274	283	1	---	R.HLVYESDKDK.D
1259.6864	1258.6791	1258.6769	1.79	164	175	1	---	K.IADKDGDLIATK.E
1265.6592	1264.6519	1264.6200	25.2	105	113	1	---	K.RWIYEDVER.Q
1340.7389	1339.7317	1339.6521	59.4	234	243	1	---	K.TEREQFVEFR.D
1534.8120	1533.8047	1533.7232	53.1	192	204	0	---	K.DIVVQETMEDIDK.N

No match to: 843.4335, 888.4500, 895.3970, 906.4704, 908.5083, 914.4639, 922.4699, 1009.4438, 1049.4386, 1066.4633, 1077.4646, 1108.4728, 1169.5911, 1170.0892, 1189.6611, 1194.5557, 1205.5920, 1212.6192, 1218.6543, 1231.6425, 1237.6639, 1247.6491, 1249.6639, 1255.6491, 1271.6156, 1275.6818, 1281.6541, 1291.6754, 1301.6959, 1304.5938, 1307.6705, 1326.6012, 1334.5881, 1429.7302, 1433.8111, 1462.7902, 1477.8012, 1481.7954, 1490.7698, 1493.7944, 1498.6923, 1505.7880, 1506.2822, 1507.0891, 1509.7892, 1515.7405, 1521.8025, 1525.7827, 1547.8074, 1563.8131, 1599.8532, 1606.8106, 1611.7753, 1685.8462, 1711.8596, 1723.7778, 1727.8452, 1728.9532, 1738.0576, 1776.9531, 1792.9390, 1802.9620, 1818.9616, 1821.9696, 1823.9891, 1841.9405, 1861.9851, 2113.0764, 2129.0720, 2145.0632, 2146.8377, 2149.1282, 2164.8462, 2184.0111, 2191.8581, 2210.8694, 2236.8811, 2252.8802, 2338.1486, 2462.2482, 2558.1498, 2609.3629, 2678.3299, 2804.1100, 2818.1227, 2995.7197, 3010.5509, 3026.5407, 3036.5610, 3052.5555, 3234.3318

19. A0A286S143_VIPAS **Mass:** 38971 **Score:** 17 **Expect:** 26 **Matches:** 5
E3 ubiquitin-protein ligase RAG1 (Fragment) OS=Vipera aspis OX=8706 GN=RAG1 PE=4 SV=1

Observed	Mr(expt)	Mr(calc)	ppm	Start	End	Miss	Ions	Peptide
843.4335	842.4262	842.4974	-84.51	207	213	1	---	K.VKQLNNK.S
1049.4386	1048.4313	1048.4760	-42.64	46	53	0	---	K.EVFHMSQR.E + Oxidation (M)
1247.6491	1246.6419	1246.5652	61.5	315	324	0	---	K.GCHEEVFLEK.Y
1599.8532	1598.8459	1598.8021	27.4	62	74	1	---	K.LQHLCRICGDSLK.T
2609.3629	2608.3556	2608.1700	71.2	116	136	1	---	K.IDVRGDIDTTHPTHFCHNCWK.V

No match to: 880.4569, 888.4500, 895.3970, 896.4510, 906.4704, 908.5083, 914.4639, 922.4699, 1009.4438, 1066.4633, 1077.4646, 1108.4728, 1169.5911, 1170.0892, 1189.6611, 1194.5557, 1205.5920, 1212.6192, 1218.6543, 1231.6425, 1233.6705, 1237.6639, 1249.6639, 1255.6491, 1259.6864, 1265.6592, 1271.6156, 1275.6818, 1281.6541, 1291.6754, 1301.6959, 1304.5938, 1307.6705, 1326.6012, 1334.5881, 1340.7389, 1429.7302, 1433.8111, 1462.7902, 1477.8012, 1481.7954, 1490.7698, 1493.7944, 1498.6923, 1505.7880, 1506.2822, 1507.0891, 1509.7892, 1515.7405, 1521.8025, 1525.7827, 1534.8120, 1547.8074, 1563.8131, 1606.8106, 1611.7753, 1685.8462, 1711.8596, 1723.7778, 1727.8452, 1728.9532, 1738.0576, 1776.9531, 1792.9390, 1802.9620, 1818.9616, 1821.9696, 1823.9891, 1841.9405, 1861.9851, 2113.0764, 2129.0720, 2145.0632, 2146.8377, 2149.1282, 2164.8462, 2184.0111,

2191.8581, 2210.8694, 2236.8811, 2252.8802, 2338.1486, 2462.2482, 2558.1498, 2678.3299, 2804.1100, 2818.1227, 2995.7197, 3010.5509, 3026.5407, 3036.5610, 3052.5555, 3234.3318

20. A0A6G5ZW48_9SAUR **Mass:** 86121 **Score:** 17 **Expect:** 26 **Matches:** 8

Atriopeptidase OS=Vipera anatolica senliki OX=2604287 PE=2 SV=1

Observed	Mr(expt)	Mr(calc)	ppm	Start	End	Miss	Ions	Peptide
922.4699	921.4626	921.4417	22.8	386 -	392	1	---	K.DTRNNFR.K
1108.4728	1107.4655	1107.5342	-62.08	59 -	68	1	---	K.TSDCIKSAAR.I
1281.6541	1280.6468	1280.6513	-3.55	184 -	194	0	---	K.SIAELNFQFGR.K
1301.6959	1300.6886	1300.6155	56.2	372 -	382	0	---	R.YIMDMVSSLSR.E
1505.7880	1504.7807	1504.7310	33.1	654 -	665	1	---	R.QAYRAYESYVQK.N
1685.8462	1684.8389	1684.8321	4.05	359 -	371	1	---	K.YSAREIQNYISWR.Y
1821.9696	1820.9624	1820.8284	73.6	4 -	19	0	---	K.SESQMDITEMNSPKPK.R
2184.0111	2183.0038	2182.9908	5.96	1 -	19	1	---	-.MAKSESQMDITEMNSPKPK.R + 2 Oxidation (M)

No match to: 843.4335, 880.4569, 888.4500, 895.3970, 896.4510, 906.4704, 908.5083, 914.4639, 1009.4438, 1049.4386, 1066.4633, 1077.4646, 1169.5911, 1170.0892, 1189.6611, 1194.5557, 1205.5920, 1212.6192, 1218.6543, 1231.6425, 1233.6705, 1237.6639, 1247.6491, 1249.6639, 1255.6491, 1259.6864, 1265.6592, 1271.6156, 1275.6818, 1291.6754, 1304.5938, 1307.6705, 1326.6012, 1334.5881, 1340.7389, 1429.7302, 1433.8111, 1462.7902, 1477.8012, 1481.7954, 1490.7698, 1493.7944, 1498.6923, 1506.2822, 1507.0891, 1509.7892, 1515.7405, 1521.8025, 1525.7827, 1534.8120, 1547.8074, 1563.8131, 1599.8532, 1606.8106, 1611.7753, 1711.8596, 1723.7778, 1727.8452, 1728.9532, 1738.0576, 1776.9531, 1792.9390, 1802.9620, 1818.9616, 1823.9891, 1841.9405, 1861.9851, 2113.0764, 2129.0720, 2145.0632, 2146.8377, 2149.1282, 2164.8462, 2191.8581, 2210.8694, 2236.8811, 2252.8802, 2338.1486, 2462.2482, 2558.1498, 2609.3629, 2678.3299, 2804.1100, 2818.1227, 2995.7197, 3010.5509, 3026.5407, 3036.5610, 3052.5555, 3234.3318

Search Parameters

Type of search : MS/MS Ion Search
Enzyme : Trypsin
Fixed modifications : Carbamidomethyl (C)
Variable modifications : Oxidation (M)
Mass values : Monoisotopic
Protein Mass : Unrestricted
Peptide Mass Tolerance : ± 100 ppm
Fragment Mass Tolerance: ± 0.7 Da
Max Missed Cleavages : 1
Instrument type : MALDI-TOF-TOF
Query1 (843.4335,1+) : <no title>
Query2 (880.4569,1+) : <no title>
Query3 (888.4500,1+) : <no title>
Query4 (895.3970,1+) : <no title>
Query5 (896.4510,1+) : <no title>
Query6 (906.4704,1+) : <no title>
Query7 (908.5083,1+) : <no title>
Query8 (914.4639,1+) : <no title>
Query9 (922.4699,1+) : <no title>
Query10 (1009.4438,1+) : <no title>
Query11 (1049.4386,1+) : <no title>
Query12 (1066.4633,1+) : <no title>
Query13 (1077.4646,1+) : <no title>
Query14 (1108.4728,1+) : <no title>
Query15 (1169.5911,1+) : <no title>
Query16 (1170.0892,1+) : <no title>
Query17 (1189.6611,1+) : <no title>
Query18 (1194.5557,1+) : <no title>
Query19 (1205.5920,1+) : <no title>
Query20 (1212.6192,1+) : <no title>
Query21 (1218.6543,1+) : <no title>
Query22 (1231.6425,1+) : <no title>
Query23 (1233.6705,1+) : \\PROTEOMICS1\D\DATA\2105\210505_TLIEPOL_1_68252-68266\68252_68266_LPARDO\0_H9\1\1233.6705.LIFT\1SREF\PDATA\1\1R
Query24 (1237.6639,1+) : <no title>
Query25 (1247.6491,1+) : <no title>
Query26 (1249.6639,1+) : <no title>
Query27 (1255.6491,1+) : <no title>
Query28 (1259.6864,1+) : <no title>
Query29 (1265.6592,1+) : <no title>
Query30 (1271.6156,1+) : <no title>
Query31 (1275.6818,1+) : <no title>
Query32 (1281.6541,1+) : <no title>
Query33 (1291.6754,1+) : <no title>
Query34 (1301.6959,1+) : <no title>
Query35 (1304.5938,1+) : <no title>
Query36 (1307.6705,1+) : <no title>
Query37 (1326.6012,1+) : <no title>
Query38 (1334.5881,1+) : <no title>
Query39 (1340.7389,1+) : <no title>
Query40 (1429.7302,1+) : <no title>
Query41 (1433.8111,1+) : <no title>
Query42 (1462.7902,1+) : <no title>
Query43 (1477.8012,1+) : \\PROTEOMICS1\D\DATA\2105\210505_TLIEPOL_1_68252-68266\68252_68266_LPARDO\0_H9\1\1477.8012.LIFT\1SREF\PDATA\1\1R
Query44 (1481.7954,1+) : <no title>
Query45 (1490.7698,1+) : <no title>
Query46 (1493.7944,1+) : <no title>
Query47 (1498.6923,1+) : <no title>
Query48 (1505.7880,1+) : <no title>
Query49 (1506.2822,1+) : <no title>
Query50 (1507.0891,1+) : <no title>
Query51 (1509.7892,1+) : <no title>
Query52 (1515.7405,1+) : <no title>
Query53 (1521.8025,1+) : \\PROTEOMICS1\D\DATA\2105\210505_TLIEPOL_1_68252-68266\68252_68266_LPARDO\0_H9\1\1521.8025.LIFT\1SREF\PDATA\1\1R
Query54 (1525.7827,1+) : <no title>
Query55 (1534.8120,1+) : <no title>
Query56 (1547.8074,1+) : <no title>
Query57 (1563.8131,1+) : <no title>
Query58 (1599.8532,1+) : <no title>
Query59 (1606.8106,1+) : <no title>
Query60 (1611.7753,1+) : <no title>
Query61 (1685.8462,1+) : \\PROTEOMICS1\D\DATA\2105\210505_TLIEPOL_1_68252-68266\68252_68266_LPARDO\0_H9\1\1685.8462.LIFT\1SREF\PDATA\1\1R
Query62 (1711.8596,1+) : <no title>
Query63 (1723.7778,1+) : <no title>
Query64 (1727.8452,1+) : <no title>
Query65 (1728.9532,1+) : <no title>
Query66 (1738.0576,1+) : <no title>
Query67 (1776.9531,1+) : \\PROTEOMICS1\D\DATA\2105\210505_TLIEPOL_1_68252-68266\68252_68266_LPARDO\0_H9\1\1776.9531.LIFT\1SREF\PDATA\1\1R

```
Query68 (1792.9390,1+) : <no title>
Query69 (1802.9620,1+) : <no title>
Query70 (1818.9616,1+) : <no title>
Query71 (1821.9696,1+) : <no title>
Query72 (1823.9891,1+) : \\PROTEOMICS1\D\DATA\2105\210505_TLIEPOL_1_68252-68266\68252_68266_LPARDO\0_H9\1\1823.9891.LIFT\1SREF\PDATA\1\1R
Query73 (1841.9405,1+) : <no title>
Query74 (1861.9851,1+) : <no title>
Query75 (2113.0764,1+) : \\PROTEOMICS1\D\DATA\2105\210505_TLIEPOL_1_68252-68266\68252_68266_LPARDO\0_H9\1\2113.0764.LIFT\1SREF\PDATA\1\1R
Query76 (2129.0720,1+) : <no title>
Query77 (2145.0632,1+) : <no title>
Query78 (2146.8377,1+) : <no title>
Query79 (2149.1282,1+) : \\PROTEOMICS1\D\DATA\2105\210505_TLIEPOL_1_68252-68266\68252_68266_LPARDO\0_H9\1\2149.1282.LIFT\1SREF\PDATA\1\1R
Query80 (2164.8462,1+) : <no title>
Query81 (2184.0111,1+) : <no title>
Query82 (2191.8581,1+) : <no title>
Query83 (2210.8694,1+) : <no title>
Query84 (2236.8811,1+) : <no title>
Query85 (2252.8802,1+) : <no title>
Query86 (2338.1486,1+) : <no title>
Query87 (2462.2482,1+) : <no title>
Query88 (2558.1498,1+) : <no title>
Query89 (2609.3629,1+) : <no title>
Query90 (2678.3299,1+) : <no title>
Query91 (2804.1100,1+) : <no title>
Query92 (2818.1227,1+) : <no title>
Query93 (2995.7197,1+) : <no title>
Query94 (3010.5509,1+) : <no title>
Query95 (3026.5407,1+) : <no title>
Query96 (3036.5610,1+) : <no title>
Query97 (3052.5555,1+) : <no title>
Query98 (3234.3318,1+) : <no title>
```

Mascot: http://www.matrixscience.com/

MATRIX SCIENCE Mascot Search Results Fr6-2

User :
Email :
Search title : vipera_All_8IDplus4 file:\\Proteomics1\d\data\2105\210505_tliepol_1_68252-68266\68252_68266_lpardo\0_G10\1\1SRef\pdata\1\peaklist.xml
Database : vipera (1414 sequences; 347452 residues)
Timestamp : 5 May 2021 at 09:35:49 GMT
Top Score : 61 for VSP2_MACLB, Venom serine proteinase-like protein 2 OS=Macrovipera lebetina OX=8709 PE=2 SV=1

Mascot Score Histogram

Protein score is -10*Log(P), where P is the probability that the observed match is a random event.
Protein scores greater than 44 are significant (p<0.05).

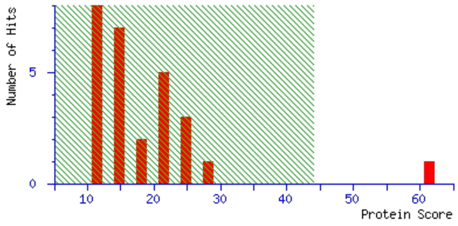

Concise Protein Summary Report

Format As | Concise Protein Summary | Help
Significance threshold p< 0.05 Max. number of hits 10

Re-Search All | Search Unmatched

1. VSP2_MACLB **Mass:** 29559 **Score:** 61 **Expect:** 0.001 **Matches:** 10
Venom serine proteinase-like protein 2 OS=Macrovipera lebetina OX=8709 PE=2 SV=1
A0A6G5ZVX7_9SAUR **Mass:** 29593 **Score:** 39 **Expect:** 0.18 **Matches:** 7
Serine protease 7 OS=Vipera anatolica senliki OX=2604287 PE=2 SV=1
A0A6G5ZUR6_9SAUR **Mass:** 29579 **Score:** 39 **Expect:** 0.18 **Matches:** 7
Serine protease 6 OS=Vipera anatolica senliki OX=2604287 PE=2 SV=1
VSPH1_VIPAA **Mass:** 29593 **Score:** 39 **Expect:** 0.18 **Matches:** 7
Vaa serine proteinase homolog 1 OS=Vipera ammodytes ammodytes OX=8705 PE=1 SV=1
A0A6G5ZUW7_9SAUR **Mass:** 22103 **Score:** 18 **Expect:** 25 **Matches:** 5
Serine protease 10 (Fragment) OS=Vipera anatolica senliki OX=2604287 PE=2 SV=1
A0A6B7FMQ3_VIPAA **Mass:** 28909 **Score:** 13 **Expect:** 69 **Matches:** 3
Snake venom serine protease OS=Vipera ammodytes ammodytes OX=8705 PE=2 SV=1

---

2. VASP1_VIPAA **Mass:** 22635 **Score:** 28 **Expect:** 2.3 **Matches:** 5
Snake venom serine protease VaSP1 (Fragments) OS=Vipera ammodytes ammodytes OX=8705 PE=1 SV=1

---

3. A0A6G5ZW27_9SAUR **Mass:** 38471 **Score:** 26 **Expect:** 3.7 **Matches:** 5
Venom factor 6 (Fragment) OS=Vipera anatolica senliki OX=2604287 PE=2 SV=1
A0A6G5ZUM0_9SAUR **Mass:** 9192 **Score:** 19 **Expect:** 16 **Matches:** 2
Venom factor 3 (Fragment) OS=Vipera anatolica senliki OX=2604287 PE=2 SV=1

---

4. A0A0F6N0C3_9PERO **Mass:** 80354 **Score:** 25 **Expect:** 4.3 **Matches:** 6
Echiitoxin a-subunit OS=Echiichthys vipera OX=94984 PE=2 SV=1

---

5. A0A6G5ZW48_9SAUR **Mass:** 86121 **Score:** 23 **Expect:** 6.5 **Matches:** 9
Atriopeptidase OS=Vipera anatolica senliki OX=2604287 PE=2 SV=1
A0A6G5ZUN8_9SAUR **Mass:** 61639 **Score:** 23 **Expect:** 7.4 **Matches:** 8
Atriopeptidase (Fragment) OS=Vipera anatolica senliki OX=2604287 PE=2 SV=1

---

6. Q6EIM3_VIPAP **Mass:** 34394 **Score:** 23 **Expect:** 7.4 **Matches:** 4
NADH-ubiquinone oxidoreductase chain 2 (Fragment) OS=Vipera aspis aspis OX=194601 GN=ND2 PE=3 SV=1

---

7. Q6A373_VIPAA **Mass:** 16390 **Score:** 23 **Expect:** 7.6 **Matches:** 4
Ammodytin L(1'') variant OS=Vipera ammodytes ammodytes OX=8705 PE=2 SV=1

---

8. A0A6G5ZUB2_9SAUR **Mass:** 81727 **Score:** 22 **Expect:** 8.3 **Matches:** 7
Disintegrin and metalloproteinase domain-containing protein 9 (Fragment) OS=Vipera anatolica senliki OX=2604287 PE=2 SV=1

---

9. A0A0H3Y7H2_9PERO **Mass:** 22231 **Score:** 20 **Expect:** 13 **Matches:** 3
Uncharacterized protein (Fragment) OS=Echiichthys vipera OX=94984 GN=ptr PE=4 SV=1

---

10. A0A6G5ZW30_9SAUR **Mass:** 186570 **Score:** 20 **Expect:** 14 **Matches:** 11
Venom factor 1 OS=Vipera anatolica senliki OX=2604287 PE=2 SV=1
A0A6G5ZUM5_9SAUR **Mass:** 57543 **Score:** 12 **Expect:** 98 **Matches:** 4
Venom factor 5 (Fragment) OS=Vipera anatolica senliki OX=2604287 PE=2 SV=1

Search Parameters

Type of search : Peptide Mass Fingerprint
Enzyme : Trypsin
Fixed modifications : Carbamidomethyl (C)
Variable modifications : Oxidation (M)
Mass values : Monoisotopic
Protein Mass : Unrestricted
Peptide Mass Tolerance : ± 100 ppm
Peptide Charge State : 1+
Max Missed Cleavages : 1
Number of queries : 100
Selected for scoring : 60

Mascot: http://www.matrixscience.com/

Mascot Search Results

```
User           :
Email          :
Search title   : vipera_all_Lift file:\\Proteomics1\d\data\2105\210505_tliepol_1_68252-68266\68252_68266_lpardo\0_G10\1\0_G10.xml
Database       : vipera  (1414 sequences; 347452 residues)
Timestamp      : 5 May 2021 at 09:57:15 GMT
Protein hits   : VASP1_VIPAA       Snake venom serine protease VaSP1 (Fragments) OS=Vipera ammodytes ammodytes OX=8705 PE=1 SV=1
                 VSP2_MACLB        Venom serine proteinase-like protein 2 OS=Macrovipera lebetina OX=8709 PE=2 SV=1
                 A0A6G5ZVV8_9SAUR  Dipeptidylpeptidase 1 (Fragment) OS=Vipera anatolica senliki OX=2604287 PE=2 SV=1
                 A0A170U0F6_VIPBN  Prolactin receptor (Fragment) OS=Vipera berus nikolskii OX=1808362 GN=PRLR PE=4 SV=1
```

Mascot Score Histogram

Ions score is -10*Log(P), where P is the probability that the observed match is a random event.
Individual ions scores > 12 indicate identity or extensive homology (p<0.05).
Protein scores are derived from ions scores as a non-probabilistic basis for ranking protein hits.

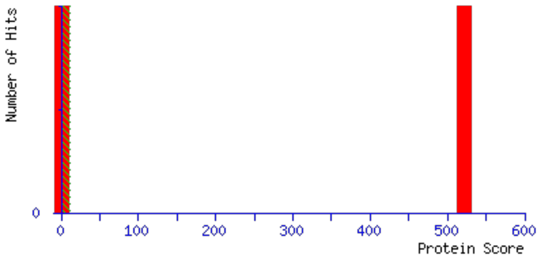

Peptide Summary Report

Format As: Peptide Summary — Help

Significance threshold p< 0.05 — Max. number of hits 10

Standard scoring ○ MudPIT scoring ◉ Ions score or expect cut-off 0 — Show sub-sets 0

Show pop-ups ◉ Suppress pop-ups ○ Sort unassigned Decreasing Score — Require bold red ☐

Overview Table

Click on column header to jump to entry in results list.
Move mouse over any indicator to highlight identical peptides.
Click on an indicator to see details of individual match.
Use check boxes to select sub-set of queries for new search.

Mouse over: -Query- -Accession- -Sequence-

Hit:	1	2	3	4
☑ 866.5712 (1+)				
☑ 888.4510 (1+)		●		
☑ 1077.4785 (1+)				
☑ 1172.6386 (1+)				
☑ 1186.6312 (1+)				
☑ 1189.6689 (1+)	●	●	●	
☑ 1205.6624 (1+)	●	●		
☑ 1208.6590 (1+)				
☑ 1212.6257 (1+)		●		
☑ 1215.6815 (1+)				
☑ 1221.6559 (1+)				
☑ 1230.6987 (1+)				
☑ 1231.6710 (1+)				
☑ 1232.1392 (1+)				
☑ 1234.6802 (1+)				
☑ 1237.6524 (1+)				
☑ 1246.6827 (1+)				
☑ 1304.6060 (1+)				
☑ 1320.5943 (1+)				
☑ 1330.6201 (1+)				
☑ 1346.6114 (1+)				
☑ 1372.7216 (1+)				
☑ 1429.7399 (1+)				
☑ 1450.7394 (1+)				
☑ 1592.8032 (1+)	●	●		

☑ 1632.6900 (1+)				
☑ 1642.9239 (1+)				
☑ 1649.7186 (1+)				
☑ 1665.7056 (1+)				
☑ 1731.9567 (1+)				
☑ 1802.9521 (1+)				
☑ 1852.0286 (1+)				
☑ 1997.0124 (1+)				
☑ 2022.0758 (1+)				
☑ 2038.0531 (1+)				
☑ 2053.0494 (1+)				
☑ 2056.0667 (1+)				
☑ 2066.0564 (1+)				
☑ 2069.0742 (1+)				
☑ 2075.0641 (1+)				
☑ 2080.0660 (1+)				
☑ 2094.0792 (1+)				
☑ 2106.0776 (1+)				
☑ 2109.0960 (1+)				
☑ 2111.0719 (1+)				
☑ 2113.0883 (1+)	●	●		
☑ 2118.0892 (1+)				
☑ 2124.0832 (1+)				
☑ 2127.0923 (1+)				
☑ 2130.0855 (1+)				
☑ 2134.0649 (1+)				
☑ 2137.0790 (1+)				
☑ 2139.1052 (1+)				
☑ 2145.0792 (1+)				
☑ 2155.1018 (1+)				●
☑ 2159.0676 (1+)				
☑ 2161.0764 (1+)				
☑ 2171.0968 (1+)				
☑ 2187.0862 (1+)				
☑ 2257.1391 (1+)				
☑ 2265.0817 (1+)				
☑ 2285.1075 (1+)				
☑ 2300.1160 (1+)				
☑ 2371.2527 (1+)				
☑ 2386.2189 (1+)				
☑ 2405.2424 (1+)				
☑ 2418.2571 (1+)				
☑ 2442.1046 (1+)				
☑ 2443.2546 (1+)				
☑ 2447.4393 (1+)				
☑ 2455.2529 (1+)				
☑ 2458.2475 (1+)				
☑ 2462.2618 (1+)	●			
☑ 2473.2683 (1+)				
☑ 2476.2783 (1+)				
☑ 2485.1932 (1+)				
☑ 2488.2746 (1+)				
☑ 2494.2292 (1+)				
☑ 2501.1388 (1+)				
☑ 2504.2755 (1+)				
☑ 2510.1608 (1+)				
☑ 2513.1576 (1+)				
☑ 2518.8602 (1+)				
☑ 2519.2909 (1+)				
☑ 2530.2932 (1+)				
☑ 2535.2088 (1+)				
☑ 2539.1622 (1+)				
☑ 2551.1621 (1+)				
☑ 2556.0916 (1+)				
☑ 2558.1673 (1+)		●		
☑ 2569.1725 (1+)				
☑ 2572.1856 (1+)				
☑ 2574.1490 (1+)		●		

☑ 2575.3485 (1+)				
☑ 2584.1815 (1+)				
☑ 2590.1477 (1+)				
☑ 2600.1769 (1+)				
☑ 2968.4276 (1+)				
☑ 3096.5257 (1+)				
☑ 3540.7719 (1+)				

Select All | Select None | Search Selected | ☐ **Error tolerant** | Archive Report

1. VASP1_VIPAA **Mass:** 22635 **Score:** 522 **Matches:** 5(5) **Sequences:** 4(4) **emPAI:** 2.42
Snake venom serine protease VaSP1 (Fragments) OS=Vipera ammodytes ammodytes OX=8705 PE=1 SV=1
☐ Check to include this hit in error tolerant search or archive report

Query	Observed	Mr(expt)	Mr(calc)	ppm	Miss	Score	Expect	Rank	Unique	Peptide
6	1189.6689	1188.6617	1188.6325	24.5	1	81	1e-008	1		R.WDKDIMLIR.L
7	1205.6624	1204.6551	1204.6274	23.0	1	(33)	0.00071	1		R.WDKDIMLIR.L + Oxidation (M)
25	1592.8032	1591.7960	1591.7698	16.4	0	110	9.1e-012	1		R.TLCAGILQGGIDSCK.G
46	2113.0883	2112.0810	2112.0462	16.5	0	174	4.1e-018	1		R.FYCAGTLINQEWVLTAAR.C
73	2462.2618	2461.2545	2461.2172	15.2	0	176	2.6e-018	1	U	-.VIGGDECNINEHPFLVALHTAR.X

2. VSP2_MACLB **Mass:** 29559 **Score:** 508 **Matches:** 8(6) **Sequences:** 6(5) **emPAI:** 2.11
Venom serine proteinase-like protein 2 OS=Macrovipera lebetina OX=8709 PE=2 SV=1
☐ Check to include this hit in error tolerant search or archive report

Query	Observed	Mr(expt)	Mr(calc)	ppm	Miss	Score	Expect	Rank	Unique	Peptide
2	888.4510	887.4438	887.4211	25.5	0	7	0.2	1	U	K.FFCLSSK.T
6	1189.6689	1188.6617	1188.6325	24.5	1	81	1e-008	1		R.WDKDIMLIR.L
7	1205.6624	1204.6551	1204.6274	23.0	1	(33)	0.00071	1		R.WDKDIMLIR.L + Oxidation (M)
9	1212.6257	1211.6184	1211.5894	23.9	0	43	4.8e-005	1	U	K.NVPNEDQQIR.V
25	1592.8032	1591.7960	1591.7698	16.4	0	110	9.1e-012	1		R.TLCAGILQGGIDSCK.V
46	2113.0883	2112.0810	2112.0462	16.5	0	174	4.1e-018	1		R.FYCAGTLINQEWVLTAAR.C
90	2558.1673	2557.1600	2557.1189	16.1	0	132	5.9e-014	1	U	K.VTYPDVPHCANINMFDYSVCR.K
93	2574.1490	2573.1417	2573.1138	10.9	0	(11)	0.077	1	U	K.VTYPDVPHCANINMFDYSVCR.K + Oxidation (M)

3. A0A6G5ZVV8_9SAUR **Mass:** 89505 **Score:** 0 **Matches:** 1(0) **Sequences:** 1(0)
Dipeptidylpeptidase 1 (Fragment) OS=Vipera anatolica senliki OX=2604287 PE=2 SV=1
☐ Check to include this hit in error tolerant search or archive report

Query	Observed	Mr(expt)	Mr(calc)	ppm	Miss	Score	Expect	Rank	Unique	Peptide
6	1189.6689	1188.6617	1188.6615	0.12	1	8	0.2	2	U	K.WVSNTKTVVR.W

Proteins matching the same set of peptides:
A0A6G5ZVW0_9SAUR **Mass:** 91593 **Score:** 0 **Matches:** 1(0) **Sequences:** 1(0)
Dipeptidylpeptidase 2 (Fragment) OS=Vipera anatolica senliki OX=2604287 PE=2 SV=1

4. A0A170U0F6_VIPBN **Mass:** 20679 **Score:** 0 **Matches:** 1(0) **Sequences:** 1(0)
Prolactin receptor (Fragment) OS=Vipera berus nikolskii OX=1808362 GN=PRLR PE=4 SV=1
☐ Check to include this hit in error tolerant search or archive report

Query	Observed	Mr(expt)	Mr(calc)	ppm	Miss	Score	Expect	Rank	Unique	Peptide
55	2155.1018	2154.0945	2154.0627	14.8	1	0	0.99	1	U	K.EVCKLLPAAEVPDSDEVQR.N

Peptide matches not assigned to protein hits: (no details means no match)

	Query	Observed	Mr(expt)	Mr(calc)	ppm	Miss	Score	Expect	Rank	Unique	Peptide
☑	1	*866.5712*	*865.5640*								
☑	3	*1077.4785*	*1076.4712*								
☑	4	*1172.6386*	*1171.6313*								
☑	5	*1186.6312*	*1185.6239*								
☑	8	*1208.6590*	*1207.6517*								
☑	10	*1215.6815*	*1214.6743*								
☑	11	*1221.6559*	*1220.6486*								
☑	12	*1230.6987*	*1229.6914*								
☑	13	*1231.6710*	*1230.6637*								
☑	14	*1232.1392*	*1231.1319*								
☑	15	*1234.6802*	*1233.6729*								
☑	16	*1237.6524*	*1236.6452*								
☑	17	*1246.6827*	*1245.6754*								
☑	18	*1304.6060*	*1303.5987*								
☑	19	*1320.5943*	*1319.5871*								
☑	20	*1330.6201*	*1329.6128*								
☑	21	*1346.6114*	*1345.6041*								
☑	22	*1372.7216*	*1371.7144*								
☑	23	*1429.7399*	*1428.7327*								
☑	24	*1450.7394*	*1449.7321*								
☑	26	*1632.6900*	*1631.6827*								

☑	27	1642.9239	1641.9166
☑	28	1649.7186	1648.7113
☑	29	1665.7056	1664.6983
☑	30	1731.9567	1730.9494
☑	31	1802.9521	1801.9449
☑	32	1852.0286	1851.0213
☑	33	1997.0124	1996.0051
☑	34	2022.0758	2021.0686
☑	35	2038.0531	2037.0459
☑	36	2053.0494	2052.0421
☑	37	2056.0667	2055.0595
☑	38	2066.0564	2065.0492
☑	39	2069.0742	2068.0669
☑	40	2075.0641	2074.0568
☑	41	2080.0660	2079.0587
☑	42	2094.0792	2093.0719
☑	43	2106.0776	2105.0704
☑	44	2109.0960	2108.0887
☑	45	2111.0719	2110.0647
☑	47	2118.0892	2117.0819
☑	48	2124.0832	2123.0759
☑	49	2127.0923	2126.0850
☑	50	2130.0855	2129.0783
☑	51	2134.0649	2133.0576
☑	52	2137.0790	2136.0717
☑	53	2139.1052	2138.0979
☑	54	2145.0792	2144.0719
☑	56	2159.0676	2158.0603
☑	57	2161.0764	2160.0691
☑	58	2171.0968	2170.0895
☑	59	2187.0862	2186.0790
☑	60	2257.1391	2256.1318
☑	61	2265.0817	2264.0745
☑	62	2285.1075	2284.1002
☑	63	2300.1160	2299.1087
☑	64	2371.2527	2370.2454
☑	65	2386.2189	2385.2116
☑	66	2405.2424	2404.2352
☑	67	2418.2571	2417.2498
☑	68	2442.1046	2441.0973
☑	69	2443.2546	2442.2473
☑	70	2447.4393	2446.4320
☑	71	2455.2529	2454.2456
☑	72	2458.2475	2457.2402
☑	74	2473.2683	2472.2610
☑	75	2476.2783	2475.2710
☑	76	2485.1932	2484.1860
☑	77	2488.2746	2487.2673
☑	78	2494.2292	2493.2219
☑	79	2501.1388	2500.1315
☑	80	2504.2755	2503.2683
☑	81	2510.1608	2509.1536
☑	82	2513.1576	2512.1504
☑	83	2518.8602	2517.8529
☑	84	2519.2909	2518.2836
☑	85	2530.2932	2529.2859
☑	86	2535.2088	2534.2015
☑	87	2539.1622	2538.1549
☑	88	2551.1621	2550.1548
☑	89	2556.0916	2555.0843
☑	91	2569.1725	2568.1652
☑	92	2572.1856	2571.1783
☑	94	2575.3485	2574.3412
☑	95	2584.1815	2583.1742
☑	96	2590.1477	2589.1405
☑	97	2600.1769	2599.1697
☑	98	2968.4276	2967.4204
☑	99	3096.5257	3095.5184
☑	100	3540.7719	3539.7646

Search Parameters

```
Type of search         : MS/MS Ion Search
Enzyme                 : Trypsin
Fixed modifications    : Carbamidomethyl (C)
Variable modifications : Oxidation (M)
Mass values            : Monoisotopic
Protein Mass           : Unrestricted
Peptide Mass Tolerance : ± 100 ppm
Fragment Mass Tolerance: ± 0.7 Da
Max Missed Cleavages   : 1
Instrument type        : MALDI-TOF-TOF
```

```
Number of queries      : 100
```

Mascot: http://www.matrixscience.com/

Mascot Search Results

```
User         :
Email        :
Search title : vipera_all_Lift file:\\Proteomics1\d\data\2105\210505_tliepol_1_68252-68266\68252_68266_lpardo\0_G10\1\0_G10.xml
Database     : vipera  (1414 sequences; 347452 residues)
Timestamp    : 5 May 2021 at 09:57:15 GMT
Warning      : A Peptide summary report will usually give a much clearer picture of MS/MS search results.
Top Score    : 592 for VSP2_MACLB, Venom serine proteinase-like protein 2 OS=Macrovipera lebetina OX=8709 PE=2 SV=1
```

Mascot Score Histogram

Protein score is -10*Log(P), where P is the probability that the observed match is a random event.
Protein scores greater than 44 are significant (p<0.05).
Protein scores are derived from ions scores as a non-probabilistic basis for ranking protein hits.

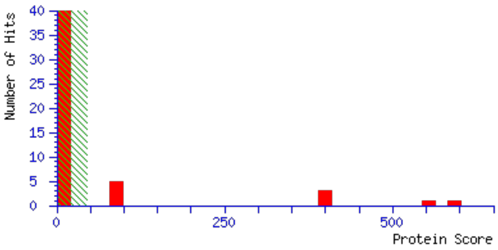

Protein Summary Report

Format As | Protein Summary (deprecated) | Help

Significance threshold p< 0.05 Max. number of hits 20

Overview Table

Click on column header to jump to entry in results list.
Move mouse over any indicator to highlight identical peptides.
Click on an indicator to see details of individual match.
Use check boxes to select sub-set of queries for new search.

Mouse over: -Query- -Accession- -Sequence-

Hit:	1	2	3	4	5	6	7	8	9	10	11	12	13	14	15	16	17	18	19	20
☑ 866.5712 (1+)																				
☑ 888.4510 (1+)	●		●	●	●	●	●													
☑ 1077.4785 (1+)																				
☑ 1172.6386 (1+)																				
☑ 1186.6312 (1+)																				
☑ 1189.6689 (1+)	●	●	●	●	●	●	●	●	●	●										
☑ 1205.6624 (1+)	●	●	●	●	●	●	●	●	●	●										
☑ 1208.6590 (1+)																				
☑ 1212.6257 (1+)	●																			
☑ 1215.6815 (1+)																				
☑ 1221.6559 (1+)																				
☑ 1230.6987 (1+)																				
☑ 1231.6710 (1+)																				
☑ 1232.1392 (1+)																				
☑ 1234.6802 (1+)																				
☑ 1237.6524 (1+)																				
☑ 1246.6827 (1+)																				
☑ 1304.6060 (1+)																				
☑ 1320.5943 (1+)																				
☑ 1330.6201 (1+)																				
☑ 1346.6114 (1+)																				
☑ 1372.7216 (1+)																				
☑ 1429.7399 (1+)																				
☑ 1450.7394 (1+)																				
☑ 1592.8032 (1+)	●	●	●	●	●															
☑ 1632.6900 (1+)																				
☑ 1642.9239 (1+)																				
☑ 1649.7186 (1+)																				
☑ 1665.7056 (1+)																				
☑ 1731.9567 (1+)																				
☑ 1802.9521 (1+)																				
☑ 1852.0286 (1+)																				
☑ 1997.0124 (1+)																				
☑ 2022.0758 (1+)																				

☑ 2038.0531 (1+)																				
☑ 2053.0494 (1+)																				
☑ 2056.0667 (1+)																				
☑ 2066.0564 (1+)																				
☑ 2069.0742 (1+)																				
☑ 2075.0641 (1+)																				
☑ 2080.0660 (1+)																				
☑ 2094.0792 (1+)																				
☑ 2106.0776 (1+)																				
☑ 2109.0960 (1+)																				
☑ 2111.0719 (1+)																				
☑ 2113.0883 (1+)	●	●	●	●	●															
☑ 2118.0892 (1+)																				
☑ 2124.0832 (1+)																				
☑ 2127.0923 (1+)																				
☑ 2130.0855 (1+)																				
☑ 2134.0649 (1+)																				
☑ 2137.0790 (1+)																				
☑ 2139.1052 (1+)																				
☑ 2145.0792 (1+)																				
☑ 2155.1018 (1+)																				
☑ 2159.0676 (1+)																				
☑ 2161.0764 (1+)																				
☑ 2171.0968 (1+)																				
☑ 2187.0862 (1+)																				
☑ 2257.1391 (1+)																				
☑ 2265.0817 (1+)																				
☑ 2285.1075 (1+)																				
☑ 2300.1160 (1+)																				
☑ 2371.2527 (1+)																				
☑ 2386.2189 (1+)																				
☑ 2405.2424 (1+)																				
☑ 2418.2571 (1+)																				
☑ 2442.1046 (1+)																				
☑ 2443.2546 (1+)																				
☑ 2447.4393 (1+)																				
☑ 2455.2529 (1+)																				
☑ 2458.2475 (1+)																				
☑ 2462.2618 (1+)		●																		
☑ 2473.2683 (1+)																				
☑ 2476.2783 (1+)																				
☑ 2485.1932 (1+)																				
☑ 2488.2746 (1+)																				
☑ 2494.2292 (1+)																				
☑ 2501.1388 (1+)																				
☑ 2504.2755 (1+)																				
☑ 2510.1608 (1+)																				
☑ 2513.1576 (1+)																				
☑ 2518.8602 (1+)																				
☑ 2519.2909 (1+)																				
☑ 2530.2932 (1+)																				
☑ 2535.2088 (1+)																				
☑ 2539.1622 (1+)																				
☑ 2551.1621 (1+)																				
☑ 2556.0916 (1+)																				
☑ 2558.1673 (1+)	●																			
☑ 2569.1725 (1+)																				
☑ 2572.1856 (1+)																				
☑ 2574.1490 (1+)	●																			
☑ 2575.3485 (1+)																				
☑ 2584.1815 (1+)																				
☑ 2590.1477 (1+)																				
☑ 2600.1769 (1+)																				
☑ 2968.4276 (1+)																				
☑ 3096.5257 (1+)																				
☑ 3540.7719 (1+)																				

Select All | Select None | Search Selected

Index

	Accession	Mass	Score	Description
1.	VSP2_MACLB	29559	592	Venom serine proteinase-like protein 2 OS=Macrovipera lebetina OX=8709 PE=2 SV=1
2.	VASP1_VIPAA	22635	561	Snake venom serine protease VaSP1 (Fragments) OS=Vipera ammodytes ammodytes OX=8705 PE=1 SV=1

3.	**A0A6G5ZVX7_9SAUR**	29593	399	Serine protease 7 OS=Vipera anatolica senliki OX=2604287 PE=2 SV=1
4.	**A0A6G5ZUR6_9SAUR**	29579	399	Serine protease 6 OS=Vipera anatolica senliki OX=2604287 PE=2 SV=1
5.	**VSPH1_VIPAA**	29593	399	Vaa serine proteinase homolog 1 OS=Vipera ammodytes ammodytes OX=8705 PE=1 SV=1
6.	**A0A6G5ZUW7_9SAUR**	22103	105	Serine protease 10 (Fragment) OS=Vipera anatolica senliki OX=2604287 PE=2 SV=1
7.	**A0A6B7FMQ3_VIPAA**	28909	90	Snake venom serine protease OS=Vipera ammodytes ammodytes OX=8705 PE=2 SV=1
8.	**D8MIA1_BITRH**	29504	88	Rhinocerase 3 protein (Fragment) OS=Bitis rhinoceros OX=715877 GN=rhinocerase 3 PE=3 SV=1
9.	**D8MIA0_BITRH**	29517	88	Rhinocerase 2 protein (Fragment) OS=Bitis rhinoceros OX=715877 GN=rhinocerase 2 PE=3 SV=1
10.	**VSPY_MACLB**	28944	78	Chymotrypsin-like protease VLCTLP OS=Macrovipera lebetina OX=8709 PE=1 SV=1
11.	**Q6A373_VIPAA**	16390	26	Ammodytin L(1'') variant OS=Vipera ammodytes ammodytes OX=8705 PE=2 SV=1
12.	**A0A6G5ZUN8_9SAUR**	61639	24	Atriopeptidase (Fragment) OS=Vipera anatolica senliki OX=2604287 PE=2 SV=1
13.	**A0A6G5ZW48_9SAUR**	86121	22	Atriopeptidase OS=Vipera anatolica senliki OX=2604287 PE=2 SV=1
14.	**A0A6G5ZW27_9SAUR**	38471	19	Venom factor 6 (Fragment) OS=Vipera anatolica senliki OX=2604287 PE=2 SV=1
15.	**PA2HL_VIPAA**	16481	19	Basic phospholipase A2 homolog ammodytin L OS=Vipera ammodytes ammodytes OX=8705 PE=1 SV=1
16.	**Q6A371_VIPAM**	16461	19	Ammodytin L(3) isoform OS=Vipera ammodytes montandoni OX=235554 PE=2 SV=1
17.	**Q6A382_VIPAA**	16431	19	Ammodytin L(1'') variant OS=Vipera ammodytes ammodytes OX=8705 PE=2 SV=1
18.	**Q6A367_VIPAM**	16403	19	Ammodytin L(3) variant OS=Vipera ammodytes montandoni OX=235554 PE=2 SV=1
19.	**Q6A369_VIPAM**	16449	19	Ammodytin L(3) variant OS=Vipera ammodytes montandoni OX=235554 PE=2 SV=1
20.	**Q6A368_VIPAM**	16489	19	Ammodytin L(3) variant OS=Vipera ammodytes montandoni OX=235554 PE=2 SV=1

Results List

1. VSP2_MACLB **Mass:** 29559 **Score:** 592 **Expect:** 8.9e-057 **Matches:** 10

Venom serine proteinase-like protein 2 OS=Macrovipera lebetina OX=8709 PE=2 SV=1

Observed	Mr(expt)	Mr(calc)	ppm	Start	End	Miss	Ions	Peptide
866.5712	865.5640	865.5385	29.4	75 -	82	0	---	R.IILGVHSK.N
888.4510	887.4438	887.4211	25.5	98 -	104	0	7	K.FFCLSSK.T
1189.6689	1188.6617	1188.6325	24.5	109 -	117	1	81	R.WDKDIMLIR.L
1205.6624	1204.6551	1204.6274	23.0	109 -	117	1	(33)	R.WDKDIMLIR.L + Oxidation (M)
1208.6590	1207.6517	1207.6271	20.4	146 -	156	0	---	R.IMGWGTITTTK.V
1212.6257	1211.6184	1211.5894	23.9	83 -	92	0	43	K.NVPNEDQQIR.V
1592.8032	1591.7960	1591.7698	16.4	189 -	203	0	110	R.TLCAGILQGGIDSCK.V
2113.0883	2112.0810	2112.0462	16.5	50 -	67	0	174	R.FYCAGTLINQEWVLTAAR.C
2558.1673	2557.1600	2557.1189	16.1	157 -	177	0	132	K.VTYPDVPHCANINMFDYSVCR.K
2574.1490	2573.1417	2573.1138	10.9	157 -	177	0	(11)	K.VTYPDVPHCANINMFDYSVCR.K + Oxidation (M)

No match to: 1077.4785, 1172.6386, 1186.6312, 1215.6815, 1221.6559, 1230.6987, 1231.6710, 1232.1392, 1234.6802, 1237.6524, 1246.6827, 1304.6060, 1320.5943, 1330.6201, 1346.6114, 1372.7216, 1429.7399, 1450.7394, 1632.6900, 1642.9239, 1649.7186, 1665.7056, 1731.9567, 1802.9521, 1852.0286, 1997.0124, 2022.0758, 2038.0531, 2053.0494, 2056.0667, 2066.0564, 2069.0742, 2075.0641, 2080.0660, 2094.0792, 2106.0776, 2109.0960, 2111.0719, 2118.0892, 2124.0832, 2127.0923, 2130.0855, 2134.0649, 2137.0790, 2139.1052, 2145.0792, 2155.1018, 2159.0676, 2161.0764, 2171.0968, 2187.0862, 2257.1391, 2265.0817, 2285.1075, 2300.1160, 2371.2527, 2386.2189, 2405.2424, 2418.2571, 2442.1046, 2443.2546, 2447.4393, 2455.2529, 2458.2475, 2462.2618, 2473.2683, 2476.2783, 2485.1932, 2488.2746, 2494.2292, 2501.1388, 2504.2755, 2510.1608, 2513.1576, 2518.8602, 2519.2909, 2530.2932, 2535.2088, 2539.1622, 2551.1621, 2556.0916, 2569.1725, 2572.1856, 2575.3485, 2584.1815, 2590.1477, 2600.1769, 2968.4276, 3096.5257, 3540.7719

2. VASP1_VIPAA **Mass:** 22635 **Score:** 561 **Expect:** 1.1e-053 **Matches:** 5

Snake venom serine protease VaSP1 (Fragments) OS=Vipera ammodytes ammodytes OX=8705 PE=1 SV=1

Observed	Mr(expt)	Mr(calc)	ppm	Start	End	Miss	Ions	Peptide
1189.6689	1188.6617	1188.6325	24.5	85 -	93	1	81	R.WDKDIMLIR.L
1205.6624	1204.6551	1204.6274	23.0	85 -	93	1	(33)	R.WDKDIMLIR.L + Oxidation (M)
1592.8032	1591.7960	1591.7698	16.4	165 -	179	0	110	R.TLCAGILQGGIDSCK.G
2113.0883	2112.0810	2112.0462	16.5	26 -	43	0	174	R.FYCAGTLINQEWVLTAAR.C
2462.2618	2461.2545	2461.2172	15.2	1 -	22	0	176	-.VIGGDECNINEHPFLVALHTAR.X

No match to: 866.5712, 888.4510, 1077.4785, 1172.6386, 1186.6312, 1208.6590, 1212.6257, 1215.6815, 1221.6559, 1230.6987, 1231.6710, 1232.1392, 1234.6802, 1237.6524, 1246.6827, 1304.6060, 1320.5943, 1330.6201, 1346.6114, 1372.7216, 1429.7399, 1450.7394, 1632.6900, 1642.9239, 1649.7186, 1665.7056, 1731.9567, 1802.9521, 1852.0286, 1997.0124, 2022.0758, 2038.0531, 2053.0494, 2056.0667, 2066.0564, 2069.0742, 2075.0641, 2080.0660, 2094.0792, 2106.0776, 2109.0960, 2111.0719, 2118.0892, 2124.0832, 2127.0923, 2130.0855, 2134.0649, 2137.0790, 2139.1052, 2145.0792, 2155.1018, 2159.0676, 2161.0764, 2171.0968, 2187.0862, 2257.1391, 2265.0817, 2285.1075, 2300.1160, 2371.2527, 2386.2189, 2405.2424, 2418.2571, 2442.1046, 2443.2546, 2447.4393, 2455.2529, 2458.2475, 2473.2683, 2476.2783, 2485.1932, 2488.2746, 2494.2292, 2501.1388, 2504.2755, 2510.1608, 2513.1576, 2518.8602, 2519.2909, 2530.2932, 2535.2088, 2539.1622, 2551.1621, 2556.0916, 2558.1673, 2569.1725, 2572.1856, 2574.1490, 2575.3485, 2584.1815, 2590.1477, 2600.1769, 2968.4276, 3096.5257, 3540.7719

3. A0A6G5ZVX7_9SAUR **Mass:** 29593 **Score:** 399 **Expect:** 1.8e-037 **Matches:** 7

Serine protease 7 OS=Vipera anatolica senliki OX=2604287 PE=2 SV=1

Observed	Mr(expt)	Mr(calc)	ppm	Start	End	Miss	Ions	Peptide
866.5712	865.5640	865.5385	29.4	75 -	82	0	---	R.IILGVHSK.N
888.4510	887.4438	887.4211	25.5	98 -	104	0	7	K.FFCLSSK.T
1189.6689	1188.6617	1188.6325	24.5	109 -	117	1	81	R.WDKDIMLIR.L
1205.6624	1204.6551	1204.6274	23.0	109 -	117	1	(33)	R.WDKDIMLIR.L + Oxidation (M)
1208.6590	1207.6517	1207.6271	20.4	146 -	156	0	---	R.IMGWGTITTTK.V
1592.8032	1591.7960	1591.7698	16.4	189 -	203	0	110	R.TLCAGILQGGIDSCK.V
2113.0883	2112.0810	2112.0462	16.5	50 -	67	0	174	R.FYCAGTLINQEWVLTAAR.C

No match to: 1077.4785, 1172.6386, 1186.6312, 1212.6257, 1215.6815, 1221.6559, 1230.6987, 1231.6710, 1232.1392, 1234.6802, 1237.6524, 1246.6827, 1304.6060, 1320.5943, 1330.6201, 1346.6114, 1372.7216, 1429.7399, 1450.7394, 1632.6900, 1642.9239, 1649.7186, 1665.7056, 1731.9567, 1802.9521, 1852.0286, 1997.0124, 2022.0758, 2038.0531, 2053.0494, 2056.0667, 2066.0564, 2069.0742, 2075.0641, 2080.0660, 2094.0792, 2106.0776, 2109.0960, 2111.0719, 2118.0892, 2124.0832, 2127.0923, 2130.0855, 2134.0649, 2137.0790, 2139.1052, 2145.0792, 2155.1018, 2159.0676, 2161.0764, 2171.0968, 2187.0862, 2257.1391, 2265.0817, 2285.1075, 2300.1160, 2371.2527, 2386.2189, 2405.2424, 2418.2571, 2442.1046, 2443.2546, 2447.4393, 2455.2529, 2458.2475, 2462.2618, 2473.2683, 2476.2783, 2485.1932, 2488.2746, 2494.2292, 2501.1388, 2504.2755, 2510.1608, 2513.1576, 2518.8602, 2519.2909, 2530.2932, 2535.2088, 2539.1622, 2551.1621, 2556.0916, 2558.1673, 2569.1725, 2572.1856, 2574.1490, 2575.3485, 2584.1815, 2590.1477, 2600.1769, 2968.4276, 3096.5257, 3540.7719

4. A0A6G5ZUR6_9SAUR **Mass:** 29579 **Score:** 399 **Expect:** 1.8e-037 **Matches:** 7

Serine protease 6 OS=Vipera anatolica senliki OX=2604287 PE=2 SV=1

Observed	Mr(expt)	Mr(calc)	ppm	Start	End	Miss	Ions	Peptide
866.5712	865.5640	865.5385	29.4	75 -	82	0	---	R.IILGVHSK.N
888.4510	887.4438	887.4211	25.5	98 -	104	0	7	K.FFCLSSK.S
1189.6689	1188.6617	1188.6325	24.5	109 -	117	1	81	R.WDKDIMLIR.L
1205.6624	1204.6551	1204.6274	23.0	109 -	117	1	(33)	R.WDKDIMLIR.L + Oxidation (M)
1208.6590	1207.6517	1207.6271	20.4	146 -	156	0	---	R.IMGWGTITTTK.V
1592.8032	1591.7960	1591.7698	16.4	189 -	203	0	110	R.TLCAGILQGGIDSCK.V
2113.0883	2112.0810	2112.0462	16.5	50 -	67	0	174	R.FYCAGTLINQEWVLTAAR.C

No match to: 1077.4785, 1172.6386, 1186.6312, 1212.6257, 1215.6815, 1221.6559, 1230.6987, 1231.6710, 1232.1392, 1234.6802, 1237.6524, 1246.6827, 1304.6060, 1320.5943, 1330.6201, 1346.6114, 1372.7216, 1429.7399, 1450.7394, 1632.6900, 1642.9239, 1649.7186, 1665.7056, 1731.9567, 1802.9521, 1852.0286, 1997.0124, 2022.0758, 2038.0531, 2053.0494, 2056.0667, 2066.0564, 2069.0742, 2075.0641, 2080.0660, 2094.0792, 2106.0776, 2109.0960, 2111.0719, 2118.0892, 2124.0832, 2127.0923, 2130.0855, 2134.0649, 2137.0790, 2139.1052, 2145.0792, 2155.1018, 2159.0676, 2161.0764, 2171.0968, 2187.0862, 2257.1391, 2265.0817, 2285.1075, 2300.1160, 2371.2527, 2386.2189, 2405.2424, 2418.2571, 2442.1046, 2443.2546, 2447.4393, 2455.2529, 2458.2475, 2462.2618, 2473.2683, 2476.2783, 2485.1932, 2488.2746, 2494.2292, 2501.1388, 2504.2755, 2510.1608, 2513.1576, 2518.8602, 2519.2909, 2530.2932, 2535.2088, 2539.1622, 2551.1621, 2556.0916, 2558.1673, 2569.1725, 2572.1856, 2574.1490, 2575.3485, 2584.1815, 2590.1477, 2600.1769, 2968.4276, 3096.5257, 3540.7719

5. VSPH1_VIPAA **Mass:** 29593 **Score:** 399 **Expect:** 1.8e-037 **Matches:** 7

Vaa serine proteinase homolog 1 OS=Vipera ammodytes ammodytes OX=8705 PE=1 SV=1

Observed	Mr(expt)	Mr(calc)	ppm	Start	End	Miss	Ions	Peptide
866.5712	865.5640	865.5385	29.4	75 -	82	0	---	R.IILGVHSK.N
888.4510	887.4438	887.4211	25.5	98 -	104	0	7	K.FFCLSSK.T
1189.6689	1188.6617	1188.6325	24.5	109 -	117	1	81	R.WDKDIMLIR.L
1205.6624	1204.6551	1204.6274	23.0	109 -	117	1	(33)	R.WDKDIMLIR.L + Oxidation (M)
1208.6590	1207.6517	1207.6271	20.4	146 -	156	0	---	R.IMGWGTITTTK.V
1592.8032	1591.7960	1591.7698	16.4	189 -	203	0	110	R.TLCAGILQGGIDSCK.V
2113.0883	2112.0810	2112.0462	16.5	50 -	67	0	174	R.FYCAGTLINQEWVLTAAR.C

No match to: 1077.4785, 1172.6386, 1186.6312, 1212.6257, 1215.6815, 1221.6559, 1230.6987, 1231.6710, 1232.1392, 1234.6802, 1237.6524, 1246.6827, 1304.6060, 1320.5943, 1330.6201, 1346.6114, 1372.7216, 1429.7399, 1450.7394, 1632.6900, 1642.9239, 1649.7186, 1665.7056, 1731.9567, 1802.9521, 1852.0286, 1997.0124, 2022.0758, 2038.0531, 2053.0494, 2056.0667, 2066.0564, 2069.0742, 2075.0641, 2080.0660, 2094.0792, 2106.0776, 2109.0960, 2111.0719, 2118.0892, 2124.0832, 2127.0923, 2130.0855, 2134.0649, 2137.0790, 2139.1052, 2145.0792, 2155.1018, 2159.0676, 2161.0764, 2171.0968, 2187.0862, 2257.1391, 2265.0817, 2285.1075, 2300.1160, 2371.2527, 2386.2189, 2405.2424, 2418.2571, 2442.1046, 2443.2546, 2447.4393, 2455.2529, 2458.2475, 2462.2618, 2473.2683, 2476.2783, 2485.1932, 2488.2746, 2494.2292, 2501.1388, 2504.2755, 2510.1608, 2513.1576, 2518.8602, 2519.2909, 2530.2932, 2535.2088, 2539.1622, 2551.1621, 2556.0916, 2558.1673, 2569.1725, 2572.1856, 2574.1490, 2575.3485, 2584.1815, 2590.1477, 2600.1769, 2968.4276, 3096.5257, 3540.7719

6. A0A6G5ZUW7_9SAUR **Mass:** 22103 **Score:** 105 **Expect:** 4.5e-008 **Matches:** 5

Serine protease 10 (Fragment) OS=Vipera anatolica senliki OX=2604287 PE=2 SV=1

Observed	Mr(expt)	Mr(calc)	ppm	Start	End	Miss	Ions	Peptide
866.5712	865.5640	865.5385	29.4	72 -	79	0	---	R.IILGVHSK.N
888.4510	887.4438	887.4211	25.5	95 -	101	0	7	K.FFCLSSK.S
1189.6689	1188.6617	1188.6325	24.5	106 -	114	1	81	R.WDKDIMLIR.L
1205.6624	1204.6551	1204.6274	23.0	106 -	114	1	(33)	R.WDKDIMLIR.L + Oxidation (M)
1208.6590	1207.6517	1207.6271	20.4	143 -	153	0	---	R.IMGWGTITTK.V

No match to: 1077.4785, 1172.6386, 1186.6312, 1212.6257, 1215.6815, 1221.6559, 1230.6987, 1231.6710, 1232.1392, 1234.6802, 1237.6524, 1246.6827, 1304.6060, 1320.5943, 1330.6201, 1346.6114, 1372.7216, 1429.7399, 1450.7394, 1592.8032, 1632.6900, 1642.9239, 1649.7186, 1665.7056, 1731.9567, 1802.9521, 1852.0286, 1997.0124, 2022.0758, 2038.0531, 2053.0494, 2056.0667, 2066.0564, 2069.0742, 2075.0641, 2080.0660, 2094.0792, 2106.0776, 2109.0960, 2111.0719, 2113.0883, 2118.0892, 2124.0832, 2127.0923, 2130.0855, 2134.0649, 2137.0790, 2139.1052, 2145.0792, 2155.1018, 2159.0676, 2161.0764, 2171.0968, 2187.0862, 2257.1391, 2265.0817, 2285.1075, 2300.1160, 2371.2527, 2386.2189, 2405.2424, 2418.2571, 2442.1046, 2443.2546, 2447.4393, 2455.2529, 2458.2475, 2462.2618, 2473.2683, 2476.2783, 2485.1932, 2488.2746, 2494.2292, 2501.1388, 2504.2755, 2510.1608, 2513.1576, 2518.8602, 2519.2909, 2530.2932, 2535.2088, 2539.1622, 2551.1621, 2556.0916, 2558.1673, 2569.1725, 2572.1856, 2574.1490, 2575.3485, 2584.1815, 2590.1477, 2600.1769, 2968.4276, 3096.5257, 3540.7719

7. A0A6B7FMQ3_VIPAA **Mass:** 28909 **Score:** 90 **Expect:** 1.3e-006 **Matches:** 3

Snake venom serine protease OS=Vipera ammodytes ammodytes OX=8705 PE=2 SV=1

Observed	Mr(expt)	Mr(calc)	ppm	Start	End	Miss	Ions	Peptide
888.4510	887.4438	887.4211	25.5	95 -	101	0	7	K.FFCLSSK.N
1189.6689	1188.6617	1188.6325	24.5	106 -	114	1	81	K.WDKDIMLIR.L
1205.6624	1204.6551	1204.6274	23.0	106 -	114	1	(33)	K.WDKDIMLIR.L + Oxidation (M)

No match to: 866.5712, 1077.4785, 1172.6386, 1186.6312, 1208.6590, 1212.6257, 1215.6815, 1221.6559, 1230.6987, 1231.6710, 1232.1392, 1234.6802, 1237.6524, 1246.6827, 1304.6060, 1320.5943, 1330.6201, 1346.6114, 1372.7216, 1429.7399, 1450.7394, 1592.8032, 1632.6900, 1642.9239, 1649.7186, 1665.7056, 1731.9567, 1802.9521, 1852.0286, 1997.0124, 2022.0758, 2038.0531, 2053.0494, 2056.0667, 2066.0564, 2069.0742, 2075.0641, 2080.0660, 2094.0792, 2106.0776, 2109.0960, 2111.0719, 2113.0883, 2118.0892, 2124.0832, 2127.0923, 2130.0855, 2134.0649, 2137.0790, 2139.1052, 2145.0792, 2155.1018, 2159.0676, 2161.0764, 2171.0968, 2187.0862, 2257.1391, 2265.0817, 2285.1075, 2300.1160, 2371.2527, 2386.2189, 2405.2424, 2418.2571, 2442.1046, 2443.2546, 2447.4393, 2455.2529, 2458.2475, 2462.2618, 2473.2683, 2476.2783, 2485.1932, 2488.2746, 2494.2292, 2501.1388, 2504.2755, 2510.1608, 2513.1576, 2518.8602, 2519.2909, 2530.2932, 2535.2088, 2539.1622, 2551.1621, 2556.0916, 2558.1673, 2569.1725, 2572.1856, 2574.1490, 2575.3485, 2584.1815, 2590.1477, 2600.1769, 2968.4276, 3096.5257, 3540.7719

8. D8MIA1_BITRH **Mass:** 29504 **Score:** 88 **Expect:** 2e-006 **Matches:** 3

Rhinocerase 3 protein (Fragment) OS=Bitis rhinoceros OX=715877 GN=rhinocerase 3 PE=3 SV=1

Observed	Mr(expt)	Mr(calc)	ppm	Start	End	Miss	Ions	Peptide
1189.6689	1188.6617	1188.6325	24.5	108 -	116	1	81	R.WDKDIMLIR.L
1205.6624	1204.6551	1204.6274	23.0	108 -	116	1	(33)	R.WDKDIMLIR.L + Oxidation (M)
2968.4276	2967.4204	2967.5713	-50.85	117 -	144	1	---	R.LKRPVNDGTHIAPLSLPSNPPSVGSVCR.I

No match to: 866.5712, 888.4510, 1077.4785, 1172.6386, 1186.6312, 1208.6590, 1212.6257, 1215.6815, 1221.6559, 1230.6987, 1231.6710, 1232.1392, 1234.6802, 1237.6524, 1246.6827, 1304.6060, 1320.5943, 1330.6201, 1346.6114, 1372.7216, 1429.7399, 1450.7394, 1592.8032, 1632.6900, 1642.9239, 1649.7186, 1665.7056, 1731.9567, 1802.9521, 1852.0286, 1997.0124, 2022.0758, 2038.0531, 2053.0494, 2056.0667, 2066.0564, 2069.0742, 2075.0641, 2080.0660, 2094.0792, 2106.0776, 2109.0960, 2111.0719, 2113.0883, 2118.0892, 2124.0832, 2127.0923, 2130.0855, 2134.0649, 2137.0790, 2139.1052, 2145.0792, 2155.1018, 2159.0676, 2161.0764, 2171.0968, 2187.0862, 2257.1391, 2265.0817, 2285.1075, 2300.1160, 2371.2527, 2386.2189, 2405.2424, 2418.2571, 2442.1046, 2443.2546, 2447.4393, 2455.2529, 2458.2475, 2462.2618, 2473.2683, 2476.2783, 2485.1932, 2488.2746, 2494.2292, 2501.1388, 2504.2755, 2510.1608, 2513.1576, 2518.8602, 2519.2909, 2530.2932, 2535.2088, 2539.1622, 2551.1621, 2556.0916, 2558.1673, 2569.1725, 2572.1856, 2574.1490, 2575.3485, 2584.1815, 2590.1477, 2600.1769, 3096.5257, 3540.7719

9. D8MIA0_BITRH **Mass:** 29517 **Score:** 88 **Expect:** 2e-006 **Matches:** 3

Rhinocerase 2 protein (Fragment) OS=Bitis rhinoceros OX=715877 GN=rhinocerase 2 PE=3 SV=1

Observed	Mr(expt)	Mr(calc)	ppm	Start	End	Miss	Ions	Peptide
1189.6689	1188.6617	1188.6325	24.5	108 -	116	1	81	R.WDKDIMLIR.L
1205.6624	1204.6551	1204.6274	23.0	108 -	116	1	(33)	R.WDKDIMLIR.L + Oxidation (M)
2968.4276	2967.4204	2967.5713	-50.85	117 -	144	1	---	R.LKRPVNDGTHIAPLSLPSNPPSVGSVCR.I

No match to: 866.5712, 888.4510, 1077.4785, 1172.6386, 1186.6312, 1208.6590, 1212.6257, 1215.6815, 1221.6559, 1230.6987, 1231.6710, 1232.1392, 1234.6802, 1237.6524, 1246.6827, 1304.6060, 1320.5943, 1330.6201, 1346.6114, 1372.7216, 1429.7399, 1450.7394, 1592.8032, 1632.6900, 1642.9239, 1649.7186, 1665.7056, 1731.9567, 1802.9521, 1852.0286, 1997.0124, 2022.0758, 2038.0531, 2053.0494, 2056.0667, 2066.0564, 2069.0742, 2075.0641, 2080.0660, 2094.0792, 2106.0776, 2109.0960, 2111.0719, 2113.0883, 2118.0892, 2124.0832, 2127.0923, 2130.0855, 2134.0649, 2137.0790, 2139.1052, 2145.0792, 2155.1018, 2159.0676, 2161.0764, 2171.0968, 2187.0862, 2257.1391, 2265.0817, 2285.1075, 2300.1160, 2371.2527, 2386.2189, 2405.2424, 2418.2571, 2442.1046, 2443.2546, 2447.4393, 2455.2529, 2458.2475, 2462.2618, 2473.2683, 2476.2783, 2485.1932, 2488.2746, 2494.2292, 2501.1388, 2504.2755, 2510.1608, 2513.1576, 2518.8602, 2519.2909, 2530.2932, 2535.2088, 2539.1622, 2551.1621, 2556.0916, 2558.1673, 2569.1725, 2572.1856, 2574.1490, 2575.3485, 2584.1815, 2590.1477, 2600.1769, 3096.5257, 3540.7719

10. VSPY_MACLB **Mass:** 28944 **Score:** 78 **Expect:** 2e-005 **Matches:** 2

Chymotrypsin-like protease VLCTLP OS=Macrovipera lebetina OX=8709 PE=1 SV=1

Observed	Mr(expt)	Mr(calc)	ppm	Start	End	Miss	Ions	Peptide

1189.6689	1188.6617	1188.6325	24.5	106 -	114	1	81	K.WDKDIMLIR.L
1205.6624	1204.6551	1204.6274	23.0	106 -	114	1	(33)	K.WDKDIMLIR.L + Oxidation (M)

No match to: 866.5712, 888.4510, 1077.4785, 1172.6386, 1186.6312, 1208.6590, 1212.6257, 1215.6815, 1221.6559, 1230.6987, 1231.6710, 1232.1392, 1234.6802, 1237.6524, 1246.6827, 1304.6060, 1320.5943, 1330.6201, 1346.6114, 1372.7216, 1429.7399, 1450.7394, 1592.8032, 1632.6900, 1642.9239, 1649.7186, 1665.7056, 1731.9567, 1802.9521, 1852.0286, 1997.0124, 2022.0758, 2038.0531, 2053.0494, 2056.0667, 2066.0564, 2069.0742, 2075.0641, 2080.0660, 2094.0792, 2106.0776, 2109.0960, 2111.0719, 2113.0883, 2118.0892, 2124.0832, 2127.0923, 2130.0855, 2134.0649, 2137.0790, 2139.1052, 2145.0792, 2155.1018, 2159.0676, 2161.0764, 2171.0968, 2187.0862, 2257.1391, 2265.0817, 2285.1075, 2300.1160, 2371.2527, 2386.2189, 2405.2424, 2418.2571, 2442.1046, 2443.2546, 2447.4393, 2455.2529, 2458.2475, 2462.2618, 2473.2683, 2476.2783, 2485.1932, 2488.2746, 2494.2292, 2501.1388, 2504.2755, 2510.1608, 2513.1576, 2518.8602, 2519.2909, 2530.2932, 2535.2088, 2539.1622, 2551.1621, 2556.0916, 2558.1673, 2569.1725, 2572.1856, 2574.1490, 2575.3485, 2584.1815, 2590.1477, 2600.1769, 2968.4276, 3096.5257, 3540.7719

11. Q6A373_VIPAA **Mass:** 16390 **Score:** 26 **Expect:** 3.9 **Matches:** 4
Ammodytin L(1'') variant OS=Vipera ammodytes ammodytes OX=8705 PE=2 SV=1

Observed	Mr(expt)	Mr(calc)	ppm	Start	End	Miss	Ions	Peptide
1246.6827	1245.6754	1245.6248	40.7	70 -	79	1	---	K.LSDCRPKTNR.Y
2075.0641	2074.0568	2073.9037	73.8	32 -	49	0	---	K.NPLTSYSFYGCHCGLGNK.G
2155.1018	2154.0945	2153.9656	59.8	100 -	117	1	---	K.QICECDGAAAICFRENLK.T
2458.2475	2457.2402	2457.1206	48.7	32 -	53	1	---	K.NPLTSYSFYGCHCGLGNKGTPK.D

No match to: 866.5712, 888.4510, 1077.4785, 1172.6386, 1186.6312, 1189.6689, 1205.6624, 1208.6590, 1212.6257, 1215.6815, 1221.6559, 1230.6987, 1231.6710, 1232.1392, 1234.6802, 1237.6524, 1304.6060, 1320.5943, 1330.6201, 1346.6114, 1372.7216, 1429.7399, 1450.7394, 1592.8032, 1632.6900, 1642.9239, 1649.7186, 1665.7056, 1731.9567, 1802.9521, 1852.0286, 1997.0124, 2022.0758, 2038.0531, 2053.0494, 2056.0667, 2066.0564, 2069.0742, 2080.0660, 2094.0792, 2106.0776, 2109.0960, 2111.0719, 2113.0883, 2118.0892, 2124.0832, 2127.0923, 2130.0855, 2134.0649, 2137.0790, 2139.1052, 2145.0792, 2159.0676, 2161.0764, 2171.0968, 2187.0862, 2257.1391, 2265.0817, 2285.1075, 2300.1160, 2371.2527, 2386.2189, 2405.2424, 2418.2571, 2442.1046, 2443.2546, 2447.4393, 2455.2529, 2462.2618, 2473.2683, 2476.2783, 2485.1932, 2488.2746, 2494.2292, 2501.1388, 2504.2755, 2510.1608, 2513.1576, 2518.8602, 2519.2909, 2530.2932, 2535.2088, 2539.1622, 2551.1621, 2556.0916, 2558.1673, 2569.1725, 2572.1856, 2574.1490, 2575.3485, 2584.1815, 2590.1477, 2600.1769, 2968.4276, 3096.5257, 3540.7719

12. A0A6G5ZUN8_9SAUR **Mass:** 61639 **Score:** 24 **Expect:** 5.1 **Matches:** 8
Atriopeptidase (Fragment) OS=Vipera anatolica senliki OX=2604287 PE=2 SV=1

Observed	Mr(expt)	Mr(calc)	ppm	Start	End	Miss	Ions	Peptide
1208.6590	1207.6517	1207.5985	44.0	363 -	371	0	---	R.EIQNYISWR.Y
1246.6827	1245.6754	1245.6201	44.5	126 -	137	1	---	K.DSNDIDAVAKAK.T
2022.0758	2021.0686	2021.1084	-19.74	337 -	353	1	---	K.NTEDIIVYAPEYLIKLK.S
2053.0494	2052.0421	2051.9503	44.7	2 -	19	1	---	M.AKSESQMDITEMNSPKPK.R + 2 Oxidation (M)
2127.0923	2126.0850	2126.0603	11.6	255 -	272	1	---	R.QERNLPINDTEISIEAER.I
2130.0855	2129.0783	2129.0316	21.9	501 -	517	1	---	K.EEEYFENLIENLKFGQK.E
2968.4276	2967.4204	2967.2877	44.7	69 -	93	0	---	R.IIENMDTTAEPCSNFYQYACGGWLK.K
3096.5257	3095.5184	3095.3827	43.8	69 -	94	1	---	R.IIENMDTTAEPCSNFYQYACGGWLKK.H

No match to: 866.5712, 888.4510, 1077.4785, 1172.6386, 1186.6312, 1189.6689, 1205.6624, 1212.6257, 1215.6815, 1221.6559, 1230.6987, 1231.6710, 1232.1392, 1234.6802, 1237.6524, 1304.6060, 1320.5943, 1330.6201, 1346.6114, 1372.7216, 1429.7399, 1450.7394, 1592.8032, 1632.6900, 1642.9239, 1649.7186, 1665.7056, 1731.9567, 1802.9521, 1852.0286, 1997.0124, 2038.0531, 2056.0667, 2066.0564, 2069.0742, 2075.0641, 2080.0660, 2094.0792, 2106.0776, 2109.0960, 2111.0719, 2113.0883, 2118.0892, 2124.0832, 2134.0649, 2137.0790, 2139.1052, 2145.0792, 2155.1018, 2159.0676, 2161.0764, 2171.0968, 2187.0862, 2257.1391, 2265.0817, 2285.1075, 2300.1160, 2371.2527, 2386.2189, 2405.2424, 2418.2571, 2442.1046, 2443.2546, 2447.4393, 2455.2529, 2458.2475, 2462.2618, 2473.2683, 2476.2783, 2485.1932, 2488.2746, 2494.2292, 2501.1388, 2504.2755, 2510.1608, 2513.1576, 2518.8602, 2519.2909, 2530.2932, 2535.2088, 2539.1622, 2551.1621, 2556.0916, 2558.1673, 2569.1725, 2572.1856, 2574.1490, 2575.3485, 2584.1815, 2590.1477, 2600.1769, 3540.7719

13. A0A6G5ZW48_9SAUR **Mass:** 86121 **Score:** 22 **Expect:** 8 **Matches:** 9
Atriopeptidase OS=Vipera anatolica senliki OX=2604287 PE=2 SV=1

Observed	Mr(expt)	Mr(calc)	ppm	Start	End	Miss	Ions	Peptide
1208.6590	1207.6517	1207.5985	44.0	363 -	371	0	---	R.EIQNYISWR.Y
1246.6827	1245.6754	1245.6201	44.5	126 -	137	1	---	K.DSNDIDAVAKAK.T
2022.0758	2021.0686	2021.1084	-19.74	337 -	353	1	---	K.NTEDIIVYAPEYLIKLK.S
2053.0494	2052.0421	2051.9503	44.7	2 -	19	1	---	M.AKSESQMDITEMNSPKPK.R + 2 Oxidation (M)
2127.0923	2126.0850	2126.0603	11.6	255 -	272	1	---	R.QERNLPINDTEISIEAER.I
2130.0855	2129.0783	2129.0316	21.9	501 -	517	1	---	K.EEEYFENLIENLKFGQK.E
2300.1160	2299.1087	2299.1154	-2.93	716 -	735	1	---	R.VLGSLQNNLEFSEAFKCTDK.D
2968.4276	2967.4204	2967.2877	44.7	69 -	93	0	---	R.IIENMDTTAEPCSNFYQYACGGWLK.K
3096.5257	3095.5184	3095.3827	43.8	69 -	94	1	---	R.IIENMDTTAEPCSNFYQYACGGWLKK.H

No match to: 866.5712, 888.4510, 1077.4785, 1172.6386, 1186.6312, 1189.6689, 1205.6624, 1212.6257, 1215.6815, 1221.6559, 1230.6987, 1231.6710, 1232.1392, 1234.6802, 1237.6524, 1304.6060, 1320.5943, 1330.6201, 1346.6114, 1372.7216, 1429.7399, 1450.7394, 1592.8032, 1632.6900, 1642.9239, 1649.7186, 1665.7056, 1731.9567, 1802.9521, 1852.0286, 1997.0124, 2038.0531, 2056.0667, 2066.0564, 2069.0742, 2075.0641, 2080.0660, 2094.0792, 2106.0776, 2109.0960, 2111.0719, 2113.0883, 2118.0892, 2124.0832, 2134.0649, 2137.0790, 2139.1052, 2145.0792, 2155.1018, 2159.0676, 2161.0764, 2171.0968, 2187.0862, 2257.1391, 2265.0817, 2285.1075, 2371.2527, 2386.2189, 2405.2424, 2418.2571, 2442.1046, 2443.2546, 2447.4393, 2455.2529, 2458.2475, 2462.2618, 2473.2683, 2476.2783, 2485.1932, 2488.2746, 2494.2292, 2501.1388, 2504.2755, 2510.1608, 2513.1576, 2518.8602, 2519.2909, 2530.2932, 2535.2088, 2539.1622, 2551.1621, 2556.0916, 2558.1673, 2569.1725, 2572.1856, 2574.1490, 2575.3485, 2584.1815, 2590.1477, 2600.1769, 3540.7719

14. A0A6G5ZW27_9SAUR **Mass:** 38471 **Score:** 19 **Expect:** 17 **Matches:** 5
Venom factor 6 (Fragment) OS=Vipera anatolica senliki OX=2604287 PE=2 SV=1

Observed	Mr(expt)	Mr(calc)	ppm	Start	End	Miss	Ions	Peptide
1212.6257	1211.6184	1211.6550	-30.22	140 -	149	1	---	K.AVYVLNDKYK.I
1304.6060	1303.5987	1303.6884	-68.82	223 -	233	1	---	K.VAEFQDQALRK.C
2442.1046	2441.0973	2441.3101	-87.15	51 -	72	1	---	R.GPGQNLVTMTLRITPDLIPSFR.F + Oxidation (M)
2476.2783	2475.2710	2475.1021	68.2	254 -	273	1	---	K.YIQEGDACKAAFLECCHYIK.G
2488.2746	2487.2673	2487.2322	14.1	73 -	94	0	---	R.FVAYYQVGNSEIVADSVWVDVK.D

No match to: 866.5712, 888.4510, 1077.4785, 1172.6386, 1186.6312, 1189.6689, 1205.6624, 1208.6590, 1215.6815, 1221.6559, 1230.6987, 1231.6710, 1232.1392, 1234.6802, 1237.6524, 1246.6827, 1320.5943, 1330.6201, 1346.6114, 1372.7216, 1429.7399, 1450.7394, 1592.8032, 1632.6900, 1642.9239, 1649.7186, 1665.7056, 1731.9567, 1802.9521, 1852.0286, 1997.0124, 2022.0758, 2038.0531, 2053.0494, 2056.0667, 2066.0564, 2069.0742, 2075.0641, 2080.0660, 2094.0792, 2106.0776, 2109.0960, 2111.0719, 2113.0883, 2118.0892, 2124.0832, 2127.0923, 2130.0855, 2134.0649, 2137.0790, 2139.1052, 2145.0792, 2155.1018, 2159.0676, 2161.0764, 2171.0968, 2187.0862, 2257.1391, 2265.0817, 2285.1075, 2300.1160, 2371.2527, 2386.2189, 2405.2424, 2418.2571, 2443.2546, 2447.4393, 2455.2529, 2458.2475, 2462.2618, 2473.2683, 2485.1932, 2494.2292, 2501.1388, 2504.2755, 2510.1608, 2513.1576, 2518.8602, 2519.2909, 2530.2932, 2535.2088, 2539.1622, 2551.1621, 2556.0916, 2558.1673, 2569.1725, 2572.1856, 2574.1490, 2575.3485, 2584.1815, 2590.1477, 2600.1769, 2968.4276, 3096.5257, 3540.7719

15. PA2HL_VIPAA **Mass:** 16481 **Score:** 19 **Expect:** 17 **Matches:** 3
Basic phospholipase A2 homolog ammodytin L OS=Vipera ammodytes ammodytes OX=8705 PE=1 SV=1

Observed	Mr(expt)	Mr(calc)	ppm	Start	End	Miss	Ions	Peptide
2075.0641	2074.0568	2073.9037	73.8	32 -	49	0	---	K.NPLTSYSFYGCHCGLGNK.G
2458.2475	2457.2402	2457.3818	-57.60	2 -	23	1	---	M.RILWIVAVCLIGVEGSVIEFGK.M
2485.1932	2484.1860	2484.1678	7.30	32 -	53	1	---	K.NPLTSYSFYGCHCGLGNKGKPK.D

No match to: 866.5712, 888.4510, 1077.4785, 1172.6386, 1186.6312, 1189.6689, 1205.6624, 1208.6590, 1212.6257, 1215.6815, 1221.6559, 1230.6987, 1231.6710, 1232.1392, 1234.6802, 1237.6524, 1246.6827, 1304.6060, 1320.5943, 1330.6201, 1346.6114, 1372.7216, 1429.7399, 1450.7394, 1592.8032, 1632.6900, 1642.9239, 1649.7186, 1665.7056, 1731.9567, 1802.9521, 1852.0286, 1997.0124, 2022.0758, 2038.0531,

2053.0494, 2056.0667, 2066.0564, 2069.0742, 2080.0660, 2094.0792, 2106.0776, 2109.0960, 2111.0719, 2113.0883, 2118.0892, 2124.0832, 2127.0923, 2130.0855, 2134.0649, 2137.0790, 2139.1052, 2145.0792, 2155.1018, 2159.0676, 2161.0764, 2171.0968, 2187.0862, 2257.1391, 2265.0817, 2285.1075, 2300.1160, 2371.2527, 2386.2189, 2405.2424, 2418.2571, 2442.1046, 2443.2546, 2447.4393, 2455.2529, 2462.2618, 2473.2683, 2476.2783, 2488.2746, 2494.2292, 2501.1388, 2504.2755, 2510.1608, 2513.1576, 2518.8602, 2519.2909, 2530.2932, 2535.2088, 2539.1622, 2551.1621, 2556.0916, 2558.1673, 2569.1725, 2572.1856, 2574.1490, 2575.3485, 2584.1815, 2590.1477, 2600.1769, 2968.4276, 3096.5257, 3540.7719

16. Q6A371_VIPAM **Mass:** 16461 **Score:** 19 **Expect:** 17 **Matches:** 3

Ammodytin L(3) isoform OS=Vipera ammodytes montandoni OX=235554 PE=2 SV=1

Observed	Mr(expt)	Mr(calc)	ppm	Start	End	Miss	Ions	Peptide
2075.0641	2074.0568	2073.9037	73.8	32 -	49	0	---	K.NPITSYSFYGCHCGLGNK.G
2458.2475	2457.2402	2457.3818	-57.60	2 -	23	1	---	M.RILWIVAVCLIGVEGSVIEFGK.M
2485.1932	2484.1860	2484.1678	7.30	32 -	53	1	---	K.NPITSYSFYGCHCGLGNKGKPK.D

No match to: 866.5712, 888.4510, 1077.4785, 1172.6386, 1186.6312, 1189.6689, 1205.6624, 1208.6590, 1212.6257, 1215.6815, 1221.6559, 1230.6987, 1231.6710, 1232.1392, 1234.6802, 1237.6524, 1246.6827, 1304.6060, 1320.5943, 1330.6201, 1346.6114, 1372.7216, 1429.7399, 1450.7394, 1592.8032, 1632.6900, 1642.9239, 1649.7186, 1665.7056, 1731.9567, 1802.9521, 1852.0286, 1997.0124, 2022.0758, 2038.0531, 2053.0494, 2056.0667, 2066.0564, 2069.0742, 2080.0660, 2094.0792, 2106.0776, 2109.0960, 2111.0719, 2113.0883, 2118.0892, 2124.0832, 2127.0923, 2130.0855, 2134.0649, 2137.0790, 2139.1052, 2145.0792, 2155.1018, 2159.0676, 2161.0764, 2171.0968, 2187.0862, 2257.1391, 2265.0817, 2285.1075, 2300.1160, 2371.2527, 2386.2189, 2405.2424, 2418.2571, 2442.1046, 2443.2546, 2447.4393, 2455.2529, 2462.2618, 2473.2683, 2476.2783, 2488.2746, 2494.2292, 2501.1388, 2504.2755, 2510.1608, 2513.1576, 2518.8602, 2519.2909, 2530.2932, 2535.2088, 2539.1622, 2551.1621, 2556.0916, 2558.1673, 2569.1725, 2572.1856, 2574.1490, 2575.3485, 2584.1815, 2590.1477, 2600.1769, 2968.4276, 3096.5257, 3540.7719

17. Q6A382_VIPAA **Mass:** 16431 **Score:** 19 **Expect:** 17 **Matches:** 3

Ammodytin L(1'') variant OS=Vipera ammodytes ammodytes OX=8705 PE=2 SV=1

Observed	Mr(expt)	Mr(calc)	ppm	Start	End	Miss	Ions	Peptide
2075.0641	2074.0568	2073.9037	73.8	32 -	49	0	---	K.NPLTSYSFYGCHCGLGNK.G
2418.2571	2417.2498	2417.3141	-26.60	2 -	23	1	---	M.RTLWIVAVCLIGVEGSAIEFGK.M
2485.1932	2484.1860	2484.1678	7.30	32 -	53	1	---	K.NPLTSYSFYGCHCGLGNKGKPK.D

No match to: 866.5712, 888.4510, 1077.4785, 1172.6386, 1186.6312, 1189.6689, 1205.6624, 1208.6590, 1212.6257, 1215.6815, 1221.6559, 1230.6987, 1231.6710, 1232.1392, 1234.6802, 1237.6524, 1246.6827, 1304.6060, 1320.5943, 1330.6201, 1346.6114, 1372.7216, 1429.7399, 1450.7394, 1592.8032, 1632.6900, 1642.9239, 1649.7186, 1665.7056, 1731.9567, 1802.9521, 1852.0286, 1997.0124, 2022.0758, 2038.0531, 2053.0494, 2056.0667, 2066.0564, 2069.0742, 2080.0660, 2094.0792, 2106.0776, 2109.0960, 2111.0719, 2113.0883, 2118.0892, 2124.0832, 2127.0923, 2130.0855, 2134.0649, 2137.0790, 2139.1052, 2145.0792, 2155.1018, 2159.0676, 2161.0764, 2171.0968, 2187.0862, 2257.1391, 2265.0817, 2285.1075, 2300.1160, 2371.2527, 2386.2189, 2405.2424, 2442.1046, 2443.2546, 2447.4393, 2455.2529, 2458.2475, 2462.2618, 2473.2683, 2476.2783, 2488.2746, 2494.2292, 2501.1388, 2504.2755, 2510.1608, 2513.1576, 2518.8602, 2519.2909, 2530.2932, 2535.2088, 2539.1622, 2551.1621, 2556.0916, 2558.1673, 2569.1725, 2572.1856, 2574.1490, 2575.3485, 2584.1815, 2590.1477, 2600.1769, 2968.4276, 3096.5257, 3540.7719

18. Q6A367_VIPAM **Mass:** 16403 **Score:** 19 **Expect:** 17 **Matches:** 3

Ammodytin L(3) variant OS=Vipera ammodytes montandoni OX=235554 PE=2 SV=1

Observed	Mr(expt)	Mr(calc)	ppm	Start	End	Miss	Ions	Peptide
2075.0641	2074.0568	2073.9037	73.8	32 -	49	0	---	K.NPITSYSFYGCHCGLGNK.G
2458.2475	2457.2402	2457.3818	-57.60	2 -	23	1	---	M.RILWIVAVCLIGVEGSVIEFGK.M
2485.1932	2484.1860	2484.1678	7.30	32 -	53	1	---	K.NPITSYSFYGCHCGLGNKGKPK.G

No match to: 866.5712, 888.4510, 1077.4785, 1172.6386, 1186.6312, 1189.6689, 1205.6624, 1208.6590, 1212.6257, 1215.6815, 1221.6559, 1230.6987, 1231.6710, 1232.1392, 1234.6802, 1237.6524, 1246.6827, 1304.6060, 1320.5943, 1330.6201, 1346.6114, 1372.7216, 1429.7399, 1450.7394, 1592.8032, 1632.6900, 1642.9239, 1649.7186, 1665.7056, 1731.9567, 1802.9521, 1852.0286, 1997.0124, 2022.0758, 2038.0531, 2053.0494, 2056.0667, 2066.0564, 2069.0742, 2080.0660, 2094.0792, 2106.0776, 2109.0960, 2111.0719, 2113.0883, 2118.0892, 2124.0832, 2127.0923, 2130.0855, 2134.0649, 2137.0790, 2139.1052, 2145.0792, 2155.1018, 2159.0676, 2161.0764, 2171.0968, 2187.0862, 2257.1391, 2265.0817, 2285.1075, 2300.1160, 2371.2527, 2386.2189, 2405.2424, 2418.2571, 2442.1046, 2443.2546, 2447.4393, 2455.2529, 2462.2618, 2473.2683, 2476.2783, 2488.2746, 2494.2292, 2501.1388, 2504.2755, 2510.1608, 2513.1576, 2518.8602, 2519.2909, 2530.2932, 2535.2088, 2539.1622, 2551.1621, 2556.0916, 2558.1673, 2569.1725, 2572.1856, 2574.1490, 2575.3485, 2584.1815, 2590.1477, 2600.1769, 2968.4276, 3096.5257, 3540.7719

19. Q6A369_VIPAM **Mass:** 16449 **Score:** 19 **Expect:** 17 **Matches:** 3

Ammodytin L(3) variant OS=Vipera ammodytes montandoni OX=235554 PE=2 SV=1

Observed	Mr(expt)	Mr(calc)	ppm	Start	End	Miss	Ions	Peptide
2075.0641	2074.0568	2073.9037	73.8	32 -	49	0	---	K.NPITSYSFYGCHCGLGNK.G
2458.2475	2457.2402	2457.3818	-57.60	2 -	23	1	---	M.RILWIVAVCLIGVEGSVIEFGK.M
2485.1932	2484.1860	2484.1678	7.30	32 -	53	1	---	K.NPITSYSFYGCHCGLGNKGKPK.D

No match to: 866.5712, 888.4510, 1077.4785, 1172.6386, 1186.6312, 1189.6689, 1205.6624, 1208.6590, 1212.6257, 1215.6815, 1221.6559, 1230.6987, 1231.6710, 1232.1392, 1234.6802, 1237.6524, 1246.6827, 1304.6060, 1320.5943, 1330.6201, 1346.6114, 1372.7216, 1429.7399, 1450.7394, 1592.8032, 1632.6900, 1642.9239, 1649.7186, 1665.7056, 1731.9567, 1802.9521, 1852.0286, 1997.0124, 2022.0758, 2038.0531, 2053.0494, 2056.0667, 2066.0564, 2069.0742, 2080.0660, 2094.0792, 2106.0776, 2109.0960, 2111.0719, 2113.0883, 2118.0892, 2124.0832, 2127.0923, 2130.0855, 2134.0649, 2137.0790, 2139.1052, 2145.0792, 2155.1018, 2159.0676, 2161.0764, 2171.0968, 2187.0862, 2257.1391, 2265.0817, 2285.1075, 2300.1160, 2371.2527, 2386.2189, 2405.2424, 2418.2571, 2442.1046, 2443.2546, 2447.4393, 2455.2529, 2462.2618, 2473.2683, 2476.2783, 2488.2746, 2494.2292, 2501.1388, 2504.2755, 2510.1608, 2513.1576, 2518.8602, 2519.2909, 2530.2932, 2535.2088, 2539.1622, 2551.1621, 2556.0916, 2558.1673, 2569.1725, 2572.1856, 2574.1490, 2575.3485, 2584.1815, 2590.1477, 2600.1769, 2968.4276, 3096.5257, 3540.7719

20. Q6A368_VIPAM **Mass:** 16489 **Score:** 19 **Expect:** 17 **Matches:** 3

Ammodytin L(3) variant OS=Vipera ammodytes montandoni OX=235554 PE=2 SV=1

Observed	Mr(expt)	Mr(calc)	ppm	Start	End	Miss	Ions	Peptide
2075.0641	2074.0568	2073.9037	73.8	32 -	49	0	---	K.NPITSYSFYGCHCGLGNK.G
2458.2475	2457.2402	2457.3818	-57.60	2 -	23	1	---	M.RILWIVAVCLIGVEGSVIEFGK.M
2485.1932	2484.1860	2484.1678	7.30	32 -	53	1	---	K.NPITSYSFYGCHCGLGNKGKPK.D

No match to: 866.5712, 888.4510, 1077.4785, 1172.6386, 1186.6312, 1189.6689, 1205.6624, 1208.6590, 1212.6257, 1215.6815, 1221.6559, 1230.6987, 1231.6710, 1232.1392, 1234.6802, 1237.6524, 1246.6827, 1304.6060, 1320.5943, 1330.6201, 1346.6114, 1372.7216, 1429.7399, 1450.7394, 1592.8032, 1632.6900, 1642.9239, 1649.7186, 1665.7056, 1731.9567, 1802.9521, 1852.0286, 1997.0124, 2022.0758, 2038.0531, 2053.0494, 2056.0667, 2066.0564, 2069.0742, 2080.0660, 2094.0792, 2106.0776, 2109.0960, 2111.0719, 2113.0883, 2118.0892, 2124.0832, 2127.0923, 2130.0855, 2134.0649, 2137.0790, 2139.1052, 2145.0792, 2155.1018, 2159.0676, 2161.0764, 2171.0968, 2187.0862, 2257.1391, 2265.0817, 2285.1075, 2300.1160, 2371.2527, 2386.2189, 2405.2424, 2418.2571, 2442.1046, 2443.2546, 2447.4393, 2455.2529, 2462.2618, 2473.2683, 2476.2783, 2488.2746, 2494.2292, 2501.1388, 2504.2755, 2510.1608, 2513.1576, 2518.8602, 2519.2909, 2530.2932, 2535.2088, 2539.1622, 2551.1621, 2556.0916, 2558.1673, 2569.1725, 2572.1856, 2574.1490, 2575.3485, 2584.1815, 2590.1477, 2600.1769, 2968.4276, 3096.5257, 3540.7719

Search Parameters

Type of search : MS/MS Ion Search
Enzyme : Trypsin
Fixed modifications : Carbamidomethyl (C)
Variable modifications : Oxidation (M)
Mass values : Monoisotopic
Protein Mass : Unrestricted
Peptide Mass Tolerance : ± 100 ppm
Fragment Mass Tolerance: ± 0.7 Da
Max Missed Cleavages : 1

```
Instrument type       : MALDI-TOF-TOF
Query1 (866.5712,1+)   : <no title>
Query2 (888.4510,1+)   : \\PROTEOMICS1\D\DATA\2105\210505_TLIEPOL_1_68252-68266\68252_68266_LPARDO\0_G10\1\888.4510.LIFT\1SREF\PDATA\1\1R
Query3 (1077.4785,1+)  : <no title>
Query4 (1172.6386,1+)  : <no title>
Query5 (1186.6312,1+)  : <no title>
Query6 (1189.6689,1+)  : \\PROTEOMICS1\D\DATA\2105\210505_TLIEPOL_1_68252-68266\68252_68266_LPARDO\0_G10\1\1189.6689.LIFT\1SREF\PDATA\1\1R
Query7 (1205.6624,1+)  : \\PROTEOMICS1\D\DATA\2105\210505_TLIEPOL_1_68252-68266\68252_68266_LPARDO\0_G10\1\1205.6624.LIFT\1SREF\PDATA\1\1R
Query8 (1208.6590,1+)  : <no title>
Query9 (1212.6257,1+)  : \\PROTEOMICS1\D\DATA\2105\210505_TLIEPOL_1_68252-68266\68252_68266_LPARDO\0_G10\1\1212.6257.LIFT\1SREF\PDATA\1\1R
Query10 (1215.6815,1+) : <no title>
Query11 (1221.6559,1+) : <no title>
Query12 (1230.6987,1+) : <no title>
Query13 (1231.6710,1+) : <no title>
Query14 (1232.1392,1+) : <no title>
Query15 (1234.6802,1+) : <no title>
Query16 (1237.6524,1+) : <no title>
Query17 (1246.6827,1+) : <no title>
Query18 (1304.6060,1+) : <no title>
Query19 (1320.5943,1+) : <no title>
Query20 (1330.6201,1+) : <no title>
Query21 (1346.6114,1+) : <no title>
Query22 (1372.7216,1+) : <no title>
Query23 (1429.7399,1+) : <no title>
Query24 (1450.7394,1+) : <no title>
Query25 (1592.8032,1+) : \\PROTEOMICS1\D\DATA\2105\210505_TLIEPOL_1_68252-68266\68252_68266_LPARDO\0_G10\1\1592.8032.LIFT\1SREF\PDATA\1\1R
Query26 (1632.6900,1+) : <no title>
Query27 (1642.9239,1+) : <no title>
Query28 (1649.7186,1+) : <no title>
Query29 (1665.7056,1+) : <no title>
Query30 (1731.9567,1+) : <no title>
Query31 (1802.9521,1+) : <no title>
Query32 (1852.0286,1+) : <no title>
Query33 (1997.0124,1+) : <no title>
Query34 (2022.0758,1+) : <no title>
Query35 (2038.0531,1+) : <no title>
Query36 (2053.0494,1+) : <no title>
Query37 (2056.0667,1+) : <no title>
Query38 (2066.0564,1+) : <no title>
Query39 (2069.0742,1+) : <no title>
Query40 (2075.0641,1+) : <no title>
Query41 (2080.0660,1+) : <no title>
Query42 (2094.0792,1+) : <no title>
Query43 (2106.0776,1+) : <no title>
Query44 (2109.0960,1+) : <no title>
Query45 (2111.0719,1+) : <no title>
Query46 (2113.0883,1+) : \\PROTEOMICS1\D\DATA\2105\210505_TLIEPOL_1_68252-68266\68252_68266_LPARDO\0_G10\1\2113.0883.LIFT\1SREF\PDATA\1\1R
Query47 (2118.0892,1+) : <no title>
Query48 (2124.0832,1+) : <no title>
Query49 (2127.0923,1+) : <no title>
Query50 (2130.0855,1+) : <no title>
Query51 (2134.0649,1+) : <no title>
Query52 (2137.0790,1+) : <no title>
Query53 (2139.1052,1+) : \\PROTEOMICS1\D\DATA\2105\210505_TLIEPOL_1_68252-68266\68252_68266_LPARDO\0_G10\1\2139.1052.LIFT\1SREF\PDATA\1\1R
Query54 (2145.0792,1+) : \\PROTEOMICS1\D\DATA\2105\210505_TLIEPOL_1_68252-68266\68252_68266_LPARDO\0_G10\1\2145.0792.LIFT\1SREF\PDATA\1\1R
Query55 (2155.1018,1+) : \\PROTEOMICS1\D\DATA\2105\210505_TLIEPOL_1_68252-68266\68252_68266_LPARDO\0_G10\1\2155.1018.LIFT\1SREF\PDATA\1\1R
Query56 (2159.0676,1+) : <no title>
Query57 (2161.0764,1+) : <no title>
Query58 (2171.0968,1+) : <no title>
Query59 (2187.0862,1+) : <no title>
Query60 (2257.1391,1+) : <no title>
Query61 (2265.0817,1+) : <no title>
Query62 (2285.1075,1+) : <no title>
Query63 (2300.1160,1+) : <no title>
Query64 (2371.2527,1+) : <no title>
Query65 (2386.2189,1+) : <no title>
Query66 (2405.2424,1+) : <no title>
Query67 (2418.2571,1+) : <no title>
Query68 (2442.1046,1+) : <no title>
Query69 (2443.2546,1+) : <no title>
Query70 (2447.4393,1+) : <no title>
Query71 (2455.2529,1+) : <no title>
Query72 (2458.2475,1+) : <no title>
Query73 (2462.2618,1+) : \\PROTEOMICS1\D\DATA\2105\210505_TLIEPOL_1_68252-68266\68252_68266_LPARDO\0_G10\1\2462.2618.LIFT\1SREF\PDATA\1\1R
Query74 (2473.2683,1+) : <no title>
Query75 (2476.2783,1+) : <no title>
Query76 (2485.1932,1+) : <no title>
Query77 (2488.2746,1+) : <no title>
Query78 (2494.2292,1+) : <no title>
Query79 (2501.1388,1+) : <no title>
Query80 (2504.2755,1+) : <no title>
Query81 (2510.1608,1+) : <no title>
Query82 (2513.1576,1+) : <no title>
Query83 (2518.8602,1+) : <no title>
Query84 (2519.2909,1+) : <no title>
Query85 (2530.2932,1+) : <no title>
Query86 (2535.2088,1+) : <no title>
Query87 (2539.1622,1+) : <no title>
Query88 (2551.1621,1+) : <no title>
Query89 (2556.0916,1+) : <no title>
Query90 (2558.1673,1+) : \\PROTEOMICS1\D\DATA\2105\210505_TLIEPOL_1_68252-68266\68252_68266_LPARDO\0_G10\1\2558.1673.LIFT\1SREF\PDATA\1\1R
Query91 (2569.1725,1+) : <no title>
Query92 (2572.1856,1+) : <no title>
Query93 (2574.1490,1+) : \\PROTEOMICS1\D\DATA\2105\210505_TLIEPOL_1_68252-68266\68252_68266_LPARDO\0_G10\1\2574.1490.LIFT\1SREF\PDATA\1\1R
Query94 (2575.3485,1+) : <no title>
Query95 (2584.1815,1+) : <no title>
Query96 (2590.1477,1+) : <no title>
Query97 (2600.1769,1+) : <no title>
Query98 (2968.4276,1+) : <no title>
Query99 (3096.5257,1+) : <no title>
Query100 (3540.7719,1+): <no title>
```

Mascot: http://www.matrixscience.com/

Mascot Search Results Fr6-3

```
User         :
Email        :
Search title : vipera_All_8IDplus4 file:\\Proteomics1\d\data\2105\210505_tliepol_1_68252-68266\68252_68266_lpardo\0_H10\1\1SRef\pdata\1\peaklist.xml
Database     : vipera  (1414 sequences; 347452 residues)
Timestamp    : 5 May 2021 at 09:36:54 GMT
Top Score    : 88 for Mixture 1, VSPF5_MACLB + VSP2_MACLB
```

Mascot Score Histogram

Protein score is -10*Log(P), where P is the probability that the observed match is a random event.
Protein scores greater than 44 are significant (p<0.05).

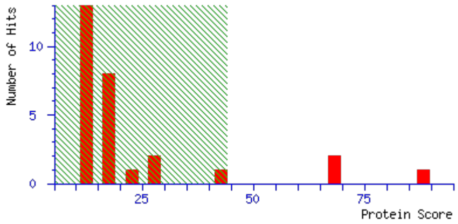

Concise Protein Summary Report

Format As | Concise Protein Summary | Help

Significance threshold p< 0.05 Max. number of hits 10

Re-Search All | Search Unmatched

1. **Mixture 1** **Total score:** 88 **Expect:** 2e-006 **Matches:** 20
   Components (only one family member shown for each component):
   VSPF5_MACLB **Mass:** 29261 **Score:** 68 **Expect:** 0.00025 **Matches:** 10
   Factor V activator OS=Macrovipera lebetina OX=8709 PE=1 SV=1
   VSP2_MACLB **Mass:** 29559 **Score:** 66 **Expect:** 0.00034 **Matches:** 11
   Venom serine proteinase-like protein 2 OS=Macrovipera lebetina OX=8709 PE=2 SV=1

2. VSPF5_MACLB **Mass:** 29261 **Score:** 68 **Expect:** 0.00025 **Matches:** 10
   Factor V activator OS=Macrovipera lebetina OX=8709 PE=1 SV=1
   SLMB_MACLB **Mass:** 15415 **Score:** 12 **Expect:** 80 **Matches:** 2
   Snaclec macrovipecetin subunit beta OS=Macrovipera lebetina OX=8709 PE=1 SV=1

3. VSP2_MACLB **Mass:** 29559 **Score:** 66 **Expect:** 0.00034 **Matches:** 11
   Venom serine proteinase-like protein 2 OS=Macrovipera lebetina OX=8709 PE=2 SV=1
   A0A6G5ZVX7_9SAUR **Mass:** 29593 **Score:** 44 **Expect:** 0.06 **Matches:** 8
   Serine protease 7 OS=Vipera anatolica senliki OX=2604287 PE=2 SV=1
   VSPH1_VIPAA **Mass:** 29593 **Score:** 44 **Expect:** 0.06 **Matches:** 8
   Vaa serine proteinase homolog 1 OS=Vipera ammodytes ammodytes OX=8705 PE=1 SV=1
   DID2A_MACLB **Mass:** 14638 **Score:** 15 **Expect:** 46 **Matches:** 2
   Disintegrin lebein-2-alpha OS=Macrovipera lebetina OX=8709 PE=1 SV=1
   A0A6B7FMQ3_VIPAA **Mass:** 28909 **Score:** 14 **Expect:** 55 **Matches:** 3
   Snake venom serine protease OS=Vipera ammodytes ammodytes OX=8705 PE=2 SV=1

4. A0A6G5ZUR6_9SAUR **Mass:** 29579 **Score:** 44 **Expect:** 0.06 **Matches:** 9
   Serine protease 6 OS=Vipera anatolica senliki OX=2604287 PE=2 SV=1
   A0A6G5ZUW7_9SAUR **Mass:** 22103 **Score:** 24 **Expect:** 5.3 **Matches:** 7
   Serine protease 10 (Fragment) OS=Vipera anatolica senliki OX=2604287 PE=2 SV=1

5. VASP1_VIPAA **Mass:** 22635 **Score:** 29 **Expect:** 1.7 **Matches:** 5
   Snake venom serine protease VaSP1 (Fragments) OS=Vipera ammodytes ammodytes OX=8705 PE=1 SV=1

6. A0A6G5ZVL0_9SAUR **Mass:** 21283 **Score:** 26 **Expect:** 3.2 **Matches:** 4
   Cholinesterase 14 (Fragment) OS=Vipera anatolica senliki OX=2604287 PE=2 SV=1
   A0A6G5ZVP1_9SAUR **Mass:** 29798 **Score:** 23 **Expect:** 7.8 **Matches:** 4
   Cholinesterase 8 (Fragment) OS=Vipera anatolica senliki OX=2604287 PE=2 SV=1

7. A0A0F6N0C3_9PERO **Mass:** 80354 **Score:** 20 **Expect:** 13 **Matches:** 8
   Echiitoxin a-subunit OS=Echiichthys vipera OX=94984 PE=2 SV=1

8. A0A6G5ZVU2_9SAUR **Mass:** 59723 **Score:** 20 **Expect:** 16 **Matches:** 7
   Carboxylic ester hydrolase OS=Vipera anatolica senliki OX=2604287 PE=2 SV=1
   A0A6G5ZU77_9SAUR **Mass:** 59771 **Score:** 20 **Expect:** 16 **Matches:** 7
   Carboxylic ester hydrolase OS=Vipera anatolica senliki OX=2604287 PE=2 SV=1

9. VM3VB_MACLB **Mass:** 71022 **Score:** 19 **Expect:** 17 **Matches:** 7
   Zinc metalloproteinase-disintegrin-like VLAIP-B OS=Macrovipera lebetina OX=8709 PE=1 SV=1

10. A0A6G5ZVY6_9SAUR **Mass:** 24441 **Score:** 18 **Expect:** 22 **Matches:** 3
    Veficolin 6 (Fragment) OS=Vipera anatolica senliki OX=2604287 PE=2 SV=1
    A0A6G5ZUK5_9SAUR **Mass:** 26961 **Score:** 17 **Expect:** 28 **Matches:** 3
    Veficolin 5 (Fragment) OS=Vipera anatolica senliki OX=2604287 PE=2 SV=1

Search Parameters

```
Type of search         : Peptide Mass Fingerprint
Enzyme                 : Trypsin
Fixed modifications    : Carbamidomethyl (C)
Variable modifications : Oxidation (M)
Mass values            : Monoisotopic
Protein Mass           : Unrestricted
Peptide Mass Tolerance : ± 100 ppm
```

```
Peptide Charge State   : 1+
Max Missed Cleavages   : 1
Number of queries      : 92
Selected for scoring   : 56
```

**Mascot:** http://www.matrixscience.com/

Mascot Search Results

```
User          :
Email         :
Search title  : vipera_all_Lift file:\\Proteomics1\d\data\2105\210505_tliepol_1_68252-68266\68252_68266_lpardo\0_H10\1\0_H10.xml
Database      : vipera  (1414 sequences; 347452 residues)
Timestamp     : 5 May 2021 at 10:03:36 GMT
Protein hits  : VSPF5_MACLB      Factor V activator OS=Macrovipera lebetina OX=8709 PE=1 SV=1
                VASP1_VIPAA      Snake venom serine protease VaSP1 (Fragments) OS=Vipera ammodytes ammodytes OX=8705 PE=1 SV=1
                VSP2_MACLB       Venom serine proteinase-like protein 2 OS=Macrovipera lebetina OX=8709 PE=2 SV=1
                A0A6G5ZVW9_9SAUR Reticulocalbin 2 OS=Vipera anatolica senliki OX=2604287 PE=2 SV=1
                A0A223PK38_DABRR Coagulation factor V OS=Daboia russelii OX=8707 PE=2 SV=1
                A0A1I9KNN4_VIPAA Serine proteinase SP-3 OS=Vipera ammodytes ammodytes OX=8705 PE=2 SV=1
```

Mascot Score Histogram

Ions score is -10*Log(P), where P is the probability that the observed match is a random event.
Individual ions scores > 11 indicate identity or extensive homology (p<0.05).
Protein scores are derived from ions scores as a non-probabilistic basis for ranking protein hits.

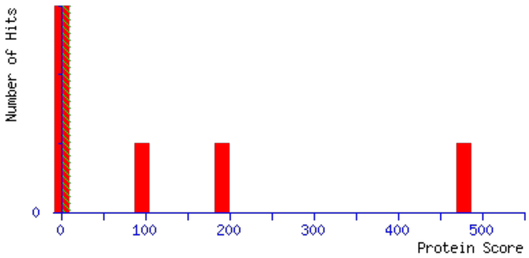

Peptide Summary Report

Format As: Peptide Summary — Help

Significance threshold p< 0.05 — Max. number of hits 10

Standard scoring ○ MudPIT scoring ◉ — Ions score or expect cut-off 0 — Show sub-sets 0

Show pop-ups ◉ Suppress pop-ups ○ — Sort unassigned Decreasing Score — Require bold red ☐

Overview Table

Click on column header to jump to entry in results list.
Move mouse over any indicator to highlight identical peptides.
Click on an indicator to see details of individual match.
Use check boxes to select sub-set of queries for new search.

Mouse over: -Query- -Accession- -Sequence-

Hit:	1	2	3	4	5	6
☑ 855.0780 (1+)						
☑ 871.0503 (1+)						
☑ 888.4499 (1+)						
☑ 903.4366 (1+)	●					●
☑ 909.4935 (1+)						
☑ 909.9951 (1+)						
☑ 919.5794 (1+)						
☑ 927.4661 (1+)						
☑ 929.4770 (1+)	●			●		
☑ 955.4912 (1+)						
☑ 961.5699 (1+)						
☑ 979.4703 (1+)						
☑ 979.9708 (1+)						
☑ 1022.1352 (1+)						
☑ 1044.1135 (1+)						
☑ 1057.5718 (1+)						
☑ 1066.0963 (1+)						
☑ 1066.4632 (1+)						
☑ 1077.4726 (1+)						
☑ 1117.6238 (1+)	●					
☑ 1189.6643 (1+)						
☑ 1205.6543 (1+)						
☑ 1208.6533 (1+)						

☑ 1212.6224 (1+)						
☑ 1224.6497 (1+)						
☑ 1227.6140 (1+)						
☑ 1230.6575 (1+)						
☑ 1232.1331 (1+)						
☑ 1232.6485 (1+)						
☑ 1237.6417 (1+)						
☑ 1249.1193 (1+)						
☑ 1256.6413 (1+)						
☑ 1304.6017 (1+)						
☑ 1408.8054 (1+)						
☑ 1429.7346 (1+)						
☑ 1477.8040 (1+)						
☑ 1493.1123 (1+)						
☑ 1521.7995 (1+)						
☑ 1531.8534 (1+)						
☑ 1547.8397 (1+)						
☑ 1569.7966 (1+)						
☑ 1585.7773 (1+)						
☑ 1592.7956 (1+)						
☑ 1649.7106 (1+)						
☑ 1685.8453 (1+)						
☑ 1687.9391 (1+)						
☑ 1703.9311 (1+)						
☑ 1776.9571 (1+)						
☑ 1802.9661 (1+)						
☑ 1817.9764 (1+)	●					
☑ 1829.8674 (1+)						
☑ 1839.9471 (1+)						
☑ 1843.9920 (1+)						
☑ 1847.8682 (1+)	●					
☑ 1855.9112 (1+)						
☑ 1859.9808 (1+)						
☑ 1869.8569 (1+)						
☑ 1885.8106 (1+)						
☑ 1900.9041 (1+)						
☑ 1913.9195 (1+)						
☑ 1938.9168 (1+)						
☑ 1942.9134 (1+)						
☑ 1954.9108 (1+)						
☑ 1957.9288 (1+)	●					
☑ 1961.9224 (1+)						
☑ 1968.9271 (1+)						
☑ 1971.9093 (1+)						
☑ 1973.9214 (1+)						
☑ 1983.9369 (1+)						
☑ 1986.8952 (1+)						
☑ 1989.9174 (1+)						
☑ 1994.8815 (1+)						
☑ 2000.9197 (1+)						
☑ 2005.9086 (1+)						
☑ 2021.8911 (1+)						
☑ 2056.0536 (1+)						
☑ 2094.0666 (1+)						
☑ 2109.8996 (1+)						
☑ 2113.0822 (1+)		●	●			
☑ 2128.0609 (1+)						
☑ 2145.0641 (1+)						
☑ 2224.1628 (1+)						
☑ 2243.0980 (1+)						
☑ 2443.2428 (1+)						
☑ 2462.2547 (1+)		●				
☑ 2473.2605 (1+)						
☑ 2558.1567 (1+)			●			
☑ 2574.1403 (1+)						
☑ 2730.4559 (1+)						
☑ 3191.4147 (1+)	●				●	
☑ 3345.7177 (1+)						

☑ 3348.6589 (1+)

Select All | Select None | Search Selected | ☐ Error tolerant | Archive Report

1. VSPF5_MACLB **Mass:** 29261 **Score:** 479 **Matches:** 7(7) **Sequences:** 7(7)
Factor V activator OS=Macrovipera lebetina OX=8709 PE=1 SV=1
☐ Check to include this hit in error tolerant search or archive report

Query	Observed	Mr(expt)	Mr(calc)	ppm	Miss	Score	Expect	Rank	Unique	Peptide
4	903.4366	902.4293	902.4093	22.1	0	14	0.037	1	U	R.NEDEQIR.V
9	929.4770	928.4697	928.4477	23.7	0	30	0.001	1	U	K.FFCLNTK.F
20	1117.6238	1116.6165	1116.5961	18.2	0	47	2.3e-005	1	U	R.TLCAGILQGGK.D
50	1817.9764	1816.9691	1816.9431	14.3	0	130	9.6e-014	1	U	K.NSAHIAPISLPSSPSSPR.S
54	1847.8682	1846.8609	1846.8407	10.9	0	70	1e-007	1	U	K.ISTTEETYPDVPHCAK.I
64	1957.9288	1956.9215	1956.8941	14.0	0	102	6.7e-011	1	U	K.HAWCEALYPWVPADSR.T
90	3191.4147	3190.4074	3190.3714	11.3	0	164	3.9e-017	1	U	K.DTCEGDSGGPLICNGQIQGIVSGGSDPCGQR.L

2. VASP1_VIPAA **Mass:** 22635 **Score:** 191 **Matches:** 2(2) **Sequences:** 2(2)
Snake venom serine protease VaSP1 (Fragments) OS=Vipera ammodytes ammodytes OX=8705 PE=1 SV=1
☐ Check to include this hit in error tolerant search or archive report

Query	Observed	Mr(expt)	Mr(calc)	ppm	Miss	Score	Expect	Rank	Unique	Peptide
79	2113.0822	2112.0749	2112.0462	13.6	0	76	2.7e-008	1		R.FYCAGTLINQEWVLTAAR.C
85	2462.2547	2461.2474	2461.2172	12.3	0	129	1.6e-013	1	U	-.VIGGDECNINEHPFLVALHTAR.X

3. VSP2_MACLB **Mass:** 29559 **Score:** 106 **Matches:** 2(2) **Sequences:** 2(2)
Venom serine proteinase-like protein 2 OS=Macrovipera lebetina OX=8709 PE=2 SV=1
☐ Check to include this hit in error tolerant search or archive report

Query	Observed	Mr(expt)	Mr(calc)	ppm	Miss	Score	Expect	Rank	Unique	Peptide
79	2113.0822	2112.0749	2112.0462	13.6	0	76	2.7e-008	1		R.FYCAGTLINQEWVLTAAR.C
87	2558.1567	2557.1495	2557.1189	12.0	0	43	4.6e-005	1	U	K.VTYPDVPHCANINMFDYSVCR.K

4. A0A6G5ZVW9_9SAUR **Mass:** 36042 **Score:** 0 **Matches:** 1(0) **Sequences:** 1(0)
Reticulocalbin 2 OS=Vipera anatolica senliki OX=2604287 PE=2 SV=1
☐ Check to include this hit in error tolerant search or archive report

Query	Observed	Mr(expt)	Mr(calc)	ppm	Miss	Score	Expect	Rank	Unique	Peptide
9	929.4770	928.4697	928.4290	43.8	1	9	0.12	2	U	R.FKNDYDK.D

5. A0A223PK38_DABRR **Mass:** 167642 **Score:** 0 **Matches:** 1(0) **Sequences:** 1(0)
Coagulation factor V OS=Daboia russelii OX=8707 PE=2 SV=1
☐ Check to include this hit in error tolerant search or archive report

Query	Observed	Mr(expt)	Mr(calc)	ppm	Miss	Score	Expect	Rank	Unique	Peptide
90	3191.4147	3190.4074	3190.6921	-89.22	1	2	0.6	2	U	K.DIHSGLIGPILICQKGTIDNYNRPIDIR.E

Proteins matching the same set of peptides:
A0A6G5ZUS6_9SAUR **Mass:** 13128 **Score:** 0 **Matches:** 1(0) **Sequences:** 1(0)
Coagulation factor V 5 (Fragment) OS=Vipera anatolica senliki OX=2604287 PE=2 SV=1

6. A0A1I9KNN4_VIPAA **Score:** 0 **Matches:** 1(0) **Sequences:** 1(0)
Serine proteinase SP-3 OS=Vipera ammodytes ammodytes OX=8705 PE=2 SV=1
☐ Check to include this hit in error tolerant search or archive report

Query	Observed	Mr(expt)	Mr(calc)	ppm	Miss	Score	Expect	Rank	Unique	Peptide
4	903.4366	902.4293	902.5008	-79.19	0	1	0.78	2	U	R.TLCAGILR.G

Proteins matching the same set of peptides:
A0A1I9KNR5_VIPAA **Score:** 0 **Matches:** 1(0) **Sequences:** 1(0)

Peptide matches not assigned to protein hits: (no details means no match)

	Query	Observed	Mr(expt)	Mr(calc)	ppm	Miss	Score	Expect	Rank	Unique	Peptide
☑	1	*855.0780*	*854.0707*								
☑	2	*871.0503*	*870.0430*								
☑	3	*888.4499*	*887.4427*								
☑	5	*909.4935*	*908.4863*								
☑	6	*909.9951*	*908.9879*								
☑	7	*919.5794*	*918.5721*								
☑	8	*927.4661*	*926.4588*								
☑	10	*955.4912*	*954.4840*								
☑	11	*961.5699*	*960.5626*								
☑	12	*979.4703*	*978.4630*								
☑	13	*979.9708*	*978.9635*								
☑	14	*1022.1352*	*1021.1279*								
☑	15	*1044.1135*	*1043.1062*								

☑	16	*1057.5718*	*1056.5646*
☑	17	*1066.0963*	*1065.0890*
☑	18	*1066.4632*	*1065.4559*
☑	19	*1077.4726*	*1076.4653*
☑	21	*1189.6643*	*1188.6570*
☑	22	*1205.6543*	*1204.6471*
☑	23	*1208.6533*	*1207.6460*
☑	24	*1212.6224*	*1211.6152*
☑	25	*1224.6497*	*1223.6424*
☑	26	*1227.6140*	*1226.6067*
☑	27	*1230.6575*	*1229.6502*
☑	28	*1232.1331*	*1231.1258*
☑	29	*1232.6485*	*1231.6412*
☑	30	*1237.6417*	*1236.6344*
☑	31	*1249.1193*	*1248.1120*
☑	32	*1256.6413*	*1255.6340*
☑	33	*1304.6017*	*1303.5944*
☑	34	*1408.8054*	*1407.7981*
☑	35	*1429.7346*	*1428.7273*
☑	36	*1477.8040*	*1476.7967*
☑	37	*1493.1123*	*1492.1050*
☑	38	*1521.7995*	*1520.7922*
☑	39	*1531.8534*	*1530.8461*
☑	40	*1547.8397*	*1546.8324*
☑	41	*1569.7966*	*1568.7893*
☑	42	*1585.7773*	*1584.7700*
☑	43	*1592.7956*	*1591.7883*
☑	44	*1649.7106*	*1648.7033*
☑	45	*1685.8453*	*1684.8381*
☑	46	*1687.9391*	*1686.9318*
☑	47	*1703.9311*	*1702.9238*
☑	48	*1776.9571*	*1775.9498*
☑	49	*1802.9661*	*1801.9588*
☑	51	*1829.8674*	*1828.8601*
☑	52	*1839.9471*	*1838.9398*
☑	53	*1843.9920*	*1842.9847*
☑	55	*1855.9112*	*1854.9039*
☑	56	*1859.9808*	*1858.9735*
☑	57	*1869.8569*	*1868.8497*
☑	58	*1885.8106*	*1884.8033*
☑	59	*1900.9041*	*1899.8969*
☑	60	*1913.9195*	*1912.9122*
☑	61	*1938.9168*	*1937.9095*
☑	62	*1942.9134*	*1941.9061*
☑	63	*1954.9108*	*1953.9036*
☑	65	*1961.9224*	*1960.9151*
☑	66	*1968.9271*	*1967.9198*
☑	67	*1971.9093*	*1970.9021*
☑	68	*1973.9214*	*1972.9142*
☑	69	*1983.9369*	*1982.9297*
☑	70	*1986.8952*	*1985.8879*
☑	71	*1989.9174*	*1988.9101*
☑	72	*1994.8815*	*1993.8742*
☑	73	*2000.9197*	*1999.9124*
☑	74	*2005.9086*	*2004.9013*
☑	75	*2021.8911*	*2020.8838*
☑	76	*2056.0536*	*2055.0464*
☑	77	*2094.0666*	*2093.0594*
☑	78	*2109.8996*	*2108.8923*
☑	80	*2128.0609*	*2127.0537*
☑	81	*2145.0641*	*2144.0568*
☑	82	*2224.1628*	*2223.1555*
☑	83	*2243.0980*	*2242.0907*
☑	84	*2443.2428*	*2442.2355*
☑	86	*2473.2605*	*2472.2533*
☑	88	*2574.1403*	*2573.1330*
☑	89	*2730.4559*	*2729.4486*
☑	91	*3345.7177*	*3344.7105*
☑	92	*3348.6589*	*3347.6516*

Search Parameters

```
Type of search         : MS/MS Ion Search
Enzyme                 : Trypsin
Fixed modifications    : Carbamidomethyl (C)
Variable modifications : Oxidation (M)
Mass values            : Monoisotopic
Protein Mass           : Unrestricted
Peptide Mass Tolerance : ± 100 ppm
Fragment Mass Tolerance: ± 0.7 Da
Max Missed Cleavages   : 1
Instrument type        : MALDI-TOF-TOF
```

```
Number of queries      : 92
```

**Mascot:** http://www.matrixscience.com/

Mascot Search Results

```
User          :
Email         :
Search title  : vipera_all_Lift file:\\Proteomics1\d\data\2105\210505_tliepol_1_68252-68266\68252_68266_lpardo\0_H10\1\0_H10.xml
Database      : vipera  (1414 sequences; 347452 residues)
Timestamp     : 5 May 2021 at 10:03:36 GMT
Warning       : A Peptide summary report will usually give a much clearer picture of MS/MS search results.
Top Score     : 625 for VSPF5_MACLB, Factor V activator OS=Macrovipera lebetina OX=8709 PE=1 SV=1
```

Mascot Score Histogram

Protein score is -10*Log(P), where P is the probability that the observed match is a random event.
Protein scores greater than 44 are significant (p<0.05).
Protein scores are derived from ions scores as a non-probabilistic basis for ranking protein hits.

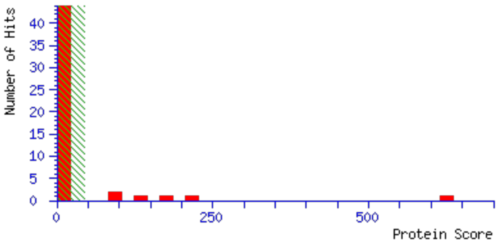

Protein Summary Report

Format As | Protein Summary (deprecated) | Help

Significance threshold p< 0.05 Max. number of hits 20

Overview Table

Click on column header to jump to entry in results list.
Move mouse over any indicator to highlight identical peptides.
Click on an indicator to see details of individual match.
Use check boxes to select sub-set of queries for new search.

Mouse over: -Query- -Accession- -Sequence-

Hit:	1	2	3	4	5	6	7	8	9	10	11	12	13	14	15	16	17	18	19	20
☑ 855.0780 (1+)																				
☑ 871.0503 (1+)																				
☑ 888.4499 (1+)																				
☑ 903.4366 (1+)	●																			
☑ 909.4935 (1+)																				
☑ 909.9951 (1+)																				
☑ 919.5794 (1+)																				
☑ 927.4661 (1+)																				
☑ 929.4770 (1+)	●																			
☑ 955.4912 (1+)																				
☑ 961.5699 (1+)																				
☑ 979.4703 (1+)																				
☑ 979.9708 (1+)																				
☑ 1022.1352 (1+)																				
☑ 1044.1135 (1+)																				
☑ 1057.5718 (1+)																				
☑ 1066.0963 (1+)																				
☑ 1066.4632 (1+)																				
☑ 1077.4726 (1+)																				
☑ 1117.6238 (1+)	●																			
☑ 1189.6643 (1+)																				
☑ 1205.6543 (1+)																				
☑ 1208.6533 (1+)																				
☑ 1212.6224 (1+)																				
☑ 1224.6497 (1+)																				
☑ 1227.6140 (1+)																				
☑ 1230.6575 (1+)																				
☑ 1232.1331 (1+)																				
☑ 1232.6485 (1+)																				
☑ 1237.6417 (1+)																				
☑ 1249.1193 (1+)																				
☑ 1256.6413 (1+)																				
☑ 1304.6017 (1+)																				
☑ 1408.8054 (1+)																				

☑ 1429.7346 (1+)																				
☑ 1477.8040 (1+)																				
☑ 1493.1123 (1+)																				
☑ 1521.7995 (1+)																				
☑ 1531.8534 (1+)																				
☑ 1547.8397 (1+)																				
☑ 1569.7966 (1+)																				
☑ 1585.7773 (1+)																				
☑ 1592.7956 (1+)																				
☑ 1649.7106 (1+)																				
☑ 1685.8453 (1+)																				
☑ 1687.9391 (1+)																				
☑ 1703.9311 (1+)																				
☑ 1776.9571 (1+)																				
☑ 1802.9661 (1+)																				
☑ 1817.9764 (1+)	●																			
☑ 1829.8674 (1+)																				
☑ 1839.9471 (1+)																				
☑ 1843.9920 (1+)																				
☑ 1847.8682 (1+)	●																			
☑ 1855.9112 (1+)																				
☑ 1859.9808 (1+)																				
☑ 1869.8569 (1+)																				
☑ 1885.8106 (1+)																				
☑ 1900.9041 (1+)																				
☑ 1913.9195 (1+)																				
☑ 1938.9168 (1+)																				
☑ 1942.9134 (1+)																				
☑ 1954.9108 (1+)																				
☑ 1957.9288 (1+)	●																			
☑ 1961.9224 (1+)																				
☑ 1968.9271 (1+)																				
☑ 1971.9093 (1+)																				
☑ 1973.9214 (1+)																				
☑ 1983.9369 (1+)																				
☑ 1986.8952 (1+)																				
☑ 1989.9174 (1+)																				
☑ 1994.8815 (1+)																				
☑ 2000.9197 (1+)																				
☑ 2005.9086 (1+)																				
☑ 2021.8911 (1+)																				
☑ 2056.0536 (1+)																				
☑ 2094.0666 (1+)																				
☑ 2109.8996 (1+)																				
☑ 2113.0822 (1+)		●	●	●	●	●														
☑ 2128.0609 (1+)																				
☑ 2145.0641 (1+)																				
☑ 2224.1628 (1+)																				
☑ 2243.0980 (1+)																				
☑ 2443.2428 (1+)																				
☑ 2462.2547 (1+)		●																		
☑ 2473.2605 (1+)																				
☑ 2558.1567 (1+)			●																	
☑ 2574.1403 (1+)																				
☑ 2730.4559 (1+)																				
☑ 3191.4147 (1+)	●							●				●								
☑ 3345.7177 (1+)																				
☑ 3348.6589 (1+)																				

Select All | Select None | Search Selected

Index

	Accession	Mass	Score	Description
1.	VSPF5_MACLB	29261	625	Factor V activator OS=Macrovipera lebetina OX=8709 PE=1 SV=1
2.	VASP1_VIPAA	22635	226	Snake venom serine protease VaSP1 (Fragments) OS=Vipera ammodytes ammodytes OX=8705 PE=1 SV=1
3.	VSP2_MACLB	29559	169	Venom serine proteinase-like protein 2 OS=Macrovipera lebetina OX=8709 PE=2 SV=1
4.	A0A6G5ZUR6_9SAUR	29579	115	Serine protease 6 OS=Vipera anatolica senliki OX=2604287 PE=2 SV=1
5.	A0A6G5ZVX7_9SAUR	29593	108	Serine protease 7 OS=Vipera anatolica senliki OX=2604287 PE=2 SV=1
6.	VSPH1_VIPAA	29593	108	Vaa serine proteinase homolog 1 OS=Vipera ammodytes ammodytes OX=8705 PE=1 SV=1
7.	A0A0H3Y6S3_9PERO	29853	24	Glycotransferase (Fragment) OS=Echiichthys vipera OX=94984 GN=glyt PE=3 SV=1
8.	A0A6G5ZUS6_9SAUR	13128	23	Coagulation factor V 5 (Fragment) OS=Vipera anatolica senliki OX=2604287 PE=2 SV=1
9.	A0A6G5ZUW7_9SAUR	22103	22	Serine protease 10 (Fragment) OS=Vipera anatolica senliki OX=2604287 PE=2 SV=1
10.	VM3VB_MACLB	71022	20	Zinc metalloproteinase-disintegrin-like VLAIP-B OS=Macrovipera lebetina OX=8709 PE=1 SV=1
11.	A0A6G5ZVL0_9SAUR	21283	19	Cholinesterase 14 (Fragment) OS=Vipera anatolica senliki OX=2604287 PE=2 SV=1
12.	A0A223PK38_DABRR	167642	18	Coagulation factor V OS=Daboia russelii OX=8707 PE=2 SV=1
13.	A0A0F6N0C3_9PERO	80354	18	Echiitoxin a-subunit OS=Echiichthys vipera OX=94984 PE=2 SV=1
14.	A0A223PK42_DABRR	63916	17	Group 4C secretory phospholipase A2 (Fragment) OS=Daboia russelii OX=8707 PE=2 SV=1

15. **A0A6G5ZVU2_9SAUR** 59723 17 Carboxylic ester hydrolase OS=Vipera anatolica senliki OX=2604287 PE=2 SV=1
16. **A0A6G5ZU77_9SAUR** 59771 17 Carboxylic ester hydrolase OS=Vipera anatolica senliki OX=2604287 PE=2 SV=1
17. **A0A223PK58_DABRR** 88544 16 Phospholipase A-2-activating protein (Fragment) OS=Daboia russelii OX=8707 PE=2 SV=1
18. **VSPA_MACLB** 29274 16 Alpha-fibrinogenase OS=Macrovipera lebetina OX=8709 PE=1 SV=1
19. **A0A6G5ZW74_9SAUR** 32808 16 Veficolin 9 (Fragment) OS=Vipera anatolica senliki OX=2604287 PE=2 SV=1
20. **Q8UVG8_DABRR** 52931 16 Vimentin OS=Daboia russelii OX=8707 PE=2 SV=1

Results List

1. VSPF5_MACLB **Mass:** 29261 **Score:** 625 **Expect:** 4.5e-060 **Matches:** 10

Factor V activator OS=Macrovipera lebetina OX=8709 PE=1 SV=1

Observed	Mr(expt)	Mr(calc)	ppm	Start	End	Miss	Ions	Peptide
903.4366	902.4293	902.4093	22.1	86 -	92	0	14	R.NEDEQIR.V
919.5794	918.5721	918.5538	19.9	231 -	238	0	---	R.LKPAVYTK.V
929.4770	928.4697	928.4477	23.7	98 -	104	0	30	K.FFCLNTK.F
1057.5718	1056.5646	1056.5426	20.8	97 -	104	1	---	R.KFFCLNTK.F
1117.6238	1116.6165	1116.5961	18.2	189 -	199	0	47	R.TLCAGILQGGK.D
1817.9764	1816.9691	1816.9431	14.3	124 -	141	0	130	K.NSAHIAPISLPSSPSSPR.S
1847.8682	1846.8609	1846.8407	10.9	152 -	167	0	70	K.ISTTEETYPDVPHCAK.I
1957.9288	1956.9215	1956.8941	14.0	173 -	188	0	102	K.HAWCEALYPWVPADSR.T
2558.1567	2557.1495	2557.2940	-56.52	168 -	188	1	---	K.IFIVKHAWCEALYPWVPADSR.T
3191.4147	3190.4074	3190.3714	11.3	200 -	230	0	164	K.DTCEGDSGGPLICNGQIQGIVSGGSDPCGQR.L

No match to: 855.0780, 871.0503, 888.4499, 909.4935, 909.9951, 927.4661, 955.4912, 961.5699, 979.4703, 979.9708, 1022.1352, 1044.1135, 1066.0963, 1066.4632, 1077.4726, 1189.6643, 1205.6543, 1208.6533, 1212.6224, 1224.6497, 1227.6140, 1230.6575, 1232.1331, 1232.6485, 1237.6417, 1249.1193, 1256.6413, 1304.6017, 1408.8054, 1429.7346, 1477.8040, 1493.1123, 1521.7995, 1531.8534, 1547.8397, 1569.7966, 1585.7773, 1592.7956, 1649.7106, 1685.8453, 1687.9391, 1703.9311, 1776.9571, 1802.9661, 1829.8674, 1839.9471, 1843.9920, 1855.9112, 1859.9808, 1869.8569, 1885.8106, 1900.9041, 1913.9195, 1938.9168, 1942.9134, 1954.9108, 1961.9224, 1968.9271, 1971.9093, 1973.9214, 1983.9369, 1986.8952, 1989.9174, 1994.8815, 2000.9197, 2005.9086, 2021.8911, 2056.0536, 2094.0666, 2109.8996, 2113.0822, 2128.0609, 2145.0641, 2224.1628, 2243.0980, 2443.2428, 2462.2547, 2473.2605, 2574.1403, 2730.4559, 3345.7177, 3348.6589

2. VASP1_VIPAA **Mass:** 22635 **Score:** 226 **Expect:** 3.6e-020 **Matches:** 5

Snake venom serine protease VaSP1 (Fragments) OS=Vipera ammodytes ammodytes OX=8705 PE=1 SV=1

Observed	Mr(expt)	Mr(calc)	ppm	Start	End	Miss	Ions	Peptide
1189.6643	1188.6570	1188.6325	20.6	85 -	93	1	---	R.WDKDIMLIR.L
1205.6543	1204.6471	1204.6274	16.3	85 -	93	1	---	R.WDKDIMLIR.L + Oxidation (M)
1592.7956	1591.7883	1591.7698	11.6	165 -	179	0	---	R.TLCAGILQGGIDSCK.G
2113.0822	2112.0749	2112.0462	13.6	26 -	43	0	76	R.FYCAGTLINQEWVLTAAR.C
2462.2547	2461.2474	2461.2172	12.3	1 -	22	0	129	-.VIGGDECNINEHPFLVALHTAR.X

No match to: 855.0780, 871.0503, 888.4499, 903.4366, 909.4935, 909.9951, 919.5794, 927.4661, 929.4770, 955.4912, 961.5699, 979.4703, 979.9708, 1022.1352, 1044.1135, 1057.5718, 1066.0963, 1066.4632, 1077.4726, 1117.6238, 1208.6533, 1212.6224, 1224.6497, 1227.6140, 1230.6575, 1232.1331, 1232.6485, 1237.6417, 1249.1193, 1256.6413, 1304.6017, 1408.8054, 1429.7346, 1477.8040, 1493.1123, 1521.7995, 1531.8534, 1547.8397, 1569.7966, 1585.7773, 1649.7106, 1685.8453, 1687.9391, 1703.9311, 1776.9571, 1802.9661, 1817.9764, 1829.8674, 1839.9471, 1843.9920, 1847.8682, 1855.9112, 1859.9808, 1869.8569, 1885.8106, 1900.9041, 1913.9195, 1938.9168, 1942.9134, 1954.9108, 1957.9288, 1961.9224, 1968.9271, 1971.9093, 1973.9214, 1983.9369, 1986.8952, 1989.9174, 1994.8815, 2000.9197, 2005.9086, 2021.8911, 2056.0536, 2094.0666, 2109.8996, 2128.0609, 2145.0641, 2224.1628, 2243.0980, 2443.2428, 2473.2605, 2558.1567, 2574.1403, 2730.4559, 3191.4147, 3345.7177, 3348.6589

3. VSP2_MACLB **Mass:** 29559 **Score:** 169 **Expect:** 1.8e-014 **Matches:** 11

Venom serine proteinase-like protein 2 OS=Macrovipera lebetina OX=8709 PE=2 SV=1

Observed	Mr(expt)	Mr(calc)	ppm	Start	End	Miss	Ions	Peptide
888.4499	887.4427	887.4211	24.3	98 -	104	0	---	K.FFCLSSK.T
1189.6643	1188.6570	1188.6325	20.6	109 -	117	1	---	R.WDKDIMLIR.L
1205.6543	1204.6471	1204.6274	16.3	109 -	117	1	---	R.WDKDIMLIR.L + Oxidation (M)
1208.6533	1207.6460	1207.6271	15.6	146 -	156	0	---	R.IMGWGTITTTK.V
1212.6224	1211.6152	1211.5894	21.2	83 -	92	0	---	K.NVPNEDQQIR.V
1224.6497	1223.6424	1223.6220	16.7	146 -	156	0	---	R.IMGWGTITTTK.V + Oxidation (M)
1592.7956	1591.7883	1591.7698	11.6	189 -	203	0	---	R.TLCAGILQGGIDSCK.V
2113.0822	2112.0749	2112.0462	13.6	50 -	67	0	76	R.FYCAGTLINQEWVLTAAR.C
2558.1567	2557.1495	2557.1189	12.0	157 -	177	0	43	K.VTYPDVPHCANINMFDYSVCR.K
2574.1403	2573.1330	2573.1138	7.48	157 -	177	0	---	K.VTYPDVPHCANINMFDYSVCR.K + Oxidation (M)
2730.4559	2729.4486	2729.4171	11.6	120 -	145	0	---	K.KPVNDSTHIVPLSLPSSPPSVGSVCR.I

No match to: 855.0780, 871.0503, 903.4366, 909.4935, 909.9951, 919.5794, 927.4661, 929.4770, 955.4912, 961.5699, 979.4703, 979.9708, 1022.1352, 1044.1135, 1057.5718, 1066.0963, 1066.4632, 1077.4726, 1117.6238, 1227.6140, 1230.6575, 1232.1331, 1232.6485, 1237.6417, 1249.1193, 1256.6413, 1304.6017, 1408.8054, 1429.7346, 1477.8040, 1493.1123, 1521.7995, 1531.8534, 1547.8397, 1569.7966, 1585.7773, 1649.7106, 1685.8453, 1687.9391, 1703.9311, 1776.9571, 1802.9661, 1817.9764, 1829.8674, 1839.9471, 1843.9920, 1847.8682, 1855.9112, 1859.9808, 1869.8569, 1885.8106, 1900.9041, 1913.9195, 1938.9168, 1942.9134, 1954.9108, 1957.9288, 1961.9224, 1968.9271, 1971.9093, 1973.9214, 1983.9369, 1986.8952, 1989.9174, 1994.8815, 2000.9197, 2005.9086, 2021.8911, 2056.0536, 2094.0666, 2109.8996, 2128.0609, 2145.0641, 2224.1628, 2243.0980, 2443.2428, 2462.2547, 2473.2605, 3191.4147, 3345.7177, 3348.6589

4. A0A6G5ZUR6_9SAUR **Mass:** 29579 **Score:** 115 **Expect:** 4.5e-009 **Matches:** 9

Serine protease 6 OS=Vipera anatolica senliki OX=2604287 PE=2 SV=1

Observed	Mr(expt)	Mr(calc)	ppm	Start	End	Miss	Ions	Peptide
888.4499	887.4427	887.4211	24.3	98 -	104	0	---	K.FFCLSSK.S
955.4912	954.4840	954.4559	29.4	105 -	111	1	---	K.SYTRWDK.D
1189.6643	1188.6570	1188.6325	20.6	109 -	117	1	---	R.WDKDIMLIR.L
1205.6543	1204.6471	1204.6274	16.3	109 -	117	1	---	R.WDKDIMLIR.L + Oxidation (M)
1208.6533	1207.6460	1207.6271	15.6	146 -	156	0	---	R.IMGWGTITTTK.V
1224.6497	1223.6424	1223.6220	16.7	146 -	156	0	---	R.IMGWGTITTTK.V + Oxidation (M)
1592.7956	1591.7883	1591.7698	11.6	189 -	203	0	---	R.TLCAGILQGGIDSCK.V
2113.0822	2112.0749	2112.0462	13.6	50 -	67	0	76	R.FYCAGTLINQEWVLTAAR.C
2730.4559	2729.4486	2729.3919	20.8	120 -	145	0	---	K.RPVNDSTHIAPLSLPSSPPSVGSVCR.I

No match to: 855.0780, 871.0503, 903.4366, 909.4935, 909.9951, 919.5794, 927.4661, 929.4770, 961.5699, 979.4703, 979.9708, 1022.1352, 1044.1135, 1057.5718, 1066.0963, 1066.4632, 1077.4726, 1117.6238, 1212.6224, 1227.6140, 1230.6575, 1232.1331, 1232.6485, 1237.6417, 1249.1193, 1256.6413, 1304.6017, 1408.8054, 1429.7346, 1477.8040, 1493.1123, 1521.7995, 1531.8534, 1547.8397, 1569.7966, 1585.7773, 1649.7106, 1685.8453, 1687.9391, 1703.9311, 1776.9571, 1802.9661, 1817.9764, 1829.8674, 1839.9471, 1843.9920, 1847.8682, 1855.9112, 1859.9808, 1869.8569, 1885.8106, 1900.9041, 1913.9195, 1938.9168, 1942.9134, 1954.9108, 1957.9288, 1961.9224, 1968.9271, 1971.9093, 1973.9214, 1983.9369, 1986.8952, 1989.9174, 1994.8815, 2000.9197, 2005.9086, 2021.8911, 2056.0536, 2094.0666, 2109.8996, 2128.0609, 2145.0641, 2224.1628, 2243.0980, 2443.2428, 2462.2547, 2473.2605, 2558.1567, 2574.1403, 3191.4147, 3345.7177, 3348.6589

5. A0A6G5ZVX7_9SAUR **Mass:** 29593 **Score:** 108 **Expect:** 2.2e-008 **Matches:** 8

Serine protease 7 OS=Vipera anatolica senliki OX=2604287 PE=2 SV=1

Observed	Mr(expt)	Mr(calc)	ppm	Start	End	Miss	Ions	Peptide
888.4499	887.4427	887.4211	24.3	98 -	104	0	---	K.FFCLSSK.T

1189.6643	1188.6570	1188.6325	20.6	109 - 117	1	---	R.WDKDIMLIR.L
1205.6543	1204.6471	1204.6274	16.3	109 - 117	1	---	R.WDKDIMLIR.L + Oxidation (M)
1208.6533	1207.6460	1207.6271	15.6	146 - 156	0	---	R.IMGWGTITTTK.V
1224.6497	1223.6424	1223.6220	16.7	146 - 156	0	---	R.IMGWGTITTTK.V + Oxidation (M)
1592.7956	1591.7883	1591.7698	11.6	189 - 203	0	---	R.TLCAGILQGGIDSCK.V
2113.0822	2112.0749	2112.0462	13.6	50 - 67	0	76	R.FYCAGTLINQEWVLTAAR.C
2730.4559	2729.4486	2729.3919	20.8	120 - 145	0	---	K.RPVNDSTHIAPLSLPSSPPSVGSVCR.I

No match to: 855.0780, 871.0503, 903.4366, 909.4935, 909.9951, 919.5794, 927.4661, 929.4770, 955.4912, 961.5699, 979.4703, 979.9708, 1022.1352, 1044.1135, 1057.5718, 1066.0963, 1066.4632, 1077.4726, 1117.6238, 1212.6224, 1227.6140, 1230.6575, 1232.1331, 1232.6485, 1237.6417, 1249.1193, 1256.6413, 1304.6017, 1408.8054, 1429.7346, 1477.8040, 1493.1123, 1521.7995, 1531.8534, 1547.8397, 1569.7966, 1585.7773, 1649.7106, 1685.8453, 1687.9391, 1703.9311, 1776.9571, 1802.9661, 1817.9764, 1829.8674, 1839.9471, 1843.9920, 1847.8682, 1855.9112, 1859.9808, 1869.8569, 1885.8106, 1900.9041, 1913.9195, 1938.9168, 1942.9134, 1954.9108, 1957.9288, 1961.9224, 1968.9271, 1971.9093, 1973.9214, 1983.9369, 1986.8952, 1989.9174, 1994.8815, 2000.9197, 2005.9086, 2021.8911, 2056.0536, 2094.0666, 2109.8996, 2128.0609, 2145.0641, 2224.1628, 2243.0980, 2443.2428, 2462.2547, 2473.2605, 2558.1567, 2574.1403, 3191.4147, 3345.7177, 3348.6589

6. VSPH1_VIPAA **Mass:** 29593 **Score:** 108 **Expect:** 2.2e-008 **Matches:** 8

Vaa serine proteinase homolog 1 OS=Vipera ammodytes ammodytes OX=8705 PE=1 SV=1

Observed	Mr(expt)	Mr(calc)	ppm	Start - End	Miss	Ions	Peptide
888.4499	887.4427	887.4211	24.3	98 - 104	0	---	K.FFCLSSK.T
1189.6643	1188.6570	1188.6325	20.6	109 - 117	1	---	R.WDKDIMLIR.L
1205.6543	1204.6471	1204.6274	16.3	109 - 117	1	---	R.WDKDIMLIR.L + Oxidation (M)
1208.6533	1207.6460	1207.6271	15.6	146 - 156	0	---	R.IMGWGTITTTK.V
1224.6497	1223.6424	1223.6220	16.7	146 - 156	0	---	R.IMGWGTITTTK.V + Oxidation (M)
1592.7956	1591.7883	1591.7698	11.6	189 - 203	0	---	R.TLCAGILQGGIDSCK.V
2113.0822	2112.0749	2112.0462	13.6	50 - 67	0	76	R.FYCAGTLINQEWVLTAAR.C
2730.4559	2729.4486	2729.3919	20.8	120 - 145	0	---	K.RPVNDSTHIAPLSLPSSPPSVGSVCR.I

No match to: 855.0780, 871.0503, 903.4366, 909.4935, 909.9951, 919.5794, 927.4661, 929.4770, 955.4912, 961.5699, 979.4703, 979.9708, 1022.1352, 1044.1135, 1057.5718, 1066.0963, 1066.4632, 1077.4726, 1117.6238, 1212.6224, 1227.6140, 1230.6575, 1232.1331, 1232.6485, 1237.6417, 1249.1193, 1256.6413, 1304.6017, 1408.8054, 1429.7346, 1477.8040, 1493.1123, 1521.7995, 1531.8534, 1547.8397, 1569.7966, 1585.7773, 1649.7106, 1685.8453, 1687.9391, 1703.9311, 1776.9571, 1802.9661, 1817.9764, 1829.8674, 1839.9471, 1843.9920, 1847.8682, 1855.9112, 1859.9808, 1869.8569, 1885.8106, 1900.9041, 1913.9195, 1938.9168, 1942.9134, 1954.9108, 1957.9288, 1961.9224, 1968.9271, 1971.9093, 1973.9214, 1983.9369, 1986.8952, 1989.9174, 1994.8815, 2000.9197, 2005.9086, 2021.8911, 2056.0536, 2094.0666, 2109.8996, 2128.0609, 2145.0641, 2224.1628, 2243.0980, 2443.2428, 2462.2547, 2473.2605, 2558.1567, 2574.1403, 3191.4147, 3345.7177, 3348.6589

7. A0A0H3Y6S3_9PERO **Mass:** 29853 **Score:** 24 **Expect:** 5.8 **Matches:** 6

Glycotransferase (Fragment) OS=Echiichthys vipera OX=94984 GN=glyt PE=3 SV=1

Observed	Mr(expt)	Mr(calc)	ppm	Start - End	Miss	Ions	Peptide
919.5794	918.5721	918.5035	74.6	1 - 7	1	---	-.EIRQFAR.A
961.5699	960.5626	960.4817	84.2	189 - 195	0	---	R.NPEWLFR.I
1212.6224	1211.6152	1211.5652	41.2	180 - 188	1	---	K.DVPRHLCCR.N
1230.6575	1229.6502	1229.6615	-9.20	170 - 179	1	---	K.EEQERILVSK.D
1687.9391	1686.9318	1686.7871	85.8	184 - 195	1	---	R.HLCCRNPEWLFR.I
1859.9808	1858.9735	1858.9400	18.0	129 - 144	0	---	K.TLATLPGMDLHYVSWR.N

No match to: 855.0780, 871.0503, 888.4499, 903.4366, 909.4935, 909.9951, 927.4661, 929.4770, 955.4912, 979.4703, 979.9708, 1022.1352, 1044.1135, 1057.5718, 1066.0963, 1066.4632, 1077.4726, 1117.6238, 1189.6643, 1205.6543, 1208.6533, 1224.6497, 1227.6140, 1232.1331, 1232.6485, 1237.6417, 1249.1193, 1256.6413, 1304.6017, 1408.8054, 1429.7346, 1477.8040, 1493.1123, 1521.7995, 1531.8534, 1547.8397, 1569.7966, 1585.7773, 1592.7956, 1649.7106, 1685.8453, 1703.9311, 1776.9571, 1802.9661, 1817.9764, 1829.8674, 1839.9471, 1843.9920, 1847.8682, 1855.9112, 1869.8569, 1885.8106, 1900.9041, 1913.9195, 1938.9168, 1942.9134, 1954.9108, 1957.9288, 1961.9224, 1968.9271, 1971.9093, 1973.9214, 1983.9369, 1986.8952, 1989.9174, 1994.8815, 2000.9197, 2005.9086, 2021.8911, 2056.0536, 2094.0666, 2109.8996, 2113.0822, 2128.0609, 2145.0641, 2224.1628, 2243.0980, 2443.2428, 2462.2547, 2473.2605, 2558.1567, 2574.1403, 2730.4559, 3191.4147, 3345.7177, 3348.6589

8. A0A6G5ZUS6_9SAUR **Mass:** 13128 **Score:** 23 **Expect:** 6.5 **Matches:** 3

Coagulation factor V 5 (Fragment) OS=Vipera anatolica senliki OX=2604287 PE=2 SV=1

Observed	Mr(expt)	Mr(calc)	ppm	Start - End	Miss	Ions	Peptide
1829.8674	1828.8601	1828.8389	11.6	97 - 110	1	---	K.MYKDENVHWHLLNM.-
1957.9288	1956.9215	1956.9152	3.24	1 - 16	1	---	-.QICRSWAYYSGVNPEK.D
3191.4147	3190.4074	3190.6921	-89.22	17 - 44	1	2	K.DIHSGLIGPILICQKGTIDNYNRPIDIR.E

No match to: 855.0780, 871.0503, 888.4499, 903.4366, 909.4935, 909.9951, 919.5794, 927.4661, 929.4770, 955.4912, 961.5699, 979.4703, 979.9708, 1022.1352, 1044.1135, 1057.5718, 1066.0963, 1066.4632, 1077.4726, 1117.6238, 1189.6643, 1205.6543, 1208.6533, 1212.6224, 1224.6497, 1227.6140, 1230.6575, 1232.1331, 1232.6485, 1237.6417, 1249.1193, 1256.6413, 1304.6017, 1408.8054, 1429.7346, 1477.8040, 1493.1123, 1521.7995, 1531.8534, 1547.8397, 1569.7966, 1585.7773, 1592.7956, 1649.7106, 1685.8453, 1687.9391, 1703.9311, 1776.9571, 1802.9661, 1817.9764, 1839.9471, 1843.9920, 1847.8682, 1855.9112, 1859.9808, 1869.8569, 1885.8106, 1900.9041, 1913.9195, 1938.9168, 1942.9134, 1954.9108, 1961.9224, 1968.9271, 1971.9093, 1973.9214, 1983.9369, 1986.8952, 1989.9174, 1994.8815, 2000.9197, 2005.9086, 2021.8911, 2056.0536, 2094.0666, 2109.8996, 2113.0822, 2128.0609, 2145.0641, 2224.1628, 2243.0980, 2443.2428, 2462.2547, 2473.2605, 2558.1567, 2574.1403, 2730.4559, 3345.7177, 3348.6589

9. A0A6G5ZUW7_9SAUR **Mass:** 22103 **Score:** 22 **Expect:** 8.3 **Matches:** 7

Serine protease 10 (Fragment) OS=Vipera anatolica senliki OX=2604287 PE=2 SV=1

Observed	Mr(expt)	Mr(calc)	ppm	Start - End	Miss	Ions	Peptide
888.4499	887.4427	887.4211	24.3	95 - 101	0	---	K.FFCLSSK.S
955.4912	954.4840	954.4559	29.4	102 - 108	1	---	K.SYTRWDK.D
1189.6643	1188.6570	1188.6325	20.6	106 - 114	1	---	R.WDKDIMLIR.L
1205.6543	1204.6471	1204.6274	16.3	106 - 114	1	---	R.WDKDIMLIR.L + Oxidation (M)
1208.6533	1207.6460	1207.6271	15.6	143 - 153	0	---	R.IMGWGTITTTK.V
1224.6497	1223.6424	1223.6220	16.7	143 - 153	0	---	R.IMGWGTITTTK.V + Oxidation (M)
2730.4559	2729.4486	2729.3919	20.8	117 - 142	0	---	K.RPVNDSTHIAPLSLPSSPPSVGSVCR.I

No match to: 855.0780, 871.0503, 903.4366, 909.4935, 909.9951, 919.5794, 927.4661, 929.4770, 961.5699, 979.4703, 979.9708, 1022.1352, 1044.1135, 1057.5718, 1066.0963, 1066.4632, 1077.4726, 1117.6238, 1212.6224, 1227.6140, 1230.6575, 1232.1331, 1232.6485, 1237.6417, 1249.1193, 1256.6413, 1304.6017, 1408.8054, 1429.7346, 1477.8040, 1493.1123, 1521.7995, 1531.8534, 1547.8397, 1569.7966, 1585.7773, 1592.7956, 1649.7106, 1685.8453, 1687.9391, 1703.9311, 1776.9571, 1802.9661, 1817.9764, 1829.8674, 1839.9471, 1843.9920, 1847.8682, 1855.9112, 1859.9808, 1869.8569, 1885.8106, 1900.9041, 1913.9195, 1938.9168, 1942.9134, 1954.9108, 1957.9288, 1961.9224, 1968.9271, 1971.9093, 1973.9214, 1983.9369, 1986.8952, 1989.9174, 1994.8815, 2000.9197, 2005.9086, 2021.8911, 2056.0536, 2094.0666, 2109.8996, 2113.0822, 2128.0609, 2145.0641, 2224.1628, 2243.0980, 2443.2428, 2462.2547, 2473.2605, 2558.1567, 2574.1403, 3191.4147, 3345.7177, 3348.6589

10. VM3VB_MACLB **Mass:** 71022 **Score:** 20 **Expect:** 14 **Matches:** 7

Zinc metalloproteinase-disintegrin-like VLAIP-B OS=Macrovipera lebetina OX=8709 PE=1 SV=1

Observed	Mr(expt)	Mr(calc)	ppm	Start - End	Miss	Ions	Peptide
1232.6485	1231.6412	1231.6197	17.5	317 - 327	0	---	K.YSVGIVQDHSK.I
1256.6413	1255.6340	1255.6520	-14.36	185 - 195	0	---	K.ASQLNLTPEQR.T
1477.8040	1476.7967	1476.7646	21.7	134 - 145	0	---	K.LQGEMYLIEPLR.I + Oxidation (M)
1592.7956	1591.7883	1591.6469	88.8	501 - 513	0	---	K.GYCYNGTCPIMEK.Q

1957.9288	1956.9215	1956.8890	16.7	351 - 367	1	---	R.IDCTCGAKSCIMSGILR.C + Oxidation (M)
1973.9214	1972.9142	1972.8439	35.6	78 - 94	0	---	K.GLFSEDYSETHYTPDGR.E
2558.1567	2557.1495	2557.1069	16.6	359 - 379	1	---	K.SCIMSGILRCETSYLFSDCSR.E + Oxidation (M)

No match to: 855.0780, 871.0503, 888.4499, 903.4366, 909.4935, 909.9951, 919.5794, 927.4661, 929.4770, 955.4912, 961.5699, 979.4703, 979.9708, 1022.1352, 1044.1135, 1057.5718, 1066.0963, 1066.4632, 1077.4726, 1117.6238, 1189.6643, 1205.6543, 1208.6533, 1212.6224, 1224.6497, 1227.6140, 1230.6575, 1232.1331, 1237.6417, 1249.1193, 1304.6017, 1408.8054, 1429.7346, 1493.1123, 1521.7995, 1531.8534, 1547.8397, 1569.7966, 1585.7773, 1649.7106, 1685.8453, 1687.9391, 1703.9311, 1776.9571, 1802.9661, 1817.9764, 1829.8674, 1839.9471, 1843.9920, 1847.8682, 1855.9112, 1859.9808, 1869.8569, 1885.8106, 1900.9041, 1913.9195, 1938.9168, 1942.9134, 1954.9108, 1961.9224, 1968.9271, 1971.9093, 1983.9369, 1986.8952, 1989.9174, 1994.8815, 2000.9197, 2005.9086, 2021.8911, 2056.0536, 2094.0666, 2109.8996, 2113.0822, 2128.0609, 2145.0641, 2224.1628, 2243.0980, 2443.2428, 2462.2547, 2473.2605, 2574.1403, 2730.4559, 3191.4147, 3345.7177, 3348.6589

11. A0A6G5ZVL0_9SAUR **Mass:** 21283 **Score:** 19 **Expect:** 18 **Matches:** 4
Cholinesterase 14 (Fragment) OS=Vipera anatolica senliki OX=2604287 PE=2 SV=1

Observed	Mr(expt)	Mr(calc)	ppm	Start - End	Miss	Ions	Peptide
1205.6543	1204.6471	1204.5935	44.5	125 - 135	0	---	R.SITEAEEALSR.Q
1208.6533	1207.6460	1207.5370	90.2	46 - 55	0	---	K.FSEANHGQYR.L
1685.8453	1684.8381	1684.7840	32.1	64 - 77	0	---	R.DYFMVCPLAELAEK.I
1954.9108	1953.9036	1953.9692	-33.60	64 - 79	1	---	R.DYFMVCPLAELAEKIR.K

No match to: 855.0780, 871.0503, 888.4499, 903.4366, 909.4935, 909.9951, 919.5794, 927.4661, 929.4770, 955.4912, 961.5699, 979.4703, 979.9708, 1022.1352, 1044.1135, 1057.5718, 1066.0963, 1066.4632, 1077.4726, 1117.6238, 1189.6643, 1212.6224, 1224.6497, 1227.6140, 1230.6575, 1232.1331, 1232.6485, 1237.6417, 1249.1193, 1256.6413, 1304.6017, 1408.8054, 1429.7346, 1477.8040, 1493.1123, 1521.7995, 1531.8534, 1547.8397, 1569.7966, 1585.7773, 1592.7956, 1649.7106, 1687.9391, 1703.9311, 1776.9571, 1802.9661, 1817.9764, 1829.8674, 1839.9471, 1843.9920, 1847.8682, 1855.9112, 1859.9808, 1869.8569, 1885.8106, 1900.9041, 1913.9195, 1938.9168, 1942.9134, 1957.9288, 1961.9224, 1968.9271, 1971.9093, 1973.9214, 1983.9369, 1986.8952, 1989.9174, 1994.8815, 2000.9197, 2005.9086, 2021.8911, 2056.0536, 2094.0666, 2109.8996, 2113.0822, 2128.0609, 2145.0641, 2224.1628, 2243.0980, 2443.2428, 2462.2547, 2473.2605, 2558.1567, 2574.1403, 2730.4559, 3191.4147, 3345.7177, 3348.6589

12. A0A223PK38_DABRR **Mass:** 167642 **Score:** 18 **Expect:** 23 **Matches:** 9
Coagulation factor V OS=Daboia russelii OX=8707 PE=2 SV=1

Observed	Mr(expt)	Mr(calc)	ppm	Start - End	Miss	Ions	Peptide
903.4366	902.4293	902.4862	-63.01	4 - 11	0	---	R.YSVNPVPK.C
1208.6533	1207.6460	1207.5656	66.6	507 - 516	0	---	K.LYHSAVDMTR.D + Oxidation (M)
1429.7346	1428.7273	1428.6191	75.7	1252 - 1264	0	---	K.TQMVFEGNSDGTK.V + Oxidation (M)
1649.7106	1648.7033	1648.8169	-68.86	198 - 212	1	---	K.EGSLNADGTQKLFNR.E
1829.8674	1828.8601	1828.9108	-27.70	860 - 874	1	---	K.AIFRSYLDDTFQTPR.T
1900.9041	1899.8969	1900.0206	-65.14	1328 - 1343	1	---	K.TWLSSWEPSLARLNLK.G
2000.9197	1999.9124	2000.0116	-49.59	107 - 124	0	---	K.NFATQPVSIHPQSAVYNK.W
3191.4147	3190.4074	3190.6921	-89.22	981 - 1008	1	2	K.DIHSGLIGPILICQKGTIDNYNRPIDIR.E
3348.6589	3347.6516	3347.7554	-31.00	95 - 124	1	---	R.GEVGDILIIYFKNFATQPVSIHPQSAVYNK.W

No match to: 855.0780, 871.0503, 888.4499, 909.4935, 909.9951, 919.5794, 927.4661, 929.4770, 955.4912, 961.5699, 979.4703, 979.9708, 1022.1352, 1044.1135, 1057.5718, 1066.0963, 1066.4632, 1077.4726, 1117.6238, 1189.6643, 1205.6543, 1212.6224, 1224.6497, 1227.6140, 1230.6575, 1232.1331, 1232.6485, 1237.6417, 1249.1193, 1256.6413, 1304.6017, 1408.8054, 1477.8040, 1493.1123, 1521.7995, 1531.8534, 1547.8397, 1569.7966, 1585.7773, 1592.7956, 1685.8453, 1687.9391, 1703.9311, 1776.9571, 1802.9661, 1817.9764, 1839.9471, 1843.9920, 1847.8682, 1855.9112, 1859.9808, 1869.8569, 1885.8106, 1913.9195, 1938.9168, 1942.9134, 1954.9108, 1957.9288, 1961.9224, 1968.9271, 1971.9093, 1973.9214, 1983.9369, 1986.8952, 1989.9174, 1994.8815, 2005.9086, 2021.8911, 2056.0536, 2094.0666, 2109.8996, 2113.0822, 2128.0609, 2145.0641, 2224.1628, 2243.0980, 2443.2428, 2462.2547, 2473.2605, 2558.1567, 2574.1403, 2730.4559, 3345.7177

13. A0A0F6N0C3_9PERO **Mass:** 80354 **Score:** 18 **Expect:** 25 **Matches:** 8
Echiitoxin a-subunit OS=Echiichthys vipera OX=94984 PE=2 SV=1

Observed	Mr(expt)	Mr(calc)	ppm	Start - End	Miss	Ions	Peptide
929.4770	928.4697	928.5454	-81.54	263 - 270	1	---	R.EITVGRVR.N
955.4912	954.4840	954.4018	86.1	444 - 450	0	---	K.AWCFDTR.V
1900.9041	1899.8969	1899.8785	9.67	498 - 513	0	---	K.EGSLMTQDYTFSRPPR.V + Oxidation (M)
1983.9369	1982.9297	1982.9475	-9.01	373 - 389	1	---	K.ICVDMMEGTQIKVVSNK.T + 2 Oxidation (M)
2005.9086	2004.9013	2004.9673	-32.95	159 - 176	1	---	K.SEDSNLQDIEGKMEAVIK.S
2021.8911	2020.8838	2020.9623	-38.80	159 - 176	1	---	K.SEDSNLQDIEGKMEAVIK.K + Oxidation (M)
2113.0822	2112.0749	2112.1368	-29.29	305 - 321	1	---	R.VSNFQQIFRDYILTIQK.K
2145.0641	2144.0568	2144.1279	-33.16	297 - 313	1	---	K.QFPLLHDRVSNFQQIFR.D

No match to: 855.0780, 871.0503, 888.4499, 903.4366, 909.4935, 909.9951, 919.5794, 927.4661, 961.5699, 979.4703, 979.9708, 1022.1352, 1044.1135, 1057.5718, 1066.0963, 1066.4632, 1077.4726, 1117.6238, 1189.6643, 1205.6543, 1208.6533, 1212.6224, 1224.6497, 1227.6140, 1230.6575, 1232.1331, 1232.6485, 1237.6417, 1249.1193, 1256.6413, 1304.6017, 1408.8054, 1429.7346, 1477.8040, 1493.1123, 1521.7995, 1531.8534, 1547.8397, 1569.7966, 1585.7773, 1592.7956, 1649.7106, 1685.8453, 1687.9391, 1703.9311, 1776.9571, 1802.9661, 1817.9764, 1829.8674, 1839.9471, 1843.9920, 1847.8682, 1855.9112, 1859.9808, 1869.8569, 1885.8106, 1913.9195, 1938.9168, 1942.9134, 1954.9108, 1957.9288, 1961.9224, 1968.9271, 1971.9093, 1973.9214, 1986.8952, 1989.9174, 1994.8815, 2000.9197, 2056.0536, 2094.0666, 2109.8996, 2128.0609, 2224.1628, 2243.0980, 2443.2428, 2462.2547, 2473.2605, 2558.1567, 2574.1403, 2730.4559, 3191.4147, 3345.7177, 3348.6589

14. A0A223PK42_DABRR **Mass:** 63916 **Score:** 17 **Expect:** 29 **Matches:** 6
Group 4C secretory phospholipase A2 (Fragment) OS=Daboia russelii OX=8707 PE=2 SV=1

Observed	Mr(expt)	Mr(calc)	ppm	Start - End	Miss	Ions	Peptide
961.5699	960.5626	960.5128	51.9	273 - 280	1	---	K.DLETDIKK.L
1227.6140	1226.6067	1226.5779	23.5	263 - 272	1	---	K.YERSEDLTSK.D
1703.9311	1702.9238	1702.9101	8.06	7 - 22	1	---	R.ISKSLSDGEEVAITVR.K
1829.8674	1828.8601	1828.8374	12.5	154 - 170	1	---	K.VGEKQLSEHAGSCENGK.N
2021.8911	2020.8838	2020.9629	-39.13	510 - 526	1	---	R.EQFSTFKLSYSAEEIDK.L
2243.0980	2242.0907	2242.0299	27.1	108 - 125	1	---	K.CQFENLVQGQWDFNKAMK.A

No match to: 855.0780, 871.0503, 888.4499, 903.4366, 909.4935, 909.9951, 919.5794, 927.4661, 929.4770, 955.4912, 979.4703, 979.9708, 1022.1352, 1044.1135, 1057.5718, 1066.0963, 1066.4632, 1077.4726, 1117.6238, 1189.6643, 1205.6543, 1208.6533, 1212.6224, 1224.6497, 1230.6575, 1232.1331, 1232.6485, 1237.6417, 1249.1193, 1256.6413, 1304.6017, 1408.8054, 1429.7346, 1477.8040, 1493.1123, 1521.7995, 1531.8534, 1547.8397, 1569.7966, 1585.7773, 1592.7956, 1649.7106, 1685.8453, 1687.9391, 1776.9571, 1802.9661, 1817.9764, 1839.9471, 1843.9920, 1847.8682, 1855.9112, 1859.9808, 1869.8569, 1885.8106, 1900.9041, 1913.9195, 1938.9168, 1942.9134, 1954.9108, 1957.9288, 1961.9224, 1968.9271, 1971.9093, 1973.9214, 1983.9369, 1986.8952, 1989.9174, 1994.8815, 2000.9197, 2005.9086, 2056.0536, 2094.0666, 2109.8996, 2113.0822, 2128.0609, 2145.0641, 2224.1628, 2443.2428, 2462.2547, 2473.2605, 2558.1567, 2574.1403, 2730.4559, 3191.4147, 3345.7177, 3348.6589

15. A0A6G5ZVU2_9SAUR **Mass:** 59723 **Score:** 17 **Expect:** 30 **Matches:** 7
Carboxylic ester hydrolase OS=Vipera anatolica senliki OX=2604287 PE=2 SV=1

Observed	Mr(expt)	Mr(calc)	ppm	Start - End	Miss	Ions	Peptide
1205.6543	1204.6471	1204.5935	44.5	462 - 472	0	---	R.SITEAEEALSR.Q
1208.6533	1207.6460	1207.5370	90.2	383 - 392	0	---	K.FSEANHGQYR.L
1569.7966	1568.7893	1568.7981	-5.57	254 - 267	1	---	K.TPREAHTDAIMLSK.E
1585.7773	1584.7700	1584.7930	-14.49	254 - 267	1	---	K.TPREAHTDAIMLSK.E + Oxidation (M)
1685.8453	1684.8381	1684.7840	32.1	401 - 414	0	---	R.DYFMVCPLAELAEK.I

1859.9808	1858.9735	1858.8917	44.0	257	273	1	---	R.EAHTDAIMLSKEVGCAK.D
1954.9108	1953.9036	1953.9692	-33.60	401	416	1	---	R.DYFMVCPLAELAEKIR.K

No match to: 855.0780, 871.0503, 888.4499, 903.4366, 909.4935, 909.9951, 919.5794, 927.4661, 929.4770, 955.4912, 961.5699, 979.4703, 979.9708, 1022.1352, 1044.1135, 1057.5718, 1066.0963, 1066.4632, 1077.4726, 1117.6238, 1189.6643, 1212.6224, 1224.6497, 1227.6140, 1230.6575, 1232.1331, 1232.6485, 1237.6417, 1249.1193, 1256.6413, 1304.6017, 1408.8054, 1429.7346, 1477.8040, 1493.1123, 1521.7995, 1531.8534, 1547.8397, 1592.7956, 1649.7106, 1687.9391, 1703.9311, 1776.9571, 1802.9661, 1817.9764, 1829.8674, 1839.9471, 1843.9920, 1847.8682, 1855.9112, 1869.8569, 1885.8106, 1900.9041, 1913.9195, 1938.9168, 1942.9134, 1957.9288, 1961.9224, 1968.9271, 1971.9093, 1973.9214, 1983.9369, 1986.8952, 1989.9174, 1994.8815, 2000.9197, 2005.9086, 2021.8911, 2056.0536, 2094.0666, 2109.8996, 2113.0822, 2128.0609, 2145.0641, 2224.1628, 2243.0980, 2443.2428, 2462.2547, 2473.2605, 2558.1567, 2574.1403, 2730.4559, 3191.4147, 3345.7177, 3348.6589

16. A0A6G5ZU77_9SAUR **Mass:** 59771 **Score:** 17 **Expect:** 30 **Matches:** 7

Carboxylic ester hydrolase OS=Vipera anatolica senliki OX=2604287 PE=2 SV=1

Observed	Mr(expt)	Mr(calc)	ppm	Start	End	Miss	Ions	Peptide
1205.6543	1204.6471	1204.5935	44.5	462	472	0	---	R.SITEAEEALSR.Q
1208.6533	1207.6460	1207.5370	90.2	383	392	0	---	K.FSEANHGQYR.L
1569.7966	1568.7893	1568.7981	-5.57	254	267	1	---	K.TPREAHTDAIMLSK.E
1585.7773	1584.7700	1584.7930	-14.49	254	267	1	---	K.TPREAHTDAIMLSK.E + Oxidation (M)
1685.8453	1684.8381	1684.7840	32.1	401	414	0	---	R.DYFMVCPLAELAEK.I
1859.9808	1858.9735	1858.8917	44.0	257	273	1	---	R.EAHTDAIMLSKEVGCAK.D
1954.9108	1953.9036	1953.9692	-33.60	401	416	1	---	R.DYFMVCPLAELAEKIR.K

No match to: 855.0780, 871.0503, 888.4499, 903.4366, 909.4935, 909.9951, 919.5794, 927.4661, 929.4770, 955.4912, 961.5699, 979.4703, 979.9708, 1022.1352, 1044.1135, 1057.5718, 1066.0963, 1066.4632, 1077.4726, 1117.6238, 1189.6643, 1212.6224, 1224.6497, 1227.6140, 1230.6575, 1232.1331, 1232.6485, 1237.6417, 1249.1193, 1256.6413, 1304.6017, 1408.8054, 1429.7346, 1477.8040, 1493.1123, 1521.7995, 1531.8534, 1547.8397, 1592.7956, 1649.7106, 1687.9391, 1703.9311, 1776.9571, 1802.9661, 1817.9764, 1829.8674, 1839.9471, 1843.9920, 1847.8682, 1855.9112, 1869.8569, 1885.8106, 1900.9041, 1913.9195, 1938.9168, 1942.9134, 1957.9288, 1961.9224, 1968.9271, 1971.9093, 1973.9214, 1983.9369, 1986.8952, 1989.9174, 1994.8815, 2000.9197, 2005.9086, 2021.8911, 2056.0536, 2094.0666, 2109.8996, 2113.0822, 2128.0609, 2145.0641, 2224.1628, 2243.0980, 2443.2428, 2462.2547, 2473.2605, 2558.1567, 2574.1403, 2730.4559, 3191.4147, 3345.7177, 3348.6589

17. A0A223PK58_DABRR **Mass:** 88544 **Score:** 16 **Expect:** 32 **Matches:** 7

Phospholipase A-2-activating protein (Fragment) OS=Daboia russelii OX=8707 PE=2 SV=1

Observed	Mr(expt)	Mr(calc)	ppm	Start	End	Miss	Ions	Peptide
1057.5718	1056.5646	1056.6331	-64.89	592	599	1	---	R.EQLKILWK.A
1829.8674	1828.8601	1828.8890	-15.79	617	632	1	---	R.LAVRHPSVNESFCGEK.X
1847.8682	1846.8609	1846.9532	-49.96	673	689	0	---	K.LMLAQGDSIMSQAIELK.L
1855.9112	1854.9039	1854.8788	13.6	362	376	1	---	K.VEAYQWSSSEDKWIK.I
1942.9134	1941.9061	1941.9003	2.99	457	474	1	---	K.GQMLGRTDTGFSDPFTGR.G
2000.9197	1999.9124	2000.0520	-69.79	600	616	0	---	K.AINWPNDFVFPALDILR.L
2145.0641	2144.0568	2144.0143	19.8	23	41	1	---	R.GLSCCLFPTGSFVSVSRDR.T

No match to: 855.0780, 871.0503, 888.4499, 903.4366, 909.4935, 909.9951, 919.5794, 927.4661, 929.4770, 955.4912, 961.5699, 979.4703, 979.9708, 1022.1352, 1044.1135, 1066.0963, 1066.4632, 1077.4726, 1117.6238, 1189.6643, 1205.6543, 1208.6533, 1212.6224, 1224.6497, 1227.6140, 1230.6575, 1232.1331, 1232.6485, 1237.6417, 1249.1193, 1256.6413, 1304.6017, 1408.8054, 1429.7346, 1477.8040, 1493.1123, 1521.7995, 1531.8534, 1547.8397, 1569.7966, 1585.7773, 1592.7956, 1649.7106, 1685.8453, 1687.9391, 1703.9311, 1776.9571, 1802.9661, 1817.9764, 1839.9471, 1843.9920, 1859.9808, 1869.8569, 1885.8106, 1900.9041, 1913.9195, 1938.9168, 1954.9108, 1957.9288, 1961.9224, 1968.9271, 1971.9093, 1973.9214, 1983.9369, 1986.8952, 1989.9174, 1994.8815, 2005.9086, 2021.8911, 2056.0536, 2094.0666, 2109.8996, 2113.0822, 2128.0609, 2224.1628, 2243.0980, 2443.2428, 2462.2547, 2473.2605, 2558.1567, 2574.1403, 2730.4559, 3191.4147, 3345.7177, 3348.6589

18. VSPA_MACLB **Mass:** 29274 **Score:** 16 **Expect:** 36 **Matches:** 4

Alpha-fibrinogenase OS=Macrovipera lebetina OX=8709 PE=1 SV=1

Observed	Mr(expt)	Mr(calc)	ppm	Start	End	Miss	Ions	Peptide
919.5794	918.5721	918.5174	59.5	176	184	0	---	R.AIYGSLPAK.S
927.4661	926.4588	926.4498	9.74	103	109	1	---	K.SYTKWDK.D
1687.9391	1686.9318	1686.9053	15.7	122	137	0	---	K.TSTHIAPLSLPSNPPR.L
1942.9134	1941.9061	1941.9805	-38.26	155	170	0	---	R.ETLPYVPHCANIMILR.Y + Oxidation (M)

No match to: 855.0780, 871.0503, 888.4499, 903.4366, 909.4935, 909.9951, 929.4770, 955.4912, 961.5699, 979.4703, 979.9708, 1022.1352, 1044.1135, 1057.5718, 1066.0963, 1066.4632, 1077.4726, 1117.6238, 1189.6643, 1205.6543, 1208.6533, 1212.6224, 1224.6497, 1227.6140, 1230.6575, 1232.1331, 1232.6485, 1237.6417, 1249.1193, 1256.6413, 1304.6017, 1408.8054, 1429.7346, 1477.8040, 1493.1123, 1521.7995, 1531.8534, 1547.8397, 1569.7966, 1585.7773, 1592.7956, 1649.7106, 1685.8453, 1703.9311, 1776.9571, 1802.9661, 1817.9764, 1829.8674, 1839.9471, 1843.9920, 1847.8682, 1855.9112, 1859.9808, 1869.8569, 1885.8106, 1900.9041, 1913.9195, 1938.9168, 1954.9108, 1957.9288, 1961.9224, 1968.9271, 1971.9093, 1973.9214, 1983.9369, 1986.8952, 1989.9174, 1994.8815, 2000.9197, 2005.9086, 2021.8911, 2056.0536, 2094.0666, 2109.8996, 2113.0822, 2128.0609, 2145.0641, 2224.1628, 2243.0980, 2443.2428, 2462.2547, 2473.2605, 2558.1567, 2574.1403, 2730.4559, 3191.4147, 3345.7177, 3348.6589

19. A0A6G5ZW74_9SAUR **Mass:** 32808 **Score:** 16 **Expect:** 37 **Matches:** 4

Veficolin 9 (Fragment) OS=Vipera anatolica senliki OX=2604287 PE=2 SV=1

Observed	Mr(expt)	Mr(calc)	ppm	Start	End	Miss	Ions	Peptide
909.4935	908.4863	908.4504	39.4	163	169	1	---	R.DWAAYKR.G
1942.9134	1941.9061	1941.8329	37.7	269	284	0	---	K.GGWWYNECHHANLNGK.Y
1989.9174	1988.9101	1988.9473	-18.68	10	28	1	---	R.GAESTCPEAQVVKLDGNSK.L
1994.8815	1993.8742	1993.8775	-1.68	136	152	0	---	R.VLCDMHTDGGGWTVFQR.R + Oxidation (M)

No match to: 855.0780, 871.0503, 888.4499, 903.4366, 909.9951, 919.5794, 927.4661, 929.4770, 955.4912, 961.5699, 979.4703, 979.9708, 1022.1352, 1044.1135, 1057.5718, 1066.0963, 1066.4632, 1077.4726, 1117.6238, 1189.6643, 1205.6543, 1208.6533, 1212.6224, 1224.6497, 1227.6140, 1230.6575, 1232.1331, 1232.6485, 1237.6417, 1249.1193, 1256.6413, 1304.6017, 1408.8054, 1429.7346, 1477.8040, 1493.1123, 1521.7995, 1531.8534, 1547.8397, 1569.7966, 1585.7773, 1592.7956, 1649.7106, 1685.8453, 1687.9391, 1703.9311, 1776.9571, 1802.9661, 1817.9764, 1829.8674, 1839.9471, 1843.9920, 1847.8682, 1855.9112, 1859.9808, 1869.8569, 1885.8106, 1900.9041, 1913.9195, 1938.9168, 1954.9108, 1957.9288, 1961.9224, 1968.9271, 1971.9093, 1973.9214, 1983.9369, 1986.8952, 2000.9197, 2005.9086, 2021.8911, 2056.0536, 2094.0666, 2109.8996, 2113.0822, 2128.0609, 2145.0641, 2224.1628, 2243.0980, 2443.2428, 2462.2547, 2473.2605, 2558.1567, 2574.1403, 2730.4559, 3191.4147, 3345.7177, 3348.6589

20. Q8UVG8_DABRR **Mass:** 52931 **Score:** 16 **Expect:** 38 **Matches:** 5

Vimentin OS=Daboia russelii OX=8707 PE=2 SV=1

Observed	Mr(expt)	Mr(calc)	ppm	Start	End	Miss	Ions	Peptide
1057.5718	1056.5646	1056.5134	48.4	369	376	1	---	K.EEMARHLR.E + Oxidation (M)
1408.8054	1407.7981	1407.7470	36.3	36	48	0	---	R.YSLGSSIRPSSVR.L
1776.9571	1775.9498	1775.8550	53.4	290	305	1	---	K.FADLSEAANRNNDALR.Q
1938.9168	1937.9095	1937.9483	-19.98	100	115	1	---	K.AELQELNDRFANYIDK.V
2443.2428	2442.2355	2442.1485	35.6	73	95	1	---	R.TGGDAMVDFSLADAINTEFKANR.T

No match to: 855.0780, 871.0503, 888.4499, 903.4366, 909.4935, 909.9951, 919.5794, 927.4661, 929.4770, 955.4912, 961.5699, 979.4703, 979.9708, 1022.1352, 1044.1135, 1066.0963, 1066.4632, 1077.4726, 1117.6238, 1189.6643, 1205.6543, 1208.6533, 1212.6224, 1224.6497, 1227.6140, 1230.6575, 1232.1331, 1232.6485, 1237.6417, 1249.1193, 1256.6413, 1304.6017, 1429.7346, 1477.8040, 1493.1123, 1521.7995, 1531.8534, 1547.8397, 1569.7966, 1585.7773, 1592.7956, 1649.7106, 1685.8453, 1687.9391, 1703.9311, 1802.9661, 1817.9764, 1829.8674, 1839.9471, 1843.9920, 1847.8682, 1855.9112, 1859.9808, 1869.8569, 1885.8106, 1900.9041, 1913.9195, 1942.9134, 1954.9108, 1957.9288, 1961.9224, 1968.9271, 1971.9093, 1973.9214, 1983.9369, 1986.8952, 1989.9174, 1994.8815, 2000.9197, 2005.9086, 2021.8911, 2056.0536, 2094.0666, 2109.8996, 2113.0822, 2128.0609, 2145.0641, 2224.1628, 2243.0980, 2462.2547, 2473.2605, 2558.1567, 2574.1403,

2730.4559, 3191.4147, 3345.7177, 3348.6589

Search Parameters

```
Type of search         : MS/MS Ion Search
Enzyme                 : Trypsin
Fixed modifications    : Carbamidomethyl (C)
Variable modifications : Oxidation (M)
Mass values            : Monoisotopic
Protein Mass           : Unrestricted
Peptide Mass Tolerance : ± 100 ppm
Fragment Mass Tolerance: ± 0.7 Da
Max Missed Cleavages   : 1
Instrument type        : MALDI-TOF-TOF
Query1  (855.0780,1+)   : <no title>
Query2  (871.0503,1+)   : <no title>
Query3  (888.4499,1+)   : <no title>
Query4  (903.4366,1+)   : \\PROTEOMICS1\D\DATA\2105\210505_TLIEPOL_1_68252-68266\68252_68266_LPARDO\0_H10\1\903.4366.LIFT\1SREF\PDATA\1\1R
Query5  (909.4935,1+)   : <no title>
Query6  (909.9951,1+)   : <no title>
Query7  (919.5794,1+)   : <no title>
Query8  (927.4661,1+)   : <no title>
Query9  (929.4770,1+)   : \\PROTEOMICS1\D\DATA\2105\210505_TLIEPOL_1_68252-68266\68252_68266_LPARDO\0_H10\1\929.4770.LIFT\1SREF\PDATA\1\1R
Query10 (955.4912,1+)   : <no title>
Query11 (961.5699,1+)   : <no title>
Query12 (979.4703,1+)   : <no title>
Query13 (979.9708,1+)   : <no title>
Query14 (1022.1352,1+) : <no title>
Query15 (1044.1135,1+) : <no title>
Query16 (1057.5718,1+) : <no title>
Query17 (1066.0963,1+) : <no title>
Query18 (1066.4632,1+) : <no title>
Query19 (1077.4726,1+) : <no title>
Query20 (1117.6238,1+) : \\PROTEOMICS1\D\DATA\2105\210505_TLIEPOL_1_68252-68266\68252_68266_LPARDO\0_H10\1\1117.6238.LIFT\1SREF\PDATA\1\1R
Query21 (1189.6643,1+) : <no title>
Query22 (1205.6543,1+) : <no title>
Query23 (1208.6533,1+) : <no title>
Query24 (1212.6224,1+) : <no title>
Query25 (1224.6497,1+) : <no title>
Query26 (1227.6140,1+) : <no title>
Query27 (1230.6575,1+) : <no title>
Query28 (1232.1331,1+) : <no title>
Query29 (1232.6485,1+) : <no title>
Query30 (1237.6417,1+) : <no title>
Query31 (1249.1193,1+) : <no title>
Query32 (1256.6413,1+) : <no title>
Query33 (1304.6017,1+) : <no title>
Query34 (1408.8054,1+) : \\PROTEOMICS1\D\DATA\2105\210505_TLIEPOL_1_68252-68266\68252_68266_LPARDO\0_H10\1\1408.8054.LIFT\1SREF\PDATA\1\1R
Query35 (1429.7346,1+) : <no title>
Query36 (1477.8040,1+) : <no title>
Query37 (1493.1123,1+) : <no title>
Query38 (1521.7995,1+) : <no title>
Query39 (1531.8534,1+) : \\PROTEOMICS1\D\DATA\2105\210505_TLIEPOL_1_68252-68266\68252_68266_LPARDO\0_H10\1\1531.8534.LIFT\1SREF\PDATA\1\1R
Query40 (1547.8397,1+) : <no title>
Query41 (1569.7966,1+) : <no title>
Query42 (1585.7773,1+) : <no title>
Query43 (1592.7956,1+) : <no title>
Query44 (1649.7106,1+) : <no title>
Query45 (1685.8453,1+) : <no title>
Query46 (1687.9391,1+) : <no title>
Query47 (1703.9311,1+) : <no title>
Query48 (1776.9571,1+) : <no title>
Query49 (1802.9661,1+) : <no title>
Query50 (1817.9764,1+) : \\PROTEOMICS1\D\DATA\2105\210505_TLIEPOL_1_68252-68266\68252_68266_LPARDO\0_H10\1\1817.9764.LIFT\1SREF\PDATA\1\1R
Query51 (1829.8674,1+) : <no title>
Query52 (1839.9471,1+) : <no title>
Query53 (1843.9920,1+) : <no title>
Query54 (1847.8682,1+) : \\PROTEOMICS1\D\DATA\2105\210505_TLIEPOL_1_68252-68266\68252_68266_LPARDO\0_H10\1\1847.8682.LIFT\1SREF\PDATA\1\1R
Query55 (1855.9112,1+) : <no title>
Query56 (1859.9808,1+) : <no title>
Query57 (1869.8569,1+) : <no title>
Query58 (1885.8106,1+) : <no title>
Query59 (1900.9041,1+) : <no title>
Query60 (1913.9195,1+) : <no title>
Query61 (1938.9168,1+) : <no title>
Query62 (1942.9134,1+) : <no title>
Query63 (1954.9108,1+) : <no title>
Query64 (1957.9288,1+) : \\PROTEOMICS1\D\DATA\2105\210505_TLIEPOL_1_68252-68266\68252_68266_LPARDO\0_H10\1\1957.9288.LIFT\1SREF\PDATA\1\1R
Query65 (1961.9224,1+) : <no title>
Query66 (1968.9271,1+) : <no title>
Query67 (1971.9093,1+) : <no title>
Query68 (1973.9214,1+) : <no title>
Query69 (1983.9369,1+) : <no title>
Query70 (1986.8952,1+) : <no title>
Query71 (1989.9174,1+) : <no title>
Query72 (1994.8815,1+) : <no title>
Query73 (2000.9197,1+) : <no title>
Query74 (2005.9086,1+) : <no title>
Query75 (2021.8911,1+) : <no title>
Query76 (2056.0536,1+) : <no title>
Query77 (2094.0666,1+) : <no title>
Query78 (2109.8996,1+) : <no title>
Query79 (2113.0822,1+) : \\PROTEOMICS1\D\DATA\2105\210505_TLIEPOL_1_68252-68266\68252_68266_LPARDO\0_H10\1\2113.0822.LIFT\1SREF\PDATA\1\1R
Query80 (2128.0609,1+) : <no title>
Query81 (2145.0641,1+) : <no title>
Query82 (2224.1628,1+) : <no title>
Query83 (2243.0980,1+) : <no title>
Query84 (2443.2428,1+) : <no title>
Query85 (2462.2547,1+) : \\PROTEOMICS1\D\DATA\2105\210505_TLIEPOL_1_68252-68266\68252_68266_LPARDO\0_H10\1\2462.2547.LIFT\1SREF\PDATA\1\1R
Query86 (2473.2605,1+) : <no title>
Query87 (2558.1567,1+) : \\PROTEOMICS1\D\DATA\2105\210505_TLIEPOL_1_68252-68266\68252_68266_LPARDO\0_H10\1\2558.1567.LIFT\1SREF\PDATA\1\1R
Query88 (2574.1403,1+) : <no title>
Query89 (2730.4559,1+) : <no title>
Query90 (3191.4147,1+) : \\PROTEOMICS1\D\DATA\2105\210505_TLIEPOL_1_68252-68266\68252_68266_LPARDO\0_H10\1\3191.4147.LIFT\1SREF\PDATA\1\1R
Query91 (3345.7177,1+) : <no title>
Query92 (3348.6589,1+) : <no title>
```

Mascot: http://www.matrixscience.com/

Mascot Search Results Fr6-4

User :
Email :
Search title : vipera_All_8IDplus4 file:\\Proteomics1\d\data\2105\210505_tliepol_1_68252-68266\68252_68266_lpardo\0_G11\1\1SRef\pdata\1\peaklist.xml
Database : vipera (1414 sequences; 347452 residues)
Timestamp : 5 May 2021 at 09:37:54 GMT
Top Score : 56 for VSP2_MACLB, Venom serine proteinase-like protein 2 OS=Macrovipera lebetina OX=8709 PE=2 SV=1

Mascot Score Histogram

Protein score is -10*Log(P), where P is the probability that the observed match is a random event.
Protein scores greater than 44 are significant (p<0.05).

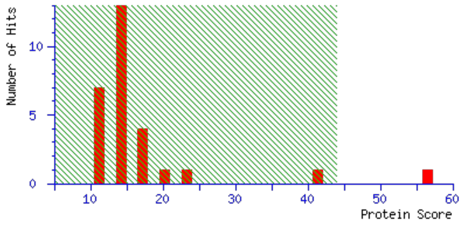

Concise Protein Summary Report

Format As | Concise Protein Summary | Help
Significance threshold p< 0.05 Max. number of hits 10

Re-Search All | Search Unmatched

1. VSP2_MACLB **Mass:** 29559 **Score:** 56 **Expect:** 0.0032 **Matches:** 10
Venom serine proteinase-like protein 2 OS=Macrovipera lebetina OX=8709 PE=2 SV=1
A0A6B7FMQ3_VIPAA **Mass:** 28909 **Score:** 16 **Expect:** 36 **Matches:** 3
Snake venom serine protease OS=Vipera ammodytes ammodytes OX=8705 PE=2 SV=1
A0A0H3U059_VIPAS **Mass:** 19999 **Score:** 13 **Expect:** 76 **Matches:** 2
Adrenocorticotropic hormone (Fragment) OS=Vipera aspis OX=8706 GN=POMC PE=3 SV=1
PA2N_DABRR **Mass:** 2192 **Score:** 12 **Expect:** 91 **Matches:** 1
Neutral phospholipase A2 RVV-PFIIc' (Fragment) OS=Daboia russelii OX=8707 PE=1 SV=1

---

2. A0A6G5ZVX7_9SAUR **Mass:** 29593 **Score:** 41 **Expect:** 0.1 **Matches:** 8
Serine protease 7 OS=Vipera anatolica senliki OX=2604287 PE=2 SV=1
A0A6G5ZUR6_9SAUR **Mass:** 29579 **Score:** 41 **Expect:** 0.1 **Matches:** 8
Serine protease 6 OS=Vipera anatolica senliki OX=2604287 PE=2 SV=1
VSPH1_VIPAA **Mass:** 29593 **Score:** 41 **Expect:** 0.1 **Matches:** 8
Vaa serine proteinase homolog 1 OS=Vipera ammodytes ammodytes OX=8705 PE=1 SV=1
A0A6G5ZUW7_9SAUR **Mass:** 22103 **Score:** 31 **Expect:** 1.1 **Matches:** 7
Serine protease 10 (Fragment) OS=Vipera anatolica senliki OX=2604287 PE=2 SV=1

---

3. VASP1_VIPAA **Mass:** 22635 **Score:** 22 **Expect:** 9.1 **Matches:** 4
Snake venom serine protease VaSP1 (Fragments) OS=Vipera ammodytes ammodytes OX=8705 PE=1 SV=1

---

4. VSPF5_MACLB **Mass:** 29261 **Score:** 20 **Expect:** 16 **Matches:** 5
Factor V activator OS=Macrovipera lebetina OX=8709 PE=1 SV=1

---

5. A0A6G5ZUQ8_9SAUR **Mass:** 130702 **Score:** 19 **Expect:** 20 **Matches:** 9
Rabankyrin OS=Vipera anatolica senliki OX=2604287 PE=2 SV=1

---

6. Q8UVG8_DABRR **Mass:** 52931 **Score:** 18 **Expect:** 22 **Matches:** 5
Vimentin OS=Daboia russelii OX=8707 PE=2 SV=1

---

7. A0A6G5ZVV8_9SAUR **Mass:** 89505 **Score:** 16 **Expect:** 36 **Matches:** 7
Dipeptidylpeptidase 1 (Fragment) OS=Vipera anatolica senliki OX=2604287 PE=2 SV=1
A0A1J0CZM7_VIPAA **Mass:** 17673 **Score:** 13 **Expect:** 73 **Matches:** 2
Snaclec-7 OS=Vipera ammodytes ammodytes OX=8705 PE=2 SV=1
A0A6G5ZVW0_9SAUR **Mass:** 91593 **Score:** 11 **Expect:** 1.1e+002 **Matches:** 6
Dipeptidylpeptidase 2 (Fragment) OS=Vipera anatolica senliki OX=2604287 PE=2 SV=1

---

8. A0A6G5ZUL5_9SAUR **Mass:** 33969 **Score:** 16 **Expect:** 37 **Matches:** 4
Veficolin 11 (Fragment) OS=Vipera anatolica senliki OX=2604287 PE=2 SV=1

---

9. A0A223PK38_DABRR **Mass:** 167642 **Score:** 16 **Expect:** 40 **Matches:** 8
Coagulation factor V OS=Daboia russelii OX=8707 PE=2 SV=1

---

10. A0A1I9KNR7_VIPAA **Mass:** 14615 **Score:** 15 **Expect:** 42 **Matches:** 2
Disintegrin Dis-1 OS=Vipera ammodytes ammodytes OX=8705 PE=2 SV=1
A0A1I9KNR4_VIPAA **Mass:** 14601 **Score:** 15 **Expect:** 42 **Matches:** 2
Disintegrin Dis-1 OS=Vipera ammodytes ammodytes OX=8705 PE=2 SV=1

Search Parameters

Type of search : Peptide Mass Fingerprint
Enzyme : Trypsin
Fixed modifications : Carbamidomethyl (C)
Variable modifications : Oxidation (M)
Mass values : Monoisotopic
Protein Mass : Unrestricted
Peptide Mass Tolerance : ± 100 ppm
Peptide Charge State : 1+
Max Missed Cleavages : 1
Number of queries : 92
Selected for scoring : 47

**Mascot:** http://www.matrixscience.com/

Mascot Search Results

```
User          :
Email         :
Search title  : vipera_all_Lift file:\\Proteomics1\d\data\2105\210505_tliepol_1_68252-68266\68252_68266_lpardo\0_G11\1\0_G11.xml
Database      : vipera  (1414 sequences; 347452 residues)
Timestamp     : 5 May 2021 at 10:08:24 GMT
Protein hits  : VSP2_MACLB        Venom serine proteinase-like protein 2 OS=Macrovipera lebetina OX=8709 PE=2 SV=1
                VASP1_VIPAA       Snake venom serine protease VaSP1 (Fragments) OS=Vipera ammodytes ammodytes OX=8705 PE=1 SV=1
                VSPF5_MACLB       Factor V activator OS=Macrovipera lebetina OX=8709 PE=1 SV=1
                A0A6G5ZVV8_9SAUR  Dipeptidylpeptidase 1 (Fragment) OS=Vipera anatolica senliki OX=2604287 PE=2 SV=1
                A0A223PK18_DABRR  C-type lectin domain family 16 member A (Fragment) OS=Daboia russelii OX=8707 PE=2 SV=1
                A0A6G9L6B2_VIPAM  Cytochrome c oxidase subunit 1 (Fragment) OS=Vipera ammodytes OX=8704 GN=COI PE=3 SV=1
```

Mascot Score Histogram

Ions score is -10*Log(P), where P is the probability that the observed match is a random event.
Individual ions scores > 12 indicate identity or extensive homology (p<0.05).
Protein scores are derived from ions scores as a non-probabilistic basis for ranking protein hits.

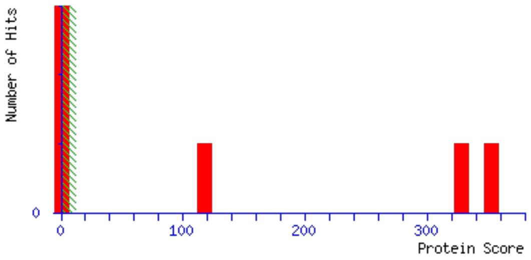

Peptide Summary Report

Format As: Peptide Summary — Help

Significance threshold p< 0.05 — Max. number of hits 10

Standard scoring ○ MudPIT scoring ◉ — Ions score or expect cut-off 0 — Show sub-sets 0

Show pop-ups ◉ Suppress pop-ups ○ — Sort unassigned Decreasing Score — Require bold red ☐

Overview Table

Click on column header to jump to entry in results list.
Move mouse over any indicator to highlight identical peptides.
Click on an indicator to see details of individual match.
Use check boxes to select sub-set of queries for new search.

Mouse over: -Query- -Accession- -Sequence-

Hit:	1	2	3	4	5	6
☑ 855.0839 (1+)						
☑ 866.5652 (1+)						
☑ 869.4391 (1+)						
☑ 871.0597 (1+)						
☑ 888.4497 (1+)	●					
☑ 914.4659 (1+)						
☑ 1058.5857 (1+)						
☑ 1067.4623 (1+)						
☑ 1077.4684 (1+)						
☑ 1126.5764 (1+)						
☑ 1151.2418 (1+)						
☑ 1172.6337 (1+)						
☑ 1187.6382 (1+)						
☑ 1188.6071 (1+)						
☑ 1189.6613 (1+)	●	●		●		
☑ 1205.6521 (1+)	●	●			●	
☑ 1208.6536 (1+)	●					
☑ 1212.6208 (1+)	●					
☑ 1215.6757 (1+)						
☑ 1221.6454 (1+)						
☑ 1224.6463 (1+)						
☑ 1227.6124 (1+)						
☑ 1231.6432 (1+)						

☑ 1232.1318 (1+)						
☑ 1234.6710 (1+)						
☑ 1237.6406 (1+)						
☑ 1243.6014 (1+)						
☑ 1246.6598 (1+)						
☑ 1250.6174 (1+)						
☑ 1256.6373 (1+)						
☑ 1277.7306 (1+)						
☑ 1279.5707 (1+)						
☑ 1280.0813 (1+)						
☑ 1304.5978 (1+)						
☑ 1320.5878 (1+)						
☑ 1372.7097 (1+)						
☑ 1404.1747 (1+)						
☑ 1404.6770 (1+)						
☑ 1408.8019 (1+)						
☑ 1429.7334 (1+)						
☑ 1440.7365 (1+)						
☑ 1475.7741 (1+)						
☑ 1531.8497 (1+)						
☑ 1547.8329 (1+)						
☑ 1581.7222 (1+)						
☑ 1649.7094 (1+)						
☑ 1687.9344 (1+)						
☑ 1802.9425 (1+)						
☑ 1817.9724 (1+)			●			
☑ 1847.8615 (1+)						
☑ 1957.9238 (1+)						
☑ 1993.9932 (1+)						
☑ 2022.0576 (1+)						
☑ 2053.0407 (1+)						
☑ 2056.0529 (1+)						
☑ 2061.0381 (1+)						
☑ 2069.0547 (1+)						
☑ 2094.0682 (1+)						
☑ 2098.0584 (1+)						
☑ 2106.0616 (1+)						
☑ 2113.0782 (1+)	●	●				
☑ 2118.0725 (1+)						
☑ 2125.0731 (1+)						
☑ 2128.0741 (1+)						
☑ 2135.0518 (1+)						
☑ 2139.0868 (1+)						
☑ 2142.0621 (1+)						
☑ 2145.0668 (1+)						
☑ 2155.0825 (1+)						
☑ 2160.0656 (1+)						
☑ 2171.0780 (1+)						
☑ 2265.0755 (1+)						
☑ 2405.2266 (1+)						
☑ 2417.2380 (1+)						
☑ 2442.0921 (1+)						
☑ 2443.2436 (1+)						
☑ 2455.2338 (1+)						
☑ 2462.2518 (1+)		●				
☑ 2473.2567 (1+)						
☑ 2476.2685 (1+)						
☑ 2484.2157 (1+)						
☑ 2488.2653 (1+)						
☑ 2501.1605 (1+)						
☑ 2512.1416 (1+)						
☑ 2530.2773 (1+)						
☑ 2539.1490 (1+)						
☑ 2558.1567 (1+)	●					
☑ 2569.1587 (1+)						
☑ 2574.1447 (1+)	●					●
☑ 2575.3339 (1+)						
☑ 2614.2493 (1+)						

☑ **3348.6481 (1+)**

Select All | Select None | Search Selected | ☐ **Error tolerant** | Archive Report

1. VSP2_MACLB **Mass:** 29559 **Score:** 352 **Matches:** 8(8) **Sequences:** 6(6)
Venom serine proteinase-like protein 2 OS=Macrovipera lebetina OX=8709 PE=2 SV=1
☐ Check to include this hit in error tolerant search or archive report

Query	Observed	Mr(expt)	Mr(calc)	ppm	Miss	Score	Expect	Rank	Unique	Peptide
5	888.4497	887.4425	887.4211	24.0	0	26	0.0027	1	U	K.FFCLSSK.T
15	1189.6613	1188.6541	1188.6325	18.1	1	81	1e-008	1		R.WDKDIMLIR.L
16	1205.6521	1204.6448	1204.6274	14.4	1	(30)	0.0015	1		R.WDKDIMLIR.L + Oxidation (M)
17	1208.6536	1207.6464	1207.6271	15.9	0	41	0.00016	1	U	R.IMGWGTITTTK.V
18	1212.6208	1211.6135	1211.5894	19.9	0	29	0.0011	1	U	K.NVPNEDQQIR.V
61	2113.0782	2112.0710	2112.0462	11.7	0	115	2.9e-012	1		R.FYCAGTLINQEWVLTAAR.C
87	2558.1567	2557.1494	2557.1189	11.9	0	97	1.9e-010	1	U	K.VTYPDVPHCANINMFDYSVCR.K
89	2574.1447	2573.1374	2573.1138	9.19	0	(27)	0.0022	1	U	K.VTYPDVPHCANINMFDYSVCR.K + Oxidation (M)

---

2. VASP1_VIPAA **Mass:** 22635 **Score:** 325 **Matches:** 4(4) **Sequences:** 3(3)
Snake venom serine protease VaSP1 (Fragments) OS=Vipera ammodytes ammodytes OX=8705 PE=1 SV=1
☐ Check to include this hit in error tolerant search or archive report

Query	Observed	Mr(expt)	Mr(calc)	ppm	Miss	Score	Expect	Rank	Unique	Peptide
15	1189.6613	1188.6541	1188.6325	18.1	1	81	1e-008	1		R.WDKDIMLIR.L
16	1205.6521	1204.6448	1204.6274	14.4	1	(30)	0.0015	1		R.WDKDIMLIR.L + Oxidation (M)
61	2113.0782	2112.0710	2112.0462	11.7	0	115	2.9e-012	1		R.FYCAGTLINQEWVLTAAR.C
78	2462.2518	2461.2446	2461.2172	11.1	0	139	1.4e-014	1	U	-.VIGGDECNINEHPFLVALHTAR.X

---

3. VSPF5_MACLB **Mass:** 29261 **Score:** 108 **Matches:** 1(1) **Sequences:** 1(1)
Factor V activator OS=Macrovipera lebetina OX=8709 PE=1 SV=1
☐ Check to include this hit in error tolerant search or archive report

Query	Observed	Mr(expt)	Mr(calc)	ppm	Miss	Score	Expect	Rank	Unique	Peptide
49	1817.9724	1816.9652	1816.9431	12.1	0	108	1.6e-011	1	U	K.NSAHIAPISLPSSPSSPR.S

---

4. A0A6G5ZVV8_9SAUR **Mass:** 89505 **Score:** 0 **Matches:** 1(0) **Sequences:** 1(0)
Dipeptidylpeptidase 1 (Fragment) OS=Vipera anatolica senliki OX=2604287 PE=2 SV=1
☐ Check to include this hit in error tolerant search or archive report

Query	Observed	Mr(expt)	Mr(calc)	ppm	Miss	Score	Expect	Rank	Unique	Peptide
15	1189.6613	1188.6541	1188.6615	-6.28	1	7	0.24	2	U	K.WVSNTKTVVR.W

Proteins matching the same set of peptides:
A0A6G5ZVW0_9SAUR **Mass:** 91593 **Score:** 0 **Matches:** 1(0) **Sequences:** 1(0)
Dipeptidylpeptidase 2 (Fragment) OS=Vipera anatolica senliki OX=2604287 PE=2 SV=1

---

5. A0A223PK18_DABRR **Score:** 0 **Matches:** 1(0) **Sequences:** 1(0)
C-type lectin domain family 16 member A (Fragment) OS=Daboia russelii OX=8707 PE=2 SV=1
☐ Check to include this hit in error tolerant search or archive report

Query	Observed	Mr(expt)	Mr(calc)	ppm	Miss	Score	Expect	Rank	Unique	Peptide
16	1205.6521	1204.6448	1204.5619	68.8	1	3	0.81	2	U	R.RSSCDPTVQR.S

---

6. A0A6G9L6B2_VIPAM **Score:** 0 **Matches:** 1(0) **Sequences:** 1(0)
Cytochrome c oxidase subunit 1 (Fragment) OS=Vipera ammodytes OX=8704 GN=COI PE=3 SV=1
☐ Check to include this hit in error tolerant search or archive report

Query	Observed	Mr(expt)	Mr(calc)	ppm	Miss	Score	Expect	Rank	Unique	Peptide
89	2574.1447	2573.1374	2573.3320	-75.63	1	1	0.78	2	U	-.TLYLMFGAWSRLVGACLSILMR.M + Oxidation (M)

Peptide matches not assigned to protein hits: (no details means no match)

	Query	Observed	Mr(expt)	Mr(calc)	ppm	Miss	Score	Expect	Rank	Unique	Peptide
☑	1	*855.0839*	*854.0767*								
☑	2	*866.5652*	*865.5580*								
☑	3	*869.4391*	*868.4319*								
☑	4	*871.0597*	*870.0525*								
☑	6	*914.4659*	*913.4587*								
☑	7	*1058.5857*	*1057.5784*								
☑	8	*1067.4623*	*1066.4550*								
☑	9	*1077.4684*	*1076.4611*								
☑	10	*1126.5764*	*1125.5691*								
☑	11	*1151.2418*	*1150.2345*								
☑	12	*1172.6337*	*1171.6264*								
☑	13	*1187.6382*	*1186.6309*								
☑	14	*1188.6071*	*1187.5998*								

☑	19	*1215.6757*	*1214.6684*
☑	20	*1221.6454*	*1220.6382*
☑	21	*1224.6463*	*1223.6390*
☑	22	*1227.6124*	*1226.6051*
☑	23	*1231.6432*	*1230.6359*
☑	24	*1232.1318*	*1231.1246*
☑	25	*1234.6710*	*1233.6637*
☑	26	*1237.6406*	*1236.6333*
☑	27	*1243.6014*	*1242.5941*
☑	28	*1246.6598*	*1245.6525*
☑	29	*1250.6174*	*1249.6101*
☑	30	*1256.6373*	*1255.6301*
☑	31	*1277.7306*	*1276.7233*
☑	32	*1279.5707*	*1278.5634*
☑	33	*1280.0813*	*1279.0740*
☑	34	*1304.5978*	*1303.5905*
☑	35	*1320.5878*	*1319.5805*
☑	36	*1372.7097*	*1371.7024*
☑	37	*1404.1747*	*1403.1674*
☑	38	*1404.6770*	*1403.6697*
☑	39	*1408.8019*	*1407.7946*
☑	40	*1429.7334*	*1428.7261*
☑	41	*1440.7365*	*1439.7292*
☑	42	*1475.7741*	*1474.7668*
☑	43	*1531.8497*	*1530.8425*
☑	44	*1547.8329*	*1546.8256*
☑	45	*1581.7222*	*1580.7149*
☑	46	*1649.7094*	*1648.7021*
☑	47	*1687.9344*	*1686.9272*
☑	48	*1802.9425*	*1801.9352*
☑	50	*1847.8615*	*1846.8542*
☑	51	*1957.9238*	*1956.9165*
☑	52	*1993.9932*	*1992.9859*
☑	53	*2022.0576*	*2021.0503*
☑	54	*2053.0407*	*2052.0334*
☑	55	*2056.0529*	*2055.0457*
☑	56	*2061.0381*	*2060.0308*
☑	57	*2069.0547*	*2068.0474*
☑	58	*2094.0682*	*2093.0609*
☑	59	*2098.0584*	*2097.0511*
☑	60	*2106.0616*	*2105.0544*
☑	62	*2118.0725*	*2117.0652*
☑	63	*2125.0731*	*2124.0658*
☑	64	*2128.0741*	*2127.0669*
☑	65	*2135.0518*	*2134.0445*
☑	66	*2139.0868*	*2138.0795*
☑	67	*2142.0621*	*2141.0548*
☑	68	*2145.0668*	*2144.0595*
☑	69	*2155.0825*	*2154.0752*
☑	70	*2160.0656*	*2159.0583*
☑	71	*2171.0780*	*2170.0707*
☑	72	*2265.0755*	*2264.0682*
☑	73	*2405.2266*	*2404.2193*
☑	74	*2417.2380*	*2416.2308*
☑	75	*2442.0921*	*2441.0848*
☑	76	*2443.2436*	*2442.2363*
☑	77	*2455.2338*	*2454.2265*
☑	79	*2473.2567*	*2472.2494*
☑	80	*2476.2685*	*2475.2612*
☑	81	*2484.2157*	*2483.2084*
☑	82	*2488.2653*	*2487.2580*
☑	83	*2501.1605*	*2500.1533*
☑	84	*2512.1416*	*2511.1344*
☑	85	*2530.2773*	*2529.2700*
☑	86	*2539.1490*	*2538.1417*
☑	88	*2569.1587*	*2568.1515*
☑	90	*2575.3339*	*2574.3266*
☑	91	*2614.2493*	*2613.2420*
☑	92	*3348.6481*	*3347.6408*

Search Parameters

```
Type of search         : MS/MS Ion Search
Enzyme                 : Trypsin
Fixed modifications    : Carbamidomethyl (C)
Variable modifications : Oxidation (M)
Mass values            : Monoisotopic
Protein Mass           : Unrestricted
Peptide Mass Tolerance : ± 100 ppm
Fragment Mass Tolerance: ± 0.7 Da
Max Missed Cleavages   : 1
Instrument type        : MALDI-TOF-TOF
```

Number of queries : 92

**Mascot:** http://www.matrixscience.com/

Mascot Search Results

```
User          :
Email         :
Search title  : vipera_all_Lift file:\\Proteomics1\d\data\2105\210505_tliepol_1_68252-68266\68252_68266_lpardo\0_G11\1\0_G11.xml
Database      : vipera  (1414 sequences; 347452 residues)
Timestamp     : 5 May 2021 at 10:08:24 GMT
Warning       : A Peptide summary report will usually give a much clearer picture of MS/MS search results.
Top Score     : 426 for VSP2_MACLB, Venom serine proteinase-like protein 2 OS=Macrovipera lebetina OX=8709 PE=2 SV=1
```

Mascot Score Histogram

Protein score is -10*Log(P), where P is the probability that the observed match is a random event.
Protein scores greater than 44 are significant (p<0.05).
Protein scores are derived from ions scores as a non-probabilistic basis for ranking protein hits.

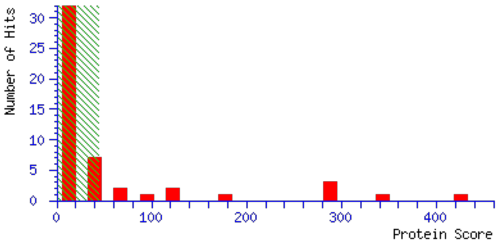

Protein Summary Report

Format As | Protein Summary (deprecated) | Help

Significance threshold p< 0.05 Max. number of hits 30

Overview Table

Click on column header to jump to entry in results list.
Move mouse over any indicator to highlight identical peptides.
Click on an indicator to see details of individual match.
Use check boxes to select sub-set of queries for new search.

Mouse over: -Query- -Accession- -Sequence-

Hit:	1	2	3	4	5	6	7	8	9	10	11	12	13	14	15	16	17	18	19	20	21	22	23	24	25	26	27	28	29	30
☑ 855.0839 (1+)																														
☑ 866.5652 (1+)																														
☑ 869.4391 (1+)																														
☑ 871.0597 (1+)																														
☑ 888.4497 (1+)	●		●	●	●	●		●				●		●	●	●	●	●												
☑ 914.4659 (1+)																														
☑ 1058.5857 (1+)																														
☑ 1067.4623 (1+)																														
☑ 1077.4684 (1+)																														
☑ 1126.5764 (1+)																														
☑ 1151.2418 (1+)																														
☑ 1172.6337 (1+)																														
☑ 1187.6382 (1+)																														
☑ 1188.6071 (1+)																														
☑ 1189.6613 (1+)	●	●	●	●	●	●		●	●	●	●										●									
☑ 1205.6521 (1+)	●	●	●	●	●	●		●	●	●	●																			
☑ 1208.6536 (1+)	●		●	●	●	●																								
☑ 1212.6208 (1+)	●																													
☑ 1215.6757 (1+)																														
☑ 1221.6454 (1+)																														
☑ 1224.6463 (1+)																														
☑ 1227.6124 (1+)																														
☑ 1231.6432 (1+)																														
☑ 1232.1318 (1+)																														
☑ 1234.6710 (1+)																														
☑ 1237.6406 (1+)																														
☑ 1243.6014 (1+)																														
☑ 1246.6598 (1+)																														
☑ 1250.6174 (1+)																														
☑ 1256.6373 (1+)																														
☑ 1277.7306 (1+)																														
☑ 1279.5707 (1+)																														
☑ 1280.0813 (1+)																														
☑ 1304.5978 (1+)																														

☑ 1320.5878 (1+)																														
☑ 1372.7097 (1+)																														
☑ 1404.1747 (1+)																														
☑ 1404.6770 (1+)																														
☑ 1408.8019 (1+)																														
☑ 1429.7334 (1+)																														
☑ 1440.7365 (1+)																														
☑ 1475.7741 (1+)																														
☑ 1531.8497 (1+)																														
☑ 1547.8329 (1+)																														
☑ 1581.7222 (1+)																														
☑ 1649.7094 (1+)																														
☑ 1687.9344 (1+)																														
☑ 1802.9425 (1+)																														
☑ 1817.9724 (1+)							●																							
☑ 1847.8615 (1+)																														
☑ 1957.9238 (1+)																														
☑ 1993.9932 (1+)																														
☑ 2022.0576 (1+)																														
☑ 2053.0407 (1+)																														
☑ 2056.0529 (1+)																														
☑ 2061.0381 (1+)																														
☑ 2069.0547 (1+)																														
☑ 2094.0682 (1+)																														
☑ 2098.0584 (1+)																														
☑ 2106.0616 (1+)																														
☑ 2113.0782 (1+)	●	●	●	●	●																									
☑ 2118.0725 (1+)																														
☑ 2125.0731 (1+)																														
☑ 2128.0741 (1+)																														
☑ 2135.0518 (1+)																														
☑ 2139.0868 (1+)																														
☑ 2142.0621 (1+)																														
☑ 2145.0668 (1+)																														
☑ 2155.0825 (1+)																														
☑ 2160.0656 (1+)																														
☑ 2171.0780 (1+)																														
☑ 2265.0755 (1+)																														
☑ 2405.2266 (1+)																														
☑ 2417.2380 (1+)																														
☑ 2442.0921 (1+)																														
☑ 2443.2436 (1+)																														
☑ 2455.2338 (1+)																														
☑ 2462.2518 (1+)		●																												
☑ 2473.2567 (1+)																														
☑ 2476.2685 (1+)																														
☑ 2484.2157 (1+)																														
☑ 2488.2653 (1+)																														
☑ 2501.1605 (1+)																														
☑ 2512.1416 (1+)																														
☑ 2530.2773 (1+)																														
☑ 2539.1490 (1+)																														
☑ 2558.1567 (1+)	●																													
☑ 2569.1587 (1+)																														
☑ 2574.1447 (1+)	●																													
☑ 2575.3339 (1+)																														
☑ 2614.2493 (1+)																														
☑ 3348.6481 (1+)																														

Select All | Select None | Search Selected

Index

	Accession	Mass	Score	Description
1.	VSP2_MACLB	29559	426	Venom serine proteinase-like protein 2 OS=Macrovipera lebetina OX=8709 PE=2 SV=1
2.	VASP1_VIPAA	22635	350	Snake venom serine protease VaSP1 (Fragments) OS=Vipera ammodytes ammodytes OX=8705 PE=1 SV=1
3.	A0A6G5ZVX7_9SAUR	29593	288	Serine protease 7 OS=Vipera anatolica senliki OX=2604287 PE=2 SV=1
4.	A0A6G5ZUR6_9SAUR	29579	288	Serine protease 6 OS=Vipera anatolica senliki OX=2604287 PE=2 SV=1
5.	VSPH1_VIPAA	29593	288	Vaa serine proteinase homolog 1 OS=Vipera ammodytes ammodytes OX=8705 PE=1 SV=1
6.	A0A6G5ZUW7_9SAUR	22103	166	Serine protease 10 (Fragment) OS=Vipera anatolica senliki OX=2604287 PE=2 SV=1
7.	VSPF5_MACLB	29261	134	Factor V activator OS=Macrovipera lebetina OX=8709 PE=1 SV=1
8.	A0A6B7FMQ3_VIPAA	28909	110	Snake venom serine protease OS=Vipera ammodytes ammodytes OX=8705 PE=2 SV=1
9.	D8MIA0_BITRH	29517	86	Rhinocerase 2 protein (Fragment) OS=Bitis rhinoceros OX=715877 GN=rhinocerase 2 PE=3 SV=1
10.	D8MIA1_BITRH	29504	81	Rhinocerase 3 protein (Fragment) OS=Bitis rhinoceros OX=715877 GN=rhinocerase 3 PE=3 SV=1
11.	VSPY_MACLB	28944	78	Chymotrypsin-like protease VLCTLP OS=Macrovipera lebetina OX=8709 PE=1 SV=1
12.	VSPBF_MACLB	28963	32	Beta-fibrinogenase OS=Macrovipera lebetina OX=8709 PE=1 SV=1
13.	A0A0H3Y7H2_9PERO	22231	29	Uncharacterized protein (Fragment) OS=Echiichthys vipera OX=94984 GN=ptr PE=4 SV=1
14.	A0A1I9KNR8_VIPAA	29252	26	Snake venom serine protease OS=Vipera ammodytes ammodytes OX=8705 PE=2 SV=1

15.	**A0A6B7FQF8_VIPAA**	28983	26	Serine proteinase SP-6 OS=Vipera ammodytes ammodytes OX=8705 PE=2 SV=1
16.	**A0A6G5ZW78_9SAUR**	28826	26	Serine protease 2 OS=Vipera anatolica senliki OX=2604287 PE=2 SV=1
17.	**VSP3_MACLB**	29017	26	Serine protease VLSP-3 OS=Macrovipera lebetina OX=8709 PE=2 SV=1
18.	**VSP_VIPBN**	28882	26	Snake venom serine protease nikobin OS=Vipera berus nikolskii OX=1808362 GN=sp-VN PE=2 SV=1
19.	**A0A6G5ZW27_9SAUR**	38471	23	Venom factor 6 (Fragment) OS=Vipera anatolica senliki OX=2604287 PE=2 SV=1
20.	**A0A6G5ZUA3_9SAUR**	25767	20	Cysteine-rich secretory protein 1 (Fragment) OS=Vipera anatolica senliki OX=2604287 PE=2 SV=1
21.	**A0A6G5ZVV8_9SAUR**	89505	20	Dipeptidylpeptidase 1 (Fragment) OS=Vipera anatolica senliki OX=2604287 PE=2 SV=1
22.	**A0A0H3Y6S3_9PERO**	29853	20	Glycotransferase (Fragment) OS=Echiichthys vipera OX=94984 GN=glyt PE=3 SV=1
23.	**A0A6G5ZUS6_9SAUR**	13128	20	Coagulation factor V 5 (Fragment) OS=Vipera anatolica senliki OX=2604287 PE=2 SV=1
24.	**A0A6G5ZUQ0_9SAUR**	27438	19	Putative serine carboxypeptidase 1 (Fragment) OS=Vipera anatolica senliki OX=2604287 PE=2 SV=1
25.	**PA2AB_DABSI**	15673	19	Acidic phospholipase A2 daboiatoxin B chain (Fragment) OS=Daboia siamensis OX=343250 PE=1 SV=1
26.	**A0A6G5ZW20_9SAUR**	26588	18	Veficolin 8 (Fragment) OS=Vipera anatolica senliki OX=2604287 PE=2 SV=1
27.	**A0A6G5ZVX6_9SAUR**	37165	18	Veficolin 1 OS=Vipera anatolica senliki OX=2604287 PE=2 SV=1
28.	**A0A6G5ZW74_9SAUR**	32808	18	Veficolin 9 (Fragment) OS=Vipera anatolica senliki OX=2604287 PE=2 SV=1
29.	**A0A170TXL0_VIPUR**	25071	18	Brain-derived neurotrophic factor (Fragment) OS=Vipera ursinii macrops OX=927675 GN=BDNF PE=3 SV=1
30.	**A0A6G5ZUQ8_9SAUR**	130702	17	Rabankyrin OS=Vipera anatolica senliki OX=2604287 PE=2 SV=1

Results List

1. VSP2_MACLB **Mass:** 29559 **Score:** 426 **Expect:** 3.6e-040 **Matches:** 10

Venom serine proteinase-like protein 2 OS=Macrovipera lebetina OX=8709 PE=2 SV=1

Observed	Mr(expt)	Mr(calc)	ppm	Start	End	Miss	Ions	Peptide
866.5652	865.5580	865.5385	22.5	75 -	82	0	---	R.IILGVHSK.N
888.4497	887.4425	887.4211	24.0	98 -	104	0	26	K.FFCLSSK.T
1189.6613	1188.6541	1188.6325	18.1	109 -	117	1	81	R.WDKDIMLIR.L
1205.6521	1204.6448	1204.6274	14.4	109 -	117	1	(30)	R.WDKDIMLIR.L + Oxidation (M)
1208.6536	1207.6464	1207.6271	15.9	146 -	156	0	41	R.IMGWGTITTTK.V
1212.6208	1211.6135	1211.5894	19.9	83 -	92	0	29	K.NVPNEDQQIR.V
1224.6463	1223.6390	1223.6220	13.9	146 -	156	0	---	R.IMGWGTITTTK.V + Oxidation (M)
2113.0782	2112.0710	2112.0462	11.7	50 -	67	0	115	R.FYCAGTLINQEWVLTAAR.C
2558.1567	2557.1494	2557.1189	11.9	157 -	177	0	97	K.VTYPDVPHCANINMFDYSVCR.K
2574.1447	2573.1374	2573.1138	9.19	157 -	177	0	(27)	K.VTYPDVPHCANINMFDYSVCR.K + Oxidation (M)

No match to: 855.0839, 869.4391, 871.0597, 914.4659, 1058.5857, 1067.4623, 1077.4684, 1126.5764, 1151.2418, 1172.6337, 1187.6382, 1188.6071, 1215.6757, 1221.6454, 1227.6124, 1231.6432, 1232.1318, 1234.6710, 1237.6406, 1243.6014, 1246.6598, 1250.6174, 1256.6373, 1277.7306, 1279.5707, 1280.0813, 1304.5978, 1320.5878, 1372.7097, 1404.1747, 1404.6770, 1408.8019, 1429.7334, 1440.7365, 1475.7741, 1531.8497, 1547.8329, 1581.7222, 1649.7094, 1687.9344, 1802.9425, 1817.9724, 1847.8615, 1957.9238, 1993.9932, 2022.0576, 2053.0407, 2056.0529, 2061.0381, 2069.0547, 2094.0682, 2098.0584, 2106.0616, 2118.0725, 2125.0731, 2128.0741, 2135.0518, 2139.0868, 2142.0621, 2145.0668, 2155.0825, 2160.0656, 2171.0780, 2265.0755, 2405.2266, 2417.2380, 2442.0921, 2443.2436, 2455.2338, 2462.2518, 2473.2567, 2476.2685, 2484.2157, 2488.2653, 2501.1605, 2512.1416, 2530.2773, 2539.1490, 2569.1587, 2575.3339, 2614.2493, 3348.6481

2. VASP1_VIPAA **Mass:** 22635 **Score:** 350 **Expect:** 1.4e-032 **Matches:** 4

Snake venom serine protease VaSP1 (Fragments) OS=Vipera ammodytes ammodytes OX=8705 PE=1 SV=1

Observed	Mr(expt)	Mr(calc)	ppm	Start	End	Miss	Ions	Peptide
1189.6613	1188.6541	1188.6325	18.1	85 -	93	1	81	R.WDKDIMLIR.L
1205.6521	1204.6448	1204.6274	14.4	85 -	93	1	(30)	R.WDKDIMLIR.L + Oxidation (M)
2113.0782	2112.0710	2112.0462	11.7	26 -	43	0	115	R.FYCAGTLINQEWVLTAAR.C
2462.2518	2461.2446	2461.2172	11.1	1 -	22	0	139	-.VIGGDECNINEHPFLVALHTAR.X

No match to: 855.0839, 866.5652, 869.4391, 871.0597, 888.4497, 914.4659, 1058.5857, 1067.4623, 1077.4684, 1126.5764, 1151.2418, 1172.6337, 1187.6382, 1188.6071, 1208.6536, 1212.6208, 1215.6757, 1221.6454, 1224.6463, 1227.6124, 1231.6432, 1232.1318, 1234.6710, 1237.6406, 1243.6014, 1246.6598, 1250.6174, 1256.6373, 1277.7306, 1279.5707, 1280.0813, 1304.5978, 1320.5878, 1372.7097, 1404.1747, 1404.6770, 1408.8019, 1429.7334, 1440.7365, 1475.7741, 1531.8497, 1547.8329, 1581.7222, 1649.7094, 1687.9344, 1802.9425, 1817.9724, 1847.8615, 1957.9238, 1993.9932, 2022.0576, 2053.0407, 2056.0529, 2061.0381, 2069.0547, 2094.0682, 2098.0584, 2106.0616, 2118.0725, 2125.0731, 2128.0741, 2135.0518, 2139.0868, 2142.0621, 2145.0668, 2155.0825, 2160.0656, 2171.0780, 2265.0755, 2405.2266, 2417.2380, 2442.0921, 2443.2436, 2455.2338, 2473.2567, 2476.2685, 2484.2157, 2488.2653, 2501.1605, 2512.1416, 2530.2773, 2539.1490, 2558.1567, 2569.1587, 2574.1447, 2575.3339, 2614.2493, 3348.6481

3. A0A6G5ZVX7_9SAUR **Mass:** 29593 **Score:** 288 **Expect:** 2.2e-026 **Matches:** 8

Serine protease 7 OS=Vipera anatolica senliki OX=2604287 PE=2 SV=1

Observed	Mr(expt)	Mr(calc)	ppm	Start	End	Miss	Ions	Peptide
866.5652	865.5580	865.5385	22.5	75 -	82	0	---	R.IILGVHSK.N
888.4497	887.4425	887.4211	24.0	98 -	104	0	26	K.FFCLSSK.T
1189.6613	1188.6541	1188.6325	18.1	109 -	117	1	81	R.WDKDIMLIR.L
1205.6521	1204.6448	1204.6274	14.4	109 -	117	1	(30)	R.WDKDIMLIR.L + Oxidation (M)
1208.6536	1207.6464	1207.6271	15.9	146 -	156	0	41	R.IMGWGTITTTK.V
1224.6463	1223.6390	1223.6220	13.9	146 -	156	0	---	R.IMGWGTITTTK.V + Oxidation (M)
1231.6432	1230.6359	1230.5299	86.2	83 -	92	0	---	K.NVPNEDEQMR.V
2113.0782	2112.0710	2112.0462	11.7	50 -	67	0	115	R.FYCAGTLINQEWVLTAAR.C

No match to: 855.0839, 869.4391, 871.0597, 914.4659, 1058.5857, 1067.4623, 1077.4684, 1126.5764, 1151.2418, 1172.6337, 1187.6382, 1188.6071, 1212.6208, 1215.6757, 1221.6454, 1227.6124, 1232.1318, 1234.6710, 1237.6406, 1243.6014, 1246.6598, 1250.6174, 1256.6373, 1277.7306, 1279.5707, 1280.0813, 1304.5978, 1320.5878, 1372.7097, 1404.1747, 1404.6770, 1408.8019, 1429.7334, 1440.7365, 1475.7741, 1531.8497, 1547.8329, 1581.7222, 1649.7094, 1687.9344, 1802.9425, 1817.9724, 1847.8615, 1957.9238, 1993.9932, 2022.0576, 2053.0407, 2056.0529, 2061.0381, 2069.0547, 2094.0682, 2098.0584, 2106.0616, 2118.0725, 2125.0731, 2128.0741, 2135.0518, 2139.0868, 2142.0621, 2145.0668, 2155.0825, 2160.0656, 2171.0780, 2265.0755, 2405.2266, 2417.2380, 2442.0921, 2443.2436, 2455.2338, 2462.2518, 2473.2567, 2476.2685, 2484.2157, 2488.2653, 2501.1605, 2512.1416, 2530.2773, 2539.1490, 2558.1567, 2569.1587, 2574.1447, 2575.3339, 2614.2493, 3348.6481

4. A0A6G5ZUR6_9SAUR **Mass:** 29579 **Score:** 288 **Expect:** 2.2e-026 **Matches:** 8

Serine protease 6 OS=Vipera anatolica senliki OX=2604287 PE=2 SV=1

Observed	Mr(expt)	Mr(calc)	ppm	Start	End	Miss	Ions	Peptide
866.5652	865.5580	865.5385	22.5	75 -	82	0	---	R.IILGVHSK.N
888.4497	887.4425	887.4211	24.0	98 -	104	0	26	K.FFCLSSK.S
1189.6613	1188.6541	1188.6325	18.1	109 -	117	1	81	R.WDKDIMLIR.L
1205.6521	1204.6448	1204.6274	14.4	109 -	117	1	(30)	R.WDKDIMLIR.L + Oxidation (M)
1208.6536	1207.6464	1207.6271	15.9	146 -	156	0	41	R.IMGWGTITTTK.V
1224.6463	1223.6390	1223.6220	13.9	146 -	156	0	---	R.IMGWGTITTTK.V + Oxidation (M)
1231.6432	1230.6359	1230.5299	86.2	83 -	92	0	---	K.NVPNEDEQMR.V
2113.0782	2112.0710	2112.0462	11.7	50 -	67	0	115	R.FYCAGTLINQEWVLTAAR.C

No match to: 855.0839, 869.4391, 871.0597, 914.4659, 1058.5857, 1067.4623, 1077.4684, 1126.5764, 1151.2418, 1172.6337, 1187.6382, 1188.6071, 1212.6208, 1215.6757, 1221.6454, 1227.6124, 1232.1318, 1234.6710, 1237.6406, 1243.6014, 1246.6598, 1250.6174, 1256.6373, 1277.7306, 1279.5707, 1280.0813, 1304.5978, 1320.5878, 1372.7097, 1404.1747, 1404.6770, 1408.8019, 1429.7334, 1440.7365, 1475.7741, 1531.8497, 1547.8329, 1581.7222, 1649.7094, 1687.9344, 1802.9425, 1817.9724, 1847.8615, 1957.9238, 1993.9932, 2022.0576, 2053.0407, 2056.0529, 2061.0381, 2069.0547, 2094.0682, 2098.0584, 2106.0616, 2118.0725, 2125.0731, 2128.0741, 2135.0518, 2139.0868, 2142.0621, 2145.0668, 2155.0825, 2160.0656, 2171.0780, 2265.0755, 2405.2266, 2417.2380, 2442.0921, 2443.2436, 2455.2338, 2462.2518, 2473.2567, 2476.2685, 2484.2157, 2488.2653, 2501.1605, 2512.1416, 2530.2773, 2539.1490, 2558.1567, 2569.1587, 2574.1447, 2575.3339, 2614.2493, 3348.6481

5. VSPH1_VIPAA **Mass:** 29593 **Score:** 288 **Expect:** 2.2e-026 **Matches:** 8
Vaa serine proteinase homolog 1 OS=Vipera ammodytes ammodytes OX=8705 PE=1 SV=1

Observed	Mr(expt)	Mr(calc)	ppm	Start	End	Miss	Ions	Peptide
866.5652	865.5580	865.5385	22.5	75	82	0	---	R.IILGVHSK.N
888.4497	887.4425	887.4211	24.0	98	104	0	26	K.FFCLSSK.T
1189.6613	1188.6541	1188.6325	18.1	109	117	1	81	R.WDKDIMLIR.L
1205.6521	1204.6448	1204.6274	14.4	109	117	1	(30)	R.WDKDIMLIR.L + Oxidation (M)
1208.6536	1207.6464	1207.6271	15.9	146	156	0	41	R.IMGWGTITTTK.V
1224.6463	1223.6390	1223.6220	13.9	146	156	0	---	R.IMGWGTITTTK.V + Oxidation (M)
1231.6432	1230.6359	1230.5299	86.2	83	92	0	---	K.NVPNEDEQMR.V
2113.0782	2112.0710	2112.0462	11.7	50	67	0	115	R.FYCAGTLINQEWVLTAAR.C

No match to: 855.0839, 869.4391, 871.0597, 914.4659, 1058.5857, 1067.4623, 1077.4684, 1126.5764, 1151.2418, 1172.6337, 1187.6382, 1188.6071, 1212.6208, 1215.6757, 1221.6454, 1227.6124, 1232.1318, 1234.6710, 1237.6406, 1243.6014, 1246.6598, 1250.6174, 1256.6373, 1277.7306, 1279.5707, 1280.0813, 1304.5978, 1320.5878, 1372.7097, 1404.1747, 1404.6770, 1408.8019, 1429.7334, 1440.7365, 1475.7741, 1531.8497, 1547.8329, 1581.7222, 1649.7094, 1687.9344, 1802.9425, 1817.9724, 1847.8615, 1957.9238, 1993.9932, 2022.0576, 2053.0407, 2056.0529, 2061.0381, 2069.0547, 2094.0682, 2098.0584, 2106.0616, 2118.0725, 2125.0731, 2128.0741, 2135.0518, 2139.0868, 2142.0621, 2145.0668, 2155.0825, 2160.0656, 2171.0780, 2265.0755, 2405.2266, 2417.2380, 2442.0921, 2443.2436, 2455.2338, 2462.2518, 2473.2567, 2476.2685, 2484.2157, 2488.2653, 2501.1605, 2512.1416, 2530.2773, 2539.1490, 2558.1567, 2569.1587, 2574.1447, 2575.3339, 2614.2493, 3348.6481

6. A0A6G5ZUW7_9SAUR **Mass:** 22103 **Score:** 166 **Expect:** 3.6e-014 **Matches:** 7
Serine protease 10 (Fragment) OS=Vipera anatolica senliki OX=2604287 PE=2 SV=1

Observed	Mr(expt)	Mr(calc)	ppm	Start	End	Miss	Ions	Peptide
866.5652	865.5580	865.5385	22.5	72	79	0	---	R.IILGVHSK.N
888.4497	887.4425	887.4211	24.0	95	101	0	26	K.FFCLSSK.S
1189.6613	1188.6541	1188.6325	18.1	106	114	1	81	R.WDKDIMLIR.L
1205.6521	1204.6448	1204.6274	14.4	106	114	1	(30)	R.WDKDIMLIR.L + Oxidation (M)
1208.6536	1207.6464	1207.6271	15.9	143	153	0	41	R.IMGWGTITTTK.V
1224.6463	1223.6390	1223.6220	13.9	143	153	0	---	R.IMGWGTITTTK.V + Oxidation (M)
1231.6432	1230.6359	1230.5299	86.2	80	89	0	---	K.NVPNEDEQMR.V

No match to: 855.0839, 869.4391, 871.0597, 914.4659, 1058.5857, 1067.4623, 1077.4684, 1126.5764, 1151.2418, 1172.6337, 1187.6382, 1188.6071, 1212.6208, 1215.6757, 1221.6454, 1227.6124, 1232.1318, 1234.6710, 1237.6406, 1243.6014, 1246.6598, 1250.6174, 1256.6373, 1277.7306, 1279.5707, 1280.0813, 1304.5978, 1320.5878, 1372.7097, 1404.1747, 1404.6770, 1408.8019, 1429.7334, 1440.7365, 1475.7741, 1531.8497, 1547.8329, 1581.7222, 1649.7094, 1687.9344, 1802.9425, 1817.9724, 1847.8615, 1957.9238, 1993.9932, 2022.0576, 2053.0407, 2056.0529, 2061.0381, 2069.0547, 2094.0682, 2098.0584, 2106.0616, 2113.0782, 2118.0725, 2125.0731, 2128.0741, 2135.0518, 2139.0868, 2142.0621, 2145.0668, 2155.0825, 2160.0656, 2171.0780, 2265.0755, 2405.2266, 2417.2380, 2442.0921, 2443.2436, 2455.2338, 2462.2518, 2473.2567, 2476.2685, 2484.2157, 2488.2653, 2501.1605, 2512.1416, 2530.2773, 2539.1490, 2558.1567, 2569.1587, 2574.1447, 2575.3339, 2614.2493, 3348.6481

7. VSPF5_MACLB **Mass:** 29261 **Score:** 134 **Expect:** 5.6e-011 **Matches:** 5
Factor V activator OS=Macrovipera lebetina OX=8709 PE=1 SV=1

Observed	Mr(expt)	Mr(calc)	ppm	Start	End	Miss	Ions	Peptide
1817.9724	1816.9652	1816.9431	12.1	124	141	0	108	K.NSAHIAPISLPSSPSSPR.S
1847.8615	1846.8542	1846.8407	7.30	152	167	0	---	K.ISTTEETYPDVPHCAK.I
1957.9238	1956.9165	1956.8941	11.5	173	188	0	---	K.HAWCEALYPWVPADSR.T
2061.0381	2060.0308	2060.0763	-22.06	124	143	1	---	K.NSAHIAPISLPSSPSSPRSR.C
2558.1567	2557.1494	2557.2940	-56.55	168	188	1	---	K.IFIVKHAWCEALYPWVPADSR.T

No match to: 855.0839, 866.5652, 869.4391, 871.0597, 888.4497, 914.4659, 1058.5857, 1067.4623, 1077.4684, 1126.5764, 1151.2418, 1172.6337, 1187.6382, 1188.6071, 1189.6613, 1205.6521, 1208.6536, 1212.6208, 1215.6757, 1221.6454, 1224.6463, 1227.6124, 1231.6432, 1232.1318, 1234.6710, 1237.6406, 1243.6014, 1246.6598, 1250.6174, 1256.6373, 1277.7306, 1279.5707, 1280.0813, 1304.5978, 1320.5878, 1372.7097, 1404.1747, 1404.6770, 1408.8019, 1429.7334, 1440.7365, 1475.7741, 1531.8497, 1547.8329, 1581.7222, 1649.7094, 1687.9344, 1802.9425, 1993.9932, 2022.0576, 2053.0407, 2056.0529, 2069.0547, 2094.0682, 2098.0584, 2106.0616, 2113.0782, 2118.0725, 2125.0731, 2128.0741, 2135.0518, 2139.0868, 2142.0621, 2145.0668, 2155.0825, 2160.0656, 2171.0780, 2265.0755, 2405.2266, 2417.2380, 2442.0921, 2443.2436, 2455.2338, 2462.2518, 2473.2567, 2476.2685, 2484.2157, 2488.2653, 2501.1605, 2512.1416, 2530.2773, 2539.1490, 2569.1587, 2574.1447, 2575.3339, 2614.2493, 3348.6481

8. A0A6B7FMQ3_VIPAA **Mass:** 28909 **Score:** 110 **Expect:** 1.4e-008 **Matches:** 3
Snake venom serine protease OS=Vipera ammodytes ammodytes OX=8705 PE=2 SV=1

Observed	Mr(expt)	Mr(calc)	ppm	Start	End	Miss	Ions	Peptide
888.4497	887.4425	887.4211	24.0	95	101	0	26	K.FFCLSSK.N
1189.6613	1188.6541	1188.6325	18.1	106	114	1	81	K.WDKDIMLIR.L
1205.6521	1204.6448	1204.6274	14.4	106	114	1	(30)	K.WDKDIMLIR.L + Oxidation (M)

No match to: 855.0839, 866.5652, 869.4391, 871.0597, 914.4659, 1058.5857, 1067.4623, 1077.4684, 1126.5764, 1151.2418, 1172.6337, 1187.6382, 1188.6071, 1208.6536, 1212.6208, 1215.6757, 1221.6454, 1224.6463, 1227.6124, 1231.6432, 1232.1318, 1234.6710, 1237.6406, 1243.6014, 1246.6598, 1250.6174, 1256.6373, 1277.7306, 1279.5707, 1280.0813, 1304.5978, 1320.5878, 1372.7097, 1404.1747, 1404.6770, 1408.8019, 1429.7334, 1440.7365, 1475.7741, 1531.8497, 1547.8329, 1581.7222, 1649.7094, 1687.9344, 1802.9425, 1817.9724, 1847.8615, 1957.9238, 1993.9932, 2022.0576, 2053.0407, 2056.0529, 2061.0381, 2069.0547, 2094.0682, 2098.0584, 2106.0616, 2113.0782, 2118.0725, 2125.0731, 2128.0741, 2135.0518, 2139.0868, 2142.0621, 2145.0668, 2155.0825, 2160.0656, 2171.0780, 2265.0755, 2405.2266, 2417.2380, 2442.0921, 2443.2436, 2455.2338, 2462.2518, 2473.2567, 2476.2685, 2484.2157, 2488.2653, 2501.1605, 2512.1416, 2530.2773, 2539.1490, 2558.1567, 2569.1587, 2574.1447, 2575.3339, 2614.2493, 3348.6481

9. D8MIA0_BITRH **Mass:** 29517 **Score:** 86 **Expect:** 3.3e-006 **Matches:** 3
Rhinocerase 2 protein (Fragment) OS=Bitis rhinoceros OX=715877 GN=rhinocerase 2 PE=3 SV=1

Observed	Mr(expt)	Mr(calc)	ppm	Start	End	Miss	Ions	Peptide
1189.6613	1188.6541	1188.6325	18.1	108	116	1	81	R.WDKDIMLIR.L
1205.6521	1204.6448	1204.6274	14.4	108	116	1	(30)	R.WDKDIMLIR.L + Oxidation (M)
1279.5707	1278.5634	1278.5146	38.2	82	91	0	---	K.NVQNEDEEMR.V + Oxidation (M)

No match to: 855.0839, 866.5652, 869.4391, 871.0597, 888.4497, 914.4659, 1058.5857, 1067.4623, 1077.4684, 1126.5764, 1151.2418, 1172.6337, 1187.6382, 1188.6071, 1208.6536, 1212.6208, 1215.6757, 1221.6454, 1224.6463, 1227.6124, 1231.6432, 1232.1318, 1234.6710, 1237.6406, 1243.6014, 1246.6598, 1250.6174, 1256.6373, 1277.7306, 1280.0813, 1304.5978, 1320.5878, 1372.7097, 1404.1747, 1404.6770, 1408.8019, 1429.7334, 1440.7365, 1475.7741, 1531.8497, 1547.8329, 1581.7222, 1649.7094, 1687.9344, 1802.9425, 1817.9724, 1847.8615, 1957.9238, 1993.9932, 2022.0576, 2053.0407, 2056.0529, 2061.0381, 2069.0547, 2094.0682, 2098.0584, 2106.0616, 2113.0782, 2118.0725, 2125.0731, 2128.0741, 2135.0518, 2139.0868, 2142.0621, 2145.0668, 2155.0825, 2160.0656, 2171.0780, 2265.0755, 2405.2266, 2417.2380, 2442.0921, 2443.2436, 2455.2338, 2462.2518, 2473.2567, 2476.2685, 2484.2157, 2488.2653, 2501.1605, 2512.1416, 2530.2773, 2539.1490, 2558.1567, 2569.1587, 2574.1447, 2575.3339, 2614.2493, 3348.6481

10. D8MIA1_BITRH **Mass:** 29504 **Score:** 81 **Expect:** 1.2e-005 **Matches:** 2
Rhinocerase 3 protein (Fragment) OS=Bitis rhinoceros OX=715877 GN=rhinocerase 3 PE=3 SV=1

Observed	Mr(expt)	Mr(calc)	ppm	Start	End	Miss	Ions	Peptide
1189.6613	1188.6541	1188.6325	18.1	108	116	1	81	R.WDKDIMLIR.L
1205.6521	1204.6448	1204.6274	14.4	108	116	1	(30)	R.WDKDIMLIR.L + Oxidation (M)

No match to: 855.0839, 866.5652, 869.4391, 871.0597, 888.4497, 914.4659, 1058.5857, 1067.4623, 1077.4684, 1126.5764, 1151.2418, 1172.6337, 1187.6382, 1188.6071, 1208.6536, 1212.6208, 1215.6757, 1221.6454, 1224.6463, 1227.6124, 1231.6432, 1232.1318, 1234.6710, 1237.6406, 1243.6014, 1246.6598, 1250.6174, 1256.6373, 1277.7306, 1279.5707, 1280.0813, 1304.5978, 1320.5878, 1372.7097, 1404.1747, 1404.6770, 1408.8019, 1429.7334, 1440.7365, 1475.7741, 1531.8497, 1547.8329, 1581.7222, 1649.7094, 1687.9344, 1802.9425, 1817.9724, 1847.8615, 1957.9238, 1993.9932, 2022.0576, 2053.0407, 2056.0529, 2061.0381, 2069.0547, 2094.0682, 2098.0584, 2106.0616, 2113.0782,

2118.0725, 2125.0731, 2128.0741, 2135.0518, 2139.0868, 2142.0621, 2145.0668, 2155.0825, 2160.0656, 2171.0780, 2265.0755, 2405.2266, 2417.2380, 2442.0921, 2443.2436, 2455.2338, 2462.2518, 2473.2567, 2476.2685, 2484.2157, 2488.2653, 2501.1605, 2512.1416, 2530.2773, 2539.1490, 2558.1567, 2569.1587, 2574.1447, 2575.3339, 2614.2493, 3348.6481

11. VSPY_MACLB **Mass:** 28944 **Score:** 78 **Expect:** 2e-005 **Matches:** 2

Chymotrypsin-like protease VLCTLP OS=Macrovipera lebetina OX=8709 PE=1 SV=1

Observed	Mr(expt)	Mr(calc)	ppm	Start	End	Miss	Ions	Peptide
1189.6613	1188.6541	1188.6325	18.1	106 -	114	1	81	K.WDKDIMLIR.L
1205.6521	1204.6448	1204.6274	14.4	106 -	114	1	(30)	K.WDKDIMLIR.L + Oxidation (M)

No match to: 855.0839, 866.5652, 869.4391, 871.0597, 888.4497, 914.4659, 1058.5857, 1067.4623, 1077.4684, 1126.5764, 1151.2418, 1172.6337, 1187.6382, 1188.6071, 1208.6536, 1212.6208, 1215.6757, 1221.6454, 1224.6463, 1227.6124, 1231.6432, 1232.1318, 1234.6710, 1237.6406, 1243.6014, 1246.6598, 1250.6174, 1256.6373, 1277.7306, 1279.5707, 1280.0813, 1304.5978, 1320.5878, 1372.7097, 1404.1747, 1404.6770, 1408.8019, 1429.7334, 1440.7365, 1475.7741, 1531.8497, 1547.8329, 1581.7222, 1649.7094, 1687.9344, 1802.9425, 1817.9724, 1847.8615, 1957.9238, 1993.9932, 2022.0576, 2053.0407, 2056.0529, 2061.0381, 2069.0547, 2094.0682, 2098.0584, 2106.0616, 2113.0782, 2118.0725, 2125.0731, 2128.0741, 2135.0518, 2139.0868, 2142.0621, 2145.0668, 2155.0825, 2160.0656, 2171.0780, 2265.0755, 2405.2266, 2417.2380, 2442.0921, 2443.2436, 2455.2338, 2462.2518, 2473.2567, 2476.2685, 2484.2157, 2488.2653, 2501.1605, 2512.1416, 2530.2773, 2539.1490, 2558.1567, 2569.1587, 2574.1447, 2575.3339, 2614.2493, 3348.6481

12. VSPBF_MACLB **Mass:** 28963 **Score:** 32 **Expect:** 0.85 **Matches:** 2

Beta-fibrinogenase OS=Macrovipera lebetina OX=8709 PE=1 SV=1

Observed	Mr(expt)	Mr(calc)	ppm	Start	End	Miss	Ions	Peptide
888.4497	887.4425	887.4211	24.0	95 -	101	0	26	K.FFCLSSK.N
2118.0725	2117.0652	2117.0629	1.09	72 -	88	1	---	R.IYLGWHNFSLPNMNQKR.R

No match to: 855.0839, 866.5652, 869.4391, 871.0597, 914.4659, 1058.5857, 1067.4623, 1077.4684, 1126.5764, 1151.2418, 1172.6337, 1187.6382, 1188.6071, 1189.6613, 1205.6521, 1208.6536, 1212.6208, 1215.6757, 1221.6454, 1224.6463, 1227.6124, 1231.6432, 1232.1318, 1234.6710, 1237.6406, 1243.6014, 1246.6598, 1250.6174, 1256.6373, 1277.7306, 1279.5707, 1280.0813, 1304.5978, 1320.5878, 1372.7097, 1404.1747, 1404.6770, 1408.8019, 1429.7334, 1440.7365, 1475.7741, 1531.8497, 1547.8329, 1581.7222, 1649.7094, 1687.9344, 1802.9425, 1817.9724, 1847.8615, 1957.9238, 1993.9932, 2022.0576, 2053.0407, 2056.0529, 2061.0381, 2069.0547, 2094.0682, 2098.0584, 2106.0616, 2113.0782, 2125.0731, 2128.0741, 2135.0518, 2139.0868, 2142.0621, 2145.0668, 2155.0825, 2160.0656, 2171.0780, 2265.0755, 2405.2266, 2417.2380, 2442.0921, 2443.2436, 2455.2338, 2462.2518, 2473.2567, 2476.2685, 2484.2157, 2488.2653, 2501.1605, 2512.1416, 2530.2773, 2539.1490, 2558.1567, 2569.1587, 2574.1447, 2575.3339, 2614.2493, 3348.6481

13. A0A0H3Y7H2_9PERO **Mass:** 22231 **Score:** 29 **Expect:** 1.9 **Matches:** 5

Uncharacterized protein (Fragment) OS=Echiichthys vipera OX=94984 GN=ptr PE=4 SV=1

Observed	Mr(expt)	Mr(calc)	ppm	Start	End	Miss	Ions	Peptide
1227.6124	1226.6051	1226.6329	-22.67	128 -	137	1	---	R.MYLVARTTEK.K + Oxidation (M)
1243.6014	1242.5941	1242.6819	-70.70	139 -	148	1	---	K.REEVVELLEK.L
1581.7222	1580.7149	1580.7430	-17.77	115 -	127	1	---	K.NRETDEYDIIASR.M
2061.0381	2060.0308	2059.9884	20.6	117 -	133	1	---	R.ETDEYDIIASRMYLVAR.T + Oxidation (M)
2530.2773	2529.2700	2529.2475	8.90	66 -	86	1	---	K.ISWMDNFFHYLRVVNVSASTK.S + Oxidation (M)

No match to: 855.0839, 866.5652, 869.4391, 871.0597, 888.4497, 914.4659, 1058.5857, 1067.4623, 1077.4684, 1126.5764, 1151.2418, 1172.6337, 1187.6382, 1188.6071, 1189.6613, 1205.6521, 1208.6536, 1212.6208, 1215.6757, 1221.6454, 1224.6463, 1231.6432, 1232.1318, 1234.6710, 1237.6406, 1246.6598, 1250.6174, 1256.6373, 1277.7306, 1279.5707, 1280.0813, 1304.5978, 1320.5878, 1372.7097, 1404.1747, 1404.6770, 1408.8019, 1429.7334, 1440.7365, 1475.7741, 1531.8497, 1547.8329, 1649.7094, 1687.9344, 1802.9425, 1817.9724, 1847.8615, 1957.9238, 1993.9932, 2022.0576, 2053.0407, 2056.0529, 2069.0547, 2094.0682, 2098.0584, 2106.0616, 2113.0782, 2118.0725, 2125.0731, 2128.0741, 2135.0518, 2139.0868, 2142.0621, 2145.0668, 2155.0825, 2160.0656, 2171.0780, 2265.0755, 2405.2266, 2417.2380, 2442.0921, 2443.2436, 2455.2338, 2462.2518, 2473.2567, 2476.2685, 2484.2157, 2488.2653, 2501.1605, 2512.1416, 2539.1490, 2558.1567, 2569.1587, 2574.1447, 2575.3339, 2614.2493, 3348.6481

14. A0A1I9KNR8_VIPAA **Mass:** 29252 **Score:** 26 **Expect:** 3.6 **Matches:** 1

Snake venom serine protease OS=Vipera ammodytes ammodytes OX=8705 PE=2 SV=1

Observed	Mr(expt)	Mr(calc)	ppm	Start	End	Miss	Ions	Peptide
888.4497	887.4425	887.4211	24.0	98 -	104	0	26	K.FFCLSSK.S

No match to: 855.0839, 866.5652, 869.4391, 871.0597, 914.4659, 1058.5857, 1067.4623, 1077.4684, 1126.5764, 1151.2418, 1172.6337, 1187.6382, 1188.6071, 1189.6613, 1205.6521, 1208.6536, 1212.6208, 1215.6757, 1221.6454, 1224.6463, 1227.6124, 1231.6432, 1232.1318, 1234.6710, 1237.6406, 1243.6014, 1246.6598, 1250.6174, 1256.6373, 1277.7306, 1279.5707, 1280.0813, 1304.5978, 1320.5878, 1372.7097, 1404.1747, 1404.6770, 1408.8019, 1429.7334, 1440.7365, 1475.7741, 1531.8497, 1547.8329, 1581.7222, 1649.7094, 1687.9344, 1802.9425, 1817.9724, 1847.8615, 1957.9238, 1993.9932, 2022.0576, 2053.0407, 2056.0529, 2061.0381, 2069.0547, 2094.0682, 2098.0584, 2106.0616, 2113.0782, 2118.0725, 2125.0731, 2128.0741, 2135.0518, 2139.0868, 2142.0621, 2145.0668, 2155.0825, 2160.0656, 2171.0780, 2265.0755, 2405.2266, 2417.2380, 2442.0921, 2443.2436, 2455.2338, 2462.2518, 2473.2567, 2476.2685, 2484.2157, 2488.2653, 2501.1605, 2512.1416, 2530.2773, 2539.1490, 2558.1567, 2569.1587, 2574.1447, 2575.3339, 2614.2493, 3348.6481

15. A0A6B7FQF8_VIPAA **Mass:** 28983 **Score:** 26 **Expect:** 3.6 **Matches:** 1

Serine proteinase SP-6 OS=Vipera ammodytes ammodytes OX=8705 PE=2 SV=1

Observed	Mr(expt)	Mr(calc)	ppm	Start	End	Miss	Ions	Peptide
888.4497	887.4425	887.4211	24.0	95 -	101	0	26	K.FFCLSSK.N

No match to: 855.0839, 866.5652, 869.4391, 871.0597, 914.4659, 1058.5857, 1067.4623, 1077.4684, 1126.5764, 1151.2418, 1172.6337, 1187.6382, 1188.6071, 1189.6613, 1205.6521, 1208.6536, 1212.6208, 1215.6757, 1221.6454, 1224.6463, 1227.6124, 1231.6432, 1232.1318, 1234.6710, 1237.6406, 1243.6014, 1246.6598, 1250.6174, 1256.6373, 1277.7306, 1279.5707, 1280.0813, 1304.5978, 1320.5878, 1372.7097, 1404.1747, 1404.6770, 1408.8019, 1429.7334, 1440.7365, 1475.7741, 1531.8497, 1547.8329, 1581.7222, 1649.7094, 1687.9344, 1802.9425, 1817.9724, 1847.8615, 1957.9238, 1993.9932, 2022.0576, 2053.0407, 2056.0529, 2061.0381, 2069.0547, 2094.0682, 2098.0584, 2106.0616, 2113.0782, 2118.0725, 2125.0731, 2128.0741, 2135.0518, 2139.0868, 2142.0621, 2145.0668, 2155.0825, 2160.0656, 2171.0780, 2265.0755, 2405.2266, 2417.2380, 2442.0921, 2443.2436, 2455.2338, 2462.2518, 2473.2567, 2476.2685, 2484.2157, 2488.2653, 2501.1605, 2512.1416, 2530.2773, 2539.1490, 2558.1567, 2569.1587, 2574.1447, 2575.3339, 2614.2493, 3348.6481

16. A0A6G5ZW78_9SAUR **Mass:** 28826 **Score:** 26 **Expect:** 3.6 **Matches:** 1

Serine protease 2 OS=Vipera anatolica senliki OX=2604287 PE=2 SV=1

Observed	Mr(expt)	Mr(calc)	ppm	Start	End	Miss	Ions	Peptide
888.4497	887.4425	887.4211	24.0	95 -	101	0	26	K.FFCLSSK.E

No match to: 855.0839, 866.5652, 869.4391, 871.0597, 914.4659, 1058.5857, 1067.4623, 1077.4684, 1126.5764, 1151.2418, 1172.6337, 1187.6382, 1188.6071, 1189.6613, 1205.6521, 1208.6536, 1212.6208, 1215.6757, 1221.6454, 1224.6463, 1227.6124, 1231.6432, 1232.1318, 1234.6710, 1237.6406, 1243.6014, 1246.6598, 1250.6174, 1256.6373, 1277.7306, 1279.5707, 1280.0813, 1304.5978, 1320.5878, 1372.7097, 1404.1747, 1404.6770, 1408.8019, 1429.7334, 1440.7365, 1475.7741, 1531.8497, 1547.8329, 1581.7222, 1649.7094, 1687.9344, 1802.9425, 1817.9724, 1847.8615, 1957.9238, 1993.9932, 2022.0576, 2053.0407, 2056.0529, 2061.0381, 2069.0547, 2094.0682, 2098.0584, 2106.0616, 2113.0782, 2118.0725, 2125.0731, 2128.0741, 2135.0518, 2139.0868, 2142.0621, 2145.0668, 2155.0825, 2160.0656, 2171.0780, 2265.0755, 2405.2266, 2417.2380, 2442.0921, 2443.2436, 2455.2338, 2462.2518, 2473.2567, 2476.2685, 2484.2157, 2488.2653, 2501.1605, 2512.1416, 2530.2773, 2539.1490, 2558.1567, 2569.1587, 2574.1447, 2575.3339, 2614.2493, 3348.6481

17. VSP3_MACLB **Mass:** 29017 **Score:** 26 **Expect:** 3.6 **Matches:** 1

Serine protease VLSP-3 OS=Macrovipera lebetina OX=8709 PE=2 SV=1

Observed	Mr(expt)	Mr(calc)	ppm	Start	End	Miss	Ions	Peptide
888.4497	887.4425	887.4211	24.0	96 -	102	0	26	K.FFCLSSK.N

No match to: 855.0839, 866.5652, 869.4391, 871.0597, 914.4659, 1058.5857, 1067.4623, 1077.4684, 1126.5764, 1151.2418, 1172.6337, 1187.6382, 1188.6071, 1189.6613, 1205.6521, 1208.6536, 1212.6208, 1215.6757, 1221.6454, 1224.6463, 1227.6124, 1231.6432, 1232.1318, 1234.6710, 1237.6406, 1243.6014, 1246.6598, 1250.6174, 1256.6373, 1277.7306, 1279.5707, 1280.0813, 1304.5978, 1320.5878, 1372.7097, 1404.1747, 1404.6770, 1408.8019, 1429.7334, 1440.7365, 1475.7741, 1531.8497, 1547.8329, 1581.7222, 1649.7094, 1687.9344, 1802.9425, 1817.9724, 1847.8615, 1957.9238, 1993.9932, 2022.0576, 2053.0407, 2056.0529, 2061.0381, 2069.0547, 2094.0682, 2098.0584, 2106.0616, 2113.0782, 2118.0725, 2125.0731, 2128.0741, 2135.0518, 2139.0868, 2142.0621, 2145.0668, 2155.0825, 2160.0656, 2171.0780, 2265.0755, 2405.2266, 2417.2380, 2442.0921, 2443.2436, 2455.2338, 2462.2518, 2473.2567, 2476.2685, 2484.2157, 2488.2653, 2501.1605, 2512.1416,

2530.2773, 2539.1490, 2558.1567, 2569.1587, 2574.1447, 2575.3339, 2614.2493, 3348.6481

18. VSP_VIPBN **Mass:** 28882 **Score:** 26 **Expect:** 3.6 **Matches:** 1
Snake venom serine protease nikobin OS=Vipera berus nikolskii OX=1808362 GN=sp-VN PE=2 SV=1

Observed	Mr(expt)	Mr(calc)	ppm	Start	End	Miss	Ions	Peptide
888.4497	887.4425	887.4211	24.0	95	101	0	26	K.FFCLSSK.E

No match to: 855.0839, 866.5652, 869.4391, 871.0597, 914.4659, 1058.5857, 1067.4623, 1077.4684, 1126.5764, 1151.2418, 1172.6337, 1187.6382, 1188.6071, 1189.6613, 1205.6521, 1208.6536, 1212.6208, 1215.6757, 1221.6454, 1224.6463, 1227.6124, 1231.6432, 1232.1318, 1234.6710, 1237.6406, 1243.6014, 1246.6598, 1250.6174, 1256.6373, 1277.7306, 1279.5707, 1280.0813, 1304.5978, 1320.5878, 1372.7097, 1404.1747, 1404.6770, 1408.8019, 1429.7334, 1440.7365, 1475.7741, 1531.8497, 1547.8329, 1581.7222, 1649.7094, 1687.9344, 1802.9425, 1817.9724, 1847.8615, 1957.9238, 1993.9932, 2022.0576, 2053.0407, 2056.0529, 2061.0381, 2069.0547, 2094.0682, 2098.0584, 2106.0616, 2113.0782, 2118.0725, 2125.0731, 2128.0741, 2135.0518, 2139.0868, 2142.0621, 2145.0668, 2155.0825, 2160.0656, 2171.0780, 2265.0755, 2405.2266, 2417.2380, 2442.0921, 2443.2436, 2455.2338, 2462.2518, 2473.2567, 2476.2685, 2484.2157, 2488.2653, 2501.1605, 2512.1416, 2530.2773, 2539.1490, 2558.1567, 2569.1587, 2574.1447, 2575.3339, 2614.2493, 3348.6481

19. A0A6G5ZW27_9SAUR **Mass:** 38471 **Score:** 23 **Expect:** 6.8 **Matches:** 6
Venom factor 6 (Fragment) OS=Vipera anatolica senliki OX=2604287 PE=2 SV=1

Observed	Mr(expt)	Mr(calc)	ppm	Start	End	Miss	Ions	Peptide
1212.6208	1211.6135	1211.6550	-34.25	140	149	1	---	K.AVYVLNDKYK.I
1243.6014	1242.5941	1242.5775	13.3	197	207	1	---	R.SDAKCPQPANR.R
1304.5978	1303.5906	1303.6884	-75.08	223	233	1	---	K.VAEFQDQALRK.C
2442.0921	2441.0848	2441.3101	-92.26	51	72	1	---	R.GPGQNLVTMTLRITPDLIPSFR.F + Oxidation (M)
2476.2685	2475.2612	2475.1021	64.3	254	273	1	---	K.YIQEGDACKAAFLECCHYIK.G
2488.2653	2487.2580	2487.2322	10.4	73	94	0	---	R.FVAYYQVGNSEIVADSVWVDVK.D

No match to: 855.0839, 866.5652, 869.4391, 871.0597, 888.4497, 914.4659, 1058.5857, 1067.4623, 1077.4684, 1126.5764, 1151.2418, 1172.6337, 1187.6382, 1188.6071, 1189.6613, 1205.6521, 1208.6536, 1215.6757, 1221.6454, 1224.6463, 1227.6124, 1231.6432, 1232.1318, 1234.6710, 1237.6406, 1246.6598, 1250.6174, 1256.6373, 1277.7306, 1279.5707, 1280.0813, 1320.5878, 1372.7097, 1404.1747, 1404.6770, 1408.8019, 1429.7334, 1440.7365, 1475.7741, 1531.8497, 1547.8329, 1581.7222, 1649.7094, 1687.9344, 1802.9425, 1817.9724, 1847.8615, 1957.9238, 1993.9932, 2022.0576, 2053.0407, 2056.0529, 2061.0381, 2069.0547, 2094.0682, 2098.0584, 2106.0616, 2113.0782, 2118.0725, 2125.0731, 2128.0741, 2135.0518, 2139.0868, 2142.0621, 2145.0668, 2155.0825, 2160.0656, 2171.0780, 2265.0755, 2405.2266, 2417.2380, 2443.2436, 2455.2338, 2462.2518, 2473.2567, 2484.2157, 2501.1605, 2512.1416, 2530.2773, 2539.1490, 2558.1567, 2569.1587, 2574.1447, 2575.3339, 2614.2493, 3348.6481

20. A0A6G5ZUA3_9SAUR **Mass:** 25767 **Score:** 20 **Expect:** 13 **Matches:** 4
Cysteine-rich secretory protein 1 (Fragment) OS=Vipera anatolica senliki OX=2604287 PE=2 SV=1

Observed	Mr(expt)	Mr(calc)	ppm	Start	End	Miss	Ions	Peptide
1404.6770	1403.6697	1403.6364	23.7	75	85	1	---	R.CIYDHSPRNSR.V
2098.0584	2097.0511	2096.9526	47.0	58	74	1	---	K.MEWYPEAAANAERWAFR.C
2118.0725	2117.0652	2116.9479	55.4	86	104	0	---	R.VIGGIECGENIYMSTSPMK.W + 2 Oxidation (M)
2443.2436	2442.2363	2442.1341	41.8	83	104	1	---	R.NSRVIGGIECGENIYMSTSPMK.W

No match to: 855.0839, 866.5652, 869.4391, 871.0597, 888.4497, 914.4659, 1058.5857, 1067.4623, 1077.4684, 1126.5764, 1151.2418, 1172.6337, 1187.6382, 1188.6071, 1189.6613, 1205.6521, 1208.6536, 1212.6208, 1215.6757, 1221.6454, 1224.6463, 1227.6124, 1231.6432, 1232.1318, 1234.6710, 1237.6406, 1243.6014, 1246.6598, 1250.6174, 1256.6373, 1277.7306, 1279.5707, 1280.0813, 1304.5978, 1320.5878, 1372.7097, 1404.1747, 1408.8019, 1429.7334, 1440.7365, 1475.7741, 1531.8497, 1547.8329, 1581.7222, 1649.7094, 1687.9344, 1802.9425, 1817.9724, 1847.8615, 1957.9238, 1993.9932, 2022.0576, 2053.0407, 2056.0529, 2061.0381, 2069.0547, 2094.0682, 2106.0616, 2113.0782, 2125.0731, 2128.0741, 2135.0518, 2139.0868, 2142.0621, 2145.0668, 2155.0825, 2160.0656, 2171.0780, 2265.0755, 2405.2266, 2417.2380, 2442.0921, 2455.2338, 2462.2518, 2473.2567, 2476.2685, 2484.2157, 2488.2653, 2501.1605, 2512.1416, 2530.2773, 2539.1490, 2558.1567, 2569.1587, 2574.1447, 2575.3339, 2614.2493, 3348.6481

21. A0A6G5ZVV8_9SAUR **Mass:** 89505 **Score:** 20 **Expect:** 14 **Matches:** 7
Dipeptidylpeptidase 1 (Fragment) OS=Vipera anatolica senliki OX=2604287 PE=2 SV=1

Observed	Mr(expt)	Mr(calc)	ppm	Start	End	Miss	Ions	Peptide
1077.4684	1076.4611	1076.5138	-48.95	352	360	0	---	K.QNEEPVFSK.D
1189.6613	1188.6541	1188.6615	-6.28	307	316	1	7	K.WVSNTKTVVR.W
1212.6208	1211.6135	1211.6258	-10.17	1	11	1	---	-.KELGSNSPPQR.N
1250.6174	1249.6101	1249.6415	-25.13	108	118	1	---	K.ASRYSVSPDLR.Y
1372.7097	1371.7024	1371.6711	22.9	341	351	0	---	K.YEFTSEVWLAK.Q
1581.7222	1580.7149	1580.8311	-73.47	618	631	1	---	K.QSFIDGNRLSIFGK.G
2069.0547	2068.0474	2068.0425	2.37	373	390	1	---	K.QGGRGEFHHIAMFNVQIK.N

No match to: 855.0839, 866.5652, 869.4391, 871.0597, 888.4497, 914.4659, 1058.5857, 1067.4623, 1126.5764, 1151.2418, 1172.6337, 1187.6382, 1188.6071, 1205.6521, 1208.6536, 1215.6757, 1221.6454, 1224.6463, 1227.6124, 1231.6432, 1232.1318, 1234.6710, 1237.6406, 1243.6014, 1246.6598, 1256.6373, 1277.7306, 1279.5707, 1280.0813, 1304.5978, 1320.5878, 1404.1747, 1404.6770, 1408.8019, 1429.7334, 1440.7365, 1475.7741, 1531.8497, 1547.8329, 1649.7094, 1687.9344, 1802.9425, 1817.9724, 1847.8615, 1957.9238, 1993.9932, 2022.0576, 2053.0407, 2056.0529, 2061.0381, 2094.0682, 2098.0584, 2106.0616, 2113.0782, 2118.0725, 2125.0731, 2128.0741, 2135.0518, 2139.0868, 2142.0621, 2145.0668, 2155.0825, 2160.0656, 2171.0780, 2265.0755, 2405.2266, 2417.2380, 2442.0921, 2443.2436, 2455.2338, 2462.2518, 2473.2567, 2476.2685, 2484.2157, 2488.2653, 2501.1605, 2512.1416, 2530.2773, 2539.1490, 2558.1567, 2569.1587, 2574.1447, 2575.3339, 2614.2493, 3348.6481

22. A0A0H3Y6S3_9PERO **Mass:** 29853 **Score:** 20 **Expect:** 15 **Matches:** 6
Glycotransferase (Fragment) OS=Echiichthys vipera OX=94984 GN=glyt PE=3 SV=1

Observed	Mr(expt)	Mr(calc)	ppm	Start	End	Miss	Ions	Peptide
1212.6208	1211.6135	1211.5652	39.9	180	188	1	---	K.DVPRHLCCR.N
1234.6710	1233.6637	1233.5911	58.9	13	23	0	---	K.MNITGVDEVEK.D
1243.6014	1242.5941	1242.6397	-36.72	252	261	0	---	K.LTVSWQIPWN.-
1250.6174	1249.6101	1249.5860	19.3	13	23	0	---	K.MNITGVDEVEK.D + Oxidation (M)
1687.9344	1686.9272	1686.7871	83.0	184	195	1	---	R.HLCCRNPEWLFR.I
2265.0755	2264.0682	2264.1544	-38.09	49	67	0	---	R.LILNEAELIMALAQEFQMR.V + 2 Oxidation (M)

No match to: 855.0839, 866.5652, 869.4391, 871.0597, 888.4497, 914.4659, 1058.5857, 1067.4623, 1077.4684, 1126.5764, 1151.2418, 1172.6337, 1187.6382, 1188.6071, 1189.6613, 1205.6521, 1208.6536, 1215.6757, 1221.6454, 1224.6463, 1227.6124, 1231.6432, 1232.1318, 1237.6406, 1246.6598, 1256.6373, 1277.7306, 1279.5707, 1280.0813, 1304.5978, 1320.5878, 1372.7097, 1404.1747, 1404.6770, 1408.8019, 1429.7334, 1440.7365, 1475.7741, 1531.8497, 1547.8329, 1581.7222, 1649.7094, 1802.9425, 1817.9724, 1847.8615, 1957.9238, 1993.9932, 2022.0576, 2053.0407, 2056.0529, 2061.0381, 2069.0547, 2094.0682, 2098.0584, 2106.0616, 2113.0782, 2118.0725, 2125.0731, 2128.0741, 2135.0518, 2139.0868, 2142.0621, 2145.0668, 2155.0825, 2160.0656, 2171.0780, 2405.2266, 2417.2380, 2442.0921, 2443.2436, 2455.2338, 2462.2518, 2473.2567, 2476.2685, 2484.2157, 2488.2653, 2501.1605, 2512.1416, 2530.2773, 2539.1490, 2558.1567, 2569.1587, 2574.1447, 2575.3339, 2614.2493, 3348.6481

23. A0A6G5ZUS6_9SAUR **Mass:** 13128 **Score:** 20 **Expect:** 15 **Matches:** 3
Coagulation factor V 5 (Fragment) OS=Vipera anatolica senliki OX=2604287 PE=2 SV=1

Observed	Mr(expt)	Mr(calc)	ppm	Start	End	Miss	Ions	Peptide
1188.6071	1187.5998	1187.5611	32.6	58	66	1	---	R.SWYFEKSNK.R
1957.9238	1956.9165	1956.9152	0.69	1	16	1	---	-.QICRSWAYYSGVNPEK.D
2530.2773	2529.2700	2529.1926	30.6	45	63	1	---	R.EFVLLFMVFDEERSWYFEK.S + Oxidation (M)

No match to: 855.0839, 866.5652, 869.4391, 871.0597, 888.4497, 914.4659, 1058.5857, 1067.4623, 1077.4684, 1126.5764, 1151.2418, 1172.6337, 1187.6382, 1189.6613, 1205.6521, 1208.6536, 1212.6208, 1215.6757, 1221.6454, 1224.6463, 1227.6124, 1231.6432, 1232.1318, 1234.6710, 1237.6406, 1243.6014, 1246.6598, 1250.6174, 1256.6373, 1277.7306, 1279.5707, 1280.0813, 1304.5978, 1320.5878, 1372.7097, 1404.1747, 1404.6770, 1408.8019, 1429.7334, 1440.7365, 1475.7741, 1531.8497, 1547.8329, 1581.7222, 1649.7094, 1687.9344, 1802.9425,

1817.9724, 1847.8615, 1993.9932, 2022.0576, 2053.0407, 2056.0529, 2061.0381, 2069.0547, 2094.0682, 2098.0584, 2106.0616, 2113.0782, 2118.0725, 2125.0731, 2128.0741, 2135.0518, 2139.0868, 2142.0621, 2145.0668, 2155.0825, 2160.0656, 2171.0780, 2265.0755, 2405.2266, 2417.2380, 2442.0921, 2443.2436, 2455.2338, 2462.2518, 2473.2567, 2476.2685, 2484.2157, 2488.2653, 2501.1605, 2512.1416, 2539.1490, 2558.1567, 2569.1587, 2574.1447, 2575.3339, 2614.2493, 3348.6481

24. A0A6G5ZUQ0_9SAUR **Mass:** 27438 **Score:** 19 **Expect:** 16 **Matches:** 4

Putative serine carboxypeptidase 1 (Fragment) OS=Vipera anatolica senliki OX=2604287 PE=2 SV=1

Observed	Mr(expt)	Mr(calc)	ppm	Start	End	Miss	Ions	Peptide
1475.7741	1474.7668	1474.6618	71.2	215	226	1	---	R.ALDMMDRFIMGK.G + 3 Oxidation (M)
2053.0407	2052.0334	2052.0276	2.84	22	38	0	---	R.IYVQNQSDLGQQYIQQK.K
2098.0584	2097.0511	2097.1834	-63.06	137	155	0	---	R.VLLYNGQLDIIVAAPLTER.F
2160.0656	2159.0583	2159.0609	-1.18	1	19	0	---	-.QMLGGYADFLYQIGLVDEK.Q

No match to: 855.0839, 866.5652, 869.4391, 871.0597, 888.4497, 914.4659, 1058.5857, 1067.4623, 1077.4684, 1126.5764, 1151.2418, 1172.6337, 1187.6382, 1188.6071, 1189.6613, 1205.6521, 1208.6536, 1212.6208, 1215.6757, 1221.6454, 1224.6463, 1227.6124, 1231.6432, 1232.1318, 1234.6710, 1237.6406, 1243.6014, 1246.6598, 1250.6174, 1256.6373, 1277.7306, 1279.5707, 1280.0813, 1304.5978, 1320.5878, 1372.7097, 1404.1747, 1404.6770, 1408.8019, 1429.7334, 1440.7365, 1531.8497, 1547.8329, 1581.7222, 1649.7094, 1687.9344, 1802.9425, 1817.9724, 1847.8615, 1957.9238, 1993.9932, 2022.0576, 2056.0529, 2061.0381, 2069.0547, 2094.0682, 2106.0616, 2113.0782, 2118.0725, 2125.0731, 2128.0741, 2135.0518, 2139.0868, 2142.0621, 2145.0668, 2155.0825, 2171.0780, 2265.0755, 2405.2266, 2417.2380, 2442.0921, 2443.2436, 2455.2338, 2462.2518, 2473.2567, 2476.2685, 2484.2157, 2488.2653, 2501.1605, 2512.1416, 2530.2773, 2539.1490, 2558.1567, 2569.1587, 2574.1447, 2575.3339, 2614.2493, 3348.6481

25. PA2AB_DABSI **Mass:** 15673 **Score:** 19 **Expect:** 18 **Matches:** 3

Acidic phospholipase A2 daboiatoxin B chain (Fragment) OS=Daboia siamensis OX=343250 PE=1 SV=1

Observed	Mr(expt)	Mr(calc)	ppm	Start	End	Miss	Ions	Peptide
1547.8329	1546.8256	1546.7232	66.2	78	91	1	---	R.TGKIVCGGDDPCLR.A
1847.8615	1846.8542	1846.8342	10.8	99	113	1	---	R.VAAICFRENMNTYDK.K + Oxidation (M)
2512.1416	2511.1344	2511.0584	30.3	29	50	1	---	K.YISYGCYCGWGGQGTPKDATDR.C

No match to: 855.0839, 866.5652, 869.4391, 871.0597, 888.4497, 914.4659, 1058.5857, 1067.4623, 1077.4684, 1126.5764, 1151.2418, 1172.6337, 1187.6382, 1188.6071, 1189.6613, 1205.6521, 1208.6536, 1212.6208, 1215.6757, 1221.6454, 1224.6463, 1227.6124, 1231.6432, 1232.1318, 1234.6710, 1237.6406, 1243.6014, 1246.6598, 1250.6174, 1256.6373, 1277.7306, 1279.5707, 1280.0813, 1304.5978, 1320.5878, 1372.7097, 1404.1747, 1404.6770, 1408.8019, 1429.7334, 1440.7365, 1475.7741, 1531.8497, 1581.7222, 1649.7094, 1687.9344, 1802.9425, 1817.9724, 1957.9238, 1993.9932, 2022.0576, 2053.0407, 2056.0529, 2061.0381, 2069.0547, 2094.0682, 2098.0584, 2106.0616, 2113.0782, 2118.0725, 2125.0731, 2128.0741, 2135.0518, 2139.0868, 2142.0621, 2145.0668, 2155.0825, 2160.0656, 2171.0780, 2265.0755, 2405.2266, 2417.2380, 2442.0921, 2443.2436, 2455.2338, 2462.2518, 2473.2567, 2476.2685, 2484.2157, 2488.2653, 2501.1605, 2530.2773, 2539.1490, 2558.1567, 2569.1587, 2574.1447, 2575.3339, 2614.2493, 3348.6481

26. A0A6G5ZW20_9SAUR **Mass:** 26588 **Score:** 18 **Expect:** 22 **Matches:** 4

Veficolin 8 (Fragment) OS=Vipera anatolica senliki OX=2604287 PE=2 SV=1

Observed	Mr(expt)	Mr(calc)	ppm	Start	End	Miss	Ions	Peptide
1067.4623	1066.4550	1066.5230	-63.72	103	110	1	---	K.FDTLQCKR.G
1215.6757	1214.6684	1214.6983	-24.57	28	38	1	---	K.LDGNSKLTVLR.G
1993.9932	1992.9859	1992.8935	46.4	39	59	0	---	R.GCPGMPGSPGPQGPPGFPGAR.G + Oxidation (M)
2113.0782	2112.0710	2111.9517	56.5	141	158	1	---	R.VLCDMDTDGGGWTVFQRR.S

No match to: 855.0839, 866.5652, 869.4391, 871.0597, 888.4497, 914.4659, 1058.5857, 1077.4684, 1126.5764, 1151.2418, 1172.6337, 1187.6382, 1188.6071, 1189.6613, 1205.6521, 1208.6536, 1212.6208, 1221.6454, 1224.6463, 1227.6124, 1231.6432, 1232.1318, 1234.6710, 1237.6406, 1243.6014, 1246.6598, 1250.6174, 1256.6373, 1277.7306, 1279.5707, 1280.0813, 1304.5978, 1320.5878, 1372.7097, 1404.1747, 1404.6770, 1408.8019, 1429.7334, 1440.7365, 1475.7741, 1531.8497, 1547.8329, 1581.7222, 1649.7094, 1687.9344, 1802.9425, 1817.9724, 1847.8615, 1957.9238, 2022.0576, 2053.0407, 2056.0529, 2061.0381, 2069.0547, 2094.0682, 2098.0584, 2106.0616, 2118.0725, 2125.0731, 2128.0741, 2135.0518, 2139.0868, 2142.0621, 2145.0668, 2155.0825, 2160.0656, 2171.0780, 2265.0755, 2405.2266, 2417.2380, 2442.0921, 2443.2436, 2455.2338, 2462.2518, 2473.2567, 2476.2685, 2484.2157, 2488.2653, 2501.1605, 2512.1416, 2530.2773, 2539.1490, 2558.1567, 2569.1587, 2574.1447, 2575.3339, 2614.2493, 3348.6481

27. A0A6G5ZVX6_9SAUR **Mass:** 37165 **Score:** 18 **Expect:** 22 **Matches:** 6

Veficolin 1 OS=Vipera anatolica senliki OX=2604287 PE=2 SV=1

Observed	Mr(expt)	Mr(calc)	ppm	Start	End	Miss	Ions	Peptide
914.4659	913.4587	913.4869	-30.88	267	274	0	---	K.LQDPNSLK.C
1067.4623	1066.4550	1066.5230	-63.72	110	117	1	---	K.FDTLQCKR.G
1215.6757	1214.6684	1214.6983	-24.57	35	45	1	---	K.LDGNSKLTVLR.G
1231.6432	1230.6359	1230.6179	14.6	323	332	1	---	K.HTEMKFRPVA.- + Oxidation (M)
1243.6014	1242.5941	1242.5669	21.9	313	322	1	---	K.SGRGYYYSYK.H
1993.9932	1992.9859	1993.0311	-22.68	4	21	0	---	R.AGKPAALFLLCLMATTCR.G

No match to: 855.0839, 866.5652, 869.4391, 871.0597, 888.4497, 1058.5857, 1077.4684, 1126.5764, 1151.2418, 1172.6337, 1187.6382, 1188.6071, 1189.6613, 1205.6521, 1208.6536, 1212.6208, 1221.6454, 1224.6463, 1227.6124, 1232.1318, 1234.6710, 1237.6406, 1246.6598, 1250.6174, 1256.6373, 1277.7306, 1279.5707, 1280.0813, 1304.5978, 1320.5878, 1372.7097, 1404.1747, 1404.6770, 1408.8019, 1429.7334, 1440.7365, 1475.7741, 1531.8497, 1547.8329, 1581.7222, 1649.7094, 1687.9344, 1802.9425, 1817.9724, 1847.8615, 1957.9238, 2022.0576, 2053.0407, 2056.0529, 2061.0381, 2069.0547, 2094.0682, 2098.0584, 2106.0616, 2113.0782, 2118.0725, 2125.0731, 2128.0741, 2135.0518, 2139.0868, 2142.0621, 2145.0668, 2155.0825, 2160.0656, 2171.0780, 2265.0755, 2405.2266, 2417.2380, 2442.0921, 2443.2436, 2455.2338, 2462.2518, 2473.2567, 2476.2685, 2484.2157, 2488.2653, 2501.1605, 2512.1416, 2530.2773, 2539.1490, 2558.1567, 2569.1587, 2574.1447, 2575.3339, 2614.2493, 3348.6481

28. A0A6G5ZW74_9SAUR **Mass:** 32808 **Score:** 18 **Expect:** 23 **Matches:** 5

Veficolin 9 (Fragment) OS=Vipera anatolica senliki OX=2604287 PE=2 SV=1

Observed	Mr(expt)	Mr(calc)	ppm	Start	End	Miss	Ions	Peptide
914.4659	913.4587	913.4869	-30.88	255	262	0	---	K.LQDPNSLK.C
1067.4623	1066.4550	1066.5230	-63.72	98	105	1	---	K.FDTLQCKR.G
1215.6757	1214.6684	1214.6983	-24.57	23	33	1	---	K.LDGNSKLTVLR.G
1993.9932	1992.9859	1992.8935	46.4	34	54	0	---	R.GCPGMPGSPGPQGPPGFPGAR.G + Oxidation (M)
2135.0518	2134.0445	2133.9837	28.5	136	153	1	---	R.VLCDMHTDGGGWTVFQRR.S

No match to: 855.0839, 866.5652, 869.4391, 871.0597, 888.4497, 1058.5857, 1077.4684, 1126.5764, 1151.2418, 1172.6337, 1187.6382, 1188.6071, 1189.6613, 1205.6521, 1208.6536, 1212.6208, 1221.6454, 1224.6463, 1227.6124, 1231.6432, 1232.1318, 1234.6710, 1237.6406, 1243.6014, 1246.6598, 1250.6174, 1256.6373, 1277.7306, 1279.5707, 1280.0813, 1304.5978, 1320.5878, 1372.7097, 1404.1747, 1404.6770, 1408.8019, 1429.7334, 1440.7365, 1475.7741, 1531.8497, 1547.8329, 1581.7222, 1649.7094, 1687.9344, 1802.9425, 1817.9724, 1847.8615, 1957.9238, 2022.0576, 2053.0407, 2056.0529, 2061.0381, 2069.0547, 2094.0682, 2098.0584, 2106.0616, 2113.0782, 2118.0725, 2125.0731, 2128.0741, 2139.0868, 2142.0621, 2145.0668, 2155.0825, 2160.0656, 2171.0780, 2265.0755, 2405.2266, 2417.2380, 2442.0921, 2443.2436, 2455.2338, 2462.2518, 2473.2567, 2476.2685, 2484.2157, 2488.2653, 2501.1605, 2512.1416, 2530.2773, 2539.1490, 2558.1567, 2569.1587, 2574.1447, 2575.3339, 2614.2493, 3348.6481

29. A0A170TXL0_VIPUR **Mass:** 25071 **Score:** 18 **Expect:** 24 **Matches:** 4

Brain-derived neurotrophic factor (Fragment) OS=Vipera ursinii macrops OX=927675 GN=BDNF PE=3 SV=1

Observed	Mr(expt)	Mr(calc)	ppm	Start	End	Miss	Ions	Peptide
914.4659	913.4587	913.4327	28.4	172	179	1	---	K.CSTKGYAK.E
1058.5857	1057.5784	1057.4936	80.2	1	9	1	---	-.SYMKAAPMK.E + 2 Oxidation (M)
1404.6770	1403.6697	1403.7085	-27.63	161	171	1	---	K.GQLKQYFYETK.C
2125.0731	2124.0658	2123.9793	40.7	121	139	1	---	R.RGELSVCDSTSEWVTAAEK.K

No match to: 855.0839, 866.5652, 869.4391, 871.0597, 888.4497, 1067.4623, 1077.4684, 1126.5764, 1151.2418, 1172.6337, 1187.6382, 1188.6071, 1189.6613, 1205.6521, 1208.6536, 1212.6208, 1215.6757, 1221.6454, 1224.6463, 1227.6124, 1231.6432, 1232.1318, 1234.6710,

1237.6406, 1243.6014, 1246.6598, 1250.6174, 1256.6373, 1277.7306, 1279.5707, 1280.0813, 1304.5978, 1320.5878, 1372.7097, 1404.1747, 1408.8019, 1429.7334, 1440.7365, 1475.7741, 1531.8497, 1547.8329, 1581.7222, 1649.7094, 1687.9344, 1802.9425, 1817.9724, 1847.8615, 1957.9238, 1993.9932, 2022.0576, 2053.0407, 2056.0529, 2061.0381, 2069.0547, 2094.0682, 2098.0584, 2106.0616, 2113.0782, 2118.0725, 2128.0741, 2135.0518, 2139.0868, 2142.0621, 2145.0668, 2155.0825, 2160.0656, 2171.0780, 2265.0755, 2405.2266, 2417.2380, 2442.0921, 2443.2436, 2455.2338, 2462.2518, 2473.2567, 2476.2685, 2484.2157, 2488.2653, 2501.1605, 2512.1416, 2530.2773, 2539.1490, 2558.1567, 2569.1587, 2574.1447, 2575.3339, 2614.2493, 3348.6481

30. A0A6G5ZUQ8_9SAUR **Mass:** 130702 **Score:** 17 **Expect:** 28 **Matches:** 9

Rabankyrin OS=Vipera anatolica senliki OX=2604287 PE=2 SV=1

Observed	Mr(expt)	Mr(calc)	ppm	Start	End	Miss	Ions	Peptide
1188.6071	1187.5998	1187.6584	-49.29	1136 -	1145	1	---	K.CSTKEIPIIK.F
1243.6014	1242.5941	1242.6615	-54.26	1054 -	1064	1	---	K.GNANLCRAIVR.A
1429.7334	1428.7261	1428.7911	-45.49	12 -	22	1	---	K.HLMLLRQEYVK.L
1581.7222	1580.7149	1580.6451	44.2	755 -	769	0	---	R.QPGANGEGDEEAHDR.Q
1957.9238	1956.9165	1956.7441	88.1	1100 -	1115	0	---	K.EPPWCDGSNCYECGVK.F
2128.0741	2127.0669	2127.1072	-18.97	632 -	650	1	---	R.QDSKSALFLLEHQANISAR.T
2155.0825	2154.0752	2154.1096	-15.96	54 -	71	0	---	R.LLTIVAELYEQEQYSDLK.I
2476.2685	2475.2612	2475.2982	-14.96	1065 -	1087	1	---	R.AGARLGINNNQGINIFNYQVATK.Q
2569.1587	2568.1515	2568.3483	-76.62	369 -	390	1	---	R.TPLHSSIVVRNDQVFSQLLQCK.Q

No match to: 855.0839, 866.5652, 869.4391, 871.0597, 888.4497, 914.4659, 1058.5857, 1067.4623, 1077.4684, 1126.5764, 1151.2418, 1172.6337, 1187.6382, 1189.6613, 1205.6521, 1208.6536, 1212.6208, 1215.6757, 1221.6454, 1224.6463, 1227.6124, 1231.6432, 1232.1318, 1234.6710, 1237.6406, 1246.6598, 1250.6174, 1256.6373, 1277.7306, 1279.5707, 1280.0813, 1304.5978, 1320.5878, 1372.7097, 1404.1747, 1404.6770, 1408.8019, 1440.7365, 1475.7741, 1531.8497, 1547.8329, 1649.7094, 1687.9344, 1802.9425, 1817.9724, 1847.8615, 1993.9932, 2022.0576, 2053.0407, 2056.0529, 2061.0381, 2069.0547, 2094.0682, 2098.0584, 2106.0616, 2113.0782, 2118.0725, 2125.0731, 2135.0518, 2139.0868, 2142.0621, 2145.0668, 2160.0656, 2171.0780, 2265.0755, 2405.2266, 2417.2380, 2442.0921, 2443.2436, 2455.2338, 2462.2518, 2473.2567, 2484.2157, 2488.2653, 2501.1605, 2512.1416, 2530.2773, 2539.1490, 2558.1567, 2574.1447, 2575.3339, 2614.2493, 3348.6481

Search Parameters

**Type of search**	: MS/MS Ion Search
**Enzyme**	: Trypsin
**Fixed modifications**	: Carbamidomethyl (C)
**Variable modifications**	: Oxidation (M)
**Mass values**	: Monoisotopic
**Protein Mass**	: Unrestricted
**Peptide Mass Tolerance**	: ± 100 ppm
**Fragment Mass Tolerance**	: ± 0.7 Da
**Max Missed Cleavages**	: 1
**Instrument type**	: MALDI-TOF-TOF
**Query1 (855.0839,1+)**	: <no title>
**Query2 (866.5652,1+)**	: <no title>
**Query3 (869.4391,1+)**	: <no title>
**Query4 (871.0597,1+)**	: <no title>
**Query5 (888.4497,1+)**	: \\PROTEOMICS1\D\DATA\2105\210505_TLIEPOL_1_68252-68266\68252_68266_LPARDO\0_G11\1\888.4497.LIFT\1SREF\PDATA\1\1R
**Query6 (914.4659,1+)**	: <no title>
**Query7 (1058.5857,1+)**	: <no title>
**Query8 (1067.4623,1+)**	: <no title>
**Query9 (1077.4684,1+)**	: <no title>
**Query10 (1126.5764,1+)**	: <no title>
**Query11 (1151.2418,1+)**	: <no title>
**Query12 (1172.6337,1+)**	: <no title>
**Query13 (1187.6382,1+)**	: <no title>
**Query14 (1188.6071,1+)**	: <no title>
**Query15 (1189.6613,1+)**	: \\PROTEOMICS1\D\DATA\2105\210505_TLIEPOL_1_68252-68266\68252_68266_LPARDO\0_G11\1\1189.6613.LIFT\1SREF\PDATA\1\1R
**Query16 (1205.6521,1+)**	: \\PROTEOMICS1\D\DATA\2105\210505_TLIEPOL_1_68252-68266\68252_68266_LPARDO\0_G11\1\1205.6521.LIFT\1SREF\PDATA\1\1R
**Query17 (1208.6536,1+)**	: \\PROTEOMICS1\D\DATA\2105\210505_TLIEPOL_1_68252-68266\68252_68266_LPARDO\0_G11\1\1208.6536.LIFT\1SREF\PDATA\1\1R
**Query18 (1212.6208,1+)**	: \\PROTEOMICS1\D\DATA\2105\210505_TLIEPOL_1_68252-68266\68252_68266_LPARDO\0_G11\1\1212.6208.LIFT\1SREF\PDATA\1\1R
**Query19 (1215.6757,1+)**	: <no title>
**Query20 (1221.6454,1+)**	: <no title>
**Query21 (1224.6463,1+)**	: <no title>
**Query22 (1227.6124,1+)**	: <no title>
**Query23 (1231.6432,1+)**	: <no title>
**Query24 (1232.1318,1+)**	: <no title>
**Query25 (1234.6710,1+)**	: <no title>
**Query26 (1237.6406,1+)**	: <no title>
**Query27 (1243.6014,1+)**	: <no title>
**Query28 (1246.6598,1+)**	: <no title>
**Query29 (1250.6174,1+)**	: <no title>
**Query30 (1256.6373,1+)**	: <no title>
**Query31 (1277.7306,1+)**	: <no title>
**Query32 (1279.5707,1+)**	: <no title>
**Query33 (1280.0813,1+)**	: <no title>
**Query34 (1304.5978,1+)**	: <no title>
**Query35 (1320.5878,1+)**	: <no title>
**Query36 (1372.7097,1+)**	: <no title>
**Query37 (1404.1747,1+)**	: <no title>
**Query38 (1404.6770,1+)**	: <no title>
**Query39 (1408.8019,1+)**	: <no title>
**Query40 (1429.7334,1+)**	: \\PROTEOMICS1\D\DATA\2105\210505_TLIEPOL_1_68252-68266\68252_68266_LPARDO\0_G11\1\1429.7334.LIFT\1SREF\PDATA\1\1R
**Query41 (1440.7365,1+)**	: <no title>
**Query42 (1475.7741,1+)**	: <no title>
**Query43 (1531.8497,1+)**	: <no title>
**Query44 (1547.8329,1+)**	: <no title>
**Query45 (1581.7222,1+)**	: <no title>
**Query46 (1649.7094,1+)**	: <no title>
**Query47 (1687.9344,1+)**	: <no title>
**Query48 (1802.9425,1+)**	: <no title>
**Query49 (1817.9724,1+)**	: \\PROTEOMICS1\D\DATA\2105\210505_TLIEPOL_1_68252-68266\68252_68266_LPARDO\0_G11\1\1817.9724.LIFT\1SREF\PDATA\1\1R
**Query50 (1847.8615,1+)**	: <no title>
**Query51 (1957.9238,1+)**	: <no title>
**Query52 (1993.9932,1+)**	: <no title>
**Query53 (2022.0576,1+)**	: <no title>
**Query54 (2053.0407,1+)**	: <no title>
**Query55 (2056.0529,1+)**	: <no title>
**Query56 (2061.0381,1+)**	: <no title>
**Query57 (2069.0547,1+)**	: <no title>
**Query58 (2094.0682,1+)**	: <no title>
**Query59 (2098.0584,1+)**	: <no title>
**Query60 (2106.0616,1+)**	: <no title>
**Query61 (2113.0782,1+)**	: \\PROTEOMICS1\D\DATA\2105\210505_TLIEPOL_1_68252-68266\68252_68266_LPARDO\0_G11\1\2113.0782.LIFT\1SREF\PDATA\1\1R
**Query62 (2118.0725,1+)**	: <no title>
**Query63 (2125.0731,1+)**	: <no title>
**Query64 (2128.0741,1+)**	: <no title>

Query65 (2135.0518,1+) : <no title>
Query66 (2139.0868,1+) : <no title>
Query67 (2142.0621,1+) : <no title>
Query68 (2145.0668,1+) : \\PROTEOMICS1\D\DATA\2105\210505_TLIEPOL_1_68252-68266\68252_68266_LPARDO\0_G11\1\2145.0668.LIFT\1SREF\PDATA\1\1R
Query69 (2155.0825,1+) : <no title>
Query70 (2160.0656,1+) : <no title>
Query71 (2171.0780,1+) : <no title>
Query72 (2265.0755,1+) : <no title>
Query73 (2405.2266,1+) : <no title>
Query74 (2417.2380,1+) : <no title>
Query75 (2442.0921,1+) : <no title>
Query76 (2443.2436,1+) : <no title>
Query77 (2455.2338,1+) : <no title>
Query78 (2462.2518,1+) : \\PROTEOMICS1\D\DATA\2105\210505_TLIEPOL_1_68252-68266\68252_68266_LPARDO\0_G11\1\2462.2518.LIFT\1SREF\PDATA\1\1R
Query79 (2473.2567,1+) : <no title>
Query80 (2476.2685,1+) : <no title>
Query81 (2484.2157,1+) : <no title>
Query82 (2488.2653,1+) : <no title>
Query83 (2501.1605,1+) : <no title>
Query84 (2512.1416,1+) : <no title>
Query85 (2530.2773,1+) : <no title>
Query86 (2539.1490,1+) : <no title>
Query87 (2558.1567,1+) : \\PROTEOMICS1\D\DATA\2105\210505_TLIEPOL_1_68252-68266\68252_68266_LPARDO\0_G11\1\2558.1567.LIFT\1SREF\PDATA\1\1R
Query88 (2569.1587,1+) : <no title>
Query89 (2574.1447,1+) : \\PROTEOMICS1\D\DATA\2105\210505_TLIEPOL_1_68252-68266\68252_68266_LPARDO\0_G11\1\2574.1447.LIFT\1SREF\PDATA\1\1R
Query90 (2575.3339,1+) : <no title>
Query91 (2614.2493,1+) : <no title>
Query92 (3348.6481,1+) : <no title>

Mascot: http://www.matrixscience.com/

Mascot Search Results Fr6-5

```
User          :
Email         :
Search title  : vipera_All_8IDplus4 file:\\Proteomics1\d\data\2105\210505_tliepol_1_68252-68266\68252_68266_lpardo\0_H11\1\1SRef\pdata\1\peaklist.xml
Database      : vipera  (1414 sequences; 347452 residues)
Timestamp     : 5 May 2021 at 09:38:21 GMT
Top Score     : 41 for OXLA_DABRR, L-amino-acid oxidase OS=Daboia russelii OX=8707 PE=1 SV=1
```

Mascot Score Histogram

Protein score is -10*Log(P), where P is the probability that the observed match is a random event.
Protein scores greater than 44 are significant (p<0.05).

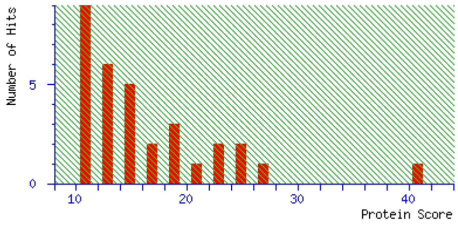

Concise Protein Summary Report

Format As | Concise Protein Summary | Help

Significance threshold p< 0.05 Max. number of hits 10

Re-Search All | Search Unmatched

1. OXLA_DABRR **Mass:** 57251 **Score:** 41 **Expect:** 0.12 **Matches:** 10
L-amino-acid oxidase OS=Daboia russelii OX=8707 PE=1 SV=1

---

2. A0A6B7FPT0_VIPAA **Mass:** 15144 **Score:** 27 **Expect:** 2.8 **Matches:** 4
Endogenous tripeptide metalloproteinase inhibitor MPi-5 OS=Vipera ammodytes ammodytes OX=8705 PE=2 SV=1
A0A1J0CZN1_VIPAA **Mass:** 20176 **Score:** 26 **Expect:** 3.4 **Matches:** 4
SVMPi-1 OS=Vipera ammodytes ammodytes OX=8705 PE=2 SV=1
A0A1I9KNP4_VIPAA **Mass:** 20248 **Score:** 26 **Expect:** 3.4 **Matches:** 4
Endogenous tripeptide metalloproteinase inhibitor OS=Vipera ammodytes ammodytes OX=8705 PE=2 SV=1
A0A6B7FNM6_VIPAA **Mass:** 23049 **Score:** 16 **Expect:** 37 **Matches:** 3
MPi-3 OS=Vipera ammodytes ammodytes OX=8705 PE=2 SV=1
A0A1I9KNP8_VIPAA **Mass:** 27209 **Score:** 14 **Expect:** 53 **Matches:** 3
Endogenous tripeptide metalloproteinase inhibitor OS=Vipera ammodytes ammodytes OX=8705 PE=2 SV=1
3SA1_DABRR **Mass:** 2363 **Score:** 12 **Expect:** 98 **Matches:** 1
Cytotoxin drCT-1 (Fragment) OS=Daboia russelii OX=8707 PE=1 SV=1

---

3. A0A6G5ZVL0_9SAUR **Mass:** 21283 **Score:** 25 **Expect:** 4.4 **Matches:** 5
Cholinesterase 14 (Fragment) OS=Vipera anatolica senliki OX=2604287 PE=2 SV=1
A0A6G5ZVP1_9SAUR **Mass:** 29798 **Score:** 21 **Expect:** 10 **Matches:** 5
Cholinesterase 8 (Fragment) OS=Vipera anatolica senliki OX=2604287 PE=2 SV=1
A0A6G5ZVU2_9SAUR **Mass:** 59723 **Score:** 13 **Expect:** 74 **Matches:** 5
Carboxylic ester hydrolase OS=Vipera anatolica senliki OX=2604287 PE=2 SV=1
A0A6G5ZU77_9SAUR **Mass:** 59771 **Score:** 13 **Expect:** 74 **Matches:** 5
Carboxylic ester hydrolase OS=Vipera anatolica senliki OX=2604287 PE=2 SV=1

---

4. OXLA_VIPBB **Mass:** 10345 **Score:** 25 **Expect:** 4.7 **Matches:** 3
L-amino-acid oxidase (Fragments) OS=Vipera berus berus OX=31156 PE=1 SV=1

---

5. V9I1A1_BUNFA **Mass:** 27785 **Score:** 24 **Expect:** 6.3 **Matches:** 4
Venom nerve growth factor OS=Bungarus fasciatus OX=8613 PE=2 SV=1

---

6. OXLA_MACLB **Mass:** 12541 **Score:** 23 **Expect:** 7.6 **Matches:** 4
L-amino-acid oxidase (Fragments) OS=Macrovipera lebetina OX=8709 PE=1 SV=2

---

7. A0A6G5ZW74_9SAUR **Mass:** 32808 **Score:** 21 **Expect:** 11 **Matches:** 4
Veficolin 9 (Fragment) OS=Vipera anatolica senliki OX=2604287 PE=2 SV=1
A0A6G5ZVU3_9SAUR **Mass:** 11896 **Score:** 14 **Expect:** 63 **Matches:** 2
Veficolin 4 (Fragment) OS=Vipera anatolica senliki OX=2604287 PE=2 SV=1
A0A6G5ZVX6_9SAUR **Mass:** 37165 **Score:** 12 **Expect:** 85 **Matches:** 3
Veficolin 1 OS=Vipera anatolica senliki OX=2604287 PE=2 SV=1

---

8. K4GRJ2_DABRR **Mass:** 41352 **Score:** 20 **Expect:** 16 **Matches:** 5
Solute carrier family 8 member 3 (Fragment) OS=Daboia russelii OX=8707 GN=SLC8A3 PE=4 SV=1

---

9. A0A6G5ZVY6_9SAUR **Mass:** 24441 **Score:** 18 **Expect:** 23 **Matches:** 3
Veficolin 6 (Fragment) OS=Vipera anatolica senliki OX=2604287 PE=2 SV=1
A0A6G5ZUK5_9SAUR **Mass:** 26961 **Score:** 17 **Expect:** 29 **Matches:** 3
Veficolin 5 (Fragment) OS=Vipera anatolica senliki OX=2604287 PE=2 SV=1

---

10. A0A6B7FRF2_VIPAA **Mass:** 57466 **Score:** 18 **Expect:** 23 **Matches:** 7
Amine oxidase OS=Vipera ammodytes ammodytes OX=8705 PE=2 SV=1

Search Parameters

```
Type of search          : Peptide Mass Fingerprint
Enzyme                  : Trypsin
Fixed modifications     : Carbamidomethyl (C)
Variable modifications  : Oxidation (M)
Mass values             : Monoisotopic
Protein Mass            : Unrestricted
Peptide Mass Tolerance  : ± 100 ppm
```

```
Peptide Charge State   : 1+
Max Missed Cleavages   : 1
Number of queries      : 90
Selected for scoring   : 63
```

**Mascot:** http://www.matrixscience.com/

MATRIX SCIENCE Mascot Search Results

```
User          :
Email         :
Search title  : vipera_all_Lift file:\\Proteomics1\d\data\2105\210505_tliepol_1_68252-68266\68252_68266_lpardo\0_H11\1\0_H11.xml
Database      : vipera  (1414 sequences; 347452 residues)
Timestamp     : 5 May 2021 at 10:09:59 GMT
Protein hits  : A0A6G5ZUR6_9SAUR Serine protease 6 OS=Vipera anatolica senliki OX=2604287 PE=2 SV=1
                OXLA_MACLB       L-amino-acid oxidase (Fragments) OS=Macrovipera lebetina OX=8709 PE=1 SV=2
                OXLA_DABRR       L-amino-acid oxidase OS=Daboia russelii OX=8707 PE=1 SV=1
                A0A0C5DKK6_MACLB C-type lectin-like protein 2A OS=Macrovipera lebetina OX=8709 PE=2 SV=1
                A0A6G5ZU77_9SAUR Carboxylic ester hydrolase OS=Vipera anatolica senliki OX=2604287 PE=2 SV=1
                A0A6G5ZUK1_9SAUR Veficolin 7 (Fragment) OS=Vipera anatolica senliki OX=2604287 PE=2 SV=1
                A0A223PK32_DABRR Atrial natriuretic peptide receptor 3 (Fragment) OS=Daboia russelii OX=8707 PE=2 SV=1
```

Mascot Score Histogram

Ions score is -10*Log(P), where P is the probability that the observed match is a random event.
Individual ions scores > 11 indicate identity or extensive homology (p<0.05).
Protein scores are derived from ions scores as a non-probabilistic basis for ranking protein hits.

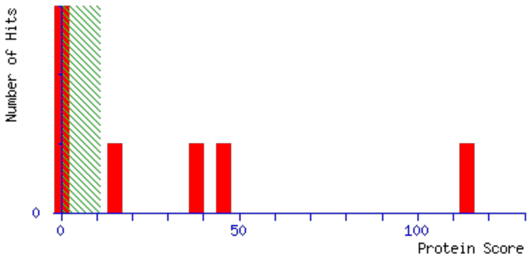

Peptide Summary Report

Format As: Peptide Summary — Help

Significance threshold p< 0.05 — Max. number of hits 10

Standard scoring ○ MudPIT scoring ◉ — Ions score or expect cut-off 0 — Show sub-sets 0

Show pop-ups ◉ Suppress pop-ups ○ — Sort unassigned Decreasing Score — Require bold red ☐

Overview Table

Click on column header to jump to entry in results list.
Move mouse over any indicator to highlight identical peptides.
Click on an indicator to see details of individual match.
Use check boxes to select sub-set of queries for new search.

Mouse over: -Query- -Accession- -Sequence-

Hit:	1	2	3	4	5	6	7
☑ 807.4626 (1+)							
☑ 817.1426 (1+)							
☑ 830.0417 (1+)							
☑ 837.1202 (1+)							
☑ 839.1338 (1+)							
☑ 855.1078 (1+)							
☑ 861.1117 (1+)							
☑ 865.5404 (1+)							
☑ 868.5782 (1+)							
☑ 871.0816 (1+)							
☑ 877.0924 (1+)							
☑ 880.0422 (1+)							
☑ 882.6070 (1+)							
☑ 896.0391 (1+)							
☑ 903.4150 (1+)							
☑ 946.0345 (1+)							
☑ 952.4629 (1+)							
☑ 1015.5952 (1+)				●	●		
☑ 1022.1687 (1+)							
☑ 1022.5059 (1+)							
☑ 1044.1445 (1+)							
☑ 1049.5180 (1+)							

☑ 1064.1066 (1+)							
☑ 1064.5361 (1+)							
☑ 1066.1255 (1+)							
☑ 1068.5260 (1+)							
☑ 1082.1004 (1+)							
☑ 1126.5931 (1+)							
☑ 1149.5092 (1+)							
☑ 1157.6688 (1+)						●	
☑ 1161.6596 (1+)							
☑ 1173.6625 (1+)							
☑ 1175.6989 (1+)							
☑ 1179.6574 (1+)							
☑ 1189.6575 (1+)							
☑ 1195.6212 (1+)							
☑ 1205.6570 (1+)							
☑ 1233.6978 (1+)							
☑ 1249.1574 (1+)							
☑ 1252.6580 (1+)							
☑ 1255.0832 (1+)							
☑ 1255.6667 (1+)							
☑ 1267.6429 (1+)							
☑ 1271.1302 (1+)							
☑ 1277.7173 (1+)							
☑ 1285.7642 (1+)							
☑ 1293.6535 (1+)			●				
☑ 1309.6407 (1+)							
☑ 1311.7849 (1+)							
☑ 1319.6678 (1+)							
☑ 1323.6421 (1+)							
☑ 1325.6405 (1+)							
☑ 1330.5704 (1+)							
☑ 1335.6602 (1+)							
☑ 1340.6439 (1+)							
☑ 1346.5595 (1+)							
☑ 1356.5932 (1+)							
☑ 1370.7905 (1+)							
☑ 1404.1990 (1+)							
☑ 1404.7008 (1+)							
☑ 1408.8268 (1+)							
☑ 1475.7653 (1+)							
☑ 1493.7263 (1+)		●					
☑ 1497.8121 (1+)							
☑ 1500.7156 (1+)							
☑ 1519.7543 (1+)							
☑ 1522.7117 (1+)							
☑ 1535.7486 (1+)							
☑ 1538.6707 (1+)							
☑ 1592.8193 (1+)	●						
☑ 1606.8322 (1+)							
☑ 1614.8130 (1+)							
☑ 1625.9006 (1+)							
☑ 1630.7808 (1+)							
☑ 1636.7653 (1+)							●
☑ 1698.8360 (1+)							
☑ 1718.7900 (1+)							
☑ 1818.0057 (1+)							
☑ 1957.9524 (1+)							
☑ 1967.8707 (1+)							
☑ 1994.0207 (1+)							
☑ 2134.9888 (1+)							
☑ 2260.1615 (1+)							
☑ 2265.2520 (1+)							
☑ 2277.1374 (1+)							
☑ 2281.2429 (1+)							
☑ 2682.1989 (1+)							
☑ 2914.5586 (1+)							
☑ 3095.6408 (1+)							
☑ 3347.7282 (1+)							

Select All | Select None | Search Selected | ☐ **Error tolerant** | Archive Report

1. A0A6G5ZUR6_9SAUR **Mass:** 29579 **Score:** 114 **Matches:** 1(1) **Sequences:** 1(1)
Serine protease 6 OS=Vipera anatolica senliki OX=2604287 PE=2 SV=1
☐ Check to include this hit in error tolerant search or archive report

Query	Observed	Mr(expt)	Mr(calc)	ppm	Miss	Score	Expect	Rank	Unique	Peptide
70	1592.8193	1591.8121	1591.7698	26.5	0	114	4.3e-012	1	U	R.TLCAGILQGGIDSCK.V

Proteins matching the same set of peptides:
A0A6G5ZVX7_9SAUR **Mass:** 29593 **Score:** 114 **Matches:** 1(1) **Sequences:** 1(1)
Serine protease 7 OS=Vipera anatolica senliki OX=2604287 PE=2 SV=1
VASP1_VIPAA **Mass:** 22635 **Score:** 114 **Matches:** 1(1) **Sequences:** 1(1)
Snake venom serine protease VaSP1 (Fragments) OS=Vipera ammodytes ammodytes OX=8705 PE=1 SV=1
VSPH1_VIPAA **Mass:** 29593 **Score:** 114 **Matches:** 1(1) **Sequences:** 1(1)
Vaa serine proteinase homolog 1 OS=Vipera ammodytes ammodytes OX=8705 PE=1 SV=1
VSP2_MACLB **Mass:** 29559 **Score:** 114 **Matches:** 1(1) **Sequences:** 1(1)
Venom serine proteinase-like protein 2 OS=Macrovipera lebetina OX=8709 PE=2 SV=1

---

2. OXLA_MACLB **Mass:** 12541 **Score:** 47 **Matches:** 1(1) **Sequences:** 1(1)
L-amino-acid oxidase (Fragments) OS=Macrovipera lebetina OX=8709 PE=1 SV=2
☐ Check to include this hit in error tolerant search or archive report

Query	Observed	Mr(expt)	Mr(calc)	ppm	Miss	Score	Expect	Rank	Unique	Peptide
63	1493.7263	1492.7190	1492.6616	38.5	1	47	1.9e-005	1	U	-.ADDKNPLEECFR.E

Proteins matching the same set of peptides:
OXLA_VIPBB **Mass:** 10345 **Score:** 47 **Matches:** 1(1) **Sequences:** 1(1)
L-amino-acid oxidase (Fragments) OS=Vipera berus berus OX=31156 PE=1 SV=1

---

3. OXLA_DABRR **Mass:** 57251 **Score:** 40 **Matches:** 1(1) **Sequences:** 1(1)
L-amino-acid oxidase OS=Daboia russelii OX=8707 PE=1 SV=1
☐ Check to include this hit in error tolerant search or archive report

Query	Observed	Mr(expt)	Mr(calc)	ppm	Miss	Score	Expect	Rank	Unique	Peptide
47	1293.6535	1292.6462	1292.5972	37.9	0	40	0.0001	1	U	K.EGWYANLGPMR.V

Proteins matching the same set of peptides:
OXLA_VIPAA **Mass:** 55056 **Score:** 40 **Matches:** 1(1) **Sequences:** 1(1)
L-amino-acid oxidase OS=Vipera ammodytes ammodytes OX=8705 PE=1 SV=1

---

4. A0A0C5DKK6_MACLB **Mass:** 18110 **Score:** 17 **Matches:** 1(1) **Sequences:** 1(1)
C-type lectin-like protein 2A OS=Macrovipera lebetina OX=8709 PE=2 SV=1
☐ Check to include this hit in error tolerant search or archive report

Query	Observed	Mr(expt)	Mr(calc)	ppm	Miss	Score	Expect	Rank	Unique	Peptide
18	1015.5952	1014.5879	1014.5399	47.3	0	17	0.025	1	U	K.YHAWIGLR.D

Proteins matching the same set of peptides:
A0A1I9KNN1_VIPAA **Mass:** 17843 **Score:** 17 **Matches:** 1(1) **Sequences:** 1(1)
C-type lectin snaclec-3 OS=Vipera ammodytes ammodytes OX=8705 PE=2 SV=1

---

5. A0A6G5ZU77_9SAUR **Mass:** 59771 **Score:** 0 **Matches:** 1(0) **Sequences:** 1(0)
Carboxylic ester hydrolase OS=Vipera anatolica senliki OX=2604287 PE=2 SV=1
☐ Check to include this hit in error tolerant search or archive report

Query	Observed	Mr(expt)	Mr(calc)	ppm	Miss	Score	Expect	Rank	Unique	Peptide
18	1015.5952	1014.5879	1014.4957	90.9	0	3	0.64	2	U	R.LAFSQYMR.D

Proteins matching the same set of peptides:
A0A6G5ZVL0_9SAUR **Mass:** 21283 **Score:** 0 **Matches:** 1(0) **Sequences:** 1(0)
Cholinesterase 14 (Fragment) OS=Vipera anatolica senliki OX=2604287 PE=2 SV=1
A0A6G5ZVP1_9SAUR **Mass:** 29798 **Score:** 0 **Matches:** 1(0) **Sequences:** 1(0)
Cholinesterase 8 (Fragment) OS=Vipera anatolica senliki OX=2604287 PE=2 SV=1
A0A6G5ZVU2_9SAUR **Mass:** 59723 **Score:** 0 **Matches:** 1(0) **Sequences:** 1(0)
Carboxylic ester hydrolase OS=Vipera anatolica senliki OX=2604287 PE=2 SV=1

---

6. A0A6G5ZUK1_9SAUR **Mass:** 15084 **Score:** 0 **Matches:** 1(0) **Sequences:** 1(0)
Veficolin 7 (Fragment) OS=Vipera anatolica senliki OX=2604287 PE=2 SV=1
☐ Check to include this hit in error tolerant search or archive report

Query	Observed	Mr(expt)	Mr(calc)	ppm	Miss	Score	Expect	Rank	Unique	Peptide
30	1157.6688	1156.6615	1156.6088	45.6	1	2	0.58	1	U	K.DKLQDPNSLK.C

Proteins matching the same set of peptides:
A0A6G5ZVU3_9SAUR **Mass:** 11896 **Score:** 0 **Matches:** 1(0) **Sequences:** 1(0)
Veficolin 4 (Fragment) OS=Vipera anatolica senliki OX=2604287 PE=2 SV=1

A0A6G5ZVX6_9SAUR **Mass:** 37165 **Score:** 0 **Matches:** 1(0) **Sequences:** 1(0)
Veficolin 1 OS=Vipera anatolica senliki OX=2604287 PE=2 SV=1
A0A6G5ZW74_9SAUR **Mass:** 32808 **Score:** 0 **Matches:** 1(0) **Sequences:** 1(0)
Veficolin 9 (Fragment) OS=Vipera anatolica senliki OX=2604287 PE=2 SV=1

---

7. A0A223PK32_DABRR **Mass:** 57907 **Score:** 0 **Matches:** 1(0) **Sequences:** 1(0)
Atrial natriuretic peptide receptor 3 (Fragment) OS=Daboia russelii OX=8707 PE=2 SV=1
☐ Check to include this hit in error tolerant search or archive report

Query	Observed	Mr(expt)	Mr(calc)	ppm	Miss	Score	Expect	Rank	Unique	Peptide
75	1636.7653	1635.7580	1635.7100	29.3	1	2	0.64	1	U	K.FRLFYSDSDCGNR.A

Peptide matches not assigned to protein hits: (no details means no match)

	Query	Observed	Mr(expt)	Mr(calc)	ppm	Miss	Score	Expect	Rank	Unique	Peptide
☑	1	807.4626	806.4553								
☑	2	817.1426	816.1354								
☑	3	830.0417	829.0345								
☑	4	837.1202	836.1129								
☑	5	839.1338	838.1266								
☑	6	855.1078	854.1006								
☑	7	861.1117	860.1044								
☑	8	865.5404	864.5331								
☑	9	868.5782	867.5710								
☑	10	871.0816	870.0743								
☑	11	877.0924	876.0851								
☑	12	880.0422	879.0349								
☑	13	882.6070	881.5998								
☑	14	896.0391	895.0318								
☑	15	903.4150	902.4077								
☑	16	946.0345	945.0272								
☑	17	952.4629	951.4556								
☑	19	1022.1687	1021.1615								
☑	20	1022.5059	1021.4986								
☑	21	1044.1445	1043.1372								
☑	22	1049.5180	1048.5108								
☑	23	1064.1066	1063.0993								
☑	24	1064.5361	1063.5288								
☑	25	1066.1255	1065.1182								
☑	26	1068.5260	1067.5187								
☑	27	1082.1004	1081.0932								
☑	28	1126.5931	1125.5858								
☑	29	1149.5092	1148.5019								
☑	31	1161.6596	1160.6523								
☑	32	1173.6625	1172.6552								
☑	33	1175.6989	1174.6916								
☑	34	1179.6574	1178.6502								
☑	35	1189.6575	1188.6502								
☑	36	1195.6212	1194.6139								
☑	37	1205.6570	1204.6497								
☑	38	1233.6978	1232.6905								
☑	39	1249.1574	1248.1501								
☑	40	1252.6580	1251.6507								
☑	41	1255.0832	1254.0759								
☑	42	1255.6667	1254.6594								
☑	43	1267.6429	1266.6356								
☑	44	1271.1302	1270.1229								
☑	45	1277.7173	1276.7100								
☑	46	1285.7642	1284.7569								
☑	48	1309.6407	1308.6334								
☑	49	1311.7849	1310.7776								
☑	50	1319.6678	1318.6605								
☑	51	1323.6421	1322.6349								
☑	52	1325.6405	1324.6333								
☑	53	1330.5704	1329.5631								
☑	54	1335.6602	1334.6529								
☑	55	1340.6439	1339.6367								
☑	56	1346.5595	1345.5522								
☑	57	1356.5932	1355.5859								
☑	58	1370.7905	1369.7832								
☑	59	1404.1990	1403.1918								
☑	60	1404.7008	1403.6935								
☑	61	1408.8268	1407.8195								
☑	62	1475.7653	1474.7581								
☑	64	1497.8121	1496.8048								
☑	65	1500.7156	1499.7083								
☑	66	1519.7543	1518.7470								
☑	67	1522.7117	1521.7044								
☑	68	1535.7486	1534.7413								
☑	69	1538.6707	1537.6634								

☑	71	*1606.8322*	*1605.8249*
☑	72	*1614.8130*	*1613.8057*
☑	73	*1625.9006*	*1624.8933*
☑	74	*1630.7808*	*1629.7735*
☑	76	*1698.8360*	*1697.8287*
☑	77	*1718.7900*	*1717.7827*
☑	78	*1818.0057*	*1816.9984*
☑	79	*1957.9524*	*1956.9451*
☑	80	*1967.8707*	*1966.8634*
☑	81	*1994.0207*	*1993.0134*
☑	82	*2134.9888*	*2133.9816*
☑	83	*2260.1615*	*2259.1542*
☑	84	*2265.2520*	*2264.2447*
☑	85	*2277.1374*	*2276.1301*
☑	86	*2281.2429*	*2280.2356*
☑	87	*2682.1989*	*2681.1916*
☑	88	*2914.5586*	*2913.5514*
☑	89	*3095.6408*	*3094.6335*
☑	90	*3347.7282*	*3346.7209*

Search Parameters

```
Type of search         : MS/MS Ion Search
Enzyme                 : Trypsin
Fixed modifications    : Carbamidomethyl (C)
Variable modifications : Oxidation (M)
Mass values            : Monoisotopic
Protein Mass           : Unrestricted
Peptide Mass Tolerance : ± 100 ppm
Fragment Mass Tolerance: ± 0.7 Da
Max Missed Cleavages   : 1
Instrument type        : MALDI-TOF-TOF
Number of queries      : 90
```

Mascot: http://www.matrixscience.com/

Mascot Search Results

```
User          :
Email         :
Search title  : vipera_all_Lift file:\\Proteomics1\d\data\2105\210505_tliepol_1_68252-68266\68252_68266_lpardo\0_H11\1\0_H11.xml
Database      : vipera  (1414 sequences; 347452 residues)
Timestamp     : 5 May 2021 at 10:09:59 GMT
Warning       : A Peptide summary report will usually give a much clearer picture of MS/MS search results.
Top Score     : 121 for VASP1_VIPAA, Snake venom serine protease VaSP1 (Fragments) OS=Vipera ammodytes ammodytes OX=8705 PE=1 SV=1
```

Mascot Score Histogram

Protein score is -10*Log(P), where P is the probability that the observed match is a random event.
Protein scores greater than 44 are significant (p<0.05).
Protein scores are derived from ions scores as a non-probabilistic basis for ranking protein hits.

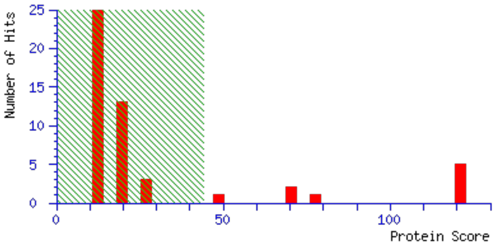

Protein Summary Report

Format As | Protein Summary (deprecated) | Help

Significance threshold p< 0.05 Max. number of hits 20

Overview Table

Click on column header to jump to entry in results list.
Move mouse over any indicator to highlight identical peptides.
Click on an indicator to see details of individual match.
Use check boxes to select sub-set of queries for new search.

Mouse over: -Query- -Accession- -Sequence-

Hit:	1	2	3	4	5	6	7	8	9	10	11	12	13	14	15	16	17	18	19	20
☑ 807.4626 (1+)																				
☑ 817.1426 (1+)																				
☑ 830.0417 (1+)																				
☑ 837.1202 (1+)																				
☑ 839.1338 (1+)																				
☑ 855.1078 (1+)																				
☑ 861.1117 (1+)																				
☑ 865.5404 (1+)																				
☑ 868.5782 (1+)																				
☑ 871.0816 (1+)																				
☑ 877.0924 (1+)																				
☑ 880.0422 (1+)																				
☑ 882.6070 (1+)																				
☑ 896.0391 (1+)																				
☑ 903.4150 (1+)																				
☑ 946.0345 (1+)																				
☑ 952.4629 (1+)																				
☑ 1015.5952 (1+)										●	●	●					●			
☑ 1022.1687 (1+)																				
☑ 1022.5059 (1+)																				
☑ 1044.1445 (1+)																				
☑ 1049.5180 (1+)																				
☑ 1064.1066 (1+)																				
☑ 1064.5361 (1+)																				
☑ 1066.1255 (1+)																				
☑ 1068.5260 (1+)																				
☑ 1082.1004 (1+)																				
☑ 1126.5931 (1+)																				
☑ 1149.5092 (1+)																				
☑ 1157.6688 (1+)																			●	
☑ 1161.6596 (1+)																				
☑ 1173.6625 (1+)																				
☑ 1175.6989 (1+)																				
☑ 1179.6574 (1+)																				
☑ 1189.6575 (1+)																				

☑ 1195.6212 (1+)																				
☑ 1205.6570 (1+)																				
☑ 1233.6978 (1+)																				
☑ 1249.1574 (1+)																				
☑ 1252.6580 (1+)																				
☑ 1255.0832 (1+)																				
☑ 1255.6667 (1+)																				
☑ 1267.6429 (1+)																				
☑ 1271.1302 (1+)																				
☑ 1277.7173 (1+)																				
☑ 1285.7642 (1+)																				
☑ 1293.6535 (1+)								●	●											
☑ 1309.6407 (1+)																				
☑ 1311.7849 (1+)																				
☑ 1319.6678 (1+)																				
☑ 1323.6421 (1+)																				
☑ 1325.6405 (1+)																				
☑ 1330.5704 (1+)																				
☑ 1335.6602 (1+)																				
☑ 1340.6439 (1+)																				
☑ 1346.5595 (1+)																				
☑ 1356.5932 (1+)																				
☑ 1370.7905 (1+)																				
☑ 1404.1990 (1+)																				
☑ 1404.7008 (1+)																				
☑ 1408.8268 (1+)																				
☑ 1475.7653 (1+)																				
☑ 1493.7263 (1+)						●	●													
☑ 1497.8121 (1+)																				
☑ 1500.7156 (1+)																				
☑ 1519.7543 (1+)																				
☑ 1522.7117 (1+)																				
☑ 1535.7486 (1+)																				
☑ 1538.6707 (1+)																				
☑ 1592.8193 (1+)	●	●	●	●	●															
☑ 1606.8322 (1+)																				
☑ 1614.8130 (1+)																				
☑ 1625.9006 (1+)																				
☑ 1630.7808 (1+)																				
☑ 1636.7653 (1+)																				
☑ 1698.8360 (1+)																				
☑ 1718.7900 (1+)																				
☑ 1818.0057 (1+)																				
☑ 1957.9524 (1+)																				
☑ 1967.8707 (1+)																				
☑ 1994.0207 (1+)																				
☑ 2134.9888 (1+)																				
☑ 2260.1615 (1+)																				
☑ 2265.2520 (1+)																				
☑ 2277.1374 (1+)																				
☑ 2281.2429 (1+)																				
☑ 2682.1989 (1+)																				
☑ 2914.5586 (1+)																				
☑ 3095.6408 (1+)																				
☑ 3347.7282 (1+)																				

Select All | Select None | Search Selected

Index

	Accession	Mass	Score	Description
1.	VASP1_VIPAA	22635	121	Snake venom serine protease VaSP1 (Fragments) OS=Vipera ammodytes ammodytes OX=8705 PE=1 SV=1
2.	A0A6G5ZVX7_9SAUR	29593	120	Serine protease 7 OS=Vipera anatolica senliki OX=2604287 PE=2 SV=1
3.	A0A6G5ZUR6_9SAUR	29579	120	Serine protease 6 OS=Vipera anatolica senliki OX=2604287 PE=2 SV=1
4.	VSP2_MACLB	29559	120	Venom serine proteinase-like protein 2 OS=Macrovipera lebetina OX=8709 PE=2 SV=1
5.	VSPH1_VIPAA	29593	120	Vaa serine proteinase homolog 1 OS=Vipera ammodytes ammodytes OX=8705 PE=1 SV=1
6.	OXLA_MACLB	12541	75	L-amino-acid oxidase (Fragments) OS=Macrovipera lebetina OX=8709 PE=1 SV=2
7.	OXLA_VIPBB	10345	68	L-amino-acid oxidase (Fragments) OS=Vipera berus berus OX=31156 PE=1 SV=1
8.	OXLA_DABRR	57251	67	L-amino-acid oxidase OS=Daboia russelii OX=8707 PE=1 SV=1
9.	OXLA_VIPAA	55056	46	L-amino-acid oxidase OS=Vipera ammodytes ammodytes OX=8705 PE=1 SV=1
10.	A0A6G5ZVL0_9SAUR	21283	30	Cholinesterase 14 (Fragment) OS=Vipera anatolica senliki OX=2604287 PE=2 SV=1
11.	A0A6G5ZVP1_9SAUR	29798	26	Cholinesterase 8 (Fragment) OS=Vipera anatolica senliki OX=2604287 PE=2 SV=1
12.	A0A1I9KNN1_VIPAA	17843	26	C-type lectin snaclec-3 OS=Vipera ammodytes ammodytes OX=8705 PE=2 SV=1
13.	A0A6B7FPT0_VIPAA	15144	22	Endogenous tripeptide metalloproteinase inhibitor MPi-5 OS=Vipera ammodytes ammodytes OX=8705 PE=2 SV=1
14.	PA2A2_MACLB	16247	21	Acidic phospholipase A2 2 OS=Macrovipera lebetina OX=8709 PE=1 SV=1
15.	A0A1J0CZN1_VIPAA	20176	21	SVMPi-1 OS=Vipera ammodytes ammodytes OX=8705 PE=2 SV=1
16.	A0A1I9KNP4_VIPAA	20248	21	Endogenous tripeptide metalloproteinase inhibitor OS=Vipera ammodytes ammodytes OX=8705 PE=2 SV=1
17.	A0A0C5DKK6_MACLB	18110	21	C-type lectin-like protein 2A OS=Macrovipera lebetina OX=8709 PE=2 SV=1
18.	K4GTV5_DABRR	24685	20	MutS protein 6 (Fragment) OS=Daboia russelii OX=8707 GN=MSH6 PE=4 SV=1
19.	A0A6G5ZW74_9SAUR	32808	19	Veficolin 9 (Fragment) OS=Vipera anatolica senliki OX=2604287 PE=2 SV=1

20. Q8UVG8_DABRR 52931 19 Vimentin OS=Daboia russelii OX=8707 PE=2 SV=1

Results List

1. VASP1_VIPAA **Mass:** 22635 **Score:** 121 **Expect:** 1.1e-009 **Matches:** 3
Snake venom serine protease VaSP1 (Fragments) OS=Vipera ammodytes ammodytes OX=8705 PE=1 SV=1

Observed	Mr(expt)	Mr(calc)	ppm	Start	End	Miss	Ions	Peptide
1189.6575	1188.6502	1188.6325	14.9	85	93	1	---	R.WDKDIMLIR.L
1205.6570	1204.6497	1204.6274	18.5	85	93	1	---	R.WDKDIMLIR.L + Oxidation (M)
1592.8193	1591.8121	1591.7698	26.5	165	179	0	114	R.TLCAGILQGGIDSCK.G

No match to: 807.4626, 817.1426, 830.0417, 837.1202, 839.1338, 855.1078, 861.1117, 865.5404, 868.5782, 871.0816, 877.0924, 880.0422, 882.6070, 896.0391, 903.4150, 946.0345, 952.4629, 1015.5952, 1022.1687, 1022.5059, 1044.1445, 1049.5180, 1064.1066, 1064.5361, 1066.1255, 1068.5260, 1082.1004, 1126.5931, 1149.5092, 1157.6688, 1161.6596, 1173.6625, 1175.6989, 1179.6574, 1195.6212, 1233.6978, 1249.1574, 1252.6580, 1255.0832, 1255.6667, 1267.6429, 1271.1302, 1277.7173, 1285.7642, 1293.6535, 1309.6407, 1311.7849, 1319.6678, 1323.6421, 1325.6405, 1330.5704, 1335.6602, 1340.6439, 1346.5595, 1356.5932, 1370.7905, 1404.1990, 1404.7008, 1408.8268, 1475.7653, 1493.7263, 1497.8121, 1500.7156, 1519.7543, 1522.7117, 1535.7486, 1538.6707, 1606.8322, 1614.8130, 1625.9006, 1630.7808, 1636.7653, 1698.8360, 1718.7900, 1818.0057, 1957.9524, 1967.8707, 1994.0207, 2134.9888, 2260.1615, 2265.2520, 2277.1374, 2281.2429, 2682.1989, 2914.5586, 3095.6408, 3347.7282

2. A0A6G5ZVX7_9SAUR **Mass:** 29593 **Score:** 120 **Expect:** 1.4e-009 **Matches:** 3
Serine protease 7 OS=Vipera anatolica senliki OX=2604287 PE=2 SV=1

Observed	Mr(expt)	Mr(calc)	ppm	Start	End	Miss	Ions	Peptide
1189.6575	1188.6502	1188.6325	14.9	109	117	1	---	R.WDKDIMLIR.L
1205.6570	1204.6497	1204.6274	18.5	109	117	1	---	R.WDKDIMLIR.L + Oxidation (M)
1592.8193	1591.8121	1591.7698	26.5	189	203	0	114	R.TLCAGILQGGIDSCK.V

No match to: 807.4626, 817.1426, 830.0417, 837.1202, 839.1338, 855.1078, 861.1117, 865.5404, 868.5782, 871.0816, 877.0924, 880.0422, 882.6070, 896.0391, 903.4150, 946.0345, 952.4629, 1015.5952, 1022.1687, 1022.5059, 1044.1445, 1049.5180, 1064.1066, 1064.5361, 1066.1255, 1068.5260, 1082.1004, 1126.5931, 1149.5092, 1157.6688, 1161.6596, 1173.6625, 1175.6989, 1179.6574, 1195.6212, 1233.6978, 1249.1574, 1252.6580, 1255.0832, 1255.6667, 1267.6429, 1271.1302, 1277.7173, 1285.7642, 1293.6535, 1309.6407, 1311.7849, 1319.6678, 1323.6421, 1325.6405, 1330.5704, 1335.6602, 1340.6439, 1346.5595, 1356.5932, 1370.7905, 1404.1990, 1404.7008, 1408.8268, 1475.7653, 1493.7263, 1497.8121, 1500.7156, 1519.7543, 1522.7117, 1535.7486, 1538.6707, 1606.8322, 1614.8130, 1625.9006, 1630.7808, 1636.7653, 1698.8360, 1718.7900, 1818.0057, 1957.9524, 1967.8707, 1994.0207, 2134.9888, 2260.1615, 2265.2520, 2277.1374, 2281.2429, 2682.1989, 2914.5586, 3095.6408, 3347.7282

3. A0A6G5ZUR6_9SAUR **Mass:** 29579 **Score:** 120 **Expect:** 1.4e-009 **Matches:** 3
Serine protease 6 OS=Vipera anatolica senliki OX=2604287 PE=2 SV=1

Observed	Mr(expt)	Mr(calc)	ppm	Start	End	Miss	Ions	Peptide
1189.6575	1188.6502	1188.6325	14.9	109	117	1	---	R.WDKDIMLIR.L
1205.6570	1204.6497	1204.6274	18.5	109	117	1	---	R.WDKDIMLIR.L + Oxidation (M)
1592.8193	1591.8121	1591.7698	26.5	189	203	0	114	R.TLCAGILQGGIDSCK.V

No match to: 807.4626, 817.1426, 830.0417, 837.1202, 839.1338, 855.1078, 861.1117, 865.5404, 868.5782, 871.0816, 877.0924, 880.0422, 882.6070, 896.0391, 903.4150, 946.0345, 952.4629, 1015.5952, 1022.1687, 1022.5059, 1044.1445, 1049.5180, 1064.1066, 1064.5361, 1066.1255, 1068.5260, 1082.1004, 1126.5931, 1149.5092, 1157.6688, 1161.6596, 1173.6625, 1175.6989, 1179.6574, 1195.6212, 1233.6978, 1249.1574, 1252.6580, 1255.0832, 1255.6667, 1267.6429, 1271.1302, 1277.7173, 1285.7642, 1293.6535, 1309.6407, 1311.7849, 1319.6678, 1323.6421, 1325.6405, 1330.5704, 1335.6602, 1340.6439, 1346.5595, 1356.5932, 1370.7905, 1404.1990, 1404.7008, 1408.8268, 1475.7653, 1493.7263, 1497.8121, 1500.7156, 1519.7543, 1522.7117, 1535.7486, 1538.6707, 1606.8322, 1614.8130, 1625.9006, 1630.7808, 1636.7653, 1698.8360, 1718.7900, 1818.0057, 1957.9524, 1967.8707, 1994.0207, 2134.9888, 2260.1615, 2265.2520, 2277.1374, 2281.2429, 2682.1989, 2914.5586, 3095.6408, 3347.7282

4. VSP2_MACLB **Mass:** 29559 **Score:** 120 **Expect:** 1.4e-009 **Matches:** 3
Venom serine proteinase-like protein 2 OS=Macrovipera lebetina OX=8709 PE=2 SV=1

Observed	Mr(expt)	Mr(calc)	ppm	Start	End	Miss	Ions	Peptide
1189.6575	1188.6502	1188.6325	14.9	109	117	1	---	R.WDKDIMLIR.L
1205.6570	1204.6497	1204.6274	18.5	109	117	1	---	R.WDKDIMLIR.L + Oxidation (M)
1592.8193	1591.8121	1591.7698	26.5	189	203	0	114	R.TLCAGILQGGIDSCK.V

No match to: 807.4626, 817.1426, 830.0417, 837.1202, 839.1338, 855.1078, 861.1117, 865.5404, 868.5782, 871.0816, 877.0924, 880.0422, 882.6070, 896.0391, 903.4150, 946.0345, 952.4629, 1015.5952, 1022.1687, 1022.5059, 1044.1445, 1049.5180, 1064.1066, 1064.5361, 1066.1255, 1068.5260, 1082.1004, 1126.5931, 1149.5092, 1157.6688, 1161.6596, 1173.6625, 1175.6989, 1179.6574, 1195.6212, 1233.6978, 1249.1574, 1252.6580, 1255.0832, 1255.6667, 1267.6429, 1271.1302, 1277.7173, 1285.7642, 1293.6535, 1309.6407, 1311.7849, 1319.6678, 1323.6421, 1325.6405, 1330.5704, 1335.6602, 1340.6439, 1346.5595, 1356.5932, 1370.7905, 1404.1990, 1404.7008, 1408.8268, 1475.7653, 1493.7263, 1497.8121, 1500.7156, 1519.7543, 1522.7117, 1535.7486, 1538.6707, 1606.8322, 1614.8130, 1625.9006, 1630.7808, 1636.7653, 1698.8360, 1718.7900, 1818.0057, 1957.9524, 1967.8707, 1994.0207, 2134.9888, 2260.1615, 2265.2520, 2277.1374, 2281.2429, 2682.1989, 2914.5586, 3095.6408, 3347.7282

5. VSPH1_VIPAA **Mass:** 29593 **Score:** 120 **Expect:** 1.4e-009 **Matches:** 3
Vaa serine proteinase homolog 1 OS=Vipera ammodytes ammodytes OX=8705 PE=1 SV=1

Observed	Mr(expt)	Mr(calc)	ppm	Start	End	Miss	Ions	Peptide
1189.6575	1188.6502	1188.6325	14.9	109	117	1	---	R.WDKDIMLIR.L
1205.6570	1204.6497	1204.6274	18.5	109	117	1	---	R.WDKDIMLIR.L + Oxidation (M)
1592.8193	1591.8121	1591.7698	26.5	189	203	0	114	R.TLCAGILQGGIDSCK.V

No match to: 807.4626, 817.1426, 830.0417, 837.1202, 839.1338, 855.1078, 861.1117, 865.5404, 868.5782, 871.0816, 877.0924, 880.0422, 882.6070, 896.0391, 903.4150, 946.0345, 952.4629, 1015.5952, 1022.1687, 1022.5059, 1044.1445, 1049.5180, 1064.1066, 1064.5361, 1066.1255, 1068.5260, 1082.1004, 1126.5931, 1149.5092, 1157.6688, 1161.6596, 1173.6625, 1175.6989, 1179.6574, 1195.6212, 1233.6978, 1249.1574, 1252.6580, 1255.0832, 1255.6667, 1267.6429, 1271.1302, 1277.7173, 1285.7642, 1293.6535, 1309.6407, 1311.7849, 1319.6678, 1323.6421, 1325.6405, 1330.5704, 1335.6602, 1340.6439, 1346.5595, 1356.5932, 1370.7905, 1404.1990, 1404.7008, 1408.8268, 1475.7653, 1493.7263, 1497.8121, 1500.7156, 1519.7543, 1522.7117, 1535.7486, 1538.6707, 1606.8322, 1614.8130, 1625.9006, 1630.7808, 1636.7653, 1698.8360, 1718.7900, 1818.0057, 1957.9524, 1967.8707, 1994.0207, 2134.9888, 2260.1615, 2265.2520, 2277.1374, 2281.2429, 2682.1989, 2914.5586, 3095.6408, 3347.7282

6. OXLA_MACLB **Mass:** 12541 **Score:** 75 **Expect:** 4.9e-005 **Matches:** 4
L-amino-acid oxidase (Fragments) OS=Macrovipera lebetina OX=8709 PE=1 SV=2

Observed	Mr(expt)	Mr(calc)	ppm	Start	End	Miss	Ions	Peptide
1064.5361	1063.5288	1063.4757	50.0	5	12	0	---	K.NPLEECFR.E
1126.5931	1125.5858	1125.5012	75.2	58	67	0	---	K.FDEIVGGMDK.K + Oxidation (M)
1493.7263	1492.7190	1492.6616	38.5	1	12	1	47	-.ADDKNPLEECFR.E
1500.7156	1499.7083	1499.6667	27.7	13	24	0	---	R.EDDYEEFLEIAK.N

No match to: 807.4626, 817.1426, 830.0417, 837.1202, 839.1338, 855.1078, 861.1117, 865.5404, 868.5782, 871.0816, 877.0924, 880.0422, 882.6070, 896.0391, 903.4150, 946.0345, 952.4629, 1015.5952, 1022.1687, 1022.5059, 1044.1445, 1049.5180, 1064.1066, 1066.1255, 1068.5260, 1082.1004, 1149.5092, 1157.6688, 1161.6596, 1173.6625, 1175.6989, 1179.6574, 1189.6575, 1195.6212, 1205.6570, 1233.6978, 1249.1574, 1252.6580, 1255.0832, 1255.6667, 1267.6429, 1271.1302, 1277.7173, 1285.7642, 1293.6535, 1309.6407, 1311.7849, 1319.6678, 1323.6421, 1325.6405, 1330.5704, 1335.6602, 1340.6439, 1346.5595, 1356.5932, 1370.7905, 1404.1990, 1404.7008, 1408.8268, 1475.7653, 1497.8121, 1519.7543, 1522.7117, 1535.7486, 1538.6707, 1592.8193, 1606.8322, 1614.8130, 1625.9006, 1630.7808, 1636.7653, 1698.8360, 1718.7900, 1818.0057, 1957.9524, 1967.8707, 1994.0207, 2134.9888, 2260.1615, 2265.2520, 2277.1374, 2281.2429, 2682.1989, 2914.5586, 3095.6408, 3347.7282

7. OXLA_VIPBB **Mass:** 10345 **Score:** 68 **Expect:** 0.00024 **Matches:** 3
L-amino-acid oxidase (Fragments) OS=Vipera berus berus OX=31156 PE=1 SV=1

Observed	Mr(expt)	Mr(calc)	ppm	Start	End	Miss	Ions	Peptide
1064.5361	1063.5288	1063.4757	50.0	5 -	12	0	---	K.NPLEECFR.E
1493.7263	1492.7190	1492.6616	38.5	1 -	12	1	47	-.ADDKNPLEECFR.E
1500.7156	1499.7083	1499.6667	27.7	13 -	24	0	---	R.EDDYEEFLEIAK.N

No match to: 807.4626, 817.1426, 830.0417, 837.1202, 839.1338, 855.1078, 861.1117, 865.5404, 868.5782, 871.0816, 877.0924, 880.0422, 882.6070, 896.0391, 903.4150, 946.0345, 952.4629, 1015.5952, 1022.1687, 1022.5059, 1044.1445, 1049.5180, 1064.1066, 1066.1255, 1068.5260, 1082.1004, 1126.5931, 1149.5092, 1157.6688, 1161.6596, 1173.6625, 1175.6989, 1179.6574, 1189.6575, 1195.6212, 1205.6570, 1233.6978, 1249.1574, 1252.6580, 1255.0832, 1255.6667, 1267.6429, 1271.1302, 1277.7173, 1285.7642, 1293.6535, 1309.6407, 1311.7849, 1319.6678, 1323.6421, 1325.6405, 1330.5704, 1335.6602, 1340.6439, 1346.5595, 1356.5932, 1370.7905, 1404.1990, 1404.7008, 1408.8268, 1475.7653, 1497.8121, 1519.7543, 1522.7117, 1535.7486, 1538.6707, 1592.8193, 1606.8322, 1614.8130, 1625.9006, 1630.7808, 1636.7653, 1698.8360, 1718.7900, 1818.0057, 1957.9524, 1967.8707, 1994.0207, 2134.9888, 2260.1615, 2265.2520, 2277.1374, 2281.2429, 2682.1989, 2914.5586, 3095.6408, 3347.7282

8. OXLA_DABRR **Mass:** 57251 **Score:** 67 **Expect:** 0.00027 **Matches:** 10
L-amino-acid oxidase OS=Daboia russelii OX=8707 PE=1 SV=1

Observed	Mr(expt)	Mr(calc)	ppm	Start	End	Miss	Ions	Peptide
903.4150	902.4077	902.4457	-42.13	497 -	504	1	---	R.DVNRASEL.-
1064.5361	1063.5288	1063.4757	50.0	23 -	30	0	---	K.NPLEECFR.E
1255.6667	1254.6594	1254.6642	-3.81	341 -	351	1	---	R.SGTKIFLTCTK.K
1293.6535	1292.6462	1292.5972	37.9	98 -	108	0	40	K.EGWYANLGPMR.V
1309.6407	1308.6334	1308.5921	31.6	98 -	108	0	---	K.EGWYANLGPMR.V + Oxidation (M)
1370.7905	1369.7832	1369.7314	37.9	77 -	89	0	---	K.VTVLEASERPGGR.V
1500.7156	1499.7083	1499.6667	27.7	31 -	42	0	---	R.EDDYEEFLEIAK.N
1625.9006	1624.8933	1624.9009	-4.65	77 -	91	1	---	K.VTVLEASERPGGRVR.T
1698.8360	1697.8287	1697.9352	-62.71	154 -	169	1	---	K.DPGLLKYPVKPSEAGK.S
1967.8707	1966.8634	1967.0034	-71.16	299 -	315	0	---	K.NLLLETADYVIVCTTSR.A

No match to: 807.4626, 817.1426, 830.0417, 837.1202, 839.1338, 855.1078, 861.1117, 865.5404, 868.5782, 871.0816, 877.0924, 880.0422, 882.6070, 896.0391, 946.0345, 952.4629, 1015.5952, 1022.1687, 1022.5059, 1044.1445, 1049.5180, 1064.1066, 1066.1255, 1068.5260, 1082.1004, 1126.5931, 1149.5092, 1157.6688, 1161.6596, 1173.6625, 1175.6989, 1179.6574, 1189.6575, 1195.6212, 1205.6570, 1233.6978, 1249.1574, 1252.6580, 1255.0832, 1267.6429, 1271.1302, 1277.7173, 1285.7642, 1311.7849, 1319.6678, 1323.6421, 1325.6405, 1330.5704, 1335.6602, 1340.6439, 1346.5595, 1356.5932, 1404.1990, 1404.7008, 1408.8268, 1475.7653, 1493.7263, 1497.8121, 1519.7543, 1522.7117, 1535.7486, 1538.6707, 1592.8193, 1606.8322, 1614.8130, 1630.7808, 1636.7653, 1718.7900, 1818.0057, 1957.9524, 1994.0207, 2134.9888, 2260.1615, 2265.2520, 2277.1374, 2281.2429, 2682.1989, 2914.5586, 3095.6408, 3347.7282

9. OXLA_VIPAA **Mass:** 55056 **Score:** 46 **Expect:** 0.037 **Matches:** 5
L-amino-acid oxidase OS=Vipera ammodytes ammodytes OX=8705 PE=1 SV=1

Observed	Mr(expt)	Mr(calc)	ppm	Start	End	Miss	Ions	Peptide
1064.5361	1063.5288	1063.4757	50.0	3 -	10	0	---	R.NPLEECFR.E
1293.6535	1292.6462	1292.5972	37.9	78 -	88	0	40	K.EGWYANLGPMR.I
1309.6407	1308.6334	1308.5921	31.6	78 -	88	0	---	K.EGWYANLGPMR.I + Oxidation (M)
1335.6602	1334.6529	1334.6037	36.8	1 -	10	1	---	-.DRNPLEECFR.E
1538.6707	1537.6634	1537.7624	-64.34	150 -	164	0	---	K.SAGQLYEESLGSAVK.D

No match to: 807.4626, 817.1426, 830.0417, 837.1202, 839.1338, 855.1078, 861.1117, 865.5404, 868.5782, 871.0816, 877.0924, 880.0422, 882.6070, 896.0391, 903.4150, 946.0345, 952.4629, 1015.5952, 1022.1687, 1022.5059, 1044.1445, 1049.5180, 1064.1066, 1066.1255, 1068.5260, 1082.1004, 1126.5931, 1149.5092, 1157.6688, 1161.6596, 1173.6625, 1175.6989, 1179.6574, 1189.6575, 1195.6212, 1205.6570, 1233.6978, 1249.1574, 1252.6580, 1255.0832, 1255.6667, 1267.6429, 1271.1302, 1277.7173, 1285.7642, 1311.7849, 1319.6678, 1323.6421, 1325.6405, 1330.5704, 1340.6439, 1346.5595, 1356.5932, 1370.7905, 1404.1990, 1404.7008, 1408.8268, 1475.7653, 1493.7263, 1497.8121, 1500.7156, 1519.7543, 1522.7117, 1535.7486, 1592.8193, 1606.8322, 1614.8130, 1625.9006, 1630.7808, 1636.7653, 1698.8360, 1718.7900, 1818.0057, 1957.9524, 1967.8707, 1994.0207, 2134.9888, 2260.1615, 2265.2520, 2277.1374, 2281.2429, 2682.1989, 2914.5586, 3095.6408, 3347.7282

10. A0A6G5ZVL0_9SAUR **Mass:** 21283 **Score:** 30 **Expect:** 1.4 **Matches:** 5
Cholinesterase 14 (Fragment) OS=Vipera anatolica senliki OX=2604287 PE=2 SV=1

Observed	Mr(expt)	Mr(calc)	ppm	Start	End	Miss	Ions	Peptide
952.4629	951.4556	951.5138	-61.10	37 -	45	0	---	K.EVAHAVAQK.F
1015.5952	1014.5879	1014.4957	90.9	56 -	63	0	3	R.LAFSQYMR.D
1205.6570	1204.6497	1204.5935	46.6	125 -	135	0	---	R.SITEAEEALSR.Q
1519.7543	1518.7470	1518.6860	40.2	136 -	146	1	---	R.QMMRYWAQFAR.D + 2 Oxidation (M)
2682.1989	2681.1916	2681.2692	-28.92	56 -	77	1	---	R.LAFSQYMRDYFMVCPLAELAEK.I

No match to: 807.4626, 817.1426, 830.0417, 837.1202, 839.1338, 855.1078, 861.1117, 865.5404, 868.5782, 871.0816, 877.0924, 880.0422, 882.6070, 896.0391, 903.4150, 946.0345, 1022.1687, 1022.5059, 1044.1445, 1049.5180, 1064.1066, 1064.5361, 1066.1255, 1068.5260, 1082.1004, 1126.5931, 1149.5092, 1157.6688, 1161.6596, 1173.6625, 1175.6989, 1179.6574, 1189.6575, 1195.6212, 1233.6978, 1249.1574, 1252.6580, 1255.0832, 1255.6667, 1267.6429, 1271.1302, 1277.7173, 1285.7642, 1293.6535, 1309.6407, 1311.7849, 1319.6678, 1323.6421, 1325.6405, 1330.5704, 1335.6602, 1340.6439, 1346.5595, 1356.5932, 1370.7905, 1404.1990, 1404.7008, 1408.8268, 1475.7653, 1493.7263, 1497.8121, 1500.7156, 1522.7117, 1535.7486, 1538.6707, 1592.8193, 1606.8322, 1614.8130, 1625.9006, 1630.7808, 1636.7653, 1698.8360, 1718.7900, 1818.0057, 1957.9524, 1967.8707, 1994.0207, 2134.9888, 2260.1615, 2265.2520, 2277.1374, 2281.2429, 2914.5586, 3095.6408, 3347.7282

11. A0A6G5ZVP1_9SAUR **Mass:** 29798 **Score:** 26 **Expect:** 3.9 **Matches:** 5
Cholinesterase 8 (Fragment) OS=Vipera anatolica senliki OX=2604287 PE=2 SV=1

Observed	Mr(expt)	Mr(calc)	ppm	Start	End	Miss	Ions	Peptide
952.4629	951.4556	951.5138	-61.10	100 -	108	0	---	K.EVAHAVAQK.F
1015.5952	1014.5879	1014.4957	90.9	119 -	126	0	3	R.LAFSQYMR.D
1205.6570	1204.6497	1204.5935	46.6	188 -	198	0	---	R.SITEAEEALSR.Q
1519.7543	1518.7470	1518.6860	40.2	199 -	209	1	---	R.QMMRYWAQFAR.D + 2 Oxidation (M)
2682.1989	2681.1916	2681.2692	-28.92	119 -	140	1	---	R.LAFSQYMRDYFMVCPLAELAEK.I

No match to: 807.4626, 817.1426, 830.0417, 837.1202, 839.1338, 855.1078, 861.1117, 865.5404, 868.5782, 871.0816, 877.0924, 880.0422, 882.6070, 896.0391, 903.4150, 946.0345, 1022.1687, 1022.5059, 1044.1445, 1049.5180, 1064.1066, 1064.5361, 1066.1255, 1068.5260, 1082.1004, 1126.5931, 1149.5092, 1157.6688, 1161.6596, 1173.6625, 1175.6989, 1179.6574, 1189.6575, 1195.6212, 1233.6978, 1249.1574, 1252.6580, 1255.0832, 1255.6667, 1267.6429, 1271.1302, 1277.7173, 1285.7642, 1293.6535, 1309.6407, 1311.7849, 1319.6678, 1323.6421, 1325.6405, 1330.5704, 1335.6602, 1340.6439, 1346.5595, 1356.5932, 1370.7905, 1404.1990, 1404.7008, 1408.8268, 1475.7653, 1493.7263, 1497.8121, 1500.7156, 1522.7117, 1535.7486, 1538.6707, 1592.8193, 1606.8322, 1614.8130, 1625.9006, 1630.7808, 1636.7653, 1698.8360, 1718.7900, 1818.0057, 1957.9524, 1967.8707, 1994.0207, 2134.9888, 2260.1615, 2265.2520, 2277.1374, 2281.2429, 2914.5586, 3095.6408, 3347.7282

12. A0A1I9KNN1_VIPAA **Mass:** 17843 **Score:** 26 **Expect:** 4 **Matches:** 2
C-type lectin snaclec-3 OS=Vipera ammodytes ammodytes OX=8705 PE=2 SV=1

Observed	Mr(expt)	Mr(calc)	ppm	Start	End	Miss	Ions	Peptide
1015.5952	1014.5879	1014.5399	47.3	87 -	94	0	17	K.YHAWIGLR.D
1630.7808	1629.7735	1629.8012	-16.99	87 -	99	1	---	K.YHAWIGLRDQSER.Q

No match to: 807.4626, 817.1426, 830.0417, 837.1202, 839.1338, 855.1078, 861.1117, 865.5404, 868.5782, 871.0816, 877.0924, 880.0422, 882.6070, 896.0391, 903.4150, 946.0345, 952.4629, 1022.1687, 1022.5059, 1044.1445, 1049.5180, 1064.1066, 1064.5361, 1066.1255, 1068.5260, 1082.1004, 1126.5931, 1149.5092, 1157.6688, 1161.6596, 1173.6625, 1175.6989, 1179.6574, 1189.6575, 1195.6212, 1205.6570, 1233.6978, 1249.1574, 1252.6580, 1255.0832, 1255.6667, 1267.6429, 1271.1302, 1277.7173, 1285.7642, 1293.6535, 1309.6407, 1311.7849, 1319.6678, 1323.6421, 1325.6405, 1330.5704, 1335.6602, 1340.6439, 1346.5595, 1356.5932, 1370.7905, 1404.1990, 1404.7008, 1408.8268, 1475.7653, 1493.7263, 1497.8121, 1500.7156, 1519.7543, 1522.7117, 1535.7486, 1538.6707, 1592.8193, 1606.8322, 1614.8130, 1625.9006, 1636.7653, 1698.8360, 1718.7900, 1818.0057, 1957.9524, 1967.8707, 1994.0207, 2134.9888, 2260.1615, 2265.2520, 2277.1374, 2281.2429,

2682.1989, 2914.5586, 3095.6408, 3347.7282

13. A0A6B7FPT0_VIPAA **Mass:** 15144 **Score:** 22 **Expect:** 9.6 **Matches:** 4

Endogenous tripeptide metalloproteinase inhibitor MPi-5 OS=Vipera ammodytes ammodytes OX=8705 PE=2 SV=1

Observed	Mr(expt)	Mr(calc)	ppm	Start	End	Miss	Ions	Peptide
1064.5361	1063.5288	1063.4981	28.8	82 -	89	0	---	R.MVHHEQQR.G
1475.7653	1474.7581	1474.7562	1.25	113 -	126	1	---	R.IDRIGSVSGLGCNK.V
1493.7263	1492.7190	1492.7106	5.66	79 -	89	1	---	K.WARMVHHEQQR.G + Oxidation (M)
1698.8360	1697.8287	1697.8195	5.42	28 -	41	1	---	K.QKWLGPESPPMEQR.W + Oxidation (M)

No match to: 807.4626, 817.1426, 830.0417, 837.1202, 839.1338, 855.1078, 861.1117, 865.5404, 868.5782, 871.0816, 877.0924, 880.0422, 882.6070, 896.0391, 903.4150, 946.0345, 952.4629, 1015.5952, 1022.1687, 1022.5059, 1044.1445, 1049.5180, 1064.1066, 1066.1255, 1068.5260, 1082.1004, 1126.5931, 1149.5092, 1157.6688, 1161.6596, 1173.6625, 1175.6989, 1179.6574, 1189.6575, 1195.6212, 1205.6570, 1233.6978, 1249.1574, 1252.6580, 1255.0832, 1255.6667, 1267.6429, 1271.1302, 1277.7173, 1285.7642, 1293.6535, 1309.6407, 1311.7849, 1319.6678, 1323.6421, 1325.6405, 1330.5704, 1335.6602, 1340.6439, 1346.5595, 1356.5932, 1370.7905, 1404.1990, 1404.7008, 1408.8268, 1497.8121, 1500.7156, 1519.7543, 1522.7117, 1535.7486, 1538.6707, 1592.8193, 1606.8322, 1614.8130, 1625.9006, 1630.7808, 1636.7653, 1718.7900, 1818.0057, 1957.9524, 1967.8707, 1994.0207, 2134.9888, 2260.1615, 2265.2520, 2277.1374, 2281.2429, 2682.1989, 2914.5586, 3095.6408, 3347.7282

14. PA2A2_MACLB **Mass:** 16247 **Score:** 21 **Expect:** 11 **Matches:** 3

Acidic phospholipase A2 2 OS=Macrovipera lebetina OX=8709 PE=1 SV=1

Observed	Mr(expt)	Mr(calc)	ppm	Start	End	Miss	Ions	Peptide
1957.9524	1956.9451	1956.8485	49.4	123 -	137	1	---	K.YMLYSLFDCKEESEK.C + Oxidation (M)
2682.1989	2681.1916	2681.1374	20.2	77 -	99	0	---	K.LSTYSYSFQNGDIVCGDDDPCLR.A
3095.6408	3094.6335	3094.5620	23.1	1 -	27	1	---	-.MRTLWIVAVWLTGVEGDLSQFGDMINK.K + Oxidation (M)

No match to: 807.4626, 817.1426, 830.0417, 837.1202, 839.1338, 855.1078, 861.1117, 865.5404, 868.5782, 871.0816, 877.0924, 880.0422, 882.6070, 896.0391, 903.4150, 946.0345, 952.4629, 1015.5952, 1022.1687, 1022.5059, 1044.1445, 1049.5180, 1064.1066, 1064.5361, 1066.1255, 1068.5260, 1082.1004, 1126.5931, 1149.5092, 1157.6688, 1161.6596, 1173.6625, 1175.6989, 1179.6574, 1189.6575, 1195.6212, 1205.6570, 1233.6978, 1249.1574, 1252.6580, 1255.0832, 1255.6667, 1267.6429, 1271.1302, 1277.7173, 1285.7642, 1293.6535, 1309.6407, 1311.7849, 1319.6678, 1323.6421, 1325.6405, 1330.5704, 1335.6602, 1340.6439, 1346.5595, 1356.5932, 1370.7905, 1404.1990, 1404.7008, 1408.8268, 1475.7653, 1493.7263, 1497.8121, 1500.7156, 1519.7543, 1522.7117, 1535.7486, 1538.6707, 1592.8193, 1606.8322, 1614.8130, 1625.9006, 1630.7808, 1636.7653, 1698.8360, 1718.7900, 1818.0057, 1967.8707, 1994.0207, 2134.9888, 2260.1615, 2265.2520, 2277.1374, 2281.2429, 2914.5586, 3347.7282

15. A0A1J0CZN1_VIPAA **Mass:** 20176 **Score:** 21 **Expect:** 11 **Matches:** 4

SVMPi-1 OS=Vipera ammodytes ammodytes OX=8705 PE=2 SV=1

Observed	Mr(expt)	Mr(calc)	ppm	Start	End	Miss	Ions	Peptide
1064.5361	1063.5288	1063.4981	28.8	125 -	132	0	---	R.MVHHEQQR.G
1475.7653	1474.7581	1474.7562	1.25	156 -	169	1	---	R.IDRIGSVSGLGCNK.V
1493.7263	1492.7190	1492.7106	5.66	122 -	132	1	---	K.WARMVHHEQQR.G + Oxidation (M)
1698.8360	1697.8287	1697.8195	5.42	28 -	41	1	---	K.QKWLGPESPPMEQR.W + Oxidation (M)

No match to: 807.4626, 817.1426, 830.0417, 837.1202, 839.1338, 855.1078, 861.1117, 865.5404, 868.5782, 871.0816, 877.0924, 880.0422, 882.6070, 896.0391, 903.4150, 946.0345, 952.4629, 1015.5952, 1022.1687, 1022.5059, 1044.1445, 1049.5180, 1064.1066, 1066.1255, 1068.5260, 1082.1004, 1126.5931, 1149.5092, 1157.6688, 1161.6596, 1173.6625, 1175.6989, 1179.6574, 1189.6575, 1195.6212, 1205.6570, 1233.6978, 1249.1574, 1252.6580, 1255.0832, 1255.6667, 1267.6429, 1271.1302, 1277.7173, 1285.7642, 1293.6535, 1309.6407, 1311.7849, 1319.6678, 1323.6421, 1325.6405, 1330.5704, 1335.6602, 1340.6439, 1346.5595, 1356.5932, 1370.7905, 1404.1990, 1404.7008, 1408.8268, 1497.8121, 1500.7156, 1519.7543, 1522.7117, 1535.7486, 1538.6707, 1592.8193, 1606.8322, 1614.8130, 1625.9006, 1630.7808, 1636.7653, 1718.7900, 1818.0057, 1957.9524, 1967.8707, 1994.0207, 2134.9888, 2260.1615, 2265.2520, 2277.1374, 2281.2429, 2682.1989, 2914.5586, 3095.6408, 3347.7282

16. A0A1I9KNP4_VIPAA **Mass:** 20248 **Score:** 21 **Expect:** 11 **Matches:** 4

Endogenous tripeptide metalloproteinase inhibitor OS=Vipera ammodytes ammodytes OX=8705 PE=2 SV=1

Observed	Mr(expt)	Mr(calc)	ppm	Start	End	Miss	Ions	Peptide
1064.5361	1063.5288	1063.4981	28.8	125 -	132	0	---	R.MVHHEQQR.G
1475.7653	1474.7581	1474.7562	1.25	156 -	169	1	---	R.IDRIGSVSGLGCNK.V
1493.7263	1492.7190	1492.7106	5.66	122 -	132	1	---	K.WARMVHHEQQR.G + Oxidation (M)
1698.8360	1697.8287	1697.8195	5.42	28 -	41	1	---	K.QKWLGPESPPMEQR.W + Oxidation (M)

No match to: 807.4626, 817.1426, 830.0417, 837.1202, 839.1338, 855.1078, 861.1117, 865.5404, 868.5782, 871.0816, 877.0924, 880.0422, 882.6070, 896.0391, 903.4150, 946.0345, 952.4629, 1015.5952, 1022.1687, 1022.5059, 1044.1445, 1049.5180, 1064.1066, 1066.1255, 1068.5260, 1082.1004, 1126.5931, 1149.5092, 1157.6688, 1161.6596, 1173.6625, 1175.6989, 1179.6574, 1189.6575, 1195.6212, 1205.6570, 1233.6978, 1249.1574, 1252.6580, 1255.0832, 1255.6667, 1267.6429, 1271.1302, 1277.7173, 1285.7642, 1293.6535, 1309.6407, 1311.7849, 1319.6678, 1323.6421, 1325.6405, 1330.5704, 1335.6602, 1340.6439, 1346.5595, 1356.5932, 1370.7905, 1404.1990, 1404.7008, 1408.8268, 1497.8121, 1500.7156, 1519.7543, 1522.7117, 1535.7486, 1538.6707, 1592.8193, 1606.8322, 1614.8130, 1625.9006, 1630.7808, 1636.7653, 1718.7900, 1818.0057, 1957.9524, 1967.8707, 1994.0207, 2134.9888, 2260.1615, 2265.2520, 2277.1374, 2281.2429, 2682.1989, 2914.5586, 3095.6408, 3347.7282

17. A0A0C5DKK6_MACLB **Mass:** 18110 **Score:** 21 **Expect:** 12 **Matches:** 1

C-type lectin-like protein 2A OS=Macrovipera lebetina OX=8709 PE=2 SV=1

Observed	Mr(expt)	Mr(calc)	ppm	Start	End	Miss	Ions	Peptide
1015.5952	1014.5879	1014.5399	47.3	89 -	96	0	17	K.YHAWIGLR.D

No match to: 807.4626, 817.1426, 830.0417, 837.1202, 839.1338, 855.1078, 861.1117, 865.5404, 868.5782, 871.0816, 877.0924, 880.0422, 882.6070, 896.0391, 903.4150, 946.0345, 952.4629, 1022.1687, 1022.5059, 1044.1445, 1049.5180, 1064.1066, 1064.5361, 1066.1255, 1068.5260, 1082.1004, 1126.5931, 1149.5092, 1157.6688, 1161.6596, 1173.6625, 1175.6989, 1179.6574, 1189.6575, 1195.6212, 1205.6570, 1233.6978, 1249.1574, 1252.6580, 1255.0832, 1255.6667, 1267.6429, 1271.1302, 1277.7173, 1285.7642, 1293.6535, 1309.6407, 1311.7849, 1319.6678, 1323.6421, 1325.6405, 1330.5704, 1335.6602, 1340.6439, 1346.5595, 1356.5932, 1370.7905, 1404.1990, 1404.7008, 1408.8268, 1475.7653, 1493.7263, 1497.8121, 1500.7156, 1519.7543, 1522.7117, 1535.7486, 1538.6707, 1592.8193, 1606.8322, 1614.8130, 1625.9006, 1630.7808, 1636.7653, 1698.8360, 1718.7900, 1818.0057, 1957.9524, 1967.8707, 1994.0207, 2134.9888, 2260.1615, 2265.2520, 2277.1374, 2281.2429, 2682.1989, 2914.5586, 3095.6408, 3347.7282

18. K4GTV5_DABRR **Mass:** 24685 **Score:** 20 **Expect:** 13 **Matches:** 4

MutS protein 6 (Fragment) OS=Daboia russelii OX=8707 GN=MSH6 PE=4 SV=1

Observed	Mr(expt)	Mr(calc)	ppm	Start	End	Miss	Ions	Peptide
1233.6978	1232.6905	1232.5938	78.4	42 -	51	0	---	K.FHVGQFLDDR.H
1285.7642	1284.7569	1284.7037	41.4	73 -	84	1	---	K.GNPSAETQKILK.G
1309.6407	1308.6334	1308.6422	-6.74	196 -	206	1	---	K.SNSVFEKTNQR.M
3095.6408	3094.6335	3094.5355	31.7	168 -	193	1	---	K.CIIDKELLSMANFEEYIPVDVDIVNR.T

No match to: 807.4626, 817.1426, 830.0417, 837.1202, 839.1338, 855.1078, 861.1117, 865.5404, 868.5782, 871.0816, 877.0924, 880.0422, 882.6070, 896.0391, 903.4150, 946.0345, 952.4629, 1015.5952, 1022.1687, 1022.5059, 1044.1445, 1049.5180, 1064.1066, 1064.5361, 1066.1255, 1068.5260, 1082.1004, 1126.5931, 1149.5092, 1157.6688, 1161.6596, 1173.6625, 1175.6989, 1179.6574, 1189.6575, 1195.6212, 1205.6570, 1249.1574, 1252.6580, 1255.0832, 1255.6667, 1267.6429, 1271.1302, 1277.7173, 1293.6535, 1311.7849, 1319.6678, 1323.6421, 1325.6405, 1330.5704, 1335.6602, 1340.6439, 1346.5595, 1356.5932, 1370.7905, 1404.1990, 1404.7008, 1408.8268, 1475.7653, 1493.7263, 1497.8121, 1500.7156, 1519.7543, 1522.7117, 1535.7486, 1538.6707, 1592.8193, 1606.8322, 1614.8130, 1625.9006, 1630.7808, 1636.7653, 1698.8360, 1718.7900, 1818.0057, 1957.9524, 1967.8707, 1994.0207, 2134.9888, 2260.1615, 2265.2520, 2277.1374, 2281.2429, 2682.1989, 2914.5586, 3347.7282

19. A0A6G5ZW74_9SAUR **Mass:** 32808 **Score:** 19 **Expect:** 18 **Matches:** 4

Veficolin 9 (Fragment) OS=Vipera anatolica senliki OX=2604287 PE=2 SV=1

Observed	Mr(expt)	Mr(calc)	ppm	Start	End	Miss	Ions	Peptide

1157.6688	1156.6615	1156.6088	45.6	253 - 262	1	2	K.DKLQDPNSLK.C
1330.5704	1329.5631	1329.6136	-37.96	244 - 254	1	---	K.HSHMPFSTKDK.L + Oxidation (M)
1994.0207	1993.0134	1992.8935	60.2	34 - 54	0	---	R.GCPGMPGSPGPQGPPGFPGAR.G + Oxidation (M)
2134.9888	2133.9816	2133.9837	-1.01	136 - 153	1	---	R.VLCDMHTDGGGWTVFQRR.S

No match to: 807.4626, 817.1426, 830.0417, 837.1202, 839.1338, 855.1078, 861.1117, 865.5404, 868.5782, 871.0816, 877.0924, 880.0422, 882.6070, 896.0391, 903.4150, 946.0345, 952.4629, 1015.5952, 1022.1687, 1022.5059, 1044.1445, 1049.5180, 1064.1066, 1064.5361, 1066.1255, 1068.5260, 1082.1004, 1126.5931, 1149.5092, 1161.6596, 1173.6625, 1175.6989, 1179.6574, 1189.6575, 1195.6212, 1205.6570, 1233.6978, 1249.1574, 1252.6580, 1255.0832, 1255.6667, 1267.6429, 1271.1302, 1277.7173, 1285.7642, 1293.6535, 1309.6407, 1311.7849, 1319.6678, 1323.6421, 1325.6405, 1335.6602, 1340.6439, 1346.5595, 1356.5932, 1370.7905, 1404.1990, 1404.7008, 1408.8268, 1475.7653, 1493.7263, 1497.8121, 1500.7156, 1519.7543, 1522.7117, 1535.7486, 1538.6707, 1592.8193, 1606.8322, 1614.8130, 1625.9006, 1630.7808, 1636.7653, 1698.8360, 1718.7900, 1818.0057, 1957.9524, 1967.8707, 2260.1615, 2265.2520, 2277.1374, 2281.2429, 2682.1989, 2914.5586, 3095.6408, 3347.7282

20. Q8UVG8_DABRR **Mass:** 52931 **Score:** 19 **Expect:** 19 **Matches:** 6

Vimentin OS=Daboia russelii OX=8707 PE=2 SV=1

Observed	Mr(expt)	Mr(calc)	ppm	Start - End	Miss	Ions	Peptide
1161.6596	1160.6523	1160.6302	19.1	116 - 124	1	---	K.VRFLEQQNK.I
1173.6625	1172.6552	1172.7129	-49.17	435 - 444	1	---	R.TLLIKTVETR.D
1309.6407	1308.6334	1308.5986	26.6	278 - 287	0	---	K.NLQEAEEWYK.S
1311.7849	1310.7776	1310.6540	94.3	386 - 396	0	---	K.MALDIEIATYR.K + Oxidation (M)
1408.8268	1407.8195	1407.7470	51.5	36 - 48	0	---	R.YSLGSSIRPSSVR.L
1625.9006	1624.8933	1624.8065	53.4	49 - 63	1	---	R.LVSCPGGIYASKAMR.V + Oxidation (M)

No match to: 807.4626, 817.1426, 830.0417, 837.1202, 839.1338, 855.1078, 861.1117, 865.5404, 868.5782, 871.0816, 877.0924, 880.0422, 882.6070, 896.0391, 903.4150, 946.0345, 952.4629, 1015.5952, 1022.1687, 1022.5059, 1044.1445, 1049.5180, 1064.1066, 1064.5361, 1066.1255, 1068.5260, 1082.1004, 1126.5931, 1149.5092, 1157.6688, 1175.6989, 1179.6574, 1189.6575, 1195.6212, 1205.6570, 1233.6978, 1249.1574, 1252.6580, 1255.0832, 1255.6667, 1267.6429, 1271.1302, 1277.7173, 1285.7642, 1293.6535, 1319.6678, 1323.6421, 1325.6405, 1330.5704, 1335.6602, 1340.6439, 1346.5595, 1356.5932, 1370.7905, 1404.1990, 1404.7008, 1475.7653, 1493.7263, 1497.8121, 1500.7156, 1519.7543, 1522.7117, 1535.7486, 1538.6707, 1592.8193, 1606.8322, 1614.8130, 1630.7808, 1636.7653, 1698.8360, 1718.7900, 1818.0057, 1957.9524, 1967.8707, 1994.0207, 2134.9888, 2260.1615, 2265.2520, 2277.1374, 2281.2429, 2682.1989, 2914.5586, 3095.6408, 3347.7282

Search Parameters

Type of search : MS/MS Ion Search
Enzyme : Trypsin
Fixed modifications : Carbamidomethyl (C)
Variable modifications : Oxidation (M)
Mass values : Monoisotopic
Protein Mass : Unrestricted
Peptide Mass Tolerance : ± 100 ppm
Fragment Mass Tolerance: ± 0.7 Da
Max Missed Cleavages : 1
Instrument type : MALDI-TOF-TOF
Query1 (807.4626,1+) : <no title>
Query2 (817.1426,1+) : <no title>
Query3 (830.0417,1+) : <no title>
Query4 (837.1202,1+) : <no title>
Query5 (839.1338,1+) : <no title>
Query6 (855.1078,1+) : \\PROTEOMICS1\D\DATA\2105\210505_TLIEPOL_1_68252-68266\68252_68266_LPARDO\0_H11\1\855.1078.LIFT\1SREF\PDATA\1\1R
Query7 (861.1117,1+) : <no title>
Query8 (865.5404,1+) : <no title>
Query9 (868.5782,1+) : <no title>
Query10 (871.0816,1+) : <no title>
Query11 (877.0924,1+) : <no title>
Query12 (880.0422,1+) : <no title>
Query13 (882.6070,1+) : <no title>
Query14 (896.0391,1+) : <no title>
Query15 (903.4150,1+) : <no title>
Query16 (946.0345,1+) : <no title>
Query17 (952.4629,1+) : <no title>
Query18 (1015.5952,1+) : \\PROTEOMICS1\D\DATA\2105\210505_TLIEPOL_1_68252-68266\68252_68266_LPARDO\0_H11\1\1015.5952.LIFT\1SREF\PDATA\1\1R
Query19 (1022.1687,1+) : <no title>
Query20 (1022.5059,1+) : <no title>
Query21 (1044.1445,1+) : <no title>
Query22 (1049.5180,1+) : <no title>
Query23 (1064.1066,1+) : <no title>
Query24 (1064.5361,1+) : <no title>
Query25 (1066.1255,1+) : <no title>
Query26 (1068.5260,1+) : <no title>
Query27 (1082.1004,1+) : <no title>
Query28 (1126.5931,1+) : <no title>
Query29 (1149.5092,1+) : <no title>
Query30 (1157.6688,1+) : \\PROTEOMICS1\D\DATA\2105\210505_TLIEPOL_1_68252-68266\68252_68266_LPARDO\0_H11\1\1157.6688.LIFT\1SREF\PDATA\1\1R
Query31 (1161.6596,1+) : <no title>
Query32 (1173.6625,1+) : <no title>
Query33 (1175.6989,1+) : <no title>
Query34 (1179.6574,1+) : <no title>
Query35 (1189.6575,1+) : <no title>
Query36 (1195.6212,1+) : <no title>
Query37 (1205.6570,1+) : <no title>
Query38 (1233.6978,1+) : <no title>
Query39 (1249.1574,1+) : <no title>
Query40 (1252.6580,1+) : <no title>
Query41 (1255.0832,1+) : <no title>
Query42 (1255.6667,1+) : <no title>
Query43 (1267.6429,1+) : <no title>
Query44 (1271.1302,1+) : <no title>
Query45 (1277.7173,1+) : <no title>
Query46 (1285.7642,1+) : <no title>
Query47 (1293.6535,1+) : \\PROTEOMICS1\D\DATA\2105\210505_TLIEPOL_1_68252-68266\68252_68266_LPARDO\0_H11\1\1293.6535.LIFT\1SREF\PDATA\1\1R
Query48 (1309.6407,1+) : <no title>
Query49 (1311.7849,1+) : <no title>
Query50 (1319.6678,1+) : <no title>
Query51 (1323.6421,1+) : <no title>
Query52 (1325.6405,1+) : <no title>
Query53 (1330.5704,1+) : \\PROTEOMICS1\D\DATA\2105\210505_TLIEPOL_1_68252-68266\68252_68266_LPARDO\0_H11\1\1330.5704.LIFT\1SREF\PDATA\1\1R
Query54 (1335.6602,1+) : <no title>
Query55 (1340.6439,1+) : <no title>
Query56 (1346.5595,1+) : <no title>
Query57 (1356.5932,1+) : <no title>
Query58 (1370.7905,1+) : <no title>
Query59 (1404.1990,1+) : <no title>
Query60 (1404.7008,1+) : <no title>
Query61 (1408.8268,1+) : <no title>
Query62 (1475.7653,1+) : <no title>

```
Query63 (1493.7263,1+) : \\PROTEOMICS1\D\DATA\2105\210505_TLIEPOL_1_68252-68266\68252_68266_LPARDO\0_H11\1\1493.7263.LIFT\1SREF\PDATA\1\1R
Query64 (1497.8121,1+) : <no title>
Query65 (1500.7156,1+) : <no title>
Query66 (1519.7543,1+) : <no title>
Query67 (1522.7117,1+) : <no title>
Query68 (1535.7486,1+) : <no title>
Query69 (1538.6707,1+) : <no title>
Query70 (1592.8193,1+) : \\PROTEOMICS1\D\DATA\2105\210505_TLIEPOL_1_68252-68266\68252_68266_LPARDO\0_H11\1\1592.8193.LIFT\1SREF\PDATA\1\1R
Query71 (1606.8322,1+) : <no title>
Query72 (1614.8130,1+) : <no title>
Query73 (1625.9006,1+) : <no title>
Query74 (1630.7808,1+) : <no title>
Query75 (1636.7653,1+) : \\PROTEOMICS1\D\DATA\2105\210505_TLIEPOL_1_68252-68266\68252_68266_LPARDO\0_H11\1\1636.7653.LIFT\1SREF\PDATA\1\1R
Query76 (1698.8360,1+) : <no title>
Query77 (1718.7900,1+) : <no title>
Query78 (1818.0057,1+) : <no title>
Query79 (1957.9524,1+) : <no title>
Query80 (1967.8707,1+) : <no title>
Query81 (1994.0207,1+) : <no title>
Query82 (2134.9888,1+) : <no title>
Query83 (2260.1615,1+) : <no title>
Query84 (2265.2520,1+) : <no title>
Query85 (2277.1374,1+) : <no title>
Query86 (2281.2429,1+) : <no title>
Query87 (2682.1989,1+) : <no title>
Query88 (2914.5586,1+) : <no title>
Query89 (3095.6408,1+) : <no title>
Query90 (3347.7282,1+) : <no title>
```

Mascot: http://www.matrixscience.com/

Mascot Search Results

Fr6-6

```
User          :
Email         :
Search title  : vipera_All_8IDplus4 file:\\Proteomics1\d\data\2105\210505_tliepol_1_68252-68266\68252_68266_lpardo\0_G12\1\1SRef\pdata\1\peaklist.xml
Database      : vipera  (1414 sequences; 347452 residues)
Timestamp     : 5 May 2021 at 09:37:36 GMT
Top Score     : 104 for PA2A1_MACLB, Acidic phospholipase A2 1 OS=Macrovipera lebetina OX=8709 PE=1 SV=1
```

Mascot Score Histogram

Protein score is -10*Log(P), where P is the probability that the observed match is a random event.
Protein scores greater than 44 are significant (p<0.05).

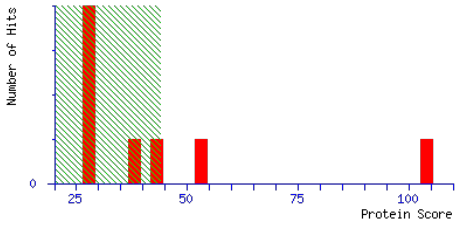

Concise Protein Summary Report

Format As | Concise Protein Summary | Help

Significance threshold p< 0.05 Max. number of hits 10

Re-Search All | Search Unmatched

1. PA2A1_MACLB **Mass:** 16337 **Score:** **104** **Expect:** 5.6e-008 **Matches:** 10
Acidic phospholipase A2 1 OS=Macrovipera lebetina OX=8709 PE=1 SV=1
PA2A2_MACLB **Mass:** 16247 **Score:** **70** **Expect:** 0.00014 **Matches:** 7
Acidic phospholipase A2 2 OS=Macrovipera lebetina OX=8709 PE=1 SV=1
PA2A_MACLN **Mass:** 16243 **Score:** **55** **Expect:** 0.0041 **Matches:** 6
Acidic phospholipase A2 MVL-PLA2 OS=Macrovipera lebetina transmediterranea OX=384075 PE=1 SV=1
Q6A3G8_VIPAA **Mass:** 15517 **Score:** 41 **Expect:** 0.11 **Matches:** 6
Ammodytin I1(A) variant OS=Vipera ammodytes ammodytes OX=8705 PE=2 SV=1
Q6A3G4_VIPAM **Mass:** 16280 **Score:** 40 **Expect:** 0.13 **Matches:** 6
Ammodytin I1(D) isoform OS=Vipera ammodytes montandoni OX=235554 PE=2 SV=1
Q6A3N0_VIPAM **Mass:** 16279 **Score:** 40 **Expect:** 0.13 **Matches:** 6
Ammodytin I1(A) isoform OS=Vipera ammodytes ruffoi OX=246549 PE=2 SV=1
Q6A3G5_VIPAE **Mass:** 16280 **Score:** 40 **Expect:** 0.13 **Matches:** 6
Ammodytin I1(D) isoform OS=Vipera ammodytes meridionalis OX=73841 PE=2 SV=1
Q6YC90_VIPAS **Mass:** 16279 **Score:** 40 **Expect:** 0.13 **Matches:** 6
Ammodytin I1 isoform 2 OS=Vipera aspis OX=8706 GN=AmtI1 PE=3 SV=1
Q6A3K8_VIPAP **Mass:** 16267 **Score:** 40 **Expect:** 0.13 **Matches:** 6
Ammodytin I1(A') isoform OS=Vipera aspis aspis OX=194601 PE=2 SV=1
Q6A3K4_VIPAP **Mass:** 16252 **Score:** 40 **Expect:** 0.13 **Matches:** 6
Ammodytin I1(A) variant OS=Vipera aspis aspis OX=194601 PE=2 SV=1
Q6A3K7_VIPAP **Mass:** 16279 **Score:** 40 **Expect:** 0.13 **Matches:** 6
Ammodytin I1(A) isoform OS=Vipera aspis aspis OX=194601 PE=2 SV=1
PA2A1_VIPAA **Mass:** 16279 **Score:** 40 **Expect:** 0.13 **Matches:** 6
Acidic phospholipase A2 ammodytin I1 OS=Vipera ammodytes ammodytes OX=8705 PE=2 SV=1
Q7T1D1_VIPBB **Mass:** 16258 **Score:** 40 **Expect:** 0.15 **Matches:** 6
Ammodytin I1 OS=Vipera berus berus OX=31156 GN=AmtI1 PE=2 SV=1
Q6A3K0_VIPBB **Mass:** 16214 **Score:** 40 **Expect:** 0.15 **Matches:** 6
Ammodytin I1(B') variant OS=Vipera berus berus OX=31156 PE=2 SV=1
Q1RP80_VIPBN **Mass:** 16258 **Score:** 40 **Expect:** 0.15 **Matches:** 6
Phospholipase A2 ammodytin I1 OS=Vipera berus nikolskii OX=1808362 GN=I1 PE=2 SV=1
Q6A3M8_VIPAM **Mass:** 16378 **Score:** 40 **Expect:** 0.15 **Matches:** 6
Ammodytin I1(A) variant OS=Vipera ammodytes ruffoi OX=246549 PE=2 SV=1
Q6A3I8_VIPUR **Mass:** 16290 **Score:** 39 **Expect:** 0.18 **Matches:** 6
Ammodytin I1(B) isoform OS=Vipera ursinii OX=103942 PE=2 SV=1
Q6A3M9_VIPAM **Mass:** 16286 **Score:** 37 **Expect:** 0.27 **Matches:** 6
Ammodytin I1(B) isoform OS=Vipera ammodytes ruffoi OX=246549 PE=2 SV=1
Q6A3M4_VIPAP **Mass:** 16217 **Score:** 28 **Expect:** 2.3 **Matches:** 4
Ammodytin I1(C) variant OS=Vipera aspis aspis OX=194601 PE=2 SV=1

---

2. PA2A_DABPA **Mass:** 16277 **Score:** **55** **Expect:** 0.0041 **Matches:** 7
Acidic phospholipase A2 VpaPLA2 OS=Daboia palaestinae OX=1170828 PE=1 SV=1

---

3. PA2A7_DABPA **Mass:** 16190 **Score:** 42 **Expect:** 0.085 **Matches:** 4
Acidic phospholipase A2 VP7 OS=Daboia palaestinae OX=1170828 PE=3 SV=1

---

4. Q6A3H1_VIPAS **Mass:** 16292 **Score:** 40 **Expect:** 0.13 **Matches:** 7
Ammodytin I1(C) variant OS=Vipera aspis atra OX=246182 PE=2 SV=1
Q6A3I9_VIPBB **Mass:** 16187 **Score:** 28 **Expect:** 2.3 **Matches:** 5
Ammodytin I1(C) variant OS=Vipera berus berus OX=31156 PE=2 SV=1
Q6A3H5_VIPAS **Mass:** 16245 **Score:** 28 **Expect:** 2.3 **Matches:** 5
Ammodytin I1(F) variant OS=Vipera aspis atra OX=246182 PE=2 SV=1

---

5. PA2BA_VIPRE **Mass:** 2608 **Score:** 29 **Expect:** 1.7 **Matches:** 3
Basic phospholipase A2 homolog Vur-S49 analog (Fragment) OS=Vipera renardi OX=927686 PE=1 SV=1

---

6. PA2A8_DABPA **Mass:** 16194 **Score:** 28 **Expect:** 2.2 **Matches:** 4
Acidic phospholipase A2 VP8 OS=Daboia palaestinae OX=1170828 PE=3 SV=1

---

7. Q6A3C6_VIPAM **Mass:** 16212 **Score:** 28 **Expect:** 2.3 **Matches:** 5

Ammodytin I2(A) isoform OS=Vipera ammodytes ruffoi OX=246549 PE=2 SV=1

---

8. Q6A3J3_VIPBB **Mass:** 16280 **Score:** 28 **Expect:** 2.3 **Matches:** 6
Ammodytin I1(C) variant OS=Vipera berus berus OX=31156 PE=2 SV=1
Q6A3H6_VIPAS **Mass:** 16258 **Score:** 28 **Expect:** 2.3 **Matches:** 6
Ammodytin I1(C) isoform OS=Vipera aspis atra OX=246182 PE=2 SV=1
Q6A3J2_VIPBB **Mass:** 16244 **Score:** 28 **Expect:** 2.3 **Matches:** 6
Ammodytin I1(C) variant OS=Vipera berus berus OX=31156 PE=2 SV=1
Q6A3J0_VIPBB **Mass:** 16280 **Score:** 28 **Expect:** 2.3 **Matches:** 6
Ammodytin I1(C) variant OS=Vipera berus berus OX=31156 PE=2 SV=1
Q6A3H7_VIPAS **Mass:** 16244 **Score:** 28 **Expect:** 2.3 **Matches:** 6
Ammodytin I1(F) isoform OS=Vipera aspis atra OX=246182 PE=2 SV=1
Q6A3H4_VIPAS **Mass:** 16244 **Score:** 28 **Expect:** 2.3 **Matches:** 6
Ammodytin I1(C) isoform OS=Vipera aspis atra OX=246182 PE=2 SV=1
Q7T1D3_VIPAS **Mass:** 16258 **Score:** 28 **Expect:** 2.3 **Matches:** 6
Ammodytin I1 isoform 1 OS=Vipera aspis OX=8706 GN=AmtI1 PE=3 SV=1
Q6A3I1_VIPAP **Mass:** 16288 **Score:** 28 **Expect:** 2.3 **Matches:** 6
Ammodytin I1(C) variant OS=Vipera aspis aspis OX=194601 PE=2 SV=1
Q6A3I0_VIPAP **Mass:** 16272 **Score:** 28 **Expect:** 2.3 **Matches:** 6
Ammodytin I1(C) variant OS=Vipera aspis aspis OX=194601 PE=2 SV=1
Q6A3H8_VIPAP **Mass:** 16330 **Score:** 28 **Expect:** 2.3 **Matches:** 6
Ammodytin I1(C) variant OS=Vipera aspis aspis OX=194601 PE=2 SV=1
Q6A3I3_VIPAP **Mass:** 16286 **Score:** 28 **Expect:** 2.3 **Matches:** 6
Ammodytin I1(C) isoform OS=Vipera aspis aspis OX=194601 PE=2 SV=1
Q6A3M5_VIPAP **Mass:** 16257 **Score:** 28 **Expect:** 2.3 **Matches:** 6
Ammodytin I1(C) variant OS=Vipera aspis aspis OX=194601 PE=2 SV=1
Q6A3G1_VIPAP **Mass:** 16246 **Score:** 28 **Expect:** 2.3 **Matches:** 6
Ammodytin I1(C') isoform OS=Vipera aspis aspis OX=194601 PE=2 SV=1
Q6A3F7_VIPAP **Mass:** 16310 **Score:** 28 **Expect:** 2.3 **Matches:** 6
Ammodytin I1(C) variant OS=Vipera aspis aspis OX=194601 PE=2 SV=1
Q7T1D4_VIPAP **Mass:** 16258 **Score:** 28 **Expect:** 2.3 **Matches:** 6
Ammodytin I1 OS=Vipera aspis aspis OX=194601 GN=AmtI1 PE=2 SV=1
Q7T1D2_VIPAZ **Mass:** 16258 **Score:** 28 **Expect:** 2.3 **Matches:** 6
Ammodytin I1 OS=Vipera aspis zinnikeri OX=55427 GN=AmtI1 PE=2 SV=1
Q6A3J4_VIPBB **Mass:** 16258 **Score:** 28 **Expect:** 2.3 **Matches:** 6
Ammodytin I1(C) isoform OS=Vipera berus berus OX=31156 PE=2 SV=1
Q6A3J6_VIPAZ **Mass:** 16330 **Score:** 28 **Expect:** 2.3 **Matches:** 6
Ammodytin I1(C) isoform OS=Vipera aspis zinnikeri OX=55427 PE=2 SV=1
Q6A3I4_VIPAP **Mass:** 16272 **Score:** 28 **Expect:** 2.3 **Matches:** 5
Ammodytin I1(C) variant OS=Vipera aspis aspis OX=194601 PE=2 SV=1
Q6A3K9_VIPAP **Mass:** 16202 **Score:** 28 **Expect:** 2.3 **Matches:** 5
Ammodytin I1(E) variant OS=Vipera aspis aspis OX=194601 PE=2 SV=1
Q6A3L1_VIPAP **Mass:** 16272 **Score:** 28 **Expect:** 2.3 **Matches:** 5
Ammodytin I1(E) isoform OS=Vipera aspis aspis OX=194601 PE=2 SV=1
Q6A3L0_VIPAP **Mass:** 16360 **Score:** 28 **Expect:** 2.3 **Matches:** 5
Ammodytin I1(E) variant OS=Vipera aspis aspis OX=194601 PE=2 SV=1
Q6A3H2_VIPAS **Mass:** 16198 **Score:** 28 **Expect:** 2.3 **Matches:** 5
Ammodytin I1(C) variant OS=Vipera aspis atra OX=246182 PE=2 SV=1

Search Parameters

Type of search : Peptide Mass Fingerprint
Enzyme : Trypsin
Fixed modifications : Carbamidomethyl (C)
Variable modifications : Oxidation (M)
Mass values : Monoisotopic
Protein Mass : Unrestricted
Peptide Mass Tolerance : ± 100 ppm
Peptide Charge State : 1+
Max Missed Cleavages : 1
Number of queries : 90
Selected for scoring : 36

Mascot: http://www.matrixscience.com/

Mascot Search Results

**User**	:	
**Email**	:	
**Search title**	:	**vipera_all_Lift file:\\Proteomics1\d\data\2105\210505_tliepol_1_68252-68266\68252_68266_lpardo\0_G12\1\0_G12.xml**
**Database**	:	**vipera (1414 sequences; 347452 residues)**
**Timestamp**	:	**5 May 2021 at 10:05:58 GMT**
**Protein hits**	:	PA2A1_MACLB Acidic phospholipase A2 1 OS=Macrovipera lebetina OX=8709 PE=1 SV=1
		Q6A3H1_VIPAS Ammodytin I1(C) variant OS=Vipera aspis atra OX=246182 PE=2 SV=1
		PA2A2_DABRR Acidic phospholipase A2 Drk-a2 OS=Daboia russelii OX=8707 PE=2 SV=1
		A0A223PK33_DABRR Beta-nerve growth factor OS=Daboia russelii OX=8707 PE=2 SV=1
		A0A223PK29_DABRR Disintegrin and metalloproteinase domain-containing protein 19 (Fragment) OS=Daboia russelii OX=8707 PE=2 SV=1
		A0A0H3Y3P3_9PERO Cardiac muscle myosin heavy chain 6 (Fragment) OS=Echiichthys vipera OX=94984 GN=myh6 PE=3 SV=1
		PA2BS_VIPRE Basic phospholipase A2 homolog Vur-S49 OS=Vipera renardi OX=927686 PE=2 SV=1
		A0A223PK32_DABRR Atrial natriuretic peptide receptor 3 (Fragment) OS=Daboia russelii OX=8707 PE=2 SV=1
		A0A223PK16_DABRR C-type lectin domain family 11 member A (Fragment) OS=Daboia russelii OX=8707 PE=2 SV=1
		VM3VB_MACLB Zinc metalloproteinase-disintegrin-like VLAIP-B OS=Macrovipera lebetina OX=8709 PE=1 SV=1

Mascot Score Histogram

Ions score is -10*Log(P), where P is the probability that the observed match is a random event.
Individual ions scores > 14 indicate identity or extensive homology (p<0.05).
Protein scores are derived from ions scores as a non-probabilistic basis for ranking protein hits.

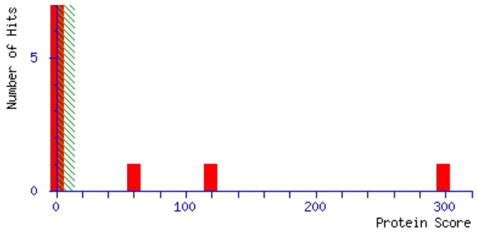

Peptide Summary Report

Format As [Peptide Summary] Help

Significance threshold p< [0.05] Max. number of hits [10]

Standard scoring ○ MudPIT scoring ◉ Ions score or expect cut-off [0] Show sub-sets [0]

Show pop-ups ◉ Suppress pop-ups ○ Sort unassigned [Decreasing Score] Require bold red ☐

Overview Table

Click on column header to jump to entry in results list.
Move mouse over any indicator to highlight identical peptides.
Click on an indicator to see details of individual match.
Use check boxes to select sub-set of queries for new search.

Mouse over: [-Query-]
[-Accession-] [-Sequence-]

Hit:	1	2	3	4	5	6	7	8	9	10
☑ 817.1395 (1+)										
☑ 837.1000 (1+)										
☑ 839.1118 (1+)										
☑ 852.3774 (1+)										
☑ 855.0882 (1+)										
☑ 859.0799 (1+)										
☑ 861.0964 (1+)										
☑ 868.5738 (1+)										
☑ 871.0597 (1+)										
☑ 874.0428 (1+)										
☑ 877.0680 (1+)										
☑ 882.6029 (1+)										
☑ 903.4152 (1+)										
☑ 905.5111 (1+)										
☑ 909.3941 (1+)										
☑ 987.5256 (1+)	●		●				●			
☑ 1022.1471 (1+)										
☑ 1044.1224 (1+)										
☑ 1064.0902 (1+)										
☑ 1066.1043 (1+)										
☑ 1082.0794 (1+)										
☑ 1149.5023 (1+)										
☑ 1157.6635 (1+)										
☑ 1175.6978 (1+)										
☑ 1201.6609 (1+)										
☑ 1222.6157 (1+)										
☑ 1249.1322 (1+)										
☑ 1252.6490 (1+)										

☑ 1267.6375 (1+)										
☑ 1271.1084 (1+)										
☑ 1283.6283 (1+)										
☑ 1289.6181 (1+)										
☑ 1293.6475 (1+)										
☑ 1305.5904 (1+)										
☑ 1309.6407 (1+)										
☑ 1320.6258 (1+)										
☑ 1323.6473 (1+)										
☑ 1339.6437 (1+)	●	●	●							
☑ 1345.6280 (1+)										
☑ 1355.6382 (1+)	●		●							
☑ 1361.6097 (1+)										
☑ 1365.6708 (1+)										
☑ 1377.5960 (1+)										
☑ 1393.5844 (1+)										
☑ 1395.7311 (1+)										
☑ 1411.7256 (1+)										
☑ 1436.7603 (1+)										
☑ 1475.7776 (1+)										
☑ 1476.1123 (1+)										
☑ 1557.8703 (1+)										
☑ 1636.7539 (1+)										
☑ 1707.8181 (1+)										
☑ 1718.7880 (1+)										
☑ 1732.8009 (1+)	●	●								
☑ 1734.7794 (1+)										
☑ 1740.7760 (1+)										
☑ 1744.8013 (1+)										
☑ 1748.7978 (1+)	●	●								
☑ 1754.7913 (1+)										
☑ 1756.7547 (1+)										
☑ 1770.7537 (1+)										
☑ 1772.7239 (1+)										
☑ 1838.9638 (1+)										
☑ 1851.8058 (1+)										
☑ 1862.8756 (1+)										
☑ 1876.8772 (1+)										
☑ 1921.8557 (1+)										
☑ 1924.8861 (1+)										
☑ 1941.8985 (1+)	●					●				●
☑ 1948.8578 (1+)										
☑ 1967.8661 (1+)										
☑ 2005.8049 (1+)										
☑ 2118.8044 (1+)										
☑ 2170.8299 (1+)										
☑ 2217.9647 (1+)								●	●	
☑ 2234.8584 (1+)	●			●	●					
☑ 2255.8811 (1+)										
☑ 2265.2040 (1+)										
☑ 2277.1368 (1+)										
☑ 2566.1425 (1+)										
☑ 2618.1737 (1+)										
☑ 2625.1754 (1+)										
☑ 2636.1875 (1+)										
☑ 2663.1955 (1+)										
☑ 2682.2062 (1+)	●									
☑ 2695.2126 (1+)										
☑ 2708.2152 (1+)										
☑ 2720.1459 (1+)										
☑ 2724.2164 (1+)										
☑ 3347.7264 (1+)										

Select All | Select None | Search Selected | ☐ **Error tolerant** | Archive Report

1. PA2A1_MACLB **Mass:** 16337 **Score:** 298 **Matches:** 8(6) **Sequences:** 6(5)
Acidic phospholipase A2 1 OS=Macrovipera lebetina OX=8709 PE=1 SV=1
☐ Check to include this hit in error tolerant search or archive report

Query	Observed	Mr(expt)	Mr(calc)	ppm	Miss	Score	Expect	Rank	Unique	Peptide
16	987.5256	986.5184	986.4781	40.8	0	3	1.7	1		K.GKPQDATDR.C
38	1339.6437	1338.6364	1338.5988	28.1	0	56	3.6e-006	1	U	K.YMLYSLFDCK.E
40	1355.6382	1354.6310	1354.5937	27.5	0	(11)	0.13	1	U	K.YMLYSLFDCK.E + Oxidation (M)
54	1732.8009	1731.7936	1731.7596	19.6	0	87	5.2e-009	1		R.VAAICFGENMNTYDK.K
58	1748.7978	1747.7906	1747.7545	20.6	0	(55)	1.1e-005	1		R.VAAICFGENMNTYDK.K + Oxidation (M)
69	1941.8985	1940.8912	1940.8536	19.4	1	44	4e-005	1	U	K.YMLYSLFDCKEESEK.C
76	2234.8584	2233.8511	2233.8108	18.0	0	31	0.00088	1	U	R.CCFVHDCCYGSVNGCDPK.L
85	2682.2062	2681.1989	2681.1374	22.9	0	98	1.5e-010	1	U	K.LSTYSYSFQNGDIVCGDDDPCLR.A

2. Q6A3H1_VIPAS **Mass:** 16292 **Score:** 125 **Matches:** 3(2) **Sequences:** 2(1)
Ammodytin I1(C) variant OS=Vipera aspis atra OX=246182 PE=2 SV=1
☐ Check to include this hit in error tolerant search or archive report

Query	Observed	Mr(expt)	Mr(calc)	ppm	Miss	Score	Expect	Rank	Unique	Peptide
38	1339.6437	1338.6364	1338.5988	28.1	0	4	0.64	3	U	K.YMFYSLLDCK.E
54	1732.8009	1731.7936	1731.7596	19.6	0	87	5.2e-009	1		R.VAAICFGENMNTYDK.K
58	1748.7978	1747.7906	1747.7545	20.6	0	(55)	1.1e-005	1		R.VAAICFGENMNTYDK.K + Oxidation (M)

3. PA2A2_DABRR **Mass:** 16430 **Score:** 56 **Matches:** 3(1) **Sequences:** 2(1)
Acidic phospholipase A2 Drk-a2 OS=Daboia russelii OX=8707 PE=2 SV=1
☐ Check to include this hit in error tolerant search or archive report

Query	Observed	Mr(expt)	Mr(calc)	ppm	Miss	Score	Expect	Rank	Unique	Peptide
16	987.5256	986.5184	986.4781	40.8	0	3	1.7	1		K.GKPQDATDR.C
38	1339.6437	1338.6364	1338.5988	28.1	0	56	3.6e-006	1	U	K.YMLYSIFDCK.E
40	1355.6382	1354.6310	1354.5937	27.5	0	(11)	0.13	1	U	K.YMLYSIFDCK.E + Oxidation (M)

4. A0A223PK33_DABRR **Score:** 0 **Matches:** 1(0) **Sequences:** 1(0)
Beta-nerve growth factor OS=Daboia russelii OX=8707 PE=2 SV=1
☐ Check to include this hit in error tolerant search or archive report

Query	Observed	Mr(expt)	Mr(calc)	ppm	Miss	Score	Expect	Rank	Unique	Peptide
76	2234.8584	2233.8511	2234.0563	-91.86	1	10	0.11	2	U	R.DEQSVEFLDNADSLNRNIR.A

Proteins matching the same set of peptides:
NGFV_MACLB **Score:** 0 **Matches:** 1(0) **Sequences:** 1(0)
B8QCI0_DABRR **Score:** 0 **Matches:** 1(0) **Sequences:** 1(0)
V9I1B5_DABRR **Score:** 0 **Matches:** 1(0) **Sequences:** 1(0)
V9I1K1_DABRR **Score:** 0 **Matches:** 1(0) **Sequences:** 1(0)

5. A0A223PK29_DABRR **Score:** 0 **Matches:** 1(0) **Sequences:** 1(0)
Disintegrin and metalloproteinase domain-containing protein 19 (Fragment) OS=Daboia russelii OX=8707 PE=2 SV=1
☐ Check to include this hit in error tolerant search or archive report

Query	Observed	Mr(expt)	Mr(calc)	ppm	Miss	Score	Expect	Rank	Unique	Peptide
76	2234.8584	2233.8511	2234.0579	-92.55	0	8	0.16	3	U	K.CDISENLHSTLWSFLAWR.R

6. A0A0H3Y3P3_9PERO **Score:** 0 **Matches:** 1(0) **Sequences:** 1(0)
Cardiac muscle myosin heavy chain 6 (Fragment) OS=Echiichthys vipera OX=94984 GN=myh6 PE=3 SV=1
☐ Check to include this hit in error tolerant search or archive report

Query	Observed	Mr(expt)	Mr(calc)	ppm	Miss	Score	Expect	Rank	Unique	Peptide
69	1941.8985	1940.8912	1940.9423	-26.33	1	5	0.3	2	U	K.MGVYKLTGAIMHYGNMR.F

7. PA2BS_VIPRE **Mass:** 16504 **Score:** 0 **Matches:** 1(0) **Sequences:** 1(0)
Basic phospholipase A2 homolog Vur-S49 OS=Vipera renardi OX=927686 PE=2 SV=1
☐ Check to include this hit in error tolerant search or archive report

Query	Observed	Mr(expt)	Mr(calc)	ppm	Miss	Score	Expect	Rank	Unique	Peptide
16	987.5256	986.5184	986.5145	3.91	1	3	1.7	1	U	K.GKPKDATDR.C

Proteins matching the same set of peptides:
PA2HL_VIPAA **Mass:** 16481 **Score:** 0 **Matches:** 1(0) **Sequences:** 1(0)
Basic phospholipase A2 homolog ammodytin L OS=Vipera ammodytes ammodytes OX=8705 PE=1 SV=1
Q6A368_VIPAM **Mass:** 16489 **Score:** 0 **Matches:** 1(0) **Sequences:** 1(0)
Ammodytin L(3) variant OS=Vipera ammodytes montandoni OX=235554 PE=2 SV=1
Q6A369_VIPAM **Mass:** 16449 **Score:** 0 **Matches:** 1(0) **Sequences:** 1(0)
Ammodytin L(3) variant OS=Vipera ammodytes montandoni OX=235554 PE=2 SV=1
Q6A370_VIPAM **Mass:** 16461 **Score:** 0 **Matches:** 1(0) **Sequences:** 1(0)
Ammodytin L(3) variant OS=Vipera ammodytes montandoni OX=235554 PE=2 SV=1
Q6A371_VIPAM **Mass:** 16461 **Score:** 0 **Matches:** 1(0) **Sequences:** 1(0)
Ammodytin L(3) isoform OS=Vipera ammodytes montandoni OX=235554 PE=2 SV=1
Q6A374_VIPAA **Mass:** 16399 **Score:** 0 **Matches:** 1(0) **Sequences:** 1(0)
Ammodytin L(1'') variant OS=Vipera ammodytes ammodytes OX=8705 PE=2 SV=1
Q6A378_VIPAA **Mass:** 16459 **Score:** 0 **Matches:** 1(0) **Sequences:** 1(0)
Ammodytin L(1'') isoform OS=Vipera ammodytes ammodytes OX=8705 PE=2 SV=1
Q6A380_VIPAA **Mass:** 16415 **Score:** 0 **Matches:** 1(0) **Sequences:** 1(0)
Ammodytin L(1'') variant OS=Vipera ammodytes ammodytes OX=8705 PE=2 SV=1
Q6A381_VIPAA **Mass:** 16433 **Score:** 0 **Matches:** 1(0) **Sequences:** 1(0)
Ammodytin L(1'') variant OS=Vipera ammodytes ammodytes OX=8705 PE=2 SV=1
Q6A382_VIPAA **Mass:** 16431 **Score:** 0 **Matches:** 1(0) **Sequences:** 1(0)
Ammodytin L(1'') variant OS=Vipera ammodytes ammodytes OX=8705 PE=2 SV=1
Q6A383_VIPAA **Mass:** 16503 **Score:** 0 **Matches:** 1(0) **Sequences:** 1(0)
Ammodytin L(1') isoform OS=Vipera ammodytes ammodytes OX=8705 PE=2 SV=1
Q6A385_VIPBB **Mass:** 16442 **Score:** 0 **Matches:** 1(0) **Sequences:** 1(0)
Ammodytin L(2) variant OS=Vipera berus berus OX=31156 PE=2 SV=1
Q6A386_VIPAM **Mass:** 16504 **Score:** 0 **Matches:** 1(0) **Sequences:** 1(0)
Ammodytin L(4) isoform OS=Vipera ammodytes ruffoi OX=246549 PE=2 SV=1
Q6A387_VIPAM **Mass:** 16471 **Score:** 0 **Matches:** 1(0) **Sequences:** 1(0)
Ammodytin L(1) isoform OS=Vipera ammodytes ruffoi OX=246549 PE=2 SV=1
Q6A388_VIPBB **Mass:** 16415 **Score:** 0 **Matches:** 1(0) **Sequences:** 1(0)
Ammodytin L(2) variant OS=Vipera berus berus OX=31156 PE=2 SV=1
Q6A389_VIPBB **Mass:** 16410 **Score:** 0 **Matches:** 1(0) **Sequences:** 1(0)
Ammodytin L(2) variant OS=Vipera berus berus OX=31156 PE=2 SV=1
Q6A390_VIPBB **Mass:** 16398 **Score:** 0 **Matches:** 1(0) **Sequences:** 1(0)

Ammodytin L(2) variant OS=Vipera berus berus OX=31156 PE=2 SV=1
Q6A391_VIPBB **Mass:** 16411 **Score:** 0 **Matches:** 1(0) **Sequences:** 1(0)
Ammodytin L(2) variant OS=Vipera berus berus OX=31156 PE=2 SV=1
Q6A394_VIPBB **Mass:** 16414 **Score:** 0 **Matches:** 1(0) **Sequences:** 1(0)
Ammodytin L(2) isoform OS=Vipera berus berus OX=31156 PE=2 SV=1

---

8. A0A223PK32_DABRR **Mass:** 57907 **Score:** 0 **Matches:** 1(0) **Sequences:** 1(0)
Atrial natriuretic peptide receptor 3 (Fragment) OS=Daboia russelii OX=8707 PE=2 SV=1
☐ Check to include this hit in error tolerant search or archive report

Query	Observed	Mr(expt)	Mr(calc)	ppm	Miss	Score	Expect	Rank	Unique	Peptide
75	**2217.9647**	**2216.9575**	**2217.0816**	**-56.00**	**1**	**2**	**0.58**	**1**	**U**	**R.GVIMCASSDAIRNIMLAAHR.Q + 2 Oxidation (M)**

---

9. A0A223PK16_DABRR **Score:** 0 **Matches:** 1(0) **Sequences:** 1(0)
C-type lectin domain family 11 member A (Fragment) OS=Daboia russelii OX=8707 PE=2 SV=1
☐ Check to include this hit in error tolerant search or archive report

Query	Observed	Mr(expt)	Mr(calc)	ppm	Miss	Score	Expect	Rank	Unique	Peptide
75	2217.9647	2216.9575	2217.0873	-58.55	1	2	0.62	2	U	-.ILLSTAQGQRGDELSSEEER.K

---

10. VM3VB_MACLB **Score:** 0 **Matches:** 1(0) **Sequences:** 1(0)
Zinc metalloproteinase-disintegrin-like VLAIP-B OS=Macrovipera lebetina OX=8709 PE=1 SV=1
☐ Check to include this hit in error tolerant search or archive report

Query	Observed	Mr(expt)	Mr(calc)	ppm	Miss	Score	Expect	Rank	Unique	Peptide
69	1941.8985	1940.8912	1940.8940	-1.47	1	1	0.73	3	U	R.IDCTCGAKSCIMSGILR.C

Peptide matches not assigned to protein hits: (no details means no match)

	Query	Observed	Mr(expt)	Mr(calc)	ppm	Miss	Score	Expect	Rank	Unique	Peptide
☑	1	*817.1395*	*816.1323*								
☑	2	*837.1000*	*836.0928*								
☑	3	*839.1118*	*838.1046*								
☑	4	*852.3774*	*851.3701*								
☑	5	*855.0882*	*854.0809*								
☑	6	*859.0799*	*858.0727*								
☑	7	*861.0964*	*860.0891*								
☑	8	*868.5738*	*867.5665*								
☑	9	*871.0597*	*870.0524*								
☑	10	*874.0428*	*873.0355*								
☑	11	*877.0680*	*876.0607*								
☑	12	*882.6029*	*881.5957*								
☑	13	*903.4152*	*902.4079*								
☑	14	*905.5111*	*904.5038*								
☑	15	*909.3941*	*908.3868*								
☑	17	*1022.1471*	*1021.1399*								
☑	18	*1044.1224*	*1043.1152*								
☑	19	*1064.0902*	*1063.0829*								
☑	20	*1066.1043*	*1065.0970*								
☑	21	*1082.0794*	*1081.0721*								
☑	22	*1149.5023*	*1148.4950*								
☑	23	*1157.6635*	*1156.6562*								
☑	24	*1175.6978*	*1174.6905*								
☑	25	*1201.6609*	*1200.6536*								
☑	26	*1222.6157*	*1221.6084*								
☑	27	*1249.1322*	*1248.1249*								
☑	28	*1252.6490*	*1251.6417*								
☑	29	*1267.6375*	*1266.6303*								
☑	30	*1271.1084*	*1270.1011*								
☑	31	*1283.6283*	*1282.6210*								
☑	32	*1289.6181*	*1288.6108*								
☑	33	*1293.6475*	*1292.6402*								
☑	34	*1305.5904*	*1304.5831*								
☑	35	*1309.6407*	*1308.6334*								
☑	36	*1320.6258*	*1319.6185*								
☑	37	*1323.6473*	*1322.6400*								
☑	39	*1345.6280*	*1344.6207*								
☑	41	*1361.6097*	*1360.6025*								
☑	42	*1365.6708*	*1364.6635*								
☑	43	*1377.5960*	*1376.5888*								
☑	44	*1393.5844*	*1392.5771*								
☑	45	*1395.7311*	*1394.7238*								
☑	46	*1411.7256*	*1410.7183*								
☑	47	*1436.7603*	*1435.7530*								
☑	48	*1475.7776*	*1474.7703*								
☑	49	*1476.1123*	*1475.1051*								
☑	50	*1557.8703*	*1556.8630*								
☑	51	*1636.7539*	*1635.7466*								
☑	52	*1707.8181*	*1706.8109*								
☑	53	*1718.7880*	*1717.7807*								
☑	55	*1734.7794*	*1733.7721*								
☑	56	*1740.7760*	*1739.7687*								
☑	57	*1744.8013*	*1743.7940*								
☑	59	*1754.7913*	*1753.7840*								
☑	60	*1756.7547*	*1755.7474*								
☑	61	*1770.7537*	*1769.7465*								
☑	62	*1772.7239*	*1771.7167*								
☑	63	*1838.9638*	*1837.9566*								

☑	64	*1851.8058*	*1850.7985*
☑	65	*1862.8756*	*1861.8683*
☑	66	*1876.8772*	*1875.8699*
☑	67	*1921.8557*	*1920.8484*
☑	68	*1924.8861*	*1923.8788*
☑	70	*1948.8578*	*1947.8505*
☑	71	*1967.8661*	*1966.8588*
☑	72	*2005.8049*	*2004.7976*
☑	73	*2118.8044*	*2117.7972*
☑	74	*2170.8299*	*2169.8226*
☑	77	*2255.8811*	*2254.8738*
☑	78	*2265.2040*	*2264.1968*
☑	79	*2277.1368*	*2276.1296*
☑	80	*2566.1425*	*2565.1352*
☑	81	*2618.1737*	*2617.1664*
☑	82	*2625.1754*	*2624.1681*
☑	83	*2636.1875*	*2635.1802*
☑	84	*2663.1955*	*2662.1882*
☑	86	*2695.2126*	*2694.2053*
☑	87	*2708.2152*	*2707.2079*
☑	88	*2720.1459*	*2719.1387*
☑	89	*2724.2164*	*2723.2091*
☑	90	*3347.7264*	*3346.7191*

Search Parameters

```
Type of search         : MS/MS Ion Search
Enzyme                 : Trypsin
Fixed modifications    : Carbamidomethyl (C)
Variable modifications : Oxidation (M)
Mass values            : Monoisotopic
Protein Mass           : Unrestricted
Peptide Mass Tolerance : ± 100 ppm
Fragment Mass Tolerance: ± 0.7 Da
Max Missed Cleavages   : 1
Instrument type        : MALDI-TOF-TOF
Number of queries      : 90
```

Mascot: http://www.matrixscience.com/

Mascot Search Results

```
User          :
Email         :
Search title  : vipera_all_Lift file:\\Proteomics1\d\data\2105\210505_tliepol_1_68252-68266\68252_68266_lpardo\0_G12\1\0_G12.xml
Database      : vipera  (1414 sequences; 347452 residues)
Timestamp     : 5 May 2021 at 10:05:58 GMT
Warning       : A Peptide summary report will usually give a much clearer picture of MS/MS search results.
Top Score     : 389 for PA2A1_MACLB, Acidic phospholipase A2 1 OS=Macrovipera lebetina OX=8709 PE=1 SV=1
```

Mascot Score Histogram

Protein score is -10*Log(P), where P is the probability that the observed match is a random event.
Protein scores greater than 44 are significant (p<0.05).
Protein scores are derived from ions scores as a non-probabilistic basis for ranking protein hits.

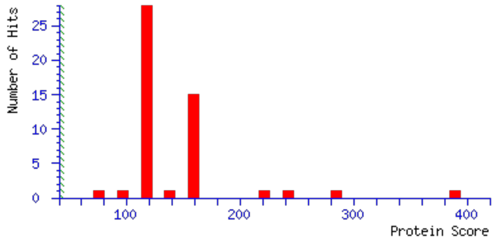

Protein Summary Report

Format As | Protein Summary (deprecated) | Help

Significance threshold p< 0.05 Max. number of hits 50

Overview Table

Click on column header to jump to entry in results list.
Move mouse over any indicator to highlight identical peptides.
Click on an indicator to see details of individual match.
Use check boxes to select sub-set of queries for new search.

Mouse over: -Query- -Accession- -Sequence-

Hit:	1	2	3	4	5	6	7	8	9	10	11	12	13	14	15	16	17	18	19	20	21	22	23	24	25	26	27	28	29	30	31	32	33	34	35	36	37	38	39	40	41	42	43	44	45	46	47	48	49	50
☑ 817.1395 (1+)																																																		
☑ 837.1000 (1+)																																																		
☑ 839.1118 (1+)																																																		
☑ 852.3774 (1+)																																																		
☑ 855.0882 (1+)																																																		
☑ 859.0799 (1+)																																																		
☑ 861.0964 (1+)																																																		
☑ 868.5738 (1+)																																																		
☑ 871.0597 (1+)																																																		
☑ 874.0428 (1+)																																																		
☑ 877.0680 (1+)																																																		
☑ 882.6029 (1+)																																																		
☑ 903.4152 (1+)																																																		
☑ 905.5111 (1+)																																																		
☑ 909.3941 (1+)																																																		
☑ 987.5256 (1+)	●	●	●	●																●																														●
☑ 1022.1471 (1+)																																																		
☑ 1044.1224 (1+)																																																		
☑ 1064.0902 (1+)																																																		
☑ 1066.1043 (1+)																																																		
☑ 1082.0794 (1+)																																																		
☑ 1149.5023 (1+)																																																		
☑ 1157.6635 (1+)																																																		
☑ 1175.6978 (1+)																																																		
☑ 1201.6609 (1+)																																																		
☑ 1222.6157 (1+)																																																		
☑ 1249.1322 (1+)																																																		
☑ 1252.6490 (1+)																																																		
☑ 1267.6375 (1+)																																																		
☑ 1271.1084 (1+)																																																		
☑ 1283.6283 (1+)																																																		
☑ 1289.6181 (1+)																																																		
☑ 1293.6475 (1+)																																																		
☑ 1305.5904 (1+)																																																		
☑ 1309.6407 (1+)																																																		

☑ 1320.6258 (1+)																																																		
☑ 1323.6473 (1+)																																																		
☑ 1339.6437 (1+)	●	●			●	●	●	●	●	●	●	●	●	●	●	●	●	●	●		●																													●
☑ 1345.6280 (1+)																																																		
☑ 1355.6382 (1+)	●	●			●	●	●	●	●	●	●	●	●	●	●	●	●	●	●																															●
☑ 1361.6097 (1+)																																																		
☑ 1365.6708 (1+)																																																		
☑ 1377.5960 (1+)																																																		
☑ 1393.5844 (1+)																																																		
☑ 1395.7311 (1+)																																																		
☑ 1411.7256 (1+)																																																		
☑ 1436.7603 (1+)																																																		
☑ 1475.7776 (1+)																																																		
☑ 1476.1123 (1+)																																																		
☑ 1557.8703 (1+)																																																		
☑ 1636.7539 (1+)																																																		
☑ 1707.8181 (1+)																																																		
☑ 1718.7880 (1+)																																																		
☑ 1732.8009 (1+)	●		●	●	●	●	●	●	●	●	●	●	●	●	●	●	●	●	●		●	●	●	●	●	●	●	●	●	●	●	●	●	●	●	●	●	●	●	●	●	●	●	●	●	●	●	●	●	
☑ 1734.7794 (1+)																																																		
☑ 1740.7760 (1+)																																																		
☑ 1744.8013 (1+)																																																		
☑ 1748.7978 (1+)	●		●	●	●	●	●	●	●	●	●	●	●	●	●	●	●	●	●		●	●	●	●	●	●	●	●	●	●	●	●	●	●	●	●	●	●	●	●	●	●	●	●	●	●	●	●	●	
☑ 1754.7913 (1+)																																																		
☑ 1756.7547 (1+)																																																		
☑ 1770.7537 (1+)																																																		
☑ 1772.7239 (1+)																																																		
☑ 1838.9638 (1+)																																																		
☑ 1851.8058 (1+)																																																		
☑ 1862.8756 (1+)																																																		
☑ 1876.8772 (1+)																																																		
☑ 1921.8557 (1+)																																																		
☑ 1924.8861 (1+)																																																		
☑ 1941.8985 (1+)	●	●																																																
☑ 1948.8578 (1+)																																																		
☑ 1967.8661 (1+)																																																		
☑ 2005.8049 (1+)																																																		
☑ 2118.8044 (1+)																																																		
☑ 2170.8299 (1+)																																																		
☑ 2217.9647 (1+)																																																		
☑ 2234.8584 (1+)	●	●																																																
☑ 2255.8811 (1+)																																																		
☑ 2265.2040 (1+)																																																		
☑ 2277.1368 (1+)																																																		
☑ 2566.1425 (1+)																																																		
☑ 2618.1737 (1+)																																																		
☑ 2625.1754 (1+)																																																		
☑ 2636.1875 (1+)																																																		
☑ 2663.1955 (1+)																																																		
☑ 2682.2062 (1+)	●	●	●	●																●																														
☑ 2695.2126 (1+)																																																		
☑ 2708.2152 (1+)																																																		
☑ 2720.1459 (1+)																																																		
☑ 2724.2164 (1+)																																																		
☑ 3347.7264 (1+)																																																		

Select All Select None Search Selected

Index

	Accession	Mass	Score	Description
1.	PA2A1_MACLB	16337	389	Acidic phospholipase A2 1 OS=Macrovipera lebetina OX=8709 PE=1 SV=1
2.	PA2A2_MACLB	16247	279	Acidic phospholipase A2 2 OS=Macrovipera lebetina OX=8709 PE=1 SV=1
3.	PA2A_DABPA	16277	235	Acidic phospholipase A2 VpaPLA2 OS=Daboia palaestinae OX=1170828 PE=1 SV=1
4.	PA2A_MACLN	16243	225	Acidic phospholipase A2 MVL-PLA2 OS=Macrovipera lebetina transmediterranea OX=384075 PE=1 SV=1
5.	Q6A3G8_VIPAA	15517	169	Ammodytin I1(A) variant OS=Vipera ammodytes ammodytes OX=8705 PE=2 SV=1
6.	Q6A3G4_VIPAM	16280	169	Ammodytin I1(D) isoform OS=Vipera ammodytes montandoni OX=235554 PE=2 SV=1
7.	Q6A3N0_VIPAM	16279	169	Ammodytin I1(A) isoform OS=Vipera ammodytes ruffoi OX=246549 PE=2 SV=1
8.	Q6A3G5_VIPAE	16280	169	Ammodytin I1(D) isoform OS=Vipera ammodytes meridionalis OX=73841 PE=2 SV=1
9.	Q6YC90_VIPAS	16279	169	Ammodytin I1 isoform 2 OS=Vipera aspis OX=8706 GN=AmtI1 PE=3 SV=1
10.	Q6A3K8_VIPAP	16267	169	Ammodytin I1(A') isoform OS=Vipera aspis aspis OX=194601 PE=2 SV=1
11.	Q6A3K4_VIPAP	16252	169	Ammodytin I1(A) variant OS=Vipera aspis aspis OX=194601 PE=2 SV=1
12.	Q6A3K7_VIPAP	16279	169	Ammodytin I1(A) isoform OS=Vipera aspis aspis OX=194601 PE=2 SV=1
13.	PA2A1_VIPAA	16279	169	Acidic phospholipase A2 ammodytin I1 OS=Vipera ammodytes ammodytes OX=8705 PE=2 SV=1
14.	Q7T1D1_VIPBB	16258	168	Ammodytin I1 OS=Vipera berus berus OX=31156 GN=AmtI1 PE=2 SV=1
15.	Q6A3K0_VIPBB	16214	168	Ammodytin I1(B') variant OS=Vipera berus berus OX=31156 PE=2 SV=1
16.	Q1RP80_VIPBN	16258	168	Phospholipase A2 ammodytin I1 OS=Vipera berus nikolskii OX=1808362 GN=I1 PE=2 SV=1
17.	Q6A3M8_VIPAM	16378	168	Ammodytin I1(A) variant OS=Vipera ammodytes ruffoi OX=246549 PE=2 SV=1
18.	Q6A3I8_VIPUR	16290	167	Ammodytin I1(B) isoform OS=Vipera ursinii OX=103942 PE=2 SV=1
19.	Q6A3M9_VIPAM	16286	166	Ammodytin I1(B) isoform OS=Vipera ammodytes ruffoi OX=246549 PE=2 SV=1
20.	PA2A7_DABPA	16190	129	Acidic phospholipase A2 VP7 OS=Daboia palaestinae OX=1170828 PE=3 SV=1

21.	Q6A3H1_VIPAS	16292	127	Ammodytin I1(C) variant OS=Vipera aspis atra OX=246182 PE=2 SV=1
22.	Q6A3J3_VIPBB	16280	123	Ammodytin I1(C) variant OS=Vipera berus berus OX=31156 PE=2 SV=1
23.	Q6A3H6_VIPAS	16258	123	Ammodytin I1(C) isoform OS=Vipera aspis atra OX=246182 PE=2 SV=1
24.	Q6A3J2_VIPBB	16244	123	Ammodytin I1(C) variant OS=Vipera berus berus OX=31156 PE=2 SV=1
25.	Q6A3J0_VIPBB	16280	123	Ammodytin I1(C) variant OS=Vipera berus berus OX=31156 PE=2 SV=1
26.	Q6A3H7_VIPAS	16244	123	Ammodytin I1(F) isoform OS=Vipera aspis atra OX=246182 PE=2 SV=1
27.	Q6A3H4_VIPAS	16244	123	Ammodytin I1(C) isoform OS=Vipera aspis atra OX=246182 PE=2 SV=1
28.	Q7T1D3_VIPAS	16258	123	Ammodytin I1 isoform 1 OS=Vipera aspis OX=8706 GN=AmtI1 PE=3 SV=1
29.	Q6A3I1_VIPAP	16288	123	Ammodytin I1(C) variant OS=Vipera aspis aspis OX=194601 PE=2 SV=1
30.	Q6A3I0_VIPAP	16272	123	Ammodytin I1(C) variant OS=Vipera aspis aspis OX=194601 PE=2 SV=1
31.	Q6A3H8_VIPAP	16330	123	Ammodytin I1(C) variant OS=Vipera aspis aspis OX=194601 PE=2 SV=1
32.	Q6A3M5_VIPAP	16257	123	Ammodytin I1(C) variant OS=Vipera aspis aspis OX=194601 PE=2 SV=1
33.	Q6A3G1_VIPAP	16246	123	Ammodytin I1(C') isoform OS=Vipera aspis aspis OX=194601 PE=2 SV=1
34.	Q7T1D4_VIPAP	16258	123	Ammodytin I1 OS=Vipera aspis aspis OX=194601 GN=AmtI1 PE=2 SV=1
35.	Q7T1D2_VIPAZ	16258	123	Ammodytin I1 OS=Vipera aspis zinnikeri OX=55427 GN=AmtI1 PE=2 SV=1
36.	Q6A3J4_VIPBB	16258	123	Ammodytin I1(C) isoform OS=Vipera berus berus OX=31156 PE=2 SV=1
37.	Q6A3J6_VIPAZ	16330	123	Ammodytin I1(C) isoform OS=Vipera aspis zinnikeri OX=55427 PE=2 SV=1
38.	Q6A3I3_VIPAP	16286	123	Ammodytin I1(C) isoform OS=Vipera aspis aspis OX=194601 PE=2 SV=1
39.	Q6A3F7_VIPAP	16310	122	Ammodytin I1(C) variant OS=Vipera aspis aspis OX=194601 PE=2 SV=1
40.	Q6A3F6_VIPAP	16200	114	Ammodytin I1 (C) variant OS=Vipera aspis aspis OX=194601 PE=2 SV=1
41.	Q6A3I9_VIPBB	16187	114	Ammodytin I1(C) variant OS=Vipera berus berus OX=31156 PE=2 SV=1
42.	Q6A3H5_VIPAS	16245	114	Ammodytin I1(F) variant OS=Vipera aspis atra OX=246182 PE=2 SV=1
43.	Q6A3I6_VIPAP	16257	113	Ammodytin I1(C) variant OS=Vipera aspis aspis OX=194601 PE=2 SV=1
44.	Q6A3I4_VIPAP	16272	112	Ammodytin I1(C) variant OS=Vipera aspis aspis OX=194601 PE=2 SV=1
45.	Q6A3K9_VIPAP	16202	112	Ammodytin I1(E) variant OS=Vipera aspis aspis OX=194601 PE=2 SV=1
46.	Q6A3L1_VIPAP	16272	112	Ammodytin I1(E) isoform OS=Vipera aspis aspis OX=194601 PE=2 SV=1
47.	Q6A3L0_VIPAP	16360	112	Ammodytin I1(E) variant OS=Vipera aspis aspis OX=194601 PE=2 SV=1
48.	Q6A3H2_VIPAS	16198	112	Ammodytin I1(C) variant OS=Vipera aspis atra OX=246182 PE=2 SV=1
49.	Q6A3M4_VIPAP	16217	104	Ammodytin I1(C) variant OS=Vipera aspis aspis OX=194601 PE=2 SV=1
50.	PA2A2_DABRR	16430	75	Acidic phospholipase A2 Drk-a2 OS=Daboia russelii OX=8707 PE=2 SV=1

Results List

1. PA2A1_MACLB **Mass:** 16337 **Score:** 389 **Expect:** 1.8e-036 **Matches:** 10

Acidic phospholipase A2 1 OS=Macrovipera lebetina OX=8709 PE=1 SV=1

Observed	Mr(expt)	Mr(calc)	ppm	Start	End	Miss	Ions	Peptide
909.3941	908.3868	908.3480	42.6	100	106	0	---	R.AVCECDR.V
987.5256	986.5184	986.4781	40.8	50	58	0	3	K.GKPQDATDR.C
1339.6437	1338.6364	1338.5988	28.1	123	132	0	56	K.YMLYSLFDCK.E
1355.6382	1354.6310	1354.5937	27.5	123	132	0	(11)	K.YMLYSLFDCK.E + Oxidation (M)
1732.8009	1731.7936	1731.7596	19.6	107	121	0	87	R.VAAICFGENMNTYDK.K
1748.7978	1747.7906	1747.7545	20.6	107	121	0	(55)	R.VAAICFGENMNTYDK.K + Oxidation (M)
1876.8772	1875.8699	1875.8495	10.9	107	122	1	---	R.VAAICFGENMNTYDKK.Y + Oxidation (M)
1941.8985	1940.8912	1940.8536	19.4	123	137	1	44	K.YMLYSLFDCKEESEK.C
2234.8584	2233.8511	2233.8108	18.0	59	76	0	31	R.CCFVHDCCYGSVNGCDPK.L
2682.2062	2681.1989	2681.1374	22.9	77	99	0	98	K.LSTYSYSFQNGDIVCGDDDPCLR.A

No match to: 817.1395, 837.1000, 839.1118, 852.3774, 855.0882, 859.0799, 861.0964, 868.5738, 871.0597, 874.0428, 877.0680, 882.6029, 903.4152, 905.5111, 1022.1471, 1044.1224, 1064.0902, 1066.1043, 1082.0794, 1149.5023, 1157.6635, 1175.6978, 1201.6609, 1222.6157, 1249.1322, 1252.6490, 1267.6375, 1271.1084, 1283.6283, 1289.6181, 1293.6475, 1305.5904, 1309.6407, 1320.6258, 1323.6473, 1345.6280, 1361.6097, 1365.6708, 1377.5960, 1393.5844, 1395.7311, 1411.7256, 1436.7603, 1475.7776, 1476.1123, 1557.8703, 1636.7539, 1707.8181, 1718.7880, 1734.7794, 1740.7760, 1744.8013, 1754.7913, 1756.7547, 1770.7537, 1772.7239, 1838.9638, 1851.8058, 1862.8756, 1921.8557, 1924.8861, 1948.8578, 1967.8661, 2005.8049, 2118.8044, 2170.8299, 2217.9647, 2255.8811, 2265.2040, 2277.1368, 2566.1425, 2618.1737, 2625.1754, 2636.1875, 2663.1955, 2695.2126, 2708.2152, 2720.1459, 2724.2164, 3347.7264

2. PA2A2_MACLB **Mass:** 16247 **Score:** 279 **Expect:** 1.8e-025 **Matches:** 7

Acidic phospholipase A2 2 OS=Macrovipera lebetina OX=8709 PE=1 SV=1

Observed	Mr(expt)	Mr(calc)	ppm	Start	End	Miss	Ions	Peptide
909.3941	908.3868	908.3480	42.6	100	106	0	---	R.AVCECDR.V
987.5256	986.5184	986.4781	40.8	50	58	0	3	K.GKPQDATDR.C
1339.6437	1338.6364	1338.5988	28.1	123	132	0	56	K.YMLYSLFDCK.E
1355.6382	1354.6310	1354.5937	27.5	123	132	0	(11)	K.YMLYSLFDCK.E + Oxidation (M)
1941.8985	1940.8912	1940.8536	19.4	123	137	1	44	K.YMLYSLFDCKEESEK.C
2234.8584	2233.8511	2233.8108	18.0	59	76	0	31	R.CCFVHDCCYGSVNGCDPK.L
2682.2062	2681.1989	2681.1374	22.9	77	99	0	98	K.LSTYSYSFQNGDIVCGDDDPCLR.A

No match to: 817.1395, 837.1000, 839.1118, 852.3774, 855.0882, 859.0799, 861.0964, 868.5738, 871.0597, 874.0428, 877.0680, 882.6029, 903.4152, 905.5111, 1022.1471, 1044.1224, 1064.0902, 1066.1043, 1082.0794, 1149.5023, 1157.6635, 1175.6978, 1201.6609, 1222.6157, 1249.1322, 1252.6490, 1267.6375, 1271.1084, 1283.6283, 1289.6181, 1293.6475, 1305.5904, 1309.6407, 1320.6258, 1323.6473, 1345.6280, 1361.6097, 1365.6708, 1377.5960, 1393.5844, 1395.7311, 1411.7256, 1436.7603, 1475.7776, 1476.1123, 1557.8703, 1636.7539, 1707.8181, 1718.7880, 1732.8009, 1734.7794, 1740.7760, 1744.8013, 1748.7978, 1754.7913, 1756.7547, 1770.7537, 1772.7239, 1838.9638, 1851.8058, 1862.8756, 1876.8772, 1921.8557, 1924.8861, 1948.8578, 1967.8661, 2005.8049, 2118.8044, 2170.8299, 2217.9647, 2255.8811, 2265.2040, 2277.1368, 2566.1425, 2618.1737, 2625.1754, 2636.1875, 2663.1955, 2695.2126, 2708.2152, 2720.1459, 2724.2164, 3347.7264

3. PA2A_DABPA **Mass:** 16277 **Score:** 235 **Expect:** 4.5e-021 **Matches:** 7

Acidic phospholipase A2 VpaPLA2 OS=Daboia palaestinae OX=1170828 PE=1 SV=1

Observed	Mr(expt)	Mr(calc)	ppm	Start	End	Miss	Ions	Peptide
909.3941	908.3868	908.3480	42.6	100	106	0	---	R.AVCECDR.V
987.5256	986.5184	986.4781	40.8	50	58	0	3	K.GKPQDATDR.C
1732.8009	1731.7936	1731.7596	19.6	107	121	0	87	R.VAAICFGENMNTYDK.K
1748.7978	1747.7906	1747.7545	20.6	107	121	0	(55)	R.VAAICFGENMNTYDK.K + Oxidation (M)
1876.8772	1875.8699	1875.8495	10.9	107	122	1	---	R.VAAICFGENMNTYDKK.Y + Oxidation (M)
2170.8299	2169.8226	2169.8039	8.59	123	138	1	---	K.YMLYSFFDCMEESEKC.- + 2 Oxidation (M)
2682.2062	2681.1989	2681.1374	22.9	77	99	0	98	K.LSTYSYSFQNGDIVCGDDDPCLR.A

No match to: 817.1395, 837.1000, 839.1118, 852.3774, 855.0882, 859.0799, 861.0964, 868.5738, 871.0597, 874.0428, 877.0680, 882.6029, 903.4152, 905.5111, 1022.1471, 1044.1224, 1064.0902, 1066.1043, 1082.0794, 1149.5023, 1157.6635, 1175.6978, 1201.6609, 1222.6157, 1249.1322, 1252.6490, 1267.6375, 1271.1084, 1283.6283, 1289.6181, 1293.6475, 1305.5904, 1309.6407, 1320.6258, 1323.6473, 1339.6437, 1345.6280, 1355.6382, 1361.6097, 1365.6708, 1377.5960, 1393.5844, 1395.7311, 1411.7256, 1436.7603, 1475.7776, 1476.1123, 1557.8703, 1636.7539, 1707.8181, 1718.7880, 1734.7794, 1740.7760, 1744.8013, 1754.7913, 1756.7547, 1770.7537, 1772.7239, 1838.9638, 1851.8058, 1862.8756, 1921.8557, 1924.8861, 1941.8985, 1948.8578, 1967.8661, 2005.8049, 2118.8044, 2217.9647, 2234.8584, 2255.8811, 2265.2040, 2277.1368, 2566.1425, 2618.1737, 2625.1754, 2636.1875, 2663.1955, 2695.2126, 2708.2152, 2720.1459, 2724.2164, 3347.7264

4. PA2A_MACLN **Mass:** 16243 **Score:** 225 **Expect:** 4.5e-020 **Matches:** 6

Acidic phospholipase A2 MVL-PLA2 OS=Macrovipera lebetina transmediterranea OX=384075 PE=1 SV=1

Observed	Mr(expt)	Mr(calc)	ppm	Start	End	Miss	Ions	Peptide
909.3941	908.3868	908.3480	42.6	100	106	0	---	R.AVCECDR.V
987.5256	986.5184	986.4781	40.8	50	58	0	3	K.GKPQDATDR.C
1732.8009	1731.7936	1731.7596	19.6	107	121	0	87	R.VAAICFGENMNTYDK.K
1748.7978	1747.7906	1747.7545	20.6	107	121	0	(55)	R.VAAICFGENMNTYDK.K + Oxidation (M)
1876.8772	1875.8699	1875.8495	10.9	107	122	1	---	R.VAAICFGENMNTYDKK.Y + Oxidation (M)

2682.2062	2681.1989	2681.1374	22.9	77 -	99	0	98	K.LSTYSYSFQNGDIVCGDDDPCLR.A

No match to: 817.1395, 837.1000, 839.1118, 852.3774, 855.0882, 859.0799, 861.0964, 868.5738, 871.0597, 874.0428, 877.0680, 882.6029, 903.4152, 905.5111, 1022.1471, 1044.1224, 1064.0902, 1066.1043, 1082.0794, 1149.5023, 1157.6635, 1175.6978, 1201.6609, 1222.6157, 1249.1322, 1252.6490, 1267.6375, 1271.1084, 1283.6283, 1289.6181, 1293.6475, 1305.5904, 1309.6407, 1320.6258, 1323.6473, 1339.6437, 1345.6280, 1355.6382, 1361.6097, 1365.6708, 1377.5960, 1393.5844, 1395.7311, 1411.7256, 1436.7603, 1475.7776, 1476.1123, 1557.8703, 1636.7539, 1707.8181, 1718.7880, 1734.7794, 1740.7760, 1744.8013, 1754.7913, 1756.7547, 1770.7537, 1772.7239, 1838.9638, 1851.8058, 1862.8756, 1921.8557, 1924.8861, 1941.8985, 1948.8578, 1967.8661, 2005.8049, 2118.8044, 2170.8299, 2217.9647, 2234.8584, 2255.8811, 2265.2040, 2277.1368, 2566.1425, 2618.1737, 2625.1754, 2636.1875, 2663.1955, 2695.2126, 2708.2152, 2720.1459, 2724.2164, 3347.7264

5. Q6A3G8_VIPAA **Mass:** 15517 **Score:** 169 **Expect:** 1.8e-014 **Matches:** 6

Ammodytin I1(A) variant OS=Vipera ammodytes ammodytes OX=8705 PE=2 SV=1

Observed	Mr(expt)	Mr(calc)	ppm	Start	End	Miss	Ions	Peptide
909.3941	908.3868	908.3480	42.6	100 -	106	0	---	R.AVCECDR.V
1339.6437	1338.6364	1338.5988	28.1	123 -	132	0	56	K.YMLYSLFDCK.-
1355.6382	1354.6310	1354.5937	27.5	123 -	132	0	(11)	K.YMLYSLFDCK.- + Oxidation (M)
1732.8009	1731.7936	1731.7596	19.6	107 -	121	0	87	R.VAAICFGENMNTYDK.K
1748.7978	1747.7906	1747.7545	20.6	107 -	121	0	(55)	R.VAAICFGENMNTYDK.K + Oxidation (M)
1876.8772	1875.8699	1875.8495	10.9	107 -	122	1	---	R.VAAICFGENMNTYDKK.Y + Oxidation (M)

No match to: 817.1395, 837.1000, 839.1118, 852.3774, 855.0882, 859.0799, 861.0964, 868.5738, 871.0597, 874.0428, 877.0680, 882.6029, 903.4152, 905.5111, 987.5256, 1022.1471, 1044.1224, 1064.0902, 1066.1043, 1082.0794, 1149.5023, 1157.6635, 1175.6978, 1201.6609, 1222.6157, 1249.1322, 1252.6490, 1267.6375, 1271.1084, 1283.6283, 1289.6181, 1293.6475, 1305.5904, 1309.6407, 1320.6258, 1323.6473, 1345.6280, 1361.6097, 1365.6708, 1377.5960, 1393.5844, 1395.7311, 1411.7256, 1436.7603, 1475.7776, 1476.1123, 1557.8703, 1636.7539, 1707.8181, 1718.7880, 1734.7794, 1740.7760, 1744.8013, 1754.7913, 1756.7547, 1770.7537, 1772.7239, 1838.9638, 1851.8058, 1862.8756, 1921.8557, 1924.8861, 1941.8985, 1948.8578, 1967.8661, 2005.8049, 2118.8044, 2170.8299, 2217.9647, 2234.8584, 2255.8811, 2265.2040, 2277.1368, 2566.1425, 2618.1737, 2625.1754, 2636.1875, 2663.1955, 2682.2062, 2695.2126, 2708.2152, 2720.1459, 2724.2164, 3347.7264

6. Q6A3G4_VIPAM **Mass:** 16280 **Score:** 169 **Expect:** 1.8e-014 **Matches:** 6

Ammodytin I1(D) isoform OS=Vipera ammodytes montandoni OX=235554 PE=2 SV=1

Observed	Mr(expt)	Mr(calc)	ppm	Start	End	Miss	Ions	Peptide
909.3941	908.3868	908.3480	42.6	100 -	106	0	---	R.AVCECDR.V
1339.6437	1338.6364	1338.5988	28.1	123 -	132	0	56	K.YMLYSLFDCK.E
1355.6382	1354.6310	1354.5937	27.5	123 -	132	0	(11)	K.YMLYSLFDCK.E + Oxidation (M)
1732.8009	1731.7936	1731.7596	19.6	107 -	121	0	87	R.VAAICFGENMNTYDK.K
1748.7978	1747.7906	1747.7545	20.6	107 -	121	0	(55)	R.VAAICFGENMNTYDK.K + Oxidation (M)
1876.8772	1875.8699	1875.8495	10.9	107 -	122	1	---	R.VAAICFGENMNTYDKK.Y + Oxidation (M)

No match to: 817.1395, 837.1000, 839.1118, 852.3774, 855.0882, 859.0799, 861.0964, 868.5738, 871.0597, 874.0428, 877.0680, 882.6029, 903.4152, 905.5111, 987.5256, 1022.1471, 1044.1224, 1064.0902, 1066.1043, 1082.0794, 1149.5023, 1157.6635, 1175.6978, 1201.6609, 1222.6157, 1249.1322, 1252.6490, 1267.6375, 1271.1084, 1283.6283, 1289.6181, 1293.6475, 1305.5904, 1309.6407, 1320.6258, 1323.6473, 1345.6280, 1361.6097, 1365.6708, 1377.5960, 1393.5844, 1395.7311, 1411.7256, 1436.7603, 1475.7776, 1476.1123, 1557.8703, 1636.7539, 1707.8181, 1718.7880, 1734.7794, 1740.7760, 1744.8013, 1754.7913, 1756.7547, 1770.7537, 1772.7239, 1838.9638, 1851.8058, 1862.8756, 1921.8557, 1924.8861, 1941.8985, 1948.8578, 1967.8661, 2005.8049, 2118.8044, 2170.8299, 2217.9647, 2234.8584, 2255.8811, 2265.2040, 2277.1368, 2566.1425, 2618.1737, 2625.1754, 2636.1875, 2663.1955, 2682.2062, 2695.2126, 2708.2152, 2720.1459, 2724.2164, 3347.7264

7. Q6A3N0_VIPAM **Mass:** 16279 **Score:** 169 **Expect:** 1.8e-014 **Matches:** 6

Ammodytin I1(A) isoform OS=Vipera ammodytes ruffoi OX=246549 PE=2 SV=1

Observed	Mr(expt)	Mr(calc)	ppm	Start	End	Miss	Ions	Peptide
909.3941	908.3868	908.3480	42.6	100 -	106	0	---	R.AVCECDR.V
1339.6437	1338.6364	1338.5988	28.1	123 -	132	0	56	K.YMLYSLFDCK.E
1355.6382	1354.6310	1354.5937	27.5	123 -	132	0	(11)	K.YMLYSLFDCK.E + Oxidation (M)
1732.8009	1731.7936	1731.7596	19.6	107 -	121	0	87	R.VAAICFGENMNTYDK.K
1748.7978	1747.7906	1747.7545	20.6	107 -	121	0	(55)	R.VAAICFGENMNTYDK.K + Oxidation (M)
1876.8772	1875.8699	1875.8495	10.9	107 -	122	1	---	R.VAAICFGENMNTYDKK.Y + Oxidation (M)

No match to: 817.1395, 837.1000, 839.1118, 852.3774, 855.0882, 859.0799, 861.0964, 868.5738, 871.0597, 874.0428, 877.0680, 882.6029, 903.4152, 905.5111, 987.5256, 1022.1471, 1044.1224, 1064.0902, 1066.1043, 1082.0794, 1149.5023, 1157.6635, 1175.6978, 1201.6609, 1222.6157, 1249.1322, 1252.6490, 1267.6375, 1271.1084, 1283.6283, 1289.6181, 1293.6475, 1305.5904, 1309.6407, 1320.6258, 1323.6473, 1345.6280, 1361.6097, 1365.6708, 1377.5960, 1393.5844, 1395.7311, 1411.7256, 1436.7603, 1475.7776, 1476.1123, 1557.8703, 1636.7539, 1707.8181, 1718.7880, 1734.7794, 1740.7760, 1744.8013, 1754.7913, 1756.7547, 1770.7537, 1772.7239, 1838.9638, 1851.8058, 1862.8756, 1921.8557, 1924.8861, 1941.8985, 1948.8578, 1967.8661, 2005.8049, 2118.8044, 2170.8299, 2217.9647, 2234.8584, 2255.8811, 2265.2040, 2277.1368, 2566.1425, 2618.1737, 2625.1754, 2636.1875, 2663.1955, 2682.2062, 2695.2126, 2708.2152, 2720.1459, 2724.2164, 3347.7264

8. Q6A3G5_VIPAE **Mass:** 16280 **Score:** 169 **Expect:** 1.8e-014 **Matches:** 6

Ammodytin I1(D) isoform OS=Vipera ammodytes meridionalis OX=73841 PE=2 SV=1

Observed	Mr(expt)	Mr(calc)	ppm	Start	End	Miss	Ions	Peptide
909.3941	908.3868	908.3480	42.6	100 -	106	0	---	R.AVCECDR.V
1339.6437	1338.6364	1338.5988	28.1	123 -	132	0	56	K.YMLYSLFDCK.E
1355.6382	1354.6310	1354.5937	27.5	123 -	132	0	(11)	K.YMLYSLFDCK.E + Oxidation (M)
1732.8009	1731.7936	1731.7596	19.6	107 -	121	0	87	R.VAAICFGENMNTYDK.K
1748.7978	1747.7906	1747.7545	20.6	107 -	121	0	(55)	R.VAAICFGENMNTYDK.K + Oxidation (M)
1876.8772	1875.8699	1875.8495	10.9	107 -	122	1	---	R.VAAICFGENMNTYDKK.Y + Oxidation (M)

No match to: 817.1395, 837.1000, 839.1118, 852.3774, 855.0882, 859.0799, 861.0964, 868.5738, 871.0597, 874.0428, 877.0680, 882.6029, 903.4152, 905.5111, 987.5256, 1022.1471, 1044.1224, 1064.0902, 1066.1043, 1082.0794, 1149.5023, 1157.6635, 1175.6978, 1201.6609, 1222.6157, 1249.1322, 1252.6490, 1267.6375, 1271.1084, 1283.6283, 1289.6181, 1293.6475, 1305.5904, 1309.6407, 1320.6258, 1323.6473, 1345.6280, 1361.6097, 1365.6708, 1377.5960, 1393.5844, 1395.7311, 1411.7256, 1436.7603, 1475.7776, 1476.1123, 1557.8703, 1636.7539, 1707.8181, 1718.7880, 1734.7794, 1740.7760, 1744.8013, 1754.7913, 1756.7547, 1770.7537, 1772.7239, 1838.9638, 1851.8058, 1862.8756, 1921.8557, 1924.8861, 1941.8985, 1948.8578, 1967.8661, 2005.8049, 2118.8044, 2170.8299, 2217.9647, 2234.8584, 2255.8811, 2265.2040, 2277.1368, 2566.1425, 2618.1737, 2625.1754, 2636.1875, 2663.1955, 2682.2062, 2695.2126, 2708.2152, 2720.1459, 2724.2164, 3347.7264

9. Q6YC90_VIPAS **Mass:** 16279 **Score:** 169 **Expect:** 1.8e-014 **Matches:** 6

Ammodytin I1 isoform 2 OS=Vipera aspis OX=8706 GN=AmtI1 PE=3 SV=1

Observed	Mr(expt)	Mr(calc)	ppm	Start	End	Miss	Ions	Peptide
909.3941	908.3868	908.3480	42.6	100 -	106	0	---	R.AVCECDR.V
1339.6437	1338.6364	1338.5988	28.1	123 -	132	0	56	K.YMLYSLFDCK.E
1355.6382	1354.6310	1354.5937	27.5	123 -	132	0	(11)	K.YMLYSLFDCK.E + Oxidation (M)
1732.8009	1731.7936	1731.7596	19.6	107 -	121	0	87	R.VAAICFGENMNTYDK.K
1748.7978	1747.7906	1747.7545	20.6	107 -	121	0	(55)	R.VAAICFGENMNTYDK.K + Oxidation (M)
1876.8772	1875.8699	1875.8495	10.9	107 -	122	1	---	R.VAAICFGENMNTYDKK.Y + Oxidation (M)

No match to: 817.1395, 837.1000, 839.1118, 852.3774, 855.0882, 859.0799, 861.0964, 868.5738, 871.0597, 874.0428, 877.0680, 882.6029, 903.4152, 905.5111, 987.5256, 1022.1471, 1044.1224, 1064.0902, 1066.1043, 1082.0794, 1149.5023, 1157.6635, 1175.6978, 1201.6609, 1222.6157, 1249.1322, 1252.6490, 1267.6375, 1271.1084, 1283.6283, 1289.6181, 1293.6475, 1305.5904, 1309.6407, 1320.6258, 1323.6473, 1345.6280, 1361.6097, 1365.6708, 1377.5960, 1393.5844, 1395.7311, 1411.7256, 1436.7603, 1475.7776, 1476.1123, 1557.8703, 1636.7539, 1707.8181, 1718.7880, 1734.7794, 1740.7760, 1744.8013, 1754.7913, 1756.7547, 1770.7537, 1772.7239, 1838.9638, 1851.8058, 1862.8756, 1921.8557, 1924.8861, 1941.8985, 1948.8578, 1967.8661, 2005.8049, 2118.8044, 2170.8299, 2217.9647, 2234.8584, 2255.8811, 2265.2040, 2277.1368, 2566.1425, 2618.1737, 2625.1754, 2636.1875, 2663.1955, 2682.2062, 2695.2126, 2708.2152, 2720.1459, 2724.2164, 3347.7264

10. Q6A3K8_VIPAP **Mass:** 16267 **Score:** 169 **Expect:** 1.8e-014 **Matches:** 6

Ammodytin I1(A') isoform OS=Vipera aspis aspis OX=194601 PE=2 SV=1

Observed	Mr(expt)	Mr(calc)	ppm	Start	End	Miss	Ions	Peptide
909.3941	908.3868	908.3480	42.6	100	106	0	---	R.AVCECDR.V
1339.6437	1338.6364	1338.5988	28.1	123	132	0	56	K.YMLYSLFDCK.E
1355.6382	1354.6310	1354.5937	27.5	123	132	0	(11)	K.YMLYSLFDCK.E + Oxidation (M)
1732.8009	1731.7936	1731.7596	19.6	107	121	0	87	R.VAAICFGENMNTYDK.K
1748.7978	1747.7906	1747.7545	20.6	107	121	0	(55)	R.VAAICFGENMNTYDK.K + Oxidation (M)
1876.8772	1875.8699	1875.8495	10.9	107	122	1	---	R.VAAICFGENMNTYDKK.Y + Oxidation (M)

No match to: 817.1395, 837.1000, 839.1118, 852.3774, 855.0882, 859.0799, 861.0964, 868.5738, 871.0597, 874.0428, 877.0680, 882.6029, 903.4152, 905.5111, 987.5256, 1022.1471, 1044.1224, 1064.0902, 1066.1043, 1082.0794, 1149.5023, 1157.6635, 1175.6978, 1201.6609, 1222.6157, 1249.1322, 1252.6490, 1267.6375, 1271.1084, 1283.6283, 1289.6181, 1293.6475, 1305.5904, 1309.6407, 1320.6258, 1323.6473, 1345.6280, 1361.6097, 1365.6708, 1377.5960, 1393.5844, 1395.7311, 1411.7256, 1436.7603, 1475.7776, 1476.1123, 1557.8703, 1636.7539, 1707.8181, 1718.7880, 1734.7794, 1740.7760, 1744.8013, 1754.7913, 1756.7547, 1770.7537, 1772.7239, 1838.9638, 1851.8058, 1862.8756, 1921.8557, 1924.8861, 1941.8985, 1948.8578, 1967.8661, 2005.8049, 2118.8044, 2170.8299, 2217.9647, 2234.8584, 2255.8811, 2265.2040, 2277.1368, 2566.1425, 2618.1737, 2625.1754, 2636.1875, 2663.1955, 2682.2062, 2695.2126, 2708.2152, 2720.1459, 2724.2164, 3347.7264

11. Q6A3K4_VIPAP **Mass:** 16252 **Score:** 169 **Expect:** 1.8e-014 **Matches:** 6
Ammodytin I1(A) variant OS=Vipera aspis aspis OX=194601 PE=2 SV=1

Observed	Mr(expt)	Mr(calc)	ppm	Start	End	Miss	Ions	Peptide
909.3941	908.3868	908.3480	42.6	100	106	0	---	R.AVCECDR.V
1339.6437	1338.6364	1338.5988	28.1	123	132	0	56	K.YMLYSLFDCK.E
1355.6382	1354.6310	1354.5937	27.5	123	132	0	(11)	K.YMLYSLFDCK.E + Oxidation (M)
1732.8009	1731.7936	1731.7596	19.6	107	121	0	87	R.VAAICFGENMNTYDK.K
1748.7978	1747.7906	1747.7545	20.6	107	121	0	(55)	R.VAAICFGENMNTYDK.K + Oxidation (M)
1876.8772	1875.8699	1875.8495	10.9	107	122	1	---	R.VAAICFGENMNTYDKK.Y + Oxidation (M)

No match to: 817.1395, 837.1000, 839.1118, 852.3774, 855.0882, 859.0799, 861.0964, 868.5738, 871.0597, 874.0428, 877.0680, 882.6029, 903.4152, 905.5111, 987.5256, 1022.1471, 1044.1224, 1064.0902, 1066.1043, 1082.0794, 1149.5023, 1157.6635, 1175.6978, 1201.6609, 1222.6157, 1249.1322, 1252.6490, 1267.6375, 1271.1084, 1283.6283, 1289.6181, 1293.6475, 1305.5904, 1309.6407, 1320.6258, 1323.6473, 1345.6280, 1361.6097, 1365.6708, 1377.5960, 1393.5844, 1395.7311, 1411.7256, 1436.7603, 1475.7776, 1476.1123, 1557.8703, 1636.7539, 1707.8181, 1718.7880, 1734.7794, 1740.7760, 1744.8013, 1754.7913, 1756.7547, 1770.7537, 1772.7239, 1838.9638, 1851.8058, 1862.8756, 1921.8557, 1924.8861, 1941.8985, 1948.8578, 1967.8661, 2005.8049, 2118.8044, 2170.8299, 2217.9647, 2234.8584, 2255.8811, 2265.2040, 2277.1368, 2566.1425, 2618.1737, 2625.1754, 2636.1875, 2663.1955, 2682.2062, 2695.2126, 2708.2152, 2720.1459, 2724.2164, 3347.7264

12. Q6A3K7_VIPAP **Mass:** 16279 **Score:** 169 **Expect:** 1.8e-014 **Matches:** 6
Ammodytin I1(A) isoform OS=Vipera aspis aspis OX=194601 PE=2 SV=1

Observed	Mr(expt)	Mr(calc)	ppm	Start	End	Miss	Ions	Peptide
909.3941	908.3868	908.3480	42.6	100	106	0	---	R.AVCECDR.V
1339.6437	1338.6364	1338.5988	28.1	123	132	0	56	K.YMLYSLFDCK.E
1355.6382	1354.6310	1354.5937	27.5	123	132	0	(11)	K.YMLYSLFDCK.E + Oxidation (M)
1732.8009	1731.7936	1731.7596	19.6	107	121	0	87	R.VAAICFGENMNTYDK.K
1748.7978	1747.7906	1747.7545	20.6	107	121	0	(55)	R.VAAICFGENMNTYDK.K + Oxidation (M)
1876.8772	1875.8699	1875.8495	10.9	107	122	1	---	R.VAAICFGENMNTYDKK.Y + Oxidation (M)

No match to: 817.1395, 837.1000, 839.1118, 852.3774, 855.0882, 859.0799, 861.0964, 868.5738, 871.0597, 874.0428, 877.0680, 882.6029, 903.4152, 905.5111, 987.5256, 1022.1471, 1044.1224, 1064.0902, 1066.1043, 1082.0794, 1149.5023, 1157.6635, 1175.6978, 1201.6609, 1222.6157, 1249.1322, 1252.6490, 1267.6375, 1271.1084, 1283.6283, 1289.6181, 1293.6475, 1305.5904, 1309.6407, 1320.6258, 1323.6473, 1345.6280, 1361.6097, 1365.6708, 1377.5960, 1393.5844, 1395.7311, 1411.7256, 1436.7603, 1475.7776, 1476.1123, 1557.8703, 1636.7539, 1707.8181, 1718.7880, 1734.7794, 1740.7760, 1744.8013, 1754.7913, 1756.7547, 1770.7537, 1772.7239, 1838.9638, 1851.8058, 1862.8756, 1921.8557, 1924.8861, 1941.8985, 1948.8578, 1967.8661, 2005.8049, 2118.8044, 2170.8299, 2217.9647, 2234.8584, 2255.8811, 2265.2040, 2277.1368, 2566.1425, 2618.1737, 2625.1754, 2636.1875, 2663.1955, 2682.2062, 2695.2126, 2708.2152, 2720.1459, 2724.2164, 3347.7264

13. PA2A1_VIPAA **Mass:** 16279 **Score:** 169 **Expect:** 1.8e-014 **Matches:** 6
Acidic phospholipase A2 ammodytin I1 OS=Vipera ammodytes ammodytes OX=8705 PE=2 SV=1

Observed	Mr(expt)	Mr(calc)	ppm	Start	End	Miss	Ions	Peptide
909.3941	908.3868	908.3480	42.6	100	106	0	---	R.AVCECDR.V
1339.6437	1338.6364	1338.5988	28.1	123	132	0	56	K.YMLYSLFDCK.E
1355.6382	1354.6310	1354.5937	27.5	123	132	0	(11)	K.YMLYSLFDCK.E + Oxidation (M)
1732.8009	1731.7936	1731.7596	19.6	107	121	0	87	R.VAAICFGENMNTYDK.K
1748.7978	1747.7906	1747.7545	20.6	107	121	0	(55)	R.VAAICFGENMNTYDK.K + Oxidation (M)
1876.8772	1875.8699	1875.8495	10.9	107	122	1	---	R.VAAICFGENMNTYDKK.Y + Oxidation (M)

No match to: 817.1395, 837.1000, 839.1118, 852.3774, 855.0882, 859.0799, 861.0964, 868.5738, 871.0597, 874.0428, 877.0680, 882.6029, 903.4152, 905.5111, 987.5256, 1022.1471, 1044.1224, 1064.0902, 1066.1043, 1082.0794, 1149.5023, 1157.6635, 1175.6978, 1201.6609, 1222.6157, 1249.1322, 1252.6490, 1267.6375, 1271.1084, 1283.6283, 1289.6181, 1293.6475, 1305.5904, 1309.6407, 1320.6258, 1323.6473, 1345.6280, 1361.6097, 1365.6708, 1377.5960, 1393.5844, 1395.7311, 1411.7256, 1436.7603, 1475.7776, 1476.1123, 1557.8703, 1636.7539, 1707.8181, 1718.7880, 1734.7794, 1740.7760, 1744.8013, 1754.7913, 1756.7547, 1770.7537, 1772.7239, 1838.9638, 1851.8058, 1862.8756, 1921.8557, 1924.8861, 1941.8985, 1948.8578, 1967.8661, 2005.8049, 2118.8044, 2170.8299, 2217.9647, 2234.8584, 2255.8811, 2265.2040, 2277.1368, 2566.1425, 2618.1737, 2625.1754, 2636.1875, 2663.1955, 2682.2062, 2695.2126, 2708.2152, 2720.1459, 2724.2164, 3347.7264

14. Q7T1D1_VIPBB **Mass:** 16258 **Score:** 168 **Expect:** 2.2e-014 **Matches:** 6
Ammodytin I1 OS=Vipera berus berus OX=31156 GN=AmtI1 PE=2 SV=1

Observed	Mr(expt)	Mr(calc)	ppm	Start	End	Miss	Ions	Peptide
909.3941	908.3868	908.3480	42.6	100	106	0	---	R.AVCECDR.V
1339.6437	1338.6364	1338.5988	28.1	123	132	0	56	K.YMLYSLFDCK.E
1355.6382	1354.6310	1354.5937	27.5	123	132	0	(11)	K.YMLYSLFDCK.E + Oxidation (M)
1732.8009	1731.7936	1731.7596	19.6	107	121	0	87	R.VAAICFGENMNTYDK.K
1748.7978	1747.7906	1747.7545	20.6	107	121	0	(55)	R.VAAICFGENMNTYDK.K + Oxidation (M)
1876.8772	1875.8699	1875.8495	10.9	107	122	1	---	R.VAAICFGENMNTYDKK.Y + Oxidation (M)

No match to: 817.1395, 837.1000, 839.1118, 852.3774, 855.0882, 859.0799, 861.0964, 868.5738, 871.0597, 874.0428, 877.0680, 882.6029, 903.4152, 905.5111, 987.5256, 1022.1471, 1044.1224, 1064.0902, 1066.1043, 1082.0794, 1149.5023, 1157.6635, 1175.6978, 1201.6609, 1222.6157, 1249.1322, 1252.6490, 1267.6375, 1271.1084, 1283.6283, 1289.6181, 1293.6475, 1305.5904, 1309.6407, 1320.6258, 1323.6473, 1345.6280, 1361.6097, 1365.6708, 1377.5960, 1393.5844, 1395.7311, 1411.7256, 1436.7603, 1475.7776, 1476.1123, 1557.8703, 1636.7539, 1707.8181, 1718.7880, 1734.7794, 1740.7760, 1744.8013, 1754.7913, 1756.7547, 1770.7537, 1772.7239, 1838.9638, 1851.8058, 1862.8756, 1921.8557, 1924.8861, 1941.8985, 1948.8578, 1967.8661, 2005.8049, 2118.8044, 2170.8299, 2217.9647, 2234.8584, 2255.8811, 2265.2040, 2277.1368, 2566.1425, 2618.1737, 2625.1754, 2636.1875, 2663.1955, 2682.2062, 2695.2126, 2708.2152, 2720.1459, 2724.2164, 3347.7264

15. Q6A3K0_VIPBB **Mass:** 16214 **Score:** 168 **Expect:** 2.2e-014 **Matches:** 6
Ammodytin I1(B') variant OS=Vipera berus berus OX=31156 PE=2 SV=1

Observed	Mr(expt)	Mr(calc)	ppm	Start	End	Miss	Ions	Peptide
909.3941	908.3868	908.3480	42.6	100	106	0	---	R.AVCECDR.V
1339.6437	1338.6364	1338.5988	28.1	123	132	0	56	K.YMLYSLFDCK.E
1355.6382	1354.6310	1354.5937	27.5	123	132	0	(11)	K.YMLYSLFDCK.E + Oxidation (M)
1732.8009	1731.7936	1731.7596	19.6	107	121	0	87	R.VAAICFGENMNTYDK.K
1748.7978	1747.7906	1747.7545	20.6	107	121	0	(55)	R.VAAICFGENMNTYDK.K + Oxidation (M)
1876.8772	1875.8699	1875.8495	10.9	107	122	1	---	R.VAAICFGENMNTYDKK.Y + Oxidation (M)

No match to: 817.1395, 837.1000, 839.1118, 852.3774, 855.0882, 859.0799, 861.0964, 868.5738, 871.0597, 874.0428, 877.0680, 882.6029, 903.4152, 905.5111, 987.5256, 1022.1471, 1044.1224, 1064.0902, 1066.1043, 1082.0794, 1149.5023, 1157.6635, 1175.6978, 1201.6609, 1222.6157, 1249.1322, 1252.6490, 1267.6375, 1271.1084, 1283.6283, 1289.6181, 1293.6475, 1305.5904, 1309.6407, 1320.6258, 1323.6473, 1345.6280, 1361.6097, 1365.6708, 1377.5960, 1393.5844, 1395.7311, 1411.7256, 1436.7603, 1475.7776, 1476.1123, 1557.8703, 1636.7539,

1707.8181, 1718.7880, 1734.7794, 1740.7760, 1744.8013, 1754.7913, 1756.7547, 1770.7537, 1772.7239, 1838.9638, 1851.8058, 1862.8756, 1921.8557, 1924.8861, 1941.8985, 1948.8578, 1967.8661, 2005.8049, 2118.8044, 2170.8299, 2217.9647, 2234.8584, 2255.8811, 2265.2040, 2277.1368, 2566.1425, 2618.1737, 2625.1754, 2636.1875, 2663.1955, 2682.2062, 2695.2126, 2708.2152, 2720.1459, 2724.2164, 3347.7264

16. Q1RP80_VIPBN **Mass:** 16258 **Score:** 168 **Expect:** 2.2e-014 **Matches:** 6

Phospholipase A2 ammodytin I1 OS=Vipera berus nikolskii OX=1808362 GN=I1 PE=2 SV=1

Observed	Mr(expt)	Mr(calc)	ppm	Start	End	Miss	Ions	Peptide
909.3941	908.3868	908.3480	42.6	100 -	106	0	---	R.AVCECDR.V
1339.6437	1338.6364	1338.5988	28.1	123 -	132	0	56	K.YMLYSLFDCK.E
1355.6382	1354.6310	1354.5937	27.5	123 -	132	0	(11)	K.YMLYSLFDCK.E + Oxidation (M)
1732.8009	1731.7936	1731.7596	19.6	107 -	121	0	87	R.VAAICFGENMNTYDK.K
1748.7978	1747.7906	1747.7545	20.6	107 -	121	0	(55)	R.VAAICFGENMNTYDK.K + Oxidation (M)
1876.8772	1875.8699	1875.8495	10.9	107 -	122	1	---	R.VAAICFGENMNTYDKK.Y + Oxidation (M)

No match to: 817.1395, 837.1000, 839.1118, 852.3774, 855.0882, 859.0799, 861.0964, 868.5738, 871.0597, 874.0428, 877.0680, 882.6029, 903.4152, 905.5111, 987.5256, 1022.1471, 1044.1224, 1064.0902, 1066.1043, 1082.0794, 1149.5023, 1157.6635, 1175.6978, 1201.6609, 1222.6157, 1249.1322, 1252.6490, 1267.6375, 1271.1084, 1283.6283, 1289.6181, 1293.6475, 1305.5904, 1309.6407, 1320.6258, 1323.6473, 1345.6280, 1361.6097, 1365.6708, 1377.5960, 1393.5844, 1395.7311, 1411.7256, 1436.7603, 1475.7776, 1476.1123, 1557.8703, 1636.7539, 1707.8181, 1718.7880, 1734.7794, 1740.7760, 1744.8013, 1754.7913, 1756.7547, 1770.7537, 1772.7239, 1838.9638, 1851.8058, 1862.8756, 1921.8557, 1924.8861, 1941.8985, 1948.8578, 1967.8661, 2005.8049, 2118.8044, 2170.8299, 2217.9647, 2234.8584, 2255.8811, 2265.2040, 2277.1368, 2566.1425, 2618.1737, 2625.1754, 2636.1875, 2663.1955, 2682.2062, 2695.2126, 2708.2152, 2720.1459, 2724.2164, 3347.7264

17. Q6A3M8_VIPAM **Mass:** 16378 **Score:** 168 **Expect:** 2.2e-014 **Matches:** 6

Ammodytin I1(A) variant OS=Vipera ammodytes ruffoi OX=246549 PE=2 SV=1

Observed	Mr(expt)	Mr(calc)	ppm	Start	End	Miss	Ions	Peptide
909.3941	908.3868	908.3480	42.6	100 -	106	0	---	R.AVCECDR.V
1339.6437	1338.6364	1338.5988	28.1	123 -	132	0	56	K.YMLYSLFDCK.E
1355.6382	1354.6310	1354.5937	27.5	123 -	132	0	(11)	K.YMLYSLFDCK.E + Oxidation (M)
1732.8009	1731.7936	1731.7596	19.6	107 -	121	0	87	R.VAAICFGENMNTYDK.K
1748.7978	1747.7906	1747.7545	20.6	107 -	121	0	(55)	R.VAAICFGENMNTYDK.K + Oxidation (M)
1876.8772	1875.8699	1875.8495	10.9	107 -	122	1	---	R.VAAICFGENMNTYDKK.Y + Oxidation (M)

No match to: 817.1395, 837.1000, 839.1118, 852.3774, 855.0882, 859.0799, 861.0964, 868.5738, 871.0597, 874.0428, 877.0680, 882.6029, 903.4152, 905.5111, 987.5256, 1022.1471, 1044.1224, 1064.0902, 1066.1043, 1082.0794, 1149.5023, 1157.6635, 1175.6978, 1201.6609, 1222.6157, 1249.1322, 1252.6490, 1267.6375, 1271.1084, 1283.6283, 1289.6181, 1293.6475, 1305.5904, 1309.6407, 1320.6258, 1323.6473, 1345.6280, 1361.6097, 1365.6708, 1377.5960, 1393.5844, 1395.7311, 1411.7256, 1436.7603, 1475.7776, 1476.1123, 1557.8703, 1636.7539, 1707.8181, 1718.7880, 1734.7794, 1740.7760, 1744.8013, 1754.7913, 1756.7547, 1770.7537, 1772.7239, 1838.9638, 1851.8058, 1862.8756, 1921.8557, 1924.8861, 1941.8985, 1948.8578, 1967.8661, 2005.8049, 2118.8044, 2170.8299, 2217.9647, 2234.8584, 2255.8811, 2265.2040, 2277.1368, 2566.1425, 2618.1737, 2625.1754, 2636.1875, 2663.1955, 2682.2062, 2695.2126, 2708.2152, 2720.1459, 2724.2164, 3347.7264

18. Q6A3I8_VIPUR **Mass:** 16290 **Score:** 167 **Expect:** 2.8e-014 **Matches:** 6

Ammodytin I1(B) isoform OS=Vipera ursinii OX=103942 PE=2 SV=1

Observed	Mr(expt)	Mr(calc)	ppm	Start	End	Miss	Ions	Peptide
909.3941	908.3868	908.3480	42.6	100 -	106	0	---	R.AVCECDR.V
1339.6437	1338.6364	1338.5988	28.1	123 -	132	0	56	K.YMLYSLFDCK.E
1355.6382	1354.6310	1354.5937	27.5	123 -	132	0	(11)	K.YMLYSLFDCK.E + Oxidation (M)
1732.8009	1731.7936	1731.7596	19.6	107 -	121	0	87	R.VAAICFGENMNTYDK.K
1748.7978	1747.7906	1747.7545	20.6	107 -	121	0	(55)	R.VAAICFGENMNTYDK.K + Oxidation (M)
1876.8772	1875.8699	1875.8495	10.9	107 -	122	1	---	R.VAAICFGENMNTYDKK.Y + Oxidation (M)

No match to: 817.1395, 837.1000, 839.1118, 852.3774, 855.0882, 859.0799, 861.0964, 868.5738, 871.0597, 874.0428, 877.0680, 882.6029, 903.4152, 905.5111, 987.5256, 1022.1471, 1044.1224, 1064.0902, 1066.1043, 1082.0794, 1149.5023, 1157.6635, 1175.6978, 1201.6609, 1222.6157, 1249.1322, 1252.6490, 1267.6375, 1271.1084, 1283.6283, 1289.6181, 1293.6475, 1305.5904, 1309.6407, 1320.6258, 1323.6473, 1345.6280, 1361.6097, 1365.6708, 1377.5960, 1393.5844, 1395.7311, 1411.7256, 1436.7603, 1475.7776, 1476.1123, 1557.8703, 1636.7539, 1707.8181, 1718.7880, 1734.7794, 1740.7760, 1744.8013, 1754.7913, 1756.7547, 1770.7537, 1772.7239, 1838.9638, 1851.8058, 1862.8756, 1921.8557, 1924.8861, 1941.8985, 1948.8578, 1967.8661, 2005.8049, 2118.8044, 2170.8299, 2217.9647, 2234.8584, 2255.8811, 2265.2040, 2277.1368, 2566.1425, 2618.1737, 2625.1754, 2636.1875, 2663.1955, 2682.2062, 2695.2126, 2708.2152, 2720.1459, 2724.2164, 3347.7264

19. Q6A3M9_VIPAM **Mass:** 16286 **Score:** 166 **Expect:** 3.6e-014 **Matches:** 6

Ammodytin I1(B) isoform OS=Vipera ammodytes ruffoi OX=246549 PE=2 SV=1

Observed	Mr(expt)	Mr(calc)	ppm	Start	End	Miss	Ions	Peptide
909.3941	908.3868	908.3480	42.6	100 -	106	0	---	R.AVCECDR.V
1339.6437	1338.6364	1338.5988	28.1	123 -	132	0	56	K.YMLYSLFDCK.E
1355.6382	1354.6310	1354.5937	27.5	123 -	132	0	(11)	K.YMLYSLFDCK.E + Oxidation (M)
1732.8009	1731.7936	1731.7596	19.6	107 -	121	0	87	R.VAAICFGENMNTYDK.K
1748.7978	1747.7906	1747.7545	20.6	107 -	121	0	(55)	R.VAAICFGENMNTYDK.K + Oxidation (M)
1876.8772	1875.8699	1875.8495	10.9	107 -	122	1	---	R.VAAICFGENMNTYDK.Y + Oxidation (M)

No match to: 817.1395, 837.1000, 839.1118, 852.3774, 855.0882, 859.0799, 861.0964, 868.5738, 871.0597, 874.0428, 877.0680, 882.6029, 903.4152, 905.5111, 987.5256, 1022.1471, 1044.1224, 1064.0902, 1066.1043, 1082.0794, 1149.5023, 1157.6635, 1175.6978, 1201.6609, 1222.6157, 1249.1322, 1252.6490, 1267.6375, 1271.1084, 1283.6283, 1289.6181, 1293.6475, 1305.5904, 1309.6407, 1320.6258, 1323.6473, 1345.6280, 1361.6097, 1365.6708, 1377.5960, 1393.5844, 1395.7311, 1411.7256, 1436.7603, 1475.7776, 1476.1123, 1557.8703, 1636.7539, 1707.8181, 1718.7880, 1734.7794, 1740.7760, 1744.8013, 1754.7913, 1756.7547, 1770.7537, 1772.7239, 1838.9638, 1851.8058, 1862.8756, 1921.8557, 1924.8861, 1941.8985, 1948.8578, 1967.8661, 2005.8049, 2118.8044, 2170.8299, 2217.9647, 2234.8584, 2255.8811, 2265.2040, 2277.1368, 2566.1425, 2618.1737, 2625.1754, 2636.1875, 2663.1955, 2682.2062, 2695.2126, 2708.2152, 2720.1459, 2724.2164, 3347.7264

20. PA2A7_DABPA **Mass:** 16190 **Score:** 129 **Expect:** 1.8e-010 **Matches:** 4

Acidic phospholipase A2 VP7 OS=Daboia palaestinae OX=1170828 PE=3 SV=1

Observed	Mr(expt)	Mr(calc)	ppm	Start	End	Miss	Ions	Peptide
909.3941	908.3868	908.3480	42.6	100 -	106	0	---	R.AVCECDR.V
987.5256	986.5184	986.4781	40.8	50 -	58	0	3	K.GKPQDATDR.C
2682.2062	2681.1989	2681.1374	22.9	77 -	99	0	98	K.LSTYSYSFQNGDIVCGDDDPCLR.A
2724.2164	2723.2091	2723.1448	23.6	100 -	122	1	---	R.AVCECDRVAAICFGENMNTYDTK.Y

No match to: 817.1395, 837.1000, 839.1118, 852.3774, 855.0882, 859.0799, 861.0964, 868.5738, 871.0597, 874.0428, 877.0680, 882.6029, 903.4152, 905.5111, 1022.1471, 1044.1224, 1064.0902, 1066.1043, 1082.0794, 1149.5023, 1157.6635, 1175.6978, 1201.6609, 1222.6157, 1249.1322, 1252.6490, 1267.6375, 1271.1084, 1283.6283, 1289.6181, 1293.6475, 1305.5904, 1309.6407, 1320.6258, 1323.6473, 1339.6437, 1345.6280, 1355.6382, 1361.6097, 1365.6708, 1377.5960, 1393.5844, 1395.7311, 1411.7256, 1436.7603, 1475.7776, 1476.1123, 1557.8703, 1636.7539, 1707.8181, 1718.7880, 1732.8009, 1734.7794, 1740.7760, 1744.8013, 1748.7978, 1754.7913, 1756.7547, 1770.7537, 1772.7239, 1838.9638, 1851.8058, 1862.8756, 1876.8772, 1921.8557, 1924.8861, 1941.8985, 1948.8578, 1967.8661, 2005.8049, 2118.8044, 2170.8299, 2217.9647, 2234.8584, 2255.8811, 2265.2040, 2277.1368, 2566.1425, 2618.1737, 2625.1754, 2636.1875, 2663.1955, 2695.2126, 2708.2152, 2720.1459, 3347.7264

21. Q6A3H1_VIPAS **Mass:** 16292 **Score:** 127 **Expect:** 2.8e-010 **Matches:** 7

Ammodytin I1(C) variant OS=Vipera aspis atra OX=246182 PE=2 SV=1

Observed	Mr(expt)	Mr(calc)	ppm	Start	End	Miss	Ions	Peptide
909.3941	908.3868	908.3480	42.6	100 -	106	0	---	R.AVCECDR.V
1339.6437	1338.6364	1338.5988	28.1	123 -	132	0	4	K.YMFYSLLDCK.E
1355.6382	1354.6310	1354.5937	27.5	123 -	132	0	---	K.YMFYSLLDCK.E + Oxidation (M)
1732.8009	1731.7936	1731.7596	19.6	107 -	121	0	87	R.VAAICFGENMNTYDK.K
1748.7978	1747.7906	1747.7545	20.6	107 -	121	0	(55)	R.VAAICFGENMNTYDK.K + Oxidation (M)

1876.8772	1875.8699	1875.8495	10.9	107	122	1	---	R.VAAICFGENMNTYDKK.Y + Oxidation (M)
2625.1754	2624.1681	2624.1159	19.9	77	99	0	---	K.LSTYSYSFENGDIVCGGDDPCLR.A

No match to: 817.1395, 837.1000, 839.1118, 852.3774, 855.0882, 859.0799, 861.0964, 868.5738, 871.0597, 874.0428, 877.0680, 882.6029, 903.4152, 905.5111, 987.5256, 1022.1471, 1044.1224, 1064.0902, 1066.1043, 1082.0794, 1149.5023, 1157.6635, 1175.6978, 1201.6609, 1222.6157, 1249.1322, 1252.6490, 1267.6375, 1271.1084, 1283.6283, 1289.6181, 1293.6475, 1305.5904, 1309.6407, 1320.6258, 1323.6473, 1345.6280, 1361.6097, 1365.6708, 1377.5960, 1393.5844, 1395.7311, 1411.7256, 1436.7603, 1475.7776, 1476.1123, 1557.8703, 1636.7539, 1707.8181, 1718.7880, 1734.7794, 1740.7760, 1744.8013, 1754.7913, 1756.7547, 1770.7537, 1772.7239, 1838.9638, 1851.8058, 1862.8756, 1921.8557, 1924.8861, 1941.8985, 1948.8578, 1967.8661, 2005.8049, 2118.8044, 2170.8299, 2217.9647, 2234.8584, 2255.8811, 2265.2040, 2277.1368, 2566.1425, 2618.1737, 2636.1875, 2663.1955, 2682.2062, 2695.2126, 2708.2152, 2720.1459, 2724.2164, 3347.7264

22. Q6A3J3_VIPBB **Mass:** 16280 **Score:** 123 **Expect:** 7.1e-010 **Matches:** 6
Ammodytin I1(C) variant OS=Vipera berus berus OX=31156 PE=2 SV=1

Observed	Mr(expt)	Mr(calc)	ppm	Start	End	Miss	Ions	Peptide
909.3941	908.3868	908.3480	42.6	100	106	0	---	R.AVCECDR.V
1305.5904	1304.5831	1304.6145	-24.02	123	132	0	---	K.YMLYSLLDCK.E
1732.8009	1731.7936	1731.7596	19.6	107	121	0	87	R.VAAICFGENMNTYDK.K
1748.7978	1747.7906	1747.7545	20.6	107	121	0	(55)	R.VAAICFGENMNTYDK.K + Oxidation (M)
1876.8772	1875.8699	1875.8495	10.9	107	122	1	---	R.VAAICFGENMNTYDKK.Y + Oxidation (M)
2625.1754	2624.1681	2624.1159	19.9	77	99	0	---	K.LSTYSYSFENGDIVCGGDDPCLR.A

No match to: 817.1395, 837.1000, 839.1118, 852.3774, 855.0882, 859.0799, 861.0964, 868.5738, 871.0597, 874.0428, 877.0680, 882.6029, 903.4152, 905.5111, 987.5256, 1022.1471, 1044.1224, 1064.0902, 1066.1043, 1082.0794, 1149.5023, 1157.6635, 1175.6978, 1201.6609, 1222.6157, 1249.1322, 1252.6490, 1267.6375, 1271.1084, 1283.6283, 1289.6181, 1293.6475, 1309.6407, 1320.6258, 1323.6473, 1339.6437, 1345.6280, 1355.6382, 1361.6097, 1365.6708, 1377.5960, 1393.5844, 1395.7311, 1411.7256, 1436.7603, 1475.7776, 1476.1123, 1557.8703, 1636.7539, 1707.8181, 1718.7880, 1734.7794, 1740.7760, 1744.8013, 1754.7913, 1756.7547, 1770.7537, 1772.7239, 1838.9638, 1851.8058, 1862.8756, 1921.8557, 1924.8861, 1941.8985, 1948.8578, 1967.8661, 2005.8049, 2118.8044, 2170.8299, 2217.9647, 2234.8584, 2255.8811, 2265.2040, 2277.1368, 2566.1425, 2618.1737, 2636.1875, 2663.1955, 2682.2062, 2695.2126, 2708.2152, 2720.1459, 2724.2164, 3347.7264

23. Q6A3H6_VIPAS **Mass:** 16258 **Score:** 123 **Expect:** 7.1e-010 **Matches:** 6
Ammodytin I1(C) isoform OS=Vipera aspis atra OX=246182 PE=2 SV=1

Observed	Mr(expt)	Mr(calc)	ppm	Start	End	Miss	Ions	Peptide
909.3941	908.3868	908.3480	42.6	100	106	0	---	R.AVCECDR.V
1305.5904	1304.5831	1304.6145	-24.02	123	132	0	---	K.YMLYSLLDCK.E
1732.8009	1731.7936	1731.7596	19.6	107	121	0	87	R.VAAICFGENMNTYDK.K
1748.7978	1747.7906	1747.7545	20.6	107	121	0	(55)	R.VAAICFGENMNTYDK.K + Oxidation (M)
1876.8772	1875.8699	1875.8495	10.9	107	122	1	---	R.VAAICFGENMNTYDKK.Y + Oxidation (M)
2625.1754	2624.1681	2624.1159	19.9	77	99	0	---	K.LSTYSYSFENGDIVCGGDDPCLR.A

No match to: 817.1395, 837.1000, 839.1118, 852.3774, 855.0882, 859.0799, 861.0964, 868.5738, 871.0597, 874.0428, 877.0680, 882.6029, 903.4152, 905.5111, 987.5256, 1022.1471, 1044.1224, 1064.0902, 1066.1043, 1082.0794, 1149.5023, 1157.6635, 1175.6978, 1201.6609, 1222.6157, 1249.1322, 1252.6490, 1267.6375, 1271.1084, 1283.6283, 1289.6181, 1293.6475, 1309.6407, 1320.6258, 1323.6473, 1339.6437, 1345.6280, 1355.6382, 1361.6097, 1365.6708, 1377.5960, 1393.5844, 1395.7311, 1411.7256, 1436.7603, 1475.7776, 1476.1123, 1557.8703, 1636.7539, 1707.8181, 1718.7880, 1734.7794, 1740.7760, 1744.8013, 1754.7913, 1756.7547, 1770.7537, 1772.7239, 1838.9638, 1851.8058, 1862.8756, 1921.8557, 1924.8861, 1941.8985, 1948.8578, 1967.8661, 2005.8049, 2118.8044, 2170.8299, 2217.9647, 2234.8584, 2255.8811, 2265.2040, 2277.1368, 2566.1425, 2618.1737, 2636.1875, 2663.1955, 2682.2062, 2695.2126, 2708.2152, 2720.1459, 2724.2164, 3347.7264

24. Q6A3J2_VIPBB **Mass:** 16244 **Score:** 123 **Expect:** 7.1e-010 **Matches:** 6
Ammodytin I1(C) variant OS=Vipera berus berus OX=31156 PE=2 SV=1

Observed	Mr(expt)	Mr(calc)	ppm	Start	End	Miss	Ions	Peptide
909.3941	908.3868	908.3480	42.6	100	106	0	---	R.AVCECDR.V
1305.5904	1304.5831	1304.6145	-24.02	123	132	0	---	K.YMLYSLLDCK.E
1732.8009	1731.7936	1731.7596	19.6	107	121	0	87	R.VAAICFGENMNTYDK.K
1748.7978	1747.7906	1747.7545	20.6	107	121	0	(55)	R.VAAICFGENMNTYDK.K + Oxidation (M)
1876.8772	1875.8699	1875.8495	10.9	107	122	1	---	R.VAAICFGENMNTYDKK.Y + Oxidation (M)
2625.1754	2624.1681	2624.1159	19.9	77	99	0	---	K.LSTYSYSFENGDIVCGGDDPCLR.A

No match to: 817.1395, 837.1000, 839.1118, 852.3774, 855.0882, 859.0799, 861.0964, 868.5738, 871.0597, 874.0428, 877.0680, 882.6029, 903.4152, 905.5111, 987.5256, 1022.1471, 1044.1224, 1064.0902, 1066.1043, 1082.0794, 1149.5023, 1157.6635, 1175.6978, 1201.6609, 1222.6157, 1249.1322, 1252.6490, 1267.6375, 1271.1084, 1283.6283, 1289.6181, 1293.6475, 1309.6407, 1320.6258, 1323.6473, 1339.6437, 1345.6280, 1355.6382, 1361.6097, 1365.6708, 1377.5960, 1393.5844, 1395.7311, 1411.7256, 1436.7603, 1475.7776, 1476.1123, 1557.8703, 1636.7539, 1707.8181, 1718.7880, 1734.7794, 1740.7760, 1744.8013, 1754.7913, 1756.7547, 1770.7537, 1772.7239, 1838.9638, 1851.8058, 1862.8756, 1921.8557, 1924.8861, 1941.8985, 1948.8578, 1967.8661, 2005.8049, 2118.8044, 2170.8299, 2217.9647, 2234.8584, 2255.8811, 2265.2040, 2277.1368, 2566.1425, 2618.1737, 2636.1875, 2663.1955, 2682.2062, 2695.2126, 2708.2152, 2720.1459, 2724.2164, 3347.7264

25. Q6A3J0_VIPBB **Mass:** 16280 **Score:** 123 **Expect:** 7.1e-010 **Matches:** 6
Ammodytin I1(C) variant OS=Vipera berus berus OX=31156 PE=2 SV=1

Observed	Mr(expt)	Mr(calc)	ppm	Start	End	Miss	Ions	Peptide
909.3941	908.3868	908.3480	42.6	100	106	0	---	R.AVCECDR.V
1305.5904	1304.5831	1304.6145	-24.02	123	132	0	---	K.YMLYSLLDCK.E
1732.8009	1731.7936	1731.7596	19.6	107	121	0	87	R.VAAICFGENMNTYDK.K
1748.7978	1747.7906	1747.7545	20.6	107	121	0	(55)	R.VAAICFGENMNTYDK.K + Oxidation (M)
1876.8772	1875.8699	1875.8495	10.9	107	122	1	---	R.VAAICFGENMNTYDKK.Y + Oxidation (M)
2625.1754	2624.1681	2624.1159	19.9	77	99	0	---	K.LSTYSYSFENGDIVCGGDDPCLR.A

No match to: 817.1395, 837.1000, 839.1118, 852.3774, 855.0882, 859.0799, 861.0964, 868.5738, 871.0597, 874.0428, 877.0680, 882.6029, 903.4152, 905.5111, 987.5256, 1022.1471, 1044.1224, 1064.0902, 1066.1043, 1082.0794, 1149.5023, 1157.6635, 1175.6978, 1201.6609, 1222.6157, 1249.1322, 1252.6490, 1267.6375, 1271.1084, 1283.6283, 1289.6181, 1293.6475, 1309.6407, 1320.6258, 1323.6473, 1339.6437, 1345.6280, 1355.6382, 1361.6097, 1365.6708, 1377.5960, 1393.5844, 1395.7311, 1411.7256, 1436.7603, 1475.7776, 1476.1123, 1557.8703, 1636.7539, 1707.8181, 1718.7880, 1734.7794, 1740.7760, 1744.8013, 1754.7913, 1756.7547, 1770.7537, 1772.7239, 1838.9638, 1851.8058, 1862.8756, 1921.8557, 1924.8861, 1941.8985, 1948.8578, 1967.8661, 2005.8049, 2118.8044, 2170.8299, 2217.9647, 2234.8584, 2255.8811, 2265.2040, 2277.1368, 2566.1425, 2618.1737, 2636.1875, 2663.1955, 2682.2062, 2695.2126, 2708.2152, 2720.1459, 2724.2164, 3347.7264

26. Q6A3H7_VIPAS **Mass:** 16244 **Score:** 123 **Expect:** 7.1e-010 **Matches:** 6
Ammodytin I1(F) isoform OS=Vipera aspis atra OX=246182 PE=2 SV=1

Observed	Mr(expt)	Mr(calc)	ppm	Start	End	Miss	Ions	Peptide
909.3941	908.3868	908.3480	42.6	100	106	0	---	R.AVCECDR.V
1305.5904	1304.5831	1304.6145	-24.02	123	132	0	---	K.YMLYSLLDCK.E
1732.8009	1731.7936	1731.7596	19.6	107	121	0	87	R.VAAICFGENMNTYDK.K
1748.7978	1747.7906	1747.7545	20.6	107	121	0	(55)	R.VAAICFGENMNTYDK.K + Oxidation (M)
1876.8772	1875.8699	1875.8495	10.9	107	122	1	---	R.VAAICFGENMNTYDKK.Y + Oxidation (M)
2625.1754	2624.1681	2624.1159	19.9	77	99	0	---	K.LSTYSYSFENGDIVCGGDDPCLR.A

No match to: 817.1395, 837.1000, 839.1118, 852.3774, 855.0882, 859.0799, 861.0964, 868.5738, 871.0597, 874.0428, 877.0680, 882.6029, 903.4152, 905.5111, 987.5256, 1022.1471, 1044.1224, 1064.0902, 1066.1043, 1082.0794, 1149.5023, 1157.6635, 1175.6978, 1201.6609, 1222.6157, 1249.1322, 1252.6490, 1267.6375, 1271.1084, 1283.6283, 1289.6181, 1293.6475, 1309.6407, 1320.6258, 1323.6473, 1339.6437, 1345.6280, 1355.6382, 1361.6097, 1365.6708, 1377.5960, 1393.5844, 1395.7311, 1411.7256, 1436.7603, 1475.7776, 1476.1123, 1557.8703, 1636.7539, 1707.8181, 1718.7880, 1734.7794, 1740.7760, 1744.8013, 1754.7913, 1756.7547, 1770.7537, 1772.7239, 1838.9638, 1851.8058, 1862.8756, 1921.8557, 1924.8861, 1941.8985, 1948.8578, 1967.8661, 2005.8049, 2118.8044, 2170.8299, 2217.9647, 2234.8584, 2255.8811, 2265.2040, 2277.1368, 2566.1425, 2618.1737, 2636.1875, 2663.1955, 2682.2062, 2695.2126, 2708.2152, 2720.1459, 2724.2164, 3347.7264

27. Q6A3H4_VIPAS **Mass:** 16244 **Score:** 123 **Expect:** 7.1e-010 **Matches:** 6

Ammodytin I1(C) isoform OS=Vipera aspis atra OX=246182 PE=2 SV=1

Observed	Mr(expt)	Mr(calc)	ppm	Start	End	Miss	Ions	Peptide
909.3941	908.3868	908.3480	42.6	100 -	106	0	---	R.AVCECDR.V
1305.5904	1304.5831	1304.6145	-24.02	123 -	132	0	---	K.YMLYSLLDCK.E
1732.8009	1731.7936	1731.7596	19.6	107 -	121	0	87	R.VAAICFGENMNTYDK.K
1748.7978	1747.7906	1747.7545	20.6	107 -	121	0	(55)	R.VAAICFGENMNTYDK.K + Oxidation (M)
1876.8772	1875.8699	1875.8495	10.9	107 -	122	1	---	R.VAAICFGENMNTYDKK.Y + Oxidation (M)
2625.1754	2624.1681	2624.1159	19.9	77 -	99	0	---	K.LSTYSYSFENGDIVCGGDDPCLR.A

No match to: 817.1395, 837.1000, 839.1118, 852.3774, 855.0882, 859.0799, 861.0964, 868.5738, 871.0597, 874.0428, 877.0680, 882.6029, 903.4152, 905.5111, 987.5256, 1022.1471, 1044.1224, 1064.0902, 1066.1043, 1082.0794, 1149.5023, 1157.6635, 1175.6978, 1201.6609, 1222.6157, 1249.1322, 1252.6490, 1267.6375, 1271.1084, 1283.6283, 1289.6181, 1293.6475, 1309.6407, 1320.6258, 1323.6473, 1339.6437, 1345.6280, 1355.6382, 1361.6097, 1365.6708, 1377.5960, 1393.5844, 1395.7311, 1411.7256, 1436.7603, 1475.7776, 1476.1123, 1557.8703, 1636.7539, 1707.8181, 1718.7880, 1740.7760, 1744.8013, 1754.7913, 1756.7547, 1770.7537, 1772.7239, 1838.9638, 1851.8058, 1862.8756, 1921.8557, 1924.8861, 1941.8985, 1948.8578, 1967.8661, 2005.8049, 2118.8044, 2170.8299, 2217.9647, 2234.8584, 2255.8811, 2265.2040, 2277.1368, 2566.1425, 2618.1737, 2636.1875, 2663.1955, 2682.2062, 2695.2126, 2708.2152, 2720.1459, 2724.2164, 3347.7264

28. Q7T1D3_VIPAS **Mass:** 16258 **Score:** 123 **Expect:** 7.1e-010 **Matches:** 6

Ammodytin I1 isoform 1 OS=Vipera aspis OX=8706 GN=AmtI1 PE=3 SV=1

Observed	Mr(expt)	Mr(calc)	ppm	Start	End	Miss	Ions	Peptide
909.3941	908.3868	908.3480	42.6	100 -	106	0	---	R.AVCECDR.V
1305.5904	1304.5831	1304.6145	-24.02	123 -	132	0	---	K.YMLYSLLDCK.E
1732.8009	1731.7936	1731.7596	19.6	107 -	121	0	87	R.VAAICFGENMNTYDK.K
1748.7978	1747.7906	1747.7545	20.6	107 -	121	0	(55)	R.VAAICFGENMNTYDK.K + Oxidation (M)
1876.8772	1875.8699	1875.8495	10.9	107 -	122	1	---	R.VAAICFGENMNTYDKK.Y + Oxidation (M)
2625.1754	2624.1681	2624.1159	19.9	77 -	99	0	---	K.LSTYSYSFENGDIVCGGDDPCLR.A

No match to: 817.1395, 837.1000, 839.1118, 852.3774, 855.0882, 859.0799, 861.0964, 868.5738, 871.0597, 874.0428, 877.0680, 882.6029, 903.4152, 905.5111, 987.5256, 1022.1471, 1044.1224, 1064.0902, 1066.1043, 1082.0794, 1149.5023, 1157.6635, 1175.6978, 1201.6609, 1222.6157, 1249.1322, 1252.6490, 1267.6375, 1271.1084, 1283.6283, 1289.6181, 1293.6475, 1309.6407, 1320.6258, 1323.6473, 1339.6437, 1345.6280, 1355.6382, 1361.6097, 1365.6708, 1377.5960, 1393.5844, 1395.7311, 1411.7256, 1436.7603, 1475.7776, 1476.1123, 1557.8703, 1636.7539, 1707.8181, 1718.7880, 1734.7794, 1740.7760, 1744.8013, 1754.7913, 1756.7547, 1770.7537, 1772.7239, 1838.9638, 1851.8058, 1862.8756, 1921.8557, 1924.8861, 1941.8985, 1948.8578, 1967.8661, 2005.8049, 2118.8044, 2170.8299, 2217.9647, 2234.8584, 2255.8811, 2265.2040, 2277.1368, 2566.1425, 2618.1737, 2636.1875, 2663.1955, 2682.2062, 2695.2126, 2708.2152, 2720.1459, 2724.2164, 3347.7264

29. Q6A3I1_VIPAP **Mass:** 16288 **Score:** 123 **Expect:** 7.1e-010 **Matches:** 6

Ammodytin I1(C) variant OS=Vipera aspis aspis OX=194601 PE=2 SV=1

Observed	Mr(expt)	Mr(calc)	ppm	Start	End	Miss	Ions	Peptide
909.3941	908.3868	908.3480	42.6	100 -	106	0	---	R.AVCECDR.V
1305.5904	1304.5831	1304.6145	-24.02	123 -	132	0	---	K.YMLYSLLDCK.E
1732.8009	1731.7936	1731.7596	19.6	107 -	121	0	87	R.VAAICFGENMNTYDK.K
1748.7978	1747.7906	1747.7545	20.6	107 -	121	0	(55)	R.VAAICFGENMNTYDK.K + Oxidation (M)
1876.8772	1875.8699	1875.8495	10.9	107 -	122	1	---	R.VAAICFGENMNTYDKK.Y + Oxidation (M)
2625.1754	2624.1681	2624.1159	19.9	77 -	99	0	---	K.LSTYSYSFENGDIVCGGDDPCLR.A

No match to: 817.1395, 837.1000, 839.1118, 852.3774, 855.0882, 859.0799, 861.0964, 868.5738, 871.0597, 874.0428, 877.0680, 882.6029, 903.4152, 905.5111, 987.5256, 1022.1471, 1044.1224, 1064.0902, 1066.1043, 1082.0794, 1149.5023, 1157.6635, 1175.6978, 1201.6609, 1222.6157, 1249.1322, 1252.6490, 1267.6375, 1271.1084, 1283.6283, 1289.6181, 1293.6475, 1309.6407, 1320.6258, 1323.6473, 1339.6437, 1345.6280, 1355.6382, 1361.6097, 1365.6708, 1377.5960, 1393.5844, 1395.7311, 1411.7256, 1436.7603, 1475.7776, 1476.1123, 1557.8703, 1636.7539, 1707.8181, 1718.7880, 1734.7794, 1740.7760, 1744.8013, 1754.7913, 1756.7547, 1770.7537, 1772.7239, 1838.9638, 1851.8058, 1862.8756, 1921.8557, 1924.8861, 1941.8985, 1948.8578, 1967.8661, 2005.8049, 2118.8044, 2170.8299, 2217.9647, 2234.8584, 2255.8811, 2265.2040, 2277.1368, 2566.1425, 2618.1737, 2636.1875, 2663.1955, 2682.2062, 2695.2126, 2708.2152, 2720.1459, 2724.2164, 3347.7264

30. Q6A3I0_VIPAP **Mass:** 16272 **Score:** 123 **Expect:** 7.1e-010 **Matches:** 6

Ammodytin I1(C) variant OS=Vipera aspis aspis OX=194601 PE=2 SV=1

Observed	Mr(expt)	Mr(calc)	ppm	Start	End	Miss	Ions	Peptide
909.3941	908.3868	908.3480	42.6	100 -	106	0	---	R.AVCECDR.V
1305.5904	1304.5831	1304.6145	-24.02	123 -	132	0	---	K.YMLYSLLDCK.E
1732.8009	1731.7936	1731.7596	19.6	107 -	121	0	87	R.VAAICFGENMNTYDK.K
1748.7978	1747.7906	1747.7545	20.6	107 -	121	0	(55)	R.VAAICFGENMNTYDK.K + Oxidation (M)
1876.8772	1875.8699	1875.8495	10.9	107 -	122	1	---	R.VAAICFGENMNTYDKK.Y + Oxidation (M)
2625.1754	2624.1681	2624.1159	19.9	77 -	99	0	---	K.LSTYSYSFENGDIVCGGDDPCLR.A

No match to: 817.1395, 837.1000, 839.1118, 852.3774, 855.0882, 859.0799, 861.0964, 868.5738, 871.0597, 874.0428, 877.0680, 882.6029, 903.4152, 905.5111, 987.5256, 1022.1471, 1044.1224, 1064.0902, 1066.1043, 1082.0794, 1149.5023, 1157.6635, 1175.6978, 1201.6609, 1222.6157, 1249.1322, 1252.6490, 1267.6375, 1271.1084, 1283.6283, 1289.6181, 1293.6475, 1309.6407, 1320.6258, 1323.6473, 1339.6437, 1345.6280, 1355.6382, 1361.6097, 1365.6708, 1377.5960, 1393.5844, 1395.7311, 1411.7256, 1436.7603, 1475.7776, 1476.1123, 1557.8703, 1636.7539, 1707.8181, 1718.7880, 1734.7794, 1740.7760, 1744.8013, 1754.7913, 1756.7547, 1770.7537, 1772.7239, 1838.9638, 1851.8058, 1862.8756, 1921.8557, 1924.8861, 1941.8985, 1948.8578, 1967.8661, 2005.8049, 2118.8044, 2170.8299, 2217.9647, 2234.8584, 2255.8811, 2265.2040, 2277.1368, 2566.1425, 2618.1737, 2636.1875, 2663.1955, 2682.2062, 2695.2126, 2708.2152, 2720.1459, 2724.2164, 3347.7264

31. Q6A3H8_VIPAP **Mass:** 16330 **Score:** 123 **Expect:** 7.1e-010 **Matches:** 6

Ammodytin I1(C) variant OS=Vipera aspis aspis OX=194601 PE=2 SV=1

Observed	Mr(expt)	Mr(calc)	ppm	Start	End	Miss	Ions	Peptide
909.3941	908.3868	908.3480	42.6	100 -	106	0	---	R.AVCECDR.V
1305.5904	1304.5831	1304.6145	-24.02	123 -	132	0	---	K.YMLYSLLDCK.E
1732.8009	1731.7936	1731.7596	19.6	107 -	121	0	87	R.VAAICFGENMNTYDK.K
1748.7978	1747.7906	1747.7545	20.6	107 -	121	0	(55)	R.VAAICFGENMNTYDK.K + Oxidation (M)
1876.8772	1875.8699	1875.8495	10.9	107 -	122	1	---	R.VAAICFGENMNTYDKK.Y + Oxidation (M)
2625.1754	2624.1681	2624.1159	19.9	77 -	99	0	---	K.LSTYSYSFENGDIVCGGDDPCLR.A

No match to: 817.1395, 837.1000, 839.1118, 852.3774, 855.0882, 859.0799, 861.0964, 868.5738, 871.0597, 874.0428, 877.0680, 882.6029, 903.4152, 905.5111, 987.5256, 1022.1471, 1044.1224, 1064.0902, 1066.1043, 1082.0794, 1149.5023, 1157.6635, 1175.6978, 1201.6609, 1222.6157, 1249.1322, 1252.6490, 1267.6375, 1271.1084, 1283.6283, 1289.6181, 1293.6475, 1309.6407, 1320.6258, 1323.6473, 1339.6437, 1345.6280, 1355.6382, 1361.6097, 1365.6708, 1377.5960, 1393.5844, 1395.7311, 1411.7256, 1436.7603, 1475.7776, 1476.1123, 1557.8703, 1636.7539, 1707.8181, 1718.7880, 1734.7794, 1740.7760, 1744.8013, 1754.7913, 1756.7547, 1770.7537, 1772.7239, 1838.9638, 1851.8058, 1862.8756, 1921.8557, 1924.8861, 1941.8985, 1948.8578, 1967.8661, 2005.8049, 2118.8044, 2170.8299, 2217.9647, 2234.8584, 2255.8811, 2265.2040, 2277.1368, 2566.1425, 2618.1737, 2636.1875, 2663.1955, 2682.2062, 2695.2126, 2708.2152, 2720.1459, 2724.2164, 3347.7264

32. Q6A3M5_VIPAP **Mass:** 16257 **Score:** 123 **Expect:** 7.1e-010 **Matches:** 6

Ammodytin I1(C) variant OS=Vipera aspis aspis OX=194601 PE=2 SV=1

Observed	Mr(expt)	Mr(calc)	ppm	Start	End	Miss	Ions	Peptide
909.3941	908.3868	908.3480	42.6	100 -	106	0	---	R.AVCECDR.V
1305.5904	1304.5831	1304.6145	-24.02	123 -	132	0	---	K.YMLYSLLDCK.E
1732.8009	1731.7936	1731.7596	19.6	107 -	121	0	87	R.VAAICFGENMNTYDK.K
1748.7978	1747.7906	1747.7545	20.6	107 -	121	0	(55)	R.VAAICFGENMNTYDK.K + Oxidation (M)
1876.8772	1875.8699	1875.8495	10.9	107 -	122	1	---	R.VAAICFGENMNTYDKK.Y + Oxidation (M)
2625.1754	2624.1681	2624.1159	19.9	77 -	99	0	---	K.LSTYSYSFENGDIVCGGDDPCLR.A

No match to: 817.1395, 837.1000, 839.1118, 852.3774, 855.0882, 859.0799, 861.0964, 868.5738, 871.0597, 874.0428, 877.0680, 882.6029, 903.4152, 905.5111, 987.5256, 1022.1471, 1044.1224, 1064.0902, 1066.1043, 1082.0794, 1149.5023, 1157.6635, 1175.6978, 1201.6609,

1222.6157, 1249.1322, 1252.6490, 1267.6375, 1271.1084, 1283.6283, 1289.6181, 1293.6475, 1309.6407, 1320.6258, 1323.6473, 1339.6437, 1345.6280, 1355.6382, 1361.6097, 1365.6708, 1377.5960, 1393.5844, 1395.7311, 1411.7256, 1436.7603, 1475.7776, 1476.1123, 1557.8703, 1636.7539, 1707.8181, 1718.7880, 1734.7794, 1740.7760, 1744.8013, 1754.7913, 1756.7547, 1770.7537, 1772.7239, 1838.9638, 1851.8058, 1862.8756, 1921.8557, 1924.8861, 1941.8985, 1948.8578, 1967.8661, 2005.8049, 2118.8044, 2170.8299, 2217.9647, 2234.8584, 2255.8811, 2265.2040, 2277.1368, 2566.1425, 2618.1737, 2636.1875, 2663.1955, 2682.2062, 2695.2126, 2708.2152, 2720.1459, 2724.2164, 3347.7264

33. Q6A3G1_VIPAP **Mass:** 16246 **Score:** 123 **Expect:** 7.1e-010 **Matches:** 6

Ammodytin I1(C') isoform OS=Vipera aspis aspis OX=194601 PE=2 SV=1

Observed	Mr(expt)	Mr(calc)	ppm	Start	End	Miss	Ions	Peptide
909.3941	908.3868	908.3480	42.6	100	106	0	---	R.AVCECDR.V
1305.5904	1304.5831	1304.6145	-24.02	123	132	0	---	K.YMLYSLLDCK.E
1732.8009	1731.7936	1731.7596	19.6	107	121	0	87	R.VAAICFGENMNTYDK.K
1748.7978	1747.7906	1747.7545	20.6	107	121	0	(55)	R.VAAICFGENMNTYDK.K + Oxidation (M)
1876.8772	1875.8699	1875.8495	10.9	107	122	1	---	R.VAAICFGENMNTYDKK.Y + Oxidation (M)
2625.1754	2624.1681	2624.1159	19.9	77	99	0	---	K.LSTYSYSFENGDIVCGGDDPCLR.A

No match to: 817.1395, 837.1000, 839.1118, 852.3774, 855.0882, 859.0799, 861.0964, 868.5738, 871.0597, 874.0428, 877.0680, 882.6029, 903.4152, 905.5111, 987.5256, 1022.1471, 1044.1224, 1064.0902, 1066.1043, 1082.0794, 1149.5023, 1157.6635, 1175.6978, 1201.6609, 1222.6157, 1249.1322, 1252.6490, 1267.6375, 1271.1084, 1283.6283, 1289.6181, 1293.6475, 1309.6407, 1320.6258, 1323.6473, 1339.6437, 1345.6280, 1355.6382, 1361.6097, 1365.6708, 1377.5960, 1393.5844, 1395.7311, 1411.7256, 1436.7603, 1475.7776, 1476.1123, 1557.8703, 1636.7539, 1707.8181, 1718.7880, 1734.7794, 1740.7760, 1744.8013, 1754.7913, 1756.7547, 1770.7537, 1772.7239, 1838.9638, 1851.8058, 1862.8756, 1921.8557, 1924.8861, 1941.8985, 1948.8578, 1967.8661, 2005.8049, 2118.8044, 2170.8299, 2217.9647, 2234.8584, 2255.8811, 2265.2040, 2277.1368, 2566.1425, 2618.1737, 2636.1875, 2663.1955, 2682.2062, 2695.2126, 2708.2152, 2720.1459, 2724.2164, 3347.7264

34. Q7T1D4_VIPAP **Mass:** 16258 **Score:** 123 **Expect:** 7.1e-010 **Matches:** 6

Ammodytin I1 OS=Vipera aspis aspis OX=194601 GN=AmtI1 PE=2 SV=1

Observed	Mr(expt)	Mr(calc)	ppm	Start	End	Miss	Ions	Peptide
909.3941	908.3868	908.3480	42.6	100	106	0	---	R.AVCECDR.V
1305.5904	1304.5831	1304.6145	-24.02	123	132	0	---	K.YMLYSLLDCK.E
1732.8009	1731.7936	1731.7596	19.6	107	121	0	87	R.VAAICFGENMNTYDK.K
1748.7978	1747.7906	1747.7545	20.6	107	121	0	(55)	R.VAAICFGENMNTYDK.K + Oxidation (M)
1876.8772	1875.8699	1875.8495	10.9	107	122	1	---	R.VAAICFGENMNTYDKK.Y + Oxidation (M)
2625.1754	2624.1681	2624.1159	19.9	77	99	0	---	K.LSTYSYSFENGDIVCGGDDPCLR.A

No match to: 817.1395, 837.1000, 839.1118, 852.3774, 855.0882, 859.0799, 861.0964, 868.5738, 871.0597, 874.0428, 877.0680, 882.6029, 903.4152, 905.5111, 987.5256, 1022.1471, 1044.1224, 1064.0902, 1066.1043, 1082.0794, 1149.5023, 1157.6635, 1175.6978, 1201.6609, 1222.6157, 1249.1322, 1252.6490, 1267.6375, 1271.1084, 1283.6283, 1289.6181, 1293.6475, 1309.6407, 1320.6258, 1323.6473, 1339.6437, 1345.6280, 1355.6382, 1361.6097, 1365.6708, 1377.5960, 1393.5844, 1395.7311, 1411.7256, 1436.7603, 1475.7776, 1476.1123, 1557.8703, 1636.7539, 1707.8181, 1718.7880, 1734.7794, 1740.7760, 1744.8013, 1754.7913, 1756.7547, 1770.7537, 1772.7239, 1838.9638, 1851.8058, 1862.8756, 1921.8557, 1924.8861, 1941.8985, 1948.8578, 1967.8661, 2005.8049, 2118.8044, 2170.8299, 2217.9647, 2234.8584, 2255.8811, 2265.2040, 2277.1368, 2566.1425, 2618.1737, 2636.1875, 2663.1955, 2682.2062, 2695.2126, 2708.2152, 2720.1459, 2724.2164, 3347.7264

35. Q7T1D2_VIPAZ **Mass:** 16258 **Score:** 123 **Expect:** 7.1e-010 **Matches:** 6

Ammodytin I1 OS=Vipera aspis zinnikeri OX=55427 GN=AmtI1 PE=2 SV=1

Observed	Mr(expt)	Mr(calc)	ppm	Start	End	Miss	Ions	Peptide
909.3941	908.3868	908.3480	42.6	100	106	0	---	R.AVCECDR.V
1305.5904	1304.5831	1304.6145	-24.02	123	132	0	---	K.YMLYSLLDCK.E
1732.8009	1731.7936	1731.7596	19.6	107	121	0	87	R.VAAICFGENMNTYDK.K
1748.7978	1747.7906	1747.7545	20.6	107	121	0	(55)	R.VAAICFGENMNTYDK.K + Oxidation (M)
1876.8772	1875.8699	1875.8495	10.9	107	122	1	---	R.VAAICFGENMNTYDKK.Y + Oxidation (M)
2625.1754	2624.1681	2624.1159	19.9	77	99	0	---	K.LSTYSYSFENGDIVCGGDDPCLR.A

No match to: 817.1395, 837.1000, 839.1118, 852.3774, 855.0882, 859.0799, 861.0964, 868.5738, 871.0597, 874.0428, 877.0680, 882.6029, 903.4152, 905.5111, 987.5256, 1022.1471, 1044.1224, 1064.0902, 1066.1043, 1082.0794, 1149.5023, 1157.6635, 1175.6978, 1201.6609, 1222.6157, 1249.1322, 1252.6490, 1267.6375, 1271.1084, 1283.6283, 1289.6181, 1293.6475, 1309.6407, 1320.6258, 1323.6473, 1339.6437, 1345.6280, 1355.6382, 1361.6097, 1365.6708, 1377.5960, 1393.5844, 1395.7311, 1411.7256, 1436.7603, 1475.7776, 1476.1123, 1557.8703, 1636.7539, 1707.8181, 1718.7880, 1734.7794, 1740.7760, 1744.8013, 1754.7913, 1756.7547, 1770.7537, 1772.7239, 1838.9638, 1851.8058, 1862.8756, 1921.8557, 1924.8861, 1941.8985, 1948.8578, 1967.8661, 2005.8049, 2118.8044, 2170.8299, 2217.9647, 2234.8584, 2255.8811, 2265.2040, 2277.1368, 2566.1425, 2618.1737, 2636.1875, 2663.1955, 2682.2062, 2695.2126, 2708.2152, 2720.1459, 2724.2164, 3347.7264

36. Q6A3J4_VIPBB **Mass:** 16258 **Score:** 123 **Expect:** 7.1e-010 **Matches:** 6

Ammodytin I1(C) isoform OS=Vipera berus berus OX=31156 PE=2 SV=1

Observed	Mr(expt)	Mr(calc)	ppm	Start	End	Miss	Ions	Peptide
909.3941	908.3868	908.3480	42.6	100	106	0	---	R.AVCECDR.V
1305.5904	1304.5831	1304.6145	-24.02	123	132	0	---	K.YMLYSLLDCK.E
1732.8009	1731.7936	1731.7596	19.6	107	121	0	87	R.VAAICFGENMNTYDK.K
1748.7978	1747.7906	1747.7545	20.6	107	121	0	(55)	R.VAAICFGENMNTYDK.K + Oxidation (M)
1876.8772	1875.8699	1875.8495	10.9	107	122	1	---	R.VAAICFGENMNTYDKK.Y + Oxidation (M)
2625.1754	2624.1681	2624.1159	19.9	77	99	0	---	K.LSTYSYSFENGDIVCGGDDPCLR.A

No match to: 817.1395, 837.1000, 839.1118, 852.3774, 855.0882, 859.0799, 861.0964, 868.5738, 871.0597, 874.0428, 877.0680, 882.6029, 903.4152, 905.5111, 987.5256, 1022.1471, 1044.1224, 1064.0902, 1066.1043, 1082.0794, 1149.5023, 1157.6635, 1175.6978, 1201.6609, 1222.6157, 1249.1322, 1252.6490, 1267.6375, 1271.1084, 1283.6283, 1289.6181, 1293.6475, 1309.6407, 1320.6258, 1323.6473, 1339.6437, 1345.6280, 1355.6382, 1361.6097, 1365.6708, 1377.5960, 1393.5844, 1395.7311, 1411.7256, 1436.7603, 1475.7776, 1476.1123, 1557.8703, 1636.7539, 1707.8181, 1718.7880, 1734.7794, 1740.7760, 1744.8013, 1754.7913, 1756.7547, 1770.7537, 1772.7239, 1838.9638, 1851.8058, 1862.8756, 1921.8557, 1924.8861, 1941.8985, 1948.8578, 1967.8661, 2005.8049, 2118.8044, 2170.8299, 2217.9647, 2234.8584, 2255.8811, 2265.2040, 2277.1368, 2566.1425, 2618.1737, 2636.1875, 2663.1955, 2682.2062, 2695.2126, 2708.2152, 2720.1459, 2724.2164, 3347.7264

37. Q6A3J6_VIPAZ **Mass:** 16330 **Score:** 123 **Expect:** 7.1e-010 **Matches:** 6

Ammodytin I1(C) isoform OS=Vipera aspis zinnikeri OX=55427 PE=2 SV=1

Observed	Mr(expt)	Mr(calc)	ppm	Start	End	Miss	Ions	Peptide
909.3941	908.3868	908.3480	42.6	100	106	0	---	R.AVCECDR.V
1305.5904	1304.5831	1304.6145	-24.02	123	132	0	---	K.YMLYSLLDCK.E
1732.8009	1731.7936	1731.7596	19.6	107	121	0	87	R.VAAICFGENMNTYDK.K
1748.7978	1747.7906	1747.7545	20.6	107	121	0	(55)	R.VAAICFGENMNTYDK.K + Oxidation (M)
1876.8772	1875.8699	1875.8495	10.9	107	122	1	---	R.VAAICFGENMNTYDKK.Y + Oxidation (M)
2625.1754	2624.1681	2624.1159	19.9	77	99	0	---	K.LSTYSYSFENGDIVCGGDDPCLR.A

No match to: 817.1395, 837.1000, 839.1118, 852.3774, 855.0882, 859.0799, 861.0964, 868.5738, 871.0597, 874.0428, 877.0680, 882.6029, 903.4152, 905.5111, 987.5256, 1022.1471, 1044.1224, 1064.0902, 1066.1043, 1082.0794, 1149.5023, 1157.6635, 1175.6978, 1201.6609, 1222.6157, 1249.1322, 1252.6490, 1267.6375, 1271.1084, 1283.6283, 1289.6181, 1293.6475, 1309.6407, 1320.6258, 1323.6473, 1339.6437, 1345.6280, 1355.6382, 1361.6097, 1365.6708, 1377.5960, 1393.5844, 1395.7311, 1411.7256, 1436.7603, 1475.7776, 1476.1123, 1557.8703, 1636.7539, 1707.8181, 1718.7880, 1734.7794, 1740.7760, 1744.8013, 1754.7913, 1756.7547, 1770.7537, 1772.7239, 1838.9638, 1851.8058, 1862.8756, 1921.8557, 1924.8861, 1941.8985, 1948.8578, 1967.8661, 2005.8049, 2118.8044, 2170.8299, 2217.9647, 2234.8584, 2255.8811, 2265.2040, 2277.1368, 2566.1425, 2618.1737, 2636.1875, 2663.1955, 2682.2062, 2695.2126, 2708.2152, 2720.1459, 2724.2164, 3347.7264

38. Q6A3I3_VIPAP **Mass:** 16286 **Score:** 123 **Expect:** 7.1e-010 **Matches:** 6

Ammodytin I1(C) isoform OS=Vipera aspis aspis OX=194601 PE=2 SV=1

Observed	Mr(expt)	Mr(calc)	ppm	Start	End	Miss	Ions	Peptide
909.3941	908.3868	908.3480	42.6	100	106	0	---	R.AVCECDR.V
1305.5904	1304.5831	1304.6145	-24.02	123	132	0	---	K.YMLYSLLDCK.E

Observed	Mr(expt)	Mr(calc)	ppm	Start	End	Miss	Ions	Peptide
1732.8009	1731.7936	1731.7596	19.6	107	121	0	87	R.VAAICFGENMNTYDK.K
1748.7978	1747.7906	1747.7545	20.6	107	121	0	(55)	R.VAAICFGENMNTYDK.K + Oxidation (M)
1876.8772	1875.8699	1875.8495	10.9	107	122	1	---	R.VAAICFGENMNTYDKK.Y + Oxidation (M)
2625.1754	2624.1681	2624.1651	1.15	28	49	1	---	K.KTGIFGIMSYIYYGCYCGWGGR.G + Oxidation (M)

No match to: 817.1395, 837.1000, 839.1118, 852.3774, 855.0882, 859.0799, 861.0964, 868.5738, 871.0597, 874.0428, 877.0680, 882.6029, 903.4152, 905.5111, 987.5256, 1022.1471, 1044.1224, 1064.0902, 1066.1043, 1082.0794, 1149.5023, 1157.6635, 1175.6978, 1201.6609, 1222.6157, 1249.1322, 1252.6490, 1267.6375, 1271.1084, 1283.6283, 1289.6181, 1293.6475, 1309.6407, 1320.6258, 1323.6473, 1339.6437, 1345.6280, 1355.6382, 1361.6097, 1365.6708, 1377.5960, 1393.5844, 1395.7311, 1411.7256, 1436.7603, 1475.7776, 1476.1123, 1557.8703, 1636.7539, 1707.8181, 1718.7880, 1734.7794, 1740.7760, 1744.8013, 1754.7913, 1756.7547, 1770.7537, 1772.7239, 1838.9638, 1851.8058, 1862.8756, 1921.8557, 1924.8861, 1941.8985, 1948.8578, 1967.8661, 2005.8049, 2118.8044, 2170.8299, 2217.9647, 2234.8584, 2255.8811, 2265.2040, 2277.1368, 2566.1425, 2618.1737, 2636.1875, 2663.1955, 2682.2062, 2695.2126, 2708.2152, 2720.1459, 2724.2164, 3347.7264

39. Q6A3F7_VIPAP **Mass:** 16310 **Score:** 122 **Expect:** 8.9e-010 **Matches:** 6
Ammodytin I1(C) variant OS=Vipera aspis aspis OX=194601 PE=2 SV=1

Observed	Mr(expt)	Mr(calc)	ppm	Start	End	Miss	Ions	Peptide
909.3941	908.3868	908.3480	42.6	100	106	0	---	R.AVCECDR.V
1305.5904	1304.5831	1304.6145	-24.02	123	132	0	---	K.YMLYSLLDCK.E
1732.8009	1731.7936	1731.7596	19.6	107	121	0	87	R.VAAICFGENMNTYDK.K
1748.7978	1747.7906	1747.7545	20.6	107	121	0	(55)	R.VAAICFGENMNTYDK.K + Oxidation (M)
1876.8772	1875.8699	1875.8495	10.9	107	122	1	---	R.VAAICFGENMNTYDKK.Y + Oxidation (M)
2625.1754	2624.1681	2624.1159	19.9	77	99	0	---	K.LSTYSYSFENGDIVCGGDDPCLR.A

No match to: 817.1395, 837.1000, 839.1118, 852.3774, 855.0882, 859.0799, 861.0964, 868.5738, 871.0597, 874.0428, 877.0680, 882.6029, 903.4152, 905.5111, 987.5256, 1022.1471, 1044.1224, 1064.0902, 1066.1043, 1082.0794, 1149.5023, 1157.6635, 1175.6978, 1201.6609, 1222.6157, 1249.1322, 1252.6490, 1267.6375, 1271.1084, 1283.6283, 1289.6181, 1293.6475, 1309.6407, 1320.6258, 1323.6473, 1339.6437, 1345.6280, 1355.6382, 1361.6097, 1365.6708, 1377.5960, 1393.5844, 1395.7311, 1411.7256, 1436.7603, 1475.7776, 1476.1123, 1557.8703, 1636.7539, 1707.8181, 1718.7880, 1734.7794, 1740.7760, 1744.8013, 1754.7913, 1756.7547, 1770.7537, 1772.7239, 1838.9638, 1851.8058, 1862.8756, 1921.8557, 1924.8861, 1941.8985, 1948.8578, 1967.8661, 2005.8049, 2118.8044, 2170.8299, 2217.9647, 2234.8584, 2255.8811, 2265.2040, 2277.1368, 2566.1425, 2618.1737, 2636.1875, 2663.1955, 2682.2062, 2695.2126, 2708.2152, 2720.1459, 2724.2164, 3347.7264

40. Q6A3F6_VIPAP **Mass:** 16200 **Score:** 114 **Expect:** 5.6e-009 **Matches:** 5
Ammodytin I1 (C) variant OS=Vipera aspis aspis OX=194601 PE=2 SV=1

Observed	Mr(expt)	Mr(calc)	ppm	Start	End	Miss	Ions	Peptide
1305.5904	1304.5831	1304.6145	-24.02	123	132	0	---	K.YMLYSLLDCK.E
1732.8009	1731.7936	1731.7596	19.6	107	121	0	87	R.VAAICFGENMNTYDK.K
1748.7978	1747.7906	1747.7545	20.6	107	121	0	(55)	R.VAAICFGENMNTYDK.K + Oxidation (M)
1876.8772	1875.8699	1875.8495	10.9	107	122	1	---	R.VAAICFGENMNTYDKK.Y + Oxidation (M)
2625.1754	2624.1681	2624.1159	19.9	77	99	0	---	K.LSTYSYSFENGDIVCGGDDPCLR.A

No match to: 817.1395, 837.1000, 839.1118, 852.3774, 855.0882, 859.0799, 861.0964, 868.5738, 871.0597, 874.0428, 877.0680, 882.6029, 903.4152, 905.5111, 909.3941, 987.5256, 1022.1471, 1044.1224, 1064.0902, 1066.1043, 1082.0794, 1149.5023, 1157.6635, 1175.6978, 1201.6609, 1222.6157, 1249.1322, 1252.6490, 1267.6375, 1271.1084, 1283.6283, 1289.6181, 1293.6475, 1309.6407, 1320.6258, 1323.6473, 1339.6437, 1345.6280, 1355.6382, 1361.6097, 1365.6708, 1377.5960, 1393.5844, 1395.7311, 1411.7256, 1436.7603, 1475.7776, 1476.1123, 1557.8703, 1636.7539, 1707.8181, 1718.7880, 1734.7794, 1740.7760, 1744.8013, 1754.7913, 1756.7547, 1770.7537, 1772.7239, 1838.9638, 1851.8058, 1862.8756, 1921.8557, 1924.8861, 1941.8985, 1948.8578, 1967.8661, 2005.8049, 2118.8044, 2170.8299, 2217.9647, 2234.8584, 2255.8811, 2265.2040, 2277.1368, 2566.1425, 2618.1737, 2636.1875, 2663.1955, 2682.2062, 2695.2126, 2708.2152, 2720.1459, 2724.2164, 3347.7264

41. Q6A3I9_VIPBB **Mass:** 16187 **Score:** 114 **Expect:** 5.6e-009 **Matches:** 5
Ammodytin I1(C) variant OS=Vipera berus berus OX=31156 PE=2 SV=1

Observed	Mr(expt)	Mr(calc)	ppm	Start	End	Miss	Ions	Peptide
909.3941	908.3868	908.3480	42.6	100	106	0	---	R.AVCECDR.V
1732.8009	1731.7936	1731.7596	19.6	107	121	0	87	R.VAAICFGENMNTYDK.K
1748.7978	1747.7906	1747.7545	20.6	107	121	0	(55)	R.VAAICFGENMNTYDK.K + Oxidation (M)
1876.8772	1875.8699	1875.8495	10.9	107	122	1	---	R.VAAICFGENMNTYDKK.Y + Oxidation (M)
2625.1754	2624.1681	2624.1159	19.9	77	99	0	---	K.LSTYSYSFENGDIVCGGDDPCLR.A

No match to: 817.1395, 837.1000, 839.1118, 852.3774, 855.0882, 859.0799, 861.0964, 868.5738, 871.0597, 874.0428, 877.0680, 882.6029, 903.4152, 905.5111, 987.5256, 1022.1471, 1044.1224, 1064.0902, 1066.1043, 1082.0794, 1149.5023, 1157.6635, 1175.6978, 1201.6609, 1222.6157, 1249.1322, 1252.6490, 1267.6375, 1271.1084, 1283.6283, 1289.6181, 1293.6475, 1305.5904, 1309.6407, 1320.6258, 1323.6473, 1339.6437, 1345.6280, 1355.6382, 1361.6097, 1365.6708, 1377.5960, 1393.5844, 1395.7311, 1411.7256, 1436.7603, 1475.7776, 1476.1123, 1557.8703, 1636.7539, 1707.8181, 1718.7880, 1734.7794, 1740.7760, 1744.8013, 1754.7913, 1756.7547, 1770.7537, 1772.7239, 1838.9638, 1851.8058, 1862.8756, 1921.8557, 1924.8861, 1941.8985, 1948.8578, 1967.8661, 2005.8049, 2118.8044, 2170.8299, 2217.9647, 2234.8584, 2255.8811, 2265.2040, 2277.1368, 2566.1425, 2618.1737, 2636.1875, 2663.1955, 2682.2062, 2695.2126, 2708.2152, 2720.1459, 2724.2164, 3347.7264

42. Q6A3H5_VIPAS **Mass:** 16245 **Score:** 114 **Expect:** 5.6e-009 **Matches:** 5
Ammodytin I1(F) variant OS=Vipera aspis atra OX=246182 PE=2 SV=1

Observed	Mr(expt)	Mr(calc)	ppm	Start	End	Miss	Ions	Peptide
909.3941	908.3868	908.3480	42.6	100	106	0	---	R.AVCECDR.V
1732.8009	1731.7936	1731.7596	19.6	107	121	0	87	R.VAAICFGENMNTYDK.K
1748.7978	1747.7906	1747.7545	20.6	107	121	0	(55)	R.VAAICFGENMNTYDK.K + Oxidation (M)
1876.8772	1875.8699	1875.8495	10.9	107	122	1	---	R.VAAICFGENMNTYDKK.Y + Oxidation (M)
2625.1754	2624.1681	2624.1159	19.9	77	99	0	---	K.LSTYSYSFENGDIVCGGDDPCLR.A

No match to: 817.1395, 837.1000, 839.1118, 852.3774, 855.0882, 859.0799, 861.0964, 868.5738, 871.0597, 874.0428, 877.0680, 882.6029, 903.4152, 905.5111, 987.5256, 1022.1471, 1044.1224, 1064.0902, 1066.1043, 1082.0794, 1149.5023, 1157.6635, 1175.6978, 1201.6609, 1222.6157, 1249.1322, 1252.6490, 1267.6375, 1271.1084, 1283.6283, 1289.6181, 1293.6475, 1305.5904, 1309.6407, 1320.6258, 1323.6473, 1339.6437, 1345.6280, 1355.6382, 1361.6097, 1365.6708, 1377.5960, 1393.5844, 1395.7311, 1411.7256, 1436.7603, 1475.7776, 1476.1123, 1557.8703, 1636.7539, 1707.8181, 1718.7880, 1734.7794, 1740.7760, 1744.8013, 1754.7913, 1756.7547, 1770.7537, 1772.7239, 1838.9638, 1851.8058, 1862.8756, 1921.8557, 1924.8861, 1941.8985, 1948.8578, 1967.8661, 2005.8049, 2118.8044, 2170.8299, 2217.9647, 2234.8584, 2255.8811, 2265.2040, 2277.1368, 2566.1425, 2618.1737, 2636.1875, 2663.1955, 2682.2062, 2695.2126, 2708.2152, 2720.1459, 2724.2164, 3347.7264

43. Q6A3I6_VIPAP **Mass:** 16257 **Score:** 113 **Expect:** 7.1e-009 **Matches:** 5
Ammodytin I1(C) variant OS=Vipera aspis aspis OX=194601 PE=2 SV=1

Observed	Mr(expt)	Mr(calc)	ppm	Start	End	Miss	Ions	Peptide
1305.5904	1304.5831	1304.6145	-24.02	123	132	0	---	K.YMLYSLLDCK.E
1732.8009	1731.7936	1731.7596	19.6	107	121	0	87	R.VAAICFGENMNTYDK.K
1748.7978	1747.7906	1747.7545	20.6	107	121	0	(55)	R.VAAICFGENMNTYDK.K + Oxidation (M)
1876.8772	1875.8699	1875.8495	10.9	107	122	1	---	R.VAAICFGENMNTYDKK.Y + Oxidation (M)
2625.1754	2624.1681	2624.1159	19.9	77	99	0	---	K.LSTYSYSFENGDIVCGGDDPCLR.A

No match to: 817.1395, 837.1000, 839.1118, 852.3774, 855.0882, 859.0799, 861.0964, 868.5738, 871.0597, 874.0428, 877.0680, 882.6029, 903.4152, 905.5111, 909.3941, 987.5256, 1022.1471, 1044.1224, 1064.0902, 1066.1043, 1082.0794, 1149.5023, 1157.6635, 1175.6978, 1201.6609, 1222.6157, 1249.1322, 1252.6490, 1267.6375, 1271.1084, 1283.6283, 1289.6181, 1293.6475, 1309.6407, 1320.6258, 1323.6473, 1339.6437, 1345.6280, 1355.6382, 1361.6097, 1365.6708, 1377.5960, 1393.5844, 1395.7311, 1411.7256, 1436.7603, 1475.7776, 1476.1123, 1557.8703, 1636.7539, 1707.8181, 1718.7880, 1734.7794, 1740.7760, 1744.8013, 1754.7913, 1756.7547, 1770.7537, 1772.7239, 1838.9638, 1851.8058, 1862.8756, 1921.8557, 1924.8861, 1941.8985, 1948.8578, 1967.8661, 2005.8049, 2118.8044, 2170.8299, 2217.9647, 2234.8584, 2255.8811, 2265.2040, 2277.1368, 2566.1425, 2618.1737, 2636.1875, 2663.1955, 2682.2062, 2695.2126, 2708.2152, 2720.1459, 2724.2164, 3347.7264

44. Q6A3I4_VIPAP **Mass:** 16272 **Score:** 112 **Expect:** 8.9e-009 **Matches:** 5
Ammodytin I1(C) variant OS=Vipera aspis aspis OX=194601 PE=2 SV=1

Observed	Mr(expt)	Mr(calc)	ppm	Start	End	Miss	Ions	Peptide
909.3941	908.3868	908.3480	42.6	100	106	0	---	R.AVCECDR.V
1305.5904	1304.5831	1304.6145	-24.02	123	132	0	---	K.YMLYSLLDCK.E
1732.8009	1731.7936	1731.7596	19.6	107	121	0	87	R.VAAICFGENMNTYDK.K
1748.7978	1747.7906	1747.7545	20.6	107	121	0	(55)	R.VAAICFGENMNTYDK.K + Oxidation (M)
1876.8772	1875.8699	1875.8495	10.9	107	122	1	---	R.VAAICFGENMNTYDKK.Y + Oxidation (M)

No match to: 817.1395, 837.1000, 839.1118, 852.3774, 855.0882, 859.0799, 861.0964, 868.5738, 871.0597, 874.0428, 877.0680, 882.6029, 903.4152, 905.5111, 987.5256, 1022.1471, 1044.1224, 1064.0902, 1066.1043, 1082.0794, 1149.5023, 1157.6635, 1175.6978, 1201.6609, 1222.6157, 1249.1322, 1252.6490, 1267.6375, 1271.1084, 1283.6283, 1289.6181, 1293.6475, 1309.6407, 1320.6258, 1323.6473, 1339.6437, 1345.6280, 1355.6382, 1361.6097, 1365.6708, 1377.5960, 1393.5844, 1395.7311, 1411.7256, 1436.7603, 1475.7776, 1476.1123, 1557.8703, 1636.7539, 1707.8181, 1718.7880, 1740.7760, 1744.8013, 1754.7913, 1756.7547, 1770.7537, 1772.7239, 1838.9638, 1851.8058, 1862.8756, 1921.8557, 1924.8861, 1941.8985, 1948.8578, 1967.8661, 2005.8049, 2118.8044, 2170.8299, 2217.9647, 2234.8584, 2255.8811, 2265.2040, 2277.1368, 2566.1425, 2618.1737, 2625.1754, 2636.1875, 2663.1955, 2682.2062, 2695.2126, 2708.2152, 2720.1459, 2724.2164, 3347.7264

45. Q6A3K9_VIPAP **Mass:** 16202 **Score:** 112 **Expect:** 8.9e-009 **Matches:** 5
Ammodytin I1(E) variant OS=Vipera aspis aspis OX=194601 PE=2 SV=1

Observed	Mr(expt)	Mr(calc)	ppm	Start	End	Miss	Ions	Peptide
909.3941	908.3868	908.3480	42.6	100	106	0	---	R.AVCECDR.V
1305.5904	1304.5831	1304.6145	-24.02	123	132	0	---	K.YMLYSLLDCK.E
1732.8009	1731.7936	1731.7596	19.6	107	121	0	87	R.VAAICFGENMNTYDK.K
1748.7978	1747.7906	1747.7545	20.6	107	121	0	(55)	R.VAAICFGENMNTYDK.K + Oxidation (M)
1876.8772	1875.8699	1875.8495	10.9	107	122	1	---	R.VAAICFGENMNTYDKK.Y + Oxidation (M)

No match to: 817.1395, 837.1000, 839.1118, 852.3774, 855.0882, 859.0799, 861.0964, 868.5738, 871.0597, 874.0428, 877.0680, 882.6029, 903.4152, 905.5111, 987.5256, 1022.1471, 1044.1224, 1064.0902, 1066.1043, 1082.0794, 1149.5023, 1157.6635, 1175.6978, 1201.6609, 1222.6157, 1249.1322, 1252.6490, 1267.6375, 1271.1084, 1283.6283, 1289.6181, 1293.6475, 1309.6407, 1320.6258, 1323.6473, 1339.6437, 1345.6280, 1355.6382, 1361.6097, 1365.6708, 1377.5960, 1393.5844, 1395.7311, 1411.7256, 1436.7603, 1475.7776, 1476.1123, 1557.8703, 1636.7539, 1707.8181, 1718.7880, 1734.7794, 1740.7760, 1744.8013, 1754.7913, 1756.7547, 1770.7537, 1772.7239, 1838.9638, 1851.8058, 1862.8756, 1921.8557, 1924.8861, 1941.8985, 1948.8578, 1967.8661, 2005.8049, 2118.8044, 2170.8299, 2217.9647, 2234.8584, 2255.8811, 2265.2040, 2277.1368, 2566.1425, 2618.1737, 2625.1754, 2636.1875, 2663.1955, 2682.2062, 2695.2126, 2708.2152, 2720.1459, 2724.2164, 3347.7264

46. Q6A3L1_VIPAP **Mass:** 16272 **Score:** 112 **Expect:** 8.9e-009 **Matches:** 5
Ammodytin I1(E) isoform OS=Vipera aspis aspis OX=194601 PE=2 SV=1

Observed	Mr(expt)	Mr(calc)	ppm	Start	End	Miss	Ions	Peptide
909.3941	908.3868	908.3480	42.6	100	106	0	---	R.AVCECDR.V
1305.5904	1304.5831	1304.6145	-24.02	123	132	0	---	K.YMLYSLLDCK.E
1732.8009	1731.7936	1731.7596	19.6	107	121	0	87	R.VAAICFGENMNTYDK.K
1748.7978	1747.7906	1747.7545	20.6	107	121	0	(55)	R.VAAICFGENMNTYDK.K + Oxidation (M)
1876.8772	1875.8699	1875.8495	10.9	107	122	1	---	R.VAAICFGENMNTYDKK.Y + Oxidation (M)

No match to: 817.1395, 837.1000, 839.1118, 852.3774, 855.0882, 859.0799, 861.0964, 868.5738, 871.0597, 874.0428, 877.0680, 882.6029, 903.4152, 905.5111, 987.5256, 1022.1471, 1044.1224, 1064.0902, 1066.1043, 1082.0794, 1149.5023, 1157.6635, 1175.6978, 1201.6609, 1222.6157, 1249.1322, 1252.6490, 1267.6375, 1271.1084, 1283.6283, 1289.6181, 1293.6475, 1309.6407, 1320.6258, 1323.6473, 1339.6437, 1345.6280, 1355.6382, 1361.6097, 1365.6708, 1377.5960, 1393.5844, 1395.7311, 1411.7256, 1436.7603, 1475.7776, 1476.1123, 1557.8703, 1636.7539, 1707.8181, 1718.7880, 1734.7794, 1740.7760, 1744.8013, 1754.7913, 1756.7547, 1770.7537, 1772.7239, 1838.9638, 1851.8058, 1862.8756, 1921.8557, 1924.8861, 1941.8985, 1948.8578, 1967.8661, 2005.8049, 2118.8044, 2170.8299, 2217.9647, 2234.8584, 2255.8811, 2265.2040, 2277.1368, 2566.1425, 2618.1737, 2625.1754, 2636.1875, 2663.1955, 2682.2062, 2695.2126, 2708.2152, 2720.1459, 2724.2164, 3347.7264

47. Q6A3L0_VIPAP **Mass:** 16360 **Score:** 112 **Expect:** 8.9e-009 **Matches:** 5
Ammodytin I1(E) variant OS=Vipera aspis aspis OX=194601 PE=2 SV=1

Observed	Mr(expt)	Mr(calc)	ppm	Start	End	Miss	Ions	Peptide
909.3941	908.3868	908.3480	42.6	100	106	0	---	R.AVCECDR.V
1305.5904	1304.5831	1304.6145	-24.02	123	132	0	---	K.YMLYSLLDCK.E
1732.8009	1731.7936	1731.7596	19.6	107	121	0	87	R.VAAICFGENMNTYDK.K
1748.7978	1747.7906	1747.7545	20.6	107	121	0	(55)	R.VAAICFGENMNTYDK.K + Oxidation (M)
1876.8772	1875.8699	1875.8495	10.9	107	122	1	---	R.VAAICFGENMNTYDKK.Y + Oxidation (M)

No match to: 817.1395, 837.1000, 839.1118, 852.3774, 855.0882, 859.0799, 861.0964, 868.5738, 871.0597, 874.0428, 877.0680, 882.6029, 903.4152, 905.5111, 987.5256, 1022.1471, 1044.1224, 1064.0902, 1066.1043, 1082.0794, 1149.5023, 1157.6635, 1175.6978, 1201.6609, 1222.6157, 1249.1322, 1252.6490, 1267.6375, 1271.1084, 1283.6283, 1289.6181, 1293.6475, 1309.6407, 1320.6258, 1323.6473, 1339.6437, 1345.6280, 1355.6382, 1361.6097, 1365.6708, 1377.5960, 1393.5844, 1395.7311, 1411.7256, 1436.7603, 1475.7776, 1476.1123, 1557.8703, 1636.7539, 1707.8181, 1718.7880, 1734.7794, 1740.7760, 1744.8013, 1754.7913, 1756.7547, 1770.7537, 1772.7239, 1838.9638, 1851.8058, 1862.8756, 1921.8557, 1924.8861, 1941.8985, 1948.8578, 1967.8661, 2005.8049, 2118.8044, 2170.8299, 2217.9647, 2234.8584, 2255.8811, 2265.2040, 2277.1368, 2566.1425, 2618.1737, 2625.1754, 2636.1875, 2663.1955, 2682.2062, 2695.2126, 2708.2152, 2720.1459, 2724.2164, 3347.7264

48. Q6A3H2_VIPAS **Mass:** 16198 **Score:** 112 **Expect:** 8.9e-009 **Matches:** 5
Ammodytin I1(C) variant OS=Vipera aspis atra OX=246182 PE=2 SV=1

Observed	Mr(expt)	Mr(calc)	ppm	Start	End	Miss	Ions	Peptide
909.3941	908.3868	908.3480	42.6	100	106	0	---	R.AVCECDR.V
1305.5904	1304.5831	1304.6145	-24.02	123	132	0	---	K.YMLYSLLDCK.E
1732.8009	1731.7936	1731.7596	19.6	107	121	0	87	R.VAAICFGENMNTYDK.K
1748.7978	1747.7906	1747.7545	20.6	107	121	0	(55)	R.VAAICFGENMNTYDK.K + Oxidation (M)
1876.8772	1875.8699	1875.8495	10.9	107	122	1	---	R.VAAICFGENMNTYDKK.Y + Oxidation (M)

No match to: 817.1395, 837.1000, 839.1118, 852.3774, 855.0882, 859.0799, 861.0964, 868.5738, 871.0597, 874.0428, 877.0680, 882.6029, 903.4152, 905.5111, 987.5256, 1022.1471, 1044.1224, 1064.0902, 1066.1043, 1082.0794, 1149.5023, 1157.6635, 1175.6978, 1201.6609, 1222.6157, 1249.1322, 1252.6490, 1267.6375, 1271.1084, 1283.6283, 1289.6181, 1293.6475, 1309.6407, 1320.6258, 1323.6473, 1339.6437, 1345.6280, 1355.6382, 1361.6097, 1365.6708, 1377.5960, 1393.5844, 1395.7311, 1411.7256, 1436.7603, 1475.7776, 1476.1123, 1557.8703, 1636.7539, 1707.8181, 1718.7880, 1734.7794, 1740.7760, 1744.8013, 1754.7913, 1756.7547, 1770.7537, 1772.7239, 1838.9638, 1851.8058, 1862.8756, 1921.8557, 1924.8861, 1941.8985, 1948.8578, 1967.8661, 2005.8049, 2118.8044, 2170.8299, 2217.9647, 2234.8584, 2255.8811, 2265.2040, 2277.1368, 2566.1425, 2618.1737, 2625.1754, 2636.1875, 2663.1955, 2682.2062, 2695.2126, 2708.2152, 2720.1459, 2724.2164, 3347.7264

49. Q6A3M4_VIPAP **Mass:** 16217 **Score:** 104 **Expect:** 5.6e-008 **Matches:** 4
Ammodytin I1(C) variant OS=Vipera aspis aspis OX=194601 PE=2 SV=1

Observed	Mr(expt)	Mr(calc)	ppm	Start	End	Miss	Ions	Peptide
909.3941	908.3868	908.3480	42.6	100	106	0	---	R.AVCECDR.V
1732.8009	1731.7936	1731.7596	19.6	107	121	0	87	R.VAAICFGENMNTYDK.K
1748.7978	1747.7906	1747.7545	20.6	107	121	0	(55)	R.VAAICFGENMNTYDK.K + Oxidation (M)
1876.8772	1875.8699	1875.8495	10.9	107	122	1	---	R.VAAICFGENMNTYDKK.Y + Oxidation (M)

No match to: 817.1395, 837.1000, 839.1118, 852.3774, 855.0882, 859.0799, 861.0964, 868.5738, 871.0597, 874.0428, 877.0680, 882.6029, 903.4152, 905.5111, 987.5256, 1022.1471, 1044.1224, 1064.0902, 1066.1043, 1082.0794, 1149.5023, 1157.6635, 1175.6978, 1201.6609, 1222.6157, 1249.1322, 1252.6490, 1267.6375, 1271.1084, 1283.6283, 1289.6181, 1293.6475, 1305.5904, 1309.6407, 1320.6258, 1323.6473, 1339.6437, 1345.6280, 1355.6382, 1361.6097, 1365.6708, 1377.5960, 1393.5844, 1395.7311, 1411.7256, 1436.7603, 1475.7776, 1476.1123, 1557.8703, 1636.7539, 1707.8181, 1718.7880, 1734.7794, 1740.7760, 1744.8013, 1754.7913, 1756.7547, 1770.7537, 1772.7239, 1838.9638,

1851.8058, 1862.8756, 1921.8557, 1924.8861, 1941.8985, 1948.8578, 1967.8661, 2005.8049, 2118.8044, 2170.8299, 2217.9647, 2234.8584, 2255.8811, 2265.2040, 2277.1368, 2566.1425, 2618.1737, 2625.1754, 2636.1875, 2663.1955, 2682.2062, 2695.2126, 2708.2152, 2720.1459, 2724.2164, 3347.7264

50. PA2A2_DABRR **Mass:** 16430 **Score:** 75 **Expect:** 4.3e-005 **Matches:** 4

Acidic phospholipase A2 Drk-a2 OS=Daboia russelii OX=8707 PE=2 SV=1

Observed	Mr(expt)	Mr(calc)	ppm	Start	End	Miss	Ions	Peptide
909.3941	908.3868	908.3480	42.6	100 -	106	0	---	R.AVCECDR.V
987.5256	986.5184	986.4781	40.8	50 -	58	0	3	K.GKPQDATDR.C
1339.6437	1338.6364	1338.5988	28.1	123 -	132	0	56	K.YMLYSIFDCK.E
1355.6382	1354.6310	1354.5937	27.5	123 -	132	0	(11)	K.YMLYSIFDCK.E + Oxidation (M)

No match to: 817.1395, 837.1000, 839.1118, 852.3774, 855.0882, 859.0799, 861.0964, 868.5738, 871.0597, 874.0428, 877.0680, 882.6029, 903.4152, 905.5111, 1022.1471, 1044.1224, 1064.0902, 1066.1043, 1082.0794, 1149.5023, 1157.6635, 1175.6978, 1201.6609, 1222.6157, 1249.1322, 1252.6490, 1267.6375, 1271.1084, 1283.6283, 1289.6181, 1293.6475, 1305.5904, 1309.6407, 1320.6258, 1323.6473, 1345.6280, 1361.6097, 1365.6708, 1377.5960, 1393.5844, 1395.7311, 1411.7256, 1436.7603, 1475.7776, 1476.1123, 1557.8703, 1636.7539, 1707.8181, 1718.7880, 1732.8009, 1734.7794, 1740.7760, 1744.8013, 1748.7978, 1754.7913, 1756.7547, 1770.7537, 1772.7239, 1838.9638, 1851.8058, 1862.8756, 1876.8772, 1921.8557, 1924.8861, 1941.8985, 1948.8578, 1967.8661, 2005.8049, 2118.8044, 2170.8299, 2217.9647, 2234.8584, 2255.8811, 2265.2040, 2277.1368, 2566.1425, 2618.1737, 2625.1754, 2636.1875, 2663.1955, 2682.2062, 2695.2126, 2708.2152, 2720.1459, 2724.2164, 3347.7264

Search Parameters

Type of search : MS/MS Ion Search
Enzyme : Trypsin
Fixed modifications : Carbamidomethyl (C)
Variable modifications : Oxidation (M)
Mass values : Monoisotopic
Protein Mass : Unrestricted
Peptide Mass Tolerance : ± 100 ppm
Fragment Mass Tolerance: ± 0.7 Da
Max Missed Cleavages : 1
Instrument type : MALDI-TOF-TOF
Query1 (817.1395,1+) : <no title>
Query2 (837.1000,1+) : <no title>
Query3 (839.1118,1+) : <no title>
Query4 (852.3774,1+) : <no title>
Query5 (855.0882,1+) : \\PROTEOMICS1\D\DATA\2105\210505_TLIEPOL_1_68252-68266\68252_68266_LPARDO\0_G12\1\855.0882.LIFT\1SREF\PDATA\1\1R
Query6 (859.0799,1+) : <no title>
Query7 (861.0964,1+) : <no title>
Query8 (868.5738,1+) : <no title>
Query9 (871.0597,1+) : <no title>
Query10 (874.0428,1+) : <no title>
Query11 (877.0680,1+) : <no title>
Query12 (882.6029,1+) : <no title>
Query13 (903.4152,1+) : <no title>
Query14 (905.5111,1+) : <no title>
Query15 (909.3941,1+) : <no title>
Query16 (987.5256,1+) : \\PROTEOMICS1\D\DATA\2105\210505_TLIEPOL_1_68252-68266\68252_68266_LPARDO\0_G12\1\987.5256.LIFT\1SREF\PDATA\1\1R
Query17 (1022.1471,1+) : <no title>
Query18 (1044.1224,1+) : <no title>
Query19 (1064.0902,1+) : <no title>
Query20 (1066.1043,1+) : <no title>
Query21 (1082.0794,1+) : <no title>
Query22 (1149.5023,1+) : <no title>
Query23 (1157.6635,1+) : <no title>
Query24 (1175.6978,1+) : <no title>
Query25 (1201.6609,1+) : <no title>
Query26 (1222.6157,1+) : <no title>
Query27 (1249.1322,1+) : <no title>
Query28 (1252.6490,1+) : <no title>
Query29 (1267.6375,1+) : <no title>
Query30 (1271.1084,1+) : <no title>
Query31 (1283.6283,1+) : <no title>
Query32 (1289.6181,1+) : <no title>
Query33 (1293.6475,1+) : <no title>
Query34 (1305.5904,1+) : <no title>
Query35 (1309.6407,1+) : <no title>
Query36 (1320.6258,1+) : <no title>
Query37 (1323.6473,1+) : <no title>
Query38 (1339.6437,1+) : \\PROTEOMICS1\D\DATA\2105\210505_TLIEPOL_1_68252-68266\68252_68266_LPARDO\0_G12\1\1339.6437.LIFT\1SREF\PDATA\1\1R
Query39 (1345.6280,1+) : <no title>
Query40 (1355.6382,1+) : \\PROTEOMICS1\D\DATA\2105\210505_TLIEPOL_1_68252-68266\68252_68266_LPARDO\0_G12\1\1355.6382.LIFT\1SREF\PDATA\1\1R
Query41 (1361.6097,1+) : <no title>
Query42 (1365.6708,1+) : <no title>
Query43 (1377.5960,1+) : <no title>
Query44 (1393.5844,1+) : <no title>
Query45 (1395.7311,1+) : <no title>
Query46 (1411.7256,1+) : <no title>
Query47 (1436.7603,1+) : <no title>
Query48 (1475.7776,1+) : <no title>
Query49 (1476.1123,1+) : <no title>
Query50 (1557.8703,1+) : <no title>
Query51 (1636.7539,1+) : <no title>
Query52 (1707.8181,1+) : <no title>
Query53 (1718.7880,1+) : \\PROTEOMICS1\D\DATA\2105\210505_TLIEPOL_1_68252-68266\68252_68266_LPARDO\0_G12\1\1718.7880.LIFT\1SREF\PDATA\1\1R
Query54 (1732.8009,1+) : \\PROTEOMICS1\D\DATA\2105\210505_TLIEPOL_1_68252-68266\68252_68266_LPARDO\0_G12\1\1732.8009.LIFT\1SREF\PDATA\1\1R
Query55 (1734.7794,1+) : <no title>
Query56 (1740.7760,1+) : <no title>
Query57 (1744.8013,1+) : <no title>
Query58 (1748.7978,1+) : \\PROTEOMICS1\D\DATA\2105\210505_TLIEPOL_1_68252-68266\68252_68266_LPARDO\0_G12\1\1748.7978.LIFT\1SREF\PDATA\1\1R
Query59 (1754.7913,1+) : <no title>
Query60 (1756.7547,1+) : <no title>
Query61 (1770.7537,1+) : <no title>
Query62 (1772.7239,1+) : <no title>
Query63 (1838.9638,1+) : <no title>
Query64 (1851.8058,1+) : <no title>
Query65 (1862.8756,1+) : <no title>
Query66 (1876.8772,1+) : <no title>
Query67 (1921.8557,1+) : <no title>
Query68 (1924.8861,1+) : <no title>
Query69 (1941.8985,1+) : \\PROTEOMICS1\D\DATA\2105\210505_TLIEPOL_1_68252-68266\68252_68266_LPARDO\0_G12\1\1941.8985.LIFT\1SREF\PDATA\1\1R
Query70 (1948.8578,1+) : <no title>
Query71 (1967.8661,1+) : \\PROTEOMICS1\D\DATA\2105\210505_TLIEPOL_1_68252-68266\68252_68266_LPARDO\0_G12\1\1967.8661.LIFT\1SREF\PDATA\1\1R
Query72 (2005.8049,1+) : <no title>
Query73 (2118.8044,1+) : <no title>
Query74 (2170.8299,1+) : <no title>

```
Query75 (2217.9647,1+) : \\PROTEOMICS1\D\DATA\2105\210505_TLIEPOL_1_68252-68266\68252_68266_LPARDO\0_G12\1\2217.9647.LIFT\1SREF\PDATA\1\1R
Query76 (2234.8584,1+) : \\PROTEOMICS1\D\DATA\2105\210505_TLIEPOL_1_68252-68266\68252_68266_LPARDO\0_G12\1\2234.8584.LIFT\1SREF\PDATA\1\1R
Query77 (2255.8811,1+) : <no title>
Query78 (2265.2040,1+) : <no title>
Query79 (2277.1368,1+) : <no title>
Query80 (2566.1425,1+) : <no title>
Query81 (2618.1737,1+) : <no title>
Query82 (2625.1754,1+) : <no title>
Query83 (2636.1875,1+) : <no title>
Query84 (2663.1955,1+) : <no title>
Query85 (2682.2062,1+) : \\PROTEOMICS1\D\DATA\2105\210505_TLIEPOL_1_68252-68266\68252_68266_LPARDO\0_G12\1\2682.2062.LIFT\1SREF\PDATA\1\1R
Query86 (2695.2126,1+) : <no title>
Query87 (2708.2152,1+) : <no title>
Query88 (2720.1459,1+) : <no title>
Query89 (2724.2164,1+) : <no title>
Query90 (3347.7264,1+) : <no title>
```

Mascot: http://www.matrixscience.com/
