# Supplementary figures and images for: Macrovipera lebetinus obtusa Venom and Its Fractions Affect Human Dermal Microvascular Endothelial and Fibrosarcoma Cells
Source: Int J Mol Sci. 2025 Apr 11;26(8):3601. doi: 10.3390/ijms26083601 (PMC12026461; doi:10.3390/ijms26083601)

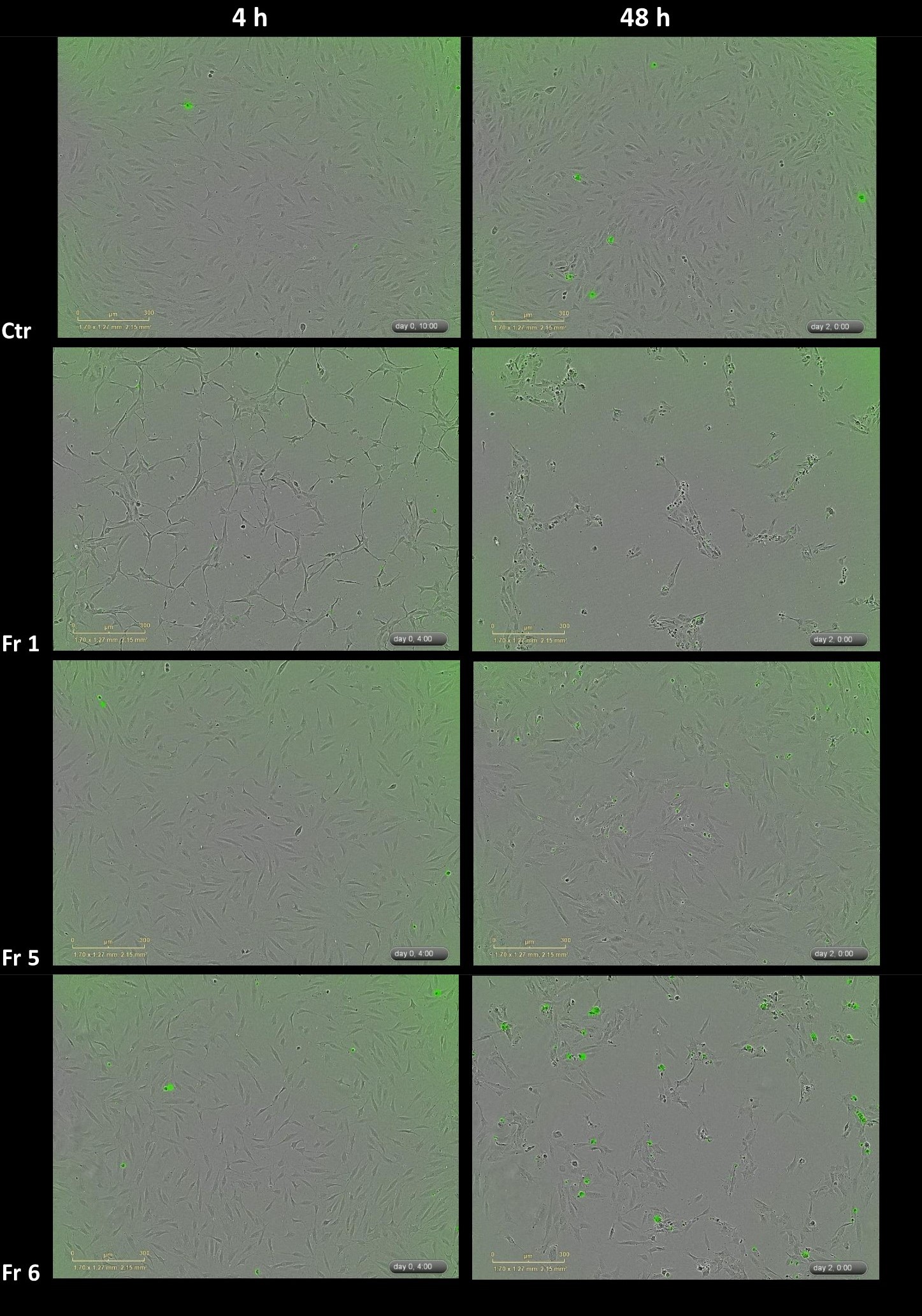

Supplement: Supplementary file 1 [file ijms-26-03601-s001.zip › Supplementary materials/Suppl. Fig 3.jpg]
